# Synthesis and Gelling Abilities of Polyfunctional Cyclohexane-1,2-dicarboxylic Acid Bisamides: Influence of the Hydroxyl Groups

**DOI:** 10.3390/molecules24020352

**Published:** 2019-01-19

**Authors:** Bernat Pi-Boleda, María Campos, Marta Sans, Antonio Basavilbaso, Ona Illa, Vicenç Branchadell, Juan Carlos Estévez, Rosa M. Ortuño

**Affiliations:** 1 Department de Química, Universitat Autònoma de Barcelona, 08193 Cerdanyola del Vallès, Barcelona, Spain; bernat@piboleda.cat (B.P.-B.); marta.sans.valls@cfel.de (M.S.); ona.illa@uab.es (O.I.); 2 CIQUS (Centro Singular de Investigación en Química Biológica y Materiales Moleculares), Departamento de Química Orgánica Universidade de Santiago de Compostela, 15782 Santiago de Compostela, Spain; maria.campos.torrado@rai.usc.es (M.C.); antonio.basavilbaso.glz@gmail.com (A.B.); 3 The Hamburg Center for Ultrafast Imaging (CUI), Luruper Chaussee 149, 22761 Hamburg, Germany

**Keywords:** polyfunctional cycloalkane bisamides, organogelator, self-assembly, chirality, hydrogen bonds

## Abstract

New enantiomerically pure C_16_-alkyl diamides derived from trihydroxy cyclohexane-1,2-dicarboxylic acid have been synthesized from (−)-shikimic acid. The hydroxyl groups in these compounds are free or, alternatively, they present full or partial protection. Their gelling abilities towards several solvents have been tested and rationalized by means of the combined use of Hansen solubility parameters, scanning electron microscopy (SEM), and circular dichroism (CD), as well as computational calculations. All the results allowed us to account for the capability of each type of organogelator to interact with different solvents and for the main mode of aggregation. Thus, compounds with fully protected hydroxyl groups are good organogelators for methanol and ethanol. In contrast, a related compound bearing three free hydroxyl groups is insoluble in water and polar solvents including alcohols but it is able to gelate some low-polarity solvents. This last behavior can be justified by strong hydrogen bonding between molecules of organogelator, which competes advantageously with polar solvent interactions. As an intermediate case, an organogelator with two free hydroxyl groups presents an ambivalent ability to gelate both apolar and polar solvents by means of two aggregation patterns. These involve hydrogen bonding interactions of the unprotected hydroxyl groups in apolar solvents and intermolecular interactions between amide groups in polar ones.

## 1. Introduction

Low molecular weight organogelators (LMWOGs) are soft materials widely used at present in several fields that include products employed as lubricants, cosmetics, drug delivery systems, tissue regeneration materials, biosensors, molecular electronic devices or chiral catalysts [1,2,3,4,5]. Very recently, applications of supramolecular gels as materials for environmental remediation [6] or for practical and eco-friendly oil spill recovery [7,8] have been reported.

The gelling ability of molecules in different solvents has been the subject of a number of studies. However, the ultimate reasons for the hierarchical self-assembling of a gelator in a specific solvent still remain incomplete [9,10]. Therefore, there is a lack of prediction tools. Rationalization of the gelling power of a LMWOG has been attempted based on various solubility indicators [11,12,13,14,15]. Among them, Hansen solubility parameters (HSPs) [13,16] have been applied to elucidate the behavior of LMWOGs towards solvents and to reduce the number of trials usually involved during the identification of a suitable gelator for a particular application [17,18,19].

**Figure 1:**
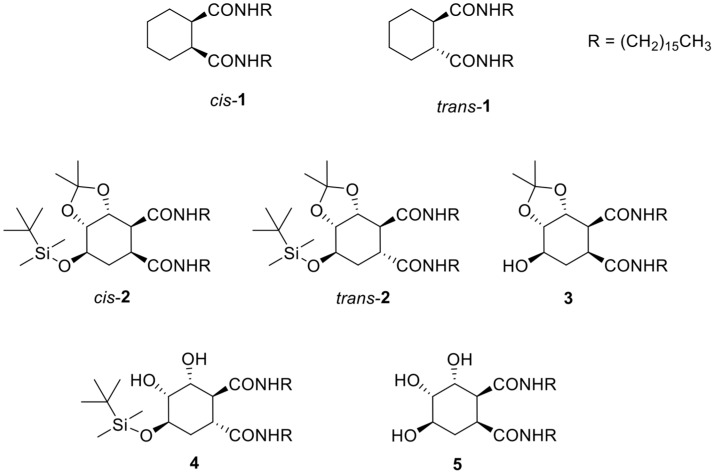
Previously studied LMWOGs (*cis*- and *trans*-**1**) [16] and new LMWOGs investigated in this work (**2**–**5**).

Amides have been reported as functional groups present in some of the simplest LMWOGs that have been described [20]. We have previously developed good LMWOGs of peptide nature based on chiral β-cyclobutane amino acids [21,22], or hybrid peptides presenting a cyclobutane β-amino acid joined in alternation with linear residues [23]. In both cases, a model to explain the self-assembly of the individual molecules to produce the gel was suggested by means of computational calculations. More recently, we reported a combined experimental and computational study to investigate and rationalize the gelling ability of diastereomeric 1,2-disubstituted carbocyclic compounds (**1**, Figure 1) [24]. The influence of the *cis*/*trans* relative configuration of the monomers in their hierarchical self-assembly was analyzed and their gelling behavior compared well with that of *trans*-cyclohexane-1,2-diamine derivatives described by Hanabusa et al. [25] and van Esch et al. [26] suggesting that regiochemistry does not play a relevant role in the gelation process. It was remarkable that chiral aggregates were observed even from meso molecules *cis*-**1** as an example of stochastic symmetry breaking induced by sonication.

Otherwise, regarding polyhydroxylated organogelators, only few results on modified carbohydrate or cholestane derivatives have been described and their ability to gelate organic solvents has been interpreted by using different techniques in each case [27,28,29,30]. Nevertheless, as far as we know, there is neither information in the literature on polyhydroxylated simple cycloalkane derivatives nor on compounds susceptible of additional hydrogen-bonding promoted by further functional groups such as amides.

**Figure 2:**
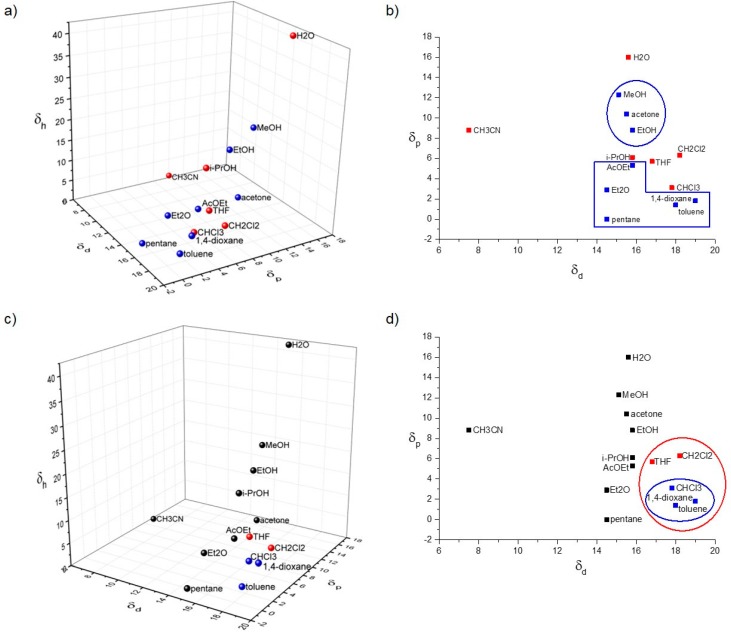
(**a**,**c**) HSPs representation and (**b**,**d**) 2D representation of δ_p_ vs. δ_d_ for the gelation study of compounds **4** (**a**,**b**) and **5** (**c**,**d**). Red: Solvents in which **4** or **5** are soluble; blue: solvents gelled.

In this paper, we describe the investigation of five new polyhydroxylated cyclohexane bisamides (*cis*- and *trans*-**2**, **3**–**5**, Figure 2), prepared from (−)-shikimic acid. All of them bear two long alkyl chains and three hydroxyl groups with different degrees of protection. These new polyfunctional LMWOGs have been compared with their parent compounds *cis*- and *trans*-**1** focusing the attention on the influence of the polar functional groups at the ring, especially the free hydroxyl groups, on their gelling power and aggregation mode in different solvents. With this purpose, the complementary results obtained from several experimental techniques (use of Hansen parameters, SEM and CD spectroscopy) as well as from computational calculations have been taken into account to understand and rationalize the main interactions involved in each case.

## 2. Results and Discussion

### 2.1. Synthesis of Organogelators ***2***–***5***

**Scheme 1:**
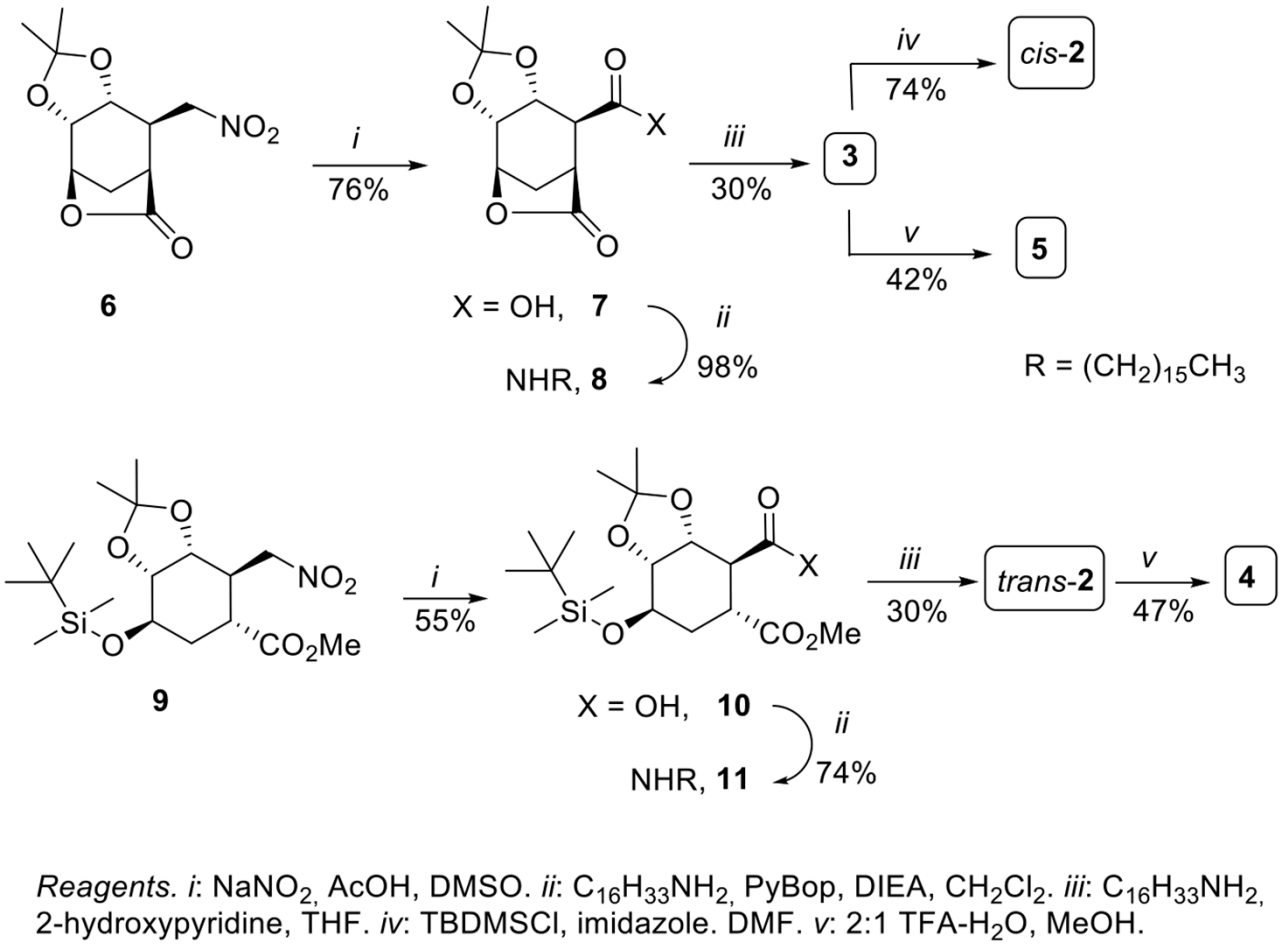
Synthesis of diamides **2**–**5**.

The synthesis of polysubstituted 1,2-cyclohexanedicarboxamides **2**–**5** was achieved from polysubstituted lactone **6** and 2-nitromethylcyclohexanecarboxylic acid methyl ester (**9**). respectively, previously obtained from commercially available (−)-shikimic acid (Scheme 1) [31].

For the preparation of 1,2-cyclohexanedicarboxamides *cis*-**2**, **3** and **5**, lactone **6** was transformed into acid **7** in 76% yield through a Nef reaction conducted under remarkably mild conditions, with sodium nitrite and acetic acid using dimethylsulfoxide as solvent and carefully controlling the pH (not lower than 3) in order to preserve the acetonide protecting group (Scheme 1). The resulting acid **7** was then condensed with hexadecylamine, using PyBop as coupling agent and diisopropylethylamine (DIEA) as a base, to give amide **8** in 98% yield. Subsequent reaction with a second equivalent of hexadecylamine in the presence of 2-hydroxypyridine afforded diamide **3** in 30% yield. In turn, **3** was transformed into fully protected derivative *cis*-**2** by protection of the free hydroxyl group as tert-butyldimethylsilyl ether by treatment with tert-butyldimethylsilyl chloride and imidazole (74% yield). Alternatively, aqueous trifluoroacetic acid-promoted hydrolysis of the acetonide in **3** afforded product **5** in 42% yield.

Following a similar route, nitroester **9** was transformed into amide **11** in 41% yield, for the two steps. This intermediate afforded diamide trans-**2** in 30% yield, when reacted with hexadecylamine and 2-hydroxypyridine as catalyst. The acetonide in fully protected diamide trans-**2** was removed as described before giving diamide **4** in 47% yield, which surprisingly preserved the silyl ether group that was inert under several conditions.

### 2.2. Gelation Studies

The gelling ability of compounds **2**–**5** was studied using 14 protic or aprotic solvents with different polarity. Compounds *cis*- and *trans*-**2**, **3** and **4** were soluble in water whereas **5** was insoluble; formation of hydrogels was not observed in any case. Results for organic solvents are summarized in Table 1 where data for related compounds *cis*- and *trans*-**1** [24] are also shown for comparison.

The gels produced were stable at room temperature but unstable at 37 °C. Thus, a gel became a solution by heating with a hand. Once the solution was left to cool down to room temperature, the gel was formed again and remained unaltered for weeks (see the Experimental Section for details on the gel preparation).

Compounds **2**–**5** presented a different behavior with respect to *cis*- and *trans*-**1**, as expected, clearly indicating that the additional substitution of the ring plays a significant role. Compounds *cis*- and *trans*-**2**, and **4** are able to gelate alcohols contrariwise to *cis*- and *trans*-**1** that are insoluble as well as trihydroxylated compound **5**. In addition, *cis*-**2** also promotes the formation of gels in pentane, ethyl acetate and isopropanol although with higher mgc (minimum gelation concentration) values. Nevertheless, the differences between these diastereoisomers are not very remarkable in contrast with the behavior of isomers **1**, being *trans*-**1** a much better gelator than the *cis* diastereomer (Table 1) [24].

Compound **3** is a bad organogelator because it is soluble in nearly all solvents tested, except in 1,4-dioxane and toluene, but it exhibits very high mgc values. Compound **3** is related to *cis*-**2** but with a free hydroxyl group; comparing their behavior, it seems clear that having an unprotected alcohol disfavors the gelling ability especially in methanol and ethanol. Compound **4**, bearing two free hydroxyl groups, gelates a large variety of solvents of very different dielectric constants and it is a good organogelator for acetone. Comparison with *trans*-**2** suggests that the structural features of **4** exert a significant influence on its properties allowing it to gelate plenty more solvents. Compound **5** has all three hydroxyl groups unprotected. Its behavior is similar to that of disubstituted cyclohexanes *cis*- and *trans*-**1** as it forms gels in 1,4-dioxane, toluene and chloroform and it is insoluble in most other solvents (except in THF and dichloromethane), although the mgc values are significantly higher for **5**. It is noteworthy that, although this compound bears three free hydroxyl groups, it is insoluble in all the alcohols tested (methanol, ethanol and isopropanol) as well as in water. Some rational behind these observations will be discussed below.

### 2.3. Hansen Solubility Parameters (HSPs)

Inspection of the data in Table 1 makes evident that the gelling properties observed for the studied LMWOGs do not only depend on the dielectric constant of the solvents but also other factors need to be considered for their understanding.

Plotting the HSPs of each solvent, a 3D space is generated and the solvents are sorted over the space depending on three parameters: δ_d_, which accounts for dispersive interactions that dominate for low polarity solvents; δ_p_, which is related to polar interactions, and δ_h_ that arises from hydrogen bonding interactions. The representation of the HSPs for **4** shows two areas of solvents corresponding to those gelated by this organogelator, which are highlighted in blue, and those in red in which it is soluble (Figure 2a,b). In order to better observe the two areas, the plane δ_p_-δ_d_ was also represented showing that there are two clusters of gelling solvents which can be related to two different aggregation patterns. One area corresponds to apolar solvents with low δ_p_ and high δ_d_ and the other to aprotic (acetone) or protic (alcohols) polar solvents, with higher δ_p_ and also higher δ_h_ in the case of methanol and ethanol (Figure 2a). These results suggest that **4** could interact with the solvent both through the unprotected hydroxyl groups and through hydrogen bonding between the amide groups. These facts resulting in two types of aggregates as corroborated by SEM (see below).

Despite bearing three unprotected hydroxyl groups, compound **5** is insoluble in polar solvents, especially in alcohols and also in water. One could have expected that the interaction of the three free hydroxyl groups of **5** should be favorable for solubility or gelation through hydrogen bonding. Instead, as shown in Figure 2, compound **5** is only soluble in apolar solvents while the solvents gelled are clustered in the region with the highest δ_d_ and lowest δ_p_ in the HSPs space (Figure 2d). This result suggests that the hydroxyl groups in **5** do not interact with solvents but they are preferably involved in inter-gelator interactions. Only few examples on the fact that gelling solvents for polyhydroxylated compounds are clustered in a region of lower δ_p_ and δ_h_ but high δ_d_ have been described in the literature [17]. These observations were fully supported by the results of computational calculations and CD (see below).

### 2.4. Scanning Electron Microscopy

**Figure 3:**
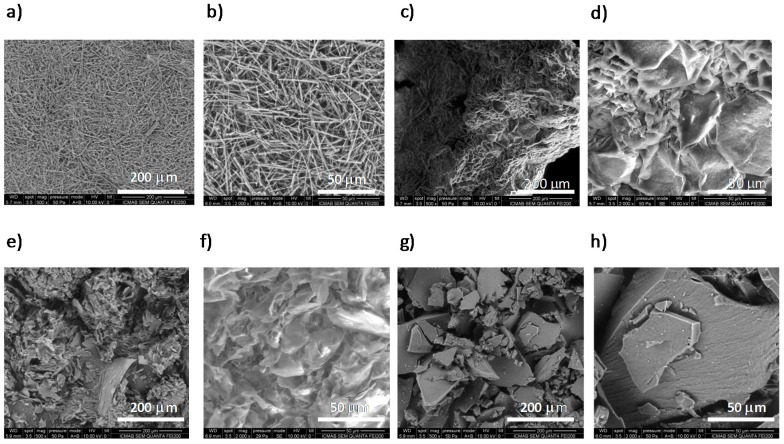
SEM images of xerogels at the mgc of: (**a**,**b**) *cis*-**2** from methanol; (**c**,**d**) *trans*-**2** from methanol; (**e**,**f**) **4** from acetone; (**g**,**h**) **4** from pentane, at two magnifications each (200 and 50 μm).

SEM experiments were carried out to investigate the morphology of some of the gels produced at the mgc for each compound. The micrographs were taken from the corresponding xerogels and selected examples are shown in Figure 3.

SEM images show that compounds *cis*- and *trans*-**2** present different morphologies despite having a similar gelling behavior in methanol. Compound *cis*-**2** forms fibers of different lengths, some of which are very long (around 200 μm) (Figure 3a,b). Its diastereoisomer, *trans*-**2**, also forms big aggregates but in this case in the shape of fibrous platelets (Figure 3c,d). Therefore, this confirms that in this case, as well as for diastereomers *cis*/*trans*-**1**, [24] the *cis*/*trans* stereochemistry plays a role on the pattern of aggregation.

Compound **4** was studied in two different solvents, acetone (Figure 3e,f) and pentane (Figure 3g,h). The appearance of the micrographs is quite different; in acetone the aggregates form disorganized shapes while in pentane the compound forms clear platelets. Therefore, the presence of the free hydroxyl groups also influences the morphology of aggregates in different solvents, which agrees with the results from consideration of the Hansen solubility parameters as discussed above.

### 2.5. Computational Calculations and Circular Dichroism

In earlier studies of other LMWOGs, we used IR and ^1^H-NMR spectroscopies to gain information about the aggregation process [21,22,23]. Nevertheless, in the present case in which different groups can contribute to the gel formation, we thought that the combined results from computational calculations and CD spectroscopy would be better to provide an overall view and a more reliable interpretation. Indeed, CD allowed us to obtain information about the hierarchical organization of the molecules in the aggregates and to corroborate the predictions from calculations that, in turn, led us to better understand the structure of the gels and their formation mode. Computational calculations of compounds **2**–**5** were carried out using the M06-2X/6-31G(d) level of theory for the optimization of the geometry of the monomer and the tetramer and using minimizations of the energy with molecular mechanics for the structure of the hexamer and the octamer (see Supplementary Materials for details).

**Figure 4:**
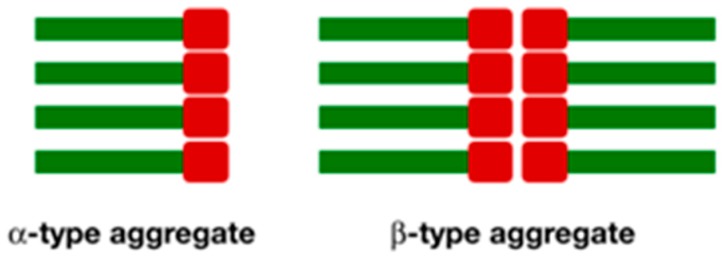
Cartoon representing the spatial disposition of monomers in α- and β-aggregates.

By means of calculations we found that monomers can aggregate according to two types of interactions. The first one, which is the only observed for **2** and **3**, occurs through the formation of –NH···OC– hydrogen bonds in one dimension (1-D) involving the amide groups (α-type aggregates). In turn, monomers with free hydroxyl groups can interact by additional –OH···OH– hydrogen bonding to give β-type aggregates. These aggregates formed by two kinds of directional bonding interactions would be dimers of α-ones (Figure 4) and have been predicted for gelators **4** and **5**.

**Figure 5:**
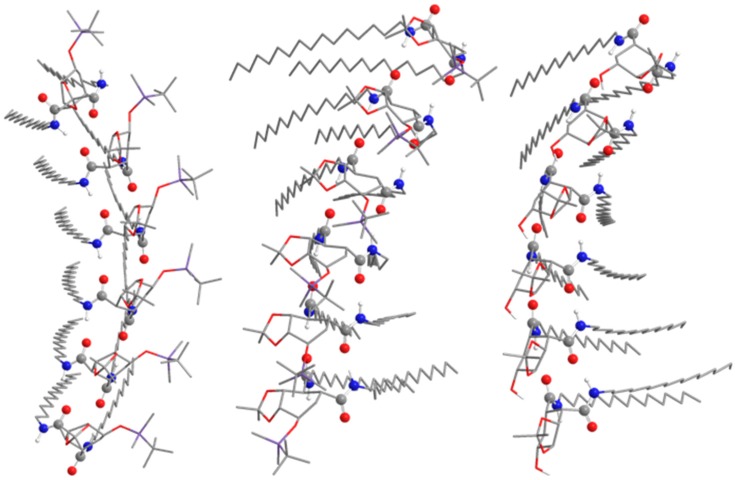
Front views of central 6 molecules in octameric 1-D aggregates (amide hydrogen-bonds) of *cis*- and *trans*-**2**, and **3**. Non-polar hydrogen atoms have been omitted for clarity. Atoms in amide groups have been represented with red (oxygen), blue (nitrogen) and grey (carbon) spheres.

Predicted structures of the octamers from compounds *cis*- and *trans*-**2**, and **3** are shown in Figure 5. As it can be observed, due to the presence of the TBDMS group and the relative *cis* configuration of the two amide groups, compound *cis*-**2** shows a significantly curved vertical aggregate. Otherwise, compounds *trans*-**2** and **3** form a right-handed helical aggregate where the chains are placed in such a way that maximizes the Van der Waals interactions.

**Figure 6:**
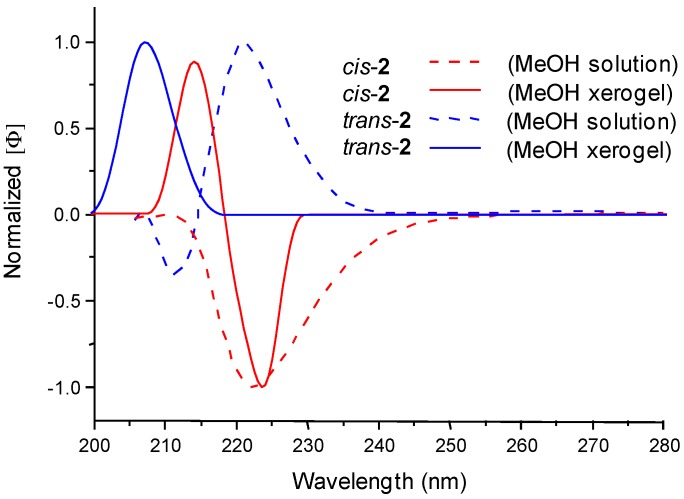
Normalized CD spectra of *cis*-and *trans*-**2**, respectively, in methanol solution (2.4 mM for *cis*-**2** and 2.7 mM for *trans*-**2**) and xerogel at the mgc in methanol in KBr (20 mM) at 25 °C.

CD spectra for *cis*- and *trans*-**2** were recorded both in methanol solution and as xerogels (dry gels) from methanol (Figure 6). In this way, more insight could be obtained on the transfer of chirality from a single molecule to the aggregates.

In solution, *cis*- and *trans*-**2** show a band in the CD spectrum. *cis*-**2** Presents a negative band with a maximum at 222 nm and *trans*-**2** shows a positive band with a maximum at 221 nm and a small negative lobule at 211 nm. The CD spectra of these organogelators in methanol solution were also computed giving predictions in very good agreement with the experimental spectra (see Figure S1 in Supplementary Materials).

For the xerogels from methanol, the shape of the CD spectra is visibly different. For *cis*-**2** as a xerogel from methanol, a bisignate Cotton effect was observed with a positive band at 215 and a negative one at 223 nm with zero crossing at 218 nm. The band of compound *trans*-**2** presents a hypsochromic shift and it has a maximum at 207 nm. This shift is associated to the formation of H-type aggregates in which two or more monomers are arranged on the top of each other, i.e., the stacking due to amide π−π* interactions is oriented in a direction that is roughly perpendicular to the molecular plane; the band is moved to lower wavelengths because the absorption is more energetic than that of the monomer suggesting a strong interaction between the monomers [32,33].

In view of the CD spectra of the xerogels (Figure 6) and their predicted structures (Figure 5), it is important to remark that the bisignate spectrum of *cis*-**2** is in agreement with a curved vertical aggregate whereas the monosignate band for *trans*-**2** suggests a helical structure for its aggregates. This relationship is consistent with previous observations for compounds *cis*- and *trans*-**1**, [24] and also applies for the other xerogels considered in this work.

**Figure 7:**
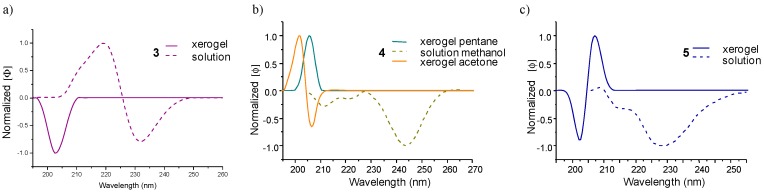
(**a**) Normalized CD spectra of methanol solution (2.47 mM) and xerogel at the mgc from toluene in KBr (20 mM) of **3** at 25 °C; (**b**) Normalized CD spectra of methanol solution (2.56 mM) and xerogels at the mgc in acetone and pentane, respectively, in KBr (20 mM) of **4** at 25 °C; (**c**) Normalized CD spectra of **5** in methanol solution (2.17 mM) and xerogel at the mgc from toluene in KBr (20 mM) at 25 °C.

Compound **3** also differs in the shape of the CD spectra in solution and in the xerogel (Figure 7a). It is bisignate in solution while it shows a band for the xerogel, which is in agreement with the predicted structure of its aggregates that suggests a helical torsion as shown in Figure 5.

Compounds **4** and **5** were also computed. As was hypothesized when the HSPs were analyzed (see above), these compounds could present two different aggregation patterns depending on the predominant interactions in apolar or polar solvents. In α-type aggregates from **4**, the molecules are placed in a zig-zag disposition because of the intermolecular interactions between amide groups (see Figure S3). This type of interactions would be favored in polar solvents and, indeed, the CD spectrum of the xerogel from acetone (prepared at the mgc) is bisignate (Figure 7b).

On the other hand, the predicted β-type structure (Figure S3) shows that compound **4** can self-assemble through hydrogen bonding interactions of the unprotected hydroxyl groups, and then forms an aggregate which shows a torsion that will produce some helicity (right-handed helix). This type of interaction is expected to be predominant in apolar solvents as confirmed by the monosignate CD spectrum of **4** as a xerogel from pentane (Figure 7b).

Therefore, the results from the consideration of HSPs for organogelator **4** are consistent with the CD spectra of the xerogels from polar or apolar solvents and can be explained by the predictions from computational calculations.

**Figure 8:**
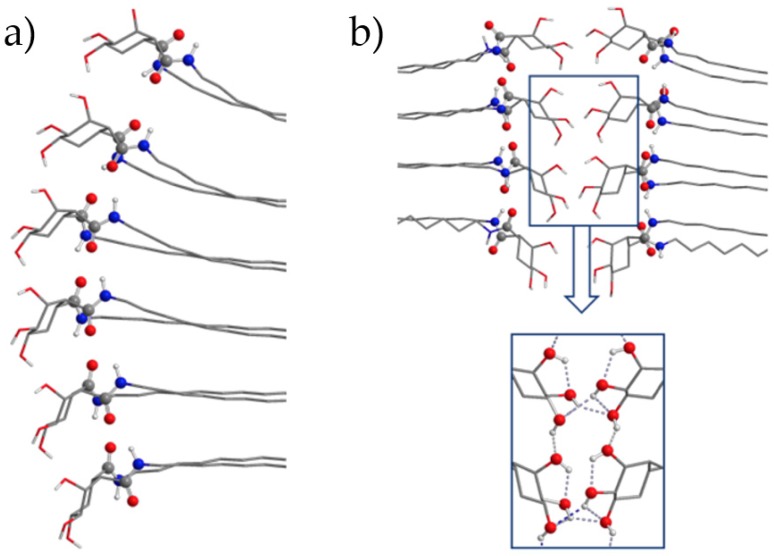
(**a**) side view of central 6 molecules in octameric 1-D aggregate (amide hydrogen-bonds) **5-α**. (**b**) side views of octameric 2-D aggregates (amide and hydroxyl hydrogen-bonds) **5-β**. Non-polar hydrogen atoms have been omitted for clarity. Atoms in amide groups have been represented with red (oxygen), blue (nitrogen) and grey (carbon) spheres.

In a similar manner, compound **5** can aggregate in two different ways. Figure 8 shows the α-type and the β-type structure, where two α-aggregates are interacting with each other through hydrogen bond interactions between the unprotected hydroxyl groups. In this way, the free hydroxyl groups of the molecule would be used to form aggregates avoiding interactions with the solvent. This could explain the insolubility of **5** in alcohols. Moreover, if the polar head of this organogelator was strongly interacting with another polar head, the parts of the molecule available for aggregation would only be the amide groups and the long alkyl chains, which would explain why **5** behaves in a very similar manner to the non-substituted cyclohexane-based compounds *cis*- and *trans*-**1** [24] (see Table 1).

Although compound **5** is rather insoluble in methanol, the low concentration required for CD spectroscopy, 2.17 mM in this case, allowed the spectrum to be recorded in this solvent. Thus, the normalized CD spectra (Figure 7c) show that while compound **5** presents a band in methanol solution, the spectrum for the xerogel is bisignate with zero crossing at 205 nm, which is consistent with the formation of a curved vertical aggregate. However, both predicted α- and β-type structures for compound **5** would be compatible with these results and the CD data does not provide enough evidence to distinguish between them.

**Figure 9:**
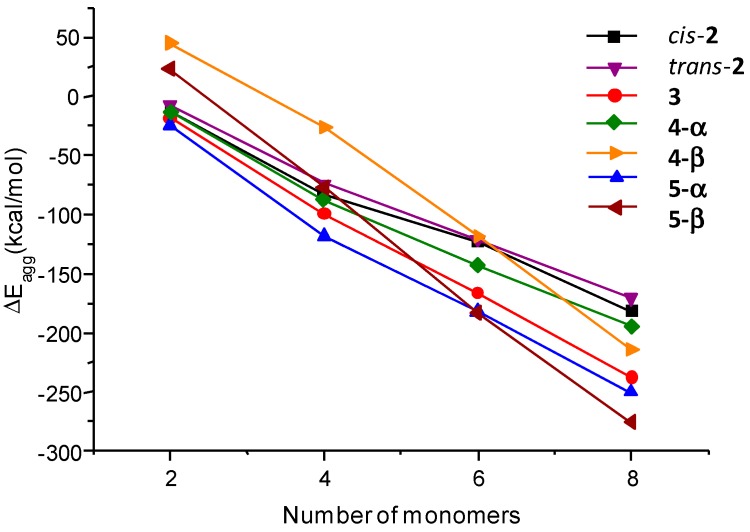
Aggregation energies of compounds **2**–**5**.

These structural features were also supported by the aggregation energy of the formation of the dimeric to the octameric aggregates, which was calculated for compounds **2**–**5** (ΔE_agg_). The aggregation energy per molecule (ΔE_agg_/n) was also considered (see Figure S2). Figure 9 shows the computed values and suggests that the aggregation of all compounds is favorable because the aggregation energy becomes more negative as the number of monomers increases.

Moreover, for compounds that have two different aggregation patterns (**4** and **5**) these data provide more information. Although the formation of a dimer with β-type structure is in both cases not favorable because the aggregation energy of the formation is positive, the formation of the tetrameric, the hexameric and the octameric aggregates becomes favorable. This fact probably means that, first of all, the monomer self-assembles by hydrogen bonding through the amide groups and then, in a second step, the formation of the β−aggregate is favorable. In addition, it is shown that the aggregation energy to form the octamer with the predicted β-type structures for **4** and **5** is more favorable than the formation of the octamers with the predicted α-type structures, allowing the possibility of the coexistence of both of them. For the case of organogelator **5**, the network formed by inter- and intramolecular hydrogen bonding between the unprotected hydroxyl groups seems to be much stronger than the predicted β-type structure of compound **4**. The β-type aggregate predominantly formed in polar media would explain the insolubility of **5**, because the free hydroxyl groups are not able to interact with the solvent.

## 3. Materials and Methods

### 3.1. General Procedures

Melting points were determined using a Kofler Thermogerate apparatus (Reichert, Wien, Austria) and are uncorrected. Specific rotations were recorded on a DIP-370 optical polarimeter (JASCO, Tokio, Japan). Infrared spectra were recorded on a UATR Spectrum two (Perkin Elmer, Waltham, MA, USA, films on NaCl). Nuclear magnetic resonance spectra were recorded on a Mercury 300 apparatus (Varian, Palo Alto, CA, USA). Mass spectra were obtained on a MS 50 TC mass spectrometer (Kratos, Manchester, UK). Thin layer chromatography (tlc) was performed using GF-254 type 60 silica gel (Merck, Kenilworth, NJ, USA) and EtOAc/hexane mixtures as eluents; the tlc spots were visualized with a Hanessian stain (dipping into a solution of 12.5 g of (NH_4_)_4_Mo_7_O_24_·4H_2_O, 5 g of Ce(SO_4_)_2_·4H_2_O and 50 mL of H_2_SO_4_ in 450 mL of H_2_O, and warming). Column chromatography was carried out using Merck type 9385 silica gel.

### 3.2. Experimental Section

*Synthesis of (3aS,4R,7S,8S,8aR)-2,2-dimethyl-6-oxohexahydro-4,7-methano[1,3]dioxolo[4–c]oxepine-8-carboxylic acid* (**7**): Sodium nitrite (0.48 g, 7.00 mmol) and acetic acid (1.33 mL, 23.3 mmol) were added over a solution of lactone 6, prepared according to reference 31, (0.6 g, 2.33 mmol) in DMSO (7.0 mL), under argon. The resulting mixture was stirred at 35 °C for 72 h, 25 mL of HCl aqueous 3 M solution were then added and the reaction allowed to stir at rt for 15 min. H_2_O (50 mL) were added over the reaction, the resulting mixture extracted with Et_2_O (3 × 25 mL), the organic layers dried with anhydrous sodium sulfate, filtered, and evaporated to dryness to give an oil that, purified by flash column chromatography (CH_2_Cl_2_/MeOH 95:5), gave compound **7** (0.43 g, 76% yield) as a yellow oil. *Rf* = 0.2 (CH_2_Cl_2_/MeOH 95:5); ${\left[\alpha \right]}_{20}^{D}$ −5.8 (*c* = 2.1 in CHCl_3_); ^1^H-NMR (300 MHz, CDCl_3_) *δ* = 1.39 (s, 3H, CH_3_); 1.56 (s, 3H, CH_3_); 2.18–2.44 (m, 2H, CH_2_); 3.32 (dd, 1H, *J* = 2.0 Hz, *J* = 4.3 Hz, H7); 3.40–3.49 (m, 1H, H8); 4.43 (dd, 1H, *J* = 4.0 Hz, *J* = 7.6 Hz, H3a); 4.53 (dd, 1H, *J* = 4.2 Hz, *J* = 8.0 Hz, H8a); 4.78 (td, 1H, *J* = 1.5 Hz, *J* = 1.7 Hz, *J* = 4.0 Hz, H4); 5.13 (bs, 1H, CO_2_H) ppm; ^13^C-NMR (75 MHz, CDCl_3_) *δ* = 23.4 (CH_2_); 24.5 (CH_3_); 26.0 (CH_3_); 34.0 (CH); 44.6 (CH); 72.8 (CH); 76.9 (CH); 114.4 (C); 173.2 (C); 176.4 (C) ppm; IR: *ν* = 3452, 1766, 1701, 1210, 1078 cm^−1^; HRMS (ESI^+^) calcd *m*/*z* for C_11_H_14_NaO_6_ [M + Na]^+^: 265.0688, found: 265.0684.

*Synthesis of (3aS,4R,7S,8S,8aR)-N-hexadecyl-2,2-dimethyl-6-oxohexahydro-4,7-methano[1,3]-dioxolo[4–c]oxepin-8-carboxamide* (**8**): PyBOP (0.78 g, 0.15 mmol) and DIEA (0.11 mL, 0.63 mmol) were added over a solution of acid **7** (40 mg, 0.10 mmol), in anhydrous CH_2_Cl_2_ (1.4 mL) and the resulting mixture was stirred for 15 min at rt, under argon. Hexadecylamine (0.124 g, 0.52 mmol) in anhydrous CH_2_Cl_2_ (1.4 mL) and anhydrous DMF (0.5 mL) was then added and the reaction stirred for 2 h at rt. The mixture was then evaporated to dryness, the resulting residue dissolved in CH_2_Cl_2_ (10 mL), extracted with saturated aqueous solution of citric acid (3 × 5 mL), saturated aqueous solution of NaHCO_3_ (3 × 5 mL) and H_2_O (10 mL). The organic solution was dried with anhydrous sodium sulfate and evaporated to give a solid, which was purified by flash column chromatography (CH_2_Cl_2_/MeOH 95:5) to afford compound **8** (50 mg, 98% yield) as a yellow solid. *Rf* = 0.3 (CH_2_Cl_2_/MeOH 95:5); m.p. 209–211 °C (MeOH); ${\left[\alpha \right]}_{20}^{D}$ +15.2 (*c* = 1.0 in CHCl_3_); ^1^H-NMR (300 MHz, CDCl_3_) *δ* = 0.86 (t, 3H, *J* = 6.6 Hz, CH_3_); 1.22–1.29 (m, 26H, 13 × CH_2_), 1.37 (s, 3H, CH_3_); 1.44–1.49 (m, 2H, CH_2_); 1.54 (s, 3H, CH_3_); 2.21–2.27 (m, 2H, CH_2_); 3.03 (dd, 1H, *J* = 4.1 Hz, *J* = 1.9 Hz, H8); 3.10 (ddd, 1H, *J* = 9.9 Hz, *J* = 6.6 Hz, *J* = 2.0 Hz, H7); 3.22 (q, 2H, *J* = 6.7 Hz, -CH_2_CO); 4.41 (dd, 1H, *J* = 8.1 Hz, *J* = 4.2 Hz, H8a); 4.48 (dd, 1H, *J* = 8.0 Hz, *J* = 4.1 Hz, H3a); 4.75 (td, 1H, *J* = 3.9 Hz, *J* = 1.9 Hz, H4); 5.69 (t, 1H, *J* = 5.7 Hz, NH) ppm; ^13^C-NMR (75 MHz, CDCl_3_) *δ* = 14.2 (CH_3_); 22.8 (CH_2_); 23.0 (CH_2_); 24.4 (CH_3_); 25.8 (CH_3_); 27.0 (CH_2_); 29.4 (CH_2_); 29.5 (CH_2_); 29.6 (CH_2_); 29.7 (CH_2_); 29.8 (CH_2_); 29.8 (CH_2_); 32.0 (CH_2_); 34.5 (CH); 40.1 (CH_2_); 43.9 (CH); 71.8 (CH); 71.9 (CH); 75.0 (CH); 113.3 (C); 171.0 (C); 172.3 (C) ppm; IR: *ν* = 1766, 1645, 1209, 1077 cm^−1^; HRMS (ESI^+^) calcd *m*/*z* for C_27_H_48_N_2_O_5_ [M + H]^+^: 466.3527, found: 466.3537.

*Synthesis of (3aR,4S,5S,7R,7aS)-N,N′-dihexadecyl-7-hydroxy-2,2-dimethylhexahydrobenzo[d][1,3]dioxole-4,5-dicarboxamide* (**3**): 2-Hydroxypyridine (5.8 mg, 0.06 mmol) and hexadecylamine (0.27 g, 1.11 mmol) were added over a solution of carboxamide **8** (47.2 mg, 0.10 mmol) in dry THF (1 mL), under argon. The resulting solution was stirred for 2 h at rt, evaporated to dryness and the amorphous solid obtained purified by flash column chromatography (CH_2_Cl_2_/MeOH, 97:3), to give compound **3** (21.2 mg, 30% yield) as a crystalline white solid. *Rf* = 0.3 (CH_2_Cl_2_/MeOH 97:3); m.p. 150–151 °C; (MeOH); ${\left[\alpha \right]}_{20}^{D}$ +51.3 (*c* = 1.0 in CHCl_3_); ^1^H-NMR (300 MHz, CDCl_3_) *δ* = 0.85–0.89 (m, 6H, 2 × CH_3_); 1.24–1.31 (m, 52H, 26 × CH_2_); 1.38–1.51 (m, 10H, 2 × CH_3_ + 2 × CH_2_); 1.96 (dt, 1H, *J* = 15.1 Hz, *J* = 5.4 Hz, H6); 2.12 (dt, 1H, *J* = 15.1 Hz, *J* = 2.6 Hz, H6′); 2.59 (dd, 1H, *J* = 10.1 Hz, *J* = 4.3 Hz, H5); 3.03–3.37 (m, 5H, H4 + 2 × CH_2_); 4.08–4.13 (m, 1H); 4.32–4.41 (m, 2H); 6.44 (d, 1H, *J* = 10.4 Hz, OH); 6.96 (t, 1H, *J* = 5.7 Hz, NH); 7.33 (t, 1H, *J* = 5.6 Hz, NH) ppm; ^13^C-NMR (75 MHz, CDCl_3_) *δ* = 14.3 (CH_3_); 22.8 (CH_2_); 26.2 (CH_3_); 27.0 (CH_2_); 27.1 (CH_2_); 28.6 (CH_3_); 29.4 (CH_2_); 29.5 (CH_2_); 29.6 (CH_2_); 29.7 (CH_2_); 29.8 (CH_2_); 29.8 (CH_2_); 30.9 (CH_2_); 32.1 (CH_2_); 39.7 (CH_2_); 39.9 (CH_2_); 40.5 (CH); 46.5 (CH); 66.1 (CH); 72.6 (CH); 80.4 (CH); 108.5 (C); 172.4 (C); 174.7 (C) ppm; IR: *ν* = 3327, 1652, 1550, 1468, 1221, 1068 cm^−1^; HRMS (ESI^+^) calcd *m*/*z* for C_43_H_83_N_2_O_5_ [M + H]^+^: 707.6302, found: 707.6291.

*Synthesis of (3aR,4S,5S,7R,7aR)-7-((tert-butyldimethylsilyl)oxy)-N,N-dihexadecyl-2,2-dimethyl-hexahydrobenzo[d][1,3]dioxole-4,5-dicarboxamide* (*cis*-**2**): Imidazole (0.13 g, 1.88 mmol) and TBDMSCl (0.15 g, 0.96 mmol) were added over a stirred solution of amide **3** (0.3 g, 0.42 mmol) in dry DMF (17 mL), under argon. The resulting mixture was stirred for 16 h at rt, quenched with brine (20 mL) and then extracted with EtOAc (32 × 20 mL). The organic layers were dried with anhydrous sodium sulfate, filtered and evaporated. The crude product obtained was purified by flash column chromatography (EtOAc/hexane 1:3) to give compound *cis*-**2** (0.26 g, 74% yield) as a white solid. *Rf* = 0.3 (EtOAc/hexane 1:3); m.p. 82–83 °C (EtOAc); ${\left[\alpha \right]}_{20}^{D}$ −16.1 (*c* = 2.2 in CHCl_3_); ^1^H-NMR (300 MHz, CDCl_3_) *δ* = 0.05 (s, 3H, CH_3_), 0.09 (s, 3H, CH_3_), 0.85–0.90 (m, 15H, (CH_3_)_3_ + 2 × CH_3_), 1.25 (bs, 52H, 26 × CH_2_), 1.34 (s, 3H, CH_3_), 1.39–1.48 (m, 7H, CH_3_ + 2 × CH_2_), 1.37–1.94 (m, 2H, H6 + H6′), 2.92 (t, 1H, *J* = 5.8 Hz, H4), 3.04 (td, 1H, *J* = 7.2 Hz, *J* = 4.9 Hz, H5), 3.17 (ddt, 4H, *J* = 10.5 Hz, *J* = 7.5 Hz, *J* = 4.8 Hz, 22 × NCH_2_), 3.73 (dt, 1H, *J* = 10.3 Hz, *J* = 6.8 Hz), 4.19 (t, 1H, *J* = 6.9 Hz), 4.52 (t, 1H, *J* = 6.4 Hz), 5.94 (t, 1H, *J* = 5.4 Hz, NH), 6.58 (t, 1H, *J* = 5.7 Hz, NH) ppm; ^13^C-NMR (75 MHz, CDCl_3_) *δ* = −4.7 (CH_3_), −4.2 (CH_3_), 14.3 (CH_3_), 18.2 (C), 22.8 (CH_2_), 25.8 (CH_3_), 26.0 (CH_3_), 27.1 (CH_2_), 28.0 (CH_3_), 29.5 (CH_2_), 29.6 (CH_2_), 29.7 (CH_2_), 29.8 (CH_2_), 30.9 (CH_2_), 32.1 (CH_2_), 39.6 (CH_2_), 39.8 (CH), 39.9 (CH_2_), 45.4 (CH), 72.2 (CH), 74.3 (CH), 80.4 (CH), 108.4 (C), 171.4 (C), 173.4 (C) ppm; IR: *ν* = 3287, 1652, 1560, 1112, 837 cm^−1^; HRMS (ESI^+^) calcd *m*/*z* for C_49_H_97_N_2_O_5_Si [M+H]^+^: 821.7161, found: 821.7129.

*Synthesis of (1S,2S,3R,4S,5R)-N,N-dihexadecyl-3,4,5-trihydroxycyclohexane-1,2-dicarboxamide* (**5**): H_2_O (9.0 mL) and TFA (18.0 mL) were added over a stirred solution of diamide **3** (0.3 g, 0.42 mmol) in MeOH (10 mL) and the obtained mixture was stirred for 12 h at rt. The solvent was removed under vacuum, the residue coevaporated with toluene (3 × 10 mL) and the crude obtained purified by flash column chromatography (CH_2_Cl_2_/MeOH 93:7) to give dicarboxamide **5** (0.13 g, 42% yield) as a white solid. *Rf* = 0.3 (CH_2_Cl_2_/MeOH 93:7); m.p. 166.0–166.6 °C (MeOH); ${\left[\alpha \right]}_{20}^{D}$ −27.3 (*c* = 2.0 in CHCl_3_); ^1^H-NMR (300 MHz, THF-d8) *δ* = 0.88 (t, 6H, *J* = 6.6 Hz, 2 × CH_3_), 1.25–1.51 (m, 56H, 28 × CH_2_), 1.79–1.96 (m, 2H, H6 + H6′), 2.83–3.17 (m, 6H, 2 × NCH_2_ + H1 + H2), 3.34–4.39 (m, 6H, H3 + H4 + H5 + 3 × OH), 7.33 (bs, 1H, NH), 7.69 (t, 1H, *J* = 5.6 Hz, NH) ppm; ^13^C-NMR (75 MHz, THF-d8, main rotamer) *δ* = 14.6 (CH_3_), 23.7 (CH_2_), 28.1 (CH_2_), 28.2 (CH_2_), 30.3 (CH_2_), 30.5 (CH_2_), 30.6 (CH_2_), 30.7 (CH_2_), 30.8 (CH_2_), 30.8 (CH_2_), 33.0 (CH_2_), 40.1 (CH_2_), 40.5 (CH_2_), 42.8 (CH), 46.4 (CH), 69.4 (CH), 70.9 (CH), 75.3 (CH), 172.7 (C), 175.8 (C) ppm; IR: *ν* = 3328, 1649, 1542, 1068 cm^−1^; HRMS (ESI^+^) calcd *m*/*z* for C_40_H_79_N_2_O_5_ [M + H]^+^: 667.5984, found: 667.5976.

*Synthesis of (3aR,4S,5R,7R,7aR)-7-((tert-butyldimethylsilyl)oxy)-5-(methoxycarbonyl)-2,2-dimethyl-hexa-hydrobenzo[d][1,3]dioxole-4-carboxylic acid* (**10**): Sodium nitrite (26 mg, 0.37 mmol) and acetic acid (71 μL, 1.24 mmol) were added over a solution of nitroester **9** [20] (50 mg, 0.12 mmol) in DMSO (0.6 mL), under argon. The resulting mixture was stirred at 35 °C for 72 h, 15 mL of H_2_O and deactivated Dowex-50 resin were then added until pH = 3 and the reaction allowed to stir for 15 min at rt. H_2_O (20 mL) were added over the reaction, the resulting mixture extracted with Et_2_O (3 × 10 mL), the organic layers dried with anhydrous sodium sulfate, filtered, and evaporated to dryness to give an oil that, purified by flash column chromatography (CH_2_Cl_2_/MeOH 95:5), gave compound **10** (30 mg, 55% yield) as a clear oil. *Rf* = 0.3 (CH_2_Cl_2_/MeOH 95:5); ${\left[\alpha \right]}_{20}^{D}$ +4.1 (*c* = 1.2 in CHCl_3_): ^1^H-NMR (300 MHz, CDCl_3_) *δ* = 0.08 (bs, 6H, 2 × CH_3_); 0.88 (bs, 9H, (CH_3_)_3_); 1.35 (s, 3H, CH_3_); 1.52 (s, 3H, CH_3_); 1.85–1.89 (m, 2H, CH_2_); 2.86 (dd, 1H, *J* = 10.7 Hz, *J* = 8.2 Hz), 2.94–3.13 (m, 1H, H5), 3.68 (s, 3H, OCH_3_); 3.99 (dd, 1H, *J* = 5.0 Hz, *J* = 2.9 Hz), 4.21 (q, 1H, *J* = 3.2 Hz), 4.40 (dd, 1H, *J* = 5.0 Hz, *J* = 8.3 Hz); 9.32 (bs, 1H, OH) ppm; ^13^C-NMR (75 MHz, CDCl_3_) *δ* = −4.9 (CH_3_); −4.8 (CH_3_); 18.1 (C); 25.8 (CH_3_); 26.2 (CH_3_); 28.2 (CH_3_); 30.9 (CH_2_); 37.0 (CH); 47.4 (CH); 52.3 (CH_3_); 67.1 (CH); 74.6 (CH); 76.9 (CH); 109.7 (C); 174.6 (C); 178.9 (C) ppm; IR: *ν* = 3375, 1735, 1258, 1100, 810 cm^−1^; HRMS (ESI^+^) calcd *m*/*z* for C_18_H_32_NaO_7_Si (M + Na)^+^: 411.1815, found: 411.1809.

*Synthesis of Methyl (3aR,4S,5R,7R,7aR)-7-((tert-butyldimethylsilyl)oxy)-4-(hexadecyl-carbamoyl)-2,2-dimethylhexahydrobenzo[d][1,3]dioxole-5-carboxylate* (**11**): A mixture containing a solution of acid **10** (50 mg, 0.13 mmol), PyBOP (0.10 g, 0.19 mmol) and DIEA (0.14 mL, 0.80 mmol) in anhydrous CH_2_Cl_2_ (1.8 mL) was stirred for 15 min at rt, under argon. Hexadecylamine (0.15 g, 0.52 mmol) in anhydrous CH_2_Cl_2_ (1.8 mL) and anhydrous DMF (0.5 mL) were then added. The reaction was stirred for 2 h at rt, evaporated to dryness, the resulting residue dissolved in CH_2_Cl_2_ (10 mL), extracted with saturated aqueous solution of citric acid (3 × 5 mL), saturated aqueous solution of NaHCO_3_ (3 × 5 mL) and water (10 mL). The organic layer was then dried with anhydrous sodium sulfate and evaporated to give a yellow solid, which on purification by flash column chromatography (CH_2_Cl_2_/MeOH 99:1) afforded compound **11** (60 mg, 74% yield) as a clear oil. *Rf* = 0.3 (CH_2_Cl_2_/MeOH 99:1); ${\left[\alpha \right]}_{20}^{D}$ +48.3 (*c* = 1.0 in CHCl_3_); ^1^H-NMR (300 MHz, CDCl_3_) *δ* = 0.07 (s, 3H, CH_3_); 0.08 (s, 3H, CH_3_); 0.84–0.89 (m, 12H, -(CH_3_)_3_ + CH_3_); 1.24–1.29 (m, 26H, 13 × CH_2_); 1.36 (s, 3H, CH_3_); 1.42–1.52 (m, 2H, CH_2_); 1.56 (s, 3H, CH_3_); 1.77–1.83 (m, 2H, H6 + H6′); 2.58 (dd, 1H, *J* = 11.9 Hz, *J* = 9.8 Hz, H4); 3.00 (ddd, 1H, *J* = 12.4 Hz, *J* = 8.9 Hz, *J* = 6.7 Hz, H5); 3.21 (q, 2H, *J* = 6.8 Hz, N-CH_2_); 3.68 (s, 3H, OCH_3_); 3.98 (dd, 1H, *J* = 4.9 Hz, *J* = 2.6 Hz); 4.20–4.25 (m, 2H); 6.19 (t, 1H, *J* = 5.8 Hz, NH) ppm; ^13^C-NMR (75 MHz, CDCl_3_) *δ* = −4.9 (CH_3_); −4.7 (CH_3_); 14.2 (CH_3_); 18.1 (C); 22.8 (CH_2_); 25.8 (CH_3_); 26.5 (CH_3_); 27.0 (CH_2_); 28.7 (CH_3_); 29.4 (CH_2_); 29.5 (CH_2_); 29.7 (CH_2_); 29.8 (CH_2_); 29.8 (CH_2_); 32.1 (CH_2_); 32.3 (CH_2_); 36.3 (CH); 39.6 (CH_2_); 48.3 (CH); 52.0 (CH_3_); 67.3 (CH); 75.1 (CH); 77.3 (CH); 109.4 (C); 172.5 (C); 176.0 (C) ppm; IR: *ν* = 3322, 1740, 1652, 1249, 1070, 837 cm^−1^; HRMS (ESI^+^) calcd m/z for C_34_H_66_NO_6_Si (M + H)^+^: 612.4659, Found: 612.4654.

*Synthesis of (3aR,4S,5R,7R,7aR)-7-((tert-butyldimethylsilyl)oxy)-N,N-dihexadecyl-2,2-dimethyl-hexahydro-benzo[d][1,3]dioxole-4,5-dicarboxamide* (*trans*-**2**): 2-Hydroxipyridine (78 mg, 0.49 mmol) and hexadecylamine (2.17 g, 8.99 mmol) were added over a solution of amide **11** (0.5 g, 0.82 mmol), in dry THF (10.2 mL), under argon. The reaction mixture was refluxed for 12 h, the solvent removed under vacuum and the obtained residue extracted with CH_2_Cl_2_ (3 × 50 mL). The organic layers were dried with anhydrous sodium sulfate, filtered and evaporated *in vacuo* to give a residue, which on purification by flash column chromathography (CH_2_Cl_2_/MeOH 99:1) gave compound *trans*-**2** (0.2 g, 30% yield) as a white solid. *Rf* = 0.4 (CH_2_Cl_2_/MeOH 99:1); m.p. 63–64 °C (MeOH); ${\left[\alpha \right]}_{20}^{D}$ +31.0 (*c* = 1.0 in CHCl_3_); ^1^H-NMR (300 MHz, CDCl_3_) *δ* = 0.07 (s, 3H, CH_3_), 0.08 (s, 3H, (CH_3_)_3_) 0.84–0.89 (m, 15H, (CH_3_)_3_ + 2 × CH_3_), 1.24 (bs, 52H, 26 × CH_2_), 1.34 (s, 3H, CH_3_), 1.41–1.47 (m, 4H, 2 × CH_2_), 1.53 (s, 3H, CH_3_), 1.63 (dt, 1H, *J* = 14.0 Hz, *J* = 3.8 Hz, H6), 2.00 (ddd, 1H, *J* = 14.1 Hz, *J* = 11.3 Hz, *J* = 2.7 Hz, H6′), 2.45 (dd, 1H, *J* = 11.7 Hz, *J* = 9.3 Hz, H4), 2.70 (td, 1H, *J* = 11.5 Hz, *J* = 3.5 Hz, H5), 3.15–3.19 (m, 4H, 2 × N-CH_2_), 3.98 (dd, 1H, *J* = 5.2 Hz, *J* = 2.9 Hz, H7), 4.25 (q, 1H, *J* = 3.0 Hz, H3a), 4.32 (dd, 1H, *J* = 9.3 Hz, *J* = 5.1 Hz, H7a), 5.76 (t, 1H, *J* = 5.8 Hz, NH), 5.93 (t, 1H, *J* = 5.7 Hz, NH) ppm; ^13^C-NMR (75 MHz, CDCl_3_) *δ* = −4.8 (CH_3_), −4.7 (CH_3_), 14.2 (CH_3_), 18.2 (C), 22.8 (CH_2_), 25.9 (CH_3_), 26.3 (CH_3_), 27.0 (CH_2_), 28.6 (CH_3_), 29.5 (CH_2_), 29.5 (CH_2_), 29.6 (CH_2_), 29.7 (CH_2_), 29.8 (CH_2_), 29.8 (CH_2_), 32.1 (CH_2_), 38.8 (CH), 39.7 (CH_2_), 39.8 (CH), 50.1 (CH_2_), 67.6 (CH), 75.6 (CH), 77.3 (CH), 109.2 (C), 172.7 (C), 174.1 (C) ppm; IR: *ν* = 1653, 1117, 1065, 838 cm^−1^; HRMS (ESI^+^) calcd m/z for C_49_H_97_N_2_O_5_Si (M + H)^+^: 821.7167, found: 821.7169.

*Synthesis of (1R,2S,3R,4R,5R)-5-((tert-butyldimethylsilyl)oxy)-N,N-dihexadecyl-3,4-dihydroxy-cyclohexane-1,2-dicarboxamide* (**4**): Trifluoroacetic acid (7.4 mL) was added over a solution of diamide *trans*-**2** (0.8 g, 0.09 mmol) in a mixture of MeOH (3.7 mL) and H_2_O (3.7 mL) and the resulting mixture was stirred for 12 h at rt. Solvent was removed under vacuum, coevaporated with toluene (3 × 5 mL) and the crude obtained purified by flash column chromatography (CH_2_Cl_2_/MeOH 9:1) to give compound **4** (0.35 g, 47% yield) as a yellow solid. *Rf* = 0.4 (CH_2_Cl_2_/MeOH 90:10); m.p. 96–97 °C (MeOH); ${\left[\alpha \right]}_{20}^{D}$ +7.0 (*c* = 1.7 in CHCl_3_); ^1^H-NMR (300 MHz, CDCl_3_) *δ* = 0.04 (s, 3H, CH_3_), 0.05 (s, 3H, CH_3_), 0.85–0.89 (m, 15H, (CH_3_)_3_ + 2 × CH_3_), 1.17–1.52 (m, 56H, 28 × CH_2_), 2.14 (t, 1H, *J* = 13.1 Hz, H6), 2.71–2.81 (m, 1H, H6′), 2.89–3.26 (m, 6H, H4 + H5 + 2 × N-CH_2_), 3.87 (t, 1H, *J* = 3.3 Hz), 4.08 (q, 1H, *J* = 2.8 Hz), 4.20 (dd, 1H, *J* = 10.5 Hz, *J* = 2.8 Hz), 5.00 (bs, 2H, 2 × OH), 6.11 (t, 1H, *J* = 5.3 Hz, NH), 7.18 (t, 1H, *J* = 5.2 Hz, NH) ppm; ^13^C-NMR (75 MHz, CDCl_3_) *δ* = −4.8 (CH_3_), 14.3 (CH_3_), 18.1 (C), 22.8 (CH_2_), 25.9 (CH_3_), 27.1 (CH_2_), 27.2 (CH_2_), 29.5 (CH_2_), 29.6 (CH_2_), 29.7 (CH_2_), 29.8 (CH_2_), 29.9 (CH_2_), 30.6 (CH_2_), 32.1 (CH_2_), 39.9 (CH_2_), 41.8 (CH), 48.2 (CH), 69.5 (CH), 70.3 (CH), 72.5 (CH), 173.1 (C), 174.5 (C) ppm; IR: *ν* = 3297, 1645, 1119, 1076, 837 cm^−1^; HRMS (ESI^+^) calcd *m*/*z* for C_46_H_93_N_2_O_5_Si (M + H)^+^: 781.6854, found: 781.6855.

*Procedure for gel preparation*: A small amount (5.0 ± 0.1 mg) of bisamide is weighted in a 2 mL transparent-glass vial with septum screw-on cap. When the 5 mg amount of bisamide is soluble in a specific solvent, a new vial containing 10 ± 0.1 mg is prepared and the solubility-gelation checked again. In a second step, a certain volume of solvent to be tested is added and the vial closed. The minimum volume added is 0.05 mL. Then the mixture is heated under the boiling point of the solvent using a balloon system in order to avoid solvent pressure and, once a solution is obtained the mixture is sonicated for 1 to 5 min. For high concentrations and also in some solvents, previous sonication is needed for a good solubilization during heating and sonication time is usually shorter than for diluted gels. Then, the mixture is left to stabilize and to reach room temperature. To state that the mixture is a gel the tube inversion test is done just by turning the vial upside down. If the sample is a gel it does not drop and if it drops a little it can be classified as gel-like mixture. We can also state the mixtures as solutions or insoluble systems. In order to determine the mgc, a new volume of solvent is added to the gel and the process is repeated until no gel is formed: the last volume added determines the mgc. Gels were stable at room temperature and, moreover, they were reversible at the body temperature. Thus, the gel became a solution by heating with a hand. Once the solution was left to cool down at room temperature, the gel was formed again.

*SEM measurements*: SEM images were acquired with Quanta ESEM FEG apparatus equipped with a field emission gun. Wet gels were disposed on a carbon-film-coated copper grid and dried by standing for 30 min on the grid. The resulting xerogels (dry gels) were then introduced into the microscope working at 10 kV and under a pressure of 50 Pa, in most cases (Figure 3a–h), and 29 Pa (Figure 3f).

*Computational details*: A conformational search of each system was carried out using a mixed low mode/torsional sampling [34] with the OPLS-2005 [35] force field implemented in the MacroModel [36] program in order to find and select an approximation of the most stable conformers. The geometries of the lowest energy conformers of each system were optimized using DFT calculations with the Gaussian09 [37] program with the M06-2X [38] functional with the 6-31G(d) basis set. This is a hybrid meta-GGA functional that includes a 54% of exact e × CHange and that was shown to describe correctly non covalent interactions [39]. For all the studied molecules, once the geometry in gas phase is obtained, the dimer is built up and the procedure is repeated. And then, the procedure is repeated again with the tetramer. Once the optimized DFT structure of the tetramer is obtained, the internal dimer of the aggregate is selected to be the model of aggregation to build up the hexamer and the octamer. A minimization of the energy of the hexamer and the octamer is carried out in toluene. Finally, a single point energy calculation in gas phase is carried out to get the energy of each system. Circular dichroism spectra were calculated by taking the optimized structure of the monomer in methanol solution and calculating different excited states with Gaussian09. Representation of the predicted circular dichroism spectra were done using GaussSum software [40].

## 4. Conclusions

Chiral pentasubstituted cyclohexane derivatives **2**–**5** show different gelling behavior depending on the degree of protection of the hydroxyl groups and, to some extent, depending on the relative *cis*/*trans* stereochemistry. Compounds *cis*- and *trans*-**2** differ only in the relative configuration of the two amide groups and both compounds are good gelators for methanol and ethanol but they are very soluble in most of the other solvents tested. Nevertheless, their SEM images reveal the formation of fibers in the case of *cis*-**2** and of platelets in the case of *trans*-**2**. Computational calculations predict the formation of a curved aggregate without a helicity trend for *cis*-**2**, whereas for *trans*-**2** a right handed helical aggregate is predicted. These results are confirmed by the CD spectra that show of a bisignate spectrum for *cis*-**2** and a monosignate positive band for *trans*-**2**, both as xerogels from methanol.

Regarding LMWOGs **3**–**5**, compound **3**, which is polar but with low hydrogen bonding ability, is a bad organogelator for apolar solvents and it is soluble in polar ones; the CD spectrum of its toluene xerogel is in agreement with the computed structure that suggests the formation of a right handed helical aggregate. LMWOG **4**, bearing two hydroxyl groups, presents ambivalent ability since two types of aggregation are predicted by calculations depending on the polarity of solvents: α-type, which is described as a vertical aggregation promoted by intermolecular amide hydrogen bonding, in polar solvents; and β-type, where two molecules are facing head-to-head and then piled vertically, in apolar ones. This solvent-mediated ambivalence is corroborate by the different morphologies shown by SEM images of xerogels form acetone and pentane, respectively, and supported by Hansen parameters and CD. Finally, LMWOG **5** with three hydroxyl groups does not interact with very polar solvents due to the formation of intra and intermolecular hydrogen bonds and, consequently, it is insoluble in alcohols and water; nevertheless, it is able to gelate low polarity solvents by means of non-hydrogen bonding interactions.

## Figures and Tables

**Table 1:** Gelling behavior of previously known LMWOG **1**^16^ and of the new compounds **2**–**5** in common organic solvents ^a, b, c^.

	Pentane	1,4-Dioxane	Toluene	Et_2_O	CHCl_3_	EtOAc	THF	CH_2_Cl_2_	^i^PrOH	Acetone	EtOH	MeOH	CH_3_CN
*cis*-**1**^16^	30	51	100	I	S	I	I	I	I	I	I	I	I
	(48)	(82)	(161)										
	O	O	O										
*trans*-**1**^16^	I	3	7	I	18	I	I	I	I	I	I	I	I
		(5)	(11)		(29)								
		T	T		T								
*cis*-**2**	83	S	S	S	S	100	S	S	100	S	21	17	S
	(101)					(122)			(122)		(26)	(21)	
	C					O			O		O	O	
*trans*-**2**	S	S	S	S	S	S	S	S	S	S	50	16	S
											(61)	(19)	
											T	O	
**3**	I	51	102	S	S	S	S	S	S	S	S	S	S
		(72)	(144)										
		T	T										
**4**	54	61	64	70	S	45	S	S	S	22	56	70	S
	(69)	(78)	(82)	(90)		(58)				(28)	(72)	(90)	
	O	O	T	O		O				O	O	O	
**5**	I	102	82	I	82	I	S	S	I	I	I	I	I
		(193)	(156)		(156)								
		O	T		T								

^a^ Dielectric constant increases from left to right; ^b^ mgc (minimum gelation concentration) in mg mL^−1^. mgc values in mM in parentheses; ^c^ I: insoluble (precipitates before formation of a gel), S: soluble, C: clear, O: opaque, T: translucent.

## Supplementary Materials

The following are available online: ^1^H-NMR and ^13^C-NMR spectra for compounds **7**, **8**, **3**, *cis*-**2**, **5**, **10**, **11**, *trans*-**2** and **4**; Figure S1. Predicted CD spectra of compounds *cis*- and *trans*-2; Figure S2. Calculated aggregation energies per monomer of compounds **2**–**5**; Figure S3. Predicted structures for aggregates 4-α and 4-β; Total energies of aggregates of compounds **2**–**5**; Cartesian coordinates of aggregates of compounds **2**–**5**.
