# Supplementary material for: Synthesis and Gelling Abilities of Polyfunctional Cyclohexane-1,2-dicarboxylic Acid Bisamides: Influence of the Hydroxyl Groups
Source: Molecules. 2019 Jan 19;24(2):352. doi: 10.3390/molecules24020352 (PMC6358840; doi:10.3390/molecules24020352)
Supplement: Supplementary file 1 [file molecules-24-00352-s001.pdf]

Synthesis and Gelling Abilities of Polyfuntional Cyclohexane-1,2-carboxylic Acid Bisamides: Influence of the Hydroxyl Groups

Bernat Pi-Boleda,[a] María Campos,[b] Marta Sans,[a,c] Antonio Basavilbaso,[b] Ona Illa,[a] Vicenç Branchadell,[a] Juan C. Estévez,*[b] Rosa M. Ortuño*[a]

a: Department de Química, Universitat Autònoma de Barcelona, 08193 Cerdanyola del Vallès, Barcelona (Spain)

b: CIQUS (Centro Singular de Investigacón en Química Biológica y Materiales Moleculares) y Departamento de Química Orgánica Universidade de Santiago de Compostela, 15782 Santiago de Compostela (Spain)

c: The Hamburg Center for Ultrafast Imaging (CUI), Luruper Chaussee 149, 2761 Hamburg (Germany)

Supplementary Materials

^{1}H NMR and ^{13}C NMR spectra for compounds:

7, 8, 3, *cis*-**2, 5, 10, 11,** *trans*-**2, 4** S2

Figure S1. Predicted CD spectra of compounds *cis*- and *trans*-**2** S11

Figure S2. Calculated aggregation energies per monomer of compounds **2-5** S11

Figure S3. Predicted structures for aggregates **4-α** and **4-β** S12

Total energies of aggregates of compounds **2-5** S13

Cartesian coordinates of aggregates of compounds **2-5** S14

^{1}H NMR (300 MHz, $CDCl_3$, **7**)

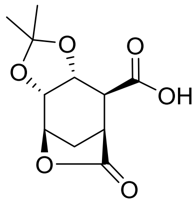

7

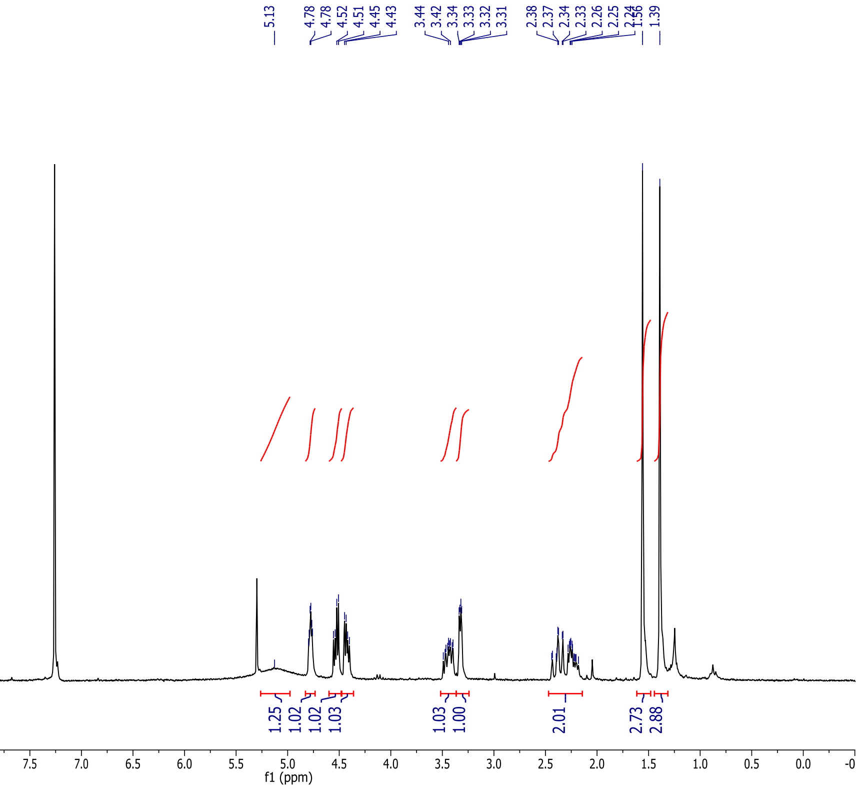

^{13}C NMR (75.0 MHz, CD_3OD, **7**)

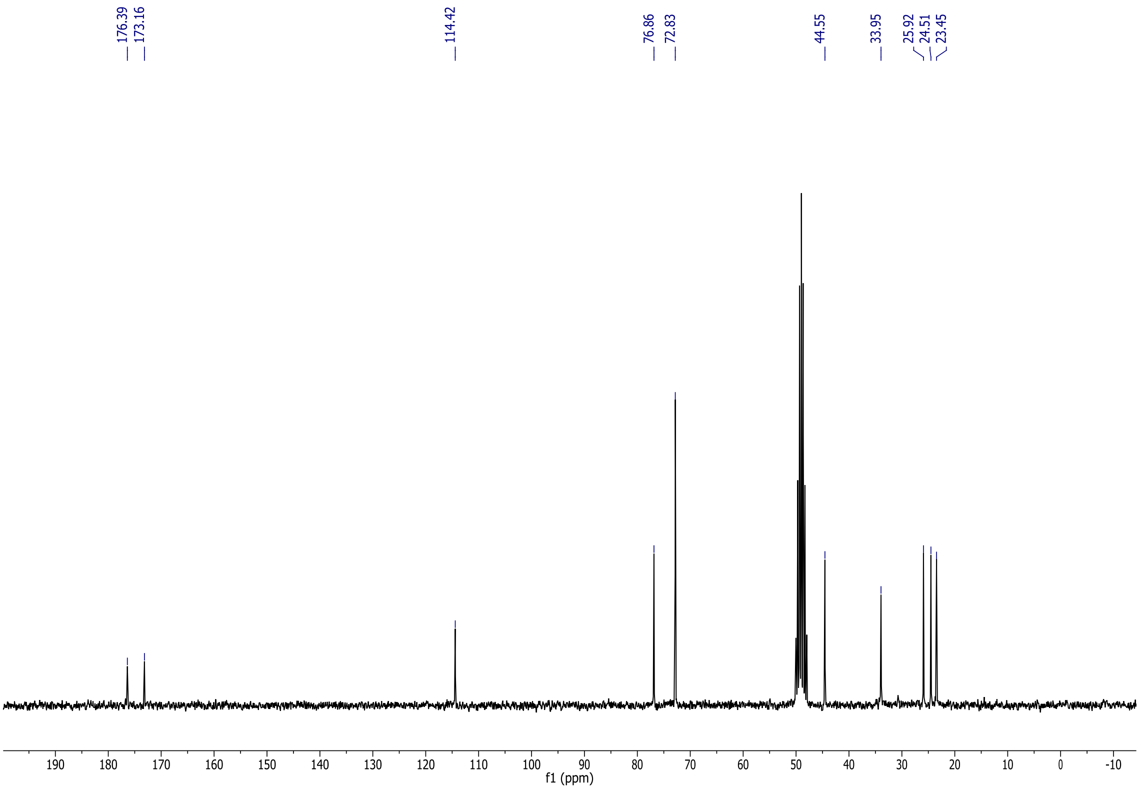

^{1}H NMR (300 MHz, $CDCl_3$, **8**)

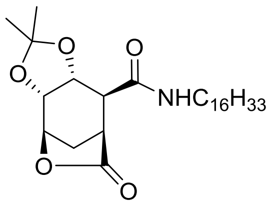

8

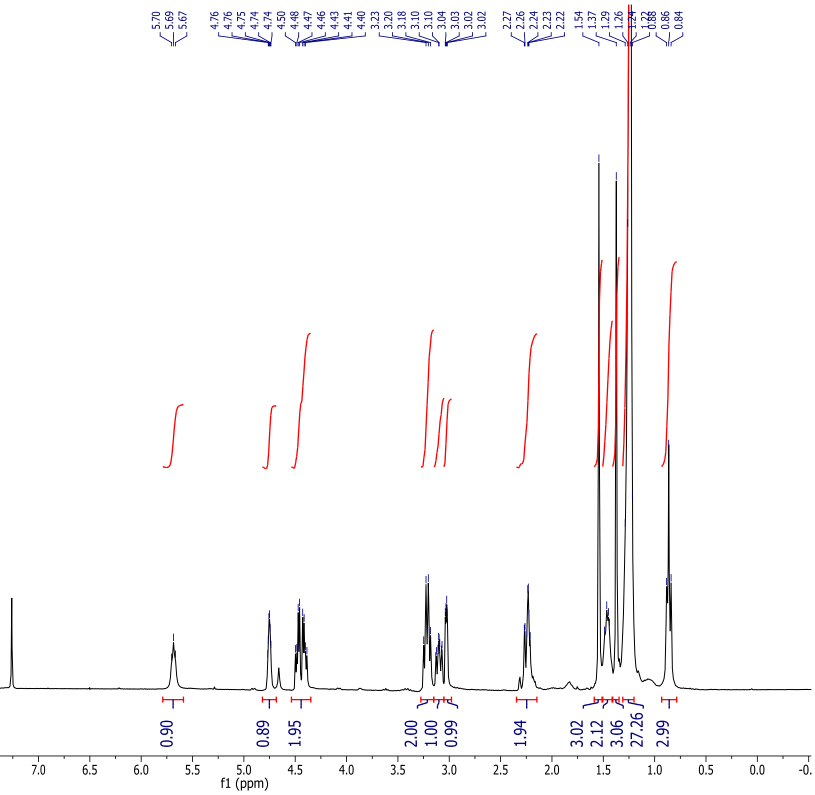

^{13}C NMR (75 MHz, $CDCl_3$, **8**)

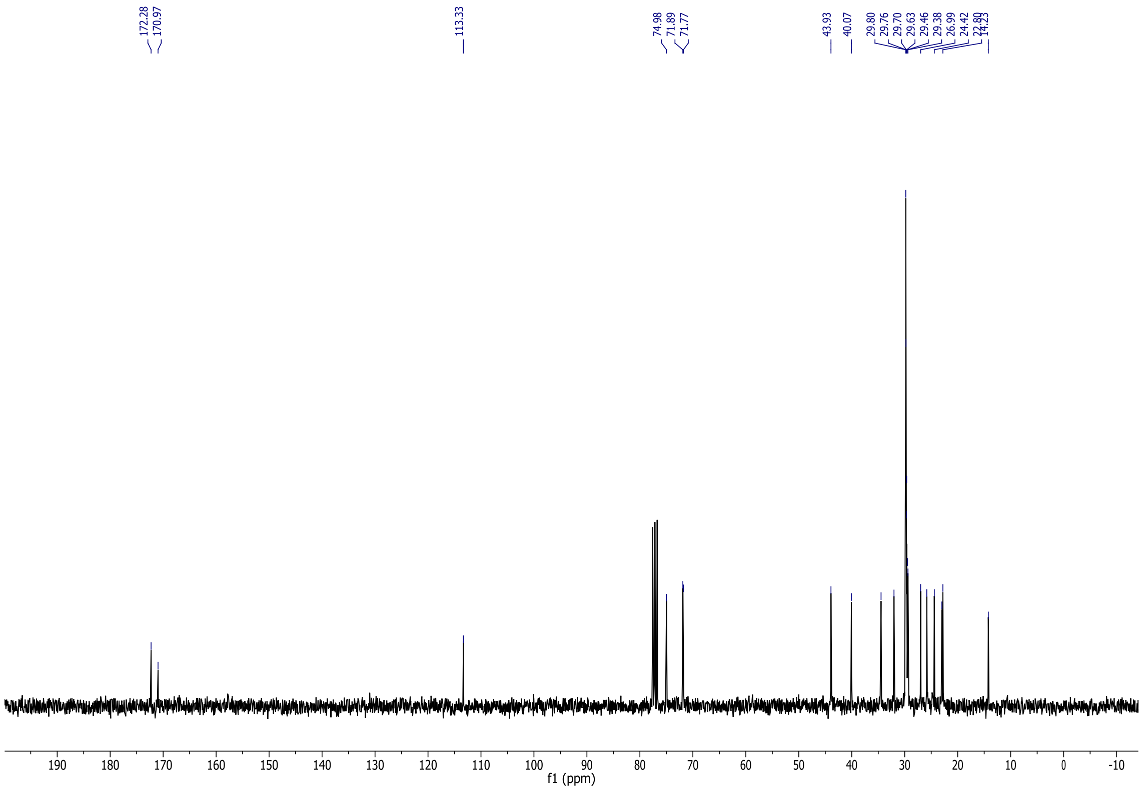

^{1}H NMR (300 MHz, $CDCl_3$, **3**)

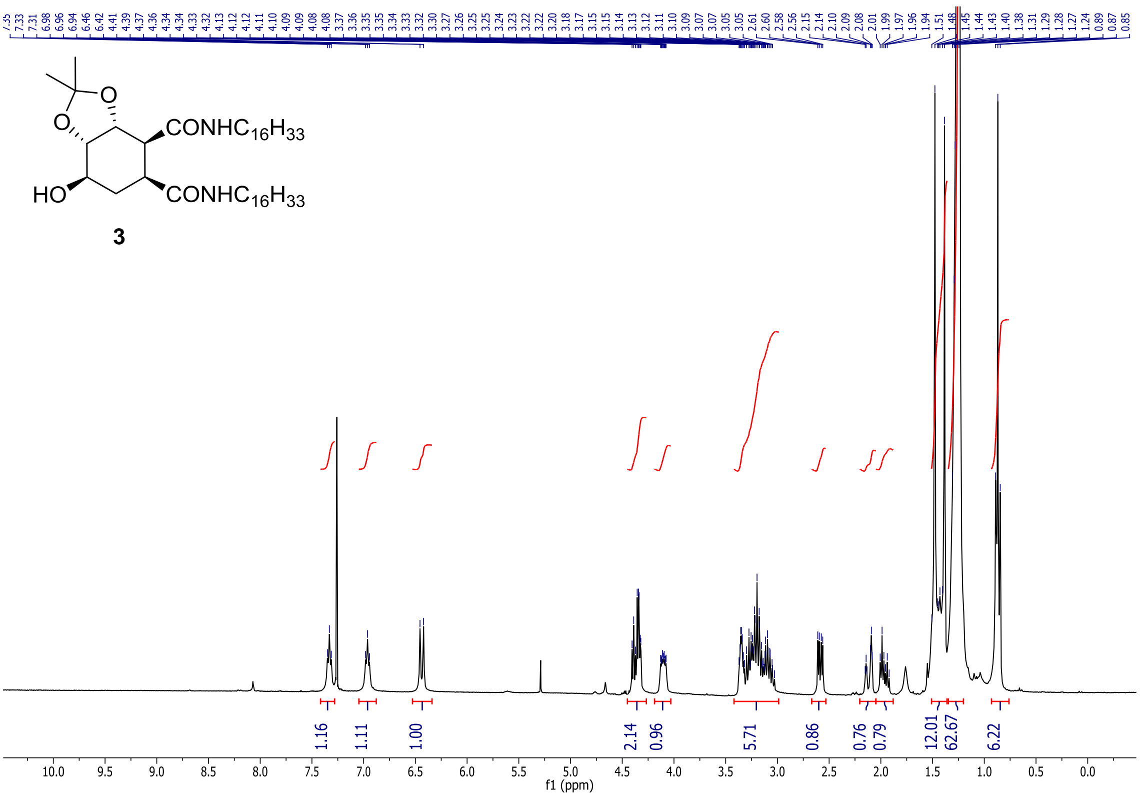

^{13}C NMR (75 MHz, $CDCl_3$, **3**)

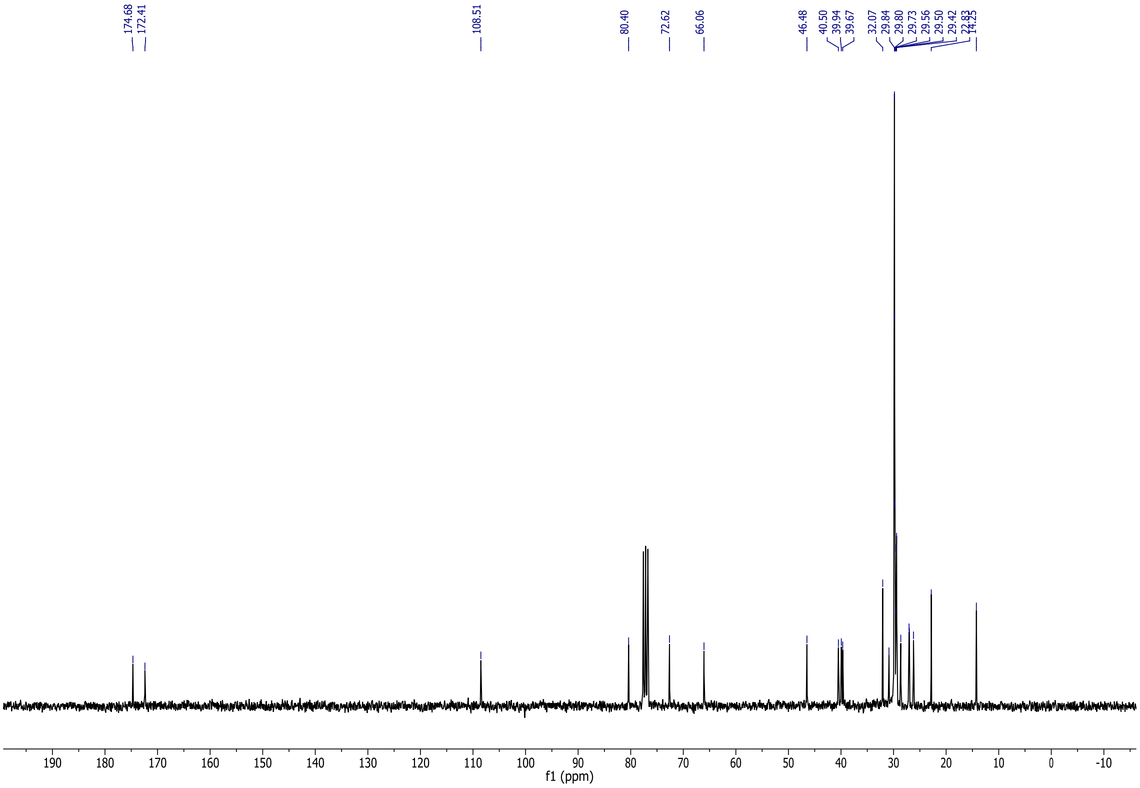

^{1}H NMR (300 MHz, $CDCl_3$, *cis*-**2**)

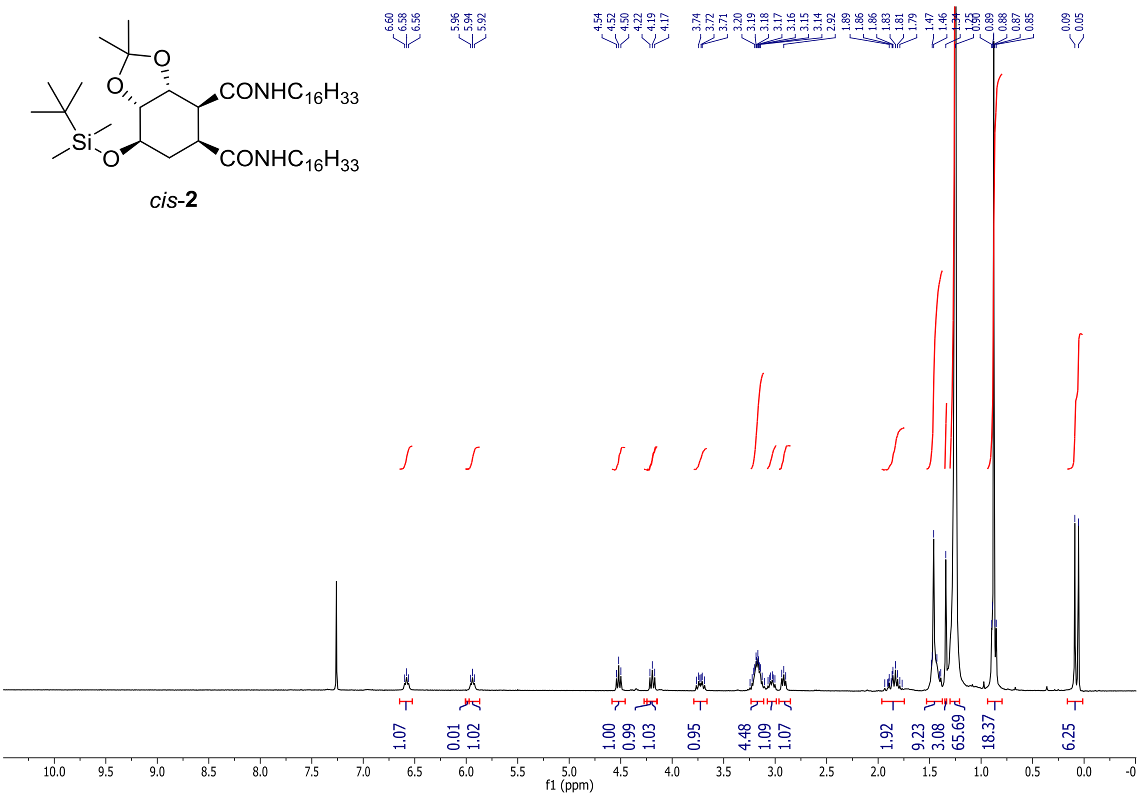

^{13}C NMR (75 MHz, $CDCl_3$, *cis*-**2**)

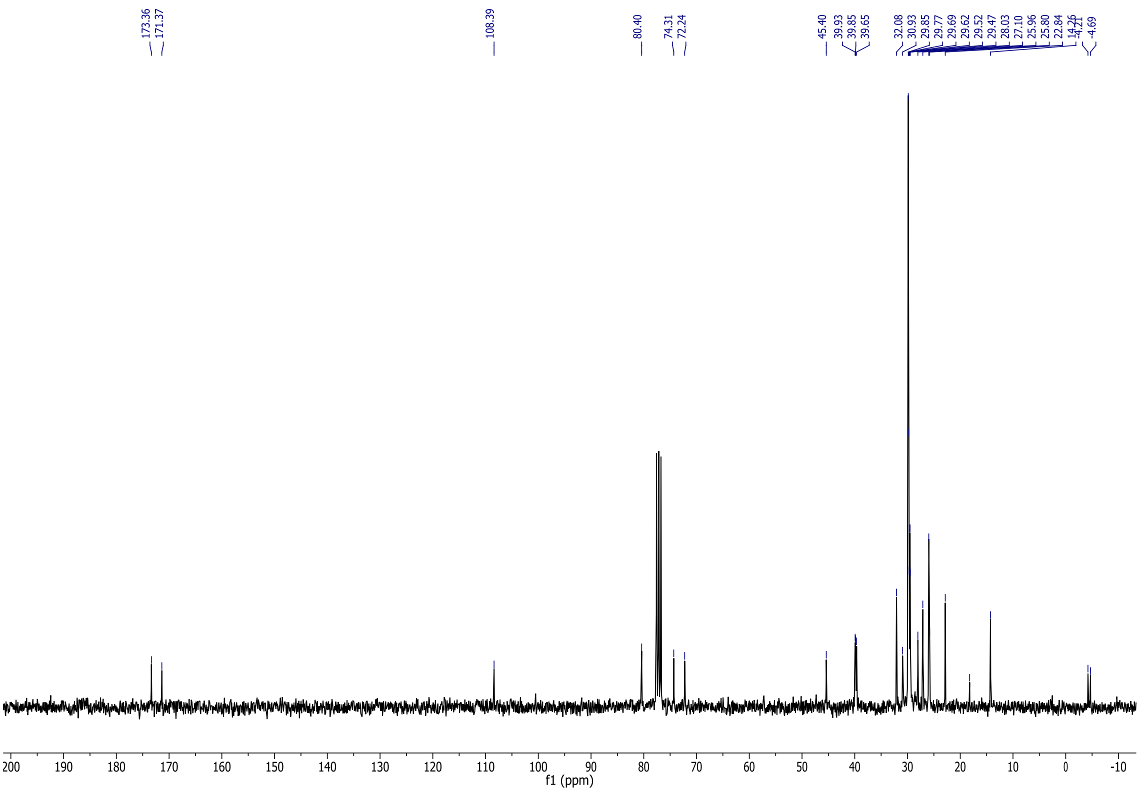

^{1}H NMR (300 MHz, THF-d_8, **5**)

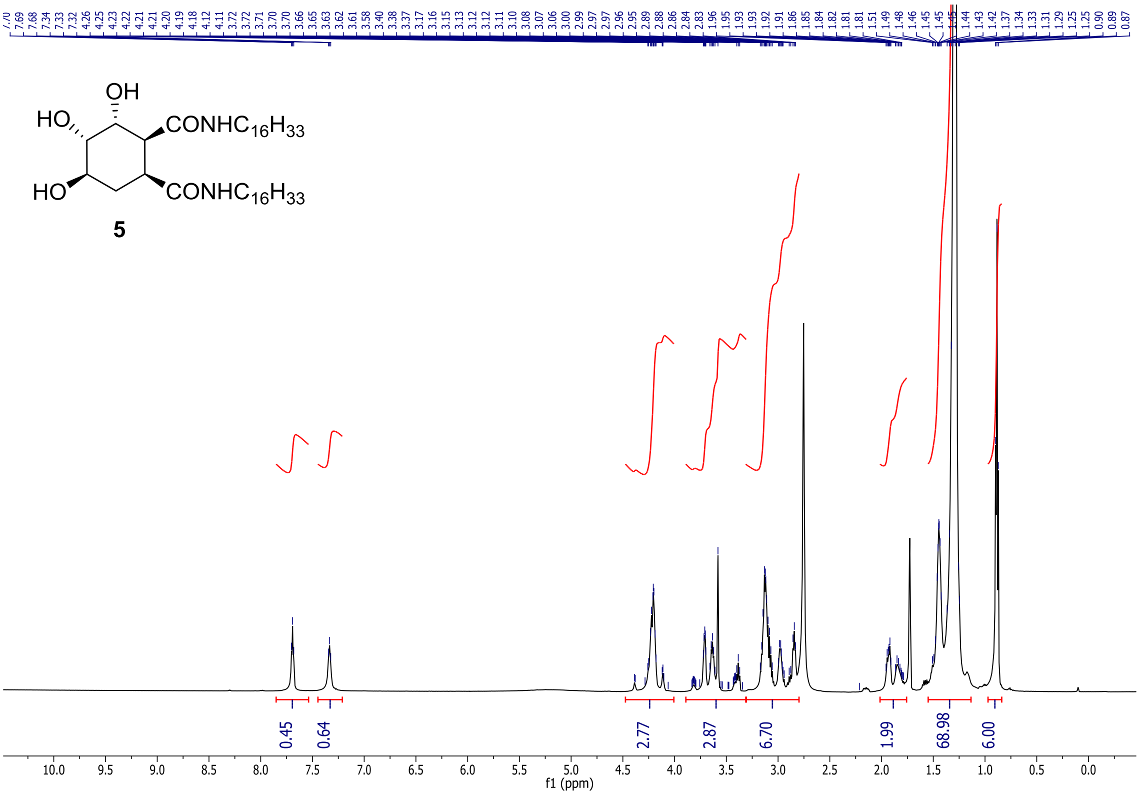

^{13}C NMR (75 MHz, THF-d_8, **5**)

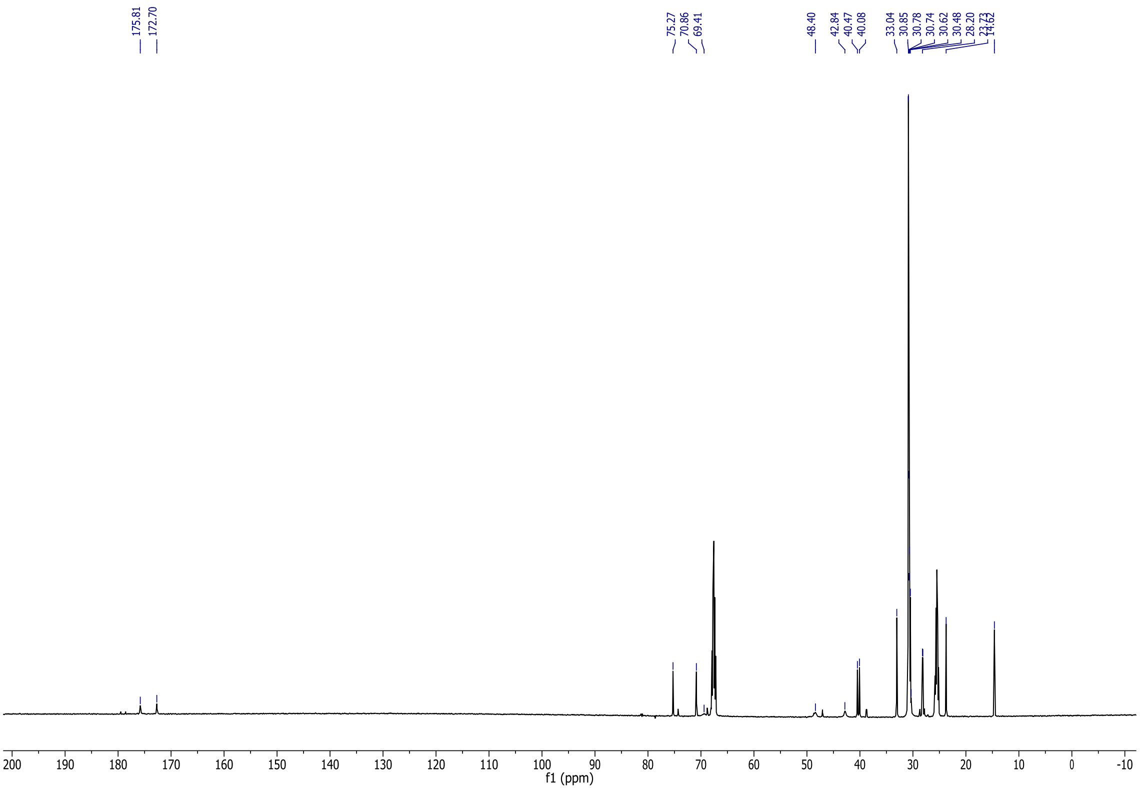

^{1}H NMR (300 MHz, $CDCl_3$, **10**)

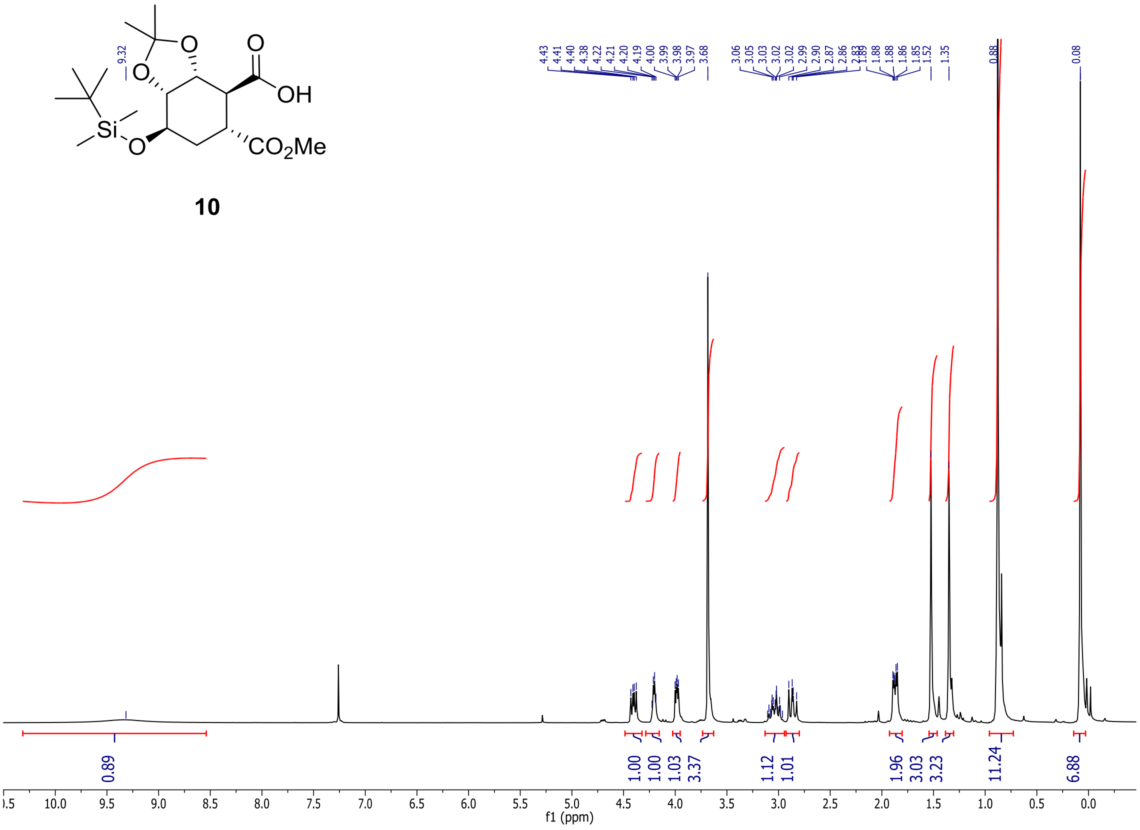

^{13}C NMR (75 MHz, $CDCl_3$, **10**)

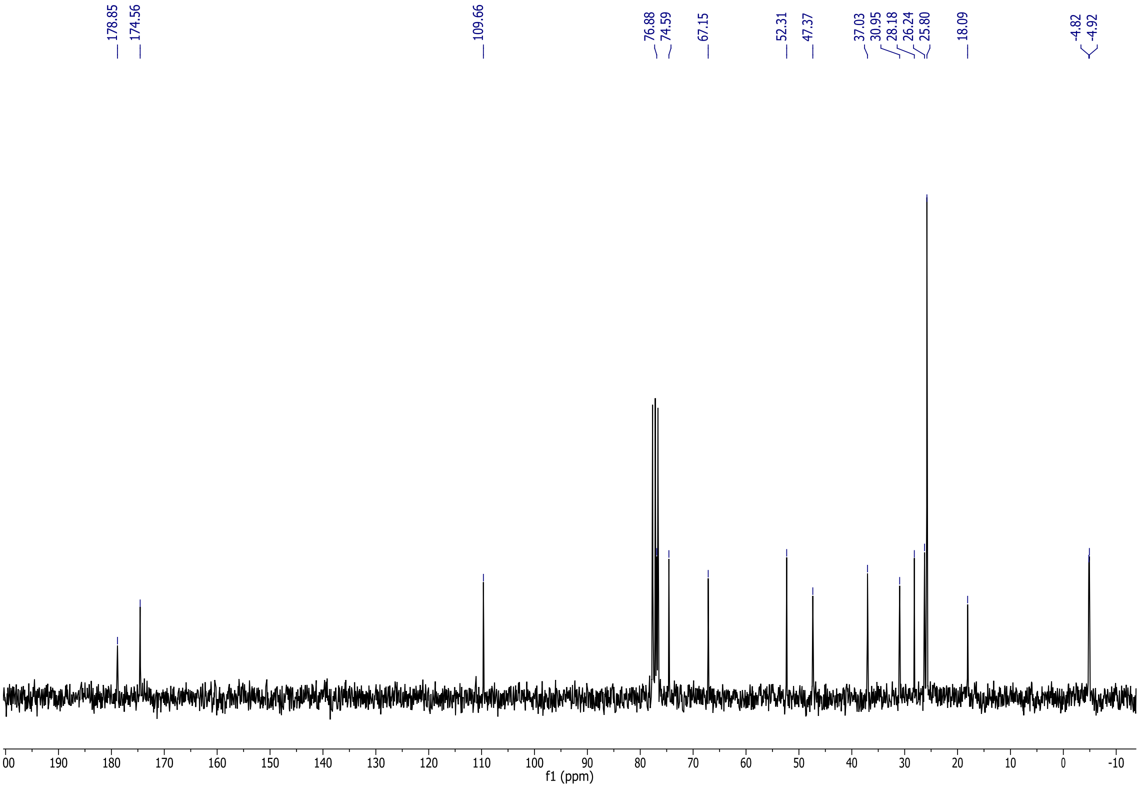

^{1}H NMR (300 MHz, $CDCl_3$, **11**)

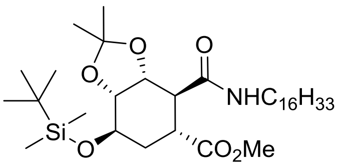

11

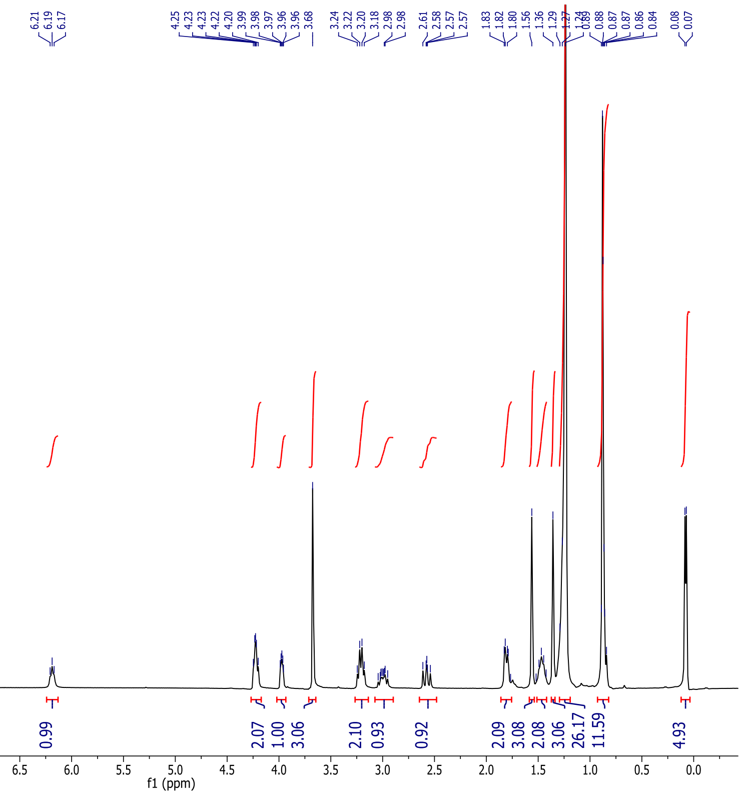

^{13}C NMR (75 MHz, $CDCl_3$, **11**)

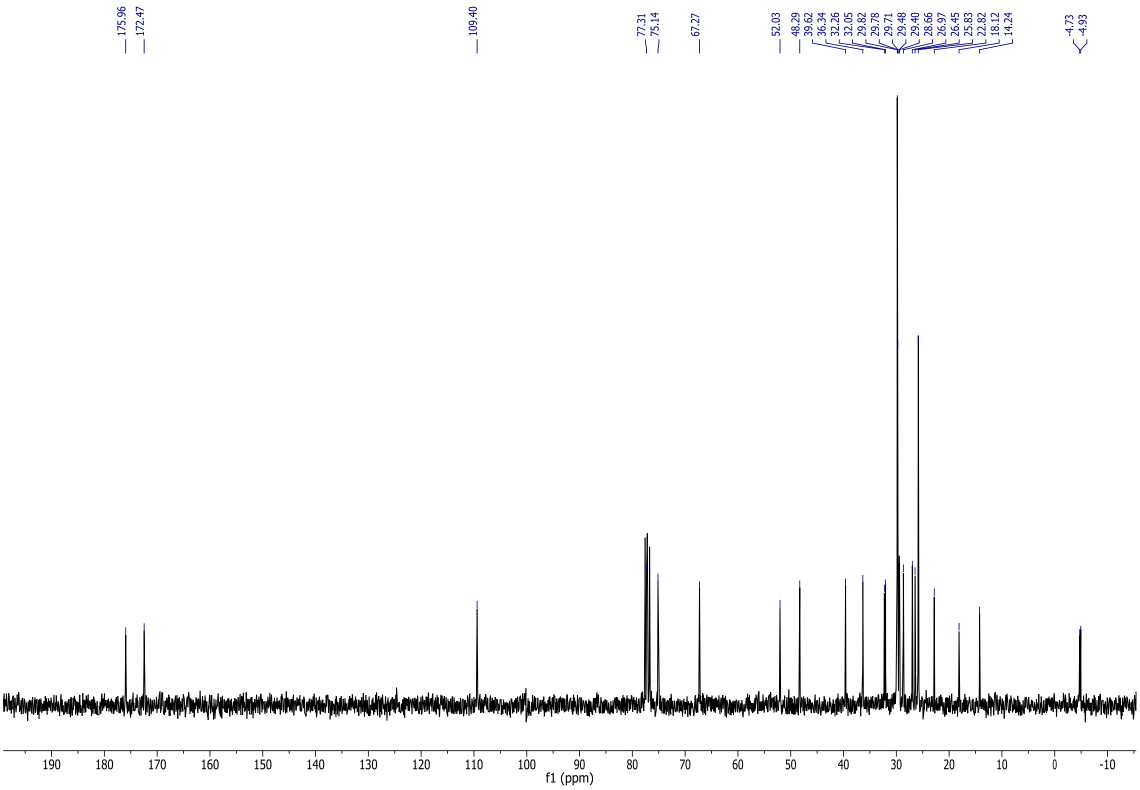

^{1}H NMR (300 MHz, $CDCl_3$, *trans*-**2**)

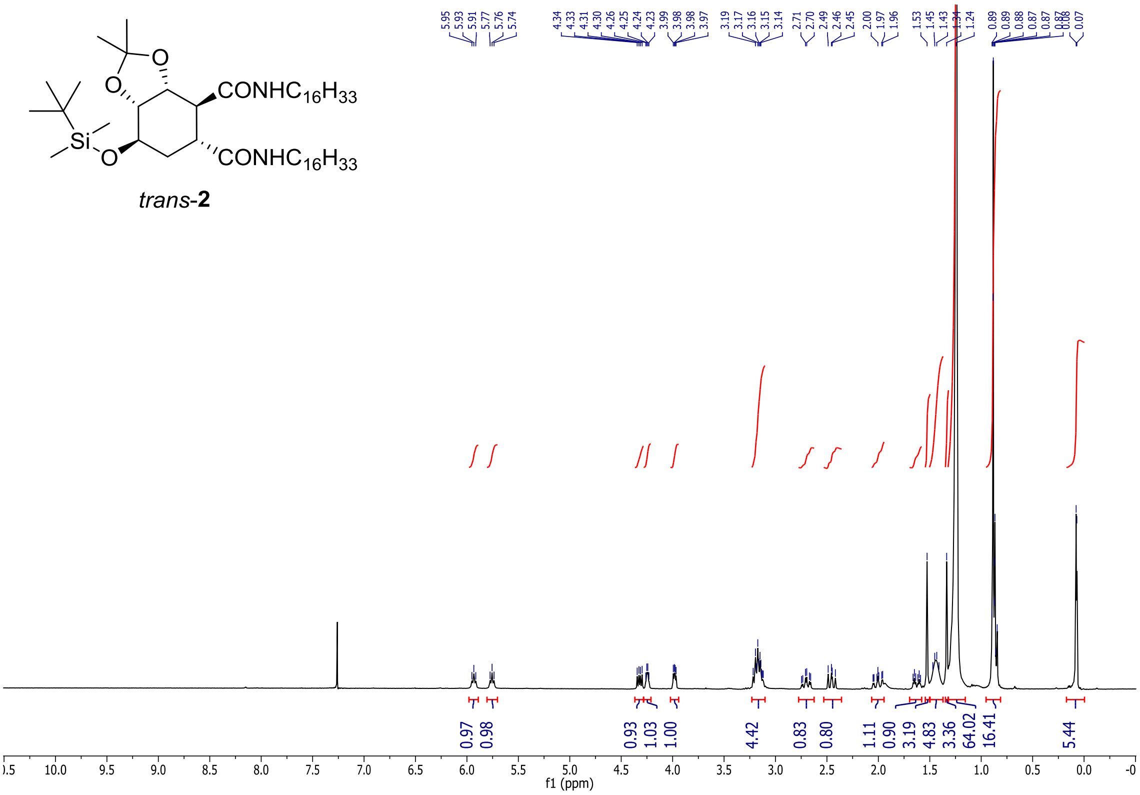

^{13}C NMR (75 MHz, $CDCl_3$, *trans*-**2**)

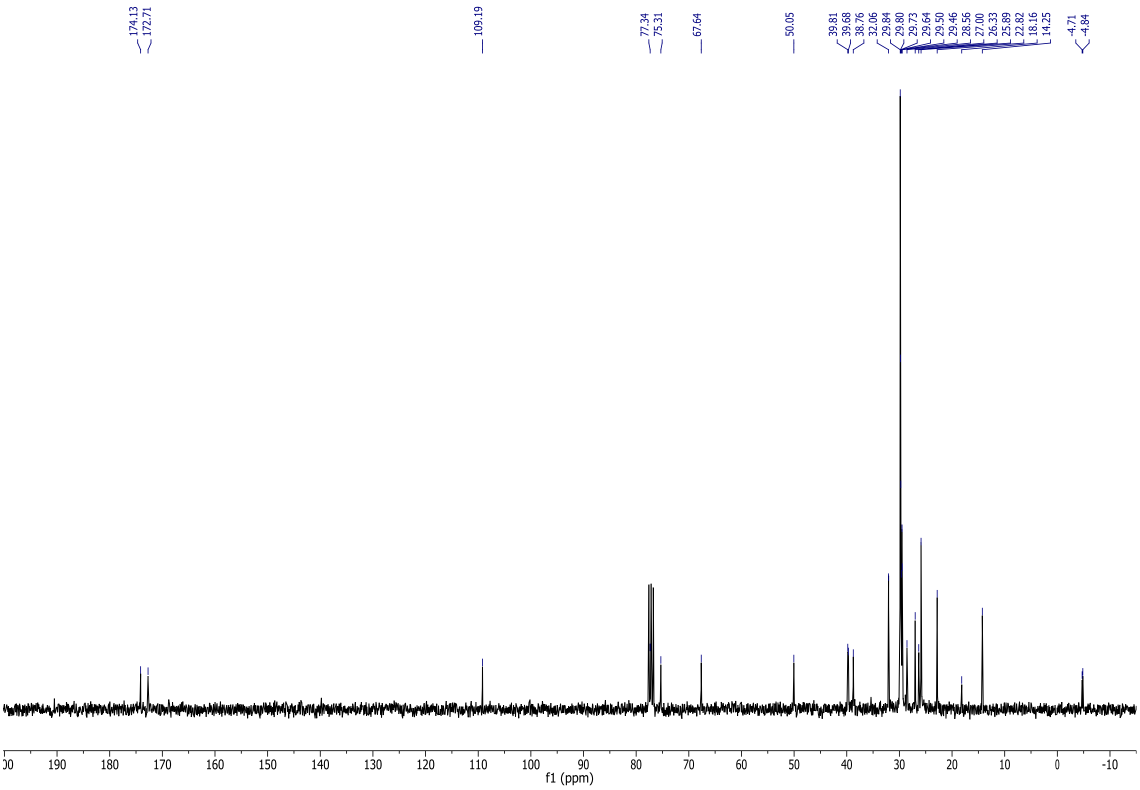

^{1}H NMR (300 MHz, $CDCl_3$, **4**)

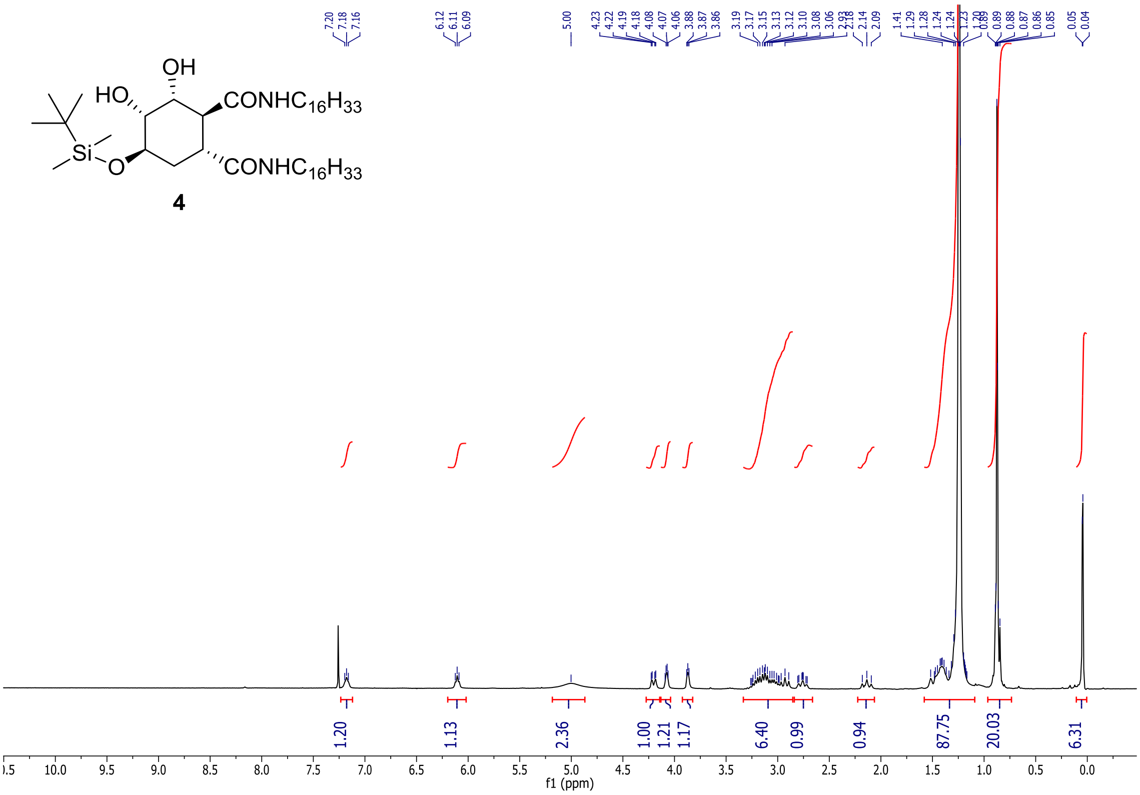

^{13}C NMR (75 MHz, $CDCl_3$, **4**)

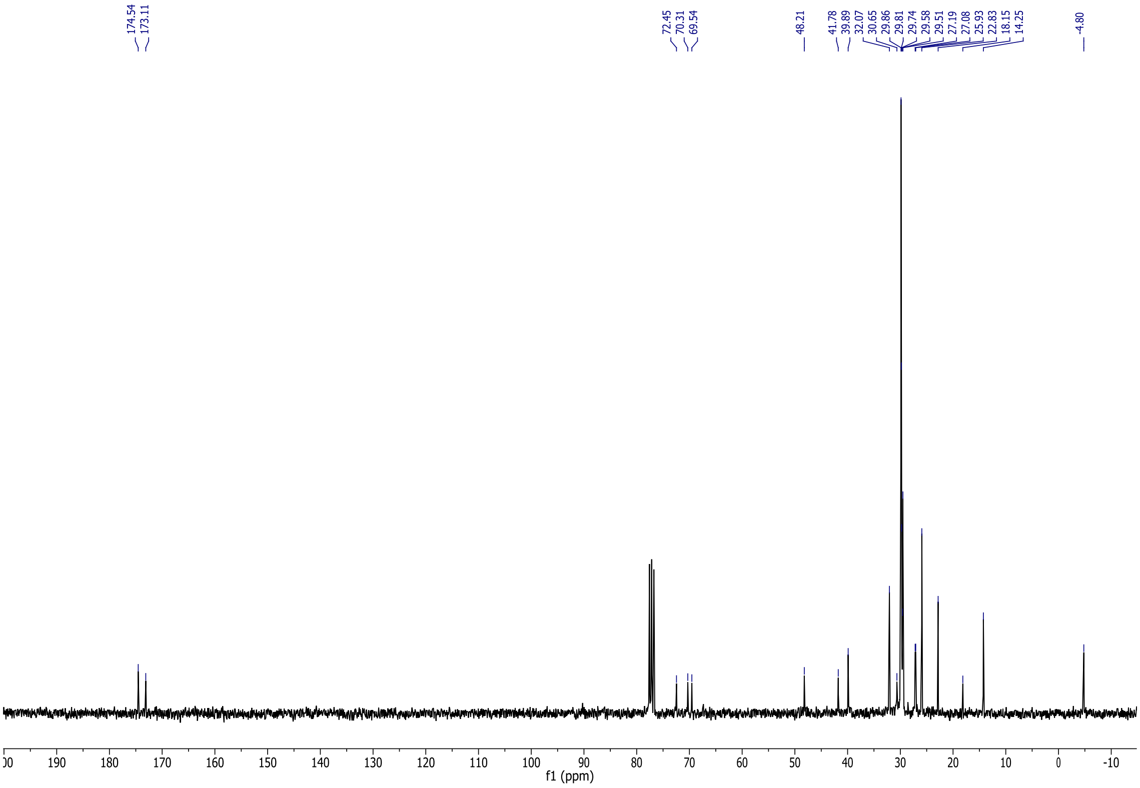

a) b)

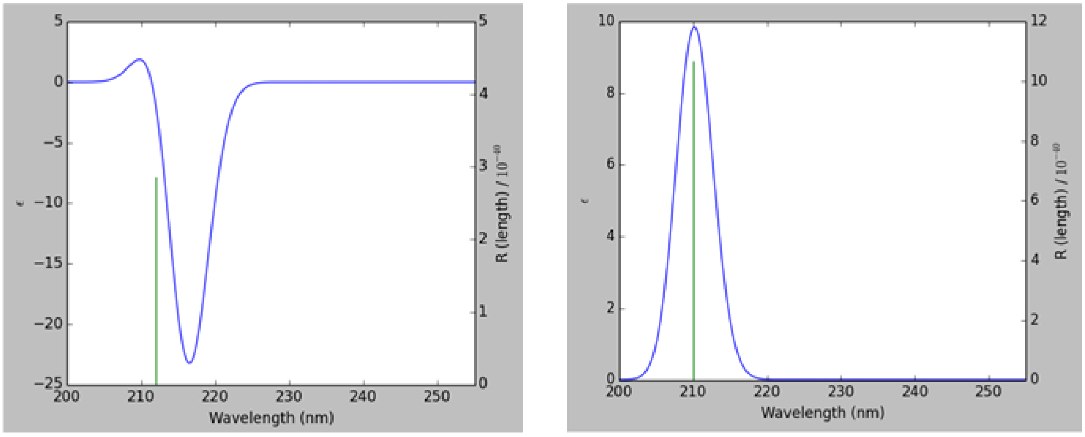

Fig S1 Predicted CD spectra of compounds a) *cis*-**2** and b) *trans*-**2**.

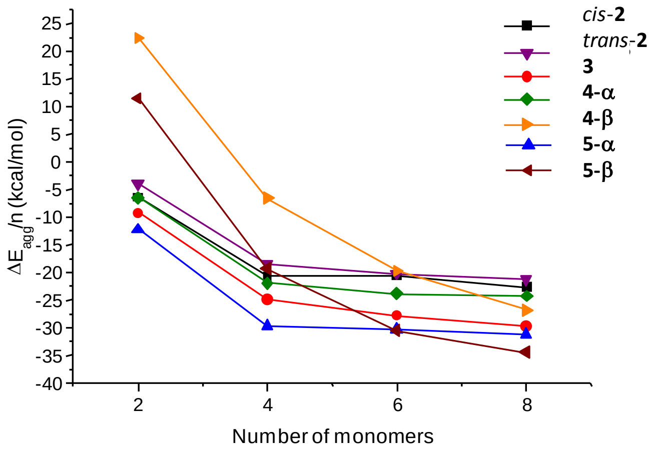

Fig S2 Calculated aggregation energies per monomer of compounds **2-5**

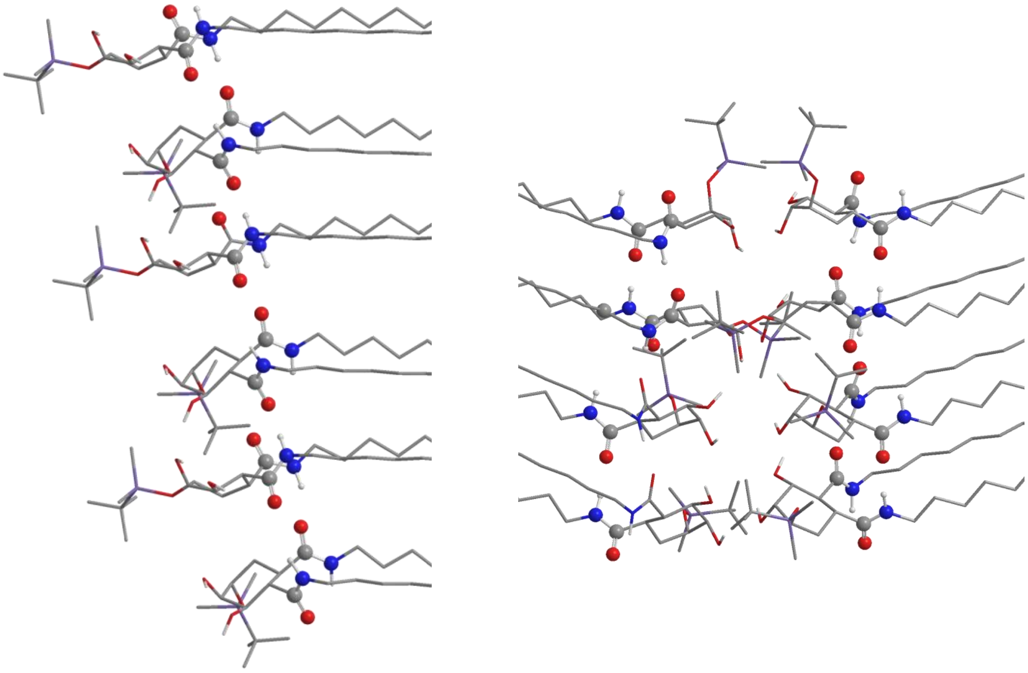

4-α **4-β**

Fig S3. Left, side view of central 6 molecules in octameric 1-D aggregate (amide hydrogen-bonds) **4-α.** Right, side views of octameric 2-D aggregates (amide and hydroxyl hydrogen-bonds) **4-β**. Non-polar hydrogen atoms have been omitted for clarity. Atoms in amide groups have been represented with red (oxygen), blue (nitrogen) and grey (carbon) spheres.

Total energies (in a.u) of aggregates of compounds **2-5** computed at the M06-2X/6-31G(d) level of calculation

n	*cis*-**2**	*trans*-**2**	**3**	**4-α**
1	-2699.11972	-2699.12057	-2172.63473	-2582.42466
2	-5398.31603	-5398.32032	-4345.35452	-5164.93920
4	-10796.72337	-10796.73309	-8690.80883	-10329.97647
6	-16195.08327	-16195.11592	-13036.24150	-15494.98322
8	-21593.47259	-21593.50166	-17381.67934	-20659.78011

n	**4-β**	**5-α**	**5-β**
1	-2582.42720	-2055.942949	-2055.93854
2	-5164.84722	-4111.975505	-4111.899911
4	-10329.87869	-8224.06170	-8223.99610
6	-15494.94451	-12336.09943	-12336.10119
8	-20660.01517	-16448.14413	-16448.18497

Cartesian coordinates (in Å) of aggregates of compounds **2-5**

cis-2 monomer

C	30.71830000	-15.63800000	-19.41910000
C	31.04810000	-15.67570000	-22.39990000
O	30.07300000	-14.65850000	-19.04440000
O	31.32070000	-16.81380000	-22.77420000
N	30.25460000	-16.89180000	-19.31040000
N	29.87640000	-15.09500000	-22.68770000
H	30.81980000	-17.64410000	-19.69180000
H	29.76210000	-14.11790000	-22.41310000
C	32.10590000	-15.43560000	-20.05690000
H	32.57950000	-14.65790000	-19.45610000
C	32.02880000	-14.88860000	-21.50950000
H	31.63690000	-13.87520000	-21.44560000
C	32.99390000	-16.69350000	-19.95640000
H	32.98000000	-17.07450000	-18.93730000
H	32.60500000	-17.48690000	-20.59040000
C	33.42990000	-14.77140000	-22.15940000
H	33.31990000	-14.69410000	-23.24280000
C	34.42940000	-16.39330000	-20.39030000
H	34.85240000	-15.65070000	-19.71830000
C	34.46490000	-15.86870000	-21.84200000
H	34.36220000	-16.69480000	-22.54370000
C	28.72970000	-15.76660000	-23.28780000
H	28.04830000	-15.00650000	-23.66900000
H	29.05270000	-16.35770000	-24.14580000
C	28.98240000	-17.25440000	-18.70600000
H	29.00540000	-16.99230000	-17.64700000
H	28.18210000	-16.66820000	-19.15930000
C	28.01180000	-16.64930000	-22.25050000
H	28.70750000	-17.40550000	-21.88350000
H	27.74450000	-16.03610000	-21.38930000
C	26.75110000	-17.34980000	-22.78100000
H	26.02190000	-16.60510000	-23.09830000
H	27.00050000	-17.93230000	-23.66730000
C	26.12470000	-18.26730000	-21.71890000
H	25.92250000	-17.68620000	-20.81880000
H	26.85050000	-19.02920000	-21.43390000
C	24.82930000	-18.95650000	-22.17340000
H	24.07670000	-18.20570000	-22.41170000
H	25.00840000	-19.51170000	-23.09360000
C	24.28550000	-19.90890000	-21.09820000
H	24.12990000	-19.35410000	-20.17260000
H	25.03680000	-20.66710000	-20.87720000
C	22.97580000	-20.60350000	-21.49770000
H	22.20330000	-19.85550000	-21.67430000
H	23.11070000	-21.13240000	-22.44090000
C	22.50270000	-21.59110000	-20.42200000
H	22.38670000	-21.06280000	-19.47550000
H	23.27420000	-22.34400000	-20.26020000
C	21.18430000	-22.29170000	-20.77750000
H	20.39820000	-21.54730000	-20.90400000
H	21.28330000	-22.79890000	-21.73720000
C	20.76700000	-23.30650000	-19.70460000
H	20.68480000	-22.79960000	-18.74310000
H	21.55110000	-24.05520000	-19.59120000
C	19.44120000	-24.00990000	-20.02410000
H	18.64770000	-23.26750000	-20.11070000
H	19.50980000	-24.50020000	-20.99530000
C	19.06500000	-25.04490000	-18.95530000
H	19.01170000	-24.55500000	-17.98300000
H	19.85520000	-25.79210000	-18.88060000
C	17.73170000	-25.74610000	-19.24660000

H	16.93650000	-25.00270000	-19.30330000
H	17.77490000	-26.22390000	-20.22570000
C	17.38120000	-26.79490000	-18.18250000
H	17.35330000	-26.31900000	-17.20220000
H	18.17150000	-27.54470000	-18.13830000
C	16.03880000	-27.48740000	-18.45310000
H	15.24050000	-26.74470000	-18.47740000
H	16.05550000	-27.95180000	-19.43940000
C	15.70680000	-28.55150000	-17.40180000
H	15.63900000	-28.11300000	-16.40650000
H	14.75180000	-29.02990000	-17.61970000
H	16.46780000	-29.33150000	-17.37380000
C	28.70650000	-18.75610000	-18.88130000
H	29.51740000	-19.33100000	-18.43090000
H	28.71280000	-19.00700000	-19.94270000
C	27.36960000	-19.18050000	-18.25530000
H	27.38290000	-18.94310000	-17.19060000
H	26.56150000	-18.59060000	-18.68820000
C	27.06040000	-20.67390000	-18.44110000
H	27.87430000	-21.26870000	-18.02420000
H	27.02120000	-20.90920000	-19.50530000
C	25.73750000	-21.07440000	-17.76980000
H	25.78950000	-20.83550000	-16.70660000
H	24.92420000	-20.47360000	-18.17720000
C	25.39610000	-22.56160000	-17.93700000
H	26.21580000	-23.16770000	-17.54950000
H	25.31010000	-22.79900000	-18.99780000
C	24.09500000	-22.93940000	-17.21190000
H	24.19040000	-22.69450000	-16.15310000
H	23.27380000	-22.33230000	-17.59300000
C	23.73260000	-24.42320000	-17.35540000
H	24.55990000	-25.03410000	-16.99260000
H	23.60750000	-24.66600000	-18.41080000
C	22.45460000	-24.78760000	-16.58470000
H	22.58670000	-24.54030000	-15.53060000
H	21.62460000	-24.17750000	-16.94140000
C	22.08240000	-26.27000000	-16.71290000
H	22.91790000	-26.88280000	-16.37270000
H	21.92670000	-26.51450000	-17.76360000
C	20.82580000	-26.63150000	-15.90780000
H	20.98700000	-26.38690000	-14.85720000
H	19.98740000	-26.01980000	-16.24140000
C	20.45080000	-28.11350000	-16.03110000
H	21.29480000	-28.72750000	-15.71500000
H	20.26830000	-28.35390000	-17.07830000
C	19.21510000	-28.47950000	-15.19740000
H	19.40180000	-28.24140000	-14.14950000
H	18.36900000	-27.86580000	-15.50730000
C	18.83890000	-29.96100000	-15.32250000
H	19.68970000	-30.57740000	-15.02960000
H	18.63330000	-30.19350000	-16.36720000
C	17.62150000	-30.33220000	-14.46530000
H	17.82990000	-30.11140000	-13.41780000
H	16.76970000	-29.71320000	-14.74790000
C	17.23490000	-31.80770000	-14.60060000
H	18.05360000	-32.45970000	-14.29560000
H	16.37250000	-32.04370000	-13.97670000
H	16.97500000	-32.05210000	-15.63010000
O	34.10050000	-13.61170000	-21.72550000
O	35.69710000	-15.18680000	-22.03080000
O	35.18010000	-17.59620000	-20.31940000
C	35.43730000	-13.82220000	-22.00710000
C	35.76780000	-13.23410000	-23.38540000
H	35.52800000	-12.17050000	-23.40030000
H	36.82960000	-13.36230000	-23.59550000
H	35.19810000	-13.73620000	-24.16640000
C	36.25590000	-13.16890000	-20.89520000
H	37.31550000	-13.38610000	-21.02840000

H	36.10230000	-12.08930000	-20.91270000
H	35.92890000	-13.54090000	-19.92770000
Si	36.03160000	-18.18270000	-18.97880000
C	35.00380000	-17.98820000	-17.40960000
H	34.71780000	-16.95150000	-17.24650000
H	34.09130000	-18.58070000	-17.45810000
H	35.55900000	-18.31500000	-16.53180000
C	36.32620000	-20.01760000	-19.27470000
H	36.93880000	-20.17860000	-20.15820000
H	36.83390000	-20.48350000	-18.43190000
H	35.38520000	-20.54400000	-19.42000000
C	37.71850000	-17.29480000	-18.77280000
C	37.50910000	-15.87760000	-18.22230000
H	38.45780000	-15.35840000	-18.08460000
H	36.91170000	-15.27830000	-18.90390000
H	37.00310000	-15.89490000	-17.25710000
C	38.41860000	-17.21620000	-20.13630000
H	38.62380000	-18.21080000	-20.53070000
H	37.79750000	-16.69120000	-20.86430000
H	39.36900000	-16.68660000	-20.07100000
C	38.59280000	-18.08940000	-17.78630000
H	38.80440000	-19.09290000	-18.15650000
H	39.55200000	-17.59800000	-17.62080000
H	38.10570000	-18.19320000	-16.81610000

cis-2 dimer

C	31.99360000	-20.70830000	-21.16810000
C	31.99600000	-20.05340000	-24.09560000
O	31.50890000	-19.72270000	-20.61080000
O	31.98420000	-21.11120000	-24.72130000
N	31.38630000	-21.90460000	-21.13010000
N	30.94710000	-19.22400000	-24.09520000
H	31.81690000	-22.68500000	-21.62040000
H	31.03600000	-18.35140000	-23.56890000
C	33.32440000	-20.57220000	-21.94600000
H	33.99580000	-20.04840000	-21.26520000
C	33.20030000	-19.67570000	-23.21490000
H	33.03030000	-18.65750000	-22.87090000
C	33.97200000	-21.93550000	-22.27160000
H	34.02340000	-22.55850000	-21.38000000
H	33.35970000	-22.47130000	-22.99300000
C	34.49830000	-19.64140000	-24.06100000
H	34.25250000	-19.29080000	-25.06530000
C	35.36730000	-21.76080000	-22.87200000
H	36.00210000	-21.24850000	-22.14960000
C	35.29140000	-20.95140000	-24.18120000
H	34.87960000	-21.56700000	-24.97660000
C	29.63780000	-19.57630000	-24.62180000
H	29.06000000	-18.66680000	-24.78000000
H	29.74440000	-20.05690000	-25.59620000
C	30.12650000	-22.16170000	-20.44290000
H	30.25330000	-21.95920000	-19.37820000
H	29.36600000	-21.46970000	-20.80760000
C	28.92490000	-20.50530000	-23.62860000
H	29.57540000	-21.35030000	-23.39850000
H	28.77400000	-19.97600000	-22.68670000
C	27.59160000	-21.05990000	-24.13230000
H	26.88480000	-20.24740000	-24.29600000
H	27.73760000	-21.54400000	-25.09810000
C	27.02770000	-22.06840000	-23.12620000
H	26.90000000	-21.58050000	-22.15910000
H	27.75860000	-22.86270000	-22.97240000
C	25.70150000	-22.69580000	-23.55910000
H	24.94740000	-21.91850000	-23.68080000
H	25.81780000	-23.16820000	-24.53470000
C	25.22970000	-23.73090000	-22.53220000

H	25.13820000	-23.25600000	-21.55470000
H	25.99130000	-24.50370000	-22.42640000
C	23.89720000	-24.38620000	-22.90460000
H	23.12500000	-23.62170000	-22.98890000
H	23.97860000	-24.85100000	-23.88750000
C	23.47950000	-25.43680000	-21.86920000
H	23.41620000	-24.97130000	-20.88530000
H	24.25390000	-26.20080000	-21.79800000
C	22.14110000	-26.10180000	-22.20540000
H	21.36110000	-25.34200000	-22.25730000
H	22.19600000	-26.55400000	-23.19600000
C	21.75560000	-27.16890000	-21.17400000
H	21.71580000	-26.71710000	-20.18280000
H	22.53360000	-27.93160000	-21.13370000
C	20.40940000	-27.83110000	-21.48790000
H	19.62900000	-27.07030000	-21.51160000
H	20.44230000	-28.26850000	-22.48620000
C	20.04220000	-28.91420000	-20.46610000
H	20.02580000	-28.47850000	-19.46710000
H	20.81720000	-29.68090000	-20.45570000
C	18.68510000	-29.56410000	-20.76240000
H	17.90900000	-28.79840000	-20.75610000
H	18.69470000	-29.98330000	-21.76900000
C	18.33010000	-30.66420000	-19.75380000
H	18.33580000	-30.24730000	-18.74660000
H	19.10090000	-31.43520000	-19.77310000
C	16.96320000	-31.30120000	-20.03650000
H	16.18640000	-30.53670000	-19.99370000
H	16.94740000	-31.69910000	-21.05160000
C	16.62340000	-32.42300000	-19.04990000
H	16.58430000	-32.04870000	-18.02710000
H	15.65350000	-32.86450000	-19.27990000
H	17.36560000	-33.22050000	-19.08800000
C	29.67060000	-23.61330000	-20.65940000
H	30.44310000	-24.28910000	-20.28910000
H	29.58150000	-23.80980000	-21.72870000
C	28.33820000	-23.94060000	-19.96680000
H	28.42670000	-23.73040000	-18.90010000
H	27.55680000	-23.28120000	-20.34590000
C	27.91580000	-25.40560000	-20.16510000
H	28.71040000	-26.06060000	-19.80490000
H	27.80790000	-25.61190000	-21.23040000
C	26.60650000	-25.75380000	-19.43990000
H	26.71330000	-25.53050000	-18.37770000
H	25.80380000	-25.11510000	-19.80930000
C	26.20600000	-27.22760000	-19.60900000
H	27.01860000	-27.86650000	-19.26020000
H	26.07420000	-27.44870000	-20.66800000
C	24.91920000	-27.58430000	-18.84940000
H	25.05290000	-27.36330000	-17.78980000
H	24.10420000	-26.94880000	-19.19660000
C	24.51940000	-29.05820000	-19.01300000
H	25.34060000	-29.69670000	-18.68440000
H	24.36420000	-29.27550000	-20.06940000
C	23.24940000	-29.41860000	-18.22830000
H	23.40780000	-29.21150000	-17.16940000
H	22.42980000	-28.77570000	-18.54970000
C	22.83960000	-30.88790000	-18.40350000
H	23.66400000	-31.53480000	-18.10050000
H	22.66200000	-31.08890000	-19.45960000
C	21.58340000	-31.25220000	-17.59960000
H	21.76380000	-31.06340000	-16.54070000
H	20.76210000	-30.59900000	-17.89460000
C	21.16120000	-32.71530000	-17.79200000
H	21.98590000	-33.37260000	-17.51290000
H	20.96530000	-32.89770000	-18.84820000
C	19.91550000	-33.08360000	-16.97440000
H	20.11210000	-32.90950000	-15.91590000

H	19.09340000	-32.42230000	-17.24890000
C	19.48360000	-34.54190000	-17.18010000
H	20.30830000	-35.20660000	-16.91870000
H	19.27470000	-34.71030000	-18.23620000
C	18.24560000	-34.91250000	-16.35220000
H	18.45170000	-34.75150000	-15.29330000
H	17.41880000	-34.25050000	-16.61110000
C	17.80710000	-36.36480000	-16.56410000
H	18.60310000	-37.06210000	-16.30070000
H	16.93930000	-36.60370000	-15.94890000
H	17.53340000	-36.54420000	-17.60330000
O	35.47940000	-18.76670000	-23.55040000
O	36.59410000	-20.49230000	-24.50850000
O	35.88040000	-23.05100000	-23.16570000
C	36.65970000	-19.14680000	-24.17010000
C	36.85830000	-18.32430000	-25.45180000
H	36.86450000	-17.26100000	-25.21080000
H	37.80890000	-18.58840000	-25.91510000
H	36.06170000	-18.51960000	-26.16830000
C	37.80430000	-18.93980000	-23.18380000
H	38.74330000	-19.28010000	-23.61960000
H	37.61060000	-19.49810000	-22.27160000
H	37.88600000	-17.88130000	-22.93840000
Si	37.30040000	-23.71740000	-22.53540000
C	37.89130000	-25.06680000	-23.71220000
H	37.15410000	-25.86210000	-23.80650000
H	38.08460000	-24.66910000	-24.70540000
H	38.81700000	-25.52120000	-23.36370000
C	38.63000000	-22.39350000	-22.36140000
H	38.36500000	-21.65400000	-21.60910000
H	39.58540000	-22.82660000	-22.06980000
H	38.77750000	-21.86620000	-23.30320000
C	36.91620000	-24.52530000	-20.84310000
C	35.67220000	-25.41880000	-20.99190000
H	35.41060000	-25.90230000	-20.05060000
H	34.80600000	-24.84070000	-21.31500000
H	35.82930000	-26.20340000	-21.73270000
C	36.65640000	-23.43920000	-19.78750000
H	37.52940000	-22.80140000	-19.64720000
H	35.82600000	-22.79770000	-20.07250000
H	36.41280000	-23.87510000	-18.81850000
C	38.11840000	-25.37790000	-20.40040000
H	39.02740000	-24.77890000	-20.33270000
H	37.94760000	-25.82760000	-19.42190000
H	38.31280000	-26.19140000	-21.09980000
C	30.71830000	-15.63800000	-19.41910000
C	31.04810000	-15.67570000	-22.39990000
O	30.07300000	-14.65850000	-19.04440000
O	31.32070000	-16.81380000	-22.77420000
N	30.25460000	-16.89180000	-19.31040000
N	29.87640000	-15.09500000	-22.68770000
H	30.81980000	-17.64410000	-19.69180000
H	29.76210000	-14.11790000	-22.41310000
C	32.10590000	-15.43560000	-20.05690000
H	32.57950000	-14.65790000	-19.45610000
C	32.02880000	-14.88860000	-21.50950000
H	31.63690000	-13.87520000	-21.44560000
C	32.99390000	-16.69350000	-19.95640000
H	32.98000000	-17.07450000	-18.93730000
H	32.60500000	-17.48690000	-20.59040000
C	33.42990000	-14.77140000	-22.15940000
H	33.31990000	-14.69410000	-23.24280000
C	34.42940000	-16.39330000	-20.39030000
H	34.85240000	-15.65070000	-19.71830000
C	34.46490000	-15.86870000	-21.84200000
H	34.36220000	-16.69480000	-22.54370000
C	28.72970000	-15.76660000	-23.28780000
H	28.04830000	-15.00650000	-23.66900000

H	29.05270000	-16.35770000	-24.14580000
C	28.98240000	-17.25440000	-18.70600000
H	29.00540000	-16.99230000	-17.64700000
H	28.18210000	-16.66820000	-19.15930000
C	28.01180000	-16.64930000	-22.25050000
H	28.70750000	-17.40550000	-21.88350000
H	27.74450000	-16.03610000	-21.38930000
C	26.75110000	-17.34980000	-22.78100000
H	26.02190000	-16.60510000	-23.09830000
H	27.00050000	-17.93230000	-23.66730000
C	26.12470000	-18.26730000	-21.71890000
H	25.92250000	-17.68620000	-20.81880000
H	26.85050000	-19.02920000	-21.43390000
C	24.82930000	-18.95650000	-22.17340000
H	24.07670000	-18.20570000	-22.41170000
H	25.00840000	-19.51170000	-23.09360000
C	24.28550000	-19.90890000	-21.09820000
H	24.12990000	-19.35410000	-20.17260000
H	25.03680000	-20.66710000	-20.87720000
C	22.97580000	-20.60350000	-21.49770000
H	22.20330000	-19.85550000	-21.67430000
H	23.11070000	-21.13240000	-22.44090000
C	22.50270000	-21.59110000	-20.42200000
H	22.38670000	-21.06280000	-19.47550000
H	23.27420000	-22.34400000	-20.26020000
C	21.18430000	-22.29170000	-20.77750000
H	20.39820000	-21.54730000	-20.90400000
H	21.28330000	-22.79890000	-21.73720000
C	20.76700000	-23.30650000	-19.70460000
H	20.68480000	-22.79960000	-18.74310000
H	21.55110000	-24.05520000	-19.59120000
C	19.44120000	-24.00990000	-20.02410000
H	18.64770000	-23.26750000	-20.11070000
H	19.50980000	-24.50020000	-20.99530000
C	19.06500000	-25.04490000	-18.95530000
H	19.01170000	-24.55500000	-17.98300000
H	19.85520000	-25.79210000	-18.88060000
C	17.73170000	-25.74610000	-19.24660000
H	16.93650000	-25.00270000	-19.30330000
H	17.77490000	-26.22390000	-20.22570000
C	17.38120000	-26.79490000	-18.18250000
H	17.35330000	-26.31900000	-17.20220000
H	18.17150000	-27.54470000	-18.13830000
C	16.03880000	-27.48740000	-18.45310000
H	15.24050000	-26.74470000	-18.47740000
H	16.05550000	-27.95180000	-19.43940000
C	15.70680000	-28.55150000	-17.40180000
H	15.63900000	-28.11300000	-16.40650000
H	14.75180000	-29.02990000	-17.61970000
H	16.46780000	-29.33150000	-17.37380000
C	28.70650000	-18.75610000	-18.88130000
H	29.51740000	-19.33100000	-18.43090000
H	28.71280000	-19.00700000	-19.94270000
C	27.36960000	-19.18050000	-18.25530000
H	27.38290000	-18.94310000	-17.19060000
H	26.56150000	-18.59060000	-18.68820000
C	27.06040000	-20.67390000	-18.44110000
H	27.87430000	-21.26870000	-18.02420000
H	27.02120000	-20.90920000	-19.50530000
C	25.73750000	-21.07440000	-17.76980000
H	25.78950000	-20.83550000	-16.70660000
H	24.92420000	-20.47360000	-18.17720000
C	25.39610000	-22.56160000	-17.93700000
H	26.21580000	-23.16770000	-17.54950000
H	25.31010000	-22.79900000	-18.99780000
C	24.09500000	-22.93940000	-17.21190000
H	24.19040000	-22.69450000	-16.15310000
H	23.27380000	-22.33230000	-17.59300000

C	23.73260000	-24.42320000	-17.35540000
H	24.55990000	-25.03410000	-16.99260000
H	23.60750000	-24.66600000	-18.41080000
C	22.45460000	-24.78760000	-16.58470000
H	22.58670000	-24.54030000	-15.53060000
H	21.62460000	-24.17750000	-16.94140000
C	22.08240000	-26.27000000	-16.71290000
H	22.91790000	-26.88280000	-16.37270000
H	21.92670000	-26.51450000	-17.76360000
C	20.82580000	-26.63150000	-15.90780000
H	20.98700000	-26.38690000	-14.85720000
H	19.98740000	-26.01980000	-16.24140000
C	20.45080000	-28.11350000	-16.03110000
H	21.29480000	-28.72750000	-15.71500000
H	20.26830000	-28.35390000	-17.07830000
C	19.21510000	-28.47950000	-15.19740000
H	19.40180000	-28.24140000	-14.14950000
H	18.36900000	-27.86580000	-15.50730000
C	18.83890000	-29.96100000	-15.32250000
H	19.68970000	-30.57740000	-15.02960000
H	18.63330000	-30.19350000	-16.36720000
C	17.62150000	-30.33220000	-14.46530000
H	17.82990000	-30.11140000	-13.41780000
H	16.76970000	-29.71320000	-14.74790000
C	17.23490000	-31.80770000	-14.60060000
H	18.05360000	-32.45970000	-14.29560000
H	16.37250000	-32.04370000	-13.97670000
H	16.97500000	-32.05210000	-15.63010000
O	34.10050000	-13.61170000	-21.72550000
O	35.69710000	-15.18680000	-22.03080000
O	35.18010000	-17.59620000	-20.31940000
C	35.43730000	-13.82220000	-22.00710000
C	35.76780000	-13.23410000	-23.38540000
H	35.52800000	-12.17050000	-23.40030000
H	36.82960000	-13.36230000	-23.59550000
H	35.19810000	-13.73620000	-24.16640000
C	36.25590000	-13.16890000	-20.89520000
H	37.31550000	-13.38610000	-21.02840000
H	36.10230000	-12.08930000	-20.91270000
H	35.92890000	-13.54090000	-19.92770000
Si	36.03160000	-18.18270000	-18.97880000
C	35.00380000	-17.98820000	-17.40960000
H	34.71780000	-16.95150000	-17.24650000
H	34.09130000	-18.58070000	-17.45810000
H	35.55900000	-18.31500000	-16.53180000
C	36.32620000	-20.01760000	-19.27470000
H	36.93880000	-20.17860000	-20.15820000
H	36.83390000	-20.48350000	-18.43190000
H	35.38520000	-20.54400000	-19.42000000
C	37.71850000	-17.29480000	-18.77280000
C	37.50910000	-15.87760000	-18.22230000
H	38.45780000	-15.35840000	-18.08460000
H	36.91170000	-15.27830000	-18.90390000
H	37.00310000	-15.89490000	-17.25710000
C	38.41860000	-17.21620000	-20.13630000
H	38.62380000	-18.21080000	-20.53070000
H	37.79750000	-16.69120000	-20.86430000
H	39.36900000	-16.68660000	-20.07100000
C	38.59280000	-18.08940000	-17.78630000
H	38.80440000	-19.09290000	-18.15650000
H	39.55200000	-17.59800000	-17.62080000
H	38.10570000	-18.19320000	-16.81610000

cis-2 tetramer

C	28.54640000	-10.69590000	-19.23180000

C	29.27040000	-11.23100000	-22.07250000
O	27.64360000	-9.86670000	-19.31970000
O	29.81730000	-12.31460000	-22.26590000
N	28.34960000	-11.92210000	-18.72800000
N	28.01680000	-10.97530000	-22.46760000
H	29.11250000	-12.59110000	-18.75320000
H	27.63520000	-10.07290000	-22.21840000
C	29.94550000	-10.32540000	-19.75220000
H	30.15150000	-9.35430000	-19.29920000
C	29.98560000	-10.10960000	-21.29550000
H	29.42530000	-9.19720000	-21.50530000
C	31.04190000	-11.28780000	-19.25790000
H	30.98230000	-11.40000000	-18.17500000
H	30.92080000	-12.28090000	-19.69000000
C	31.42210000	-9.86220000	-21.83230000
H	31.44310000	-10.04910000	-22.90780000
C	32.40960000	-10.73240000	-19.63870000
H	32.48660000	-9.72520000	-19.23690000
C	32.55980000	-10.65470000	-21.16820000
H	32.64350000	-11.64820000	-21.60220000
C	27.08380000	-11.95060000	-23.01350000
H	26.31110000	-11.40830000	-23.55930000
H	27.58400000	-12.59830000	-23.73560000
C	27.09540000	-12.36480000	-18.14050000
H	27.01970000	-11.95350000	-17.13290000
H	26.25090000	-11.96800000	-18.70690000
C	26.45170000	-12.77350000	-21.87930000
H	27.21710000	-13.40220000	-21.42090000
H	26.10450000	-12.09700000	-21.09720000
C	25.27200000	-13.64260000	-22.33370000
H	24.48980000	-13.00120000	-22.74210000
H	25.58600000	-14.30040000	-23.14390000
C	24.70060000	-14.47300000	-21.17470000
H	24.47240000	-13.81200000	-20.33780000
H	25.46370000	-15.16530000	-20.81780000
C	23.43330000	-15.25350000	-21.54990000
H	22.65320000	-14.55440000	-21.85440000
H	23.63410000	-15.88580000	-22.41490000
C	22.92450000	-16.11490000	-20.38400000
H	22.75550000	-15.47980000	-19.51370000
H	23.69890000	-16.82680000	-20.09750000
C	21.63140000	-16.87560000	-20.71110000
H	20.84070000	-16.16290000	-20.94940000
H	21.78010000	-17.48030000	-21.60610000
C	21.18060000	-17.77620000	-19.55080000
H	21.04630000	-17.16960000	-18.65460000
H	21.97070000	-18.49150000	-19.32110000
C	19.88000000	-18.53690000	-19.84770000
H	19.07660000	-17.82330000	-20.03540000
H	19.99710000	-19.11600000	-20.76410000
C	19.47840000	-19.47370000	-18.69820000
H	19.37080000	-18.89380000	-17.78090000
H	20.28090000	-20.18920000	-18.51700000
C	18.17400000	-20.23580000	-18.97360000
H	17.36130000	-19.52300000	-19.11950000
H	18.26640000	-20.79180000	-19.90710000
C	17.81130000	-21.20330000	-17.83700000
H	17.72750000	-20.64760000	-16.90250000
H	18.62140000	-21.91960000	-17.69770000
C	16.50260000	-21.96320000	-18.09660000
H	15.68520000	-21.24960000	-18.20880000
H	16.57390000	-22.49970000	-19.04320000
C	16.16700000	-22.95440000	-16.97270000
H	16.09900000	-22.41800000	-16.02560000
H	16.98220000	-23.67030000	-16.86350000
C	14.85650000	-23.71260000	-17.22520000
H	14.03360000	-23.00250000	-17.31680000
H	14.91350000	-24.23950000	-18.17810000

C	14.53570000	-24.71700000	-16.11280000
H	14.42330000	-24.21640000	-15.15090000
H	13.60600000	-25.24650000	-16.32230000
H	15.32400000	-25.46300000	-16.01250000
C	27.01870000	-13.89530000	-18.10130000
H	27.86690000	-14.29470000	-17.54350000
H	27.10070000	-14.29170000	-19.11390000
C	25.71540000	-14.37710000	-17.45620000
H	25.67790000	-14.02600000	-16.42390000
H	24.86560000	-13.91970000	-17.96440000
C	25.55910000	-15.90150000	-17.48480000
H	26.42020000	-16.37020000	-17.00670000
H	25.54910000	-16.24890000	-18.51850000
C	24.27470000	-16.34140000	-16.77390000
H	24.31630000	-16.02400000	-15.73090000
H	23.41910000	-15.82810000	-17.21440000
C	24.04060000	-17.85380000	-16.83600000
H	24.90170000	-18.37650000	-16.41790000
H	23.95920000	-18.16870000	-17.87680000
C	22.77540000	-18.25480000	-16.06890000
H	22.87880000	-17.95660000	-15.02460000
H	21.91980000	-17.70330000	-16.46040000
C	22.48420000	-19.75660000	-16.14140000
H	23.34490000	-20.31350000	-15.76930000
H	22.34840000	-20.05130000	-17.18230000
C	21.24040000	-20.13240000	-15.32700000
H	21.39280000	-19.84870000	-14.28470000
H	20.38410000	-19.55600000	-15.67890000
C	20.91250000	-21.62660000	-15.40430000
H	21.77420000	-22.20510000	-15.06950000
H	20.73520000	-21.90590000	-16.44310000
C	19.69020000	-21.99170000	-14.55280000
H	19.88030000	-21.72150000	-13.51310000
H	18.83150000	-21.39990000	-14.87150000
C	19.34070000	-23.48140000	-14.63590000
H	20.20400000	-24.07330000	-14.33010000
H	19.13410000	-23.74720000	-15.67280000
C	18.13590000	-23.84540000	-13.75870000
H	18.35310000	-23.58980000	-12.72070000
H	17.27560000	-23.24200000	-14.05030000
C	17.77110000	-25.33140000	-13.85280000
H	18.63440000	-25.93470000	-13.56980000
H	17.54470000	-25.58220000	-14.88930000
C	16.57730000	-25.69810000	-12.96110000
H	16.81080000	-25.46520000	-11.92130000
H	15.71630000	-25.08400000	-13.22690000
C	16.19600000	-27.17700000	-13.07390000
H	17.02450000	-27.82010000	-12.77650000
H	15.34670000	-27.41130000	-12.43170000
H	15.91960000	-27.43450000	-14.09610000
O	31.85610000	-8.53160000	-21.62600000
O	33.69260000	-9.85380000	-21.46710000
O	33.40510000	-11.55220000	-19.05620000
C	33.23870000	-8.57420000	-21.75670000
C	33.62410000	-8.23730000	-23.20390000
H	33.24710000	-7.24950000	-23.46910000
H	34.70930000	-8.24200000	-23.30690000
H	33.21070000	-8.97070000	-23.89520000
C	33.85560000	-7.58950000	-20.75840000
H	34.94300000	-7.62740000	-20.82440000
H	33.52100000	-6.57650000	-20.98200000
H	33.55910000	-7.83630000	-19.74100000
Si	34.21140000	-11.17180000	-17.61960000
C	36.00450000	-10.75730000	-18.02220000
H	36.52410000	-11.61300000	-18.44820000
H	36.06450000	-9.94840000	-18.74920000
H	36.55330000	-10.45020000	-17.13360000
C	33.39080000	-9.69390000	-16.77060000

H	32.33160000	-9.87300000	-16.59250000
H	33.85150000	-9.48860000	-15.80570000
H	33.48100000	-8.78560000	-17.36400000
C	34.10700000	-12.69070000	-16.46640000
C	34.86600000	-13.85770000	-17.10770000
H	34.81530000	-14.75140000	-16.48690000
H	34.44830000	-14.10170000	-18.08330000
H	35.91870000	-13.61480000	-17.25100000
C	32.63100000	-13.08240000	-16.27900000
H	32.05290000	-12.26790000	-15.84170000
H	32.16600000	-13.33820000	-17.23240000
H	32.52720000	-13.94780000	-15.62420000
C	34.73840000	-12.35120000	-15.10530000
H	34.20490000	-11.54160000	-14.60670000
H	34.72250000	-13.21110000	-14.43510000
H	35.77810000	-12.04080000	-15.21550000
C	31.99360000	-20.70830000	-21.16810000
C	31.99600000	-20.05340000	-24.09560000
O	31.50890000	-19.72270000	-20.61080000
O	31.98420000	-21.11120000	-24.72130000
N	31.38630000	-21.90460000	-21.13010000
N	30.94710000	-19.22400000	-24.09520000
H	31.81690000	-22.68500000	-21.62040000
H	31.03600000	-18.35140000	-23.56890000
C	33.32440000	-20.57220000	-21.94600000
H	33.99580000	-20.04840000	-21.26520000
C	33.20030000	-19.67570000	-23.21490000
H	33.03030000	-18.65750000	-22.87090000
C	33.97200000	-21.93550000	-22.27160000
H	34.02340000	-22.55850000	-21.38000000
H	33.35970000	-22.47130000	-22.99300000
C	34.49830000	-19.64140000	-24.06100000
H	34.25250000	-19.29080000	-25.06530000
C	35.36730000	-21.76080000	-22.87200000
H	36.00210000	-21.24850000	-22.14960000
C	35.29140000	-20.95140000	-24.18120000
H	34.87960000	-21.56700000	-24.97660000
C	29.63780000	-19.57630000	-24.62180000
H	29.06000000	-18.66680000	-24.78000000
H	29.74440000	-20.05690000	-25.59620000
C	30.12650000	-22.16170000	-20.44290000
H	30.25330000	-21.95920000	-19.37820000
H	29.36600000	-21.46970000	-20.80760000
C	28.92490000	-20.50530000	-23.62860000
H	29.57540000	-21.35030000	-23.39850000
H	28.77400000	-19.97600000	-22.68670000
C	27.59160000	-21.05990000	-24.13230000
H	26.88480000	-20.24740000	-24.29600000
H	27.73760000	-21.54400000	-25.09810000
C	27.02770000	-22.06840000	-23.12620000
H	26.90000000	-21.58050000	-22.15910000
H	27.75860000	-22.86270000	-22.97240000
C	25.70150000	-22.69580000	-23.55910000
H	24.94740000	-21.91850000	-23.68080000
H	25.81780000	-23.16820000	-24.53470000
C	25.22970000	-23.73090000	-22.53220000
H	25.13820000	-23.25600000	-21.55470000
H	25.99130000	-24.50370000	-22.42640000
C	23.89720000	-24.38620000	-22.90460000
H	23.12500000	-23.62170000	-22.98890000
H	23.97860000	-24.85100000	-23.88750000
C	23.47950000	-25.43680000	-21.86920000
H	23.41620000	-24.97130000	-20.88530000
H	24.25390000	-26.20080000	-21.79800000
C	22.14110000	-26.10180000	-22.20540000
H	21.36110000	-25.34200000	-22.25730000
H	22.19600000	-26.55400000	-23.19600000
C	21.75560000	-27.16890000	-21.17400000

H	21.71580000	-26.71710000	-20.18280000
H	22.53360000	-27.93160000	-21.13370000
C	20.40940000	-27.83110000	-21.48790000
H	19.62900000	-27.07030000	-21.51160000
H	20.44230000	-28.26850000	-22.48620000
C	20.04220000	-28.91420000	-20.46610000
H	20.02580000	-28.47850000	-19.46710000
H	20.81720000	-29.68090000	-20.45570000
C	18.68510000	-29.56410000	-20.76240000
H	17.90900000	-28.79840000	-20.75610000
H	18.69470000	-29.98330000	-21.76900000
C	18.33010000	-30.66420000	-19.75380000
H	18.33580000	-30.24730000	-18.74660000
H	19.10090000	-31.43520000	-19.77310000
C	16.96320000	-31.30120000	-20.03650000
H	16.18640000	-30.53670000	-19.99370000
H	16.94740000	-31.69910000	-21.05160000
C	16.62340000	-32.42300000	-19.04990000
H	16.58430000	-32.04870000	-18.02710000
H	15.65350000	-32.86450000	-19.27990000
H	17.36560000	-33.22050000	-19.08800000
C	29.67060000	-23.61330000	-20.65940000
H	30.44310000	-24.28910000	-20.28910000
H	29.58150000	-23.80980000	-21.72870000
C	28.33820000	-23.94060000	-19.96680000
H	28.42670000	-23.73040000	-18.90010000
H	27.55680000	-23.28120000	-20.34590000
C	27.91580000	-25.40560000	-20.16510000
H	28.71040000	-26.06060000	-19.80490000
H	27.80790000	-25.61190000	-21.23040000
C	26.60650000	-25.75380000	-19.43990000
H	26.71330000	-25.53050000	-18.37770000
H	25.80380000	-25.11510000	-19.80930000
C	26.20600000	-27.22760000	-19.60900000
H	27.01860000	-27.86650000	-19.26020000
H	26.07420000	-27.44870000	-20.66800000
C	24.91920000	-27.58430000	-18.84940000
H	25.05290000	-27.36330000	-17.78980000
H	24.10420000	-26.94880000	-19.19660000
C	24.51940000	-29.05820000	-19.01300000
H	25.34060000	-29.69670000	-18.68440000
H	24.36420000	-29.27550000	-20.06940000
C	23.24940000	-29.41860000	-18.22830000
H	23.40780000	-29.21150000	-17.16940000
H	22.42980000	-28.77570000	-18.54970000
C	22.83960000	-30.88790000	-18.40350000
H	23.66400000	-31.53480000	-18.10050000
H	22.66200000	-31.08890000	-19.45960000
C	21.58340000	-31.25220000	-17.59960000
H	21.76380000	-31.06340000	-16.54070000
H	20.76210000	-30.59900000	-17.89460000
C	21.16120000	-32.71530000	-17.79200000
H	21.98590000	-33.37260000	-17.51290000
H	20.96530000	-32.89770000	-18.84820000
C	19.91550000	-33.08360000	-16.97440000
H	20.11210000	-32.90950000	-15.91590000
H	19.09340000	-32.42230000	-17.24890000
C	19.48360000	-34.54190000	-17.18010000
H	20.30830000	-35.20660000	-16.91870000
H	19.27470000	-34.71030000	-18.23620000
C	18.24560000	-34.91250000	-16.35220000
H	18.45170000	-34.75150000	-15.29330000
H	17.41880000	-34.25050000	-16.61110000
C	17.80710000	-36.36480000	-16.56410000
H	18.60310000	-37.06210000	-16.30070000
H	16.93930000	-36.60370000	-15.94890000
H	17.53340000	-36.54420000	-17.60330000
O	35.47940000	-18.76670000	-23.55040000

O	36.59410000	-20.49230000	-24.50850000
O	35.88040000	-23.05100000	-23.16570000
C	36.65970000	-19.14680000	-24.17010000
C	36.85830000	-18.32430000	-25.45180000
H	36.86450000	-17.26100000	-25.21080000
H	37.80890000	-18.58840000	-25.91510000
H	36.06170000	-18.51960000	-26.16830000
C	37.80430000	-18.93980000	-23.18380000
H	38.74330000	-19.28010000	-23.61960000
H	37.61060000	-19.49810000	-22.27160000
H	37.88600000	-17.88130000	-22.93840000
Si	37.30040000	-23.71740000	-22.53540000
C	37.89130000	-25.06680000	-23.71220000
H	37.15410000	-25.86210000	-23.80650000
H	38.08460000	-24.66910000	-24.70540000
H	38.81700000	-25.52120000	-23.36370000
C	38.63000000	-22.39350000	-22.36140000
H	38.36500000	-21.65400000	-21.60910000
H	39.58540000	-22.82660000	-22.06980000
H	38.77750000	-21.86620000	-23.30320000
C	36.91620000	-24.52530000	-20.84310000
C	35.67220000	-25.41880000	-20.99190000
H	35.41060000	-25.90230000	-20.05060000
H	34.80600000	-24.84070000	-21.31500000
H	35.82930000	-26.20340000	-21.73270000
C	36.65640000	-23.43920000	-19.78750000
H	37.52940000	-22.80140000	-19.64720000
H	35.82600000	-22.79770000	-20.07250000
H	36.41280000	-23.87510000	-18.81850000
C	38.11840000	-25.37790000	-20.40040000
H	39.02740000	-24.77890000	-20.33270000
H	37.94760000	-25.82760000	-19.42190000
H	38.31280000	-26.19140000	-21.09980000
C	32.41350000	-25.47470000	-23.21550000
C	31.20270000	-25.01690000	-25.90240000
O	32.14550000	-24.56140000	-22.43680000
O	31.11480000	-26.12110000	-26.43700000
N	31.88360000	-26.69640000	-23.08770000
N	30.13140000	-24.28520000	-25.56640000
H	32.12040000	-27.37490000	-23.79690000
H	30.30010000	-23.38460000	-25.13570000
C	33.32350000	-25.21300000	-24.43170000
H	34.11780000	-24.56320000	-24.06720000
C	32.58030000	-24.43360000	-25.55240000
H	32.39580000	-23.43830000	-25.16170000
C	34.00300000	-26.49170000	-24.96250000
H	34.53140000	-26.99620000	-24.15210000
H	33.26330000	-27.19820000	-25.34080000
C	33.42860000	-24.24990000	-26.83060000
H	32.77270000	-23.98960000	-27.66400000
C	34.99440000	-26.17430000	-26.08530000
H	35.80420000	-25.56810000	-25.68160000
C	34.32080000	-25.42640000	-27.25910000
H	33.75840000	-26.12620000	-27.87840000
C	28.75160000	-24.70220000	-25.77790000
H	28.11060000	-23.82110000	-25.76460000
H	28.65590000	-25.13990000	-26.77380000
C	30.86850000	-27.06020000	-22.11240000
H	31.30380000	-27.05320000	-21.11190000
H	30.07680000	-26.30940000	-22.12370000
C	28.29430000	-25.70610000	-24.70620000
H	29.00600000	-26.53080000	-24.65720000
H	28.30380000	-25.23110000	-23.72460000
C	26.90180000	-26.27740000	-25.00150000
H	26.16800000	-25.47210000	-25.02340000
H	26.90380000	-26.71760000	-25.99980000
C	26.47120000	-27.34300000	-23.98550000
H	26.42260000	-26.90130000	-22.99030000

H	27.22860000	-28.12640000	-23.94040000
C	25.11710000	-27.96970000	-24.34330000
H	24.35280000	-27.19320000	-24.37910000
H	25.16920000	-28.39490000	-25.34660000
C	24.69160000	-29.06120000	-23.35400000
H	24.63500000	-28.63920000	-22.35090000
H	25.45580000	-29.83840000	-23.32160000
C	23.34110000	-29.68860000	-23.72410000
H	22.57410000	-28.91400000	-23.74730000
H	23.39490000	-30.09840000	-24.73360000
C	22.92060000	-30.79440000	-22.74840000
H	22.88060000	-30.38890000	-21.73770000
H	23.68060000	-31.57650000	-22.73950000
C	21.56040000	-31.40760000	-23.10650000
H	20.79790000	-30.62820000	-23.10300000
H	21.59440000	-31.80090000	-24.12340000
C	21.15170000	-32.52660000	-22.14060000
H	21.13580000	-32.13750000	-21.12260000
H	21.90620000	-33.31370000	-22.16040000
C	19.77990000	-33.12530000	-22.47820000
H	19.02250000	-32.34180000	-22.44230000
H	19.78800000	-33.49970000	-23.50270000
C	19.38790000	-34.26040000	-21.52360000
H	19.39920000	-33.89100000	-20.49820000
H	20.13690000	-35.05130000	-21.57710000
C	18.00460000	-34.84490000	-21.83870000
H	17.25180000	-34.05960000	-21.76040000
H	17.98290000	-35.19100000	-22.87290000
C	17.63300000	-36.00510000	-20.90570000
H	17.67220000	-35.66580000	-19.87050000
H	18.37710000	-36.79690000	-21.00100000
C	16.23930000	-36.57460000	-21.20270000
H	15.49020000	-35.79070000	-21.08320000
H	16.18790000	-36.89380000	-22.24450000
C	15.88350000	-37.75820000	-20.29660000
H	15.88820000	-37.46830000	-19.24570000
H	14.88990000	-38.14180000	-20.52960000
H	16.59200000	-38.57720000	-20.42310000
C	30.28570000	-28.44020000	-22.44720000
H	31.07350000	-29.19240000	-22.38540000
H	29.93470000	-28.44590000	-23.48040000
C	29.12530000	-28.83830000	-21.52550000
H	29.46270000	-28.83850000	-20.48810000
H	28.33750000	-28.08800000	-21.59450000
C	28.54900000	-30.21590000	-21.88340000
H	29.33460000	-30.96890000	-21.80600000
H	28.22690000	-30.21530000	-22.92580000
C	27.36820000	-30.61830000	-20.98990000
H	27.68440000	-30.62490000	-19.94590000
H	26.58220000	-29.86760000	-21.06950000
C	26.79820000	-31.99380000	-21.36440000
H	27.58710000	-32.74430000	-21.29690000
H	26.47420000	-31.98150000	-22.40610000
C	25.62240000	-32.41050000	-20.47080000
H	25.94560000	-32.43110000	-19.42910000
H	24.83640000	-31.65860000	-20.53420000
C	25.04820000	-33.78030000	-20.85890000
H	25.83790000	-34.53160000	-20.81370000
H	24.70920000	-33.75180000	-21.89550000
C	23.88590000	-34.20910000	-19.95340000
H	24.22660000	-34.24770000	-18.91770000
H	23.10140000	-33.45390000	-19.99070000
C	23.29940000	-35.57030000	-20.35290000
H	24.08760000	-36.32440000	-20.33790000
H	22.93790000	-35.52260000	-21.38110000
C	22.15590000	-36.01010000	-19.42870000
H	22.51910000	-36.06490000	-18.40150000
H	21.37300000	-35.25250000	-19.43740000

C	21.55650000	-37.36380000	-19.83430000
H	22.34250000	-38.12020000	-19.84660000
H	21.17300000	-37.30070000	-20.85370000
C	20.43190000	-37.81280000	-18.89130000
H	20.81680000	-37.88090000	-17.87290000
H	19.65030000	-37.05370000	-18.87370000
C	19.82140000	-39.16020000	-19.30090000
H	20.60530000	-39.91790000	-19.33850000
H	19.41780000	-39.08350000	-20.31160000
C	18.71460000	-39.61930000	-18.34140000
H	19.11620000	-39.70410000	-17.33070000
H	17.93020000	-38.86370000	-18.29690000
C	18.09720000	-40.95960000	-18.75500000
H	18.84660000	-41.75150000	-18.77180000
H	17.31440000	-41.25870000	-18.05750000
H	17.65060000	-40.89950000	-19.74800000
O	34.35440000	-23.19770000	-26.70170000
O	35.33140000	-24.76370000	-28.00880000
O	35.49960000	-27.43960000	-26.47810000
C	35.29830000	-23.41150000	-27.68670000
C	34.91530000	-22.61750000	-28.94280000
H	34.84210000	-21.55680000	-28.70190000
H	35.67520000	-22.75530000	-29.71200000
H	33.95840000	-22.95640000	-29.33790000
C	36.65780000	-22.98410000	-27.13370000
H	37.44100000	-23.17400000	-27.86690000
H	36.64420000	-21.91990000	-26.89710000
H	36.87520000	-23.53230000	-26.22030000
Si	36.75080000	-27.73980000	-27.57550000
C	36.08720000	-27.65930000	-29.34070000
H	35.21140000	-28.29520000	-29.45980000
H	35.79420000	-26.64780000	-29.61490000
H	36.83350000	-27.98650000	-30.06220000
C	38.16100000	-26.52080000	-27.29710000
H	38.50880000	-26.54720000	-26.26610000
H	39.01170000	-26.74370000	-27.93850000
H	37.84780000	-25.50340000	-27.51490000
C	37.36310000	-29.51240000	-27.21050000
C	36.19740000	-30.49510000	-27.41460000
H	36.49230000	-31.51900000	-27.18420000
H	35.35240000	-30.24220000	-26.77220000
H	35.83830000	-30.48080000	-28.44400000
C	37.84480000	-29.58090000	-25.75090000
H	38.68030000	-28.90320000	-25.57330000
H	37.04770000	-29.30230000	-25.05990000
H	38.17510000	-30.58540000	-25.48590000
C	38.52010000	-29.86990000	-28.15940000
H	39.36120000	-29.18660000	-28.03850000
H	38.88930000	-30.87850000	-27.97170000
H	38.20890000	-29.82530000	-29.20340000
C	30.71830000	-15.63800000	-19.41910000
C	31.04810000	-15.67570000	-22.39990000
O	30.07300000	-14.65850000	-19.04440000
O	31.32070000	-16.81380000	-22.77420000
N	30.25460000	-16.89180000	-19.31040000
N	29.87640000	-15.09500000	-22.68770000
H	30.81980000	-17.64410000	-19.69180000
H	29.76210000	-14.11790000	-22.41310000
C	32.10590000	-15.43560000	-20.05690000
H	32.57950000	-14.65790000	-19.45610000
C	32.02880000	-14.88860000	-21.50950000
H	31.63690000	-13.87520000	-21.44560000
C	32.99390000	-16.69350000	-19.95640000
H	32.98000000	-17.07450000	-18.93730000
H	32.60500000	-17.48690000	-20.59040000
C	33.42990000	-14.77140000	-22.15940000
H	33.31990000	-14.69410000	-23.24280000
C	34.42940000	-16.39330000	-20.39030000

H	34.85240000	-15.65070000	-19.71830000
C	34.46490000	-15.86870000	-21.84200000
H	34.36220000	-16.69480000	-22.54370000
C	28.72970000	-15.76660000	-23.28780000
H	28.04830000	-15.00650000	-23.66900000
H	29.05270000	-16.35770000	-24.14580000
C	28.98240000	-17.25440000	-18.70600000
H	29.00540000	-16.99230000	-17.64700000
H	28.18210000	-16.66820000	-19.15930000
C	28.01180000	-16.64930000	-22.25050000
H	28.70750000	-17.40550000	-21.88350000
H	27.74450000	-16.03610000	-21.38930000
C	26.75110000	-17.34980000	-22.78100000
H	26.02190000	-16.60510000	-23.09830000
H	27.00050000	-17.93230000	-23.66730000
C	26.12470000	-18.26730000	-21.71890000
H	25.92250000	-17.68620000	-20.81880000
H	26.85050000	-19.02920000	-21.43390000
C	24.82930000	-18.95650000	-22.17340000
H	24.07670000	-18.20570000	-22.41170000
H	25.00840000	-19.51170000	-23.09360000
C	24.28550000	-19.90890000	-21.09820000
H	24.12990000	-19.35410000	-20.17260000
H	25.03680000	-20.66710000	-20.87720000
C	22.97580000	-20.60350000	-21.49770000
H	22.20330000	-19.85550000	-21.67430000
H	23.11070000	-21.13240000	-22.44090000
C	22.50270000	-21.59110000	-20.42200000
H	22.38670000	-21.06280000	-19.47550000
H	23.27420000	-22.34400000	-20.26020000
C	21.18430000	-22.29170000	-20.77750000
H	20.39820000	-21.54730000	-20.90400000
H	21.28330000	-22.79890000	-21.73720000
C	20.76700000	-23.30650000	-19.70460000
H	20.68480000	-22.79960000	-18.74310000
H	21.55110000	-24.05520000	-19.59120000
C	19.44120000	-24.00990000	-20.02410000
H	18.64770000	-23.26750000	-20.11070000
H	19.50980000	-24.50020000	-20.99530000
C	19.06500000	-25.04490000	-18.95530000
H	19.01170000	-24.55500000	-17.98300000
H	19.85520000	-25.79210000	-18.88060000
C	17.73170000	-25.74610000	-19.24660000
H	16.93650000	-25.00270000	-19.30330000
H	17.77490000	-26.22390000	-20.22570000
C	17.38120000	-26.79490000	-18.18250000
H	17.35330000	-26.31900000	-17.20220000
H	18.17150000	-27.54470000	-18.13830000
C	16.03880000	-27.48740000	-18.45310000
H	15.24050000	-26.74470000	-18.47740000
H	16.05550000	-27.95180000	-19.43940000
C	15.70680000	-28.55150000	-17.40180000
H	15.63900000	-28.11300000	-16.40650000
H	14.75180000	-29.02990000	-17.61970000
H	16.46780000	-29.33150000	-17.37380000
C	28.70650000	-18.75610000	-18.88130000
H	29.51740000	-19.33100000	-18.43090000
H	28.71280000	-19.00700000	-19.94270000
C	27.36960000	-19.18050000	-18.25530000
H	27.38290000	-18.94310000	-17.19060000
H	26.56150000	-18.59060000	-18.68820000
C	27.06040000	-20.67390000	-18.44110000
H	27.87430000	-21.26870000	-18.02420000
H	27.02120000	-20.90920000	-19.50530000
C	25.73750000	-21.07440000	-17.76980000
H	25.78950000	-20.83550000	-16.70660000
H	24.92420000	-20.47360000	-18.17720000
C	25.39610000	-22.56160000	-17.93700000

H	26.21580000	-23.16770000	-17.54950000
H	25.31010000	-22.79900000	-18.99780000
C	24.09500000	-22.93940000	-17.21190000
H	24.19040000	-22.69450000	-16.15310000
H	23.27380000	-22.33230000	-17.59300000
C	23.73260000	-24.42320000	-17.35540000
H	24.55990000	-25.03410000	-16.99260000
H	23.60750000	-24.66600000	-18.41080000
C	22.45460000	-24.78760000	-16.58470000
H	22.58670000	-24.54030000	-15.53060000
H	21.62460000	-24.17750000	-16.94140000
C	22.08240000	-26.27000000	-16.71290000
H	22.91790000	-26.88280000	-16.37270000
H	21.92670000	-26.51450000	-17.76360000
C	20.82580000	-26.63150000	-15.90780000
H	20.98700000	-26.38690000	-14.85720000
H	19.98740000	-26.01980000	-16.24140000
C	20.45080000	-28.11350000	-16.03110000
H	21.29480000	-28.72750000	-15.71500000
H	20.26830000	-28.35390000	-17.07830000
C	19.21510000	-28.47950000	-15.19740000
H	19.40180000	-28.24140000	-14.14950000
H	18.36900000	-27.86580000	-15.50730000
C	18.83890000	-29.96100000	-15.32250000
H	19.68970000	-30.57740000	-15.02960000
H	18.63330000	-30.19350000	-16.36720000
C	17.62150000	-30.33220000	-14.46530000
H	17.82990000	-30.11140000	-13.41780000
H	16.76970000	-29.71320000	-14.74790000
C	17.23490000	-31.80770000	-14.60060000
H	18.05360000	-32.45970000	-14.29560000
H	16.37250000	-32.04370000	-13.97670000
H	16.97500000	-32.05210000	-15.63010000
O	34.10050000	-13.61170000	-21.72550000
O	35.69710000	-15.18680000	-22.03080000
O	35.18010000	-17.59620000	-20.31940000
C	35.43730000	-13.82220000	-22.00710000
C	35.76780000	-13.23410000	-23.38540000
H	35.52800000	-12.17050000	-23.40030000
H	36.82960000	-13.36230000	-23.59550000
H	35.19810000	-13.73620000	-24.16640000
C	36.25590000	-13.16890000	-20.89520000
H	37.31550000	-13.38610000	-21.02840000
H	36.10230000	-12.08930000	-20.91270000
H	35.92890000	-13.54090000	-19.92770000
Si	36.03160000	-18.18270000	-18.97880000
C	35.00380000	-17.98820000	-17.40960000
H	34.71780000	-16.95150000	-17.24650000
H	34.09130000	-18.58070000	-17.45810000
H	35.55900000	-18.31500000	-16.53180000
C	36.32620000	-20.01760000	-19.27470000
H	36.93880000	-20.17860000	-20.15820000
H	36.83390000	-20.48350000	-18.43190000
H	35.38520000	-20.54400000	-19.42000000
C	37.71850000	-17.29480000	-18.77280000
C	37.50910000	-15.87760000	-18.22230000
H	38.45780000	-15.35840000	-18.08460000
H	36.91170000	-15.27830000	-18.90390000
H	37.00310000	-15.89490000	-17.25710000
C	38.41860000	-17.21620000	-20.13630000
H	38.62380000	-18.21080000	-20.53070000
H	37.79750000	-16.69120000	-20.86430000
H	39.36900000	-16.68660000	-20.07100000
C	38.59280000	-18.08940000	-17.78630000
H	38.80440000	-19.09290000	-18.15650000
H	39.55200000	-17.59800000	-17.62080000
H	38.10570000	-18.19320000	-16.81610000

cis-2 hexamer

C	29.65290	-9.69750	-16.20160
C	30.34380	-9.81760	-19.12500
O	28.96230	-8.70610	-15.97190
O	30.61020	-10.98540	-19.40980
N	29.17910	-10.94750	-16.09780
N	29.26240	-9.18560	-19.60320
H	29.81670	-11.70280	-16.30950
H	29.11860	-8.22100	-19.32350
C	31.11710	-9.53580	-16.67030
H	31.54500	-8.76120	-16.03220
C	31.23060	-9.02660	-18.13990
H	30.87650	-7.99650	-18.15390
C	31.94100	-10.82380	-16.44310
H	31.83850	-11.15490	-15.41100
H	31.56120	-11.63740	-17.06070
C	32.69390	-8.99410	-18.64860
H	32.67940	-8.92650	-19.73830
C	33.41770	-10.61690	-16.77600
H	33.83640	-9.87110	-16.10060
C	33.58640	-10.17330	-18.24230
H	33.40610	-11.01230	-18.90640
C	28.20680	-9.81410	-20.38480
H	27.63120	-9.03260	-20.88100
H	28.64220	-10.43450	-21.17020
C	27.81670	-11.31920	-15.76000
H	27.63420	-11.09740	-14.70770
H	27.11270	-10.72490	-16.34440
C	27.29380	-10.64870	-19.47450
H	27.89560	-11.39270	-18.95140
H	26.86390	-10.00660	-18.70430
C	26.16840	-11.36860	-20.22590
H	25.49380	-10.63900	-20.67260
H	26.58540	-11.94520	-21.05160
C	25.39240	-12.30190	-19.28970
H	24.97990	-11.72300	-18.46250
H	26.08620	-13.01760	-18.84750
C	24.26440	-13.06940	-19.98760
H	23.53230	-12.36810	-20.38620
H	24.66170	-13.61410	-20.84450
C	23.58280	-14.04940	-19.02540
H	23.20930	-13.50420	-18.15800
H	24.32420	-14.75420	-18.64820
C	22.43210	-14.82940	-19.67000
H	21.66400	-14.13530	-20.00860
H	22.78980	-15.34520	-20.56160
C	21.82530	-15.84640	-18.69520
H	21.49460	-15.33170	-17.79270
H	22.59700	-16.54940	-18.38140
C	20.65050	-16.62450	-19.29730
H	19.85430	-15.93070	-19.56460
H	20.96300	-17.10110	-20.22690
C	20.11310	-17.68790	-18.33110
H	19.83030	-17.21470	-17.39060
H	20.90810	-18.39500	-18.09280
C	18.91050	-18.44940	-18.89950
H	18.09810	-17.74920	-19.09170
H	19.17380	-18.88250	-19.86480
C	18.42680	-19.55820	-17.95610
H	18.19050	-19.12880	-16.98240
H	19.23490	-20.27200	-17.79150
C	17.19640	-20.29530	-18.49870
H	16.37760	-19.58690	-18.62310
H	17.41460	-20.68740	-19.49250
C	16.75040	-21.44310	-17.58350
H	16.54830	-21.05360	-16.58560

H	17.56650	-22.15900	-17.47730
C	15.50280	-22.16400	-18.11090
H	14.67290	-21.45940	-18.17640
H	15.68500	-22.51870	-19.12560
C	15.09320	-23.34770	-17.22910
H	14.84730	-23.01690	-16.22030
H	14.21690	-23.85210	-17.63650
H	15.89520	-24.08280	-17.15700
C	27.61410	-12.81400	-16.04290
H	28.34520	-13.39270	-15.47590
H	27.81650	-13.01780	-17.09520
C	26.20370	-13.29870	-15.69020
H	26.01360	-13.10910	-14.63300
H	25.46600	-12.71800	-16.24450
C	26.00880	-14.79180	-15.98750
H	26.77620	-15.36940	-15.47030
H	26.15340	-14.97450	-17.05290
C	24.62340	-15.28930	-15.55670
H	24.49250	-15.10290	-14.49000
H	23.85180	-14.71130	-16.06570
C	24.41160	-16.78150	-15.84100
H	25.20610	-17.36060	-15.36840
H	24.49410	-16.96110	-16.91290
C	23.04960	-17.27390	-15.33370
H	22.97580	-17.08500	-14.26200
H	22.25440	-16.69410	-15.80280
C	22.81520	-18.76510	-15.60270
H	23.62230	-19.34840	-15.15760
H	22.85590	-18.94720	-16.67590
C	21.46790	-19.24800	-15.04850
H	21.43060	-19.05940	-13.97490
H	20.66170	-18.66330	-15.49190
C	21.21560	-20.73730	-15.31200
H	22.02500	-21.32650	-14.87950
H	21.23780	-20.91990	-16.38570
C	19.87370	-21.21220	-14.73840
H	19.85560	-21.03150	-13.66300
H	19.06540	-20.61810	-15.16500
C	19.60500	-22.69730	-15.01020
H	20.41450	-23.29650	-14.59170
H	19.61350	-22.87170	-16.08580
C	18.26640	-23.16750	-14.42510
H	18.26440	-23.00280	-13.34700
H	17.45860	-22.55960	-14.83230
C	17.97920	-24.64550	-14.71620
H	18.78720	-25.25900	-14.31610
H	17.97390	-24.80310	-15.79470
C	16.64270	-25.11280	-14.12360
H	16.65370	-24.97660	-13.04170
H	15.83420	-24.49000	-14.50500
C	16.33390	-26.57720	-14.44470
H	17.10070	-27.23820	-14.04040
H	15.37720	-26.87680	-14.01660
H	16.28080	-26.73810	-15.52140
O	33.45060	-7.89920	-18.18300
O	34.88770	-9.64220	-18.42020
O	34.05690	-11.86980	-16.60630
C	34.77260	-8.25670	-18.42790
C	35.20520	-7.73480	-19.80910
H	35.07900	-6.65260	-19.85320
H	36.25450	-7.97530	-19.98200
H	34.61500	-8.19150	-20.60270
C	35.64610	-7.68440	-17.31310
H	36.67730	-8.01560	-17.43670
H	35.28000	-8.01650	-16.34440
H	35.61710	-6.59650	-17.35060
Si	35.30550	-12.21180	-15.51410
C	36.11370	-13.82680	-16.07230

H	35.39560	-14.64520	-16.08170
H	36.51760	-13.73220	-17.07530
H	36.93610	-14.11650	-15.41970
C	36.57810	-10.80910	-15.54820
H	36.16380	-9.88100	-15.15830
H	37.45420	-11.05050	-14.94760
H	36.91990	-10.61190	-16.56410
C	34.59810	-12.46680	-13.75040
C	33.44490	-13.48400	-13.81560
H	33.00620	-13.65200	-12.83170
H	32.64760	-13.14440	-14.47640
H	33.78440	-14.44950	-14.19160
C	34.09140	-11.13190	-13.18340
H	34.89310	-10.39600	-13.11840
H	33.30540	-10.70730	-13.80230
H	33.68170	-11.25620	-12.18080
C	35.70920	-13.00650	-12.83230
H	36.56100	-12.32650	-12.79560
H	35.35000	-13.13700	-11.81100
H	36.07560	-13.97560	-13.17120
C	27.50830	-4.67380	-15.93920
C	28.46900	-5.27760	-18.73600
O	26.68270	-3.76160	-15.91080
O	28.96510	-6.40060	-18.79700
N	27.18460	-5.94330	-15.64380
N	27.32360	-4.95420	-19.35410
H	27.90650	-6.64950	-15.71100
H	26.99960	-3.99720	-19.26660
C	28.96570	-4.37410	-16.35900
H	29.20010	-3.41770	-15.88830
C	29.13320	-4.17170	-17.89300
H	28.61100	-3.25180	-18.14570
C	29.97590	-5.39940	-15.80650
H	29.79950	-5.55190	-14.74400
H	29.84080	-6.36670	-16.28540
C	30.61260	-3.96370	-18.31310
H	30.70890	-4.17510	-19.37980
C	31.41580	-4.93240	-16.04700
H	31.57120	-3.98630	-15.53360
C	31.69090	-4.76140	-17.55520
H	31.83820	-5.73510	-18.02230
C	26.44880	-5.88820	-20.04940
H	25.80730	-5.31630	-20.71940
H	27.03680	-6.56170	-20.67490
C	25.84900	-6.39150	-15.27270
H	25.60730	-6.00680	-14.28090
H	25.11480	-5.97480	-15.96430
C	25.59540	-6.68040	-19.04620
H	26.25370	-7.25250	-18.39140
H	25.05190	-5.98370	-18.40640
C	24.59860	-7.63650	-19.71370
H	23.91020	-7.06690	-20.33720
H	25.13350	-8.31070	-20.38220
C	23.80890	-8.45310	-18.68040
H	23.31370	-7.77380	-17.98550
H	24.50620	-9.04670	-18.08900
C	22.76160	-9.38190	-19.31020
H	22.03590	-8.78900	-19.86530
H	23.24210	-10.03360	-20.03890
C	22.03540	-10.23480	-18.25970
H	21.58470	-9.58120	-17.51250
H	22.76460	-10.84980	-17.73240
C	20.95030	-11.13930	-18.86120
H	20.19230	-10.52440	-19.34440
H	21.38240	-11.75870	-19.64600
C	20.28970	-12.03970	-17.80770
H	19.88410	-11.42110	-17.00730
H	21.04840	-12.67580	-17.35180

C	19.17100	-12.91870	-18.38440
H	18.38760	-12.28330	-18.79550
H	19.55560	-13.50330	-19.21920
C	18.57090	-13.86520	-17.33540
H	18.21150	-13.28270	-16.48760
H	19.35290	-14.52000	-16.95070
C	17.42040	-14.71840	-17.88700
H	16.61870	-14.06480	-18.22910
H	17.75820	-15.26900	-18.76450
C	16.87360	-15.70620	-16.84730
H	16.55800	-15.15840	-15.95970
H	17.67290	-16.37600	-16.52940
C	15.69540	-16.53520	-17.37590
H	14.88170	-15.86690	-17.65640
H	15.99110	-17.05410	-18.28750
C	15.19170	-17.55930	-16.34960
H	14.91310	-17.04410	-15.43040
H	16.00360	-18.23950	-16.08970
C	13.99260	-18.36930	-16.86090
H	13.16320	-17.69630	-17.08110
H	14.25040	-18.85660	-17.80130
C	13.53060	-19.43050	-15.85730
H	13.21560	-18.97290	-14.91960
H	12.68620	-19.99640	-16.25090
H	14.32940	-20.13880	-15.63600
C	25.76810	-7.92540	-15.28500
H	26.50170	-8.33460	-14.58890
H	26.04580	-8.29680	-16.27180
C	24.37150	-8.44770	-14.91700
H	24.11500	-8.11240	-13.91130
H	23.62970	-8.00730	-15.58410
C	24.27500	-9.97940	-14.98900
H	25.03390	-10.42310	-14.34330
H	24.50350	-10.31090	-16.00230
C	22.89040	-10.50050	-14.57760
H	22.68290	-10.19760	-13.55050
H	22.12500	-10.03000	-15.19490
C	22.76880	-12.02690	-14.69590
H	23.55430	-12.50210	-14.10730
H	22.93590	-12.32590	-15.73120
C	21.40120	-12.54110	-14.22480
H	21.25310	-12.26330	-13.18040
H	20.61140	-12.04440	-14.78780
C	21.24850	-14.06080	-14.37620
H	22.05520	-14.56350	-13.84170
H	21.35600	-14.33360	-15.42640
C	19.89820	-14.56410	-13.84740
H	19.80610	-14.30640	-12.79150
H	19.08960	-14.04470	-14.36080
C	19.71340	-16.07700	-14.02180
H	20.53460	-16.60300	-13.53380
H	19.76970	-16.32850	-15.08120
C	18.37930	-16.56990	-13.44490
H	18.33420	-16.32900	-12.38210
H	17.55780	-16.03190	-13.91680
C	18.16770	-18.07660	-13.63640
H	18.99810	-18.62060	-13.18530
H	18.18450	-18.31030	-14.70100
C	16.84750	-18.56340	-13.02380
H	16.83840	-18.33850	-11.95660
H	16.01770	-18.01010	-13.46230
C	16.61570	-20.06480	-13.23200
H	17.45090	-20.62360	-12.80870
H	16.60570	-20.28140	-14.30030
C	15.30500	-20.54980	-12.59830
H	15.31850	-20.34790	-11.52670
H	14.46860	-19.98470	-13.00830
C	15.05560	-22.04240	-12.82840

H	15.85470	-22.64560	-12.39730
H	14.11760	-22.35620	-12.37010
H	14.99710	-22.26850	-13.89270
O	31.04520	-2.63660	-18.12390
O	32.84480	-3.94590	-17.70780
O	32.30520	-5.91220	-15.53490
C	32.42910	-2.69380	-18.14970
C	32.93110	-2.48350	-19.58880
H	32.57020	-1.52830	-19.97160
H	34.02120	-2.47970	-19.60180
H	32.58410	-3.28060	-20.24520
C	32.96680	-1.61620	-17.20520
H	34.05330	-1.67800	-17.14250
H	32.68450	-0.62840	-17.57100
H	32.54410	-1.74570	-16.21340
Si	32.89700	-6.00470	-13.94610
C	31.55220	-5.52250	-12.69990
H	31.15650	-4.52890	-12.90060
H	30.71750	-6.22220	-12.71990
H	31.93910	-5.51620	-11.68170
C	33.37460	-7.80820	-13.64290
H	34.16300	-8.13360	-14.31840
H	33.73010	-7.96530	-12.62530
H	32.52040	-8.46650	-13.79360
C	34.43250	-4.87600	-13.72300
C	34.01690	-3.40020	-13.79530
H	34.87110	-2.73860	-13.64910
H	33.59010	-3.16470	-14.76750
H	33.27900	-3.15460	-13.03180
C	35.44660	-5.16960	-14.83650
H	35.78030	-6.20650	-14.80530
H	35.00860	-4.98470	-15.81810
H	36.33100	-4.53830	-14.74790
C	35.07320	-5.15580	-12.35200
H	35.40470	-6.19110	-12.26870
H	35.94510	-4.52220	-12.18590
H	34.37290	-4.96730	-11.53780
C	24.51320	0.16860	-17.38040
C	26.12890	-1.01170	-19.63070
O	23.65280	0.93910	-17.80310
O	26.68230	-2.05110	-19.27640
N	24.22210	-1.00090	-16.79030
N	25.13420	-0.99770	-20.53040
H	25.00340	-1.57210	-16.49700
H	24.75510	-0.09600	-20.79650
C	26.01030	0.51390	-17.54130
H	26.09610	1.57340	-17.29570
C	26.52660	0.34230	-19.00210
H	26.05680	1.11830	-19.60560
C	26.88070	-0.26250	-16.52960
H	26.48920	-0.12980	-15.52220
H	26.84150	-1.33140	-16.73480
C	28.05750	0.56950	-19.11040
H	28.40270	0.16950	-20.06580
C	28.34140	0.17360	-16.57260
H	28.41530	1.21760	-16.27050
C	28.92750	-0.01610	-17.98620
H	29.12230	-1.06800	-18.17310
C	24.48570	-2.18200	-21.08380
H	23.90360	-1.87520	-21.95240
H	25.23680	-2.88830	-21.44100
C	22.90050	-1.54930	-16.54710
H	22.41770	-0.97720	-15.75390
H	22.28350	-1.45130	-17.44090
C	23.56880	-2.84280	-20.04000
H	24.17260	-3.18280	-19.19800
H	22.88420	-2.09240	-19.64380
C	22.75500	-4.02880	-20.57820

H	22.10070	-3.68920	-21.38030
H	23.42350	-4.76840	-21.01830
C	21.91670	-4.68360	-19.47020
H	21.32160	-3.91700	-18.97330
H	22.58680	-5.09250	-18.71340
C	20.97950	-5.79230	-19.97090
H	20.27330	-5.37720	-20.68890
H	21.55320	-6.54640	-20.50880
C	20.20950	-6.45220	-18.81710
H	19.68840	-5.68290	-18.24660
H	20.92050	-6.91500	-18.13230
C	19.19360	-7.50530	-19.28250
H	18.44490	-7.03380	-19.91810
H	19.69310	-8.24920	-19.90240
C	18.50020	-8.20160	-18.10210
H	18.03620	-7.45090	-17.46210
H	19.25020	-8.70520	-17.49190
C	17.43700	-9.21840	-18.54150
H	16.65230	-8.70610	-19.09740
H	17.87890	-9.93620	-19.23220
C	16.81930	-9.96850	-17.35270
H	16.40540	-9.24880	-16.64660
H	17.60300	-10.50700	-16.81970
C	15.72130	-10.95500	-17.77480
H	14.91070	-10.40950	-18.25720
H	16.11350	-11.64160	-18.52490
C	15.16690	-11.75930	-16.59080
H	14.79830	-11.07320	-15.82850
H	15.97470	-12.32760	-16.12940
C	14.03970	-12.71710	-17.00110
H	13.21400	-12.14370	-17.42160
H	14.38910	-13.37490	-17.79690
C	13.53130	-13.56620	-15.82810
H	13.19980	-12.91030	-15.02320
H	14.35450	-14.15710	-15.42570
C	12.38190	-14.50090	-16.23000
H	11.54040	-13.91140	-16.59500
H	12.69430	-15.13440	-17.06000
C	11.91240	-15.38760	-15.07260
H	11.54660	-14.78610	-14.24080
H	11.10200	-16.04410	-15.38980
H	12.72260	-16.01690	-14.70400
C	23.03390	-3.02670	-16.14620
H	23.69620	-3.11300	-15.28320
H	23.51610	-3.58050	-16.95210
C	21.68820	-3.67940	-15.81250
H	21.24180	-3.16190	-14.96250
H	21.00100	-3.54710	-16.64810
C	21.81810	-5.17670	-15.49510
H	22.54230	-5.32230	-14.69240
H	22.21480	-5.70170	-16.36470
C	20.47370	-5.79160	-15.08660
H	20.11680	-5.29860	-14.18140
H	19.73110	-5.58700	-15.85790
C	20.54390	-7.30600	-14.85050
H	21.31310	-7.52980	-14.11020
H	20.84660	-7.80470	-15.77170
C	19.19600	-7.86400	-14.37440
H	18.92900	-7.39520	-13.42650
H	18.41630	-7.58450	-15.08290
C	19.19410	-9.38810	-14.20880
H	19.99370	-9.68620	-13.52970
H	19.41150	-9.85570	-15.16930
C	17.84990	-9.90070	-13.67410
H	17.66100	-9.46150	-12.69380
H	17.04490	-9.55730	-14.32360
C	17.78910	-11.42860	-13.57120
H	18.61140	-11.78760	-12.95130

H	17.93460	-11.86020	-14.56150
C	16.45720	-11.91650	-12.98570
H	16.33420	-11.51220	-11.98020
H	15.63210	-11.52250	-13.57860
C	16.35460	-13.44500	-12.94080
H	17.19530	-13.85140	-12.37740
H	16.43990	-13.83830	-13.95370
C	15.03990	-13.92470	-12.31160
H	14.97250	-13.55680	-11.28700
H	14.19820	-13.49070	-12.85080
C	14.90640	-15.45190	-12.31970
H	15.75970	-15.89560	-11.80550
H	14.94480	-15.80740	-13.34950
C	13.60730	-15.93210	-11.65930
H	13.58120	-15.60430	-10.61940
H	12.75250	-15.46780	-12.15010
C	13.44730	-17.45350	-11.71620
H	14.26950	-17.95560	-11.20600
H	12.51840	-17.76610	-11.23890
H	13.42490	-17.80410	-12.74780
O	28.43410	1.92860	-19.07200
O	30.11400	0.75010	-18.09630
O	29.02350	-0.66830	-15.65930
C	29.79600	1.91270	-18.78960
C	30.59580	1.93400	-20.10310
H	30.33210	2.81810	-20.68430
H	31.66360	1.96030	-19.88460
H	30.38990	1.04570	-20.69920
C	30.12600	3.11890	-17.90900
H	31.17530	3.09110	-17.61450
H	29.50690	3.11530	-17.01430
H	29.93960	4.03660	-18.46450
Si	29.87550	-0.16590	-14.28420
C	30.76520	-1.68630	-13.60050
H	30.05760	-2.47350	-13.34440
H	31.45430	-2.09710	-14.32950
H	31.34060	-1.45350	-12.70580
C	31.12780	1.16240	-14.78810
H	30.63250	2.07340	-15.11930
H	31.78370	1.43000	-13.96060
H	31.75710	0.82070	-15.60930
C	28.68950	0.48140	-12.91980
C	27.66230	-0.61260	-12.57790
H	26.94880	-0.26730	-11.82920
H	27.09480	-0.92040	-13.45550
H	28.14640	-1.50420	-12.17870
C	27.96470	1.75050	-13.39170
H	28.67050	2.52830	-13.68380
H	27.31890	1.54940	-14.24250
H	27.33340	2.16270	-12.60410
C	29.51120	0.82080	-11.66300
H	30.26640	1.57890	-11.87330
H	28.87490	1.20610	-10.86570
H	30.02580	-0.05580	-11.26930
C	21.72900	3.80490	-20.30790
C	23.76380	2.49990	-22.14140
O	20.83200	4.22010	-21.03990
O	24.52430	1.71220	-21.58250
N	21.52210	2.86130	-19.37590
N	22.81500	2.11160	-23.00540
H	22.31480	2.53860	-18.83600
H	22.24160	2.83400	-23.41750
C	23.15430	4.37330	-20.47020
H	23.01100	5.45520	-20.47300
C	23.81610	4.00830	-21.83080
H	23.23530	4.49720	-22.61530
C	24.07990	4.05520	-19.27990
H	23.56910	4.26600	-18.34270

H	24.33750	2.99700	-19.25930
C	25.25780	4.56810	-21.94710
H	25.79070	4.03690	-22.73820
C	25.36180	4.88610	-19.37360
H	25.09910	5.94380	-19.34740
C	26.12800	4.56540	-20.67380
H	26.65670	3.61660	-20.57320
C	22.39140	0.73800	-23.23640
H	21.84080	0.71370	-24.17720
H	23.25430	0.08120	-23.35700
C	20.22720	2.27390	-19.06330
H	19.65190	2.99000	-18.47490
H	19.66440	2.08820	-19.97950
C	21.49100	0.26310	-22.08460
H	22.09060	0.14320	-21.18070
H	20.75720	1.03910	-21.86300
C	20.74020	-1.03960	-22.38430
H	20.09540	-0.88920	-23.25100
H	21.44560	-1.82460	-22.65600
C	19.89240	-1.48560	-21.18470
H	19.28500	-0.64670	-20.84400
H	20.55040	-1.74370	-20.35460
C	18.96520	-2.66750	-21.49490
H	18.25930	-2.37810	-22.27440
H	19.54610	-3.49720	-21.89760
C	18.19610	-3.12660	-20.24790
H	17.67260	-2.27430	-19.81410
H	18.90560	-3.46730	-19.49330
C	17.18220	-4.24180	-20.53510
H	16.42970	-3.87630	-21.23490
H	17.68390	-5.07410	-21.02910
C	16.49750	-4.73910	-19.25350
H	16.02870	-3.89660	-18.74440
H	17.25240	-5.12960	-18.57070
C	15.44160	-5.82160	-19.51540
H	14.64950	-5.41120	-20.14280
H	15.89010	-6.63860	-20.08120
C	14.83730	-6.37080	-18.21440
H	14.41060	-5.54940	-17.63800
H	15.63050	-6.79810	-17.60060
C	13.75750	-7.43390	-18.45940
H	12.93350	-6.99040	-19.01970
H	14.16410	-8.22720	-19.08730
C	13.22460	-8.04090	-17.15310
H	12.83530	-7.24640	-16.51580
H	14.04790	-8.49980	-16.60520
C	12.12710	-9.08780	-17.39030
H	11.28070	-8.61690	-17.89180
H	12.49760	-9.85590	-18.06980
C	11.65040	-9.74820	-16.08850
H	11.29140	-8.98040	-15.40230
H	12.49470	-10.23140	-15.59640
C	10.54040	-10.78220	-16.32370
H	9.67910	-10.29580	-16.78300
H	10.88260	-11.53420	-17.03480
C	10.09890	-11.47690	-15.03130
H	9.71260	-10.75650	-14.31010
H	9.31160	-12.20420	-15.23010
H	10.92840	-12.00810	-14.56480
C	20.40090	0.96300	-18.28540
H	20.99140	1.14480	-17.38650
H	20.96990	0.25620	-18.89030
C	19.05830	0.33920	-17.88330
H	18.53350	1.02150	-17.21340
H	18.42570	0.22880	-18.76470
C	19.22030	-1.02680	-17.20300
H	19.89560	-0.93550	-16.35160
H	19.69140	-1.72250	-17.89820

C	17.87930	-1.59810	-16.72460
H	17.45490	-0.93330	-15.97110
H	17.17020	-1.61340	-17.55290
C	18.00840	-3.01280	-16.14630
H	18.74830	-3.01650	-15.34550
H	18.38330	-3.68560	-16.91830
C	16.67370	-3.53810	-15.60250
H	16.34400	-2.89930	-14.78210
H	15.90780	-3.46360	-16.37510
C	16.76080	-4.99040	-15.11870
H	17.55210	-5.08300	-14.37410
H	17.04400	-5.63200	-15.95360
C	15.43730	-5.47750	-14.51510
H	15.19490	-4.87060	-13.64180
H	14.62910	-5.32220	-15.23050
C	15.47870	-6.95700	-14.11590
H	16.30740	-7.12840	-13.42800
H	15.67990	-7.56170	-15.00040
C	14.17030	-7.41750	-13.46050
H	14.00480	-6.84780	-12.54530
H	13.33140	-7.19120	-14.11950
C	14.16910	-8.91650	-13.13940
H	15.02350	-9.15570	-12.50530
H	14.30140	-9.47980	-14.06320
C	12.87710	-9.36380	-12.44290
H	12.77540	-8.83510	-11.49430
H	12.01710	-9.07710	-13.04890
C	12.83870	-10.87650	-12.19470
H	13.70870	-11.17280	-11.60760
H	12.91770	-11.39680	-13.14940
C	11.56050	-11.32130	-11.47140
H	11.50340	-10.83550	-10.49670
H	10.68590	-10.98950	-12.03150
C	11.49080	-12.83990	-11.28600
H	12.33500	-13.20490	-10.70050
H	10.57620	-13.12800	-10.76740
H	11.50190	-13.35120	-12.24830
O	25.25240	5.93240	-22.30080
O	27.03190	5.63070	-20.92450
O	26.18050	4.56980	-18.26120
C	26.50430	6.41200	-21.94830
C	27.44610	6.32890	-23.16050
H	27.03620	6.90360	-23.99140
H	28.42290	6.73580	-22.89770
H	27.57760	5.29480	-23.47780
C	26.33410	7.85200	-21.45370
H	27.28750	8.24090	-21.09550
H	25.97680	8.48380	-22.26720
H	25.60370	7.88820	-20.64630
Si	26.13800	5.34290	-16.75150
C	24.37750	5.92650	-16.34900
H	23.96600	6.54910	-17.14280
H	23.69990	5.08660	-16.20150
H	24.35560	6.51530	-15.43290
C	26.67680	4.07100	-15.46350
H	27.67560	3.69440	-15.67540
H	26.69020	4.49250	-14.45920
H	26.00110	3.21690	-15.44920
C	27.33720	6.83670	-16.74610
C	26.82800	7.90900	-17.71950
H	27.47790	8.78450	-17.72420
H	26.80180	7.51920	-18.73430
H	25.82310	8.24530	-17.46330
C	28.73040	6.36930	-17.19130
H	29.12400	5.59880	-16.52810
H	28.69630	5.95760	-18.20060
H	29.44430	7.19330	-17.19800
C	27.41540	7.42600	-15.32690

H	27.78750	6.69390	-14.60970
H	28.08660	8.28460	-15.29180
H	26.43820	7.76170	-14.97870
C	32.80250	-19.88090	-20.11420
C	31.73100	-18.89550	-22.77460
O	32.61620	-19.07350	-19.20520
O	31.61200	-19.81770	-23.58120
N	32.17540	-21.06460	-20.15980
N	30.67990	-18.22780	-22.27660
H	32.37300	-21.66630	-20.94630
H	30.86050	-17.45320	-21.64910
C	33.74620	-19.52730	-21.28630
H	34.60520	-19.03970	-20.82680
C	33.12360	-18.48520	-22.25940
H	32.99780	-17.55570	-21.70640
C	34.27520	-20.77450	-22.03430
H	34.71790	-21.47530	-21.32620
H	33.45920	-21.31100	-22.51920
C	34.05950	-18.16760	-23.44870
H	33.48960	-17.63480	-24.21270
C	35.31730	-20.39900	-23.09520
H	36.20250	-20.01190	-22.59220
C	34.77520	-19.35940	-24.10140
H	34.12630	-19.84440	-24.83160
C	29.29020	-18.56750	-22.54890
H	28.66560	-17.71470	-22.28660
H	29.15890	-18.73210	-23.61990
C	31.15030	-21.50880	-19.22770
H	31.58430	-21.63100	-18.23430
H	30.37530	-20.74490	-19.15030
C	28.85690	-19.81010	-21.75240
H	29.57120	-20.61720	-21.91740
H	28.88410	-19.59040	-20.68480
C	27.46380	-20.30980	-22.14920
H	26.72290	-19.53730	-21.94730
H	27.44210	-20.48390	-23.22580
C	27.07920	-21.60540	-21.42310
H	27.04210	-21.42620	-20.34860
H	27.85490	-22.35460	-21.58450
C	25.73590	-22.16490	-21.90540
H	24.95430	-21.42160	-21.75070
H	25.78420	-22.34030	-22.98090
C	25.35050	-23.46890	-21.19670
H	25.27610	-23.29040	-20.12430
H	26.14210	-24.20620	-21.33400
C	24.02640	-24.04100	-21.71750
H	23.23810	-23.29780	-21.59850
H	24.11070	-24.23130	-22.78810
C	23.61870	-25.33350	-21.00060
H	23.52320	-25.13900	-19.93280
H	24.40870	-26.07680	-21.11150
C	22.30010	-25.90440	-21.53720
H	21.51500	-25.15420	-21.44160
H	22.40130	-26.11030	-22.60350
C	21.87130	-27.18350	-20.80840
H	21.76590	-26.97330	-19.74450
H	22.65560	-27.93540	-20.90030
C	20.55250	-27.74960	-21.35000
H	19.77290	-26.99220	-21.26710
H	20.65980	-27.96650	-22.41350
C	20.10840	-29.01870	-20.61200
H	20.00560	-28.79960	-19.54970
H	20.88390	-29.78040	-20.69840
C	18.78220	-29.57320	-21.14820
H	18.01250	-28.80550	-21.06900
H	18.88480	-29.79820	-22.21040
C	18.32370	-30.83240	-20.40120
H	18.23920	-30.61090	-19.33770

H	19.08450	-31.60780	-20.49590
C	16.98030	-31.36450	-20.91840
H	16.21850	-30.58940	-20.82540
H	17.06210	-31.59080	-21.98220
C	16.51640	-32.61880	-20.17080
H	16.37010	-32.41410	-19.11030
H	15.57000	-32.98240	-20.57130
H	17.24650	-33.42330	-20.26130
C	30.54430	-22.82970	-19.71990
H	31.32100	-23.59540	-19.74260
H	30.20390	-22.70940	-20.74920
C	29.37000	-23.31990	-18.86190
H	29.69200	-23.43660	-17.82630
H	28.58390	-22.56490	-18.85910
C	28.80310	-24.64900	-19.38130
H	29.58530	-25.40880	-19.35360
H	28.52290	-24.53710	-20.42950
C	27.58670	-25.14220	-18.58640
H	27.85250	-25.24600	-17.53360
H	26.79590	-24.39410	-18.63360
C	27.05760	-26.48140	-19.11930
H	27.84730	-27.23120	-19.05790
H	26.81370	-26.37890	-20.17740
C	25.82080	-26.98270	-18.36190
H	26.05510	-27.07980	-17.30110
H	25.02680	-26.24030	-18.43580
C	25.31510	-28.32780	-18.90260
H	26.10900	-29.07140	-18.82160
H	25.09230	-28.23060	-19.96580
C	24.06710	-28.83360	-18.16690
H	24.28400	-28.92870	-17.10220
H	23.27260	-28.09320	-18.25430
C	23.57190	-30.18040	-18.71280
H	24.36690	-30.92180	-18.62360
H	23.35870	-30.08420	-19.77810
C	22.31840	-30.68760	-17.98720
H	22.52820	-30.78370	-16.92120
H	21.52490	-29.94670	-18.07890
C	21.82430	-32.03330	-18.53660
H	22.61930	-32.77480	-18.44860
H	21.61110	-31.93480	-19.60170
C	20.57100	-32.54130	-17.81090
H	20.78270	-32.64120	-16.74560
H	19.77860	-31.79870	-17.89850
C	20.07250	-33.88410	-18.36320
H	20.86740	-34.62670	-18.28340
H	19.85300	-33.78040	-19.42660
C	18.82300	-34.39440	-17.63190
H	19.03920	-34.50410	-16.56860
H	18.02620	-33.65490	-17.70870
C	18.32140	-35.73200	-18.18660
H	19.07950	-36.50930	-18.08690
H	17.43160	-36.06480	-17.65170
H	18.06250	-35.64940	-19.24240
O	35.14670	-17.34580	-23.09440
O	35.87150	-18.72420	-24.74040
O	35.62360	-21.58500	-23.80620
C	36.05600	-17.48620	-24.13130
C	35.82160	-16.38080	-25.17450
H	35.91580	-15.40150	-24.70420
H	36.56080	-16.46250	-25.97160
H	34.82910	-16.46530	-25.61580
C	37.46650	-17.41660	-23.54440
H	38.20730	-17.59620	-24.32360
H	37.58440	-18.16090	-22.75990
H	37.63480	-16.42870	-23.11670
Si	37.17260	-22.20040	-24.10830
C	37.04210	-23.29900	-25.64590

H	36.34440	-24.12120	-25.49050
H	36.68770	-22.73200	-26.50610
H	38.00500	-23.73160	-25.91480
C	38.39750	-20.79290	-24.43480
H	38.53160	-20.16500	-23.55630
H	39.37930	-21.17670	-24.70920
H	38.06030	-20.15240	-25.24890
C	37.73350	-23.24670	-22.60490
C	36.65540	-24.30010	-22.29780
H	36.93150	-24.91270	-21.43920
H	35.69640	-23.83170	-22.07300
H	36.50120	-24.97160	-23.14290
C	37.93090	-22.33900	-21.37970
H	38.67930	-21.56860	-21.56630
H	37.00470	-21.83900	-21.10280
H	38.26140	-22.91040	-20.51190
C	39.06290	-23.94470	-22.94070
H	39.84310	-23.22130	-23.18020
H	39.41860	-24.54400	-22.10200
H	38.95950	-24.61240	-23.79640
C	31.85610	-15.14600	-17.47430
C	31.40840	-14.55670	-20.38640
O	31.32940	-14.25650	-16.80610
O	31.51820	-15.58240	-21.05660
N	31.40250	-16.41010	-17.47580
N	30.24150	-13.91460	-20.22740
H	31.88140	-17.08800	-18.05460
H	30.24950	-13.05520	-19.69130
C	33.04730	-14.80210	-18.39600
H	33.69240	-14.16260	-17.79410
C	32.62420	-13.96870	-19.64080
H	32.30500	-12.99420	-19.27290
C	33.88560	-16.03520	-18.79000
H	34.12590	-16.61950	-17.90370
H	33.31300	-16.68420	-19.44840
C	33.80460	-13.70680	-20.61390
H	33.40170	-13.44260	-21.59360
C	35.17090	-15.61950	-19.51240
H	35.76640	-15.00470	-18.84150
C	34.84180	-14.82950	-20.79570
H	34.50990	-15.50780	-21.57860
C	28.95270	-14.37160	-20.72910
H	28.26490	-13.52810	-20.69820
H	29.04530	-14.66460	-21.77630
C	30.22510	-16.86770	-16.75100
H	30.47300	-16.95900	-15.69270
H	29.42980	-16.12430	-16.82970
C	28.39650	-15.53350	-19.88730
H	29.08990	-16.37390	-19.93220
H	28.33940	-15.23420	-18.84000
C	27.01240	-15.99760	-20.36070
H	26.29750	-15.18670	-20.23190
H	27.04630	-16.20800	-21.42990
C	26.51510	-17.24360	-19.61400
H	26.44440	-17.02690	-18.54810
H	27.24960	-18.04180	-19.71940
C	25.15480	-17.73460	-20.12910
H	24.40960	-16.95670	-19.97390
H	25.20870	-17.89270	-21.20640
C	24.69110	-19.02940	-19.44710
H	24.59760	-18.86060	-18.37530
H	25.45380	-19.79750	-19.57150
C	23.35510	-19.54520	-20.00070
H	22.59100	-18.78110	-19.86730
H	23.44290	-19.70430	-21.07580
C	22.89770	-20.84550	-19.32490
H	22.78420	-20.67390	-18.25580
H	23.67260	-21.60420	-19.43280

C	21.57580	-21.38180	-19.89170
H	20.80440	-20.61870	-19.79360
H	21.68620	-21.57050	-20.95990
C	21.11390	-22.66420	-19.18520
H	20.98350	-22.46090	-18.12350
H	21.89340	-23.42270	-19.25980
C	19.80140	-23.22210	-19.75150
H	19.02550	-22.45970	-19.68560
H	19.92590	-23.44290	-20.81200
C	19.33780	-24.48470	-19.01140
H	19.19630	-24.25080	-17.95700
H	20.12010	-25.24280	-19.05750
C	18.03230	-25.06070	-19.57480
H	17.25170	-24.30160	-19.52660
H	18.16450	-25.29860	-20.63070
C	17.57150	-26.31420	-18.81800
H	17.43020	-26.06950	-17.76560
H	18.35560	-27.07120	-18.85620
C	16.26770	-26.89750	-19.37840
H	15.47750	-26.14760	-19.32450
H	16.39600	-27.13310	-20.43520
C	15.82030	-28.15800	-18.63030
H	15.60370	-27.93850	-17.58510
H	14.91690	-28.57500	-19.07560
H	16.59040	-28.92880	-18.65790
C	29.73310	-18.21370	-17.30440
H	30.51720	-18.96330	-17.18840
H	29.55140	-18.12550	-18.37610
C	28.45190	-18.69800	-16.60870
H	28.63900	-18.80080	-15.53910
H	27.67500	-17.93960	-16.71070
C	27.93830	-20.03380	-17.16700
H	28.70990	-20.79480	-17.04490
H	27.76530	-19.94050	-18.23980
C	26.64640	-20.50100	-16.47900
H	26.82010	-20.59020	-15.40590
H	25.87360	-19.74160	-16.60040
C	26.13840	-21.84400	-17.02410
H	26.91050	-22.60120	-16.88400
H	25.97530	-21.76480	-18.09930
C	24.84180	-22.30950	-16.34480
H	24.99830	-22.37060	-15.26710
H	24.06260	-21.56330	-16.49850
C	24.36150	-23.67190	-16.86660
H	25.14070	-24.41510	-16.69430
H	24.21710	-23.61950	-17.94600
C	23.05910	-24.14080	-16.20250
H	23.19160	-24.16740	-15.12020
H	22.27160	-23.41360	-16.39710
C	22.61310	-25.52520	-16.69550
H	23.40020	-26.25060	-16.48710
H	22.48930	-25.50430	-17.77840
C	21.30520	-25.99610	-16.04500
H	21.41590	-25.99060	-14.95990
H	20.51190	-25.28660	-16.27660
C	20.88620	-27.39880	-16.50790
H	21.67900	-28.10860	-16.26940
H	20.77750	-27.40690	-17.59260
C	19.57470	-27.86730	-15.86350
H	19.67200	-27.83660	-14.77760
H	18.77850	-27.17030	-16.12160
C	19.17070	-29.28230	-16.29990
H	19.96760	-29.98110	-16.04280
H	19.06890	-29.31200	-17.38480
C	17.85870	-29.74730	-15.65370
H	17.94980	-29.70660	-14.56770
H	17.05610	-29.06120	-15.92240
C	17.46240	-31.16600	-16.07290

H	18.22550	-31.88940	-15.78480
H	16.52650	-31.46440	-15.60040
H	17.32440	-31.23250	-17.15180
O	34.61550	-12.63000	-20.20840
O	36.00720	-14.11930	-21.19120
O	35.89350	-16.78750	-19.87570
C	35.80220	-12.77320	-20.90730
C	35.72210	-11.99490	-22.23180
H	35.50910	-10.94430	-22.03230
H	36.67290	-12.06830	-22.76020
H	34.94060	-12.39770	-22.87510
C	36.93770	-12.25850	-20.01900
H	37.90160	-12.44440	-20.49300
H	36.82130	-11.18720	-19.85170
H	36.90930	-12.75560	-19.05360
Si	37.06310	-17.58970	-18.94000
C	36.49170	-17.71970	-17.13760
H	36.31500	-16.73920	-16.70000
H	35.56810	-18.29130	-17.05230
H	37.23750	-18.21910	-16.52050
C	37.24700	-19.34020	-19.63900
H	37.53490	-19.32000	-20.68830
H	38.00600	-19.90940	-19.10420
H	36.31750	-19.90140	-19.56060
C	38.75750	-16.69210	-19.03860
C	38.70000	-15.35880	-18.27980
H	39.66720	-14.85550	-18.28650
H	37.98580	-14.68240	-18.74020
H	38.41080	-15.49900	-17.23820
C	39.10450	-16.42500	-20.51060
H	39.16750	-17.35280	-21.07920
H	38.34840	-15.79520	-20.98080
H	40.06290	-15.91450	-20.60830
C	39.84140	-17.58190	-18.40440
H	39.95030	-18.52560	-18.93910
H	40.81480	-17.09060	-18.41790
H	39.60780	-17.81580	-17.36530

cis-2 octamer

C	15.79770	11.25710	-24.60620
C	18.16070	9.73330	-25.78220
O	14.96960	11.34890	-25.51110
O	18.98610	9.23160	-25.02230
N	15.54760	10.60350	-23.46100
N	17.29680	9.00200	-26.50040
H	16.29250	10.52830	-22.78050
H	16.65470	9.49950	-27.10130
C	17.18490	11.90650	-24.78740
H	16.95890	12.92540	-25.10610
C	18.03390	11.26330	-25.92140
H	17.51100	11.43710	-26.86380
C	17.98420	12.02130	-23.47540
H	17.34680	12.40460	-22.68250
H	18.33300	11.04360	-23.14460
C	19.42000	11.94820	-26.05850
H	20.09030	11.30070	-26.62730
C	19.18650	12.94650	-23.67510
H	18.82770	13.93290	-23.96920
C	20.12950	12.38230	-24.75890
H	20.72080	11.56130	-24.35160
C	17.06380	7.57420	-26.33760
H	16.55350	7.21720	-27.23250
H	18.00950	7.03350	-26.27640
C	14.26670	10.01080	-23.10670
H	13.57890	10.80860	-22.82340

H	13.83190	9.50590	-23.97100
C	16.19680	7.31210	-25.09490
H	16.76550	7.54550	-24.19300
H	15.34510	7.99300	-25.10450
C	15.66900	5.87560	-25.01680
H	15.07110	5.66860	-25.90520
H	16.50010	5.17140	-25.03430
C	14.81530	5.64400	-23.76240
H	14.05510	6.42260	-23.69430
H	15.43670	5.74230	-22.87200
C	14.12130	4.27770	-23.76430
H	13.45660	4.21720	-24.62700
H	14.86200	3.48860	-23.89110
C	13.31780	4.02850	-22.48120
H	12.64830	4.87010	-22.30140
H	13.99640	3.98450	-21.62900
C	12.48990	2.74060	-22.55270
H	11.76880	2.82370	-23.36680
H	13.13970	1.90210	-22.80260
C	11.75050	2.44250	-21.24230
H	11.15930	3.31090	-20.95070
H	12.47660	2.27920	-20.44570
C	10.82840	1.22330	-21.35900
H	10.06300	1.42210	-22.11020
H	11.39980	0.36910	-21.72160
C	10.15850	0.86080	-20.02760
H	9.62770	1.72940	-19.63740
H	10.92480	0.60960	-19.29420
C	9.17770	-0.30960	-20.16860
H	8.37740	-0.02920	-20.85460
H	9.68900	-1.15760	-20.62380
C	8.57510	-0.73980	-18.82500
H	8.09070	0.11470	-18.35220
H	9.37540	-1.05290	-18.15430
C	7.55910	-1.87890	-18.97800
H	6.73180	-1.54420	-19.60510
H	8.02620	-2.71100	-19.50450
C	7.01530	-2.37030	-17.62990
H	6.56120	-1.53620	-17.09460
H	7.84310	-2.72020	-17.01270
C	5.98460	-3.49590	-17.78830
H	5.13460	-3.13560	-18.36860
H	6.42180	-4.31310	-18.36140
C	5.48800	-4.03310	-16.44220
H	4.99660	-3.25070	-15.86430
H	4.77060	-4.84110	-16.58650
H	6.31170	-4.42440	-15.84540
C	14.43330	9.01570	-21.95120
H	14.90090	9.51250	-21.09990
H	15.11610	8.22230	-22.25590
C	13.09480	8.40790	-21.51120
H	12.44490	9.20120	-21.13960
H	12.58920	7.97640	-22.37600
C	13.25770	7.32850	-20.43300
H	13.80290	7.73630	-19.58090
H	13.86860	6.51800	-20.83040
C	11.90710	6.77410	-19.95750
H	11.33360	7.57460	-19.48850
H	11.32570	6.44350	-20.81920
C	12.05740	5.60460	-18.97590
H	12.67290	5.91040	-18.12920
H	12.59270	4.79390	-19.47000
C	10.70430	5.09140	-18.46220
H	10.20960	5.88150	-17.89590
H	10.05420	4.86620	-19.30860
C	10.84280	3.83810	-17.58750
H	11.52610	4.03970	-16.76190
H	11.29810	3.04290	-18.17780

C	9.49760	3.35520	-17.02600
H	9.08390	4.12150	-16.36920
H	8.78420	3.23070	-17.84150
C	9.62400	2.03110	-16.25990
H	10.36800	2.13460	-15.46930
H	9.99870	1.26350	-16.93710
C	8.29480	1.56860	-15.64620
H	7.95980	2.30360	-14.91320
H	7.52690	1.53340	-16.41990
C	8.40680	0.18970	-14.98080
H	9.19920	0.20830	-14.23180
H	8.70690	-0.54430	-15.72880
C	7.09640	-0.26020	-14.31980
H	6.82940	0.44350	-13.53040
H	6.28730	-0.22440	-15.05020
C	7.19160	-1.67700	-13.73610
H	8.01600	-1.72390	-13.02370
H	7.43380	-2.37890	-14.53440
C	5.89590	-2.12000	-13.04290
H	5.67250	-1.44220	-12.21820
H	5.06050	-2.04170	-13.73920
C	5.97590	-3.55510	-12.51120
H	6.78280	-3.66330	-11.78590
H	5.04590	-3.84030	-12.01920
H	6.15420	-4.26450	-13.31920
O	19.32760	13.16640	-26.76220
O	20.96280	13.43810	-25.21050
O	19.88650	13.04700	-22.44730
C	20.48090	13.86780	-26.44330
C	21.55580	13.59750	-27.50820
H	21.18920	13.89270	-28.49160
H	22.45470	14.17070	-27.27970
H	21.81810	12.54010	-27.53240
C	20.12180	15.35510	-26.35710
H	20.99080	15.93250	-26.04060
H	19.79350	15.71460	-27.33250
H	19.31160	15.50690	-25.64510
Si	19.54480	14.14800	-21.20170
C	17.69920	14.59300	-21.17480
H	17.34960	14.93120	-22.14960
H	17.08440	13.74500	-20.87610
H	17.49550	15.39320	-20.46430
C	19.99430	13.29310	-19.57920
H	21.04250	13.00480	-19.56570
H	19.81660	13.93780	-18.71950
H	19.40620	12.38710	-19.43770
C	20.58470	15.74040	-21.43190
C	20.14870	16.45410	-22.72010
H	20.70680	17.37830	-22.87210
H	20.32650	15.82110	-23.58720
H	19.08860	16.70800	-22.70330
C	22.07060	15.36910	-21.53770
H	22.41600	14.83780	-20.65060
H	22.24610	14.72990	-22.40270
H	22.69550	16.25480	-21.65480
C	20.36890	16.67200	-20.22640
H	20.67380	16.19610	-19.29410
H	20.94900	17.58990	-20.32740
H	19.32220	16.95880	-20.12360
C	18.59530	8.67440	-20.71860
C	20.65920	7.19340	-22.37590
O	17.71490	9.27310	-21.33600
O	21.32100	6.37780	-21.74080
N	18.34360	7.58860	-19.97100
N	19.78190	6.82210	-23.31970
H	19.12630	7.12380	-19.52810
H	19.29750	7.55310	-23.82710
C	20.05600	9.16540	-20.80120

H	19.97720	10.25240	-20.86570
C	20.79890	8.70240	-22.09040
H	20.33520	9.21880	-22.92840
C	20.86160	8.85750	-19.52020
H	20.29390	9.15250	-18.63990
H	21.04230	7.78800	-19.42140
C	22.29270	9.12460	-22.09690
H	22.83470	8.50490	-22.81410
C	22.20590	9.58880	-19.55640
H	22.01890	10.65850	-19.65100
C	23.04250	9.09180	-20.75210
H	23.42500	8.09160	-20.55020
C	19.43170	5.43930	-23.62890
H	18.88380	5.43230	-24.57050
H	20.33740	4.85180	-23.78870
C	17.02690	7.00350	-19.76770
H	16.40990	7.70330	-19.20230
H	16.53880	6.85110	-20.73130
C	18.57500	4.81800	-22.51140
H	19.14950	4.78420	-21.58460
H	17.72300	5.46890	-22.31790
C	18.06690	3.40510	-22.83130
H	17.48470	3.42900	-23.75240
H	18.91050	2.73930	-23.01470
C	17.20290	2.84290	-21.69310
H	16.42690	3.56730	-21.44490
H	17.81400	2.72950	-20.79750
C	16.53810	1.50240	-22.03430
H	15.92270	1.61970	-22.92620
H	17.29980	0.76340	-22.28190
C	15.66860	0.98240	-20.88110
H	14.97320	1.76370	-20.57330
H	16.30060	0.77440	-20.01780
C	14.87200	-0.27590	-21.24940
H	14.21790	-0.05740	-22.09340
H	15.55380	-1.05620	-21.58610
C	14.03330	-0.79670	-20.07480
H	13.40800	0.00930	-19.69040
H	14.69620	-1.08890	-19.26020
C	13.14110	-1.98290	-20.46200
H	12.44980	-1.67710	-21.24720
H	13.75420	-2.77600	-20.88880
C	12.34910	-2.53260	-19.26870
H	11.78010	-1.72520	-18.80790
H	13.04430	-2.89310	-18.51010
C	11.39080	-3.66260	-19.66640
H	10.66870	-3.28910	-20.39250
H	11.94810	-4.45140	-20.17060
C	10.64650	-4.25190	-18.46130
H	10.12240	-3.45520	-17.93380
H	11.36950	-4.66450	-17.75760
C	9.64230	-5.34000	-18.86350
H	8.89770	-4.91710	-19.53800
H	10.15580	-6.11790	-19.42780
C	8.93850	-5.96670	-17.65290
H	8.44730	-5.18470	-17.07460
H	9.68240	-6.41450	-16.99410
C	7.90620	-7.02810	-18.05660
H	7.15040	-6.58160	-18.70300
H	8.39120	-7.80320	-18.64900
C	7.21970	-7.67070	-16.84750
H	6.67010	-6.92920	-16.26820
H	6.51200	-8.43710	-17.16360
H	7.94640	-8.14270	-16.18670
C	17.14380	5.66800	-19.01910
H	17.66290	5.82340	-18.07230
H	17.76300	4.98200	-19.59690
C	15.78010	5.01850	-18.75100

H	15.17590	5.69160	-18.14180
H	15.24710	4.89240	-19.69330
C	15.89750	3.65650	-18.05180
H	16.46310	3.76710	-17.12580
H	16.46900	2.97540	-18.68220
C	14.52610	3.04180	-17.74070
H	13.97770	3.70850	-17.07450
H	13.94260	2.97460	-18.65860
C	14.62250	1.64800	-17.10570
H	15.23800	1.69580	-16.20680
H	15.13110	0.97380	-17.79440
C	13.24470	1.07470	-16.74720
H	12.76490	1.72590	-16.01590
H	12.60730	1.08180	-17.63110
C	13.31680	-0.35420	-16.19280
H	13.98290	-0.37960	-15.32990
H	13.75730	-1.00920	-16.94420
C	11.93920	-0.89340	-15.78510
H	11.53060	-0.26880	-14.99020
H	11.25170	-0.80970	-16.62650
C	11.98620	-2.35400	-15.31770
H	12.69710	-2.45330	-14.49680
H	12.36020	-2.97960	-16.12830
C	10.61340	-2.86770	-14.86420
H	10.27050	-2.27500	-14.01560
H	9.88540	-2.71410	-15.66060
C	10.63360	-4.35250	-14.47880
H	11.37770	-4.51760	-13.69890
H	10.95010	-4.94440	-15.33790
C	9.26650	-4.84840	-13.98970
H	8.97540	-4.28600	-13.10180
H	8.51120	-4.63890	-14.74690
C	9.26200	-6.34910	-13.67070
H	10.02430	-6.56390	-12.92110
H	9.54030	-6.90980	-14.56360
C	7.89800	-6.83710	-13.16450
H	7.63090	-6.29800	-12.25490
H	7.12740	-6.60070	-13.89860
C	7.88050	-8.34270	-12.88290
H	8.61230	-8.60850	-12.11990
H	6.90000	-8.66130	-12.52880
H	8.11020	-8.91380	-13.78240
O	22.47880	10.46310	-22.49620
O	24.10080	10.00900	-20.97350
O	22.91050	9.32260	-18.35310
C	23.75900	10.79120	-22.07130
C	24.76540	10.51840	-23.20190
H	24.49120	11.08960	-24.08930
H	25.76540	10.81580	-22.88550
H	24.78710	9.45940	-23.45690
C	23.76020	12.26140	-21.65550
H	24.73140	12.53610	-21.24390
H	22.99000	12.43850	-20.90730
H	23.55520	12.88320	-22.52610
Si	23.33020	10.47750	-17.17950
C	24.79590	9.80470	-16.19440
H	24.61250	8.78100	-15.87740
H	25.71020	9.81470	-16.78460
H	24.99670	10.39690	-15.30320
C	23.86240	12.09620	-18.00670
H	23.03740	12.58030	-18.52340
H	24.24510	12.80760	-17.27600
H	24.65110	11.92290	-18.73820
C	21.85250	10.75950	-15.98890
C	21.30070	9.39350	-15.54560
H	20.45020	9.50700	-14.87300
H	20.96940	8.79880	-16.39680
H	22.05870	8.81240	-15.01970

C	20.75550	11.57230	-16.69480
H	21.11380	12.56600	-16.96410
H	20.41570	11.09000	-17.60780
H	19.88430	11.70550	-16.05290
C	22.32780	11.54560	-14.75370
H	22.78830	12.49280	-15.03660
H	21.49610	11.77220	-14.08590
H	23.05880	10.98270	-14.17330
C	28.54150	-9.75530	-15.56450
C	29.59030	-9.56060	-18.38770
O	27.80300	-8.82170	-15.25490
O	29.95830	-10.67070	-18.76790
N	28.08910	-11.01400	-15.69670
N	28.53100	-8.93060	-18.91570
H	28.75230	-11.72100	-15.98300
H	28.30110	-8.01130	-18.55490
C	30.04340	-9.49180	-15.83560
H	30.34730	-8.75610	-15.08970
C	30.31780	-8.84900	-17.23040
H	29.92720	-7.83540	-17.19250
C	30.90410	-10.74980	-15.60110
H	30.70280	-11.16620	-14.61420
H	30.63650	-11.52300	-16.31690
C	31.82750	-8.70890	-17.55260
H	31.94570	-8.56180	-18.62810
C	32.39930	-10.45280	-15.73580
H	32.70270	-9.77570	-14.93840
C	32.74320	-9.85710	-17.11260
H	32.75240	-10.64290	-17.85880
C	27.60310	-9.53050	-19.86510
H	27.03330	-8.73410	-20.34380
H	28.15380	-10.04530	-20.65450
C	26.70810	-11.43760	-15.49730
H	26.42300	-11.25030	-14.46100
H	26.04830	-10.83460	-16.12250
C	26.65870	-10.49990	-19.13870
H	27.24860	-11.25680	-18.61990
H	26.11330	-9.95480	-18.36710
C	25.66140	-11.21010	-20.06010
H	25.01450	-10.47510	-20.53900
H	26.19710	-11.72420	-20.85870
C	24.82200	-12.22170	-19.27010
H	24.33770	-11.71090	-18.43690
H	25.48600	-12.96700	-18.83070
C	23.75650	-12.93820	-20.10640
H	23.06070	-12.20680	-20.51660
H	24.22310	-13.43310	-20.95830
C	22.99800	-13.96880	-19.26020
H	22.58820	-13.47600	-18.37760
H	23.70130	-14.71830	-18.89560
C	21.86280	-14.67060	-20.01420
H	21.13720	-13.93090	-20.35020
H	22.25330	-15.14650	-20.91350
C	21.17030	-15.71850	-19.13300
H	20.82050	-15.24290	-18.21610
H	21.89920	-16.47100	-18.83070
C	19.98920	-16.40950	-19.82500
H	19.24490	-15.66430	-20.10310
H	20.32250	-16.86840	-20.75540
C	19.34630	-17.47430	-18.92680
H	19.04160	-17.01530	-17.98600
H	20.09050	-18.22990	-18.67400
C	18.13400	-18.15420	-19.57510
H	17.38060	-17.40350	-19.81100
H	18.42660	-18.60050	-20.52520
C	17.52290	-19.23070	-18.66880
H	17.25470	-18.78490	-17.71100
H	18.27310	-19.99230	-18.45550

C	16.28480	-19.89490	-19.28440
H	15.52830	-19.13660	-19.48390
H	16.54380	-20.32810	-20.25030
C	15.69520	-20.98240	-18.37680
H	15.45870	-20.55160	-17.40380
H	16.44730	-21.75140	-18.19960
C	14.43530	-21.62870	-18.96750
H	13.66940	-20.86860	-19.12200
H	14.66050	-22.04000	-19.95130
C	13.87140	-22.73740	-18.07360
H	13.59550	-22.34890	-17.09340
H	12.98120	-23.18170	-18.51890
H	14.60080	-23.53380	-17.92690
C	26.54000	-12.93010	-15.83520
H	27.20980	-13.52000	-15.20790
H	26.85310	-13.10770	-16.86470
C	25.09640	-13.42870	-15.64400
H	24.79190	-13.25100	-14.61180
H	24.42220	-12.83830	-16.26510
C	24.91900	-14.92210	-15.97690
H	25.61070	-15.51180	-15.37390
H	25.19360	-15.09980	-17.01740
C	23.48210	-15.41530	-15.72820
H	23.21710	-15.22620	-14.68720
H	22.78690	-14.82820	-16.32890
C	23.28070	-16.91030	-16.03620
H	23.99460	-17.49970	-15.45940
H	23.50450	-17.10060	-17.08610
C	21.85170	-17.38450	-15.71570
H	21.63850	-17.18620	-14.66460
H	21.13650	-16.79220	-16.28680
C	21.61520	-18.87710	-16.00470
H	22.34420	-19.47500	-15.45630
H	21.78950	-19.07500	-17.06190
C	20.19380	-19.32730	-15.62330
H	20.03040	-19.13430	-14.56240
H	19.46490	-18.71920	-16.15920
C	19.92030	-20.81170	-15.91530
H	20.66120	-21.42680	-15.40320
H	20.04710	-21.00030	-16.98090
C	18.50930	-21.24540	-15.48340
H	18.39010	-21.06630	-14.41420
H	17.76870	-20.62150	-15.98330
C	18.20830	-22.72090	-15.78780
H	18.96120	-23.35120	-15.31340
H	18.29230	-22.89200	-16.86060
C	16.81150	-23.15050	-15.31230
H	16.72940	-22.98380	-14.23770
H	16.05780	-22.51840	-15.78130
C	16.49710	-24.62050	-15.62490
H	17.26080	-25.25820	-15.17850
H	16.54980	-24.77750	-16.70190
C	15.11360	-25.05200	-15.11730
H	15.05260	-24.88940	-14.04080
H	14.34580	-24.42510	-15.56970
C	14.79550	-26.51970	-15.42080
H	15.52600	-27.18580	-14.96150
H	13.81230	-26.79030	-15.03540
H	14.79220	-26.70980	-16.49400
O	32.43930	-7.60760	-16.92390
O	34.01370	-9.23350	-17.04400
O	33.06320	-11.69640	-15.63750
C	33.80170	-7.86160	-16.99680
C	34.38210	-7.22720	-18.27250
H	34.17370	-6.15700	-18.28240
H	35.46150	-7.37650	-18.30100
H	33.95080	-7.68020	-19.16460
C	34.45860	-7.30340	-15.73710

H	35.52300	-7.53830	-15.73070
H	33.99150	-7.73680	-14.85530
H	34.33230	-6.22160	-15.71120
Si	34.35530	-12.06300	-14.61270
C	35.39370	-13.35950	-15.50960
H	34.78980	-14.23090	-15.75810
H	35.79860	-12.96370	-16.43940
H	36.23450	-13.69730	-14.90500
C	35.40310	-10.52270	-14.26640
H	34.83390	-9.75250	-13.74920
H	36.26450	-10.76050	-13.64350
H	35.78120	-10.08610	-15.19020
C	33.64570	-12.80760	-13.00000
C	32.66250	-13.93400	-13.36620
H	32.22340	-14.38400	-12.47550
H	31.84400	-13.56180	-13.98420
H	33.15590	-14.72910	-13.92600
C	32.91150	-11.71540	-12.20550
H	33.58400	-10.90330	-11.92790
H	32.09580	-11.28350	-12.78280
H	32.48390	-12.11350	-11.28490
C	34.79560	-13.37280	-12.14850
H	35.52380	-12.60000	-11.89990
H	34.42600	-13.78910	-11.21080
H	35.32620	-14.16960	-12.67030
C	26.37810	-4.78450	-14.93680
C	27.68130	-5.08550	-17.64980
O	25.56190	-3.86550	-14.87730
O	28.16880	-6.20800	-17.75990
N	26.01340	-6.07390	-14.85600
N	26.63770	-4.67960	-18.38730
H	26.73290	-6.78150	-14.93480
H	26.31420	-3.72680	-18.26080
C	27.87650	-4.46750	-15.14040
H	28.05680	-3.57760	-14.53600
C	28.22970	-4.08920	-16.60770
H	27.73750	-3.14150	-16.81570
C	28.80570	-5.56900	-14.59050
H	28.49290	-5.83680	-13.58450
H	28.72580	-6.47310	-15.19000
C	29.75110	-3.85580	-16.80750
H	29.97820	-3.93430	-17.87230
C	30.26850	-5.10830	-14.59410
H	30.36590	-4.23260	-13.95670
C	30.72020	-4.76590	-16.03000
H	30.89760	-5.67940	-16.59360
C	25.86920	-5.52850	-19.28850
H	25.29450	-4.88480	-19.95440
H	26.54130	-6.11470	-19.91750
C	24.64100	-6.53540	-14.70330
H	24.27220	-6.23820	-13.72060
H	24.00470	-6.04840	-15.44420
C	24.92670	-6.44700	-18.49430
H	25.51610	-7.07850	-17.82830
H	24.28860	-5.83640	-17.85400
C	24.04780	-7.34260	-19.37660
H	23.42250	-6.72120	-20.01750
H	24.67560	-7.93990	-20.03810
C	23.16740	-8.26970	-18.52780
H	22.58350	-7.66790	-17.83050
H	23.80690	-8.91080	-17.92040
C	22.21550	-9.14560	-19.35260
H	21.54910	-8.51040	-19.93530
H	22.78300	-9.73750	-20.07030
C	21.39180	-10.07760	-18.45400
H	20.87080	-9.48160	-17.70400
H	22.06690	-10.73740	-17.90850
C	20.36790	-10.92620	-19.21900

H	19.66470	-10.27240	-19.73320
H	20.86960	-11.50580	-19.99310
C	19.60740	-11.87120	-18.27960
H	19.15190	-11.28790	-17.47890
H	20.31560	-12.54910	-17.80290
C	18.51960	-12.69310	-18.98260
H	17.78580	-12.02170	-19.42630
H	18.95430	-13.25730	-19.80690
C	17.82260	-13.65460	-18.01120
H	17.42860	-13.08700	-17.16810
H	18.55830	-14.34630	-17.60050
C	16.68360	-14.45470	-18.65530
H	15.92850	-13.76870	-19.03680
H	17.05870	-15.00450	-19.51790
C	16.04120	-15.43140	-17.66150
H	15.70020	-14.87890	-16.78610
H	16.79570	-16.13450	-17.30820
C	14.86260	-16.21140	-18.25710
H	14.09570	-15.51210	-18.58780
H	15.18880	-16.74810	-19.14760
C	14.25940	-17.20200	-17.25220
H	13.96000	-16.66520	-16.35210
H	15.02480	-17.91540	-16.94540
C	13.05190	-17.96310	-17.81490
H	12.26830	-17.25770	-18.09160
H	13.33630	-18.47840	-18.73200
C	12.48640	-18.98000	-16.81830
H	12.14630	-18.48830	-15.90710
H	11.63740	-19.51340	-17.24590
H	13.23700	-19.72020	-16.54080
C	24.56270	-8.06030	-14.86390
H	25.20260	-8.53730	-14.12010
H	24.96230	-8.34630	-15.83720
C	23.13080	-8.59430	-14.71990
H	22.74500	-8.32490	-13.73590
H	22.48200	-8.10380	-15.44640
C	23.04520	-10.11680	-14.90410
H	23.71770	-10.60670	-14.19880
H	23.39800	-10.38520	-15.90030
C	21.61910	-10.64650	-14.69820
H	21.28060	-10.38030	-13.69600
H	20.93960	-10.14880	-15.39050
C	21.51070	-12.16680	-14.88340
H	22.21540	-12.66610	-14.21730
H	21.80510	-12.43420	-15.89880
C	20.09190	-12.67970	-14.59880
H	19.81150	-12.40720	-13.58050
H	19.38210	-12.17550	-15.25450
C	19.95080	-14.19790	-14.77340
H	20.68190	-14.70670	-14.14400
H	20.18600	-14.47100	-15.80250
C	18.54050	-14.68630	-14.41260
H	18.31770	-14.40670	-13.38200
H	17.80600	-14.17330	-15.03300
C	18.36550	-16.20130	-14.57520
H	19.11840	-16.72090	-13.98130
H	18.54490	-16.47840	-15.61410
C	16.96670	-16.66740	-14.14690
H	16.79620	-16.38490	-13.10730
H	16.21160	-16.14550	-14.73420
C	16.76530	-18.17940	-14.30140
H	17.53740	-18.70840	-13.74160
H	16.89560	-18.45560	-15.34750
C	15.38140	-18.63320	-13.81660
H	15.25480	-18.35180	-12.77040
H	14.60820	-18.10490	-14.37400
C	15.16520	-20.14330	-13.96740
H	15.95310	-20.67880	-13.43660

H	15.25790	-20.41460	-15.01860
C	13.79620	-20.59430	-13.44060
H	13.70120	-20.32490	-12.38820
H	13.00610	-20.06220	-13.96970
C	13.57020	-22.09970	-13.59760
H	14.33120	-22.67090	-13.06560
H	12.59700	-22.39120	-13.20220
H	13.60130	-22.38860	-14.64730
O	30.17260	-2.57610	-16.39550
O	31.89810	-3.97420	-15.95610
O	31.09040	-6.15730	-14.10040
C	31.55100	-2.66570	-16.27700
C	32.21410	-2.28910	-17.61370
H	31.90850	-1.28490	-17.90890
H	33.29880	-2.31010	-17.50570
H	31.93620	-2.98850	-18.40120
C	31.99700	-1.73110	-15.15000
H	33.07300	-1.81390	-14.99630
H	31.74860	-0.69970	-15.40250
H	31.48690	-1.99000	-14.22800
Si	31.44220	-6.50030	-12.47270
C	29.90880	-6.26070	-11.38300
H	29.51510	-5.24840	-11.45790
H	29.11080	-6.94980	-11.65730
H	30.13730	-6.44010	-10.33330
C	31.95470	-8.32000	-12.38530
H	32.88250	-8.49930	-12.92390
H	32.10950	-8.64370	-11.35680
H	31.19240	-8.96680	-12.81710
C	32.87680	-5.40140	-11.82240
C	32.42060	-3.93950	-11.72230
H	33.20810	-3.30400	-11.31640
H	32.16480	-3.54590	-12.70320
H	31.54990	-3.83460	-11.07530
C	34.07090	-5.48980	-12.78230
H	34.43630	-6.51260	-12.87210
H	33.79330	-5.13990	-13.77670
H	34.90340	-4.87440	-12.44050
C	33.29930	-5.89620	-10.42770
H	33.65370	-6.92690	-10.45880
H	34.10770	-5.28840	-10.01990
H	32.47060	-5.85360	-9.72040
C	23.60350	0.19180	-16.36700
C	25.52590	-0.72020	-18.50380
O	22.82810	1.00780	-16.86070
O	26.07350	-1.79430	-18.26170
N	23.21100	-1.00810	-15.91620
N	24.61190	-0.57840	-19.47600
H	23.93090	-1.61470	-15.54710
H	24.22640	0.34840	-19.62120
C	25.11030	0.51440	-16.26620
H	25.16550	1.52720	-15.86240
C	25.83170	0.52730	-17.64650
H	25.45710	1.38560	-18.20040
C	25.82520	-0.41820	-15.26710
H	25.28310	-0.43830	-14.32440
H	25.83110	-1.44130	-15.64040
C	27.35980	0.74520	-17.50080
H	27.84910	0.47070	-18.43760
C	27.26650	0.01060	-15.02010
H	27.27070	1.00000	-14.56420
C	28.06330	0.02030	-16.34160
H	28.30950	-0.99430	-16.63820
C	24.02840	-1.66880	-20.25050
H	23.52860	-1.23740	-21.11810
H	24.81470	-2.32360	-20.62970
C	21.86170	-1.53920	-15.91020
H	21.28920	-1.04140	-15.12670

H	21.36630	-1.33100	-16.85930
C	23.02060	-2.46160	-19.40030
H	23.54140	-2.90860	-18.55260
H	22.29140	-1.76820	-18.98090
C	22.27540	-3.56630	-20.16510
H	21.69360	-3.12080	-20.97230
H	22.99090	-4.24290	-20.63300
C	21.34960	-4.35990	-19.23040
H	20.69290	-3.66280	-18.70950
H	21.95270	-4.84500	-18.46210
C	20.48870	-5.41880	-19.93630
H	19.84970	-4.93630	-20.67540
H	21.12640	-6.11170	-20.48520
C	19.62390	-6.19560	-18.93130
H	19.03790	-5.48670	-18.34580
H	20.27470	-6.71200	-18.22530
C	18.67320	-7.21560	-19.57570
H	17.98910	-6.70230	-20.25040
H	19.24040	-7.91500	-20.18930
C	17.87190	-7.98820	-18.51670
H	17.34880	-7.27780	-17.87600
H	18.56260	-8.53200	-17.87220
C	16.85430	-8.97520	-19.10700
H	16.13120	-8.43390	-19.71560
H	17.35900	-9.66820	-19.77950
C	16.11980	-9.76230	-18.01160
H	15.65290	-9.06230	-17.31810
H	16.84570	-10.33210	-17.43160
C	15.05120	-10.71900	-18.55900
H	14.29740	-10.15070	-19.10200
H	15.50020	-11.39690	-19.28430
C	14.37850	-11.53250	-17.44400
H	13.96210	-10.85100	-16.70190
H	15.13200	-12.12740	-16.92790
C	13.26910	-12.45940	-17.95960
H	12.49430	-11.86500	-18.44190
H	13.66900	-13.11770	-18.73020
C	12.64630	-13.30300	-16.83850
H	12.27070	-12.64370	-16.05610
H	13.41950	-13.91870	-16.37850
C	11.50730	-14.20480	-17.33360
H	10.71150	-13.59350	-17.75870
H	11.86570	-14.84170	-18.14190
C	10.92770	-15.08230	-16.21950
H	10.51440	-14.47350	-15.41600
H	10.12820	-15.71750	-16.60080
H	11.69060	-15.73220	-15.79090
C	21.92820	-3.05120	-15.65560
H	22.48170	-3.24140	-14.73440
H	22.49700	-3.53000	-16.45320
C	20.54630	-3.70150	-15.55050
H	20.00300	-3.25030	-14.71950
H	19.96840	-3.48700	-16.44970
C	20.63210	-5.22020	-15.34510
H	21.25380	-5.43810	-14.47550
H	21.13030	-5.67830	-16.20000
C	19.24810	-5.84870	-15.14930
H	18.77760	-5.40410	-14.27140
H	18.60940	-5.59930	-15.99690
C	19.29740	-7.37260	-14.98270
H	19.96860	-7.63220	-14.16300
H	19.71760	-7.82640	-15.88080
C	17.90420	-7.95040	-14.70470
H	17.51210	-7.50770	-13.78810
H	17.22060	-7.65550	-15.50120
C	17.89570	-9.47780	-14.57370
H	18.60600	-9.78540	-13.80520
H	18.23670	-9.92550	-15.50740

C	16.49860	-10.00370	-14.21930
H	16.18300	-9.56800	-13.27040
H	15.77980	-9.66350	-14.96500
C	16.43630	-11.53210	-14.12190
H	17.17870	-11.88460	-13.40490
H	16.70420	-11.96710	-15.08470
C	15.04450	-12.02020	-13.69860
H	14.79570	-11.59540	-12.72540
H	14.29700	-11.64420	-14.39730
C	14.94360	-13.54800	-13.62830
H	15.71300	-13.93480	-12.95920
H	15.14900	-13.96750	-14.61290
C	13.56410	-14.01510	-13.14490
H	13.37020	-13.60230	-12.15420
H	12.79240	-13.61440	-13.80220
C	13.43980	-15.54220	-13.09560
H	14.23010	-15.95270	-12.46600
H	13.59820	-15.94590	-14.09530
C	12.07450	-16.00200	-12.56680
H	11.91990	-15.60970	-11.56100
H	11.28080	-15.58520	-13.18650
C	11.93650	-17.52620	-12.54150
H	12.70370	-17.98230	-11.91550
H	10.96440	-17.82330	-12.14780
H	12.02840	-17.94030	-13.54480
O	27.69820	2.08630	-17.23600
O	29.23460	0.79910	-16.16930
O	27.81780	-0.95200	-14.13800
C	28.99430	2.04360	-16.74200
C	29.98970	2.25720	-17.89360
H	29.79420	3.21310	-18.38030
H	31.00870	2.25910	-17.50590
H	29.90370	1.46100	-18.63250
C	29.12860	3.11840	-15.66550
H	30.12600	3.08950	-15.22650
H	28.39120	2.95340	-14.88250
H	28.95810	4.10140	-16.10510
Si	28.34640	-0.66540	-12.55350
C	29.24630	-2.22330	-11.97760
H	28.59220	-3.09340	-12.00960
H	30.09820	-2.43440	-12.61250
H	29.61980	-2.12560	-10.95940
C	29.52830	0.81500	-12.56410
H	29.01490	1.73580	-12.83670
H	29.98310	0.97200	-11.58690
H	30.33430	0.66970	-13.28320
C	26.87340	-0.37270	-11.35670
C	25.88490	-1.54770	-11.46980
H	25.01600	-1.39920	-10.82810
H	25.52250	-1.67870	-12.48820
H	26.35010	-2.48850	-11.17460
C	26.17000	0.95380	-11.68230
H	26.85460	1.79570	-11.57900
H	25.77640	0.97200	-12.69510
H	25.33190	1.13390	-11.00870
C	27.40630	-0.29850	-9.91400
H	28.14230	0.49830	-9.80110
H	26.60150	-0.10290	-9.20460
H	27.88200	-1.23110	-9.61070
C	21.31640	4.55330	-18.45480
C	23.27170	3.23890	-20.37200
O	20.46670	5.29810	-18.94140
O	23.87310	2.27020	-19.91330
N	20.99810	3.37970	-17.88540
N	22.35100	3.13200	-21.34180
H	21.74740	2.80220	-17.52450
H	21.90430	3.98460	-21.65480
C	22.80560	4.96590	-18.48760

H	22.78710	6.05350	-18.39260
C	23.53330	4.65470	-19.82950
H	23.12720	5.32890	-20.57630
C	23.59590	4.43320	-17.27810
H	23.02960	4.59800	-16.36460
H	23.74480	3.35740	-17.35750
C	25.04860	4.98380	-19.78860
H	25.56970	4.42300	-20.56750
C	24.96340	5.12100	-17.20040
H	24.81880	6.19970	-17.13340
C	25.79260	4.77990	-18.45560
H	26.16770	3.76000	-18.38900
C	21.87090	1.87810	-21.90860
H	21.34390	2.10680	-22.83510
H	22.71420	1.23840	-22.17420
C	19.64210	2.86200	-17.75660
H	19.08700	3.48880	-17.05740
H	19.13100	2.92550	-18.71790
C	20.93120	1.15790	-20.92870
H	21.48360	0.88890	-20.02720
H	20.14780	1.84810	-20.61510
C	20.28160	-0.10250	-21.51380
H	19.70100	0.16740	-22.39650
H	21.05290	-0.79720	-21.84810
C	19.37400	-0.79270	-20.48660
H	18.67070	-0.06250	-20.08570
H	19.97790	-1.13060	-19.64370
C	18.58500	-1.97800	-21.05880
H	17.95970	-1.63400	-21.88310
H	19.27200	-2.71320	-21.47800
C	17.70770	-2.63850	-19.98660
H	17.07640	-1.87860	-19.52570
H	18.34460	-3.02950	-19.19270
C	16.81650	-3.76550	-20.52660
H	16.15300	-3.37090	-21.29610
H	17.43200	-4.52260	-21.01170
C	15.98450	-4.40930	-19.40880
H	15.41350	-3.63350	-18.89870
H	16.65540	-4.83630	-18.66300
C	15.02050	-5.49630	-19.90390
H	14.31890	-5.06690	-20.61880
H	15.57590	-6.26230	-20.44440
C	14.24760	-6.13720	-18.74250
H	13.73000	-5.35680	-18.18480
H	14.95460	-6.59260	-18.04890
C	13.22960	-7.19460	-19.19050
H	12.49230	-6.73720	-19.84960
H	13.73170	-7.96020	-19.78120
C	12.52040	-7.84590	-17.99430
H	12.04980	-7.07040	-17.38980
H	13.26090	-8.32670	-17.35500
C	11.46270	-8.87920	-18.40410
H	10.69840	-8.39710	-19.01250
H	11.92000	-9.63830	-19.03830
C	10.80780	-9.55050	-17.18810
H	10.37410	-8.78690	-16.54190
H	11.57420	-10.05350	-16.59810
C	9.72330	-10.56440	-17.57640
H	8.93060	-10.06290	-18.13130
H	10.14180	-11.30990	-18.25190
C	9.11740	-11.26900	-16.35800
H	8.65450	-10.55300	-15.67890
H	8.35150	-11.98250	-16.66230
H	9.87710	-11.81720	-15.80070
C	19.66460	1.40670	-17.26690
H	20.18580	1.35480	-16.31000
H	20.24250	0.79930	-17.96370
C	18.26400	0.80020	-17.10720

H	17.70380	1.38050	-16.37340
H	17.72000	0.88390	-18.04780
C	18.31070	-0.67310	-16.67390
H	18.88720	-0.76110	-15.75260
H	18.84240	-1.25620	-17.42620
C	16.91610	-1.27340	-16.45090
H	16.40530	-0.70710	-15.67160
H	16.31870	-1.16090	-17.35560
C	16.96660	-2.75640	-16.05560
H	17.60060	-2.87600	-15.17670
H	17.43590	-3.33070	-16.85470
C	15.57700	-3.33260	-15.75220
H	15.13130	-2.77160	-14.93020
H	14.92270	-3.18880	-16.61220
C	15.61800	-4.82330	-15.38800
H	16.30440	-4.97570	-14.55460
H	16.02060	-5.39210	-16.22650
C	14.23460	-5.36760	-15.00670
H	13.85750	-4.81220	-14.14720
H	13.53130	-5.18730	-15.81990
C	14.25330	-6.86570	-14.67570
H	14.98270	-7.05620	-13.88760
H	14.58910	-7.42650	-15.54830
C	12.87750	-7.37650	-14.22600
H	12.56590	-6.82930	-13.33550
H	12.13570	-7.15840	-14.99450
C	12.86840	-8.88100	-13.92750
H	13.63200	-9.11000	-13.18340
H	13.14110	-9.43000	-14.82900
C	11.50290	-9.36390	-13.41920
H	11.24870	-8.82700	-12.50460
H	10.73190	-9.11230	-14.14760
C	11.47010	-10.87340	-13.15000
H	12.25900	-11.13560	-12.44420
H	11.69100	-11.40750	-14.07410
C	10.11660	-11.34020	-12.59700
H	9.90480	-10.82120	-11.66160
H	9.32140	-11.06430	-13.28960
C	10.06750	-12.85160	-12.35730
H	10.83260	-13.16430	-11.64640
H	9.09870	-13.15140	-11.95730
H	10.22620	-13.39820	-13.28600
O	25.27590	6.34620	-20.05870
O	26.86010	5.70720	-18.56440
O	25.64440	4.66530	-16.03990
C	26.53590	6.62490	-19.55900
C	27.57000	6.50970	-20.68880
H	27.31860	7.19980	-21.49470
H	28.56190	6.75530	-20.30870
H	27.59180	5.49530	-21.08640
C	26.49160	8.02790	-18.95510
H	27.43960	8.26310	-18.47140
H	26.29220	8.76470	-19.73360
H	25.68780	8.07680	-18.22410
Si	25.49180	5.33800	-14.48680
C	23.79130	6.16800	-14.30110
H	23.58160	6.85430	-15.11930
H	22.98780	5.43340	-14.27570
H	23.71950	6.73470	-13.37380
C	25.59250	3.90620	-13.25780
H	26.53370	3.36900	-13.36210
H	25.52170	4.25270	-12.22730
H	24.78910	3.18870	-13.42050
C	26.90840	6.60030	-14.15800
C	27.42620	7.14330	-15.49840
H	28.20510	7.89240	-15.35480
H	27.84610	6.34680	-16.11330
H	26.62600	7.60530	-16.07140

C	28.06460	5.92130	-13.40380
H	27.74630	5.55050	-12.42920
H	28.46520	5.07860	-13.95980
H	28.88760	6.61580	-13.23270
C	26.38290	7.75880	-13.29150
H	25.99430	7.39720	-12.33880
H	27.17210	8.47830	-13.07100
H	25.58120	8.30400	-13.78750
C	33.46850	-18.49570	-20.60360
C	32.52290	-17.42610	-23.35300
O	33.50720	-17.94510	-19.50440
O	32.48440	-18.40870	-24.09330
N	32.59130	-19.46960	-20.88850
N	31.43730	-16.69590	-23.06510
H	32.62150	-19.86830	-21.81650
H	31.55470	-15.88540	-22.46630
C	34.44000	-18.06080	-21.72110
H	35.25370	-17.55410	-21.20520
C	33.84410	-16.99780	-22.68640
H	33.63560	-16.10780	-22.09510
C	35.06820	-19.25720	-22.46800
H	35.48960	-19.96290	-21.75190
H	34.31240	-19.81380	-23.02140
C	34.86050	-16.58020	-23.77130
H	34.33270	-16.02680	-24.55080
C	36.16570	-18.79270	-23.43760
H	37.00130	-18.40540	-22.85640
C	35.66630	-17.71220	-24.42560
H	35.09340	-18.17450	-25.23030
C	30.09180	-16.99530	-23.52980
H	29.49190	-16.08770	-23.47100
H	30.12090	-17.28160	-24.58270
C	31.51350	-19.90570	-20.01020
H	31.89550	-20.08270	-19.00330
H	30.77610	-19.10470	-19.93820
C	29.45790	-18.10780	-22.68270
H	30.12820	-18.96650	-22.65520
H	29.34550	-17.76980	-21.65180
C	28.10600	-18.56420	-23.23570
H	27.41110	-17.72520	-23.25250
H	28.23440	-18.87780	-24.27260
C	27.50960	-19.72340	-22.42900
H	27.32250	-19.40310	-21.40350
H	28.23510	-20.53510	-22.37290
C	26.21690	-20.25150	-23.05800
H	25.48780	-19.44420	-23.12120
H	26.41810	-20.56330	-24.08380
C	25.61910	-21.42990	-22.28140
H	25.39630	-21.12080	-21.25980
H	26.35670	-22.22940	-22.21090
C	24.35020	-21.96750	-22.95220
H	23.61820	-21.16420	-23.03450
H	24.58350	-22.27460	-23.97250
C	23.73250	-23.14990	-22.19720
H	23.50100	-22.84840	-21.17550
H	24.46270	-23.95620	-22.12640
C	22.46300	-23.66890	-22.88340
H	21.74000	-22.85710	-22.96400
H	22.70040	-23.97060	-23.90430
C	21.82620	-24.84940	-22.14020
H	21.60240	-24.55400	-21.11530
H	22.54350	-25.66800	-22.07900
C	20.54420	-25.34240	-22.82370
H	19.83190	-24.51980	-22.88990
H	20.76980	-25.63670	-23.84950
C	19.89640	-26.52160	-22.08680
H	19.68880	-26.23410	-21.05630
H	20.60200	-27.35150	-22.04230

C	18.59810	-26.98960	-22.75790
H	17.89360	-26.15880	-22.80050
H	18.80470	-27.27100	-23.79130
C	17.94920	-28.17340	-22.02820
H	17.76030	-27.89980	-20.99030
H	18.64760	-29.01050	-22.00700
C	16.63580	-28.62070	-22.68460
H	15.92840	-27.79090	-22.69210
H	16.81790	-28.87980	-23.72820
C	15.99900	-29.81920	-21.97360
H	15.77000	-29.58680	-20.93350
H	15.06800	-30.10960	-22.46060
H	16.66390	-30.68300	-21.98830
C	30.86250	-21.18200	-20.56210
H	31.59430	-21.99100	-20.54940
H	30.59490	-21.03190	-21.60840
C	29.60850	-21.61290	-19.78520
H	29.85770	-21.76120	-18.73380
H	28.86840	-20.81240	-19.81840
C	28.99850	-22.90100	-20.35900
H	29.73140	-23.70590	-20.28930
H	28.79930	-22.76270	-21.42240
C	27.70130	-23.33330	-19.65930
H	27.88570	-23.47090	-18.59320
H	26.95940	-22.53930	-19.74570
C	27.13690	-24.63080	-20.25800
H	27.87870	-25.42440	-20.15960
H	26.97670	-24.49390	-21.32820
C	25.82230	-25.08290	-19.60660
H	25.97310	-25.22680	-18.53600
H	25.07680	-24.29510	-19.71090
C	25.28570	-26.37930	-20.23210
H	26.03180	-27.16780	-20.12530
H	25.14650	-26.23220	-21.30380
C	23.96190	-26.84560	-19.61040
H	24.09690	-27.00700	-18.54020
H	23.21770	-26.05660	-19.71210
C	23.43200	-28.13040	-20.26390
H	24.17850	-28.92000	-20.16910
H	23.29600	-27.96250	-21.33310
C	22.10700	-28.60690	-19.65280
H	22.24220	-28.79030	-18.58620
H	21.36490	-27.81390	-19.73750
C	21.57040	-29.87600	-20.33040
H	22.31730	-30.66790	-20.26120
H	21.42270	-29.68350	-21.39390
C	20.25230	-30.36390	-19.71350
H	20.40110	-30.56970	-18.65270
H	19.50990	-29.56870	-19.77140
C	19.70470	-31.61800	-20.40930
H	20.45240	-32.41120	-20.36960
H	19.53910	-31.40250	-21.46570
C	18.39730	-32.11860	-19.77970
H	18.56100	-32.34640	-18.72580
H	17.64900	-31.32710	-19.81040
C	17.84170	-33.35980	-20.48600
H	18.54920	-34.18830	-20.44240
H	16.91380	-33.68980	-20.01840
H	17.62960	-33.15410	-21.53560
O	35.88240	-15.73840	-23.28760
O	36.78440	-17.00460	-24.93960
O	36.56310	-19.92890	-24.18390
C	36.87110	-15.79190	-24.26000
C	36.66740	-14.64580	-25.26570
H	36.68630	-13.68790	-24.74500
H	37.46580	-14.65960	-26.00800
H	35.71490	-14.74540	-25.78470
C	38.23420	-15.69650	-23.57150

H	39.03300	-15.78410	-24.30810
H	38.34160	-16.49080	-22.83730
H	38.32350	-14.73630	-23.06400
Si	38.15120	-20.47580	-24.40370
C	38.15340	-21.56140	-25.95620
H	37.48570	-22.41510	-25.84660
H	37.82140	-21.00080	-26.82950
H	39.14780	-21.94810	-26.17570
C	39.32770	-19.01210	-24.65250
H	39.38650	-18.38900	-23.76260
H	40.33880	-19.34890	-24.87760
H	39.00530	-18.37750	-25.47720
C	38.67810	-21.51340	-22.88150
C	37.63910	-22.62340	-22.64780
H	37.89590	-23.23170	-21.78010
H	36.64610	-22.20670	-22.47510
H	37.56840	-23.29150	-23.50660
C	38.76080	-20.61520	-21.63610
H	39.46880	-19.79790	-21.77470
H	37.79350	-20.17730	-21.39780
H	39.08260	-21.18000	-20.76070
C	40.05760	-22.13980	-23.14920
H	40.81300	-21.37530	-23.33450
H	40.39480	-22.73370	-22.29920
H	40.03640	-22.79860	-24.01770
C	31.80560	-14.16270	-17.76450
C	31.61210	-13.42620	-20.78090
O	30.74190	-13.54450	-17.77280
O	31.96560	-14.28780	-21.58150
N	31.95850	-15.28640	-17.04940
N	30.34720	-12.98750	-20.69110
H	32.85360	-15.75230	-17.08110
H	30.15970	-12.27990	-19.98930
C	33.01230	-13.65750	-18.58560
H	33.55150	-13.01060	-17.89350
C	32.62480	-12.77990	-19.81420
H	32.11660	-11.89440	-19.44320
C	33.98450	-14.78280	-19.00530
H	34.21320	-15.44230	-18.17160
H	33.52820	-15.40400	-19.77340
C	33.88230	-12.26180	-20.55160
H	33.60090	-11.88370	-21.53650
C	35.30070	-14.19950	-19.52610
H	35.77350	-13.66300	-18.70630
C	35.06720	-13.23700	-20.71230
H	34.96340	-13.80440	-21.63750
C	29.19430	-13.51070	-21.40900
H	28.50460	-12.68250	-21.57350
H	29.48570	-13.87460	-22.39520
C	30.87310	-15.96630	-16.35990
H	31.27970	-16.49930	-15.50000
H	30.15960	-15.24170	-15.96290
C	28.49830	-14.61830	-20.59950
H	29.18150	-15.45990	-20.47650
H	28.27800	-14.25170	-19.59530
C	27.19750	-15.10030	-21.25970
H	26.51390	-14.25730	-21.36280
H	27.41280	-15.44490	-22.27120
C	26.50370	-16.22480	-20.47450
H	26.28220	-15.88280	-19.46240
H	27.18860	-17.06660	-20.37400
C	25.20910	-16.70210	-21.15160
H	24.51720	-15.86530	-21.24060
H	25.42940	-17.02290	-22.16960
C	24.52630	-17.85240	-20.39740
H	24.27360	-17.52660	-19.38790
H	25.22980	-18.67690	-20.28840
C	23.26250	-18.36120	-21.10840

H	22.55330	-17.54200	-21.21850
H	23.51510	-18.68090	-22.11910
C	22.59240	-19.52370	-20.36270
H	22.32400	-19.19830	-19.35800
H	23.30940	-20.33500	-20.24270
C	21.34280	-20.05830	-21.07820
H	20.62280	-19.25050	-21.20360
H	21.60740	-20.38860	-22.08260
C	20.68640	-21.21810	-20.31630
H	20.42710	-20.88420	-19.31190
H	21.40800	-22.02560	-20.19610
C	19.42980	-21.76310	-21.00990
H	18.70560	-20.95820	-21.13240
H	19.68250	-22.10200	-22.01450
C	18.78740	-22.91670	-20.22660
H	18.55260	-22.57770	-19.21780
H	19.50780	-23.72760	-20.11970
C	17.51260	-23.45550	-20.89010
H	16.78990	-22.64670	-20.99520
H	17.74100	-23.79450	-21.90060
C	16.88080	-24.60660	-20.09470
H	16.67570	-24.27210	-19.07770
H	17.59580	-25.42540	-20.01200
C	15.58440	-25.12640	-20.73090
H	14.86020	-24.31480	-20.80440
H	15.78170	-25.45410	-21.75170
C	14.96400	-26.28300	-19.94050
H	14.72350	-25.97920	-18.92140
H	14.04290	-26.62580	-20.41200
H	15.64380	-27.13310	-19.88690
C	30.17910	-16.94860	-17.31460
H	30.85180	-17.78200	-17.52310
H	29.98480	-16.46630	-18.27400
C	28.85220	-17.46560	-16.75440
H	28.99500	-17.82450	-15.73420
H	28.14500	-16.63660	-16.69540
C	28.26200	-18.59240	-17.60830
H	28.90960	-19.46710	-17.53490
H	28.25380	-18.30480	-18.65990
C	26.84010	-18.95620	-17.16950
H	26.81390	-19.07800	-16.08570
H	26.16430	-18.13210	-17.40400
C	26.34100	-20.24600	-17.82640
H	27.00740	-21.06490	-17.55210
H	26.39350	-20.15940	-18.91190
C	24.91000	-20.58850	-17.40000
H	24.84470	-20.58270	-16.31110
H	24.22730	-19.81480	-17.75320
C	24.46520	-21.95770	-17.92190
H	25.16460	-22.71960	-17.57520
H	24.51260	-21.96980	-19.01080
C	23.05080	-22.32190	-17.46070
H	23.00110	-22.27880	-16.37190
H	22.34470	-21.57870	-17.83110
C	22.63260	-23.71780	-17.93380
H	23.35410	-24.45360	-17.57620
H	22.66530	-23.75720	-19.02240
C	21.23190	-24.10320	-17.44890
H	21.19940	-24.05810	-16.35970
H	20.51090	-23.37040	-17.81060
C	20.82030	-25.50410	-17.91570
H	21.55090	-26.23460	-17.56610
H	20.84030	-25.54210	-19.00450
C	19.42630	-25.89870	-17.41760
H	19.40740	-25.86210	-16.32790
H	18.70090	-25.16370	-17.76540
C	19.00410	-27.29500	-17.89060
H	19.73440	-28.03170	-17.55330

H	19.01360	-27.32430	-18.97970
C	17.61110	-27.68510	-17.38340
H	17.59900	-27.65900	-16.29350
H	16.88480	-26.94630	-17.72020
C	17.17160	-29.07200	-17.85980
H	17.86140	-29.84440	-17.51880
H	16.18010	-29.31750	-17.47890
H	17.12880	-29.11550	-18.94740
O	34.46030	-11.18610	-19.84990
O	36.17480	-12.35030	-20.77690
O	36.13810	-15.26870	-19.95070
C	35.76930	-11.10950	-20.29020
C	35.86220	-10.08350	-21.42980
H	35.51640	-9.10940	-21.08250
H	36.89630	-9.99380	-21.76320
H	35.25040	-10.39380	-22.27650
C	36.63910	-10.71780	-19.09150
H	37.69310	-10.73280	-19.36960
H	36.37410	-9.71600	-18.75330
H	36.47130	-11.40710	-18.26540
Si	37.29730	-16.08350	-19.00900
C	36.59500	-16.49200	-17.29940
H	36.34290	-15.59200	-16.74730
H	35.69560	-17.10280	-17.37370
H	37.31360	-17.04810	-16.69860
C	37.71770	-17.72490	-19.85470
H	38.04590	-17.57550	-20.88000
H	38.51480	-18.25260	-19.33320
H	36.86050	-18.39560	-19.87220
C	38.89540	-15.03310	-18.84720
C	38.62550	-13.77490	-18.00900
H	39.53850	-13.20070	-17.84950
H	37.92320	-13.11910	-18.51450
H	38.21550	-14.01930	-17.02890
C	39.36780	-14.62100	-20.25010
H	39.58810	-15.49210	-20.86740
H	38.60460	-14.03510	-20.76440
H	40.27220	-14.01390	-20.20420
C	39.98760	-15.86940	-18.15670
H	40.24470	-16.75470	-18.73820
H	40.90420	-15.29310	-18.02690
H	39.66920	-16.20360	-17.16870

trans-2 monomer

C	-0.58890000	6.76880000	-5.28810000
C	0.70460000	5.01350000	-3.03310000
O	-0.22400000	6.55390000	-6.44220000
O	0.22680000	5.26370000	-1.92210000
N	-1.86560000	6.66320000	-4.90530000
N	0.61610000	3.80480000	-3.59920000
H	-2.07880000	6.94590000	-3.94700000
H	1.04700000	3.67580000	-4.51340000
C	0.41900000	7.23080000	-4.22930000
H	-0.13840000	7.48780000	-3.33160000
C	1.12080000	8.54370000	-4.66380000
H	0.59190000	9.02610000	-5.48790000
H	1.06500000	9.24510000	-3.83500000
C	2.57370000	6.72890000	-3.04410000
H	2.15950000	7.24300000	-2.17870000
C	2.59850000	8.37330000	-5.03780000
H	2.66370000	7.75020000	-5.93070000
C	3.38760000	7.71970000	-3.88190000
H	3.80260000	8.47050000	-3.20940000
C	-0.01410000	2.65810000	-2.96530000
H	-0.97720000	2.95500000	-2.54770000
H	0.60970000	2.32730000	-2.13350000

C	-2.99600000	6.45630000	-5.80090000
H	-2.93890000	7.19560000	-6.60200000
H	-3.91560000	6.67080000	-5.25560000
C	1.41640000	6.10400000	-3.84780000
H	1.82960000	5.65770000	-4.75330000
C	-0.21360000	1.52030000	-3.97100000
H	0.74790000	1.24990000	-4.40960000
H	-0.84350000	1.87240000	-4.78870000
C	-0.85130000	0.27990000	-3.33100000
H	-0.20710000	-0.09350000	-2.53390000
H	-1.79460000	0.56000000	-2.86250000
C	-1.10370000	-0.83530000	-4.35150000
H	-0.16070000	-1.12200000	-4.81820000
H	-1.73970000	-0.44930000	-5.14740000
C	-1.76530000	-2.07370000	-3.73380000
H	-1.10620000	-2.50730000	-2.98080000
H	-2.67730000	-1.77720000	-3.21610000
C	-2.10710000	-3.12900000	-4.79200000
H	-1.19820000	-3.43110000	-5.31370000
H	-2.75880000	-2.68000000	-5.54110000
C	-2.79540000	-4.36680000	-4.20350000
H	-2.12340000	-4.86180000	-3.50140000
H	-3.67070000	-4.05830000	-3.63210000
C	-3.22880000	-5.35580000	-5.29190000
H	-2.36090000	-5.65580000	-5.88050000
H	-3.90880000	-4.85170000	-5.97840000
C	-3.91720000	-6.60260000	-4.72320000
H	-3.22170000	-7.14340000	-4.08030000
H	-4.75280000	-6.30010000	-4.09210000
C	-4.43170000	-7.53370000	-5.82730000
H	-3.60570000	-7.82050000	-6.47950000
H	-5.14190000	-6.98880000	-6.44920000
C	-5.10620000	-8.79390000	-5.27160000
H	-4.38380000	-9.36910000	-4.69100000
H	-5.89940000	-8.50600000	-4.58170000
C	-5.69600000	-9.67440000	-6.37990000
H	-6.42950000	-9.09600000	-6.94160000
H	-4.91180000	-9.94920000	-7.08650000
C	-6.36090000	-10.94370000	-5.83260000
H	-5.61960000	-11.54590000	-5.30560000
H	-7.11440000	-10.66670000	-5.09570000
C	-7.01610000	-11.78300000	-6.93680000
H	-7.76120000	-11.17740000	-7.45250000
H	-6.26730000	-12.05210000	-7.68290000
C	-7.68050000	-13.05540000	-6.39400000
H	-6.93420000	-13.67190000	-5.89140000
H	-8.42280000	-12.79110000	-5.64010000
C	-8.35650000	-13.87940000	-7.49420000
H	-9.15160000	-13.31190000	-7.97680000
H	-8.80090000	-14.78640000	-7.08380000
H	-7.64190000	-14.17730000	-8.26210000
C	-3.05370000	5.04420000	-6.41050000
H	-2.10870000	4.80640000	-6.89710000
H	-3.79970000	5.06100000	-7.20580000
C	-3.40920000	3.92080000	-5.42550000
H	-2.63280000	3.82400000	-4.66510000
H	-4.32990000	4.18200000	-4.90260000
C	-3.59710000	2.58900000	-6.17090000
H	-2.64090000	2.25880000	-6.57920000
H	-4.24940000	2.75780000	-7.02870000
C	-4.21030000	1.47170000	-5.31740000
H	-3.52300000	1.19110000	-4.51960000
H	-5.11440000	1.84230000	-4.83300000
C	-4.56730000	0.24600000	-6.17230000
H	-3.66750000	-0.15200000	-6.64230000
H	-5.22090000	0.56180000	-6.98640000
C	-5.26910000	-0.86540000	-5.38220000
H	-4.59600000	-1.25340000	-4.61820000

H	-6.12880000	-0.44900000	-4.85590000
C	-5.74350000	-2.00570000	-6.29400000
H	-4.89500000	-2.41810000	-6.83970000
H	-6.42340000	-1.60100000	-7.04440000
C	-6.45190000	-3.13170000	-5.53070000
H	-5.75690000	-3.58430000	-4.82370000
H	-7.26750000	-2.71390000	-4.93930000
C	-7.00990000	-4.20690000	-6.47300000
H	-6.20660000	-4.60440000	-7.09310000
H	-7.72610000	-3.74690000	-7.15460000
C	-7.69060000	-5.36140000	-5.72710000
H	-8.46380000	-4.96460000	-5.06800000
H	-6.96200000	-5.85730000	-5.08590000
C	-8.31570000	-6.38330000	-6.68650000
H	-9.06600000	-5.88510000	-7.30140000
H	-7.55430000	-6.75570000	-7.37190000
C	-8.96140000	-7.56780000	-5.95650000
H	-9.69710000	-7.19860000	-5.24070000
H	-8.20220000	-8.09190000	-5.37570000
C	-9.63840000	-8.54890000	-6.92340000
H	-10.41540000	-8.02600000	-7.48240000
H	-8.91280000	-8.89680000	-7.65890000
C	-10.25170000	-9.75780000	-6.20520000
H	-10.96460000	-9.41750000	-5.45300000
H	-9.47200000	-10.29710000	-5.66700000
C	-10.95490000	-10.71680000	-7.17180000
H	-11.77930000	-10.22300000	-7.68720000
H	-11.36230000	-11.57670000	-6.63950000
H	-10.26650000	-11.09220000	-7.92840000
O	4.41060000	6.89580000	-4.39860000
O	3.54250000	5.77610000	-2.63590000
C	4.65990000	5.93290000	-3.43690000
C	5.83350000	6.39200000	-2.56360000
H	6.04250000	5.64250000	-1.80010000
H	6.72210000	6.52950000	-3.17980000
H	5.59800000	7.33480000	-2.07130000
C	4.93420000	4.60080000	-4.13550000
H	5.16390000	3.83290000	-3.39710000
H	4.05450000	4.28590000	-4.69600000
H	5.77630000	4.70010000	-4.82070000
O	3.07750000	9.67280000	-5.34270000
Si	4.66250000	10.10430000	-5.75220000
C	5.41710000	8.82980000	-6.91000000
H	6.49330000	8.96730000	-6.99250000
H	5.24590000	7.81390000	-6.56340000
H	5.00440000	8.91690000	-7.91260000
C	5.70850000	10.25710000	-4.18910000
H	5.19550000	10.83440000	-3.42200000
H	5.94290000	9.28060000	-3.76940000
H	6.65450000	10.75470000	-4.39610000
C	4.55570000	11.79260000	-6.63900000
C	5.93340000	12.15390000	-7.22080000
H	6.26720000	11.41060000	-7.94600000
H	5.90930000	13.11710000	-7.73110000
H	6.69280000	12.21440000	-6.44070000
C	3.51810000	11.69100000	-7.77040000
H	3.77890000	10.91160000	-8.48520000
H	2.53000000	11.44650000	-7.37750000
H	3.43170000	12.62820000	-8.32050000
C	4.10780000	12.86920000	-5.63650000
H	3.12940000	12.63070000	-5.21890000
H	4.80800000	12.96090000	-4.80570000
H	4.03060000	13.84810000	-6.11020000

trans-2 dimer

C	1.04610000	5.30860000	-9.55250000

C	2.21990000	3.31930000	-7.48790000
O	1.27210000	4.90020000	-10.68940000
O	1.93160000	3.58250000	-6.31710000
N	-0.18670000	5.46000000	-9.06580000
N	1.79450000	2.20340000	-8.09700000
H	-0.26780000	5.83170000	-8.11740000
H	2.04850000	2.05320000	-9.07140000
C	2.20420000	5.60220000	-8.59900000
H	1.79030000	5.91620000	-7.64340000
C	3.05370000	6.79680000	-9.10180000
H	2.50030000	7.40120000	-9.82280000
H	3.24520000	7.46190000	-8.26100000
C	4.38790000	4.69560000	-7.73310000
H	4.22660000	5.25170000	-6.80960000
C	4.39260000	6.36950000	-9.71520000
H	4.19120000	5.78430000	-10.61450000
C	5.22320000	5.53120000	-8.71810000
H	5.92400000	6.15650000	-8.16340000
C	0.93060000	1.20630000	-7.48490000
H	0.08820000	1.70450000	-7.00260000
H	1.48640000	0.67920000	-6.70820000
C	-1.40490000	5.02250000	-9.72850000
H	-1.39720000	5.32160000	-10.77840000
H	-2.24290000	5.53980000	-9.26130000
C	3.02870000	4.30700000	-8.35200000
H	3.23440000	3.81860000	-9.30630000
C	0.41770000	0.22160000	-8.54470000
H	1.26680000	-0.26880000	-9.02320000
H	-0.09940000	0.77580000	-9.32850000
C	-0.52330000	-0.84510000	-7.97090000
H	0.00620000	-1.42380000	-7.21340000
H	-1.35720000	-0.35720000	-7.46660000
C	-1.06620000	-1.79050000	-9.05120000
H	-0.23360000	-2.24980000	-9.58580000
H	-1.62580000	-1.21230000	-9.78640000
C	-1.96480000	-2.89220000	-8.47520000
H	-1.38860000	-3.50170000	-7.77810000
H	-2.76930000	-2.43730000	-7.89820000
C	-2.56830000	-3.79090000	-9.56240000
H	-1.76980000	-4.21460000	-10.17290000
H	-3.18020000	-3.18470000	-10.23030000
C	-3.41850000	-4.92760000	-8.98040000
H	-2.79160000	-5.56730000	-8.35800000
H	-4.18090000	-4.50890000	-8.32430000
C	-4.09640000	-5.77360000	-10.06590000
H	-3.34270000	-6.16270000	-10.75170000
H	-4.75410000	-5.13730000	-10.65800000
C	-4.90400000	-6.94030000	-9.48240000
H	-4.23600000	-7.60630000	-8.93480000
H	-5.62080000	-6.55680000	-8.75690000
C	-5.65280000	-7.73560000	-10.55940000
H	-4.94560000	-8.09150000	-11.30980000
H	-6.34590000	-7.07330000	-11.07820000
C	-6.42390000	-8.92860000	-9.97960000
H	-5.72460000	-9.61520000	-9.50090000
H	-7.09750000	-8.57880000	-9.19780000
C	-7.23290000	-9.67940000	-11.04500000
H	-7.95340000	-8.99750000	-11.49670000
H	-6.56880000	-10.00270000	-11.84780000
C	-7.97140000	-10.89650000	-10.47300000
H	-7.24720000	-11.59630000	-10.05400000
H	-8.60780000	-10.57910000	-9.64700000
C	-8.82750000	-11.61270000	-11.52560000
H	-9.57250000	-10.91950000	-11.91630000
H	-8.20150000	-11.90120000	-12.37120000
C	-9.53100000	-12.85500000	-10.96380000
H	-8.78620000	-13.56120000	-10.59480000
H	-10.14220000	-12.57440000	-10.10550000

C	-10.41490000	-13.55110000	-12.00380000
H	-11.20920000	-12.89030000	-12.34960000
H	-10.88520000	-14.44000000	-11.58260000
H	-9.83480000	-13.86200000	-12.87320000
C	-1.56960000	3.49860000	-9.59850000
H	-1.36950000	3.19710000	-8.56960000
H	-0.81750000	2.99960000	-10.21250000
C	-2.96720000	3.01310000	-9.99650000
H	-3.70800000	3.51120000	-9.37000000
H	-3.18200000	3.30980000	-11.02340000
C	-3.11430000	1.49250000	-9.85210000
H	-2.77790000	1.18380000	-8.86180000
H	-2.45870000	0.99630000	-10.56780000
C	-4.55820000	1.02130000	-10.05710000
H	-5.20070000	1.49240000	-9.31210000
H	-4.91720000	1.35970000	-11.02910000
C	-4.69440000	-0.50360000	-9.96060000
H	-4.28710000	-0.85050000	-9.01070000
H	-4.09310000	-0.96980000	-10.74010000
C	-6.14870000	-0.97080000	-10.08900000
H	-6.73780000	-0.54960000	-9.27330000
H	-6.57650000	-0.57680000	-11.01120000
C	-6.27410000	-2.49980000	-10.08180000
H	-5.81280000	-2.90290000	-9.18020000
H	-5.71500000	-2.91120000	-10.92150000
C	-7.73180000	-2.96850000	-10.16100000
H	-8.27830000	-2.60080000	-9.29160000
H	-8.20940000	-2.52320000	-11.03430000
C	-7.85170000	-4.49600000	-10.23930000
H	-7.34340000	-4.94910000	-9.38810000
H	-7.33480000	-4.85270000	-11.12980000
C	-9.31040000	-4.96770000	-10.27170000
H	-9.83130000	-4.47880000	-11.09570000
H	-9.81460000	-4.65010000	-9.35810000
C	-9.43140000	-6.48950000	-10.42530000
H	-8.94940000	-6.79660000	-11.35330000
H	-8.88960000	-6.98490000	-9.61950000
C	-10.89030000	-6.96350000	-10.42600000
H	-11.44190000	-6.43500000	-11.20420000
H	-11.36010000	-6.69390000	-9.47920000
C	-11.01610000	-8.47610000	-10.65060000
H	-10.54900000	-8.73960000	-11.59940000
H	-10.46160000	-9.00830000	-9.87710000
C	-12.47560000	-8.94990000	-10.65180000
H	-13.04040000	-8.40190000	-11.40720000
H	-12.93710000	-8.71530000	-9.69170000
C	-12.60400000	-10.45230000	-10.92210000
H	-12.19700000	-10.71190000	-11.89910000
H	-13.64870000	-10.76360000	-10.90620000
H	-12.07220000	-11.03630000	-10.17090000
O	5.93100000	4.52960000	-9.41270000
O	5.21560000	3.56900000	-7.49400000
C	6.15940000	3.51310000	-8.50460000
C	7.55220000	3.69150000	-7.89030000
H	7.75140000	2.88650000	-7.18280000
H	8.30860000	3.67220000	-8.67510000
H	7.61590000	4.64280000	-7.36260000
C	5.99710000	2.17920000	-9.23000000
H	6.18020000	1.35480000	-8.54080000
H	4.98000000	2.09560000	-9.61150000
H	6.70080000	2.11680000	-10.06030000
O	5.09910000	7.54900000	-10.05160000
Si	6.07840000	7.74240000	-11.41410000
C	4.98130000	7.64040000	-12.94620000
H	5.57380000	7.52320000	-13.85270000
H	4.28420000	6.80700000	-12.89450000
H	4.38230000	8.54180000	-13.06130000
C	7.43790000	6.43980000	-11.42040000

H	7.93650000	6.38750000	-10.45400000
H	7.05360000	5.44610000	-11.63660000
H	8.19560000	6.66380000	-12.16820000
C	6.87580000	9.47550000	-11.30830000
C	7.65700000	9.76030000	-12.60280000
H	7.00400000	9.73310000	-13.47600000
H	8.12620000	10.74410000	-12.57530000
H	8.44650000	9.02540000	-12.76350000
C	5.77030000	10.52770000	-11.12140000
H	5.08500000	10.54670000	-11.96880000
H	5.17950000	10.31650000	-10.23040000
H	6.18500000	11.53000000	-11.01160000
C	7.83080000	9.51470000	-10.10380000
H	7.30630000	9.26880000	-9.18110000
H	8.64530000	8.79840000	-10.21540000
H	8.27640000	10.50170000	-9.97780000
C	-0.58890000	6.76880000	-5.28810000
C	0.70460000	5.01350000	-3.03310000
O	-0.22400000	6.55390000	-6.44220000
O	0.22680000	5.26370000	-1.92210000
N	-1.86560000	6.66320000	-4.90530000
N	0.61610000	3.80480000	-3.59920000
H	-2.07880000	6.94590000	-3.94700000
H	1.04700000	3.67580000	-4.51340000
C	0.41900000	7.23080000	-4.22930000
H	-0.13840000	7.48780000	-3.33160000
C	1.12080000	8.54370000	-4.66380000
H	0.59190000	9.02610000	-5.48790000
H	1.06500000	9.24510000	-3.83500000
C	2.57370000	6.72890000	-3.04410000
H	2.15950000	7.24300000	-2.17870000
C	2.59850000	8.37330000	-5.03780000
H	2.66370000	7.75020000	-5.93070000
C	3.38760000	7.71970000	-3.88190000
H	3.80260000	8.47050000	-3.20940000
C	-0.01410000	2.65810000	-2.96530000
H	-0.97720000	2.95500000	-2.54770000
H	0.60970000	2.32730000	-2.13350000
C	-2.99600000	6.45630000	-5.80090000
H	-2.93890000	7.19560000	-6.60200000
H	-3.91560000	6.67080000	-5.25560000
C	1.41640000	6.10400000	-3.84780000
H	1.82960000	5.65770000	-4.75330000
C	-0.21360000	1.52030000	-3.97100000
H	0.74790000	1.24990000	-4.40960000
H	-0.84350000	1.87240000	-4.78870000
C	-0.85130000	0.27990000	-3.33100000
H	-0.20710000	-0.09350000	-2.53390000
H	-1.79460000	0.56000000	-2.86250000
C	-1.10370000	-0.83530000	-4.35150000
H	-0.16070000	-1.12200000	-4.81820000
H	-1.73970000	-0.44930000	-5.14740000
C	-1.76530000	-2.07370000	-3.73380000
H	-1.10620000	-2.50730000	-2.98080000
H	-2.67730000	-1.77720000	-3.21610000
C	-2.10710000	-3.12900000	-4.79200000
H	-1.19820000	-3.43110000	-5.31370000
H	-2.75880000	-2.68000000	-5.54110000
C	-2.79540000	-4.36680000	-4.20350000
H	-2.12340000	-4.86180000	-3.50140000
H	-3.67070000	-4.05830000	-3.63210000
C	-3.22880000	-5.35580000	-5.29190000
H	-2.36090000	-5.65580000	-5.88050000
H	-3.90880000	-4.85170000	-5.97840000
C	-3.91720000	-6.60260000	-4.72320000
H	-3.22170000	-7.14340000	-4.08030000
H	-4.75280000	-6.30010000	-4.09210000
C	-4.43170000	-7.53370000	-5.82730000

H	-3.60570000	-7.82050000	-6.47950000
H	-5.14190000	-6.98880000	-6.44920000
C	-5.10620000	-8.79390000	-5.27160000
H	-4.38380000	-9.36910000	-4.69100000
H	-5.89940000	-8.50600000	-4.58170000
C	-5.69600000	-9.67440000	-6.37990000
H	-6.42950000	-9.09600000	-6.94160000
H	-4.91180000	-9.94920000	-7.08650000
C	-6.36090000	-10.94370000	-5.83260000
H	-5.61960000	-11.54590000	-5.30560000
H	-7.11440000	-10.66670000	-5.09570000
C	-7.01610000	-11.78300000	-6.93680000
H	-7.76120000	-11.17740000	-7.45250000
H	-6.26730000	-12.05210000	-7.68290000
C	-7.68050000	-13.05540000	-6.39400000
H	-6.93420000	-13.67190000	-5.89140000
H	-8.42280000	-12.79110000	-5.64010000
C	-8.35650000	-13.87940000	-7.49420000
H	-9.15160000	-13.31190000	-7.97680000
H	-8.80090000	-14.78640000	-7.08380000
H	-7.64190000	-14.17730000	-8.26210000
C	-3.05370000	5.04420000	-6.41050000
H	-2.10870000	4.80640000	-6.89710000
H	-3.79970000	5.06100000	-7.20580000
C	-3.40920000	3.92080000	-5.42550000
H	-2.63280000	3.82400000	-4.66510000
H	-4.32990000	4.18200000	-4.90260000
C	-3.59710000	2.58900000	-6.17090000
H	-2.64090000	2.25880000	-6.57920000
H	-4.24940000	2.75780000	-7.02870000
C	-4.21030000	1.47170000	-5.31740000
H	-3.52300000	1.19110000	-4.51960000
H	-5.11440000	1.84230000	-4.83300000
C	-4.56730000	0.24600000	-6.17230000
H	-3.66750000	-0.15200000	-6.64230000
H	-5.22090000	0.56180000	-6.98640000
C	-5.26910000	-0.86540000	-5.38220000
H	-4.59600000	-1.25340000	-4.61820000
H	-6.12880000	-0.44900000	-4.85590000
C	-5.74350000	-2.00570000	-6.29400000
H	-4.89500000	-2.41810000	-6.83970000
H	-6.42340000	-1.60100000	-7.04440000
C	-6.45190000	-3.13170000	-5.53070000
H	-5.75690000	-3.58430000	-4.82370000
H	-7.26750000	-2.71390000	-4.93930000
C	-7.00990000	-4.20690000	-6.47300000
H	-6.20660000	-4.60440000	-7.09310000
H	-7.72610000	-3.74690000	-7.15460000
C	-7.69060000	-5.36140000	-5.72710000
H	-8.46380000	-4.96460000	-5.06800000
H	-6.96200000	-5.85730000	-5.08590000
C	-8.31570000	-6.38330000	-6.68650000
H	-9.06600000	-5.88510000	-7.30140000
H	-7.55430000	-6.75570000	-7.37190000
C	-8.96140000	-7.56780000	-5.95650000
H	-9.69710000	-7.19860000	-5.24070000
H	-8.20220000	-8.09190000	-5.37570000
C	-9.63840000	-8.54890000	-6.92340000
H	-10.41540000	-8.02600000	-7.48240000
H	-8.91280000	-8.89680000	-7.65890000
C	-10.25170000	-9.75780000	-6.20520000
H	-10.96460000	-9.41750000	-5.45300000
H	-9.47200000	-10.29710000	-5.66700000
C	-10.95490000	-10.71680000	-7.17180000
H	-11.77930000	-10.22300000	-7.68720000
H	-11.36230000	-11.57670000	-6.63950000
H	-10.26650000	-11.09220000	-7.92840000
O	4.41060000	6.89580000	-4.39860000

O	3.54250000	5.77610000	-2.63590000
C	4.65990000	5.93290000	-3.43690000
C	5.83350000	6.39200000	-2.56360000
H	6.04250000	5.64250000	-1.80010000
H	6.72210000	6.52950000	-3.17980000
H	5.59800000	7.33480000	-2.07130000
C	4.93420000	4.60080000	-4.13550000
H	5.16390000	3.83290000	-3.39710000
H	4.05450000	4.28590000	-4.69600000
H	5.77630000	4.70010000	-4.82070000
O	3.07750000	9.67280000	-5.34270000
Si	4.66250000	10.10430000	-5.75220000
C	5.41710000	8.82980000	-6.91000000
H	6.49330000	8.96730000	-6.99250000
H	5.24590000	7.81390000	-6.56340000
H	5.00440000	8.91690000	-7.91260000
C	5.70850000	10.25710000	-4.18910000
H	5.19550000	10.83440000	-3.42200000
H	5.94290000	9.28060000	-3.76940000
H	6.65450000	10.75470000	-4.39610000
C	4.55570000	11.79260000	-6.63900000
C	5.93340000	12.15390000	-7.22080000
H	6.26720000	11.41060000	-7.94600000
H	5.90930000	13.11710000	-7.73110000
H	6.69280000	12.21440000	-6.44070000
C	3.51810000	11.69100000	-7.77040000
H	3.77890000	10.91160000	-8.48520000
H	2.53000000	11.44650000	-7.37750000
H	3.43170000	12.62820000	-8.32050000
C	4.10780000	12.86920000	-5.63650000
H	3.12940000	12.63070000	-5.21890000
H	4.80800000	12.96090000	-4.80570000
H	4.03060000	13.84810000	-6.11020000

trans-2 tetramer

C	2.80980000	3.97920000	-13.80610000
C	2.31160000	1.18350000	-12.16100000
O	3.12180000	3.93030000	-14.99400000
O	2.23310000	1.38530000	-10.94690000
N	1.72040000	4.61980000	-13.37270000
N	1.38320000	0.51280000	-12.85110000
H	1.54470000	4.64040000	-12.36510000
H	1.53150000	0.40950000	-13.84350000
C	3.65390000	3.24550000	-12.75580000
H	3.28050000	3.53520000	-11.77290000
C	5.12590000	3.71400000	-12.79680000
H	5.18560000	4.76750000	-13.06360000
H	5.55660000	3.63430000	-11.79990000
C	4.78440000	0.91610000	-12.62620000
H	4.94850000	0.92010000	-11.55010000
C	5.97710000	2.90450000	-13.77600000
H	5.54270000	2.98900000	-14.77310000
C	6.03840000	1.42590000	-13.35300000
H	6.91000000	1.23670000	-12.72440000
C	0.24020000	-0.16700000	-12.26890000
H	-0.44230000	0.56970000	-11.84330000
H	0.58380000	-0.80140000	-11.45030000
C	0.75930000	5.31520000	-14.21630000
H	1.29180000	5.90790000	-14.96280000
H	0.20840000	6.02330000	-13.59640000
C	3.50230000	1.70860000	-12.97210000
H	3.31090000	1.51490000	-14.02970000
C	-0.46460000	-1.01620000	-13.33670000
H	0.26230000	-1.67120000	-13.81950000
H	-0.86300000	-0.36640000	-14.11720000

C	-1.59390000	-1.87510000	-12.75640000
H	-1.17910000	-2.54450000	-12.00220000
H	-2.30960000	-1.23460000	-12.24190000
C	-2.31920000	-2.70370000	-13.82690000
H	-1.59360000	-3.29570000	-14.38640000
H	-2.79220000	-2.03550000	-14.54710000
C	-3.37390000	-3.63920000	-13.22210000
H	-2.88620000	-4.33380000	-12.53760000
H	-4.07380000	-3.05610000	-12.62390000
C	-4.14830000	-4.43280000	-14.28360000
H	-3.44800000	-4.98510000	-14.91170000
H	-4.67480000	-3.74190000	-14.94260000
C	-5.15130000	-5.41140000	-13.65820000
H	-4.61420000	-6.12730000	-13.03520000
H	-5.82150000	-4.86580000	-12.99380000
C	-5.97850000	-6.16990000	-14.70520000
H	-5.31050000	-6.69330000	-15.39060000
H	-6.54250000	-5.45740000	-15.30790000
C	-6.94350000	-7.17710000	-14.06500000
H	-6.37320000	-7.91060000	-13.49430000
H	-7.58350000	-6.65930000	-13.35030000
C	-7.81580000	-7.90400000	-15.09760000
H	-7.17810000	-8.41170000	-15.82240000
H	-8.39850000	-7.17270000	-15.65860000
C	-8.76260000	-8.92240000	-14.44800000
H	-8.17780000	-9.67250000	-13.91480000
H	-9.37430000	-8.41820000	-13.69970000
C	-9.67290000	-9.61950000	-15.46850000
H	-10.24990000	-8.87000000	-16.01110000
H	-9.06120000	-10.13390000	-16.21080000
C	-10.63130000	-10.62190000	-14.81050000
H	-10.05530000	-11.38350000	-14.28400000
H	-11.22840000	-10.10780000	-14.05640000
C	-11.56310000	-11.30080000	-15.82390000
H	-12.12630000	-10.54120000	-16.36680000
H	-10.96720000	-11.83060000	-16.56840000
C	-12.53980000	-12.28000000	-15.15810000
H	-11.98030000	-13.06570000	-14.64960000
H	-13.11380000	-11.76120000	-14.38880000
C	-13.50440000	-12.92030000	-16.16220000
H	-14.11150000	-12.16580000	-16.66350000
H	-14.18270000	-13.61260000	-15.66260000
H	-12.96480000	-13.47930000	-16.92740000
C	-0.21630000	4.34620000	-14.90940000
H	0.34750000	3.66960000	-15.55370000
H	-0.85560000	4.92500000	-15.57710000
C	-1.10110000	3.52840000	-13.94870000
H	-0.47820000	2.93290000	-13.27960000
H	-1.67180000	4.20780000	-13.31440000
C	-2.05930000	2.60720000	-14.72160000
H	-1.47350000	1.89580000	-15.30550000
H	-2.62080000	3.20150000	-15.44390000
C	-3.05250000	1.84220000	-13.83280000
H	-2.51030000	1.24810000	-13.09730000
H	-3.66060000	2.55130000	-13.27040000
C	-3.96830000	0.93200000	-14.66700000
H	-3.35550000	0.23780000	-15.24340000
H	-4.50780000	1.53780000	-15.39650000
C	-4.97950000	0.13760000	-13.82690000
H	-4.44740000	-0.47950000	-13.10330000
H	-5.59940000	0.82610000	-13.25200000
C	-5.88080000	-0.75240000	-14.69620000
H	-5.25970000	-1.40580000	-15.30990000
H	-6.44390000	-0.12660000	-15.39000000
C	-6.85580000	-1.60710000	-13.87310000
H	-6.29210000	-2.24870000	-13.19600000
H	-7.47060000	-0.96010000	-13.24620000
C	-7.76640000	-2.47290000	-14.75590000

H	-7.15390000	-3.08540000	-15.41840000
H	-8.36430000	-1.82720000	-15.40050000
C	-8.69490000	-3.38220000	-13.93770000
H	-9.29150000	-2.77600000	-13.25490000
H	-8.09440000	-4.04620000	-13.31580000
C	-9.62950000	-4.21970000	-14.82180000
H	-10.25810000	-3.55470000	-15.41550000
H	-9.03690000	-4.79770000	-15.53170000
C	-10.51630000	-5.17090000	-14.00550000
H	-11.09170000	-4.59890000	-13.27660000
H	-9.88510000	-5.85140000	-13.43370000
C	-11.47550000	-5.98470000	-14.88480000
H	-12.13070000	-5.30560000	-15.43220000
H	-10.90390000	-6.53220000	-15.63490000
C	-12.32360000	-6.97080000	-14.06960000
H	-12.88750000	-6.43150000	-13.30760000
H	-11.66890000	-7.66010000	-13.53680000
C	-13.29420000	-7.77500000	-14.94040000
H	-14.01650000	-7.12320000	-15.43250000
H	-13.84990000	-8.49560000	-14.33960000
H	-12.76290000	-8.32870000	-15.71380000
O	6.09080000	0.60700000	-14.49930000
O	4.67160000	-0.42730000	-13.07330000
C	5.53970000	-0.60610000	-14.13800000
C	6.65970000	-1.56080000	-13.70720000
H	6.23750000	-2.52760000	-13.43290000
H	7.36450000	-1.70050000	-14.52690000
H	7.19490000	-1.15810000	-12.84760000
C	4.73740000	-1.13430000	-15.32770000
H	4.26890000	-2.08410000	-15.06990000
H	3.96210000	-0.41800000	-15.59920000
H	5.39560000	-1.28090000	-16.18430000
O	7.27240000	3.47140000	-13.78680000
Si	8.45400000	3.13730000	-14.94720000
C	7.64280000	2.94890000	-16.63910000
H	8.38260000	2.79720000	-17.42300000
H	6.96480000	2.09640000	-16.65830000
H	7.06230000	3.83330000	-16.89840000
C	9.37410000	1.56460000	-14.45730000
H	9.75090000	1.63050000	-13.43790000
H	8.72670000	0.69150000	-14.51360000
H	10.22430000	1.38240000	-15.11240000
C	9.65380000	4.62180000	-14.94560000
C	10.72870000	4.42280000	-16.02780000
H	10.28540000	4.34840000	-17.02130000
H	11.43210000	5.25580000	-16.04560000
H	11.30370000	3.51220000	-15.85670000
C	8.85240000	5.90360000	-15.23280000
H	8.38670000	5.86950000	-16.21820000
H	8.05240000	6.04110000	-14.50400000
H	9.48670000	6.78950000	-15.19890000
C	10.31870000	4.72200000	-13.56220000
H	9.57270000	4.79620000	-12.77020000
H	10.92860000	3.84380000	-13.34850000
H	10.96590000	5.59630000	-13.49100000
C	1.04610000	5.30860000	-9.55250000
C	2.21990000	3.31930000	-7.48790000
O	1.27210000	4.90020000	-10.68940000
O	1.93160000	3.58250000	-6.31710000
N	-0.18670000	5.46000000	-9.06580000
N	1.79450000	2.20340000	-8.09700000
H	-0.26780000	5.83170000	-8.11740000
H	2.04850000	2.05320000	-9.07140000
C	2.20420000	5.60220000	-8.59900000
H	1.79030000	5.91620000	-7.64340000
C	3.05370000	6.79680000	-9.10180000
H	2.50030000	7.40120000	-9.82280000
H	3.24520000	7.46190000	-8.26100000

C	4.38790000	4.69560000	-7.73310000
H	4.22660000	5.25170000	-6.80960000
C	4.39260000	6.36950000	-9.71520000
H	4.19120000	5.78430000	-10.61450000
C	5.22320000	5.53120000	-8.71810000
H	5.92400000	6.15650000	-8.16340000
C	0.93060000	1.20630000	-7.48490000
H	0.08820000	1.70450000	-7.00260000
H	1.48640000	0.67920000	-6.70820000
C	-1.40490000	5.02250000	-9.72850000
H	-1.39720000	5.32160000	-10.77840000
H	-2.24290000	5.53980000	-9.26130000
C	3.02870000	4.30700000	-8.35200000
H	3.23440000	3.81860000	-9.30630000
C	0.41770000	0.22160000	-8.54470000
H	1.26680000	-0.26880000	-9.02320000
H	-0.09940000	0.77580000	-9.32850000
C	-0.52330000	-0.84510000	-7.97090000
H	0.00620000	-1.42380000	-7.21340000
H	-1.35720000	-0.35720000	-7.46660000
C	-1.06620000	-1.79050000	-9.05120000
H	-0.23360000	-2.24980000	-9.58580000
H	-1.62580000	-1.21230000	-9.78640000
C	-1.96480000	-2.89220000	-8.47520000
H	-1.38860000	-3.50170000	-7.77810000
H	-2.76930000	-2.43730000	-7.89820000
C	-2.56830000	-3.79090000	-9.56240000
H	-1.76980000	-4.21460000	-10.17290000
H	-3.18020000	-3.18470000	-10.23030000
C	-3.41850000	-4.92760000	-8.98040000
H	-2.79160000	-5.56730000	-8.35800000
H	-4.18090000	-4.50890000	-8.32430000
C	-4.09640000	-5.77360000	-10.06590000
H	-3.34270000	-6.16270000	-10.75170000
H	-4.75410000	-5.13730000	-10.65800000
C	-4.90400000	-6.94030000	-9.48240000
H	-4.23600000	-7.60630000	-8.93480000
H	-5.62080000	-6.55680000	-8.75690000
C	-5.65280000	-7.73560000	-10.55940000
H	-4.94560000	-8.09150000	-11.30980000
H	-6.34590000	-7.07330000	-11.07820000
C	-6.42390000	-8.92860000	-9.97960000
H	-5.72460000	-9.61520000	-9.50090000
H	-7.09750000	-8.57880000	-9.19780000
C	-7.23290000	-9.67940000	-11.04500000
H	-7.95340000	-8.99750000	-11.49670000
H	-6.56880000	-10.00270000	-11.84780000
C	-7.97140000	-10.89650000	-10.47300000
H	-7.24720000	-11.59630000	-10.05400000
H	-8.60780000	-10.57910000	-9.64700000
C	-8.82750000	-11.61270000	-11.52560000
H	-9.57250000	-10.91950000	-11.91630000
H	-8.20150000	-11.90120000	-12.37120000
C	-9.53100000	-12.85500000	-10.96380000
H	-8.78620000	-13.56120000	-10.59480000
H	-10.14220000	-12.57440000	-10.10550000
C	-10.41490000	-13.55110000	-12.00380000
H	-11.20920000	-12.89030000	-12.34960000
H	-10.88520000	-14.44000000	-11.58260000
H	-9.83480000	-13.86200000	-12.87320000
C	-1.56960000	3.49860000	-9.59850000
H	-1.36950000	3.19710000	-8.56960000
H	-0.81750000	2.99960000	-10.21250000
C	-2.96720000	3.01310000	-9.99650000
H	-3.70800000	3.51120000	-9.37000000
H	-3.18200000	3.30980000	-11.02340000
C	-3.11430000	1.49250000	-9.85210000
H	-2.77790000	1.18380000	-8.86180000

H	-2.45870000	0.99630000	-10.56780000
C	-4.55820000	1.02130000	-10.05710000
H	-5.20070000	1.49240000	-9.31210000
H	-4.91720000	1.35970000	-11.02910000
C	-4.69440000	-0.50360000	-9.96060000
H	-4.28710000	-0.85050000	-9.01070000
H	-4.09310000	-0.96980000	-10.74010000
C	-6.14870000	-0.97080000	-10.08900000
H	-6.73780000	-0.54960000	-9.27330000
H	-6.57650000	-0.57680000	-11.01120000
C	-6.27410000	-2.49980000	-10.08180000
H	-5.81280000	-2.90290000	-9.18020000
H	-5.71500000	-2.91120000	-10.92150000
C	-7.73180000	-2.96850000	-10.16100000
H	-8.27830000	-2.60080000	-9.29160000
H	-8.20940000	-2.52320000	-11.03430000
C	-7.85170000	-4.49600000	-10.23930000
H	-7.34340000	-4.94910000	-9.38810000
H	-7.33480000	-4.85270000	-11.12980000
C	-9.31040000	-4.96770000	-10.27170000
H	-9.83130000	-4.47880000	-11.09570000
H	-9.81460000	-4.65010000	-9.35810000
C	-9.43140000	-6.48950000	-10.42530000
H	-8.94940000	-6.79660000	-11.35330000
H	-8.88960000	-6.98490000	-9.61950000
C	-10.89030000	-6.96350000	-10.42600000
H	-11.44190000	-6.43500000	-11.20420000
H	-11.36010000	-6.69390000	-9.47920000
C	-11.01610000	-8.47610000	-10.65060000
H	-10.54900000	-8.73960000	-11.59940000
H	-10.46160000	-9.00830000	-9.87710000
C	-12.47560000	-8.94990000	-10.65180000
H	-13.04040000	-8.40190000	-11.40720000
H	-12.93710000	-8.71530000	-9.69170000
C	-12.60400000	-10.45230000	-10.92210000
H	-12.19700000	-10.71190000	-11.89910000
H	-13.64870000	-10.76360000	-10.90620000
H	-12.07220000	-11.03630000	-10.17090000
O	5.93100000	4.52960000	-9.41270000
O	5.21560000	3.56900000	-7.49400000
C	6.15940000	3.51310000	-8.50460000
C	7.55220000	3.69150000	-7.89030000
H	7.75140000	2.88650000	-7.18280000
H	8.30860000	3.67220000	-8.67510000
H	7.61590000	4.64280000	-7.36260000
C	5.99710000	2.17920000	-9.23000000
H	6.18020000	1.35480000	-8.54080000
H	4.98000000	2.09560000	-9.61150000
H	6.70080000	2.11680000	-10.06030000
O	5.09910000	7.54900000	-10.05160000
Si	6.07840000	7.74240000	-11.41410000
C	4.98130000	7.64040000	-12.94620000
H	5.57380000	7.52320000	-13.85270000
H	4.28420000	6.80700000	-12.89450000
H	4.38230000	8.54180000	-13.06130000
C	7.43790000	6.43980000	-11.42040000
H	7.93650000	6.38750000	-10.45400000
H	7.05360000	5.44610000	-11.63660000
H	8.19560000	6.66380000	-12.16820000
C	6.87580000	9.47550000	-11.30830000
C	7.65700000	9.76030000	-12.60280000
H	7.00400000	9.73310000	-13.47600000
H	8.12620000	10.74410000	-12.57530000
H	8.44650000	9.02540000	-12.76350000
C	5.77030000	10.52770000	-11.12140000
H	5.08500000	10.54670000	-11.96880000
H	5.17950000	10.31650000	-10.23040000
H	6.18500000	11.53000000	-11.01160000

C	7.83080000	9.51470000	-10.10380000
H	7.30630000	9.26880000	-9.18110000
H	8.64530000	8.79840000	-10.21540000
H	8.27640000	10.50170000	-9.97780000
C	-0.58890000	6.76880000	-5.28810000
C	0.70460000	5.01350000	-3.03310000
O	-0.22400000	6.55390000	-6.44220000
O	0.22680000	5.26370000	-1.92210000
N	-1.86560000	6.66320000	-4.90530000
N	0.61610000	3.80480000	-3.59920000
H	-2.07880000	6.94590000	-3.94700000
H	1.04700000	3.67580000	-4.51340000
C	0.41900000	7.23080000	-4.22930000
H	-0.13840000	7.48780000	-3.33160000
C	1.12080000	8.54370000	-4.66380000
H	0.59190000	9.02610000	-5.48790000
H	1.06500000	9.24510000	-3.83500000
C	2.57370000	6.72890000	-3.04410000
H	2.15950000	7.24300000	-2.17870000
C	2.59850000	8.37330000	-5.03780000
H	2.66370000	7.75020000	-5.93070000
C	3.38760000	7.71970000	-3.88190000
H	3.80260000	8.47050000	-3.20940000
C	-0.01410000	2.65810000	-2.96530000
H	-0.97720000	2.95500000	-2.54770000
H	0.60970000	2.32730000	-2.13350000
C	-2.99600000	6.45630000	-5.80090000
H	-2.93890000	7.19560000	-6.60200000
H	-3.91560000	6.67080000	-5.25560000
C	1.41640000	6.10400000	-3.84780000
H	1.82960000	5.65770000	-4.75330000
C	-0.21360000	1.52030000	-3.97100000
H	0.74790000	1.24990000	-4.40960000
H	-0.84350000	1.87240000	-4.78870000
C	-0.85130000	0.27990000	-3.33100000
H	-0.20710000	-0.09350000	-2.53390000
H	-1.79460000	0.56000000	-2.86250000
C	-1.10370000	-0.83530000	-4.35150000
H	-0.16070000	-1.12200000	-4.81820000
H	-1.73970000	-0.44930000	-5.14740000
C	-1.76530000	-2.07370000	-3.73380000
H	-1.10620000	-2.50730000	-2.98080000
H	-2.67730000	-1.77720000	-3.21610000
C	-2.10710000	-3.12900000	-4.79200000
H	-1.19820000	-3.43110000	-5.31370000
H	-2.75880000	-2.68000000	-5.54110000
C	-2.79540000	-4.36680000	-4.20350000
H	-2.12340000	-4.86180000	-3.50140000
H	-3.67070000	-4.05830000	-3.63210000
C	-3.22880000	-5.35580000	-5.29190000
H	-2.36090000	-5.65580000	-5.88050000
H	-3.90880000	-4.85170000	-5.97840000
C	-3.91720000	-6.60260000	-4.72320000
H	-3.22170000	-7.14340000	-4.08030000
H	-4.75280000	-6.30010000	-4.09210000
C	-4.43170000	-7.53370000	-5.82730000
H	-3.60570000	-7.82050000	-6.47950000
H	-5.14190000	-6.98880000	-6.44920000
C	-5.10620000	-8.79390000	-5.27160000
H	-4.38380000	-9.36910000	-4.69100000
H	-5.89940000	-8.50600000	-4.58170000
C	-5.69600000	-9.67440000	-6.37990000
H	-6.42950000	-9.09600000	-6.94160000
H	-4.91180000	-9.94920000	-7.08650000
C	-6.36090000	-10.94370000	-5.83260000
H	-5.61960000	-11.54590000	-5.30560000
H	-7.11440000	-10.66670000	-5.09570000
C	-7.01610000	-11.78300000	-6.93680000

H	-7.76120000	-11.17740000	-7.45250000
H	-6.26730000	-12.05210000	-7.68290000
C	-7.68050000	-13.05540000	-6.39400000
H	-6.93420000	-13.67190000	-5.89140000
H	-8.42280000	-12.79110000	-5.64010000
C	-8.35650000	-13.87940000	-7.49420000
H	-9.15160000	-13.31190000	-7.97680000
H	-8.80090000	-14.78640000	-7.08380000
H	-7.64190000	-14.17730000	-8.26210000
C	-3.05370000	5.04420000	-6.41050000
H	-2.10870000	4.80640000	-6.89710000
H	-3.79970000	5.06100000	-7.20580000
C	-3.40920000	3.92080000	-5.42550000
H	-2.63280000	3.82400000	-4.66510000
H	-4.32990000	4.18200000	-4.90260000
C	-3.59710000	2.58900000	-6.17090000
H	-2.64090000	2.25880000	-6.57920000
H	-4.24940000	2.75780000	-7.02870000
C	-4.21030000	1.47170000	-5.31740000
H	-3.52300000	1.19110000	-4.51960000
H	-5.11440000	1.84230000	-4.83300000
C	-4.56730000	0.24600000	-6.17230000
H	-3.66750000	-0.15200000	-6.64230000
H	-5.22090000	0.56180000	-6.98640000
C	-5.26910000	-0.86540000	-5.38220000
H	-4.59600000	-1.25340000	-4.61820000
H	-6.12880000	-0.44900000	-4.85590000
C	-5.74350000	-2.00570000	-6.29400000
H	-4.89500000	-2.41810000	-6.83970000
H	-6.42340000	-1.60100000	-7.04440000
C	-6.45190000	-3.13170000	-5.53070000
H	-5.75690000	-3.58430000	-4.82370000
H	-7.26750000	-2.71390000	-4.93930000
C	-7.00990000	-4.20690000	-6.47300000
H	-6.20660000	-4.60440000	-7.09310000
H	-7.72610000	-3.74690000	-7.15460000
C	-7.69060000	-5.36140000	-5.72710000
H	-8.46380000	-4.96460000	-5.06800000
H	-6.96200000	-5.85730000	-5.08590000
C	-8.31570000	-6.38330000	-6.68650000
H	-9.06600000	-5.88510000	-7.30140000
H	-7.55430000	-6.75570000	-7.37190000
C	-8.96140000	-7.56780000	-5.95650000
H	-9.69710000	-7.19860000	-5.24070000
H	-8.20220000	-8.09190000	-5.37570000
C	-9.63840000	-8.54890000	-6.92340000
H	-10.41540000	-8.02600000	-7.48240000
H	-8.91280000	-8.89680000	-7.65890000
C	-10.25170000	-9.75780000	-6.20520000
H	-10.96460000	-9.41750000	-5.45300000
H	-9.47200000	-10.29710000	-5.66700000
C	-10.95490000	-10.71680000	-7.17180000
H	-11.77930000	-10.22300000	-7.68720000
H	-11.36230000	-11.57670000	-6.63950000
H	-10.26650000	-11.09220000	-7.92840000
O	4.41060000	6.89580000	-4.39860000
O	3.54250000	5.77610000	-2.63590000
C	4.65990000	5.93290000	-3.43690000
C	5.83350000	6.39200000	-2.56360000
H	6.04250000	5.64250000	-1.80010000
H	6.72210000	6.52950000	-3.17980000
H	5.59800000	7.33480000	-2.07130000
C	4.93420000	4.60080000	-4.13550000
H	5.16390000	3.83290000	-3.39710000
H	4.05450000	4.28590000	-4.69600000
H	5.77630000	4.70010000	-4.82070000
O	3.07750000	9.67280000	-5.34270000
Si	4.66250000	10.10430000	-5.75220000

C	5.41710000	8.82980000	-6.91000000
H	6.49330000	8.96730000	-6.99250000
H	5.24590000	7.81390000	-6.56340000
H	5.00440000	8.91690000	-7.91260000
C	5.70850000	10.25710000	-4.18910000
H	5.19550000	10.83440000	-3.42200000
H	5.94290000	9.28060000	-3.76940000
H	6.65450000	10.75470000	-4.39610000
C	4.55570000	11.79260000	-6.63900000
C	5.93340000	12.15390000	-7.22080000
H	6.26720000	11.41060000	-7.94600000
H	5.90930000	13.11710000	-7.73110000
H	6.69280000	12.21440000	-6.44070000
C	3.51810000	11.69100000	-7.77040000
H	3.77890000	10.91160000	-8.48520000
H	2.53000000	11.44650000	-7.37750000
H	3.43170000	12.62820000	-8.32050000
C	4.10780000	12.86920000	-5.63650000
H	3.12940000	12.63070000	-5.21890000
H	4.80800000	12.96090000	-4.80570000
H	4.03060000	13.84810000	-6.11020000
C	-2.54090000	7.49280000	-1.20980000
C	-0.95320000	6.57910000	1.38660000
O	-2.36480000	7.77990000	-2.39120000
O	-1.64780000	6.72610000	2.39100000
N	-3.19900000	6.39040000	-0.83740000
N	-0.50900000	5.38350000	0.98180000
H	-3.30930000	6.23020000	0.15480000
H	-0.01640000	5.33410000	0.09290000
C	-1.98500000	8.37960000	-0.08380000
H	-2.74130000	8.38730000	0.70350000
C	-1.81250000	9.86310000	-0.52100000
H	-2.50290000	10.12320000	-1.32520000
H	-2.08910000	10.51170000	0.31120000
C	0.16150000	8.85580000	1.20250000
H	-0.42990000	9.29600000	2.00690000
C	-0.37120000	10.19710000	-0.94420000
H	-0.11290000	9.59190000	-1.81420000
C	0.62490000	9.92530000	0.20690000
H	0.84610000	10.84770000	0.74660000
C	-0.77770000	4.13630000	1.67900000
H	-1.77410000	4.16950000	2.12470000
H	-0.06760000	4.02210000	2.49940000
C	-3.55490000	5.30360000	-1.73620000
H	-2.65970000	4.98790000	-2.27660000
H	-4.27180000	5.65490000	-2.48020000
C	-0.65760000	7.77030000	0.46980000
H	-0.05520000	7.41220000	-0.36730000
C	-0.68650000	2.95060000	0.71180000
H	0.34080000	2.83560000	0.36280000
H	-1.28700000	3.16030000	-0.17300000
C	-1.17270000	1.64560000	1.35460000
H	-0.52360000	1.38280000	2.19110000
H	-2.16780000	1.80220000	1.77380000
C	-1.23170000	0.48610000	0.35400000
H	-0.23380000	0.28940000	-0.04030000
H	-1.84860000	0.77960000	-0.49550000
C	-1.80660000	-0.79330000	0.97560000
H	-1.16470000	-1.12450000	1.79310000
H	-2.78180000	-0.57790000	1.41500000
C	-1.95610000	-1.91790000	-0.05500000
H	-0.98480000	-2.13500000	-0.50130000
H	-2.59870000	-1.57360000	-0.86530000
C	-2.54290000	-3.20190000	0.54650000
H	-1.88140000	-3.57410000	1.33000000
H	-3.49710000	-2.97860000	1.02590000
C	-2.74980000	-4.28830000	-0.51580000
H	-1.80270000	-4.49160000	-1.01730000

H	-3.42840000	-3.91120000	-1.28050000
C	-3.31130000	-5.59490000	0.06120000
H	-2.61360000	-5.99840000	0.79620000
H	-4.24070000	-5.39100000	0.59490000
C	-3.57060000	-6.63810000	-1.03350000
H	-2.65110000	-6.81400000	-1.59360000
H	-4.29080000	-6.23490000	-1.74490000
C	-4.09330000	-7.97190000	-0.48320000
H	-3.35500000	-8.39840000	0.19730000
H	-4.99400000	-7.79780000	0.10690000
C	-4.40320000	-8.97450000	-1.60330000
H	-5.16520000	-8.55360000	-2.25880000
H	-3.51430000	-9.11930000	-2.21890000
C	-4.88190000	-10.33320000	-1.07430000
H	-4.10720000	-10.76910000	-0.44220000
H	-5.75600000	-10.19090000	-0.43750000
C	-5.22970000	-11.30850000	-2.20770000
H	-6.02350000	-10.88040000	-2.81990000
H	-4.36640000	-11.42830000	-2.86380000
C	-5.67150000	-12.68380000	-1.68960000
H	-4.87340000	-13.12330000	-1.08980000
H	-6.52780000	-12.56900000	-1.02420000
C	-6.03930000	-13.64620000	-2.82430000
H	-6.86660000	-13.25770000	-3.41740000
H	-6.34340000	-14.61580000	-2.42940000
H	-5.19510000	-13.80970000	-3.49470000
C	-4.13040000	4.12550000	-0.93680000
H	-5.07880000	4.42130000	-0.48590000
H	-3.46360000	3.88410000	-0.10880000
C	-4.33880000	2.86930000	-1.79310000
H	-5.03620000	3.09340000	-2.60130000
H	-3.39680000	2.59130000	-2.26740000
C	-4.86380000	1.68240000	-0.97110000
H	-5.80660000	1.95960000	-0.49730000
H	-4.16830000	1.45950000	-0.16090000
C	-5.07450000	0.42500000	-1.82480000
H	-5.76560000	0.65660000	-2.63600000
H	-4.13320000	0.13790000	-2.29420000
C	-5.62220000	-0.75960000	-1.01600000
H	-6.54600000	-0.46290000	-0.51740000
H	-4.91790000	-1.02430000	-0.22640000
C	-5.89440000	-1.98330000	-1.90090000
H	-6.59880000	-1.70540000	-2.68570000
H	-4.97460000	-2.28110000	-2.40470000
C	-6.45660000	-3.17920000	-1.12060000
H	-7.35290000	-2.87410000	-0.57880000
H	-5.73480000	-3.49830000	-0.36810000
C	-6.79790000	-4.35440000	-2.04660000
H	-7.53930000	-4.03030000	-2.77800000
H	-5.91160000	-4.63870000	-2.61390000
C	-7.33290000	-5.57990000	-1.29460000
H	-8.19880000	-5.29450000	-0.69590000
H	-6.57650000	-5.93560000	-0.59420000
C	-7.72580000	-6.71190000	-2.25360000
H	-6.87320000	-6.96290000	-2.88470000
H	-8.51090000	-6.36050000	-2.92410000
C	-8.20620000	-7.97430000	-1.52670000
H	-7.41130000	-8.34190000	-0.87680000
H	-9.04700000	-7.72710000	-0.87720000
C	-8.62340000	-9.07880000	-2.50700000
H	-7.79720000	-9.28770000	-3.18640000
H	-9.44760000	-8.72260000	-3.12640000
C	-9.03880000	-10.37570000	-1.80080000
H	-8.21110000	-10.73370000	-1.18740000
H	-9.86420000	-10.17270000	-1.11710000
C	-9.45190000	-11.47010000	-2.79420000
H	-8.63990000	-11.64620000	-3.49890000
H	-10.30260000	-11.12820000	-3.38490000

C	-9.81250000	-12.78990000	-2.10460000
H	-8.96920000	-13.18200000	-1.53560000
H	-10.09630000	-13.54540000	-2.83770000
H	-10.65010000	-12.66140000	-1.41840000
O	1.82170000	9.33840000	-0.26810000
O	1.38960000	8.36720000	1.72140000
C	2.38940000	8.71170000	0.83070000
C	3.36150000	9.67900000	1.51980000
H	3.82470000	9.18990000	2.37690000
H	4.14080000	9.98160000	0.82010000
H	2.83930000	10.56800000	1.87150000
C	3.08040000	7.42400000	0.38100000
H	3.57280000	6.95160000	1.23080000
H	2.34760000	6.72310000	-0.01700000
H	3.82560000	7.64440000	-0.38250000
O	-0.32190000	11.57180000	-1.27900000
Si	0.70120000	12.25590000	-2.43850000
C	-0.04130000	11.92490000	-4.14030000
H	0.70030000	12.04080000	-4.92970000
H	-0.45040000	10.91970000	-4.21070000
H	-0.85940000	12.60960000	-4.35540000
C	2.43950000	11.54180000	-2.28370000
H	2.81610000	11.64010000	-1.26690000
H	2.47100000	10.48510000	-2.53710000
H	3.13900000	12.05420000	-2.93910000
C	0.76310000	14.13520000	-2.10790000
C	1.61290000	14.81690000	-3.19440000
H	1.20180000	14.64210000	-4.18950000
H	1.65990000	15.89570000	-3.04370000
H	2.63690000	14.44220000	-3.19510000
C	-0.66720000	14.70160000	-2.12820000
H	-1.13810000	14.57260000	-3.10260000
H	-1.29930000	14.20190000	-1.39240000
H	-0.67710000	15.76770000	-1.90050000
C	1.38950000	14.37610000	-0.72360000
H	0.82190000	13.86950000	0.05850000
H	2.41300000	14.00340000	-0.67680000
H	1.41640000	15.43760000	-0.47690000

trans-2 hexamer

C	-1.91380	8.89490	-0.26770
C	-0.37130	7.41890	2.05140
O	-1.55970	8.58280	-1.40270
O	-0.82260	7.70850	3.16100
N	-3.13970	8.64850	0.20750
N	-0.35870	6.16860	1.56840
H	-3.36010	8.99070	1.13520
H	0.02680	6.02370	0.64310
C	-0.92110	9.52740	0.71410
H	-1.46710	9.79600	1.61620
C	-0.34650	10.85600	0.16160
H	-0.99160	11.27720	-0.61140
H	-0.35130	11.58880	0.96720
C	1.37440	9.26040	1.73550
H	1.03620	9.84610	2.59090
C	1.08440	10.71630	-0.38080
H	1.06380	10.04450	-1.24080
C	2.04730	10.16690	0.69560
H	2.56740	10.97920	1.20540
C	-0.88170	4.99530	2.25020
H	-1.89140	5.20010	2.60830
H	-0.26400	4.77910	3.12300
C	-4.11030	7.77350	-0.43100
H	-4.16950	7.97870	-1.50140
H	-5.09190	7.99720	-0.01450

C	0.18360	8.50380	1.11030
H	0.54890	8.00570	0.20900
C	-0.89630	3.79830	1.28910
H	0.11450	3.61370	0.92230
H	-1.49620	4.05170	0.41460
C	-1.44630	2.51630	1.92930
H	-0.82540	2.23900	2.78190
H	-2.44440	2.70940	2.32100
C	-1.51180	1.34760	0.93580
H	-0.52040	1.16640	0.51880
H	-2.15120	1.62980	0.09960
C	-2.04170	0.05320	1.56980
H	-1.37650	-0.25590	2.37670
H	-3.01270	0.24650	2.02420
C	-2.18310	-1.08870	0.55400
H	-1.22160	-1.27240	0.07370
H	-2.86890	-0.77760	-0.23350
C	-2.69140	-2.39200	1.18740
H	-1.97820	-2.73400	1.93800
H	-3.62570	-2.19720	1.71250
C	-2.92160	-3.50250	0.15340
H	-1.99820	-3.68810	-0.39600
H	-3.65400	-3.15980	-0.57700
C	-3.40870	-4.81430	0.78480
H	-2.65180	-5.18880	1.47470
H	-4.30080	-4.61940	1.37890
C	-3.72880	-5.89060	-0.26120
H	-2.84870	-6.07460	-0.87840
H	-4.50380	-5.51710	-0.93010
C	-4.19390	-7.21120	0.36730
H	-3.39950	-7.61380	0.99670
H	-5.04190	-7.01950	1.02390
C	-4.59710	-8.25480	-0.68300
H	-5.40830	-7.85560	-1.29150
H	-3.76140	-8.43370	-1.36060
C	-5.03890	-9.58420	-0.05690
H	-4.21420	-10.00590	0.51890
H	-5.84670	-9.39920	0.65060
C	-5.50780	-10.60280	-1.10490
H	-6.34880	-10.18760	-1.66020
H	-4.71200	-10.77170	-1.83140
C	-5.92180	-11.94240	-0.48130
H	-5.07440	-12.37400	0.05250
H	-6.70190	-11.77590	0.26210
C	-6.42540	-12.94630	-1.52370
H	-7.30570	-12.56710	-2.04220
H	-6.69980	-13.89060	-1.05320
H	-5.66070	-13.15660	-2.27180
C	-3.73700	6.30380	-0.17500
H	-3.54640	6.16070	0.88940
H	-2.80180	6.06780	-0.68550
C	-4.82240	5.32070	-0.61970
H	-5.74960	5.55310	-0.09460
H	-5.02740	5.45060	-1.68270
C	-4.42450	3.86550	-0.34040
H	-4.10730	3.76620	0.69850
H	-3.56340	3.59860	-0.95470
C	-5.57360	2.88870	-0.60460
H	-6.41970	3.14870	0.03270
H	-5.91880	3.00280	-1.63220
C	-5.17590	1.42900	-0.35330
H	-4.79360	1.32110	0.66210
H	-4.36090	1.15270	-1.02250
C	-6.35670	0.47420	-0.55620
H	-7.15070	0.72830	0.14680
H	-6.77340	0.62410	-1.55210
C	-5.97020	-0.99960	-0.38310
H	-5.53490	-1.15280	0.60440

H	-5.19730	-1.26040	-1.10600
C	-7.17670	-1.92790	-0.56180
H	-7.92780	-1.69210	0.19290
H	-7.64310	-1.72990	-1.52680
C	-6.80840	-3.41390	-0.47330
H	-6.31700	-3.61490	0.47860
H	-6.08510	-3.65510	-1.25200
C	-8.03930	-4.31720	-0.61630
H	-8.55650	-4.07700	-1.54530
H	-8.74160	-4.09980	0.18920
C	-7.69000	-5.81050	-0.60330
H	-7.01700	-6.03110	-1.43150
H	-7.14670	-6.05230	0.31010
C	-8.93840	-6.69610	-0.70670
H	-9.50260	-6.42190	-1.59850
H	-9.59280	-6.49850	0.14320
C	-8.60400	-8.19230	-0.75860
H	-7.96870	-8.38970	-1.62160
H	-8.02410	-8.46800	0.12240
C	-9.86110	-9.06830	-0.83930
H	-10.45520	-8.77570	-1.70530
H	-10.48630	-8.89200	0.03670
C	-9.53510	-10.56190	-0.93460
H	-8.94360	-10.77900	-1.82320
H	-10.44710	-11.15620	-0.99120
H	-8.97150	-10.89890	-0.06470
O	2.99080	9.28450	0.12400
O	2.42020	8.38560	2.12240
C	3.40870	8.45440	1.15360
C	4.68050	9.03810	1.78730
H	5.02770	8.38700	2.58980
H	5.46500	9.12270	1.03490
H	4.48290	10.02600	2.20320
C	3.62880	7.04480	0.59630
H	3.98090	6.38250	1.38740
H	2.69210	6.64710	0.20620
H	4.37040	7.06790	-0.20270
O	1.52330	12.00260	-0.77240
Si	2.36090	12.34610	-2.19920
C	1.13850	12.20170	-3.64080
H	1.64530	12.23130	-4.60450
H	0.57230	11.27330	-3.59700
H	0.41200	13.01290	-3.62900
C	3.78530	11.11390	-2.38140
H	4.46880	11.16470	-1.53480
H	3.42550	10.08920	-2.43920
H	4.36220	11.30990	-3.28170
C	3.03310	14.13120	-2.05920
C	3.74590	14.51970	-3.36480
H	3.07430	14.45430	-4.22140
H	4.12300	15.54190	-3.32110
H	4.59670	13.86680	-3.56060
C	1.85990	15.09110	-1.79940
H	1.14680	15.08640	-2.62390
H	1.31760	14.81260	-0.89620
H	2.20520	16.11740	-1.67180
C	4.02630	14.20210	-0.88720
H	3.56030	13.88510	0.04510
H	4.88960	13.55780	-1.05520
H	4.39870	15.21650	-0.74180
C	-3.94680	10.33200	3.94960
C	-2.40080	9.16190	6.44810
O	-3.57360	10.32300	2.77800
O	-3.00870	9.38650	7.49700
N	-5.05240	9.69360	4.36430
N	-2.11950	7.92860	6.01350
H	-5.30420	9.78280	5.34050
H	-1.62330	7.83760	5.13500

C	-3.16160	11.11900	5.01240
H	-3.83430	11.28540	5.84990
C	-2.77530	12.54180	4.52140
H	-3.38920	12.85120	3.67390
H	-3.00980	13.24630	5.31720
C	-0.98980	11.25770	6.29210
H	-1.52950	11.73610	7.10810
C	-1.28670	12.70230	4.16900
H	-1.06050	12.05340	3.32150
C	-0.39390	12.32830	5.37360
H	-0.15940	13.20340	5.97960
C	-2.48230	6.70270	6.70100
H	-3.54250	6.72440	6.95830
H	-1.92050	6.63220	7.63350
C	-6.00650	9.02510	3.48600
H	-6.20500	9.67790	2.63400
H	-6.95680	8.92330	4.01090
C	-1.94270	10.31120	5.53600
H	-1.39910	9.89090	4.68740
C	-2.18390	5.50120	5.80020
H	-1.12960	5.50620	5.51900
H	-2.74990	5.60010	4.87360
C	-2.52740	4.16650	6.46780
H	-1.92820	4.04310	7.37050
H	-3.57020	4.17800	6.78480
C	-2.29620	2.97960	5.52800
H	-1.25400	2.96180	5.20680
H	-2.89450	3.11800	4.62800
C	-2.65680	1.64010	6.17790
H	-2.01410	1.46800	7.04170
H	-3.67940	1.68330	6.55280
C	-2.53020	0.46970	5.19890
H	-1.51300	0.42530	4.80810
H	-3.18310	0.65000	4.34560
C	-2.89270	-0.87300	5.84240
H	-2.20160	-1.08380	6.65900
H	-3.88730	-0.80660	6.28360
C	-2.86600	-2.02560	4.83430
H	-1.88170	-2.08280	4.36840
H	-3.57620	-1.81320	4.03600
C	-3.20910	-3.37500	5.47510
H	-2.46690	-3.61470	6.23710
H	-4.16880	-3.29930	5.98650
C	-3.27340	-4.50770	4.44530
H	-2.32480	-4.57150	3.91100
H	-4.03280	-4.26770	3.70200
C	-3.59530	-5.86530	5.08050
H	-2.81090	-6.12940	5.79040
H	-4.51970	-5.78590	5.65240
C	-3.73890	-6.97830	4.03590
H	-4.53440	-6.71300	3.34040
H	-2.82330	-7.04790	3.44750
C	-4.04860	-8.34260	4.66440
H	-3.23740	-8.62530	5.33620
H	-4.94620	-8.26160	5.27720
C	-4.24820	-9.44020	3.61130
H	-5.06590	-9.15750	2.94890
H	-3.35670	-9.51210	2.98710
C	-4.54870	-10.81030	4.23370
H	-3.72690	-11.10270	4.88800
H	-5.43680	-10.74200	4.86270
C	-4.76280	-11.89980	3.17810
H	-5.61170	-11.66410	2.53710
H	-4.96030	-12.86350	3.64800
H	-3.88410	-12.01270	2.54250
C	-5.52350	7.65310	2.97480
H	-4.48300	7.70700	2.65320
H	-6.10000	7.42240	2.07870

C	-5.70650	6.49090	3.96510
H	-5.06760	6.63180	4.83750
H	-6.73390	6.49760	4.32930
C	-5.42120	5.13100	3.30060
H	-4.36250	5.05660	3.05040
H	-5.95950	5.08290	2.35350
C	-5.84250	3.92320	4.15210
H	-5.25150	3.89010	5.06690
H	-6.88070	4.04820	4.45920
C	-5.71060	2.59600	3.38610
H	-4.67530	2.44760	3.08030
H	-6.29340	2.65840	2.46660
C	-6.18610	1.37860	4.19340
H	-5.56930	1.26750	5.08480
H	-7.20450	1.55240	4.54120
C	-6.15390	0.08040	3.37200
H	-5.14680	-0.08900	2.99190
H	-6.79520	0.19740	2.49830
C	-6.60990	-1.15080	4.16910
H	-5.94150	-1.30480	5.01610
H	-7.60050	-0.96600	4.58510
C	-6.65660	-2.42150	3.30790
H	-5.68190	-2.59100	2.85100
H	-7.35760	-2.26850	2.48730
C	-7.07040	-3.67100	4.09860
H	-8.02660	-3.48940	4.58990
H	-6.34510	-3.85780	4.89080
C	-7.19240	-4.91350	3.20490
H	-7.94830	-4.73000	2.44100
H	-6.25430	-5.07710	2.67490
C	-7.56090	-6.18130	3.98770
H	-8.47950	-6.00760	4.54910
H	-6.78420	-6.39360	4.72310
C	-7.75180	-7.39850	3.07180
H	-8.55370	-7.19200	2.36240
H	-6.85080	-7.55490	2.47910
C	-8.07760	-8.68070	3.84910
H	-8.96660	-8.52330	4.46070
H	-7.26450	-8.90710	4.53920
C	-8.30890	-9.88250	2.92700
H	-9.15720	-9.71170	2.26380
H	-8.51410	-10.78320	3.50560
H	-7.43510	-10.07870	2.30690
O	0.79310	11.70630	4.93150
O	0.15120	10.57770	6.78660
C	1.22470	10.90150	5.97270
C	2.25790	11.67170	6.80720
H	2.61890	11.04440	7.62250
H	3.10220	11.95960	6.18030
H	1.81070	12.57060	7.23170
C	1.79080	9.60530	5.38430
H	2.15530	8.96030	6.18380
H	1.01270	9.07330	4.83720
H	2.61330	9.82900	4.70430
O	-1.09300	14.05470	3.77940
Si	0.36730	14.77180	3.29130
C	1.33230	13.58480	2.18290
H	2.35250	13.93270	2.03220
H	1.39170	12.58480	2.60720
H	0.87230	13.50320	1.20110
C	1.42190	15.20060	4.80920
H	0.85510	15.75710	5.55340
H	1.81110	14.30690	5.29400
H	2.28130	15.81040	4.53370
C	-0.06660	16.35910	2.31590
C	1.22160	16.97710	1.74470
H	1.73880	16.28290	1.08180
H	1.00730	17.87850	1.16980

H	1.91750	17.25270	2.53750
C	-1.02660	15.99540	1.16960
H	-0.57970	15.27790	0.48210
H	-1.94690	15.55060	1.55060
H	-1.30350	16.87560	0.58880
C	-0.75310	17.36360	3.25500
H	-1.66940	16.94670	3.67180
H	-0.10660	17.64060	4.08810
H	-1.02140	18.28060	2.72970
C	-6.66490	11.47490	7.90870
C	-5.10990	10.79520	10.56560
O	-6.04310	11.34610	6.85690
O	-5.82960	10.89760	11.56000
N	-7.73280	10.73160	8.21850
N	-4.51940	9.64740	10.20920
H	-8.21380	10.94380	9.08380
H	-3.95200	9.64330	9.36990
C	-6.19930	12.47090	8.97770
H	-6.96060	12.50360	9.75410
C	-6.11550	13.91370	8.41890
H	-6.72150	14.02660	7.51830
H	-6.56410	14.58590	9.14940
C	-4.21760	13.17710	10.37690
H	-4.91270	13.58480	11.11190
C	-4.67680	14.37390	8.13260
H	-4.26990	13.75940	7.32780
C	-3.78090	14.26400	9.38710
H	-3.71850	15.22210	9.90480
C	-4.65560	8.38970	10.92370
H	-5.70850	8.20640	11.14220
H	-4.13210	8.45990	11.87810
C	-8.15400	9.54270	7.49290
H	-8.11330	9.71060	6.41530
H	-9.19900	9.35630	7.73800
C	-4.87010	11.99380	9.63210
H	-4.17950	11.67460	8.84800
C	-4.08910	7.24060	10.08250
H	-3.03890	7.43330	9.85840
H	-4.60740	7.20900	9.12380
C	-4.22410	5.88310	10.78220
H	-3.67080	5.90060	11.72190
H	-5.26970	5.71690	11.03930
C	-3.72730	4.72190	9.91290
H	-2.68780	4.89430	9.63150
H	-4.30040	4.70260	8.98610
C	-3.84880	3.36370	10.61690
H	-3.23530	3.36310	11.51850
H	-4.87880	3.21850	10.94070
C	-3.43690	2.19650	9.71170
H	-2.41540	2.34910	9.36150
H	-4.07170	2.19670	8.82600
C	-3.53920	0.83230	10.40820
H	-2.86510	0.80660	11.26500
H	-4.54710	0.70290	10.80080
C	-3.21570	-0.32970	9.46030
H	-2.21560	-0.19520	9.04670
H	-3.90560	-0.29920	8.61750
C	-3.30690	-1.70490	10.13610
H	-2.58120	-1.76350	10.94800
H	-4.29100	-1.82020	10.58910
C	-3.07070	-2.85350	9.14630
H	-2.09510	-2.73320	8.67390
H	-3.80960	-2.79030	8.34800
C	-3.15300	-4.24000	9.79910
H	-2.38400	-4.32880	10.56740
H	-4.11190	-4.34500	10.30550
C	-2.99570	-5.37340	8.77630
H	-3.77930	-5.28670	8.02410

H	-2.04810	-5.25970	8.24840
C	-3.05980	-6.76790	9.41300
H	-2.25420	-6.87310	10.14060
H	-3.99270	-6.87050	9.96670
C	-2.96320	-7.89070	8.37090
H	-3.78480	-7.79510	7.66130
H	-2.04410	-7.77570	7.79490
C	-2.99800	-9.28900	9.00130
H	-2.16470	-9.39740	9.69650
H	-3.90840	-9.40360	9.58980
C	-2.93160	-10.40650	7.95500
H	-3.77860	-10.35570	7.27150
H	-2.94890	-11.38680	8.43140
H	-2.01870	-10.33970	7.36260
C	-7.28110	8.33960	7.89180
H	-7.17760	8.31060	8.97720
H	-6.27290	8.46900	7.49430
C	-7.85750	7.00170	7.41850
H	-8.86110	6.88670	7.82960
H	-7.96890	7.00730	6.33410
C	-6.99300	5.80750	7.84750
H	-6.76760	5.88260	8.91200
H	-6.03650	5.84070	7.32390
C	-7.69000	4.46820	7.58460
H	-8.62760	4.43980	8.14130
H	-7.95980	4.39760	6.53080
C	-6.83190	3.25820	7.97650
H	-6.52120	3.34830	9.01780
H	-5.91990	3.24800	7.37870
C	-7.59180	1.94060	7.78670
H	-8.47530	1.94020	8.42610
H	-7.95920	1.88050	6.76220
C	-6.73980	0.70210	8.09130
H	-6.36020	0.75640	9.11180
H	-5.86900	0.68910	7.43550
C	-7.54080	-0.59270	7.90970
H	-8.38230	-0.59680	8.60320
H	-7.97310	-0.61070	6.90920
C	-6.69820	-1.85740	8.11590
H	-6.25490	-1.84730	9.11180
H	-5.87020	-1.86030	7.40700
C	-7.53210	-3.13210	7.93660
H	-8.01800	-3.11020	6.96120
H	-8.33420	-3.14660	8.67520
C	-6.69930	-4.41440	8.05580
H	-5.91470	-4.40590	7.29960
H	-6.19770	-4.43880	9.02340
C	-7.55630	-5.67590	7.88950
H	-8.08870	-5.62770	6.93940
H	-8.32040	-5.69990	8.66730
C	-6.72960	-6.96700	7.94160
H	-5.98110	-6.94670	7.15020
H	-6.18320	-7.01450	8.88380
C	-7.59810	-8.22260	7.78880
H	-8.16210	-8.16540	6.85760
H	-8.33430	-8.25820	8.59260
C	-6.77470	-9.51430	7.80000
H	-6.05600	-9.52880	6.98160
H	-7.41860	-10.38700	7.69030
H	-6.22260	-9.62310	8.73340
O	-2.48120	13.82350	9.05000
O	-3.00120	12.80900	11.00480
C	-1.96230	13.25670	10.20510
C	-1.16760	14.32090	10.97740
H	-0.74150	13.88300	11.88030
H	-0.35950	14.70530	10.35450
H	-1.81440	15.14910	11.26650
C	-1.10040	12.04890	9.82560

H	-0.66130	11.60920	10.72120
H	-1.71330	11.29160	9.33710
H	-0.29940	12.35490	9.15210
O	-4.73740	15.72990	7.73510
Si	-3.81490	16.42120	6.49930
C	-4.52900	15.83410	4.84440
H	-3.88060	16.09430	4.00890
H	-4.66990	14.75540	4.82670
H	-5.50360	16.28050	4.65190
C	-2.00910	15.88790	6.70270
H	-1.62510	16.15110	7.68760
H	-1.88910	14.81270	6.59030
H	-1.36910	16.36650	5.96560
C	-3.97060	18.31990	6.66230
C	-3.22550	18.99850	5.50060
H	-3.62570	18.68930	4.53440
H	-3.30830	20.08420	5.55750
H	-2.16370	18.75130	5.51140
C	-5.45890	18.70790	6.62930
H	-5.92600	18.42750	5.68520
H	-6.01270	18.21440	7.42830
H	-5.59290	19.78290	6.75360
C	-3.35540	18.75980	8.00140
H	-3.83750	18.25860	8.84030
H	-2.29150	18.52630	8.04900
H	-3.46180	19.83420	8.15430
C	-9.74350	11.94850	11.70150
C	-8.29010	11.36100	14.48490
O	-9.24380	12.20220	10.60690
O	-9.14180	11.27710	15.37040
N	-10.50120	10.86260	11.91720
N	-7.42120	10.37940	14.21330
H	-10.86190	10.71330	12.84900
H	-6.76120	10.51800	13.45770
C	-9.54880	12.90220	12.89260
H	-10.37260	12.71410	13.58390
C	-9.69490	14.39100	12.46580
H	-10.27780	14.48270	11.54770
H	-10.27460	14.91940	13.22380
C	-7.78570	13.83550	14.47500
H	-8.54740	14.02810	15.23240
C	-8.34600	15.11200	12.29490
H	-7.77680	14.60750	11.51200
C	-7.55500	15.09050	13.62270
H	-7.78620	15.96450	14.23200
C	-7.34170	9.13790	14.96530
H	-8.34310	8.72590	15.10390
H	-6.94160	9.35140	15.95770
C	-10.90430	9.91530	10.88460
H	-11.11910	10.45760	9.96180
H	-11.84450	9.45020	11.18200
C	-8.19610	12.62130	13.61360
H	-7.41710	12.47120	12.86480
C	-6.45710	8.11770	14.24240
H	-5.46260	8.53640	14.08220
H	-6.87360	7.91700	13.25500
C	-6.34410	6.80550	15.02800
H	-5.87550	6.99690	15.99420
H	-7.34490	6.42770	15.24110
C	-5.55040	5.73340	14.27350
H	-4.53750	6.08950	14.08190
H	-6.01060	5.57010	13.29950
C	-5.49700	4.40390	15.03630
H	-4.96790	4.54180	15.98010
H	-6.51100	4.09450	15.29290
C	-4.82420	3.29440	14.22030
H	-3.80040	3.58350	13.97980
H	-5.34650	3.18790	13.26980

C	-4.82300	1.94360	14.94680
H	-4.24330	2.02190	15.86730
H	-5.84190	1.69040	15.24240
C	-4.25780	0.82030	14.06950
H	-3.23730	1.06580	13.77280
H	-4.84050	0.76180	13.15060
C	-4.27640	-0.54700	14.76440
H	-3.65170	-0.51230	15.65770
H	-5.28940	-0.76960	15.10200
C	-3.79250	-1.66800	13.83600
H	-2.78480	-1.44190	13.48530
H	-4.42770	-1.69490	12.95120
C	-3.80350	-3.04780	14.50640
H	-3.13820	-3.03860	15.37060
H	-4.80340	-3.26000	14.88670
C	-3.37910	-4.16090	13.53920
H	-4.05760	-4.17010	12.68670
H	-2.38780	-3.93990	13.14160
C	-3.36970	-5.54880	14.19310
H	-2.67170	-5.55070	15.03110
H	-4.35470	-5.76110	14.61060
C	-2.98580	-6.65570	13.20140
H	-3.69310	-6.65640	12.37250
H	-2.00780	-6.43590	12.77120
C	-2.95940	-8.04750	13.84740
H	-2.24670	-8.05410	14.67290
H	-3.93530	-8.27060	14.28020
C	-2.58700	-9.14960	12.85010
H	-3.31170	-9.20620	12.03860
H	-2.56060	-10.12380	13.33870
H	-1.60530	-8.97030	12.41090
C	-9.84030	8.83850	10.61120
H	-8.86700	9.30150	10.44670
H	-10.10650	8.34870	9.67440
C	-9.72630	7.76150	11.70140
H	-9.32530	8.19360	12.61890
H	-10.72480	7.39480	11.94200
C	-8.85630	6.57940	11.24030
H	-7.82330	6.90500	11.11430
H	-9.19480	6.25820	10.25450
C	-8.91260	5.37050	12.18610
H	-8.47210	5.62990	13.14860
H	-9.95510	5.11780	12.38340
C	-8.20750	4.14120	11.59170
H	-7.15540	4.36570	11.41840
H	-8.63760	3.92620	10.61280
C	-8.32720	2.88780	12.47070
H	-7.83040	3.05770	13.42610
H	-9.37800	2.70400	12.69810
C	-7.73650	1.64700	11.78530
H	-6.68660	1.82160	11.55140
H	-8.24120	1.49600	10.83080
C	-7.86510	0.36890	12.62600
H	-7.30730	0.48230	13.55590
H	-8.90850	0.22190	12.90740
C	-7.36660	-0.86800	11.86560
H	-6.33090	-0.71750	11.56160
H	-7.94300	-0.97520	10.94660
C	-7.47510	-2.16490	12.67890
H	-8.50200	-2.29540	13.02220
H	-6.85730	-2.08870	13.57410
C	-7.05370	-3.39230	11.85890
H	-7.69430	-3.47140	10.98070
H	-6.03910	-3.25100	11.48660
C	-7.12660	-4.70210	12.65470
H	-8.13380	-4.83170	13.05250
H	-6.46000	-4.64280	13.51550
C	-6.75700	-5.92090	11.79780

H	-7.44560	-5.99060	10.95540
H	-5.76420	-5.77820	11.37150
C	-6.78800	-7.23560	12.58810
H	-7.77710	-7.37930	13.02450
H	-6.08890	-7.17760	13.42260
C	-6.43930	-8.44800	11.71870
H	-7.16140	-8.57340	10.91190
H	-6.43740	-9.36400	12.30960
H	-5.45250	-8.34010	11.26990
O	-6.16920	15.02200	13.36450
O	-6.53330	13.62810	15.10730
C	-5.59330	14.42040	14.46990
C	-5.11830	15.50720	15.44570
H	-4.64790	15.04610	16.31440
H	-4.39410	16.15720	14.95400
H	-5.96000	16.11050	15.78560
C	-4.44980	13.51950	13.99540
H	-3.96720	13.04640	14.85070
H	-4.83850	12.73920	13.34190
H	-3.71110	14.10630	13.44890
O	-8.62810	16.43680	11.86860
Si	-7.50460	17.67660	11.57530
C	-5.98590	16.99370	10.67930
H	-5.16560	17.70900	10.69960
H	-5.62280	16.07380	11.13190
H	-6.20270	16.78600	9.63380
C	-6.97850	18.45820	13.22160
H	-7.84110	18.70840	13.83820
H	-6.34210	17.79050	13.79990
H	-6.41390	19.37600	13.06270
C	-8.37130	18.98130	10.47950
C	-7.36170	20.07390	10.08590
H	-6.51890	19.65830	9.53240
H	-7.82520	20.83150	9.45340
H	-6.95980	20.58150	10.96310
C	-8.91250	18.29280	9.21420
H	-8.11280	17.83590	8.63160
H	-9.62350	17.50460	9.46540
H	-9.42520	19.00250	8.56430
C	-9.53820	19.60380	11.26500
H	-10.25580	18.84360	11.57640
H	-9.18860	20.11270	12.16380
H	-10.07580	20.33660	10.66270
C	-0.08190	5.04630	-9.18100
C	1.23590	2.99290	-7.20550
O	-0.05510	4.61740	-10.33430
O	1.15550	3.30840	-6.01750
N	-1.21520	5.28700	-8.50890
N	0.67390	1.89000	-7.71840
H	-1.13350	5.67820	-7.57840
H	0.79560	1.73140	-8.70800
C	1.22260	5.24790	-8.39990
H	0.96980	5.62290	-7.40980
C	2.10770	6.33550	-9.05720
H	1.51650	6.99810	-9.69170
H	2.50610	6.97350	-8.27010
C	3.43740	4.13700	-7.85390
H	3.49730	4.71660	-6.93260
C	3.26990	5.74530	-9.86970
H	2.85080	5.15090	-10.68410
C	4.17840	4.85960	-8.99000
H	5.01250	5.42890	-8.57920
C	-0.14740	0.92710	-7.00370
H	-0.94030	1.44860	-6.46630
H	0.46250	0.40600	-6.26450
C	-2.54440	4.88820	-8.95000
H	-2.69150	5.14900	-9.99950
H	-3.27680	5.45890	-8.37940

C	1.96140	3.88870	-8.22560
H	1.96430	3.36110	-9.18200
C	-0.75000	-0.06540	-8.00970
H	0.05520	-0.55860	-8.55620
H	-1.33100	0.48380	-8.75130
C	-1.64230	-1.13240	-7.36300
H	-1.05860	-1.70240	-6.63950
H	-2.44250	-0.64700	-6.80550
C	-2.25240	-2.08720	-8.40110
H	-1.45420	-2.55020	-8.98260
H	-2.85480	-1.51550	-9.10760
C	-3.11550	-3.18510	-7.76450
H	-2.50160	-3.77480	-7.08310
H	-3.89670	-2.72640	-7.15930
C	-3.76200	-4.11360	-8.80280
H	-2.98720	-4.55240	-9.43280
H	-4.40140	-3.52780	-9.46370
C	-4.58370	-5.23560	-8.15190
H	-3.93010	-5.84020	-7.52240
H	-5.33210	-4.80010	-7.49040
C	-5.28330	-6.14000	-9.17600
H	-4.54360	-6.55810	-9.85980
H	-5.96130	-5.54050	-9.78400
C	-6.06270	-7.28230	-8.50790
H	-5.37270	-7.90070	-7.93290
H	-6.77350	-6.86820	-7.79300
C	-6.81610	-8.16130	-9.51520
H	-6.11370	-8.56010	-10.24800
H	-7.52540	-7.54700	-10.07060
C	-7.55950	-9.32190	-8.83790
H	-6.84140	-9.95470	-8.31530
H	-8.23380	-8.92870	-8.07720
C	-8.36080	-10.17260	-9.83220
H	-9.09380	-9.54270	-10.33710
H	-7.69380	-10.55120	-10.60770
C	-9.07490	-11.35120	-9.15550
H	-8.33610	-11.99760	-8.68030
H	-9.71660	-10.97890	-8.35680
C	-9.91650	-12.17410	-10.14030
H	-10.66520	-11.52930	-10.60190
H	-9.28020	-12.53540	-10.94920
C	-10.61120	-13.36630	-9.46760
H	-9.86190	-14.02680	-9.02990
H	-11.22960	-13.01350	-8.64210
C	-11.48190	-14.16650	-10.44200
H	-12.26860	-13.54460	-10.87010
H	-11.96040	-15.00540	-9.93610
H	-10.88750	-14.56850	-11.26300
C	-2.74820	3.37960	-8.72820
H	-2.48310	3.12470	-7.70190
H	-2.05750	2.82590	-9.36550
C	-4.17950	2.90670	-9.00810
H	-4.86910	3.42390	-8.34010
H	-4.46580	3.18180	-10.02400
C	-4.32060	1.38850	-8.82760
H	-3.95970	1.10410	-7.83870
H	-3.67620	0.88160	-9.54660
C	-5.76250	0.89400	-8.99920
H	-6.39950	1.36670	-8.25070
H	-6.14340	1.20720	-9.97210
C	-5.86750	-0.63250	-8.87780
H	-5.44160	-0.95220	-7.92640
H	-5.26300	-1.09740	-9.65730
C	-7.31210	-1.13870	-8.98360
H	-7.90750	-0.70530	-8.17910
H	-7.75380	-0.78870	-9.91740
C	-7.39940	-2.66980	-8.92270
H	-6.91990	-3.02530	-8.01060

H	-6.83620	-3.09620	-9.75350
C	-8.84530	-3.18160	-8.97100
H	-9.39900	-2.78230	-8.12040
H	-9.33750	-2.79910	-9.86610
C	-8.92490	-4.71430	-8.96350
H	-8.39960	-5.10090	-8.09010
H	-8.40300	-5.10730	-9.83650
C	-10.37040	-5.22980	-8.95830
H	-10.90590	-4.82040	-9.81590
H	-10.88310	-4.85970	-8.06960
C	-10.44800	-6.76230	-8.99550
H	-9.96430	-7.12710	-9.90210
H	-9.88500	-7.17450	-8.15820
C	-11.89190	-7.28030	-8.94360
H	-12.46220	-6.85000	-9.76780
H	-12.36750	-6.93350	-8.02530
C	-11.97150	-8.81150	-9.01530
H	-11.51720	-9.15470	-9.94530
H	-11.38250	-9.24360	-8.20650
C	-13.41360	-9.33020	-8.93050
H	-14.00870	-8.89070	-9.73180
H	-13.86600	-9.00110	-7.99430
C	-13.49680	-10.85750	-9.02200
H	-13.09260	-11.21830	-9.96790
H	-14.53090	-11.19560	-8.95290
H	-12.93830	-11.33220	-8.21560
O	4.68090	3.78240	-9.74810
O	4.15000	2.91870	-7.72220
C	4.93860	2.76010	-8.85140
C	6.41460	2.81780	-8.43150
H	6.63500	2.00720	-7.73660
H	7.05510	2.71780	-9.30800
H	6.63410	3.76620	-7.94080
C	4.56560	1.43300	-9.51890
H	4.74380	0.60610	-8.83110
H	3.51160	1.43950	-9.79710
H	5.16640	1.28540	-10.41670
O	4.03340	6.81280	-10.39370
Si	4.56880	6.91050	-11.99360
C	3.04660	7.03880	-13.11410
H	3.32700	7.12350	-14.16330
H	2.40560	6.16280	-13.01740
H	2.44200	7.91020	-12.86510
C	5.55710	5.34950	-12.41250
H	6.37790	5.19910	-11.71200
H	4.93040	4.45930	-12.37020
H	5.98370	5.40050	-13.41340
C	5.66160	8.47190	-12.14180
C	6.11940	8.64350	-13.60010
H	5.26970	8.75730	-14.27400
H	6.74880	9.52640	-13.71580
H	6.69640	7.78320	-13.94070
C	4.84410	9.69810	-11.70170
H	3.97030	9.84550	-12.33680
H	4.48900	9.58660	-10.67710
H	5.43950	10.61060	-11.74500
C	6.88680	8.31520	-11.22540
H	6.58730	8.16300	-10.18850
H	7.49820	7.46110	-11.51810
H	7.52290	9.20020	-11.25710
C	-0.60670	6.90630	-4.63850
C	0.85930	5.09230	-2.51620
O	-0.50290	6.87360	-5.86370
O	0.55350	5.43590	-1.37230
N	-1.75680	6.63670	-3.99890
N	0.60860	3.87670	-3.01290
H	-1.75800	6.71440	-2.99040
H	0.89050	3.69170	-3.96820

C	0.59780	7.29400	-3.76170
H	0.19980	7.62740	-2.80600
C	1.37220	8.51850	-4.32590
H	0.76330	9.08370	-5.03330
H	1.56310	9.20360	-3.50180
C	2.87520	6.57100	-2.94050
H	2.70770	7.15650	-2.03720
C	2.72360	8.16250	-4.97180
H	2.53180	7.53480	-5.84340
C	3.63310	7.41250	-3.97190
H	4.28900	8.10120	-3.43930
C	-0.07820	2.80650	-2.31580
H	-0.98930	3.18910	-1.85300
H	0.56090	2.42470	-1.51840
C	-3.04190	6.40630	-4.65150
H	-3.16800	7.16230	-5.42880
H	-3.84130	6.58540	-3.93200
C	1.52320	6.07610	-3.49160
H	1.71260	5.55710	-4.43380
C	-0.41870	1.70010	-3.31770
H	0.49640	1.34600	-3.79500
H	-1.03790	2.11660	-4.11270
C	-1.14540	0.51730	-2.67460
H	-0.51240	0.07670	-1.90370
H	-2.04630	0.87240	-2.17430
C	-1.52190	-0.54910	-3.70800
H	-0.62330	-0.90050	-4.21650
H	-2.15400	-0.09850	-4.47280
C	-2.25080	-1.73920	-3.07860
H	-1.59880	-2.21550	-2.34590
H	-3.12430	-1.38200	-2.53320
C	-2.69330	-2.77060	-4.12100
H	-1.82650	-3.12030	-4.68280
H	-3.35760	-2.29160	-4.83960
C	-3.40560	-3.96780	-3.48430
H	-2.72220	-4.47070	-2.79980
H	-4.24310	-3.61310	-2.88360
C	-3.91840	-4.96910	-4.52410
H	-3.08990	-5.30920	-5.14630
H	-4.62160	-4.46770	-5.18850
C	-4.59940	-6.17900	-3.87580
H	-3.87990	-6.70260	-3.24580
H	-5.39670	-5.83480	-3.21700
C	-5.17960	-7.15230	-4.90750
H	-4.39140	-7.48050	-5.58590
H	-5.91730	-6.63100	-5.51700
C	-5.83000	-8.37520	-4.25070
H	-5.08010	-8.91720	-3.67380
H	-6.58700	-8.04270	-3.54050
C	-6.47180	-9.32150	-5.27200
H	-7.23190	-8.77990	-5.83470
H	-5.72130	-9.64300	-5.99500
C	-7.10540	-10.55170	-4.61090
H	-6.33780	-11.11000	-4.07390
H	-7.82980	-10.22570	-3.86480
C	-7.79470	-11.47500	-5.62380
H	-8.56370	-10.91560	-6.15610
H	-7.07140	-11.79530	-6.37460
C	-8.42610	-12.70780	-4.96300
H	-7.65630	-13.27640	-4.44030
H	-9.14500	-12.39190	-4.20600
C	-9.12610	-13.62220	-5.97370
H	-9.93400	-13.09900	-6.48410
H	-9.55620	-14.49270	-5.47820
H	-8.42840	-13.98090	-6.73090
C	-3.18220	5.00430	-5.28030
H	-2.25080	4.70080	-5.75980
H	-3.91350	5.08830	-6.08440

C	-3.66740	3.90140	-4.32140
H	-2.91290	3.70290	-3.55960
H	-4.55410	4.25410	-3.79420
C	-4.02120	2.60880	-5.08250
H	-3.11730	2.17750	-5.51310
H	-4.66500	2.86450	-5.92450
C	-4.74510	1.55030	-4.23420
H	-4.08750	1.20010	-3.43940
H	-5.60640	2.00640	-3.74640
C	-5.22920	0.36060	-5.08170
H	-4.37890	-0.10300	-5.58050
H	-5.88170	0.73060	-5.87320
C	-5.98480	-0.70350	-4.27060
H	-5.32440	-1.11710	-3.50910
H	-6.81420	-0.23460	-3.74140
C	-6.53210	-1.83870	-5.15030
H	-5.71650	-2.28890	-5.71520
H	-7.22080	-1.42170	-5.88550
C	-7.25130	-2.92980	-4.34300
H	-6.55420	-3.37350	-3.63230
H	-8.04970	-2.47860	-3.75340
C	-7.84610	-4.03140	-5.23230
H	-7.06350	-4.45720	-5.85980
H	-8.57720	-3.58930	-5.90950
C	-8.51350	-5.15260	-4.42270
H	-9.27440	-4.72550	-3.76890
H	-7.77400	-5.61990	-3.77240
C	-9.15960	-6.22180	-5.31430
H	-9.92830	-5.75680	-5.93210
H	-8.41490	-6.62470	-6.00060
C	-9.78050	-7.36980	-4.50610
H	-10.50270	-6.96580	-3.79580
H	-9.00550	-7.85750	-3.91450
C	-10.47550	-8.40830	-5.39660
H	-11.27520	-7.92360	-5.95750
H	-9.76840	-8.78780	-6.13430
C	-11.05430	-9.58160	-4.59440
H	-11.75070	-9.20640	-3.84380
H	-10.25430	-10.08200	-4.04880
C	-11.77450	-10.60090	-5.48230
H	-12.63190	-10.14870	-5.98070
H	-12.13790	-11.44300	-4.89310
H	-11.11010	-10.99460	-6.25100
O	4.39740	6.43190	-4.64000
O	3.76540	5.50030	-2.67580
C	4.70270	5.46550	-3.69570
C	6.08650	5.76380	-3.10100
H	6.34690	5.00220	-2.36570
H	6.83800	5.76610	-3.89090
H	6.08840	6.73660	-2.60910
C	4.63410	4.08910	-4.36450
H	4.89250	3.31150	-3.64550
H	3.62440	3.90140	-4.72900
H	5.32940	4.04350	-5.20320
O	3.32430	9.37620	-5.40110
Si	4.80040	9.53630	-6.22690
C	4.98620	8.15080	-7.49430
H	6.00590	8.09300	-7.87120
H	4.74290	7.17990	-7.06890
H	4.33740	8.31550	-8.35130
C	6.24830	9.44740	-5.00380
H	6.13260	10.15020	-4.18110
H	6.35010	8.45270	-4.57320
H	7.19290	9.67830	-5.49460
C	4.78200	11.22480	-7.12400
C	6.02600	11.34160	-8.02200
H	6.04960	10.55480	-8.77700
H	6.04680	12.29690	-8.54740

H	6.94580	11.26710	-7.44120
C	3.50770	11.32110	-7.98110
H	3.46320	10.52720	-8.72610
H	2.61010	11.24020	-7.36640
H	3.45700	12.27180	-8.51240
C	4.78150	12.35370	-6.08200
H	3.90870	12.28500	-5.43330
H	5.66810	12.31600	-5.44860
H	4.75960	13.33480	-6.55710

trans-2 octamer

C	-16.35200	10.98410	19.03190
C	-15.35360	11.45550	22.00370
O	-15.72120	11.28520	18.02080
O	-16.20600	11.12540	22.82830
N	-16.63980	9.72020	19.36630
N	-14.13670	10.89960	21.94360
H	-17.17140	9.57560	20.21300
H	-13.50280	11.21530	21.21960
C	-16.83410	12.06030	20.01750
H	-17.61220	11.60330	20.63260
C	-17.52200	13.24920	19.29390
H	-17.89410	12.95230	18.31180
H	-18.40800	13.53800	19.86110
C	-16.01390	13.87460	21.59200
H	-16.94770	13.79450	22.15080
C	-16.61360	14.48160	19.15160
H	-15.76970	14.22230	18.50990
C	-16.10620	14.97340	20.52690
H	-16.73140	15.78600	20.90070
C	-13.66850	9.84250	22.82400
H	-14.43700	9.07270	22.91500
H	-13.50230	10.25370	23.82070
C	-16.13580	8.52720	18.70360
H	-16.04510	8.67980	17.62660
H	-16.87640	7.73990	18.84660
C	-15.66430	12.51850	20.94060
H	-14.76690	12.66240	20.33420
C	-12.37660	9.22840	22.27300
H	-11.60870	9.99940	22.19470
H	-12.55250	8.86700	21.25950
C	-11.86200	8.07560	23.14390
H	-11.64250	8.44580	24.14620
H	-12.64810	7.32750	23.25520
C	-10.61260	7.41160	22.55320
H	-9.82500	8.15630	22.43190
H	-10.84420	7.04210	21.55430
C	-10.09520	6.25340	23.41660
H	-9.80720	6.62960	24.39910
H	-10.89970	5.53570	23.58260
C	-8.90510	5.53720	22.76700
H	-8.10370	6.25320	22.58100
H	-9.21130	5.15750	21.79260
C	-8.36770	4.37650	23.61510
H	-7.99660	4.75840	24.56700
H	-9.18240	3.69080	23.85160
C	-7.25330	3.60940	22.89220
H	-6.44090	4.29280	22.64200
H	-7.64170	3.23450	21.94580
C	-6.69950	2.43420	23.70880
H	-6.24490	2.80730	24.62730
H	-7.52010	1.78230	24.01120
C	-5.67110	1.62010	22.91200
H	-4.85220	2.26950	22.60000
H	-6.13990	1.25740	21.99770

C	-5.10700	0.42720	23.69500
H	-4.58000	0.78700	24.57950
H	-5.92890	-0.19280	24.05520
C	-4.16170	-0.42760	22.83910
H	-4.69960	-0.78240	21.96020
H	-3.34320	0.19110	22.46890
C	-3.58850	-1.63050	23.59960
H	-3.00800	-1.27780	24.45300
H	-4.40630	-2.22560	24.00760
C	-2.70790	-2.51730	22.70750
H	-3.29540	-2.87210	21.86060
H	-1.89500	-1.92220	22.28950
C	-2.12520	-3.72100	23.45980
H	-1.51300	-3.37150	24.29190
H	-2.93460	-4.30570	23.89800
C	-1.28100	-4.62510	22.55470
H	-1.87510	-5.02450	21.73310
H	-0.87930	-5.47010	23.11430
H	-0.44030	-4.07980	22.12500
C	-14.78900	8.10390	19.31200
H	-14.85020	8.17140	20.39930
H	-14.00710	8.80060	19.00460
C	-14.40130	6.66930	18.93340
H	-15.20820	6.00190	19.23900
H	-14.32070	6.57470	17.85040
C	-13.09480	6.20970	19.59590
H	-13.10710	6.48170	20.65190
H	-12.25070	6.73560	19.14860
C	-12.89770	4.69250	19.48360
H	-13.73800	4.19090	19.96530
H	-12.92500	4.39250	18.43610
C	-11.59090	4.20500	20.12220
H	-11.52780	4.56560	21.14930
H	-10.74280	4.63380	19.58820
C	-11.49480	2.67370	20.11830
H	-12.31440	2.26250	20.70920
H	-11.63630	2.30270	19.10290
C	-10.16160	2.14840	20.66480
H	-9.99990	2.53270	21.67230
H	-9.34460	2.52780	20.05100
C	-10.12370	0.61440	20.68960
H	-10.89740	0.24600	21.36440
H	-10.37400	0.23040	19.70030
C	-8.76270	0.04970	21.11500
H	-8.49780	0.43130	22.10140
H	-7.99500	0.40400	20.42710
C	-8.76300	-1.48500	21.14070
H	-9.10740	-1.86240	20.17740
H	-9.48370	-1.83390	21.88130
C	-7.38310	-2.08050	21.44710
H	-6.66910	-1.73610	20.69920
H	-7.02850	-1.71020	22.40930
C	-7.40660	-3.61510	21.46490
H	-7.82260	-3.98170	20.52600
H	-8.07850	-3.95820	22.25270
C	-6.01610	-4.22870	21.67280
H	-5.35010	-3.88470	20.88170
H	-5.59380	-3.86940	22.61150
C	-6.04980	-5.76310	21.68130
H	-6.50980	-6.12440	20.76140
H	-6.68310	-6.11150	22.49790
C	-4.65580	-6.38220	21.82150
H	-4.00440	-6.07010	21.00580
H	-4.71000	-7.47080	21.80520
H	-4.18560	-6.08680	22.75930
O	-14.75860	15.39560	20.45730
O	-14.98500	14.34580	22.44650
C	-14.26660	15.30430	21.75130

C	-14.43630	16.66080	22.45300
H	-14.04030	16.60620	23.46730
H	-13.89660	17.43250	21.90360
H	-15.48880	16.93790	22.50690
C	-12.80260	14.85730	21.70460
H	-12.39400	14.81700	22.71440
H	-12.72880	13.86210	21.26660
H	-12.21430	15.55890	21.11310
O	-17.38570	15.50780	18.55750
Si	-16.80120	16.64070	17.44620
C	-16.62270	15.77210	15.77060
H	-16.08210	16.38610	15.05170
H	-16.09140	14.82710	15.86140
H	-17.59460	15.54230	15.33620
C	-15.14170	17.31700	18.05950
H	-15.22670	17.72980	19.06400
H	-14.37470	16.54660	18.09320
H	-14.77460	18.11110	17.41510
C	-18.08490	18.05290	17.33680
C	-17.61150	19.09790	16.31210
H	-17.47750	18.65590	15.32410
H	-18.33230	19.91010	16.21290
H	-16.65990	19.54120	16.60590
C	-19.44180	17.47250	16.90210
H	-19.38590	17.01930	15.91240
H	-19.78500	16.70440	17.59630
H	-20.20940	18.24590	16.86250
C	-18.22600	18.70710	18.72210
H	-18.53680	17.98010	19.47360
H	-17.28400	19.14190	19.05710
H	-18.96830	19.50580	18.71030
C	-12.79220	11.79360	15.62920
C	-11.54140	11.63440	18.54710
O	-12.16380	12.03490	14.60070
O	-12.39260	11.46280	19.42230
N	-13.45090	10.64140	15.82390
N	-10.53130	10.78200	18.33380
H	-13.96530	10.53570	16.68900
H	-9.88320	10.99270	17.58430
C	-12.88390	12.84100	16.74900
H	-13.71770	12.55570	17.38670
C	-13.25980	14.24380	16.19580
H	-13.71720	14.17220	15.20760
H	-14.03240	14.66000	16.83950
C	-11.56870	14.16400	18.44410
H	-12.47180	14.22220	19.04990
C	-12.09030	15.24280	16.15310
H	-11.34030	14.87120	15.45330
C	-11.46720	15.40860	17.55720
H	-11.92210	16.24050	18.09460
C	-10.28160	9.58250	19.11610
H	-11.19030	8.98080	19.16560
H	-10.02560	9.86890	20.13720
C	-13.61140	9.59920	14.81620
H	-13.88430	10.06780	13.86890
H	-14.45900	8.97260	15.09540
C	-11.59770	12.86110	17.62030
H	-10.72030	12.85230	16.97090
C	-9.14350	8.76970	18.48830
H	-8.25700	9.39770	18.38820
H	-9.42830	8.47670	17.47730
C	-8.78990	7.51940	19.30400
H	-8.44890	7.81460	20.29690
H	-9.68590	6.91620	19.45060
C	-7.71290	6.66890	18.62050
H	-6.81700	7.27000	18.45980
H	-8.06790	6.37640	17.63290
C	-7.34880	5.41210	19.42110

H	-6.91010	5.69940	20.37720
H	-8.25510	4.85180	19.65010
C	-6.37990	4.50360	18.65660
H	-5.47260	5.05730	18.41220
H	-6.83620	4.22440	17.70750
C	-6.01190	3.23290	19.43290
H	-5.47690	3.50200	20.34410
H	-6.92150	2.72000	19.74450
C	-5.16130	2.27350	18.59380
H	-4.25820	2.78390	18.25750
H	-5.71790	2.00540	17.69640
C	-4.77580	0.99570	19.34950
H	-4.15420	1.25270	20.20770
H	-5.67330	0.52030	19.74490
C	-4.03530	-0.00160	18.45160
H	-3.14920	0.47470	18.03050
H	-4.67680	-0.26110	17.61000
C	-3.62480	-1.28260	19.18860
H	-2.93370	-1.03310	19.99420
H	-4.50080	-1.73100	19.65720
C	-2.97640	-2.30600	18.24860
H	-3.68100	-2.55580	17.45580
H	-2.11020	-1.85640	17.76160
C	-2.54780	-3.59100	18.96830
H	-1.81400	-3.34850	19.73770
H	-3.40550	-4.02320	19.48330
C	-1.95870	-4.62820	18.00310
H	-2.69560	-4.86290	17.23530
H	-1.10120	-4.19700	17.48500
C	-1.53310	-5.92210	18.70980
H	-0.79490	-5.69350	19.47940
H	-2.39030	-6.35910	19.22230
C	-0.94880	-6.95490	17.74050
H	-1.68100	-7.24560	16.98780
H	-0.64360	-7.85690	18.27110
H	-0.07400	-6.56070	17.22250
C	-12.35170	8.73470	14.61600
H	-11.47470	9.36300	14.45610
H	-12.48800	8.17570	13.68990
C	-12.07880	7.73300	15.75040
H	-11.82950	8.26260	16.67070
H	-12.99240	7.17390	15.95240
C	-10.95970	6.74380	15.37920
H	-10.01330	7.27580	15.27740
H	-11.17900	6.32290	14.39770
C	-10.80420	5.58430	16.37500
H	-10.50190	5.96840	17.34870
H	-11.77410	5.10820	16.51840
C	-9.80040	4.52710	15.88510
H	-8.82100	4.98370	15.74390
H	-10.11590	4.17140	14.90400
C	-9.67170	3.32280	16.82970
H	-9.29050	3.65340	17.79530
H	-10.66110	2.90400	17.01390
C	-8.76070	2.22560	16.25690
H	-7.77370	2.63820	16.04790
H	-9.16430	1.89540	15.29960
C	-8.61530	1.01040	17.18430
H	-8.15080	1.31950	18.12050
H	-9.60450	0.62960	17.43970
C	-7.79080	-0.11520	16.54150
H	-6.81250	0.26740	16.25040
H	-8.28190	-0.43470	15.62200
C	-7.60410	-1.33110	17.45950
H	-8.57880	-1.68650	17.79520
H	-7.05840	-1.03070	18.35410
C	-6.86050	-2.47720	16.75780
H	-7.43210	-2.79360	15.88480

H	-5.90300	-2.11560	16.38360
C	-6.62130	-3.68750	17.66990
H	-7.57320	-4.03070	18.07630
H	-6.01170	-3.38440	18.52150
C	-5.93650	-4.84660	16.93120
H	-6.56510	-5.16780	16.09990
H	-5.00230	-4.49720	16.49300
C	-5.64930	-6.04530	17.84420
H	-6.57880	-6.39220	18.29670
H	-5.00310	-5.73260	18.66460
C	-4.98880	-7.20830	17.09650
H	-5.63370	-7.58350	16.30140
H	-4.77830	-8.03610	17.77380
H	-4.04600	-6.90190	16.64510
O	-10.06970	15.58550	17.46500
O	-10.41600	14.25350	19.26380
C	-9.54370	15.15300	18.67220
C	-9.37320	16.35950	19.60650
H	-8.94800	16.03620	20.55690
H	-8.70650	17.09160	19.15020
H	-10.33630	16.83180	19.79970
C	-8.22120	14.42880	18.40180
H	-7.77250	14.10830	19.34210
H	-8.39800	13.54680	17.78630
H	-7.52750	15.09240	17.88480
O	-12.61670	16.46810	15.65910
Si	-11.79250	17.94110	15.46010
C	-10.07500	17.63730	14.73440
H	-9.49220	18.55620	14.72080
H	-9.51280	16.90190	15.30660
H	-10.13730	17.28540	13.70740
C	-11.61480	18.82110	17.13180
H	-12.56280	18.87740	17.66360
H	-10.90340	18.31270	17.78030
H	-11.25280	19.84060	17.00620
C	-12.81520	19.01830	14.25230
C	-12.09240	20.35550	14.01500
H	-11.09430	20.20340	13.60540
H	-12.64150	20.98270	13.31200
H	-11.98380	20.92150	14.94040
C	-12.97080	18.26570	12.91930
H	-12.00580	18.07700	12.44860
H	-13.45850	17.30080	13.06400
H	-13.57250	18.83470	12.21010
C	-14.20390	19.28410	14.85390
H	-14.73680	18.35180	15.03860
H	-14.13270	19.81970	15.80090
H	-14.82000	19.88490	14.18440
C	-1.90210	8.90570	-0.04420
C	-0.24950	7.38550	2.17450
O	-1.60190	8.61290	-1.19970
O	-0.64690	7.65990	3.30840
N	-3.10540	8.65380	0.48310
N	-0.25840	6.14190	1.67380
H	-3.28190	8.98090	1.42520
H	0.08280	6.01110	0.72930
C	-0.86240	9.51690	0.90160
H	-1.36410	9.76820	1.83400
C	-0.31160	10.85420	0.34640
H	-0.99680	11.29540	-0.37960
H	-0.26540	11.56940	1.16630
C	1.48510	9.22520	1.79290
H	1.19760	9.79800	2.67520
C	1.08470	10.71610	-0.27980
H	1.01090	10.05670	-1.14640
C	2.10390	10.14560	0.73180
H	2.65600	10.94720	1.22440
C	-0.74380	4.95660	2.36250

H	-1.73680	5.15120	2.76970
H	-0.08550	4.73170	3.20280
C	-4.10200	7.78450	-0.12230
H	-4.20720	8.00010	-1.18710
H	-5.06550	8.00210	0.33750
C	0.25800	8.48350	1.22210
H	0.57190	7.99830	0.29470
C	-0.79690	3.77270	1.38610
H	0.19690	3.59820	0.97090
H	-1.43740	4.03470	0.54350
C	-1.31100	2.47910	2.03290
H	-0.64960	2.19370	2.85180
H	-2.29020	2.66230	2.47360
C	-1.41910	1.32210	1.02910
H	-0.44760	1.14790	0.56520
H	-2.09710	1.61210	0.22660
C	-1.91700	0.02010	1.67360
H	-1.21460	-0.29460	2.44610
H	-2.86620	0.20730	2.17400
C	-2.10460	-1.11490	0.65770
H	-1.16610	-1.29580	0.13270
H	-2.82620	-0.79920	-0.09500
C	-2.58330	-2.42100	1.30830
H	-1.83610	-2.76540	2.02380
H	-3.49240	-2.22810	1.87660
C	-2.86230	-3.52980	0.28490
H	-1.96580	-3.71590	-0.30720
H	-3.62810	-3.18570	-0.40950
C	-3.32030	-4.84080	0.93980
H	-2.53220	-5.21470	1.59400
H	-4.18380	-4.64500	1.57450
C	-3.68980	-5.91950	-0.08690
H	-2.84010	-6.10540	-0.74470
H	-4.49580	-5.54760	-0.71890
C	-4.12420	-7.23780	0.56830
H	-3.30120	-7.63710	1.16180
H	-4.94130	-7.04460	1.26270
C	-4.57410	-8.28630	-0.45750
H	-5.41350	-7.89060	-1.02900
H	-3.77070	-8.46670	-1.17270
C	-4.98360	-9.61380	0.19440
H	-4.13250	-10.03090	0.73400
H	-5.75900	-9.42810	0.93730
C	-5.49650	-10.63770	-0.82720
H	-6.36240	-10.22640	-1.34600
H	-4.73370	-10.80790	-1.58790
C	-5.87910	-11.97560	-0.18020
H	-5.00790	-12.40290	0.31760
H	-6.62590	-11.80780	0.59630
C	-6.42590	-12.98460	-1.19550
H	-7.32920	-12.60930	-1.67600
H	-6.67690	-13.92780	-0.70990
H	-5.69460	-13.19600	-1.97610
C	-3.71560	6.31310	0.10300
H	-3.48150	6.16030	1.15720
H	-2.80100	6.08490	-0.44710
C	-4.81480	5.33070	-0.30760
H	-5.72150	5.55540	0.25530
H	-5.06150	5.46890	-1.36070
C	-4.40120	3.87460	-0.05650
H	-4.04290	3.76810	0.96820
H	-3.56400	3.61610	-0.70630
C	-5.55600	2.89530	-0.28390
H	-6.37870	3.14840	0.38600
H	-5.93950	3.01440	-1.29720
C	-5.14380	1.43570	-0.05610
H	-4.72170	1.32420	0.94310
H	-4.35430	1.16590	-0.75770

C	-6.32810	0.47760	-0.21800
H	-7.09650	0.72740	0.51450
H	-6.78170	0.62860	-1.19740
C	-5.93090	-0.99540	-0.06270
H	-5.45600	-1.14850	0.90650
H	-5.18670	-1.25310	-0.81610
C	-7.14120	-1.92700	-0.19390
H	-7.86290	-1.69260	0.58930
H	-7.64510	-1.73090	-1.14030
C	-6.76580	-3.41190	-0.11850
H	-6.23660	-3.61030	0.81330
H	-6.07350	-3.65240	-0.92510
C	-7.99910	-4.31870	-0.21120
H	-8.55340	-4.08190	-1.11950
H	-8.66960	-4.10170	0.62120
C	-7.64550	-5.81110	-0.20890
H	-7.00390	-6.03150	-1.06180
H	-7.06720	-6.04940	0.68370
C	-8.89450	-6.70040	-0.26300
H	-9.49250	-6.43050	-1.13370
H	-9.51700	-6.50240	0.61040
C	-8.55780	-8.19580	-0.32320
H	-7.95280	-8.39370	-1.20770
H	-7.94640	-8.46730	0.53760
C	-9.81440	-9.07560	-0.35710
H	-10.43910	-8.78810	-1.20300
H	-10.40960	-8.89770	0.53930
C	-9.48750	-10.56860	-0.45810
H	-8.92500	-10.78710	-1.36510
H	-10.39920	-11.16560	-0.48260
H	-8.89460	-10.90080	0.39390
O	3.00900	9.26860	0.09370
O	2.54670	8.34040	2.10700
C	3.47870	8.41880	1.08420
C	4.78880	8.98370	1.65320
H	5.17660	8.31730	2.42400
H	5.52970	9.07560	0.85860
H	4.62240	9.96590	2.09540
C	3.65610	7.01720	0.49240
H	4.04610	6.33910	1.25170
H	2.69600	6.63290	0.14850
H	4.35230	7.04830	-0.34610
O	1.50800	12.00510	-0.67860
Si	2.24840	12.36420	-2.15430
C	0.93320	12.23170	-3.51300
H	1.37300	12.28970	-4.50790
H	0.38470	11.29380	-3.45210
H	0.19790	13.03120	-3.43400
C	3.65670	11.13380	-2.44220
H	4.39190	11.17270	-1.63930
H	3.29180	10.11030	-2.49120
H	4.17650	11.34140	-3.37420
C	2.92650	14.14880	-2.04020
C	3.55910	14.54980	-3.38270
H	2.83700	14.49110	-4.19790
H	3.93720	15.57210	-3.35280
H	4.39710	13.89990	-3.63510
C	1.76980	15.10440	-1.70240
H	1.00940	15.10640	-2.48350
H	1.28200	14.81580	-0.77170
H	2.12090	16.13000	-1.58520
C	3.98820	14.21130	-0.92930
H	3.57850	13.88820	0.02700
H	4.83940	13.56780	-1.15320
H	4.36920	15.22460	-0.79940
C	-3.70050	10.35450	4.29760
C	-2.05320	9.13400	6.70510
O	-3.38580	10.36960	3.10890

O	-2.62320	9.34420	7.77810
N	-4.78340	9.70520	4.75400
N	-1.78660	7.90840	6.24170
H	-4.98550	9.77300	5.74300
H	-1.32490	7.83250	5.34330
C	-2.86310	11.11950	5.33720
H	-3.49710	11.27610	6.20640
C	-2.48470	12.54680	4.85240
H	-3.13570	12.87730	4.04140
H	-2.67160	13.24010	5.67040
C	-0.62900	11.21730	6.51490
H	-1.12060	11.68910	7.36460
C	-1.01300	12.69460	4.42880
H	-0.83880	12.05440	3.56250
C	-0.06530	12.29210	5.58120
H	0.21500	13.15550	6.18480
C	-2.11760	6.66980	6.92030
H	-3.16560	6.68300	7.22410
H	-1.51530	6.58240	7.82570
C	-5.77820	9.04910	3.91190
H	-6.01750	9.71500	3.08080
H	-6.70230	8.93830	4.48000
C	-1.62860	10.29360	5.79130
H	-1.13120	9.88110	4.91080
C	-1.85500	5.49080	5.98000
H	-0.81490	5.50800	5.65060
H	-2.46360	5.60650	5.08270
C	-2.15970	4.14070	6.63280
H	-1.51630	4.00160	7.50220
H	-3.18570	4.13950	7.00100
C	-1.97040	2.97610	5.65670
H	-0.94590	2.97060	5.28260
H	-2.61500	3.12900	4.79170
C	-2.29080	1.62390	6.29950
H	-1.60260	1.44150	7.12530
H	-3.29220	1.65650	6.72860
C	-2.21390	0.46840	5.29850
H	-1.21860	0.43040	4.85450
H	-2.91200	0.65840	4.48410
C	-2.54110	-0.88030	5.94730
H	-1.80670	-1.09680	6.72350
H	-3.51020	-0.81710	6.44220
C	-2.57140	-2.02780	4.93430
H	-1.61290	-2.08810	4.41780
H	-3.32230	-1.80990	4.17580
C	-2.88710	-3.37560	5.59220
H	-2.10720	-3.61770	6.31450
H	-3.81830	-3.29400	6.15300
C	-3.01180	-4.51180	4.57240
H	-2.09140	-4.58690	3.99220
H	-3.80580	-4.26920	3.86720
C	-3.31350	-5.86160	5.23370
H	-2.49730	-6.12660	5.90650
H	-4.20790	-5.76870	5.84970
C	-3.51770	-6.98350	4.20930
H	-4.34200	-6.71680	3.54870
H	-2.63130	-7.06990	3.57980
C	-3.81260	-8.33650	4.86850
H	-2.97430	-8.62010	5.50580
H	-4.68000	-8.23770	5.52130
C	-4.07140	-9.44510	3.84010
H	-4.91370	-9.16080	3.20990
H	-3.20900	-9.53650	3.17860
C	-4.36100	-10.80260	4.49420
H	-3.51420	-11.09710	5.11500
H	-5.21850	-10.71380	5.16240
C	-4.63560	-11.90320	3.46410
H	-5.50710	-11.66390	2.85560

H	-4.82680	-12.85720	3.95570
H	-3.78620	-12.03790	2.79390
C	-5.32030	7.68530	3.35640
H	-4.29570	7.74430	2.98810
H	-5.93810	7.47000	2.48450
C	-5.45910	6.50560	4.33340
H	-4.78010	6.63090	5.17740
H	-6.46840	6.50660	4.74520
C	-5.20760	5.15640	3.63360
H	-4.16200	5.08530	3.33280
H	-5.79030	5.12380	2.71250
C	-5.58900	3.93560	4.48520
H	-4.95630	3.89000	5.37100
H	-6.61190	4.05550	4.84160
C	-5.49430	2.61780	3.69770
H	-4.47530	2.47350	3.33990
H	-6.12200	2.68940	2.80900
C	-5.92880	1.39260	4.51640
H	-5.27060	1.27680	5.37710
H	-6.93010	1.56190	4.91220
C	-5.93450	0.09840	3.68860
H	-4.94690	-0.06860	3.25980
H	-6.61790	0.21760	2.84780
C	-6.35010	-1.13430	4.50530
H	-5.64110	-1.28890	5.31850
H	-7.31920	-0.95030	4.96940
C	-6.43980	-2.40520	3.64840
H	-5.48870	-2.57650	3.14480
H	-7.17990	-2.25220	2.86290
C	-6.81630	-3.65230	4.46150
H	-7.74540	-3.46690	5.00110
H	-6.05220	-3.84080	5.21600
C	-6.98840	-4.89520	3.57720
H	-7.78040	-4.70920	2.85150
H	-6.07860	-5.06390	3.00130
C	-7.32380	-6.15990	4.37980
H	-8.21170	-5.98080	4.98710
H	-6.51250	-6.37610	5.07560
C	-7.56720	-7.37590	3.47500
H	-8.40200	-7.16470	2.80590
H	-6.69760	-7.53720	2.83800
C	-7.86200	-8.65650	4.26720
H	-8.71860	-8.49460	4.92230
H	-7.01740	-8.88800	4.91670
C	-8.14460	-9.85570	3.35600
H	-9.02600	-9.68110	2.73850
H	-8.32170	-10.75730	3.94250
H	-7.30520	-10.05240	2.69000
O	1.08690	11.65850	5.06880
O	0.52660	10.51690	6.94220
C	1.55940	10.83060	6.07330
C	2.65300	11.56860	6.85820
H	3.04590	10.92320	7.64410
H	3.46670	11.84830	6.18880
H	2.24820	12.47020	7.31820
C	2.06490	9.53190	5.43700
H	2.45810	8.86740	6.20640
H	1.24750	9.02430	4.92520
H	2.85420	9.74880	4.71670
O	-0.81510	14.04860	4.04740
Si	0.62960	14.73740	3.47830
C	1.49730	13.53980	2.30290
H	2.51470	13.86490	2.09380
H	1.55820	12.53410	2.71350
H	0.97680	13.47980	1.35000
C	1.78360	15.12550	4.93360
H	1.27740	15.69110	5.71380
H	2.17630	14.21790	5.38870

H	2.64130	15.71440	4.61130
C	0.17700	16.34420	2.54510
C	1.44100	16.93090	1.89260
H	1.89200	16.22870	1.19100
H	1.21450	17.84340	1.34040
H	2.19660	17.17880	2.63840
C	-0.86840	16.01740	1.46430
H	-0.49080	15.29110	0.74520
H	-1.77420	15.59780	1.90420
H	-1.15670	16.90940	0.90760
C	-0.41520	17.35890	3.53550
H	-1.31100	16.96230	4.01210
H	0.29430	17.61170	4.32380
H	-0.69450	18.28690	3.03600
C	-6.29280	11.58690	8.32280
C	-4.70640	10.80920	10.93520
O	-5.68220	11.44980	7.26540
O	-5.41220	10.90270	11.94090
N	-7.38210	10.87530	8.63230
N	-4.13540	9.66160	10.54560
H	-7.85200	11.09520	9.50250
H	-3.57960	9.67260	9.69900
C	-5.78270	12.54950	9.40180
H	-6.52880	12.59180	10.19270
C	-5.66500	13.99720	8.86290
H	-6.28450	14.14270	7.97630
H	-6.07850	14.67070	9.61200
C	-3.75060	13.17110	10.77250
H	-4.41450	13.58470	11.53270
C	-4.21810	14.41460	8.55420
H	-3.84650	13.79420	7.73680
C	-3.30370	14.26240	9.79080
H	-3.20360	15.20980	10.32200
C	-4.26320	8.38660	11.23090
H	-5.31480	8.18670	11.44140
H	-3.74320	8.43860	12.18850
C	-7.82990	9.69400	7.91110
H	-7.79780	9.86200	6.83320
H	-8.87400	9.51920	8.16890
C	-4.45570	12.02190	10.02190
H	-3.79120	11.69930	9.21670
C	-3.67880	7.26740	10.36010
H	-2.63510	7.48840	10.13090
H	-4.20390	7.24780	9.40470
C	-3.77290	5.88830	11.02420
H	-3.20280	5.89170	11.95390
H	-4.80930	5.69100	11.29570
C	-3.26860	4.76250	10.11240
H	-2.24090	4.96680	9.80960
H	-3.86450	4.75320	9.19990
C	-3.33940	3.38460	10.78430
H	-2.69380	3.37330	11.66310
H	-4.35390	3.21310	11.14120
C	-2.94400	2.24350	9.83910
H	-1.93920	2.41780	9.45290
H	-3.61410	2.25090	8.97990
C	-2.99960	0.86700	10.51620
H	-2.28460	0.83530	11.33900
H	-3.98610	0.72370	10.95490
C	-2.71560	-0.28310	9.54190
H	-1.73950	-0.13770	9.07800
H	-3.45050	-0.25240	8.73820
C	-2.75980	-1.66140	10.21600
H	-1.98680	-1.71790	10.98290
H	-3.71460	-1.78120	10.72640
C	-2.57920	-2.81130	9.21670
H	-1.63580	-2.68800	8.68370
H	-3.36840	-2.75620	8.46760

C	-2.61090	-4.19330	9.88320
H	-1.79140	-4.27200	10.59830
H	-3.53280	-4.29730	10.45440
C	-2.51750	-5.33800	8.86560
H	-3.35130	-5.26470	8.16800
H	-1.60840	-5.22690	8.27340
C	-2.53010	-6.72340	9.52510
H	-1.67620	-6.81320	10.19740
H	-3.42230	-6.82200	10.14300
C	-2.49640	-7.86300	8.49750
H	-3.36390	-7.78370	7.84260
H	-1.61820	-7.75330	7.85990
C	-2.48080	-9.25010	9.15320
H	-1.60380	-9.34090	9.79490
H	-3.35040	-9.35910	9.80180
C	-2.47230	-10.38620	8.12490
H	-3.36080	-10.35410	7.49500
H	-2.45190	-11.35770	8.61920
H	-1.59910	-10.32450	7.47500
C	-6.96680	8.48270	8.30310
H	-6.87230	8.44470	9.38900
H	-5.95500	8.61100	7.91460
C	-7.54120	7.15110	7.81240
H	-8.54500	7.02800	8.22050
H	-7.65030	7.17010	6.72790
C	-6.66890	5.95850	8.22860
H	-6.44720	6.02270	9.29460
H	-5.71110	6.00720	7.70860
C	-7.34980	4.61580	7.94640
H	-8.28890	4.56930	8.49900
H	-7.61530	4.55500	6.89120
C	-6.47500	3.41430	8.32730
H	-6.16120	3.50200	9.36800
H	-5.56500	3.41970	7.72660
C	-7.21720	2.08800	8.13230
H	-8.09950	2.07280	8.77310
H	-7.58650	2.02790	7.10880
C	-6.34630	0.86100	8.43000
H	-5.95260	0.92450	9.44470
H	-5.48450	0.85520	7.76230
C	-7.13470	-0.44350	8.26640
H	-7.96570	-0.45330	8.97240
H	-7.58180	-0.47170	7.27280
C	-6.27450	-1.69700	8.46740
H	-5.80750	-1.66990	9.45200
H	-5.46380	-1.70080	7.73880
C	-7.10010	-2.98160	8.32490
H	-7.61060	-2.97640	7.36180
H	-7.88250	-2.99450	9.08460
C	-6.25280	-4.25460	8.43930
H	-5.48680	-4.24850	7.66400
H	-5.72830	-4.26200	9.39500
C	-7.10230	-5.52540	8.30910
H	-7.65390	-5.49540	7.36900
H	-7.84930	-5.54450	9.10360
C	-6.26440	-6.80890	8.36370
H	-5.52410	-6.78980	7.56440
H	-5.70920	-6.84270	9.30150
C	-7.12380	-8.07290	8.23010
H	-7.68920	-8.03340	7.29850
H	-7.85810	-8.10280	9.03590
C	-6.29070	-9.35790	8.25910
H	-5.57190	-9.37840	7.44080
H	-6.92790	-10.23700	8.16180
H	-5.73880	-9.44860	9.19470
O	-2.02520	13.79300	9.41570
O	-2.53100	12.75530	11.36310
C	-1.49810	13.18020	10.54260

C	-0.64130	14.19540	11.31380
H	-0.20930	13.72240	12.19600
H	0.16470	14.56070	10.67690
H	-1.24740	15.04210	11.63570
C	-0.69580	11.94940	10.10900
H	-0.25640	11.46540	10.98130
H	-1.34970	11.23370	9.61120
H	0.10260	12.24220	9.42670
O	-4.23800	15.77480	8.16790
Si	-3.33570	16.43740	6.90180
C	-4.13030	15.88300	5.27260
H	-3.50610	16.12650	4.41400
H	-4.30600	14.80930	5.25580
H	-5.09680	16.36090	5.11900
C	-1.54550	15.83450	7.03780
H	-1.11100	16.09190	8.00300
H	-1.47320	14.75420	6.93380
H	-0.91810	16.27880	6.26890
C	-3.41500	18.33990	7.07810
C	-2.67520	18.99820	5.90180
H	-3.10960	18.70870	4.94430
H	-2.71820	20.08590	5.96570
H	-1.62280	18.71400	5.88630
C	-4.88760	18.78410	7.08470
H	-5.38850	18.52890	6.15080
H	-5.43890	18.30500	7.89380
H	-4.97800	19.86220	7.22000
C	-2.74980	18.74810	8.40330
H	-3.22940	18.26110	9.25200
H	-1.69490	18.47340	8.42270
H	-2.81060	19.82490	8.56370
C	-9.39540	12.05620	12.10990
C	-7.95520	11.48380	14.87720
O	-8.79790	12.18100	11.04260
O	-8.77010	11.43170	15.80070
N	-10.29650	11.08740	12.32860
N	-7.14050	10.47010	14.56400
H	-10.76800	11.08190	13.22470
H	-6.50840	10.59370	13.78230
C	-9.15310	13.04810	13.25810
H	-9.97040	12.93300	13.96670
C	-9.24040	14.51960	12.76810
H	-9.77110	14.59160	11.81720
H	-9.85590	15.07100	13.47660
C	-7.42120	13.95770	14.86020
H	-8.21730	14.18440	15.56940
C	-7.87290	15.21800	12.64910
H	-7.28540	14.69880	11.89000
C	-7.12630	15.19170	14.00120
H	-7.34550	16.07980	14.59370
C	-7.07120	9.21310	15.28700
H	-8.07270	8.79520	15.39850
H	-6.68200	9.39820	16.28940
C	-10.76730	10.14590	11.31930
H	-11.02080	10.70350	10.41590
H	-11.69830	9.69750	11.66670
C	-7.82440	12.74120	14.00290
H	-7.03410	12.57330	13.26870
C	-6.16980	8.23050	14.53500
H	-5.19000	8.68140	14.37050
H	-6.59300	8.04150	13.54790
C	-6.00000	6.90480	15.28320
H	-5.52300	7.08530	16.24730
H	-6.98180	6.48330	15.49680
C	-5.17790	5.89280	14.47970
H	-4.19610	6.31070	14.25380
H	-5.66850	5.71950	13.52230
C	-5.01230	4.55800	15.21290

H	-4.44930	4.70990	16.13440
H	-5.99350	4.18490	15.50490
C	-4.31530	3.50670	14.34430
H	-3.33100	3.86850	14.04470
H	-4.88880	3.37110	13.42780
C	-4.17470	2.15680	15.05530
H	-3.53530	2.26670	15.93180
H	-5.15180	1.83940	15.41860
C	-3.60910	1.07340	14.13220
H	-2.63360	1.38260	13.75520
H	-4.26140	0.97500	13.26500
C	-3.48590	-0.28660	14.82770
H	-2.78030	-0.21310	15.65570
H	-4.44900	-0.55670	15.26020
C	-3.04080	-1.39250	13.86540
H	-2.08390	-1.12530	13.41570
H	-3.76000	-1.46010	13.04970
C	-2.92390	-2.75860	14.55120
H	-2.16340	-2.71260	15.33130
H	-3.86570	-2.99290	15.04670
C	-2.58160	-3.87910	13.56280
H	-3.35380	-3.92300	12.79540
H	-1.64820	-3.64280	13.05060
C	-2.46220	-5.24940	14.24120
H	-1.66050	-5.22120	14.98000
H	-3.38110	-5.46290	14.78730
C	-2.19450	-6.37700	13.23630
H	-3.00240	-6.40530	12.50560
H	-1.28230	-6.15930	12.67930
C	-2.06840	-7.75040	13.90890
H	-1.24690	-7.73120	14.62560
H	-2.97300	-7.96550	14.47910
C	-1.82790	-8.87810	12.90020
H	-2.65610	-8.95980	12.19720
H	-1.72750	-9.83820	13.40670
H	-0.91670	-8.70710	12.32640
C	-9.74170	9.04950	10.97410
H	-8.77430	9.49340	10.74060
H	-10.07720	8.56470	10.05680
C	-9.56230	7.97010	12.05200
H	-9.13970	8.40590	12.95820
H	-10.54200	7.57660	12.32220
C	-8.67780	6.81650	11.54890
H	-7.66110	7.17460	11.38410
H	-9.04660	6.48960	10.57620
C	-8.65540	5.60280	12.48820
H	-8.18770	5.87490	13.43410
H	-9.67990	5.31250	12.72170
C	-7.93160	4.40060	11.86170
H	-6.90220	4.66900	11.62580
H	-8.40840	4.15670	10.91190
C	-7.94530	3.15460	12.75750
H	-7.39910	3.36000	13.67780
H	-8.97210	2.93260	13.04960
C	-7.34910	1.92500	12.05660
H	-6.32940	2.13920	11.73810
H	-7.91830	1.72430	11.14890
C	-7.35330	0.66920	12.93830
H	-6.72700	0.83690	13.81430
H	-8.36330	0.49080	13.30850
C	-6.86810	-0.57510	12.18110
H	-5.86950	-0.39800	11.78250
H	-7.51700	-0.74210	11.32140
C	-6.84950	-1.83980	13.04990
H	-7.83280	-1.98730	13.49790
H	-6.15050	-1.70440	13.87500
C	-6.46710	-3.08960	12.24470
H	-7.18520	-3.22590	11.43570

H	-5.49710	-2.93850	11.77130
C	-6.42080	-4.36340	13.09870
H	-7.37470	-4.49400	13.61130
H	-5.66540	-4.25180	13.87680
C	-6.11830	-5.61290	12.25920
H	-6.88810	-5.72890	11.49540
H	-5.17740	-5.47570	11.72660
C	-6.04210	-6.89230	13.10260
H	-6.97350	-7.02610	13.65380
H	-5.25480	-6.79180	13.84990
C	-5.77630	-8.13730	12.24920
H	-6.57840	-8.29970	11.52860
H	-5.70230	-9.02840	12.87280
H	-4.84420	-8.04330	11.69290
O	-5.73660	15.07790	13.78470
O	-6.20160	13.73570	15.54780
C	-5.21180	14.46590	14.90980
C	-4.69910	15.54390	15.87530
H	-4.26880	15.07510	16.76060
H	-3.93450	16.14790	15.38640
H	-5.51630	16.19390	16.18830
C	-4.10950	13.49950	14.46570
H	-3.67080	13.00810	15.33430
H	-4.52590	12.73700	13.80790
H	-3.32890	14.04050	13.93000
O	-8.11150	16.54670	12.20850
Si	-6.95660	17.76560	11.94970
C	-5.44980	17.05770	11.05750
H	-4.61300	17.75310	11.08920
H	-5.11290	16.12530	11.50620
H	-5.66680	16.86630	10.00930
C	-6.40060	18.51200	13.60330
H	-7.24740	18.80340	14.22130
H	-5.79780	17.81240	14.17950
H	-5.79110	19.40140	13.44970
C	-7.78570	19.10800	10.87050
C	-6.75030	20.18630	10.50640
H	-5.90930	19.76280	9.95610
H	-7.19120	20.96310	9.88110
H	-6.34900	20.67160	11.39640
C	-8.33210	18.45400	9.58930
H	-7.53930	17.98560	9.00660
H	-9.06510	17.68030	9.82140
H	-8.82070	19.18670	8.94650
C	-8.94690	19.73930	11.65640
H	-9.68930	18.98920	11.93000
H	-8.59960	20.20960	12.57670
H	-9.45550	20.50430	11.06920
C	-0.59510	5.09880	-9.09360
C	0.85720	3.03470	-7.22410
O	-0.62920	4.68420	-10.25200
O	0.84230	3.33570	-6.02970
N	-1.69120	5.31970	-8.35600
N	0.27880	1.93120	-7.71700
H	-1.56120	5.70070	-7.42690
H	0.34520	1.78480	-8.71380
C	0.74910	5.30430	-8.38460
H	0.54840	5.66180	-7.37620
C	1.58310	6.41190	-9.07430
H	0.94890	7.07800	-9.66210
H	2.02070	7.04140	-8.30140
C	3.00450	4.21130	-7.98610
H	3.11270	4.77790	-7.06110
C	2.70090	5.84690	-9.96320
H	2.24050	5.25900	-10.75970
C	3.66950	4.95970	-9.15220
H	4.51950	5.53320	-8.78170
C	-0.49060	0.95180	-6.96750

H	-1.25620	1.45930	-6.37940
H	0.16610	0.42920	-6.27060
C	-3.03870	4.91120	-8.72710
H	-3.24630	5.18040	-9.76420
H	-3.74440	5.46830	-8.11140
C	1.51160	3.95150	-8.27330
H	1.46350	3.43640	-9.23530
C	-1.14080	-0.03630	-7.94790
H	-0.36370	-0.51700	-8.54400
H	-1.76770	0.51450	-8.65000
C	-1.98610	-1.11730	-7.26210
H	-1.35800	-1.68710	-6.57670
H	-2.75870	-0.64400	-6.65720
C	-2.64390	-2.07040	-8.27220
H	-1.87480	-2.52210	-8.89990
H	-3.28910	-1.49940	-8.94060
C	-3.46130	-3.18040	-7.59700
H	-2.80630	-3.76690	-6.95210
H	-4.21400	-2.73270	-6.94900
C	-4.15230	-4.11100	-8.60430
H	-3.40740	-4.53870	-9.27660
H	-4.83180	-3.52910	-9.22750
C	-4.92660	-5.24460	-7.91630
H	-4.23480	-5.84320	-7.32290
H	-5.64550	-4.81990	-7.21610
C	-5.66660	-6.15470	-8.90630
H	-4.95740	-6.56250	-9.62770
H	-6.38120	-5.56170	-9.47780
C	-6.39800	-7.30770	-8.20310
H	-5.67290	-7.91860	-7.66450
H	-7.07770	-6.90380	-7.45300
C	-7.18930	-8.19430	-9.17400
H	-6.51910	-8.58280	-9.94170
H	-7.93280	-7.58790	-9.69220
C	-7.88360	-9.36560	-8.46380
H	-7.13280	-9.98980	-7.97810
H	-8.52520	-8.98250	-7.67020
C	-8.72120	-10.22520	-9.41970
H	-9.48550	-9.60400	-9.88760
H	-8.08770	-10.59450	-10.22720
C	-9.38720	-11.41360	-8.71150
H	-8.61830	-12.05140	-8.27380
H	-9.99410	-11.05030	-7.88200
C	-10.26520	-12.24540	-9.65610
H	-11.04240	-11.60890	-10.08060
H	-9.66420	-12.59870	-10.49500
C	-10.91330	-13.44600	-8.95260
H	-10.13650	-14.09880	-8.55300
H	-11.49460	-13.10100	-8.09730
C	-11.82190	-14.25430	-9.88470
H	-12.63520	-13.64040	-10.27280
H	-12.26620	-15.09880	-9.35740
H	-11.26410	-14.64900	-10.73430
C	-3.21420	3.39830	-8.51020
H	-2.89090	3.13650	-7.50240
H	-2.55320	2.85860	-9.18970
C	-4.65350	2.91220	-8.71720
H	-5.31140	3.41490	-8.00740
H	-4.99720	3.19400	-9.71320
C	-4.76830	1.39080	-8.54510
H	-4.35260	1.10100	-7.57960
H	-4.15720	0.89820	-9.30230
C	-6.21180	0.88150	-8.64600
H	-6.81400	1.34030	-7.86090
H	-6.64630	1.19880	-9.59480
C	-6.29370	-0.64710	-8.53340
H	-5.81610	-0.96990	-7.60790
H	-5.72460	-1.09840	-9.34690

C	-7.73590	-1.16940	-8.57010
H	-8.29500	-0.74890	-7.73330
H	-8.22810	-0.81810	-9.47780
C	-7.80230	-2.70180	-8.51660
H	-7.27450	-3.05750	-7.63160
H	-7.27540	-3.11570	-9.37700
C	-9.24250	-3.23130	-8.49810
H	-9.75920	-2.84380	-7.61930
H	-9.78200	-2.85020	-9.36610
C	-9.30250	-4.76500	-8.49590
H	-8.73100	-5.14930	-7.65100
H	-8.81830	-5.14670	-9.39530
C	-10.73940	-5.29920	-8.42460
H	-11.32020	-4.89320	-9.25370
H	-11.21390	-4.93950	-7.51070
C	-10.79910	-6.83240	-8.46510
H	-10.35440	-7.18720	-9.39540
H	-10.19180	-7.24060	-7.65740
C	-12.23210	-7.36920	-8.34750
H	-12.84600	-6.94360	-9.14230
H	-12.66850	-7.03160	-7.40660
C	-12.29560	-8.90110	-8.42120
H	-11.88090	-9.23560	-9.37260
H	-11.66410	-9.32820	-7.64230
C	-13.72550	-9.43840	-8.27140
H	-14.36260	-9.00450	-9.04310
H	-14.13830	-9.11730	-7.31430
C	-13.79380	-10.96640	-8.36360
H	-13.42940	-11.31970	-9.32830
H	-14.81930	-11.31760	-8.24780
H	-13.19280	-11.43580	-7.58520
O	4.13930	3.90040	-9.95510
O	3.73630	2.99950	-7.91590
C	4.45980	2.86770	-9.09130
C	5.95690	2.93640	-8.75640
H	6.22600	2.11790	-8.08850
H	6.54660	2.85750	-9.67000
H	6.19410	3.87970	-8.26420
C	4.06330	1.54670	-9.75740
H	4.29000	0.71140	-9.09440
H	2.99490	1.54510	-9.97420
H	4.61270	1.42010	-10.69060
O	3.42050	6.93020	-10.51600
Si	3.84600	7.06190	-12.14610
C	2.25070	7.19440	-13.15940
H	2.45900	7.29870	-14.22350
H	1.62480	6.31120	-13.03440
H	1.65740	8.05610	-12.85570
C	4.81730	5.51810	-12.65830
H	5.68640	5.36540	-12.01920
H	4.20360	4.62080	-12.58690
H	5.17260	5.58930	-13.68550
C	4.91230	8.63660	-12.33870
C	5.26980	8.83830	-13.82110
H	4.37590	8.95560	-14.43440
H	5.88230	9.72930	-13.96240
H	5.83010	7.99000	-14.21510
C	4.11520	9.84650	-11.82280
H	3.19960	9.99630	-12.39550
H	3.83030	9.71330	-10.77900
H	4.69810	10.76560	-11.88900
C	6.19760	8.47630	-11.50940
H	5.96970	8.30250	-10.45780
H	6.79580	7.63400	-11.85800
H	6.82210	9.36820	-11.56710
C	-0.85450	6.91500	-4.50190
C	0.74530	5.08330	-2.49210
O	-0.81960	6.90640	-5.73140

O	0.50340	5.40890	-1.32790
N	-1.96370	6.61950	-3.80390
N	0.47500	3.87270	-2.99090
H	-1.90850	6.67580	-2.79570
H	0.70250	3.70270	-3.96330
C	0.39340	7.29990	-3.68660
H	0.04550	7.61240	-2.70470
C	1.12480	8.54150	-4.27110
H	0.47040	9.11600	-4.92880
H	1.36150	9.21160	-3.44630
C	2.72180	6.58460	-3.01280
H	2.60430	7.15520	-2.09240
C	2.43620	8.20670	-5.00490
H	2.19510	7.59170	-5.87320
C	3.41010	7.44750	-4.07500
H	4.09320	8.13260	-3.57270
C	-0.16460	2.78860	-2.27070
H	-1.05020	3.15910	-1.75190
H	0.52090	2.40100	-1.51590
C	-3.28030	6.38360	-4.38820
H	-3.45590	7.14620	-5.14920
H	-4.04100	6.54770	-3.62450
C	1.34300	6.08640	-3.49010
H	1.48000	5.58250	-4.44960
C	-0.55500	1.69250	-3.26600
H	0.33390	1.34840	-3.79700
H	-1.21880	2.11510	-4.02090
C	-1.24040	0.49920	-2.59740
H	-0.56470	0.05480	-1.86600
H	-2.11470	0.84480	-2.04580
C	-1.66740	-0.55990	-3.61860
H	-0.79580	-0.90320	-4.17730
H	-2.34140	-0.10600	-4.34460
C	-2.35670	-1.75810	-2.96020
H	-1.66520	-2.23480	-2.26510
H	-3.20270	-1.40880	-2.36840
C	-2.84750	-2.78690	-3.98330
H	-2.00940	-3.12930	-4.59120
H	-3.55110	-2.30830	-4.66390
C	-3.51930	-3.99070	-3.31540
H	-2.79890	-4.49130	-2.66810
H	-4.32740	-3.64310	-2.67180
C	-4.07860	-4.99300	-4.33010
H	-3.28100	-5.32620	-4.99490
H	-4.81830	-4.49470	-4.95600
C	-4.71740	-6.20860	-3.65010
H	-3.96340	-6.72780	-3.05790
H	-5.48310	-5.87110	-2.95150
C	-5.34190	-7.18530	-4.65220
H	-4.58660	-7.50670	-5.37020
H	-6.11350	-6.66910	-5.22270
C	-5.94860	-8.41390	-3.96460
H	-5.16660	-8.94980	-3.42590
H	-6.67250	-8.08830	-3.21760
C	-6.63210	-9.36530	-4.95360
H	-7.42420	-8.83020	-5.47700
H	-5.91570	-9.67940	-5.71360
C	-7.22060	-10.60170	-4.26300
H	-6.42210	-11.15360	-3.76600
H	-7.90960	-10.28300	-3.48090
C	-7.95170	-11.53010	-5.24120
H	-8.75120	-10.97690	-5.73370
H	-7.26420	-11.84360	-6.02770
C	-8.53860	-12.76880	-4.55100
H	-7.73860	-13.33130	-4.06860
H	-9.22080	-12.45950	-3.75820
C	-9.28100	-13.68810	-5.52620
H	-10.11830	-13.17150	-5.99420

H	-9.67790	-14.56280	-5.01070
H	-8.61960	-14.04000	-6.31830
C	-3.44180	4.98680	-5.02350
H	-2.53350	4.69490	-5.55210
H	-4.21300	5.07340	-5.78920
C	-3.87180	3.87080	-4.05340
H	-3.07810	3.66770	-3.33390
H	-4.73170	4.21320	-3.47710
C	-4.25900	2.58460	-4.80940
H	-3.37710	2.16320	-5.29210
H	-4.94790	2.84590	-5.61310
C	-4.93150	1.51250	-3.93620
H	-4.23100	1.15760	-3.18100
H	-5.76840	1.95790	-3.39860
C	-5.45300	0.32840	-4.76940
H	-4.62750	-0.12500	-5.31690
H	-6.14830	0.70220	-5.52160
C	-6.15870	-0.74810	-3.93030
H	-5.45740	-1.16330	-3.20720
H	-6.96300	-0.28970	-3.35510
C	-6.74280	-1.88030	-4.78980
H	-5.95420	-2.32040	-5.39930
H	-7.47130	-1.46270	-5.48520
C	-7.41180	-2.98240	-3.95470
H	-6.67630	-3.42520	-3.28340
H	-8.18250	-2.54130	-3.32220
C	-8.04280	-4.08320	-4.81960
H	-7.28970	-4.50020	-5.48770
H	-8.81000	-3.64290	-5.45680
C	-8.66100	-5.21320	-3.98360
H	-9.39200	-4.79480	-3.29110
H	-7.88690	-5.67740	-3.37240
C	-9.34180	-6.28390	-4.84700
H	-10.14310	-5.82270	-5.42490
H	-8.62830	-6.67860	-5.57040
C	-9.91380	-7.43940	-4.01390
H	-10.60480	-7.04360	-3.26900
H	-9.10740	-7.92180	-3.46110
C	-10.64100	-8.48190	-4.87350
H	-11.47040	-8.00310	-5.39480
H	-9.96620	-8.85370	-5.64470
C	-11.17100	-9.66200	-4.04780
H	-11.83520	-9.29470	-3.26490
H	-10.34200	-10.15580	-3.54100
C	-11.92210	-10.68660	-4.90350
H	-12.80630	-10.24170	-5.36020
H	-12.24940	-11.53330	-4.30000
H	-11.29070	-11.07220	-5.70340
O	4.13790	6.48230	-4.80370
O	3.63460	5.51800	-2.81820
C	4.50770	5.50500	-3.89400
C	5.92330	5.80590	-3.38110
H	6.23390	5.03580	-2.67470
H	6.62480	5.82570	-4.21550
H	5.94850	6.77150	-2.87570
C	4.40810	4.13740	-4.57700
H	4.71650	3.35230	-3.88660
H	3.37910	3.94620	-4.88080
H	5.05040	4.10930	-5.45770
O	3.00290	9.43090	-5.45060
Si	4.42090	9.61090	-6.36900
C	4.52650	8.24650	-7.66820
H	5.51750	8.20270	-8.11680
H	4.32060	7.26730	-7.24200
H	3.81760	8.41910	-8.47470
C	5.94630	9.50790	-5.24520
H	5.88390	10.19870	-4.40670
H	6.07750	8.50750	-4.83640

H	6.85620	9.74800	-5.79380
C	4.33930	11.31300	-7.23620
C	5.52120	11.44840	-8.21210
H	5.49700	10.67380	-8.97940
H	5.50490	12.41190	-8.72230
H	6.47740	11.36810	-7.69430
C	3.01120	11.41860	-8.00590
H	2.91990	10.63700	-8.75970
H	2.15620	11.32410	-7.33500
H	2.92300	12.37780	-8.51670
C	4.40500	12.42540	-6.17870
H	3.57730	12.34310	-5.47500
H	5.33180	12.38100	-5.60620
H	4.34890	13.41380	-6.63570

3 monomer

C	29.52320000	-20.61040000	-17.67660000
C	26.58600000	-20.03400000	-17.95670000
O	30.10280000	-19.60300000	-17.27130000
O	25.88390000	-21.04350000	-18.01260000
N	29.42720000	-21.71900000	-16.92880000
N	26.55480000	-19.19830000	-16.91180000
H	28.89950000	-22.51120000	-17.28700000
H	27.12800000	-18.35550000	-16.96830000
C	28.85270000	-20.58660000	-19.06560000
H	29.58440000	-20.10680000	-19.71710000
C	27.57260000	-19.70070000	-19.09250000
H	27.89150000	-18.67590000	-18.92790000
C	28.59970000	-21.99180000	-19.64680000
H	29.51480000	-22.58580000	-19.61990000
H	27.86470000	-22.51520000	-19.04380000
C	26.86060000	-19.72500000	-20.46870000
H	25.85300000	-19.31990000	-20.35410000
C	28.08410000	-21.90300000	-21.08360000
H	28.85930000	-21.45070000	-21.70050000
C	26.77060000	-21.08910000	-21.16330000
H	25.92670000	-21.65120000	-20.76900000
C	25.81440000	-19.42860000	-15.67850000
H	25.75240000	-18.48310000	-15.13930000
H	24.79070000	-19.72800000	-15.91030000
C	30.00840000	-21.85130000	-15.59980000
H	31.09340000	-21.91710000	-15.69270000
H	29.79390000	-20.95700000	-15.01250000
C	26.50700000	-20.49200000	-14.80810000
H	26.55220000	-21.43630000	-15.35340000
H	27.53990000	-20.19160000	-14.63160000
C	25.80760000	-20.72450000	-13.46220000
H	25.77800000	-19.78870000	-12.90330000
H	24.77090000	-21.01510000	-13.63450000
C	26.51100000	-21.80290000	-12.62480000
H	27.55860000	-21.53110000	-12.49540000
H	26.50590000	-22.74710000	-13.16980000
C	25.86740000	-22.00700000	-11.24660000
H	25.89520000	-21.06940000	-10.69090000
H	24.81380000	-22.25930000	-11.36760000
C	26.56540000	-23.10540000	-10.43190000
H	27.62760000	-22.87480000	-10.35040000
H	26.49770000	-24.05380000	-10.96490000
C	25.97200000	-23.26550000	-9.02580000
H	26.06980000	-22.32530000	-8.48240000
H	24.90280000	-23.46430000	-9.10070000
C	26.64440000	-24.39020000	-8.22660000
H	27.72060000	-24.21920000	-8.19490000
H	26.49830000	-25.34000000	-8.74130000
C	26.10440000	-24.49450000	-6.79410000

H	26.28150000	-23.55400000	-6.27150000
H	25.02300000	-24.62960000	-6.82080000
C	26.74200000	-25.64530000	-6.00450000
H	27.82690000	-25.53880000	-6.01870000
H	26.51810000	-26.59150000	-6.49710000
C	26.25490000	-25.69640000	-4.55030000
H	26.50770000	-24.76060000	-4.05090000
H	25.16710000	-25.76770000	-4.53270000
C	26.85520000	-26.87150000	-3.76750000
H	27.94330000	-26.82670000	-3.82120000
H	26.56130000	-27.80910000	-4.23920000
C	26.41740000	-26.87860000	-2.29680000
H	26.73450000	-25.95090000	-1.81940000
H	25.32860000	-26.89390000	-2.24130000
C	26.98470000	-28.07320000	-1.51920000
H	28.07180000	-28.07870000	-1.60240000
H	26.63640000	-28.99990000	-1.97540000
C	26.58520000	-28.05040000	-0.03770000
H	26.94700000	-27.13330000	0.42840000
H	25.49870000	-28.02700000	0.04860000
C	27.12830000	-29.25650000	0.73480000
H	28.21770000	-29.28030000	0.71230000
H	26.81810000	-29.22170000	1.77940000
H	26.76280000	-30.19220000	0.31250000
C	29.45460000	-23.08870000	-14.88190000
H	29.67890000	-23.98020000	-15.46950000
H	28.36770000	-23.01960000	-14.83200000
C	30.02210000	-23.25870000	-13.46640000
H	31.10690000	-23.35760000	-13.52210000
H	29.82320000	-22.35980000	-12.88250000
C	29.43020000	-24.47510000	-12.74190000
H	29.60440000	-25.37280000	-13.33650000
H	28.34900000	-24.35870000	-12.66770000
C	30.02140000	-24.67280000	-11.34010000
H	31.10000000	-24.81380000	-11.41930000
H	29.87020000	-23.76930000	-10.74930000
C	29.40070000	-25.86720000	-10.60450000
H	29.52220000	-26.76930000	-11.20520000
H	28.32770000	-25.70690000	-10.49940000
C	30.02260000	-26.09430000	-9.22090000
H	31.09030000	-26.28730000	-9.33160000
H	29.93270000	-25.18450000	-8.62780000
C	29.36640000	-27.25660000	-8.46520000
H	29.42450000	-28.16400000	-9.06750000
H	28.30640000	-27.04310000	-8.32760000
C	30.01850000	-27.51250000	-7.10090000
H	31.07000000	-27.76440000	-7.24380000
H	29.99800000	-26.59600000	-6.51190000
C	29.32310000	-28.63260000	-6.31690000
H	29.31320000	-29.54570000	-6.91330000
H	28.28090000	-28.36040000	-6.14950000
C	30.00130000	-28.91320000	-4.97030000
H	31.03260000	-29.22400000	-5.14160000
H	30.04930000	-27.99150000	-4.39130000
C	29.26940000	-29.98580000	-4.15360000
H	29.19700000	-30.90440000	-4.73730000
H	28.24710000	-29.65830000	-3.96380000
C	29.96670000	-30.28330000	-2.82010000
H	30.97840000	-30.64300000	-3.01210000
H	30.07130000	-29.35830000	-2.25400000
C	29.20670000	-31.31300000	-1.97390000
H	29.08510000	-32.23490000	-2.54420000
H	28.20200000	-30.94180000	-1.77000000
C	29.91540000	-31.62270000	-0.64850000
H	30.91480000	-32.01090000	-0.84950000
H	30.05210000	-30.70180000	-0.08060000
C	29.14410000	-32.63260000	0.20780000
H	29.01670000	-33.58020000	-0.31640000

H	29.67230000	-32.83720000	1.13930000
H	28.15410000	-32.25650000	0.46510000
O	27.51000000	-18.95390000	-21.45950000
O	26.54720000	-20.71410000	-22.51280000
O	27.86350000	-23.22190000	-21.53570000
H	26.92670000	-23.33200000	-21.68750000
C	27.00260000	-19.41050000	-22.66870000
C	25.82230000	-18.52330000	-23.08640000
H	26.15060000	-17.48670000	-23.17150000
H	25.43640000	-18.85250000	-24.05140000
H	25.01660000	-18.57890000	-22.35560000
C	28.12600000	-19.38840000	-23.70910000
H	27.76130000	-19.77880000	-24.65930000
H	28.47560000	-18.36610000	-23.85740000
H	28.96870000	-19.99530000	-23.38250000

3 dimer

C	29.52320000	-20.61040000	-17.67660000
C	26.58600000	-20.03400000	-17.95670000
O	30.10280000	-19.60300000	-17.27130000
O	25.88390000	-21.04350000	-18.01260000
N	29.42720000	-21.71900000	-16.92880000
N	26.55480000	-19.19830000	-16.91180000
H	28.89950000	-22.51120000	-17.28700000
H	27.12800000	-18.35550000	-16.96830000
C	28.85270000	-20.58660000	-19.06560000
H	29.58440000	-20.10680000	-19.71710000
C	27.57260000	-19.70070000	-19.09250000
H	27.89150000	-18.67590000	-18.92790000
C	28.59970000	-21.99180000	-19.64680000
H	29.51480000	-22.58580000	-19.61990000
H	27.86470000	-22.51520000	-19.04380000
C	26.86060000	-19.72500000	-20.46870000
H	25.85300000	-19.31990000	-20.35410000
C	28.08410000	-21.90300000	-21.08360000
H	28.85930000	-21.45070000	-21.70050000
C	26.77060000	-21.08910000	-21.16330000
H	25.92670000	-21.65120000	-20.76900000
C	25.81440000	-19.42860000	-15.67850000
H	25.75240000	-18.48310000	-15.13930000
H	24.79070000	-19.72800000	-15.91030000
C	30.00840000	-21.85130000	-15.59980000
H	31.09340000	-21.91710000	-15.69270000
H	29.79390000	-20.95700000	-15.01250000
C	26.50700000	-20.49200000	-14.80810000
H	26.55220000	-21.43630000	-15.35340000
H	27.53990000	-20.19160000	-14.63160000
C	25.80760000	-20.72450000	-13.46220000
H	25.77800000	-19.78870000	-12.90330000
H	24.77090000	-21.01510000	-13.63450000
C	26.51100000	-21.80290000	-12.62480000
H	27.55860000	-21.53110000	-12.49540000
H	26.50590000	-22.74710000	-13.16980000
C	25.86740000	-22.00700000	-11.24660000
H	25.89520000	-21.06940000	-10.69090000
H	24.81380000	-22.25930000	-11.36760000
C	26.56540000	-23.10540000	-10.43190000
H	27.62760000	-22.87480000	-10.35040000
H	26.49770000	-24.05380000	-10.96490000
C	25.97200000	-23.26550000	-9.02580000
H	26.06980000	-22.32530000	-8.48240000
H	24.90280000	-23.46430000	-9.10070000
C	26.64440000	-24.39020000	-8.22660000
H	27.72060000	-24.21920000	-8.19490000
H	26.49830000	-25.34000000	-8.74130000
C	26.10440000	-24.49450000	-6.79410000

H	26.28150000	-23.55400000	-6.27150000
H	25.02300000	-24.62960000	-6.82080000
C	26.74200000	-25.64530000	-6.00450000
H	27.82690000	-25.53880000	-6.01870000
H	26.51810000	-26.59150000	-6.49710000
C	26.25490000	-25.69640000	-4.55030000
H	26.50770000	-24.76060000	-4.05090000
H	25.16710000	-25.76770000	-4.53270000
C	26.85520000	-26.87150000	-3.76750000
H	27.94330000	-26.82670000	-3.82120000
H	26.56130000	-27.80910000	-4.23920000
C	26.41740000	-26.87860000	-2.29680000
H	26.73450000	-25.95090000	-1.81940000
H	25.32860000	-26.89390000	-2.24130000
C	26.98470000	-28.07320000	-1.51920000
H	28.07180000	-28.07870000	-1.60240000
H	26.63640000	-28.99990000	-1.97540000
C	26.58520000	-28.05040000	-0.03770000
H	26.94700000	-27.13330000	0.42840000
H	25.49870000	-28.02700000	0.04860000
C	27.12830000	-29.25650000	0.73480000
H	28.21770000	-29.28030000	0.71230000
H	26.81810000	-29.22170000	1.77940000
H	26.76280000	-30.19220000	0.31250000
C	29.45460000	-23.08870000	-14.88190000
H	29.67890000	-23.98020000	-15.46950000
H	28.36770000	-23.01960000	-14.83200000
C	30.02210000	-23.25870000	-13.46640000
H	31.10690000	-23.35760000	-13.52210000
H	29.82320000	-22.35980000	-12.88250000
C	29.43020000	-24.47510000	-12.74190000
H	29.60440000	-25.37280000	-13.33650000
H	28.34900000	-24.35870000	-12.66770000
C	30.02140000	-24.67280000	-11.34010000
H	31.10000000	-24.81380000	-11.41930000
H	29.87020000	-23.76930000	-10.74930000
C	29.40070000	-25.86720000	-10.60450000
H	29.52220000	-26.76930000	-11.20520000
H	28.32770000	-25.70690000	-10.49940000
C	30.02260000	-26.09430000	-9.22090000
H	31.09030000	-26.28730000	-9.33160000
H	29.93270000	-25.18450000	-8.62780000
C	29.36640000	-27.25660000	-8.46520000
H	29.42450000	-28.16400000	-9.06750000
H	28.30640000	-27.04310000	-8.32760000
C	30.01850000	-27.51250000	-7.10090000
H	31.07000000	-27.76440000	-7.24380000
H	29.99800000	-26.59600000	-6.51190000
C	29.32310000	-28.63260000	-6.31690000
H	29.31320000	-29.54570000	-6.91330000
H	28.28090000	-28.36040000	-6.14950000
C	30.00130000	-28.91320000	-4.97030000
H	31.03260000	-29.22400000	-5.14160000
H	30.04930000	-27.99150000	-4.39130000
C	29.26940000	-29.98580000	-4.15360000
H	29.19700000	-30.90440000	-4.73730000
H	28.24710000	-29.65830000	-3.96380000
C	29.96670000	-30.28330000	-2.82010000
H	30.97840000	-30.64300000	-3.01210000
H	30.07130000	-29.35830000	-2.25400000
C	29.20670000	-31.31300000	-1.97390000
H	29.08510000	-32.23490000	-2.54420000
H	28.20200000	-30.94180000	-1.77000000
C	29.91540000	-31.62270000	-0.64850000
H	30.91480000	-32.01090000	-0.84950000
H	30.05210000	-30.70180000	-0.08060000
C	29.14410000	-32.63260000	0.20780000
H	29.01670000	-33.58020000	-0.31640000

H	29.67230000	-32.83720000	1.13930000
H	28.15410000	-32.25650000	0.46510000
O	27.51000000	-18.95390000	-21.45950000
O	26.54720000	-20.71410000	-22.51280000
O	27.86350000	-23.22190000	-21.53570000
H	26.92670000	-23.33200000	-21.68750000
C	27.00260000	-19.41050000	-22.66870000
C	25.82230000	-18.52330000	-23.08640000
H	26.15060000	-17.48670000	-23.17150000
H	25.43640000	-18.85250000	-24.05140000
H	25.01660000	-18.57890000	-22.35560000
C	28.12600000	-19.38840000	-23.70910000
H	27.76130000	-19.77880000	-24.65930000
H	28.47560000	-18.36610000	-23.85740000
H	28.96870000	-19.99530000	-23.38250000
C	31.40790000	-15.72570000	-17.11800000
C	28.40030000	-15.60340000	-17.20330000
O	31.88710000	-14.72170000	-16.59200000
O	27.87600000	-16.69860000	-17.40560000
N	31.49270000	-16.93450000	-16.54390000
N	28.28250000	-14.96140000	-16.03490000
H	31.04770000	-17.72930000	-16.99640000
H	28.68550000	-14.02710000	-15.97260000
C	30.65870000	-15.58540000	-18.45770000
H	31.26530000	-14.89140000	-19.04130000
C	29.25620000	-14.92900000	-18.29330000
H	29.41720000	-13.90500000	-17.96920000
C	30.60050000	-16.89800000	-19.26260000
H	31.59920000	-17.32620000	-19.36390000
H	29.99450000	-17.63190000	-18.73980000
C	28.48100000	-14.84320000	-19.63150000
H	27.42980000	-14.63690000	-19.42000000
C	30.00010000	-16.65500000	-20.64720000
H	30.65800000	-15.98230000	-21.19580000
C	28.57120000	-16.06900000	-20.54790000
H	27.85100000	-16.81940000	-20.23040000
C	27.64590000	-15.50770000	-14.84270000
H	27.48680000	-14.68930000	-14.14030000
H	26.65970000	-15.89970000	-15.09790000
C	32.16850000	-17.17940000	-15.27690000
H	33.24450000	-17.07230000	-15.42280000
H	31.87030000	-16.42190000	-14.55040000
C	28.51210000	-16.60370000	-14.19400000
H	28.63980000	-17.43240000	-14.89240000
H	29.50980000	-16.20680000	-14.00780000
C	27.92480000	-17.14150000	-12.88150000
H	27.81020000	-16.31950000	-12.17460000
H	26.92360000	-17.53360000	-13.06240000
C	28.79900000	-18.23800000	-12.25290000
H	29.81570000	-17.86360000	-12.13240000
H	28.86380000	-19.08790000	-12.93300000
C	28.26980000	-18.70800000	-10.89050000
H	28.21540000	-17.85650000	-10.21170000
H	27.24890000	-19.07340000	-11.00240000
C	29.13890000	-19.80470000	-10.25720000
H	30.17130000	-19.45990000	-10.19440000
H	29.14630000	-20.68370000	-10.90220000
C	28.64980000	-20.19670000	-8.85570000
H	28.67100000	-19.31950000	-8.20850000
H	27.60680000	-20.50850000	-8.91000000
C	29.48290000	-21.31760000	-8.21770000
H	30.53410000	-21.02890000	-8.20390000
H	29.41660000	-22.21560000	-8.83220000
C	29.02390000	-21.63640000	-6.78770000
H	29.12660000	-20.74470000	-6.16850000
H	27.96160000	-21.88080000	-6.79500000
C	29.80530000	-22.79230000	-6.14780000
H	30.87260000	-22.57110000	-6.17460000

H	29.66130000	-23.69770000	-6.73730000
C	29.37400000	-23.05060000	-4.69730000
H	29.55460000	-22.15550000	-4.10150000
H	28.29840000	-23.22590000	-4.66560000
C	30.10070000	-24.24270000	-4.05980000
H	31.17830000	-24.08890000	-4.12150000
H	29.88330000	-25.14600000	-4.62940000
C	29.69770000	-24.45460000	-2.59390000
H	29.94400000	-23.56220000	-2.01780000
H	28.61570000	-24.57060000	-2.52890000
C	30.37840000	-25.67560000	-1.96030000
H	31.46040000	-25.57800000	-2.05230000
H	30.10080000	-26.57240000	-2.51370000
C	30.00340000	-25.85360000	-0.48260000
H	30.29560000	-24.96720000	0.08090000
H	28.92030000	-25.93150000	-0.38720000
C	30.65520000	-27.08840000	0.14840000
H	31.74250000	-27.03320000	0.09450000
H	30.37850000	-27.17960000	1.19890000
H	30.33970000	-28.00200000	-0.35410000
C	31.83750000	-18.57590000	-14.73390000
H	32.14750000	-19.33160000	-15.45720000
H	30.75680000	-18.67580000	-14.63000000
C	32.50970000	-18.85600000	-13.38260000
H	33.59250000	-18.78800000	-13.49600000
H	32.22510000	-18.08210000	-12.66980000
C	32.13680000	-20.23070000	-12.81100000
H	32.40150000	-21.00840000	-13.52860000
H	31.05570000	-20.28410000	-12.68230000
C	32.82570000	-20.51690000	-11.46950000
H	33.90800000	-20.49230000	-11.60380000
H	32.58420000	-19.72330000	-10.76270000
C	32.41270000	-21.86800000	-10.87090000
H	32.63010000	-22.66510000	-11.58300000
H	31.33380000	-21.87590000	-10.71750000
C	33.11820000	-22.16680000	-9.54100000
H	34.19710000	-22.19390000	-9.69910000
H	32.92880000	-21.35350000	-8.84090000
C	32.65580000	-23.48920000	-8.91480000
H	32.82100000	-24.30490000	-9.61960000
H	31.58100000	-23.44460000	-8.73990000
C	33.37190000	-23.80460000	-7.59460000
H	34.44470000	-23.88820000	-7.77300000
H	33.23770000	-22.97340000	-6.90270000
C	32.85550000	-25.09340000	-6.94100000
H	32.96450000	-25.92560000	-7.63750000
H	31.78820000	-24.99240000	-6.74510000
C	33.58160000	-25.42770000	-5.63120000
H	34.64530000	-25.56520000	-5.82980000
H	33.50180000	-24.58320000	-4.94710000
C	33.01710000	-26.68280000	-4.95200000
H	33.07050000	-27.52660000	-5.64120000
H	31.96020000	-26.52860000	-4.73480000
C	33.75530000	-27.03770000	-3.65450000
H	34.80800000	-27.22050000	-3.87430000
H	33.72560000	-26.18550000	-2.97570000
C	33.15430000	-28.26420000	-2.95480000
H	33.15810000	-29.11290000	-3.64020000
H	32.10940000	-28.06620000	-2.71540000
C	33.90720000	-28.64170000	-1.67220000
H	34.95090000	-28.85370000	-1.90830000
H	33.91280000	-27.79510000	-0.98540000
C	33.29140000	-29.85450000	-0.96680000
H	33.29230000	-30.73220000	-1.61380000
H	33.84980000	-30.10600000	-0.06480000
H	32.26100000	-29.65610000	-0.67280000
O	28.94260000	-13.82070000	-20.49160000
O	28.21350000	-15.51860000	-21.80480000

O	29.97300000	-17.89750000	-21.31490000
H	29.05970000	-18.13680000	-21.46280000
C	28.44520000	-14.14980000	-21.74660000
C	27.11620000	-13.41580000	-21.96990000
H	27.26910000	-12.33930000	-21.88560000
H	26.73270000	-13.64160000	-22.96510000
H	26.37310000	-13.72500000	-21.23600000
C	29.48730000	-13.76990000	-22.80240000
H	29.13350000	-14.05370000	-23.79350000
H	29.66080000	-12.69340000	-22.78380000
H	30.43340000	-14.27390000	-22.61200000

3 tetramer

C	29.52320000	-20.61040000	-17.67660000
C	26.58600000	-20.03400000	-17.95670000
O	30.10280000	-19.60300000	-17.27130000
O	25.88390000	-21.04350000	-18.01260000
N	29.42720000	-21.71900000	-16.92880000
N	26.55480000	-19.19830000	-16.91180000
H	28.89950000	-22.51120000	-17.28700000
H	27.12800000	-18.35550000	-16.96830000
C	28.85270000	-20.58660000	-19.06560000
H	29.58440000	-20.10680000	-19.71710000
C	27.57260000	-19.70070000	-19.09250000
H	27.89150000	-18.67590000	-18.92790000
C	28.59970000	-21.99180000	-19.64680000
H	29.51480000	-22.58580000	-19.61990000
H	27.86470000	-22.51520000	-19.04380000
C	26.86060000	-19.72500000	-20.46870000
H	25.85300000	-19.31990000	-20.35410000
C	28.08410000	-21.90300000	-21.08360000
H	28.85930000	-21.45070000	-21.70050000
C	26.77060000	-21.08910000	-21.16330000
H	25.92670000	-21.65120000	-20.76900000
C	25.81440000	-19.42860000	-15.67850000
H	25.75240000	-18.48310000	-15.13930000
H	24.79070000	-19.72800000	-15.91030000
C	30.00840000	-21.85130000	-15.59980000
H	31.09340000	-21.91710000	-15.69270000
H	29.79390000	-20.95700000	-15.01250000
C	26.50700000	-20.49200000	-14.80810000
H	26.55220000	-21.43630000	-15.35340000
H	27.53990000	-20.19160000	-14.63160000
C	25.80760000	-20.72450000	-13.46220000
H	25.77800000	-19.78870000	-12.90330000
H	24.77090000	-21.01510000	-13.63450000
C	26.51100000	-21.80290000	-12.62480000
H	27.55860000	-21.53110000	-12.49540000
H	26.50590000	-22.74710000	-13.16980000
C	25.86740000	-22.00700000	-11.24660000
H	25.89520000	-21.06940000	-10.69090000
H	24.81380000	-22.25930000	-11.36760000
C	26.56540000	-23.10540000	-10.43190000
H	27.62760000	-22.87480000	-10.35040000
H	26.49770000	-24.05380000	-10.96490000
C	25.97200000	-23.26550000	-9.02580000
H	26.06980000	-22.32530000	-8.48240000
H	24.90280000	-23.46430000	-9.10070000
C	26.64440000	-24.39020000	-8.22660000
H	27.72060000	-24.21920000	-8.19490000
H	26.49830000	-25.34000000	-8.74130000
C	26.10440000	-24.49450000	-6.79410000
H	26.28150000	-23.55400000	-6.27150000
H	25.02300000	-24.62960000	-6.82080000
C	26.74200000	-25.64530000	-6.00450000
H	27.82690000	-25.53880000	-6.01870000

H	26.51810000	-26.59150000	-6.49710000
C	26.25490000	-25.69640000	-4.55030000
H	26.50770000	-24.76060000	-4.05090000
H	25.16710000	-25.76770000	-4.53270000
C	26.85520000	-26.87150000	-3.76750000
H	27.94330000	-26.82670000	-3.82120000
H	26.56130000	-27.80910000	-4.23920000
C	26.41740000	-26.87860000	-2.29680000
H	26.73450000	-25.95090000	-1.81940000
H	25.32860000	-26.89390000	-2.24130000
C	26.98470000	-28.07320000	-1.51920000
H	28.07180000	-28.07870000	-1.60240000
H	26.63640000	-28.99990000	-1.97540000
C	26.58520000	-28.05040000	-0.03770000
H	26.94700000	-27.13330000	0.42840000
H	25.49870000	-28.02700000	0.04860000
C	27.12830000	-29.25650000	0.73480000
H	28.21770000	-29.28030000	0.71230000
H	26.81810000	-29.22170000	1.77940000
H	26.76280000	-30.19220000	0.31250000
C	29.45460000	-23.08870000	-14.88190000
H	29.67890000	-23.98020000	-15.46950000
H	28.36770000	-23.01960000	-14.83200000
C	30.02210000	-23.25870000	-13.46640000
H	31.10690000	-23.35760000	-13.52210000
H	29.82320000	-22.35980000	-12.88250000
C	29.43020000	-24.47510000	-12.74190000
H	29.60440000	-25.37280000	-13.33650000
H	28.34900000	-24.35870000	-12.66770000
C	30.02140000	-24.67280000	-11.34010000
H	31.10000000	-24.81380000	-11.41930000
H	29.87020000	-23.76930000	-10.74930000
C	29.40070000	-25.86720000	-10.60450000
H	29.52220000	-26.76930000	-11.20520000
H	28.32770000	-25.70690000	-10.49940000
C	30.02260000	-26.09430000	-9.22090000
H	31.09030000	-26.28730000	-9.33160000
H	29.93270000	-25.18450000	-8.62780000
C	29.36640000	-27.25660000	-8.46520000
H	29.42450000	-28.16400000	-9.06750000
H	28.30640000	-27.04310000	-8.32760000
C	30.01850000	-27.51250000	-7.10090000
H	31.07000000	-27.76440000	-7.24380000
H	29.99800000	-26.59600000	-6.51190000
C	29.32310000	-28.63260000	-6.31690000
H	29.31320000	-29.54570000	-6.91330000
H	28.28090000	-28.36040000	-6.14950000
C	30.00130000	-28.91320000	-4.97030000
H	31.03260000	-29.22400000	-5.14160000
H	30.04930000	-27.99150000	-4.39130000
C	29.26940000	-29.98580000	-4.15360000
H	29.19700000	-30.90440000	-4.73730000
H	28.24710000	-29.65830000	-3.96380000
C	29.96670000	-30.28330000	-2.82010000
H	30.97840000	-30.64300000	-3.01210000
H	30.07130000	-29.35830000	-2.25400000
C	29.20670000	-31.31300000	-1.97390000
H	29.08510000	-32.23490000	-2.54420000
H	28.20200000	-30.94180000	-1.77000000
C	29.91540000	-31.62270000	-0.64850000
H	30.91480000	-32.01090000	-0.84950000
H	30.05210000	-30.70180000	-0.08060000
C	29.14410000	-32.63260000	0.20780000
H	29.01670000	-33.58020000	-0.31640000
H	29.67230000	-32.83720000	1.13930000
H	28.15410000	-32.25650000	0.46510000
O	27.51000000	-18.95390000	-21.45950000
O	26.54720000	-20.71410000	-22.51280000

O	27.86350000	-23.22190000	-21.53570000
H	26.92670000	-23.33200000	-21.68750000
C	27.00260000	-19.41050000	-22.66870000
C	25.82230000	-18.52330000	-23.08640000
H	26.15060000	-17.48670000	-23.17150000
H	25.43640000	-18.85250000	-24.05140000
H	25.01660000	-18.57890000	-22.35560000
C	28.12600000	-19.38840000	-23.70910000
H	27.76130000	-19.77880000	-24.65930000
H	28.47560000	-18.36610000	-23.85740000
H	28.96870000	-19.99530000	-23.38250000
C	26.96460000	-25.18780000	-17.57530000
C	24.15850000	-24.22110000	-18.03930000
O	27.71150000	-24.24390000	-17.32420000
O	23.36650000	-25.15250000	-17.91040000
N	26.66330000	-26.11770000	-16.66240000
N	24.19990000	-23.18560000	-17.19110000
H	26.01310000	-26.84060000	-16.93450000
H	24.86300000	-22.43560000	-17.39150000
C	26.30900000	-25.30360000	-18.96510000
H	27.10850000	-25.07170000	-19.67000000
C	25.19230000	-24.23890000	-19.17900000
H	25.67060000	-23.26380000	-19.17620000
C	25.82590000	-26.73690000	-19.29260000
H	26.64230000	-27.44860000	-19.15930000
H	25.02670000	-27.04710000	-18.61810000
C	24.48600000	-24.38650000	-20.54850000
H	23.56070000	-23.80690000	-20.53750000
C	25.32330000	-26.80990000	-20.73530000
H	26.16020000	-26.60840000	-21.40330000
C	24.17000000	-25.81930000	-20.99740000
H	23.24010000	-26.17230000	-20.54840000
C	23.41310000	-23.08180000	-15.96930000
H	23.44050000	-22.04670000	-15.62850000
H	22.36880000	-23.31820000	-16.18260000
C	27.15450000	-26.12300000	-15.29400000
H	28.22410000	-26.33850000	-15.29990000
H	27.03030000	-25.13170000	-14.85480000
C	23.96760000	-24.01650000	-14.88110000
H	23.95470000	-25.04260000	-15.25080000
H	25.01350000	-23.77290000	-14.69530000
C	23.18310000	-23.96060000	-13.56370000
H	23.24630000	-22.95760000	-13.14100000
H	22.12680000	-24.14970000	-13.75980000
C	23.70560000	-24.99210000	-12.55370000
H	24.77240000	-24.83330000	-12.39750000
H	23.60450000	-25.99150000	-12.97880000
C	22.98150000	-24.94560000	-11.20130000
H	23.10490000	-23.95820000	-10.75540000
H	21.91020000	-25.08510000	-11.35240000
C	23.50700000	-26.01920000	-10.23740000
H	24.58740000	-25.91430000	-10.13750000
H	23.33370000	-27.00620000	-10.66800000
C	22.86200000	-25.95130000	-8.84630000
H	23.05250000	-24.97220000	-8.40540000
H	21.77890000	-26.04270000	-8.93870000
C	23.39120000	-27.04840000	-7.91130000
H	24.47900000	-26.99020000	-7.86700000
H	23.15270000	-28.02670000	-8.33020000
C	22.82060000	-26.94960000	-6.48980000
H	23.07690000	-25.97910000	-6.06320000
H	21.73130000	-26.99120000	-6.52880000
C	23.34270000	-28.06580000	-5.57390000
H	24.43290000	-28.05300000	-5.57390000
H	23.04620000	-29.03460000	-5.97760000
C	22.83280000	-27.93610000	-4.13200000
H	23.14360000	-26.97370000	-3.72370000
H	21.74200000	-27.93390000	-4.12910000

C	23.34350000	-29.06480000	-3.22550000
H	24.43290000	-29.09150000	-3.26040000
H	22.99930000	-30.02520000	-3.61080000
C	22.88380000	-28.90380000	-1.76990000
H	23.23180000	-27.94420000	-1.38530000
H	21.79400000	-28.87330000	-1.73240000
C	23.39480000	-30.03140000	-0.86270000
H	24.48170000	-30.08380000	-0.92940000
H	23.01870000	-30.98930000	-1.22390000
C	22.97990000	-29.83860000	0.60270000
H	23.33440000	-28.87150000	0.96130000
H	21.89180000	-29.81280000	0.67520000
C	23.52260000	-30.94130000	1.51760000
H	24.61200000	-30.96860000	1.49750000
H	23.21500000	-30.77690000	2.55050000
H	23.15590000	-31.92230000	1.21530000
C	26.39630000	-27.16570000	-14.46290000
H	26.51640000	-28.15100000	-14.91590000
H	25.32870000	-26.94370000	-14.48610000
C	26.87220000	-27.21890000	-13.00600000
H	27.93540000	-27.46130000	-12.98410000
H	26.76950000	-26.23390000	-12.54980000
C	26.09350000	-28.24810000	-12.17600000
H	26.16700000	-29.22810000	-12.64970000
H	25.03460000	-27.98740000	-12.17290000
C	26.60440000	-28.34420000	-10.73310000
H	27.65950000	-28.61980000	-10.74410000
H	26.54530000	-27.36540000	-10.25670000
C	25.81980000	-29.36410000	-9.89800000
H	25.84700000	-30.33700000	-10.39080000
H	24.77070000	-29.06960000	-9.85650000
C	26.37610000	-29.50050000	-8.47500000
H	27.41920000	-29.81460000	-8.52610000
H	26.37100000	-28.52650000	-7.98570000
C	25.58420000	-30.50010000	-7.62250000
H	25.56230000	-31.46950000	-8.12220000
H	24.54790000	-30.16960000	-7.54560000
C	26.18080000	-30.66470000	-6.21840000
H	27.20770000	-31.02210000	-6.30330000
H	26.23280000	-29.69250000	-5.72830000
C	25.37490000	-31.63110000	-5.34140000
H	25.30150000	-32.59990000	-5.83740000
H	24.35470000	-31.26010000	-5.23800000
C	26.00090000	-31.81550000	-3.95240000
H	27.00900000	-32.21720000	-4.06100000
H	26.10720000	-30.84440000	-3.46880000
C	25.17560000	-32.74240000	-3.05080000
H	25.05340000	-33.71110000	-3.53730000
H	24.17320000	-32.32990000	-2.93040000
C	25.81960000	-32.94160000	-1.67180000
H	26.80870000	-33.38460000	-1.79460000
H	25.97390000	-31.97170000	-1.19870000
C	24.97230000	-33.82740000	-0.74900000
H	24.80900000	-34.79630000	-1.22300000
H	23.98680000	-33.37750000	-0.62190000
C	25.62370000	-34.03450000	0.62550000
H	26.59720000	-34.51060000	0.50150000
H	25.81270000	-33.06760000	1.09290000
C	24.76020000	-34.88610000	1.56220000
H	24.58270000	-35.87730000	1.14390000
H	25.24750000	-35.01670000	2.52880000
H	23.79120000	-34.41900000	1.74060000
O	25.26230000	-23.91700000	-21.63280000
O	24.04880000	-25.66960000	-22.40440000
O	24.85240000	-28.11560000	-20.98180000
H	24.42400000	-28.09800000	-21.82750000
C	24.69750000	-24.49250000	-22.76240000
C	23.66970000	-23.52390000	-23.36310000

H	24.15240000	-22.57710000	-23.60850000
H	23.24660000	-23.95120000	-24.27240000
H	22.85800000	-23.33390000	-22.66210000
C	25.82000000	-24.81650000	-23.75230000
H	25.40870000	-25.30380000	-24.63640000
H	26.32260000	-23.89770000	-24.05690000
H	26.55770000	-25.47600000	-23.29830000
C	32.60610000	-10.82460000	-15.84670000
C	29.60330000	-11.14440000	-15.83560000
O	32.81390000	-9.88140000	-15.08660000
O	29.03150000	-12.14410000	-16.27000000
N	33.03440000	-12.06590000	-15.57750000
N	29.66150000	-10.86810000	-14.52790000
H	32.75460000	-12.82840000	-16.18840000
H	30.19760000	-10.05540000	-14.25190000
C	31.77170000	-10.57570000	-17.11450000
H	32.24780000	-9.71610000	-17.58910000
C	30.31220000	-10.15300000	-16.78180000
H	30.35390000	-9.20000000	-16.25180000
C	31.82550000	-11.73180000	-18.13090000
H	32.85910000	-12.01790000	-18.33340000
H	31.32620000	-12.60730000	-17.72640000
C	29.48250000	-9.90230000	-18.06480000
H	28.42380000	-9.85310000	-17.80220000
C	31.14340000	-11.32260000	-19.43520000
H	31.70630000	-10.49820000	-19.86970000
C	29.66760000	-10.91730000	-19.20290000
H	29.03800000	-11.78870000	-19.03170000
C	29.16180000	-11.72650000	-13.46500000
H	28.96670000	-11.10070000	-12.59350000
H	28.20660000	-12.17220000	-13.74950000
C	33.84720000	-12.40500000	-14.41740000
H	34.87970000	-12.11290000	-14.61430000
H	33.51740000	-11.83140000	-13.54890000
C	30.18790000	-12.81590000	-13.11440000
H	30.32240000	-13.48660000	-13.96530000
H	31.15800000	-12.35200000	-12.93160000
C	29.77800000	-13.62160000	-11.87720000
H	29.62550000	-12.93470000	-11.04360000
H	28.81710000	-14.10450000	-12.05550000
C	30.81990000	-14.67490000	-11.47520000
H	31.79670000	-14.20090000	-11.37030000
H	30.92050000	-15.41930000	-12.26550000
C	30.44740000	-15.35830000	-10.15450000
H	30.36640000	-14.60010000	-9.37420000
H	29.45890000	-15.80880000	-10.24450000
C	31.45610000	-16.42690000	-9.71360000
H	32.45740000	-15.99580000	-9.67970000
H	31.48710000	-17.23130000	-10.44870000
C	31.09870000	-16.99370000	-8.33390000
H	31.10180000	-16.18240000	-7.60460000
H	30.07910000	-17.37940000	-8.35540000
C	32.04870000	-18.10120000	-7.86100000
H	33.07640000	-17.73660000	-7.87790000
H	32.00340000	-18.94250000	-8.55260000
C	31.69620000	-18.57470000	-6.44500000
H	31.77980000	-17.73360000	-5.75530000
H	30.65190000	-18.88730000	-6.41930000
C	32.58010000	-19.72650000	-5.94990000
H	33.62900000	-19.43250000	-6.00050000
H	32.46350000	-20.58510000	-6.61100000
C	32.23000000	-20.13220000	-4.51210000
H	32.38440000	-19.27910000	-3.85000000
H	31.16860000	-20.37560000	-4.45490000
C	33.05020000	-21.32550000	-4.00530000
H	34.11400000	-21.09700000	-4.07900000
H	32.87090000	-22.18750000	-4.64770000
C	32.70320000	-21.68610000	-2.55470000

H	32.91610000	-20.83280000	-1.90930000
H	31.63140000	-21.87040000	-2.47550000
C	33.46750000	-22.91510000	-2.04560000
H	34.54050000	-22.74040000	-2.13260000
H	33.23950000	-23.77120000	-2.68050000
C	33.12030000	-23.25270000	-0.58940000
H	33.37450000	-22.40910000	0.05360000
H	32.04380000	-23.39620000	-0.49480000
C	33.84110000	-24.50730000	-0.08610000
H	34.92350000	-24.38360000	-0.12480000
H	33.56790000	-24.72500000	0.94660000
H	33.58020000	-25.37840000	-0.68610000
C	33.76470000	-13.90430000	-14.10330000
H	34.12080000	-14.48080000	-14.95850000
H	32.72160000	-14.18460000	-13.95610000
C	34.57410000	-14.28000000	-12.85540000
H	35.62560000	-14.03930000	-13.01820000
H	34.24470000	-13.66680000	-12.01530000
C	34.43600000	-15.76220000	-12.48400000
H	34.77860000	-16.38290000	-13.31310000
H	33.38250000	-15.99870000	-12.33520000
C	35.22020000	-16.11740000	-11.21390000
H	36.28080000	-15.91610000	-11.37150000
H	34.90120000	-15.46610000	-10.39880000
C	35.03120000	-17.58080000	-10.79480000
H	35.36210000	-18.23950000	-11.59880000
H	33.96910000	-17.77810000	-10.65150000
C	35.78800000	-17.92130000	-9.50420000
H	36.85620000	-17.75700000	-9.65260000
H	35.47810000	-17.23740000	-8.71270000
C	35.54790000	-19.36620000	-9.04790000
H	35.86840000	-20.05650000	-9.82930000
H	34.47820000	-19.52630000	-8.91550000
C	36.27480000	-19.69690000	-7.73760000
H	37.34960000	-19.56910000	-7.87370000
H	35.97460000	-18.98440000	-6.96790000
C	35.98290000	-21.12250000	-7.25090000
H	36.28960000	-21.84050000	-8.01280000
H	34.90720000	-21.24560000	-7.12810000
C	36.68380000	-21.44740000	-5.92520000
H	37.76340000	-21.35420000	-6.05120000
H	36.39630000	-20.71090000	-5.17350000
C	36.34460000	-22.85420000	-5.41440000
H	36.63150000	-23.59460000	-6.16240000
H	35.26510000	-22.94180000	-5.29410000
C	37.03100000	-23.17820000	-4.08080000
H	38.11300000	-23.11270000	-4.20360000
H	36.75860000	-22.42490000	-3.34020000
C	36.65750000	-24.57010000	-3.55290000
H	36.92180000	-25.32540000	-4.29440000
H	35.57660000	-24.62920000	-3.42640000
C	37.34350000	-24.89720000	-2.21990000
H	38.42630000	-24.85080000	-2.34340000
H	37.08560000	-24.13920000	-1.47910000
C	36.95450000	-26.27940000	-1.68460000
H	37.22250000	-27.06740000	-2.38890000
H	37.46080000	-26.48930000	-0.74210000
H	35.88190000	-26.34270000	-1.50440000
O	29.81330000	-8.69040000	-18.71210000
O	29.22350000	-10.17400000	-20.32550000
O	31.22470000	-12.42280000	-20.31470000
H	30.35050000	-12.79680000	-20.41110000
C	29.31950000	-8.82480000	-20.00340000
C	27.91580000	-8.20780000	-20.07660000
H	27.95680000	-7.15610000	-19.79270000
H	27.53160000	-8.28480000	-21.09390000
H	27.22900000	-8.72680000	-19.40910000
C	30.29080000	-8.14330000	-20.97240000

H	29.93940000	-8.26450000	-21.99710000
H	30.35710000	-7.07960000	-20.74360000
H	31.28590000	-8.57630000	-20.89090000
C	31.40790000	-15.72570000	-17.11800000
C	28.40030000	-15.60340000	-17.20330000
O	31.88710000	-14.72170000	-16.59200000
O	27.87600000	-16.69860000	-17.40560000
N	31.49270000	-16.93450000	-16.54390000
N	28.28250000	-14.96140000	-16.03490000
H	31.04770000	-17.72930000	-16.99640000
H	28.68550000	-14.02710000	-15.97260000
C	30.65870000	-15.58540000	-18.45770000
H	31.26530000	-14.89140000	-19.04130000
C	29.25620000	-14.92900000	-18.29330000
H	29.41720000	-13.90500000	-17.96920000
C	30.60050000	-16.89800000	-19.26260000
H	31.59920000	-17.32620000	-19.36390000
H	29.99450000	-17.63190000	-18.73980000
C	28.48100000	-14.84320000	-19.63150000
H	27.42980000	-14.63690000	-19.42000000
C	30.00010000	-16.65500000	-20.64720000
H	30.65800000	-15.98230000	-21.19580000
C	28.57120000	-16.06900000	-20.54790000
H	27.85100000	-16.81940000	-20.23040000
C	27.64590000	-15.50770000	-14.84270000
H	27.48680000	-14.68930000	-14.14030000
H	26.65970000	-15.89970000	-15.09790000
C	32.16850000	-17.17940000	-15.27690000
H	33.24450000	-17.07230000	-15.42280000
H	31.87030000	-16.42190000	-14.55040000
C	28.51210000	-16.60370000	-14.19400000
H	28.63980000	-17.43240000	-14.89240000
H	29.50980000	-16.20680000	-14.00780000
C	27.92480000	-17.14150000	-12.88150000
H	27.81020000	-16.31950000	-12.17460000
H	26.92360000	-17.53360000	-13.06240000
C	28.79900000	-18.23800000	-12.25290000
H	29.81570000	-17.86360000	-12.13240000
H	28.86380000	-19.08790000	-12.93300000
C	28.26980000	-18.70800000	-10.89050000
H	28.21540000	-17.85650000	-10.21170000
H	27.24890000	-19.07340000	-11.00240000
C	29.13890000	-19.80470000	-10.25720000
H	30.17130000	-19.45990000	-10.19440000
H	29.14630000	-20.68370000	-10.90220000
C	28.64980000	-20.19670000	-8.85570000
H	28.67100000	-19.31950000	-8.20850000
H	27.60680000	-20.50850000	-8.91000000
C	29.48290000	-21.31760000	-8.21770000
H	30.53410000	-21.02890000	-8.20390000
H	29.41660000	-22.21560000	-8.83220000
C	29.02390000	-21.63640000	-6.78770000
H	29.12660000	-20.74470000	-6.16850000
H	27.96160000	-21.88080000	-6.79500000
C	29.80530000	-22.79230000	-6.14780000
H	30.87260000	-22.57110000	-6.17460000
H	29.66130000	-23.69770000	-6.73730000
C	29.37400000	-23.05060000	-4.69730000
H	29.55460000	-22.15550000	-4.10150000
H	28.29840000	-23.22590000	-4.66560000
C	30.10070000	-24.24270000	-4.05980000
H	31.17830000	-24.08890000	-4.12150000
H	29.88330000	-25.14600000	-4.62940000
C	29.69770000	-24.45460000	-2.59390000
H	29.94400000	-23.56220000	-2.01780000
H	28.61570000	-24.57060000	-2.52890000
C	30.37840000	-25.67560000	-1.96030000
H	31.46040000	-25.57800000	-2.05230000

H	30.10080000	-26.57240000	-2.51370000
C	30.00340000	-25.85360000	-0.48260000
H	30.29560000	-24.96720000	0.08090000
H	28.92030000	-25.93150000	-0.38720000
C	30.65520000	-27.08840000	0.14840000
H	31.74250000	-27.03320000	0.09450000
H	30.37850000	-27.17960000	1.19890000
H	30.33970000	-28.00200000	-0.35410000
C	31.83750000	-18.57590000	-14.73390000
H	32.14750000	-19.33160000	-15.45720000
H	30.75680000	-18.67580000	-14.63000000
C	32.50970000	-18.85600000	-13.38260000
H	33.59250000	-18.78800000	-13.49600000
H	32.22510000	-18.08210000	-12.66980000
C	32.13680000	-20.23070000	-12.81100000
H	32.40150000	-21.00840000	-13.52860000
H	31.05570000	-20.28410000	-12.68230000
C	32.82570000	-20.51690000	-11.46950000
H	33.90800000	-20.49230000	-11.60380000
H	32.58420000	-19.72330000	-10.76270000
C	32.41270000	-21.86800000	-10.87090000
H	32.63010000	-22.66510000	-11.58300000
H	31.33380000	-21.87590000	-10.71750000
C	33.11820000	-22.16680000	-9.54100000
H	34.19710000	-22.19390000	-9.69910000
H	32.92880000	-21.35350000	-8.84090000
C	32.65580000	-23.48920000	-8.91480000
H	32.82100000	-24.30490000	-9.61960000
H	31.58100000	-23.44460000	-8.73990000
C	33.37190000	-23.80460000	-7.59460000
H	34.44470000	-23.88820000	-7.77300000
H	33.23770000	-22.97340000	-6.90270000
C	32.85550000	-25.09340000	-6.94100000
H	32.96450000	-25.92560000	-7.63750000
H	31.78820000	-24.99240000	-6.74510000
C	33.58160000	-25.42770000	-5.63120000
H	34.64530000	-25.56520000	-5.82980000
H	33.50180000	-24.58320000	-4.94710000
C	33.01710000	-26.68280000	-4.95200000
H	33.07050000	-27.52660000	-5.64120000
H	31.96020000	-26.52860000	-4.73480000
C	33.75530000	-27.03770000	-3.65450000
H	34.80800000	-27.22050000	-3.87430000
H	33.72560000	-26.18550000	-2.97570000
C	33.15430000	-28.26420000	-2.95480000
H	33.15810000	-29.11290000	-3.64020000
H	32.10940000	-28.06620000	-2.71540000
C	33.90720000	-28.64170000	-1.67220000
H	34.95090000	-28.85370000	-1.90830000
H	33.91280000	-27.79510000	-0.98540000
C	33.29140000	-29.85450000	-0.96680000
H	33.29230000	-30.73220000	-1.61380000
H	33.84980000	-30.10600000	-0.06480000
H	32.26100000	-29.65610000	-0.67280000
O	28.94260000	-13.82070000	-20.49160000
O	28.21350000	-15.51860000	-21.80480000
O	29.97300000	-17.89750000	-21.31490000
H	29.05970000	-18.13680000	-21.46280000
C	28.44520000	-14.14980000	-21.74660000
C	27.11620000	-13.41580000	-21.96990000
H	27.26910000	-12.33930000	-21.88560000
H	26.73270000	-13.64160000	-22.96510000
H	26.37310000	-13.72500000	-21.23600000
C	29.48730000	-13.76990000	-22.80240000
H	29.13350000	-14.05370000	-23.79350000
H	29.66080000	-12.69340000	-22.78380000
H	30.43340000	-14.27390000	-22.61200000

3 hexamer

C	32.87380	-10.45260	-15.83000
C	29.92380	-11.05040	-16.26670
O	33.00820	-9.46960	-15.10310
O	29.64750	-12.15320	-16.73520
N	33.25980	-11.68160	-15.45340
N	29.61640	-10.70440	-15.00870
H	33.09650	-12.45510	-16.08570
H	29.84460	-9.76430	-14.70960
C	32.20630	-10.28060	-17.21060
H	32.66250	-9.37890	-17.62250
C	30.67810	-10.00310	-17.11150
H	30.55500	-9.04780	-16.60720
C	32.52190	-11.42120	-18.20010
H	33.59770	-11.60010	-18.24140
H	32.06170	-12.35020	-17.87040
C	30.02450	-9.84100	-18.50590
H	28.93930	-9.90330	-18.40170
C	32.00770	-11.07030	-19.59940
H	32.54440	-10.19020	-19.95050
C	30.48080	-10.82120	-19.59680
H	29.93050	-11.75720	-19.51290
C	29.04500	-11.60320	-14.01350
H	28.66070	-10.99410	-13.19550
H	28.19100	-12.13600	-14.43500
C	33.87740	-11.99070	-14.17070
H	34.90090	-11.61340	-14.17020
H	33.34610	-11.47380	-13.37000
C	30.09810	-12.59590	-13.48870
H	30.43760	-13.23870	-14.30240
H	30.97380	-12.04150	-13.15200
C	29.58100	-13.46930	-12.33790
H	29.24400	-12.82540	-11.52520
H	28.70700	-14.03230	-12.66640
C	30.64920	-14.43950	-11.81100
H	31.54710	-13.87840	-11.55180
H	30.93730	-15.13260	-12.60200
C	30.17560	-15.22590	-10.58080
H	29.89480	-14.52440	-9.79480
H	29.27240	-15.78370	-10.82830
C	31.24010	-16.19120	-10.03920
H	32.16410	-15.64380	-9.85220
H	31.47200	-16.94230	-10.79480
C	30.79010	-16.88040	-8.74350
H	30.58080	-16.12230	-7.98850
H	29.84960	-17.40260	-8.91820
C	31.82630	-17.86960	-8.19160
H	32.78440	-17.36390	-8.07030
H	31.98610	-18.67050	-8.91360
C	31.39270	-18.46600	-6.84520
H	31.26850	-17.66240	-6.11910
H	30.41260	-18.92970	-6.95490
C	32.38380	-19.49940	-6.29240
H	33.37630	-19.05380	-6.22500
H	32.46350	-20.33430	-6.98840
C	31.96380	-20.02040	-4.91080
H	31.92320	-19.18810	-4.20810
H	30.95110	-20.41860	-4.96840
C	32.90380	-21.10280	-4.36290
H	33.92460	-20.72110	-4.33840
H	32.90690	-21.95670	-5.04010
C	32.49780	-21.56720	-2.95720
H	32.52650	-20.71880	-2.27340
H	31.46310	-21.90880	-2.97470
C	33.39360	-22.68920	-2.41530

H	34.43360	-22.36230	-2.42710
H	33.33230	-23.55290	-3.07680
C	33.00530	-23.11150	-0.99150
H	33.08120	-22.25570	-0.32060
H	31.96030	-23.42020	-0.97450
C	33.87600	-24.25070	-0.45250
H	34.92910	-23.96970	-0.43740
H	33.58620	-24.51240	0.56520
H	33.77460	-25.14650	-1.06360
C	33.86500	-13.50270	-13.90700
H	34.39910	-14.01840	-14.70620
H	32.83800	-13.86750	-13.93780
C	34.49500	-13.86690	-12.55610
H	35.53240	-13.53070	-12.53910
H	33.98260	-13.32430	-11.76170
C	34.43710	-15.37180	-12.25810
H	34.93230	-15.92340	-13.05820
H	33.39740	-15.69910	-12.25530
C	35.08860	-15.72470	-10.91380
H	36.13780	-15.42760	-10.93090
H	34.61670	-15.14300	-10.12240
C	34.98330	-17.21680	-10.57120
H	35.43480	-17.80910	-11.36780
H	33.93220	-17.50190	-10.53100
C	35.65660	-17.55950	-9.23500
H	36.71700	-17.31010	-9.28840
H	35.23200	-16.93650	-8.44820
C	35.49440	-19.03550	-8.84830
H	35.89490	-19.66860	-9.64080
H	34.43290	-19.26930	-8.77170
C	36.18700	-19.37760	-7.52200
H	37.25670	-19.18400	-7.60980
H	35.81570	-18.71540	-6.74030
C	35.96000	-20.83390	-7.09540
H	36.30510	-21.50470	-7.88280
H	34.89040	-21.00990	-6.98560
C	36.66860	-21.18350	-5.77990
H	37.74420	-21.04850	-5.89930
H	36.35330	-20.48670	-5.00370
C	36.37670	-22.61700	-5.31710
H	36.66460	-23.32020	-6.09940
H	35.30270	-22.73430	-5.17600
C	37.10120	-22.97910	-4.01380
H	38.17850	-22.89660	-4.16260
H	36.83940	-22.25590	-3.24190
C	36.75510	-24.39070	-3.52130
H	36.99050	-25.11680	-4.30040
H	35.68080	-24.45760	-3.35120
C	37.49710	-24.76770	-2.23200
H	38.57340	-24.71940	-2.40150
H	37.27410	-24.03880	-1.45280
C	37.12640	-26.16610	-1.72820
H	37.35610	-26.92900	-2.47260
H	37.67640	-26.41090	-0.81930
H	36.06310	-26.23030	-1.49900
O	30.31600	-8.59870	-19.10620
O	30.12250	-10.11920	-20.77620
O	32.31980	-12.14760	-20.45550
H	31.57780	-12.73770	-20.48030
C	30.02430	-8.76950	-20.45360
C	28.58520	-8.30150	-20.72400
H	28.47630	-7.25420	-20.43990
H	28.35560	-8.40510	-21.78480
H	27.87010	-8.89630	-20.15670
C	31.04270	-7.97890	-21.28430
H	30.85610	-8.13290	-22.34730
H	30.95730	-6.91470	-21.06230
H	32.05810	-8.29960	-21.05680

C	32.81030	-5.57340	-13.68140
C	29.95060	-6.58290	-14.13230
O	32.78510	-4.72430	-12.79170
O	29.69710	-7.57270	-14.81650
N	33.46450	-6.73770	-13.54770
N	29.79100	-6.56640	-12.80180
H	33.41550	-7.41160	-14.30130
H	30.02580	-5.71430	-12.31190
C	32.03250	-5.30980	-14.98510
H	32.32350	-4.29630	-15.26690
C	30.49110	-5.28800	-14.77520
H	30.25730	-4.47910	-14.08050
C	32.44100	-6.22390	-16.15840
H	33.52690	-6.24520	-16.26580
H	32.12310	-7.24810	-15.97360
C	29.75000	-4.96270	-16.09660
H	28.68740	-5.18410	-15.97690
C	31.81190	-5.71970	-17.45990
H	32.22340	-4.73540	-17.67690
C	30.26830	-5.66140	-17.36630
H	29.83500	-6.65750	-17.45510
C	29.42810	-7.71640	-11.98560
H	29.00890	-7.34060	-11.05180
H	28.64200	-8.29920	-12.46910
C	34.23020	-7.11270	-12.36690
H	35.17620	-6.56960	-12.37300
H	33.69440	-6.81020	-11.46540
C	30.65670	-8.59430	-11.69580
H	31.02340	-9.03870	-12.62270
H	31.46310	-7.96710	-11.31390
C	30.35860	-9.69820	-10.67430
H	29.97190	-9.23920	-9.76350
H	29.56690	-10.34630	-11.05010
C	31.59400	-10.54050	-10.32590
H	32.41060	-9.88120	-10.02950
H	31.93700	-11.08040	-11.20860
C	31.30710	-11.52570	-9.18640
H	30.97640	-10.96510	-8.31100
H	30.47670	-12.17480	-9.46410
C	32.52010	-12.38400	-8.80400
H	33.37550	-11.73870	-8.60170
H	32.80090	-13.02130	-9.64250
C	32.23220	-13.24470	-7.56760
H	31.98400	-12.59050	-6.73080
H	31.34820	-13.85610	-7.74910
C	33.40350	-14.15140	-7.16900
H	34.30630	-13.55200	-7.04840
H	33.60460	-14.86350	-7.96920
C	33.11510	-14.90510	-5.86460
H	32.95760	-14.18270	-5.06260
H	32.18070	-15.45740	-5.96570
C	34.23470	-15.87340	-5.46200
H	35.18050	-15.33430	-5.40160
H	34.35560	-16.63240	-6.23490
C	33.94580	-16.54990	-4.11570
H	33.87010	-15.78690	-3.33980
H	32.97260	-17.03890	-4.15960
C	35.01200	-17.57810	-3.71620
H	35.99150	-17.09980	-3.69640
H	35.06200	-18.36170	-4.47220
C	34.72210	-18.20510	-2.34590
H	34.71260	-17.42270	-1.58610
H	33.72170	-18.63820	-2.35230
C	35.73980	-19.28460	-1.95440
H	36.74330	-18.85850	-1.95910
H	35.73410	-20.07620	-2.70360
C	35.44690	-19.88700	-0.57360
H	35.48380	-19.10270	0.18320

H	34.43140	-20.28210	-0.55630
C	36.42670	-21.00160	-0.19400
H	37.45130	-20.63120	-0.16130
H	36.18870	-21.41060	0.78800
H	36.38710	-21.82080	-0.91080
C	34.48430	-8.62550	-12.32950
H	35.03650	-8.92980	-13.21970
H	33.53160	-9.15450	-12.36090
C	35.25890	-9.04740	-11.07450
H	36.22840	-8.54780	-11.06260
H	34.72370	-8.70110	-10.18930
C	35.46230	-10.56540	-10.97780
H	36.00430	-10.92140	-11.85480
H	34.49190	-11.06150	-10.99100
C	36.22160	-10.96270	-9.70490
H	37.20730	-10.49560	-9.70990
H	35.69760	-10.56570	-8.83440
C	36.37510	-12.48110	-9.55000
H	36.90120	-12.88840	-10.41420
H	35.38810	-12.94310	-9.54220
C	37.12440	-12.85910	-8.26520
H	38.12650	-12.42910	-8.28890
H	36.61790	-12.41330	-7.40800
C	37.22140	-14.37620	-8.06020
H	37.72850	-14.83100	-8.91210
H	36.21750	-14.79900	-8.03660
C	37.95800	-14.74480	-6.76550
H	38.97650	-14.35630	-6.80270
H	37.47130	-14.25480	-5.92110
C	37.99360	-16.25870	-6.51910
H	38.47910	-16.75640	-7.35950
H	36.97330	-16.63910	-6.48340
C	38.71700	-16.62530	-5.21640
H	39.74980	-16.27790	-5.26370
H	38.25140	-16.09730	-4.38310
C	38.69370	-18.13380	-4.93670
H	39.15350	-18.66810	-5.76910
H	37.65940	-18.47280	-4.88730
C	39.41090	-18.50220	-3.63100
H	40.45420	-18.18960	-3.68940
H	38.96750	-17.94560	-2.80440
C	39.34130	-20.00500	-3.32710
H	39.77380	-20.56620	-4.15640
H	38.29730	-20.30990	-3.26050
C	40.06290	-20.37690	-2.02480
H	41.11260	-20.08890	-2.09270
H	39.63980	-19.80790	-1.19620
C	39.96870	-21.87320	-1.70870
H	40.40620	-22.47460	-2.50600
H	40.49600	-22.11010	-0.78450
H	38.93130	-22.18270	-1.58660
O	29.85650	-3.60180	-16.45520
O	29.78200	-4.79910	-18.38190
O	32.20310	-6.59470	-18.49540
H	31.57800	-7.30620	-18.54310
C	29.53670	-3.55630	-17.80600
C	28.04230	-3.23240	-17.96420
H	27.81630	-2.27660	-17.49080
H	27.78700	-3.17120	-19.02240
H	27.42800	-4.00670	-17.50540
C	30.42250	-2.50330	-18.48400
H	30.21160	-2.47200	-19.55320
H	30.22470	-1.52040	-18.05580
H	31.47630	-2.73680	-18.34160
C	24.97900	-29.79150	-17.81890
C	22.34220	-28.57890	-18.72790
O	25.79520	-28.89050	-17.63390
O	21.38760	-29.34830	-18.62370

N	24.52290	-30.56120	-16.82150
N	22.46960	-27.47150	-17.98200
H	23.84210	-31.27170	-17.04830
H	23.27020	-26.87480	-18.15540
C	24.41730	-30.03630	-19.23570
H	25.29360	-30.02710	-19.88560
C	23.48810	-28.88260	-19.71340
H	24.09430	-27.98140	-19.77820
C	23.75270	-31.42230	-19.40770
H	24.43410	-32.20840	-19.07810
H	22.86150	-31.50630	-18.78450
C	22.93500	-29.13770	-21.13420
H	22.12350	-28.43550	-21.33570
C	23.37770	-31.66690	-20.87450
H	24.29530	-31.73140	-21.45740
C	22.45330	-30.56440	-21.43880
H	21.42670	-30.70600	-21.09800
C	21.59990	-27.11130	-16.87010
H	21.75430	-26.05630	-16.64310
H	20.55410	-27.22220	-17.16180
C	24.90050	-30.42120	-15.42360
H	25.92810	-30.76430	-15.29530
H	24.87330	-29.36950	-15.13370
C	21.91510	-27.97200	-15.63570
H	21.77620	-29.02470	-15.88450
H	22.96770	-27.85660	-15.37700
C	21.04960	-27.63510	-14.41490
H	21.21830	-26.59860	-14.12210
H	19.99470	-27.71560	-14.68030
C	21.35280	-28.56970	-13.23550
H	22.41730	-28.52350	-13.00640
H	21.14810	-29.59890	-13.53260
C	20.54790	-28.24130	-11.97080
H	20.76730	-27.22110	-11.65470
H	19.48100	-28.27320	-12.19520
C	20.86110	-29.21700	-10.82730
H	21.93610	-29.22460	-10.64700
H	20.59460	-30.22900	-11.13420
C	20.13260	-28.87460	-9.52040
H	20.41250	-27.87000	-9.20180
H	19.05610	-28.85480	-9.69470
C	20.45290	-29.87720	-8.40240
H	21.53340	-29.93530	-8.27030
H	20.12610	-30.87250	-8.70520
C	19.79840	-29.51250	-7.06270
H	20.13860	-28.52450	-6.75060
H	18.71770	-29.44040	-7.19100
C	20.11600	-30.53520	-5.96230
H	21.19700	-30.64090	-5.86960
H	19.73450	-31.51350	-6.25630
C	19.52520	-30.14640	-4.60000
H	19.91750	-29.17410	-4.29990
H	18.44480	-30.02820	-4.69090
C	19.83580	-31.18130	-3.50940
H	20.91430	-31.32800	-3.44830
H	19.40850	-32.14440	-3.79000
C	19.29940	-30.76730	-2.13200
H	19.72940	-29.80510	-1.85150
H	18.22070	-30.61720	-2.19070
C	19.61270	-31.80210	-1.04240
H	20.68800	-31.97760	-1.01050
H	19.15270	-32.75580	-1.30250
C	19.12490	-31.36180	0.34500
H	19.56590	-30.39780	0.60070
H	18.04520	-31.20970	0.32230
C	19.47000	-32.37610	1.44010
H	20.54730	-32.52480	1.51260
H	19.11650	-32.03380	2.41280

H	19.00890	-33.34360	1.24150
C	23.94720	-31.23300	-14.53850
H	23.96970	-32.27970	-14.84530
H	22.92450	-30.88860	-14.69490
C	24.29470	-31.13530	-13.04830
H	25.31280	-31.49360	-12.89300
H	24.28260	-30.09070	-12.73620
C	23.32760	-31.94190	-12.17130
H	23.31460	-32.98000	-12.50590
H	22.31380	-31.56330	-12.30460
C	23.70400	-31.89470	-10.68540
H	24.71470	-32.28430	-10.56030
H	23.72720	-30.85900	-10.34630
C	22.73650	-32.69680	-9.80520
H	22.68660	-33.72580	-10.16340
H	21.73110	-32.28700	-9.90380
C	23.15480	-32.69350	-8.32910
H	24.15380	-33.12110	-8.23940
H	23.22580	-31.66600	-7.97270
C	22.18500	-33.47450	-7.43310
H	22.09080	-34.49670	-7.80160
H	21.19190	-33.02970	-7.50010
C	22.64270	-33.50170	-5.96850
H	23.62460	-33.97210	-5.90790
H	22.76710	-32.48120	-5.60660
C	21.66370	-34.24690	-5.05230
H	21.51980	-35.26290	-5.42190
H	20.68810	-33.76210	-5.09570
C	22.15130	-34.29830	-3.59770
H	23.11190	-34.81290	-3.55760
H	22.32940	-33.28530	-3.23670
C	21.15670	-35.00040	-2.66430
H	20.96290	-36.00920	-3.03070
H	20.20230	-34.47380	-2.69280
C	21.66250	-35.07240	-1.21670
H	22.59960	-35.62950	-1.18860
H	21.89100	-34.06810	-0.85980
C	20.64850	-35.72860	-0.27020
H	20.41080	-36.73080	-0.62920
H	19.71570	-35.16420	-0.29350
C	21.16160	-35.81280	1.17420
H	22.07780	-36.40370	1.20310
H	21.42740	-34.81680	1.52880
C	20.13310	-36.42820	2.12890
H	19.87130	-37.44240	1.82600
H	20.52590	-36.47560	3.14480
H	19.21660	-35.83810	2.15460
O	23.91290	-28.94800	-22.13160
O	22.53680	-30.59600	-22.85620
O	22.70890	-32.90750	-20.95250
H	22.43270	-33.03290	-21.84870
C	23.41850	-29.59490	-23.25480
C	22.65810	-28.58400	-24.12810
H	23.32260	-27.76840	-24.41550
H	22.29180	-29.07530	-25.02990
H	21.80570	-28.17010	-23.59020
C	24.59390	-30.22720	-24.01040
H	24.22790	-30.77540	-24.87880
H	25.27910	-29.44870	-24.34780
H	25.14250	-30.91280	-23.36650
C	28.26080	-25.54390	-18.25280
C	25.42560	-24.72250	-18.97680
O	28.92140	-24.55730	-17.93060
O	24.60280	-25.63510	-19.02400
N	27.95760	-26.51780	-17.38130
N	25.39230	-23.76720	-18.03670
H	27.38780	-27.29130	-17.70010
H	26.08470	-23.02920	-18.09070

C	27.72760	-25.65810	-19.69720
H	28.57300	-25.36770	-20.32280
C	26.57920	-24.65130	-19.99810
H	26.99850	-23.65030	-19.92280
C	27.35250	-27.09760	-20.10890
H	28.16850	-27.78380	-19.87600
H	26.48560	-27.43820	-19.54790
C	26.04840	-24.79140	-21.44660
H	25.09900	-24.25890	-21.53380
C	27.03860	-27.16520	-21.60650
H	27.94290	-26.90960	-22.15650
C	25.87450	-26.21980	-21.98540
H	24.91700	-26.62970	-21.66590
C	24.49290	-23.75270	-16.89010
H	24.49190	-22.74420	-16.47630
H	23.47100	-23.96290	-17.21040
C	28.36860	-26.52640	-15.98400
H	29.43940	-26.72770	-15.93030
H	28.20630	-25.54160	-15.54270
C	24.94660	-24.76290	-15.82240
H	24.92000	-25.77130	-16.23830
H	25.98690	-24.56510	-15.56350
C	24.09390	-24.72350	-14.54750
H	24.12690	-23.71990	-14.12280
H	23.05090	-24.92000	-14.79670
C	24.57390	-25.74120	-13.50240
H	25.63170	-25.57420	-13.29900
H	24.49640	-26.74770	-13.91380
C	23.79060	-25.66670	-12.18480
H	23.88540	-24.66530	-11.76440
H	22.72900	-25.81760	-12.37930
C	24.27520	-26.70140	-11.15930
H	25.34670	-26.57850	-11.00260
H	24.13700	-27.70500	-11.56160
C	23.55080	-26.58700	-9.81120
H	23.71310	-25.59170	-9.39710
H	22.47590	-26.68210	-9.96390
C	24.01830	-27.64420	-8.80150
H	25.10080	-27.58260	-8.68880
H	23.80530	-28.63920	-9.19220
C	23.35570	-27.48120	-7.42710
H	23.59370	-26.49610	-7.02540
H	22.27190	-27.51130	-7.53820
C	23.79630	-28.56020	-6.42890
H	24.88400	-28.56460	-6.35640
H	23.50790	-29.54170	-6.80500
C	23.19540	-28.34980	-5.03290
H	23.50820	-27.37940	-4.64660
H	22.10840	-28.31310	-5.10570
C	23.60350	-29.44900	-4.04330
H	24.69080	-29.51720	-4.00440
H	23.24600	-30.41300	-4.40530
C	23.05900	-29.19810	-2.63100
H	23.43850	-28.24570	-2.25990
H	21.97410	-29.09950	-2.67140
C	23.43350	-30.31500	-1.64820
H	24.51670	-30.43700	-1.63220
H	23.02150	-31.26050	-2.00140
C	22.93050	-30.03750	-0.22520
H	23.35710	-29.10320	0.14080
H	21.85030	-29.89410	-0.24040
C	23.27730	-31.16600	0.75050
H	24.35620	-31.29780	0.83010
H	22.89360	-30.94990	1.74770
H	22.84500	-32.11270	0.42700
C	27.58550	-27.58450	-15.19600
H	27.74960	-28.56650	-15.64180
H	26.51750	-27.38330	-15.28130

C	27.98010	-27.62710	-13.71400
H	29.04370	-27.85370	-13.63140
H	27.83960	-26.64090	-13.27070
C	27.17120	-28.66130	-12.91920
H	27.28810	-29.64470	-13.37600
H	26.11090	-28.41630	-12.98160
C	27.59610	-28.73180	-11.44660
H	28.65180	-28.99900	-11.38990
H	27.50230	-27.74510	-10.99330
C	26.76930	-29.73970	-10.63770
H	26.83550	-30.72350	-11.10320
H	25.71880	-29.45130	-10.66900
C	27.23130	-29.83880	-9.17820
H	28.27390	-30.15710	-9.15200
H	27.19770	-28.85110	-8.71950
C	26.37930	-30.80780	-8.34870
H	26.38340	-31.79220	-8.81760
H	25.34410	-30.46700	-8.34950
C	26.87670	-30.93350	-6.90300
H	27.90010	-31.31010	-6.90550
H	26.91190	-29.94530	-6.44570
C	25.99450	-31.85180	-6.04800
H	25.93030	-32.83560	-6.51400
H	24.98040	-31.45320	-6.02240
C	26.52230	-32.00180	-4.61540
H	27.52210	-32.43650	-4.64220
H	26.62640	-31.01580	-4.16370
C	25.61050	-32.86550	-3.73490
H	25.48140	-33.84630	-4.19390
H	24.62050	-32.41160	-3.68950
C	26.16010	-33.03730	-2.31280
H	27.13580	-33.52270	-2.35770
H	26.32440	-32.05630	-1.86800
C	25.22250	-33.85130	-1.41160
H	25.04100	-34.82780	-1.86220
H	24.25480	-33.35260	-1.35530
C	25.78320	-34.03980	0.00450
H	26.74070	-34.55940	-0.04760
H	25.98560	-33.06650	0.45200
C	24.83170	-34.82400	0.91410
H	24.63090	-35.81760	0.51270
H	25.25850	-34.94720	1.90960
H	23.87820	-34.30780	1.02420
O	26.92760	-24.24040	-22.40080
O	25.89570	-25.99890	-23.38700
O	26.69390	-28.49820	-21.91840
H	25.75370	-28.59300	-21.83930
C	26.53910	-24.78690	-23.61590
C	25.55720	-23.82980	-24.31060
H	26.03020	-22.85870	-24.46140
H	25.26770	-24.23590	-25.28010
H	24.65780	-23.69380	-23.71090
C	27.79120	-25.03160	-24.46720
H	27.51340	-25.49460	-25.41430
H	28.29220	-24.08460	-24.67070
H	28.48910	-25.68650	-23.94810
C	30.70170	-20.71470	-18.08770
C	27.74070	-20.35640	-18.66560
O	31.16940	-19.67220	-17.63160
O	27.09720	-21.39050	-18.83670
N	30.62190	-21.84130	-17.36370
N	27.56670	-19.56630	-17.59660
H	30.20370	-22.65940	-17.78830
H	28.11050	-18.71270	-17.54900
C	30.15080	-20.72870	-19.52950
H	30.90500	-20.19940	-20.11410
C	28.82440	-19.92750	-19.67610
H	29.05130	-18.88520	-19.46400

C	30.03850	-22.14300	-20.13700
H	30.97760	-22.68490	-20.01200
H	29.27300	-22.71860	-19.62140
C	28.27900	-19.96500	-21.12540
H	27.24440	-19.61590	-21.12790
C	29.69060	-22.06100	-21.62610
H	30.51030	-21.56260	-22.14130
C	28.35640	-21.31310	-21.85670
H	27.50500	-21.93470	-21.58430
C	26.71680	-19.87870	-16.45480
H	26.56060	-18.95860	-15.89150
H	25.73450	-20.20900	-16.79690
C	31.07250	-21.96530	-15.98460
H	32.16320	-21.97590	-15.96740
H	30.75220	-21.09540	-15.40870
C	27.36670	-20.94600	-15.55710
H	27.48890	-21.87330	-16.11900
H	28.36990	-20.61910	-15.28280
C	26.56450	-21.23380	-14.28150
H	26.45410	-20.31070	-13.71180
H	25.55690	-21.55690	-14.54470
C	27.23440	-22.30240	-13.40520
H	28.26320	-22.00530	-13.20130
H	27.28850	-23.24230	-13.95550
C	26.50420	-22.52750	-12.07450
H	26.46030	-21.58670	-11.52530
H	25.47140	-22.81760	-12.26730
C	27.18240	-23.59490	-11.20350
H	28.22960	-23.32980	-11.05760
H	27.17590	-24.55100	-11.72740
C	26.50670	-23.75350	-9.83470
H	26.53450	-22.80020	-9.30680
H	25.45280	-23.99280	-9.97450
C	27.16460	-24.83540	-8.96670
H	28.22950	-24.62400	-8.86850
H	27.08530	-25.80080	-9.46650
C	26.53410	-24.92580	-7.57030
H	26.64140	-23.96690	-7.06300
H	25.46260	-25.09940	-7.66660
C	27.15250	-26.03190	-6.70440
H	28.23180	-25.88950	-6.64730
H	26.99190	-26.99830	-7.18190
C	26.56600	-26.05660	-5.28630
H	26.75610	-25.10000	-4.79940
H	25.48240	-26.15740	-5.34430
C	27.13590	-27.19010	-4.42310
H	28.22370	-27.12010	-4.40010
H	26.89630	-28.14920	-4.88160
C	26.59180	-27.15920	-2.98830
H	26.85900	-26.21180	-2.51960
H	25.50260	-27.19000	-3.01330
C	27.11310	-28.31870	-2.12900
H	28.20340	-28.31260	-2.13050
H	26.80780	-29.26470	-2.57560
C	26.60310	-28.25000	-0.68310
H	26.92500	-27.31490	-0.22400
H	25.51330	-28.23230	-0.68030
C	27.09020	-29.42420	0.17140
H	28.17890	-29.45210	0.21770
H	26.71500	-29.34420	1.19170
H	26.74500	-30.37510	-0.23310
C	30.51480	-23.24410	-15.34650
H	30.83590	-24.11120	-15.92540
H	29.42570	-23.22910	-15.39480
C	30.95920	-23.41380	-13.88830
H	32.04810	-23.45950	-13.84710
H	30.66570	-22.53510	-13.31330
C	30.36670	-24.66930	-13.23430

H	30.63250	-25.54840	-13.82280
H	29.27870	-24.60570	-13.24980
C	30.85010	-24.85710	-11.79060
H	31.93670	-24.95030	-11.78400
H	30.61310	-23.96580	-11.20970
C	30.22660	-26.08230	-11.11000
H	30.43320	-26.97510	-11.70110
H	29.14310	-25.96830	-11.08840
C	30.74960	-26.28480	-9.68220
H	31.82930	-26.43490	-9.71130
H	30.57860	-25.37800	-9.10290
C	30.08560	-27.46850	-8.96810
H	30.22590	-28.37710	-9.55450
H	29.01110	-27.29570	-8.91370
C	30.64000	-27.68420	-7.55440
H	31.70760	-27.89850	-7.61300
H	30.54000	-26.76140	-6.98390
C	29.92810	-28.81660	-6.80400
H	29.99880	-29.74020	-7.37940
H	28.86750	-28.58000	-6.72390
C	30.50590	-29.04400	-5.40160
H	31.55720	-29.32200	-5.48340
H	30.47560	-28.10830	-4.84460
C	29.74760	-30.12120	-4.61600
H	29.75250	-31.05630	-5.17710
H	28.70390	-29.82380	-4.51720
C	30.34220	-30.35860	-3.22210
H	31.37700	-30.68880	-3.32070
H	30.36960	-29.41530	-2.67770
C	29.54830	-31.38920	-2.40860
H	29.49980	-32.32940	-2.95910
H	28.52080	-31.04390	-2.29540
C	30.15570	-31.64010	-1.02180
H	31.17920	-32.00070	-1.13060
H	30.21670	-30.70030	-0.47230
C	29.34860	-32.65140	-0.20120
H	29.28860	-33.61450	-0.70870
H	29.80800	-32.81660	0.77350
H	28.33190	-32.29780	-0.03230
O	29.00440	-19.13330	-22.00250
O	28.28210	-20.90540	-23.21370
O	29.59500	-23.37890	-22.12160
H	28.68690	-23.64980	-22.08420
C	28.67850	-19.57360	-23.27800
C	27.50930	-18.73560	-23.81990
H	27.78650	-17.68100	-23.83860
H	27.26420	-19.05430	-24.83330
H	26.62370	-18.85580	-23.19670
C	29.92050	-19.45820	-24.17040
H	29.69770	-19.83330	-25.16950
H	30.22750	-18.41450	-24.24560
H	30.74790	-20.03250	-23.75680
C	32.22560	-15.59000	-17.29250
C	29.22880	-15.71650	-17.77990
O	32.51240	-14.54970	-16.70180
O	28.77230	-16.81500	-18.09150
N	32.38000	-16.79890	-16.73140
N	28.95990	-15.13050	-16.60450
H	32.10150	-17.61580	-17.26030
H	29.34570	-14.20820	-16.43940
C	31.63250	-15.51130	-18.71510
H	32.25400	-14.77600	-19.22860
C	30.17730	-14.95930	-18.73230
H	30.21720	-13.93160	-18.37900
C	31.75850	-16.82580	-19.51340
H	32.78480	-17.19520	-19.47620
H	31.13070	-17.59880	-19.07540
C	29.59430	-14.90000	-20.16590

H	28.51380	-14.75380	-20.10740
C	31.34690	-16.60740	-20.97220
H	32.04140	-15.89880	-21.42080
C	29.89020	-16.09800	-21.07990
H	29.17810	-16.89850	-20.88660
C	28.22100	-15.74950	-15.51150
H	27.93060	-14.96210	-14.81590
H	27.29760	-16.19400	-15.88670
C	32.89730	-17.02160	-15.38840
H	33.97160	-16.83230	-15.38630
H	32.44630	-16.31120	-14.69360
C	29.07770	-16.80500	-14.79040
H	29.33070	-17.60910	-15.48300
H	30.02210	-16.35230	-14.48830
C	28.38840	-17.40220	-13.55660
H	28.14710	-16.60010	-12.85850
H	27.43970	-17.85430	-13.84690
C	29.26280	-18.45030	-12.85230
H	30.23530	-18.01250	-12.62640
H	29.45020	-19.28370	-13.53020
C	28.63450	-18.97360	-11.55370
H	28.45770	-18.13590	-10.87860
H	27.65680	-19.40560	-11.76740
C	29.51250	-20.01770	-10.84870
H	30.50830	-19.60400	-10.68910
H	29.63790	-20.88630	-11.49590
C	28.92740	-20.45870	-9.50000
H	28.82290	-19.58800	-8.85230
H	27.92060	-20.84790	-9.65050
C	29.78300	-21.51820	-8.79140
H	30.80440	-21.15130	-8.68830
H	29.83580	-22.41560	-9.40820
C	29.23010	-21.87690	-7.40510
H	29.20790	-20.98180	-6.78350
H	28.19520	-22.20370	-7.50220
C	30.04290	-22.96810	-6.69460
H	31.08880	-22.66580	-6.63830
H	30.01510	-23.88370	-7.28520
C	29.51720	-23.25270	-5.28070
H	29.57830	-22.34340	-4.68270
H	28.45960	-23.50950	-5.33390
C	30.27770	-24.38070	-4.57030
H	31.34280	-24.14860	-4.55270
H	30.17010	-25.30400	-5.13910
C	29.77900	-24.60310	-3.13570
H	29.91440	-23.68790	-2.55920
H	28.70650	-24.79590	-3.15170
C	30.49240	-25.76210	-2.42670
H	31.56920	-25.59110	-2.44120
H	30.31740	-26.68580	-2.97770
C	30.02120	-25.93830	-0.97640
H	30.20770	-25.02210	-0.41580
H	28.94240	-26.09280	-0.95860
C	30.70940	-27.10930	-0.26820
H	31.79130	-26.97680	-0.24780
H	30.36470	-27.19650	0.76220
H	30.49400	-28.05300	-0.76790
C	32.60580	-18.45520	-14.92640
H	33.05790	-19.16280	-15.62270
H	31.53110	-18.63600	-14.95940
C	33.12670	-18.73240	-13.51040
H	34.20660	-18.58110	-13.48780
H	32.69930	-18.00730	-12.81740
C	32.79590	-20.15100	-13.02730
H	33.19800	-20.88080	-13.73110
H	31.71460	-20.28890	-13.02460
C	33.35280	-20.43330	-11.62590
H	34.43800	-20.32650	-11.64060

H	32.97830	-19.68310	-10.92950
C	32.98150	-21.82910	-11.10930
H	33.32610	-22.58560	-11.81520
H	31.89630	-21.91820	-11.06470
C	33.57610	-22.11580	-9.72450
H	34.66410	-22.06560	-9.78030
H	33.26580	-21.33550	-9.03010
C	33.15190	-23.48030	-9.16750
H	33.43210	-24.26700	-9.86880
H	32.06590	-23.50980	-9.08490
C	33.77510	-23.77300	-7.79680
H	34.86180	-23.78760	-7.88790
H	33.53260	-22.96210	-7.11080
C	33.29010	-25.09870	-7.19700
H	33.50400	-25.91520	-7.88750
H	32.20700	-25.06290	-7.08370
C	33.93290	-25.39820	-5.83700
H	35.01400	-25.47810	-5.95600
H	33.75640	-24.55970	-5.16420
C	33.38800	-26.68050	-5.19520
H	33.53880	-27.52240	-5.87180
H	32.31170	-26.58130	-5.05810
C	34.04480	-26.98700	-3.84330
H	35.11790	-27.12210	-3.98360
H	33.92480	-26.12850	-3.18340
C	33.45270	-28.23080	-3.16740
H	33.55060	-29.08920	-3.83310
H	32.38460	-28.08070	-3.01190
C	34.12130	-28.54800	-1.82310
H	35.18830	-28.71440	-1.97540
H	34.03460	-27.68830	-1.15810
C	33.51130	-29.77540	-1.13850
H	33.60150	-30.66340	-1.76480
H	34.01210	-29.98250	-0.19260
H	32.45380	-29.62090	-0.92630
O	30.12080	-13.83580	-20.92610
O	29.69030	-15.53130	-22.36510
O	31.47720	-17.83980	-21.64710
H	30.63590	-18.27750	-21.63960
C	29.83280	-14.15260	-22.24680
C	28.50990	-13.48270	-22.65150
H	28.58770	-12.40230	-22.52540
H	28.28880	-13.70120	-23.69650
H	27.68580	-13.84990	-22.04060
C	30.99730	-13.68790	-23.13020
H	30.80900	-13.96050	-24.16880
H	31.10430	-12.60460	-23.06370
H	31.93130	-14.14740	-22.81110

3 hexamer

C	31.01410	2.86260	-6.95530
C	28.61250	1.14010	-7.77050
O	30.76560	3.38300	-5.86870
O	28.65510	0.34570	-8.70820
N	32.01540	1.98450	-7.12140
N	28.52950	0.74370	-6.49310
H	32.14460	1.56180	-8.03190
H	28.50310	1.46000	-5.78080
C	30.12200	3.20130	-8.16510
H	30.05450	4.29040	-8.14780
C	28.67010	2.66310	-8.01440
H	28.22680	3.13230	-7.13410
C	30.73790	2.82330	-9.52780
H	31.76480	3.18630	-9.59660
H	30.77830	1.74190	-9.64240
C	27.79170	3.07420	-9.22280

H	26.86570	2.49570	-9.20770
C	29.90860	3.42780	-10.66390
H	29.96780	4.51220	-10.58750
C	28.43640	2.95330	-10.61520
H	28.34360	1.93380	-10.98940
C	28.60390	-0.63680	-6.03570
H	28.13810	-0.68480	-5.05100
H	28.02310	-1.29070	-6.68880
C	32.92630	1.57490	-6.06140
H	33.64470	2.37650	-5.88470
H	32.37340	1.43110	-5.13130
C	30.06460	-1.10960	-5.95110
H	30.50240	-1.14470	-6.95020
H	30.64500	-0.37990	-5.38530
C	30.19760	-2.48070	-5.27810
H	29.73650	-2.43410	-4.29060
H	29.63710	-3.22600	-5.84240
C	31.65660	-2.93500	-5.12950
H	32.23250	-2.15030	-4.63760
H	32.10400	-3.07650	-6.11340
C	31.76960	-4.22660	-4.31080
H	31.32890	-4.06000	-3.32700
H	31.17690	-5.01110	-4.78120
C	33.21460	-4.71290	-4.13780
H	33.82730	-3.90390	-3.73870
H	33.63540	-4.97350	-5.10890
C	33.29310	-5.91940	-3.19410
H	32.89900	-5.63210	-2.21840
H	32.64290	-6.71230	-3.56400
C	34.71540	-6.46780	-3.02240
H	35.38340	-5.66310	-2.71420
H	35.08550	-6.83290	-3.98030
C	34.76450	-7.59550	-1.98380
H	34.43280	-7.20730	-1.02000
H	34.05250	-8.37320	-2.26090
C	36.15840	-8.21680	-1.82790
H	36.88280	-7.43590	-1.59480
H	36.46830	-8.65920	-2.77450
C	36.18670	-9.28400	-0.72610
H	35.91930	-8.82370	0.22600
H	35.42070	-10.03260	-0.92950
C	37.54910	-9.97660	-0.59210
H	38.32300	-9.22900	-0.41600
H	37.79940	-10.47030	-1.53100
C	37.56300	-11.00400	0.54760
H	37.35250	-10.49880	1.49110
H	36.75520	-11.72030	0.39680
C	38.89520	-11.75730	0.65810
H	39.70750	-11.04320	0.79560
H	39.09460	-12.27930	-0.27770
C	38.89970	-12.76520	1.81530
H	38.73270	-12.24170	2.75730
H	38.06690	-13.45830	1.69820
C	40.20660	-13.55930	1.90360
H	41.05900	-12.89870	2.06190
H	40.17590	-14.26780	2.73150
H	40.38450	-14.12630	0.99070
C	33.65620	0.27660	-6.43040
H	34.22500	0.42100	-7.35000
H	32.92480	-0.50440	-6.63890
C	34.59630	-0.19260	-5.31260
H	35.35200	0.57260	-5.13050
H	34.02990	-0.29200	-4.38570
C	35.28340	-1.52800	-5.62840
H	35.85920	-1.43740	-6.55020
H	34.52560	-2.29000	-5.81040
C	36.20310	-1.98430	-4.48790
H	36.98340	-1.23840	-4.33110

H	35.63070	-2.03020	-3.56030
C	36.84700	-3.35180	-4.74980
H	37.42380	-3.31630	-5.67470
H	36.06400	-4.09450	-4.90160
C	37.75340	-3.79630	-3.59390
H	38.56000	-3.07320	-3.46590
H	37.18250	-3.78890	-2.66450
C	38.34690	-5.19440	-3.80990
H	38.92030	-5.21130	-4.73750
H	37.53630	-5.91180	-3.93460
C	39.24110	-5.63640	-2.64360
H	40.07570	-4.94160	-2.54130
H	38.67550	-5.57870	-1.71270
C	39.77790	-7.06280	-2.82110
H	40.34390	-7.12930	-3.75110
H	38.93870	-7.75030	-2.92140
C	40.66140	-7.50920	-1.64840
H	41.52310	-6.84540	-1.56880
H	40.10480	-7.40590	-0.71600
C	41.14100	-8.95970	-1.79400
H	41.69370	-9.07090	-2.72770
H	40.27540	-9.61710	-1.86870
C	42.02040	-9.41090	-0.62010
H	42.90310	-8.77280	-0.56160
H	41.47640	-9.27180	0.31500
C	42.45440	-10.87800	-0.74200
H	42.98910	-11.02430	-1.68140
H	41.56910	-11.51160	-0.79010
C	43.33930	-11.33030	0.42730
H	44.23560	-10.71040	0.46930
H	42.81120	-11.17320	1.36850
C	43.74770	-12.80300	0.31840
H	44.30170	-12.99320	-0.60130
H	44.38160	-13.09460	1.15590
H	42.87340	-13.45290	0.32300
O	27.43370	4.43910	-9.19080
O	27.64230	3.85130	-11.37310
O	30.50300	3.04270	-11.88430
H	30.13910	2.20790	-12.14860
C	27.04210	4.74210	-10.48840
C	25.51660	4.59310	-10.60680
H	25.02160	5.25810	-9.89870
H	25.19690	4.85170	-11.61650
H	25.21090	3.56790	-10.40000
C	27.50230	6.16770	-10.81880
H	27.23380	6.41690	-11.84570
H	27.02210	6.87810	-10.14550
H	28.58120	6.25990	-10.70660
C	32.53200	-0.95730	-10.35430
C	29.91080	-2.33730	-11.04270
O	32.37990	-0.22050	-9.38130
O	29.97770	-3.30110	-11.80340
N	33.31690	-2.04530	-10.31490
N	29.57980	-2.46160	-9.74970
H	33.37950	-2.62650	-11.14130
H	29.51010	-1.61530	-9.19790
C	31.76910	-0.64640	-11.65900
H	31.88840	0.43010	-11.79350
C	30.24060	-0.91580	-11.54280
H	29.84560	-0.22470	-10.80220
C	32.37840	-1.31350	-12.90940
H	33.45070	-1.11650	-12.95950
H	32.25990	-2.39390	-12.86130
C	29.49330	-0.60540	-12.86300
H	28.49290	-1.04100	-12.81890
C	31.70220	-0.78550	-14.17810
H	31.90830	0.28090	-14.25720
C	30.17860	-1.05360	-14.16250

H	29.96280	-2.10320	-14.35650
C	29.38590	-3.72960	-9.05730
H	28.87210	-3.52220	-8.11880
H	28.72750	-4.37790	-9.63810
C	34.07170	-2.47140	-9.14400
H	34.91730	-1.79690	-9.00290
H	33.44780	-2.39220	-8.25230
C	30.73090	-4.42710	-8.78480
H	31.21290	-4.68180	-9.72990
H	31.39850	-3.72900	-8.28030
C	30.59020	-5.69530	-7.93240
H	30.11310	-5.43810	-6.98630
H	29.92530	-6.40190	-8.42950
C	31.94250	-6.36960	-7.65550
H	32.62620	-5.63880	-7.22360
H	32.39070	-6.69180	-8.59580
C	31.82010	-7.56730	-6.70320
H	31.37760	-7.23350	-5.76430
H	31.12960	-8.29920	-7.12210
C	33.16800	-8.24370	-6.41380
H	33.87750	-7.49840	-6.05400
H	33.58260	-8.64530	-7.33880
C	33.04110	-9.36470	-5.37270
H	32.64580	-8.94920	-4.44550
H	32.30860	-10.09590	-5.71420
C	34.37070	-10.07440	-5.08160
H	35.12200	-9.33800	-4.79630
H	34.73420	-10.55450	-5.99030
C	34.23320	-11.11830	-3.96460
H	33.90220	-10.62530	-3.05040
H	33.44860	-11.82750	-4.22770
C	35.53470	-11.88210	-3.68510
H	36.33460	-11.17370	-3.46940
H	35.83450	-12.42820	-4.57960
C	35.38880	-12.85940	-2.51050
H	35.12740	-12.30430	-1.60950
H	34.55490	-13.53370	-2.70420
C	36.65700	-13.68340	-2.24950
H	37.50210	-13.01270	-2.09340
H	36.89060	-14.27860	-3.13220
C	36.50740	-14.60870	-1.03400
H	36.30580	-14.00980	-0.14570
H	35.63660	-15.24880	-1.17290
C	37.74560	-15.48160	-0.78950
H	38.62430	-14.84520	-0.68240
H	37.92260	-16.11110	-1.66130
C	37.59980	-16.36760	0.45550
H	37.43930	-15.74320	1.33450
H	36.71090	-16.99040	0.35800
C	38.81940	-17.26360	0.69210
H	39.72660	-16.67180	0.81390
H	38.69040	-17.86500	1.59200
H	38.97250	-17.94800	-0.14120
C	34.56190	-3.91690	-9.30540
H	35.18760	-3.99420	-10.19570
H	33.70720	-4.57300	-9.47200
C	35.35120	-4.40770	-8.08450
H	36.22410	-3.77020	-7.93900
H	34.73740	-4.29760	-7.19050
C	35.79840	-5.87080	-8.21290
H	36.39780	-5.99330	-9.11580
H	34.92140	-6.50640	-8.33600
C	36.60540	-6.34180	-6.99510
H	37.50030	-5.72670	-6.89420
H	36.01820	-6.18180	-6.09120
C	37.00620	-7.82080	-7.07840
H	37.57630	-7.99580	-7.99140
H	36.10710	-8.43210	-7.15260

C	37.83200	-8.27230	-5.86570
H	38.75060	-7.68680	-5.81390
H	37.27710	-8.05780	-4.95280
C	38.17640	-9.76710	-5.90360
H	38.71140	-9.99710	-6.82570
H	37.25310	-10.34510	-5.92880
C	39.02000	-10.20860	-4.69990
H	39.96350	-9.66160	-4.69680
H	38.50310	-9.93920	-3.77920
C	39.29890	-11.71740	-4.69550
H	39.79330	-12.00170	-5.62500
H	38.35120	-12.25430	-4.67220
C	40.15990	-12.15750	-3.50400
H	41.12620	-11.65370	-3.54780
H	39.68500	-11.83620	-2.57730
C	40.37120	-13.67660	-3.46010
H	40.82180	-14.01090	-4.39530
H	39.40200	-14.16930	-3.39040
C	41.25020	-14.11960	-2.28300
H	42.23370	-13.65600	-2.36990
H	40.81770	-13.75490	-1.35180
C	41.40390	-15.64450	-2.20530
H	41.81280	-16.01830	-3.14480
H	40.41960	-16.09870	-2.09460
C	42.30220	-16.09180	-1.04430
H	43.29460	-15.65460	-1.15980
H	41.90440	-15.71080	-0.10370
C	42.42810	-17.61580	-0.95240
H	42.84540	-18.03470	-1.86850
H	43.07970	-17.90350	-0.12720
H	41.45640	-18.07920	-0.78390
O	29.33740	0.77750	-13.09130
O	29.54960	-0.21510	-15.11820
O	32.29520	-1.43210	-15.28320
H	31.78160	-2.20100	-15.49400
C	29.04220	0.89100	-14.44410
C	27.51660	0.92380	-14.62890
H	27.09370	1.76220	-14.07430
H	27.27420	1.04120	-15.68540
H	27.06220	0.00030	-14.27140
C	29.70650	2.16090	-14.99040
H	29.52200	2.24640	-16.06140
H	29.29610	3.03920	-14.49110
H	30.78180	2.13730	-14.82070
C	33.64240	-10.37220	-15.39350
C	30.68920	-10.84170	-15.93530
O	33.78220	-9.39640	-14.65750
O	30.38880	-11.93860	-16.40320
N	33.96980	-11.61450	-15.00740
N	30.34740	-10.46930	-14.69370
H	33.80630	-12.37840	-15.65100
H	30.59970	-9.53410	-14.39640
C	33.03600	-10.17840	-16.79930
H	33.54750	-9.30070	-17.19770
C	31.51840	-9.83480	-16.75900
H	31.41790	-8.87150	-16.26440
C	33.33700	-11.33990	-17.77010
H	34.40470	-11.56580	-17.77140
H	32.82510	-12.24520	-17.45090
C	30.92540	-9.65610	-18.17860
H	29.83540	-9.66690	-18.11530
C	32.89000	-10.98090	-19.19010
H	33.47880	-10.12970	-19.52820
C	31.37700	-10.66360	-19.24580
H	30.78140	-11.57210	-19.17480
C	29.71180	-11.33340	-13.70720
H	29.31580	-10.70050	-12.91310
H	28.85960	-11.84890	-14.15330

C	34.52180	-11.95200	-13.70320
H	35.55880	-11.61630	-13.65930
H	33.97970	-11.41940	-12.92000
C	30.71650	-12.34510	-13.12850
H	31.08150	-12.99640	-13.92420
H	31.58590	-11.80790	-12.74910
C	30.12760	-13.20720	-12.00430
H	29.77880	-12.55790	-11.20090
H	29.25140	-13.74290	-12.37030
C	31.14830	-14.21130	-11.44880
H	32.04880	-13.67680	-11.14600
H	31.44960	-14.89950	-12.23930
C	30.61100	-15.00660	-10.25150
H	30.31970	-14.31250	-9.46280
H	29.70470	-15.53870	-10.54060
C	31.63660	-16.00620	-9.69730
H	32.56300	-15.48110	-9.46400
H	31.88160	-16.74110	-10.46470
C	31.13290	-16.72360	-8.43760
H	30.90540	-15.98330	-7.67040
H	30.19390	-17.23070	-8.65840
C	32.14170	-17.73820	-7.88090
H	33.09900	-17.24420	-7.71260
H	32.31990	-18.51830	-8.62130
C	31.66050	-18.37230	-6.56870
H	31.50830	-17.58970	-5.82540
H	30.68580	-18.83310	-6.72620
C	32.63380	-19.41990	-6.01080
H	33.62380	-18.97660	-5.90060
H	32.73590	-20.23720	-6.72460
C	32.16980	-19.97440	-4.65680
H	32.09880	-19.15860	-3.93750
H	31.16220	-20.37630	-4.75810
C	33.09650	-21.06370	-4.09990
H	34.11600	-20.68150	-4.04060
H	33.11960	-21.90700	-4.78980
C	32.65050	-21.54850	-2.71340
H	32.65540	-20.70940	-2.01750
H	31.61790	-21.89260	-2.76610
C	33.53290	-22.67470	-2.15890
H	34.57330	-22.34840	-2.14130
H	33.48770	-23.53280	-2.82900
C	33.10760	-23.10740	-0.74890
H	33.16410	-22.25540	-0.07090
H	32.06330	-23.41880	-0.76140
C	33.96670	-24.24780	-0.19470
H	35.01770	-23.96230	-0.14720
H	33.64910	-24.51920	0.81220
H	33.88620	-25.13870	-0.81630
C	34.43770	-13.46390	-13.45760
H	34.97850	-13.99250	-14.24380
H	33.39870	-13.78640	-13.52970
C	35.00420	-13.86850	-12.09120
H	36.05230	-13.57230	-12.03410
H	34.48550	-13.31950	-11.30480
C	34.88010	-15.37480	-11.82510
H	35.37290	-15.92980	-12.62440
H	33.82890	-15.66190	-11.85360
C	35.48680	-15.77800	-10.47540
H	36.54590	-15.51800	-10.46450
H	35.01850	-15.19840	-9.67980
C	35.32270	-17.27260	-10.17260
H	35.76060	-17.86150	-10.97930
H	34.26150	-17.51980	-10.14890
C	35.97180	-17.67000	-8.84090
H	37.04080	-17.45870	-8.88260
H	35.56480	-17.05030	-8.04240
C	35.75280	-19.14650	-8.48950

H	36.13100	-19.77700	-9.29490
H	34.68310	-19.34100	-8.41790
C	36.43180	-19.53930	-7.17150
H	37.50810	-19.38690	-7.25850
H	36.08780	-18.87580	-6.37870
C	36.14730	-20.99080	-6.76690
H	36.46510	-21.66460	-7.56320
H	35.07170	-21.12450	-6.65650
C	36.84400	-21.38100	-5.45740
H	37.92390	-21.29020	-5.57910
H	36.55980	-20.67650	-4.67670
C	36.49350	-22.80280	-5.00100
H	36.75500	-23.51530	-5.78410
H	35.41560	-22.87650	-4.86120
C	37.20110	-23.19210	-3.69690
H	38.28110	-23.15310	-3.84400
H	36.96680	-22.45620	-2.92850
C	36.79850	-24.58670	-3.19980
H	37.01210	-25.32580	-3.97300
H	35.72130	-24.61260	-3.03700
C	37.51690	-24.98130	-1.90250
H	38.59540	-24.97290	-2.06430
H	37.31490	-24.23820	-1.13050
C	37.09300	-26.36280	-1.39340
H	37.30390	-27.13790	-2.13050
H	37.62520	-26.62020	-0.47750
H	36.02590	-26.38980	-1.17490
O	31.29920	-8.43540	-18.77650
O	31.09560	-9.95560	-20.44280
O	33.18260	-12.07940	-20.02620
H	32.41000	-12.62600	-20.08650
C	31.05040	-8.60050	-20.13210
C	29.64690	-8.06540	-20.45840
H	29.57830	-7.01240	-20.18260
H	29.45240	-8.16400	-21.52660
H	28.88330	-8.62180	-19.91580
C	32.13690	-7.86330	-20.92480
H	31.98230	-8.00980	-21.99400
H	32.09640	-6.79580	-20.70530
H	33.12620	-8.23290	-20.65980
C	33.49660	-5.43500	-13.18800
C	30.67260	-6.38540	-13.78590
O	33.47130	-4.55570	-12.32810
O	30.57130	-7.44640	-14.39920
N	34.06080	-6.63310	-12.97240
N	30.30190	-6.25510	-12.50430
H	34.02590	-7.32320	-13.71210
H	30.38230	-5.33760	-12.08200
C	32.82640	-5.17470	-14.55360
H	33.15020	-4.16950	-14.82840
C	31.27280	-5.13750	-14.46580
H	31.00430	-4.28580	-13.84520
C	33.31690	-6.11510	-15.67450
H	34.40770	-6.12930	-15.70700
H	32.99550	-7.13660	-15.48240
C	30.61790	-4.88810	-15.84700
H	29.55240	-5.11850	-15.78500
C	32.77170	-5.66020	-17.03130
H	33.17730	-4.67340	-17.24890
C	31.22500	-5.63690	-17.04230
H	30.81670	-6.64470	-17.09640
C	29.86520	-7.34840	-11.64520
H	29.38300	-6.91380	-10.76970
H	29.10930	-7.94780	-12.15550
C	34.70100	-7.02810	-11.72540
H	35.65670	-6.50960	-11.63770
H	34.08880	-6.71550	-10.87790
C	31.05450	-8.22540	-11.21570

H	31.50600	-8.68890	-12.09420
H	31.82400	-7.59200	-10.77420
C	30.66710	-9.32000	-10.21270
H	30.23020	-8.85580	-9.32820
H	29.89240	-9.95600	-10.64150
C	31.86940	-10.18210	-9.80010
H	32.66640	-9.53450	-9.43480
H	32.26730	-10.69610	-10.67580
C	31.52030	-11.20900	-8.71440
H	31.13200	-10.68660	-7.83970
H	30.71650	-11.85610	-9.06560
C	32.72450	-12.06740	-8.30100
H	33.54760	-11.41660	-8.00570
H	33.07560	-12.64170	-9.15880
C	32.39340	-13.01860	-7.14300
H	32.06020	-12.43550	-6.28420
H	31.55350	-13.65420	-7.42300
C	33.58160	-13.89780	-6.72850
H	34.44030	-13.26550	-6.50190
H	33.87440	-14.53260	-7.56510
C	33.25720	-14.76960	-5.50770
H	32.99240	-14.12760	-4.66740
H	32.37410	-15.37210	-5.71830
C	34.41440	-15.69050	-5.09700
H	35.31390	-15.09630	-4.93430
H	34.63810	-16.37820	-5.91250
C	34.09290	-16.48780	-3.82540
H	33.90170	-15.79650	-3.00450
H	33.16800	-17.04450	-3.97330
C	35.21050	-17.45900	-3.42180
H	36.14830	-16.91310	-3.31570
H	35.36230	-18.18750	-4.21800
C	34.89410	-18.19220	-2.11090
H	34.76940	-17.46380	-1.30950
H	33.93740	-18.70500	-2.20590
C	35.97590	-19.20550	-1.71390
H	36.94200	-18.70330	-1.65510
H	36.06590	-19.96230	-2.49270
C	35.67120	-19.88890	-0.37360
H	35.59130	-19.13750	0.41230
H	34.69940	-20.37940	-0.42610
C	36.73430	-20.91790	0.02240
H	37.71850	-20.45640	0.10460
H	36.49690	-21.37060	0.98510
H	36.79770	-21.72020	-0.71150
C	34.91040	-8.54740	-11.68210
H	35.52220	-8.85630	-12.53090
H	33.94960	-9.04990	-11.79720
C	35.57630	-9.01130	-10.38050
H	36.55060	-8.53100	-10.28330
H	34.98220	-8.67800	-9.52920
C	35.74520	-10.53540	-10.31500
H	36.31580	-10.87810	-11.17910
H	34.76660	-11.01070	-10.38380
C	36.44680	-10.98860	-9.02810
H	37.43830	-10.53690	-8.97950
H	35.89550	-10.61620	-8.16480
C	36.57230	-12.51400	-8.92510
H	37.09690	-12.89940	-9.80010
H	35.57710	-12.95780	-8.94000
C	37.31020	-12.95320	-7.65380
H	38.31990	-12.54130	-7.66070
H	36.81000	-12.53120	-6.78260
C	37.37760	-14.47800	-7.50320
H	37.85090	-14.91420	-8.38350
H	36.36460	-14.87760	-7.46480
C	38.14330	-14.90880	-6.24580
H	39.17060	-14.54770	-6.30480

H	37.69740	-14.43340	-5.37270
C	38.14150	-16.42920	-6.04360
H	38.56180	-16.91810	-6.92310
H	37.11200	-16.77510	-5.95770
C	38.92600	-16.85710	-4.79710
H	39.96780	-16.55180	-4.90060
H	38.53370	-16.33020	-3.92800
C	38.85330	-18.36810	-4.54290
H	39.22320	-18.90610	-5.41640
H	37.81060	-18.65860	-4.41820
C	39.64950	-18.79550	-3.30340
H	40.70070	-18.53950	-3.44120
H	39.30310	-18.22700	-2.44120
C	39.51870	-20.29510	-3.00540
H	39.84470	-20.86950	-3.87330
H	38.46820	-20.53970	-2.85000
C	40.32690	-20.72600	-1.77420
H	41.38190	-20.49820	-1.93090
H	40.01140	-20.14610	-0.90650
C	40.17290	-22.21830	-1.46320
H	40.50290	-22.83320	-2.30080
H	40.76490	-22.49720	-0.59140
H	39.13400	-22.46950	-1.25180
O	30.73310	-3.54890	-16.27220
O	30.78050	-4.84280	-18.13080
O	33.24700	-6.55780	-18.01090
H	32.59280	-7.23120	-18.14520
C	30.48420	-3.57630	-17.63780
C	28.99580	-3.28500	-17.88740
H	28.73330	-2.31220	-17.46990
H	28.79530	-3.27510	-18.95900
H	28.36830	-4.04680	-17.42600
C	31.38710	-2.54350	-18.32370
H	31.23540	-2.57420	-19.40280
H	31.14760	-1.54290	-17.96210
H	32.43560	-2.74560	-18.11120
C	24.89790	-29.33350	-17.65790
C	22.32960	-27.98610	-18.57400
O	25.75440	-28.47400	-17.45770
O	21.33770	-28.70890	-18.48680
N	24.39890	-30.09110	-16.67180
N	22.50440	-26.89510	-17.81370
H	23.68690	-30.76620	-16.91070
H	23.33440	-26.33600	-17.97330
C	24.33600	-29.53660	-19.08120
H	25.21680	-29.56350	-19.72460
C	23.46770	-28.33430	-19.55380
H	24.11710	-27.46280	-19.60440
C	23.60620	-30.88670	-19.27260
H	24.24610	-31.70830	-18.94620
H	22.70720	-30.93360	-18.65660
C	22.91450	-28.54780	-20.98150
H	22.13980	-27.80480	-21.18170
C	23.23110	-31.09810	-20.74450
H	24.14900	-31.20170	-21.32130
C	22.36620	-29.94620	-21.30430
H	21.33110	-30.04070	-20.97270
C	21.64400	-26.50760	-16.70360
H	21.84560	-25.46380	-16.46260
H	20.59640	-26.56600	-17.00410
C	24.77360	-29.98410	-15.27010
H	25.78430	-30.37340	-15.14010
H	24.79080	-28.93560	-14.96820
C	21.90910	-27.39720	-15.47790
H	21.72480	-28.43950	-15.74120
H	22.96350	-27.33290	-15.20940
C	21.04950	-27.03710	-14.25950
H	21.26130	-26.01260	-13.95270

H	19.99420	-27.06780	-14.53360
C	21.30210	-27.99850	-13.08950
H	22.36600	-28.00270	-12.85300
H	21.05390	-29.01390	-13.40060
C	20.50420	-27.64990	-11.82580
H	20.76620	-26.64420	-11.49620
H	19.43840	-27.63220	-12.05690
C	20.76700	-28.65200	-10.69240
H	21.83950	-28.70970	-10.50680
H	20.45730	-29.64740	-11.01270
C	20.04740	-28.29280	-9.38500
H	20.36960	-27.30530	-9.05340
H	18.97370	-28.22360	-9.56400
C	20.31750	-29.32140	-8.27720
H	21.39370	-29.42860	-8.14090
H	19.94870	-30.29770	-8.59310
C	19.67310	-28.94370	-6.93620
H	20.05440	-27.97510	-6.61130
H	18.59720	-28.82310	-7.06830
C	19.94050	-29.99160	-5.84620
H	21.01550	-30.14490	-5.74970
H	19.51880	-30.94920	-6.15330
C	19.35990	-29.59350	-4.48220
H	19.79180	-28.64230	-4.16910
H	18.28600	-29.42810	-4.57680
C	19.62020	-30.65300	-3.40220
H	20.69120	-30.84630	-3.33750
H	19.15390	-31.59380	-3.69610
C	19.09450	-30.23240	-2.02270
H	19.56320	-29.29250	-1.72900
H	18.02340	-30.03630	-2.08480
C	19.35810	-31.29190	-0.94360
H	20.42480	-31.51320	-0.90880
H	18.85940	-32.22230	-1.21680
C	18.88240	-30.84710	0.44660
H	19.36240	-29.90560	0.71550
H	17.81020	-30.64920	0.42070
C	19.17880	-31.88760	1.53140
H	20.24840	-32.08300	1.60670
H	18.83560	-31.54140	2.50650
H	18.67760	-32.83210	1.31990
C	23.78030	-30.76340	-14.39970
H	23.75890	-31.80650	-14.71850
H	22.77460	-30.37270	-14.55770
C	24.12300	-30.69800	-12.90640
H	25.12400	-31.10120	-12.75000
H	24.15380	-29.65740	-12.58220
C	23.11740	-31.47240	-12.04380
H	23.06260	-32.50530	-12.39000
H	22.12140	-31.04980	-12.17840
C	23.48660	-31.45740	-10.55520
H	24.47950	-31.88990	-10.42880
H	23.55060	-30.42740	-10.20430
C	22.48160	-32.22830	-9.68910
H	22.39160	-33.25050	-10.05880
H	21.49450	-31.77640	-9.78900
C	22.89060	-32.25800	-8.21060
H	23.87090	-32.72680	-8.11990
H	23.00100	-31.23810	-7.84250
C	21.88440	-33.00850	-7.32870
H	21.75130	-34.02210	-7.70870
H	20.91050	-32.52310	-7.39680
C	22.33120	-33.06970	-5.86160
H	23.29300	-33.57980	-5.80020
H	22.49420	-32.05890	-5.48790
C	21.31710	-33.78450	-4.95960
H	21.13480	-34.78990	-5.34110
H	20.36210	-33.26030	-5.00370

C	21.79240	-33.87110	-3.50250
H	22.73110	-34.42450	-3.46200
H	22.00880	-32.87010	-3.12910
C	20.76400	-34.54250	-2.58320
H	20.53210	-35.53860	-2.96210
H	19.83190	-33.97750	-2.61200
C	21.25650	-34.65100	-1.13330
H	22.16990	-35.24610	-1.10540
H	21.52320	-33.66070	-0.76350
C	20.21010	-35.27560	-0.20070
H	19.93410	-36.26320	-0.57260
H	19.30130	-34.67310	-0.22360
C	20.70940	-35.39690	1.24590
H	21.60010	-36.02550	1.27400
H	21.01370	-34.41670	1.61340
C	19.64970	-35.97940	2.18700
H	19.34810	-36.97850	1.87110
H	20.03330	-36.05410	3.20480
H	18.75830	-35.35200	2.21350
O	23.90830	-28.39620	-21.96950
O	22.45920	-29.96770	-22.72130
O	22.50310	-32.30360	-20.84010
H	22.22790	-32.40630	-21.73950
C	23.39200	-29.00690	-23.10290
C	22.68870	-27.95150	-23.97150
H	23.39460	-27.16640	-24.24530
H	22.30640	-28.41520	-24.88110
H	21.85320	-27.50190	-23.43590
C	24.54100	-29.68850	-23.85620
H	24.15560	-30.20950	-24.73290
H	25.26600	-28.94110	-24.18050
H	25.05040	-30.40640	-23.21530
C	28.38610	-25.24920	-18.02520
C	25.60160	-24.28140	-18.76620
O	29.08990	-24.29840	-17.68720
O	24.73460	-25.15100	-18.83080
N	28.02970	-26.21650	-17.16630
N	25.60720	-23.33600	-17.81560
H	27.42640	-26.95870	-17.49760
H	26.33580	-22.63290	-17.85480
C	27.86090	-25.32340	-19.47510
H	28.72550	-25.07000	-20.09060
C	26.76710	-24.25750	-19.77610
H	27.23490	-23.27950	-19.68680
C	27.41800	-26.73830	-19.90410
H	28.19660	-27.46670	-19.67100
H	26.53020	-27.04040	-19.35380
C	26.24400	-24.35690	-21.23070
H	25.32360	-23.77640	-21.32140
C	27.11470	-26.77620	-21.40490
H	28.03580	-26.56180	-21.94470
C	26.00370	-25.76960	-21.78520
H	25.02360	-26.13360	-21.47880
C	24.69980	-23.29160	-16.67590
H	24.74240	-22.28860	-16.25090
H	23.67190	-23.45010	-17.00660
C	28.42880	-26.25900	-15.76610
H	29.48900	-26.50920	-15.70680
H	28.30780	-25.27260	-15.31520
C	25.09690	-24.33380	-15.61630
H	25.02900	-25.33500	-16.04460
H	26.14260	-24.18620	-15.34560
C	24.23480	-24.27130	-14.34870
H	24.30740	-23.27480	-13.91230
H	23.18650	-24.41920	-14.60920
C	24.66030	-25.32060	-13.31110
H	25.72240	-25.20210	-13.09670
H	24.54300	-26.31820	-13.73470

C	23.86900	-25.22690	-11.99960
H	24.00260	-24.23500	-11.56740
H	22.80360	-25.33020	-12.20470
C	24.29980	-26.29230	-10.98140
H	25.37430	-26.21740	-10.81500
H	24.12180	-27.28480	-11.39550
C	23.57000	-26.16080	-9.63780
H	23.77080	-25.17750	-9.21220
H	22.49330	-26.20870	-9.79960
C	23.98420	-27.24700	-8.63560
H	25.06740	-27.23320	-8.51480
H	23.73150	-28.22810	-9.03780
C	23.31920	-27.06930	-7.26420
H	23.59550	-26.09900	-6.85110
H	22.23590	-27.05250	-7.38270
C	23.70700	-28.17540	-6.27380
H	24.79300	-28.22640	-6.19450
H	23.38000	-29.14030	-6.66130
C	23.10610	-27.95300	-4.87960
H	23.45670	-27.00020	-4.48220
H	22.02220	-27.87010	-4.95880
C	23.46150	-29.07740	-3.89810
H	24.54480	-29.19120	-3.85300
H	23.06660	-30.02240	-4.27140
C	22.91850	-28.81700	-2.48690
H	23.33470	-27.88460	-2.10460
H	21.83900	-28.67320	-2.53320
C	23.24010	-29.95740	-1.51240
H	24.31730	-30.12370	-1.49050
H	22.79220	-30.88190	-1.87720
C	22.73920	-29.67250	-0.09020
H	23.20090	-28.75970	0.28740
H	21.66580	-29.48500	-0.11100
C	23.03320	-30.82300	0.87710
H	24.10540	-30.99860	0.96290
H	22.65120	-30.60090	1.87360
H	22.56570	-31.74850	0.54150
C	27.59250	-27.28880	-14.99550
H	27.71550	-28.27240	-15.45090
H	26.53540	-27.03840	-15.08650
C	27.97320	-27.36490	-13.51120
H	29.02500	-27.63940	-13.42340
H	27.87320	-26.37820	-13.05810
C	27.11330	-28.37050	-12.73310
H	27.19030	-29.35340	-13.19940
H	26.06550	-28.07830	-12.80060
C	27.52370	-28.47450	-11.25820
H	28.56640	-28.78770	-11.19690
H	27.46920	-27.48920	-10.79540
C	26.64830	-29.45350	-10.46490
H	26.67560	-30.43490	-10.93930
H	25.61150	-29.11980	-10.50070
C	27.09520	-29.58550	-9.00310
H	28.12330	-29.94760	-8.97260
H	27.09980	-28.60140	-8.53550
C	26.19740	-30.52490	-8.18790
H	26.16370	-31.50460	-8.66540
H	25.17750	-30.14080	-8.19310
C	26.67860	-30.68310	-6.73990
H	27.68560	-31.10170	-6.73830
H	26.75110	-29.70120	-6.27360
C	25.75300	-31.57130	-5.89920
H	25.65130	-32.54750	-6.37440
H	24.75620	-31.13100	-5.87710
C	26.26350	-31.75550	-4.46420
H	27.24470	-32.23090	-4.48760
H	26.40460	-30.77880	-4.00260
C	25.31020	-32.58860	-3.59840

H	25.14380	-33.55890	-4.06740
H	24.33960	-32.09430	-3.55590
C	25.84150	-32.79600	-2.17400
H	26.79650	-33.32110	-2.21640
H	26.04300	-31.82680	-1.71870
C	24.86410	-33.57830	-1.28730
H	24.64550	-34.54230	-1.74830
H	23.91760	-33.04020	-1.23330
C	25.40590	-33.80270	0.13080
H	26.34110	-34.36150	0.08080
H	25.64570	-32.84270	0.58870
C	24.41570	-34.55400	1.02670
H	24.17640	-35.53480	0.61490
H	24.82960	-34.70390	2.02400
H	23.48390	-33.99920	1.13470
O	27.15940	-23.84190	-22.17100
O	26.04980	-25.53670	-23.18430
O	26.70580	-28.08710	-21.73260
H	25.76160	-28.13530	-21.66060
C	26.75560	-24.35650	-23.39510
C	25.82980	-23.34460	-24.08920
H	26.35250	-22.39720	-24.22630
H	25.52950	-23.72660	-25.06520
H	24.93280	-23.16900	-23.49630
C	28.00190	-24.65570	-24.23740
H	27.71040	-25.09480	-25.19160
H	28.55190	-23.73320	-24.42660
H	28.66100	-25.34990	-23.71850
C	31.06780	-20.55190	-17.79260
C	28.13570	-20.03610	-18.40180
O	31.58200	-19.53820	-17.32190
O	27.44070	-21.03300	-18.59130
N	30.92360	-21.67940	-17.08000
N	27.99060	-19.24890	-17.32630
H	30.46920	-22.47150	-17.51650
H	28.57780	-18.42560	-17.26280
C	30.53350	-20.52620	-19.24040
H	31.32120	-20.03310	-19.81210
C	29.25290	-19.65510	-19.39490
H	29.53130	-18.62800	-19.17010
C	30.35360	-21.92790	-19.86070
H	31.26110	-22.51980	-19.72950
H	29.55260	-22.46610	-19.35880
C	28.72500	-19.65130	-20.85090
H	27.71060	-19.24730	-20.86310
C	30.02840	-21.81630	-21.35310
H	30.88010	-21.35930	-21.85460
C	28.73960	-20.99550	-21.59360
H	27.85250	-21.57280	-21.33790
C	27.11350	-19.53060	-16.19710
H	26.99330	-18.60910	-15.62740
H	26.12120	-19.81220	-16.55330
C	31.35290	-21.83810	-15.69780
H	32.44160	-21.90100	-15.66970
H	31.06840	-20.95950	-15.11630
C	27.70310	-20.63470	-15.30210
H	27.79260	-21.56050	-15.87250
H	28.71570	-20.35490	-15.01070
C	26.87160	-20.90070	-14.04050
H	26.79240	-19.97960	-13.46270
H	25.85500	-21.17800	-14.32060
C	27.48350	-22.00600	-13.16720
H	28.52110	-21.75500	-12.94630
H	27.50560	-22.94150	-13.72700
C	26.72660	-22.21450	-11.84860
H	26.71360	-21.27810	-11.29040
H	25.68550	-22.46030	-12.05790
C	27.34920	-23.31760	-10.98040

H	28.40470	-23.09730	-10.81930
H	27.31010	-24.26730	-11.51430
C	26.65100	-23.46230	-9.62140
H	26.71000	-22.51580	-9.08380
H	25.59020	-23.65830	-9.77570
C	27.25550	-24.57770	-8.75680
H	28.32690	-24.41030	-8.64540
H	27.14310	-25.53460	-9.26660
C	26.60700	-24.65590	-7.36790
H	26.74530	-23.70630	-6.85080
H	25.53080	-24.78740	-7.47680
C	27.17360	-25.79240	-6.50590
H	28.25710	-25.69250	-6.43740
H	26.98050	-26.74800	-6.99300
C	26.57300	-25.80600	-5.09350
H	26.79460	-24.86120	-4.59700
H	25.48700	-25.86500	-5.16230
C	27.09120	-26.96710	-4.23440
H	28.18070	-26.93950	-4.20160
H	26.81870	-27.91280	-4.70240
C	26.53630	-26.92560	-2.80410
H	26.83640	-25.99300	-2.32570
H	25.44700	-26.91310	-2.83860
C	27.00390	-28.11100	-1.94950
H	28.09360	-28.14770	-1.94140
H	26.66580	-29.04090	-2.40630
C	26.48390	-28.03320	-0.50770
H	26.83920	-27.11540	-0.03810
H	25.39590	-27.97150	-0.51420
C	26.91540	-29.23280	0.34130
H	28.00160	-29.30410	0.39820
H	26.53330	-29.14640	1.35860
H	26.53710	-30.16590	-0.07500
C	30.72820	-23.09510	-15.07890
H	31.01280	-23.97060	-15.66420
H	29.64160	-23.02720	-15.13700
C	31.15000	-23.30080	-13.61870
H	32.23520	-23.39780	-13.56820
H	30.89250	-22.41520	-13.03710
C	30.49350	-24.53370	-12.98320
H	30.72460	-25.41860	-13.57750
H	29.40980	-24.41980	-13.00850
C	30.95350	-24.75660	-11.53680
H	32.03480	-24.89800	-11.52100
H	30.75060	-23.86100	-10.94960
C	30.27000	-25.95870	-10.87320
H	30.44200	-26.85460	-11.47080
H	29.19250	-25.79660	-10.85970
C	30.77130	-26.19640	-9.44300
H	31.84380	-26.39280	-9.46440
H	30.63460	-25.28790	-8.85720
C	30.05110	-27.35570	-8.74390
H	30.15760	-28.26520	-9.33600
H	28.98460	-27.13720	-8.69730
C	30.58380	-27.60440	-7.32710
H	31.64210	-27.86250	-7.37820
H	30.51730	-26.68240	-6.75070
C	29.81920	-28.71090	-6.59020
H	29.85640	-29.63300	-7.17110
H	28.76870	-28.43060	-6.51770
C	30.37490	-28.97030	-5.18430
H	31.41490	-29.29030	-5.25840
H	30.37750	-28.03770	-4.62120
C	29.56660	-30.02040	-4.41200
H	29.53760	-30.95120	-4.97940
H	28.53530	-29.68070	-4.31960
C	30.13950	-30.29100	-3.01490
H	31.16070	-30.66280	-3.10720

H	30.20100	-29.35330	-2.46360
C	29.29720	-31.29340	-2.21510
H	29.21440	-32.22720	-2.77250
H	28.28390	-30.90670	-2.10790
C	29.88210	-31.57820	-0.82530
H	30.89030	-31.98110	-0.92820
H	29.97840	-30.64510	-0.26930
C	29.02610	-32.55920	-0.01760
H	28.92980	-33.51590	-0.53160
H	29.46960	-32.74930	0.95990
H	28.02380	-32.16390	0.14490
O	29.50550	-18.85220	-21.71080
O	28.70530	-20.57250	-22.94760
O	29.86760	-23.12340	-21.86020
H	28.94680	-23.34790	-21.82840
C	29.17370	-19.26350	-22.99430
C	28.05880	-18.35910	-23.54350
H	28.39260	-17.32090	-23.54900
H	27.81050	-18.65550	-24.56290
H	27.15980	-18.43660	-22.93290
C	30.43220	-19.20730	-23.86910
H	30.20310	-19.56040	-24.87480
H	30.79630	-18.18110	-23.92970
H	31.22170	-19.82940	-23.45050
C	32.83720	-15.50650	-16.93060
C	29.84630	-15.47470	-17.47270
O	33.16320	-14.48550	-16.32670
O	29.34100	-16.54750	-17.79870
N	32.92320	-16.72530	-16.37680
N	29.58520	-14.88160	-16.29910
H	32.61480	-17.52370	-16.91700
H	30.01230	-13.98000	-16.12200
C	32.27410	-15.39100	-18.36290
H	32.94300	-14.68820	-18.86200
C	30.85070	-14.76250	-18.40220
H	30.93830	-13.74070	-18.04020
C	32.34320	-16.70760	-19.16520
H	33.34750	-17.13150	-19.11230
H	31.66770	-17.44750	-18.74140
C	30.29950	-14.66400	-19.84620
H	29.22800	-14.45750	-19.80750
C	31.96960	-16.46270	-20.63000
H	32.71070	-15.79320	-21.06390
C	30.54610	-15.87170	-20.76200
H	29.78670	-16.63220	-20.58770
C	28.80100	-15.47090	-15.22130
H	28.53040	-14.67390	-14.52890
H	27.86720	-15.87650	-15.61470
C	33.40590	-16.98280	-15.02760
H	34.48780	-16.84450	-15.00540
H	32.97680	-16.25780	-14.33380
C	29.60100	-16.56200	-14.48830
H	29.84040	-17.36990	-15.18140
H	30.55410	-16.14650	-14.16070
C	28.86210	-17.14310	-13.27580
H	28.63680	-16.33890	-12.57500
H	27.90320	-17.55550	-13.59060
C	29.68070	-18.23130	-12.56540
H	30.66350	-17.83300	-12.31270
H	29.85190	-19.06300	-13.24970
C	29.00540	-18.74740	-11.28770
H	28.84380	-17.91200	-10.60600
H	28.01800	-19.14170	-11.52750
C	29.83120	-19.83110	-10.57920
H	30.83740	-19.45470	-10.39370
H	29.93980	-20.69480	-11.23590
C	29.20420	-20.27030	-9.24910
H	29.11340	-19.40500	-8.59230

H	28.18920	-20.62570	-9.42500
C	30.01220	-21.36540	-8.53830
H	31.04250	-21.03250	-8.41150
H	30.04840	-22.25620	-9.16580
C	29.42250	-21.72420	-7.16750
H	29.41220	-20.83590	-6.53600
H	28.38120	-22.02110	-7.28800
C	30.19160	-22.84520	-6.45460
H	31.24480	-22.57400	-6.37800
H	30.14720	-23.75410	-7.05450
C	29.63440	-23.12710	-5.05230
H	29.70930	-22.22470	-4.44550
H	28.57130	-23.35460	-5.12550
C	30.35200	-24.28090	-4.33880
H	31.42320	-24.08030	-4.30480
H	30.22520	-25.19730	-4.91490
C	29.82650	-24.49610	-2.91270
H	29.98200	-23.58880	-2.32860
H	28.74900	-24.65550	-2.94470
C	30.49360	-25.68070	-2.20120
H	31.57550	-25.54370	-2.20010
H	30.29690	-26.59530	-2.75990
C	29.99730	-25.84920	-0.75850
H	30.20800	-24.94300	-0.18980
H	28.91380	-25.96660	-0.75570
C	30.63540	-27.04740	-0.04920
H	31.72050	-26.95050	-0.01120
H	30.27210	-27.13100	0.97510
H	30.39710	-27.97960	-0.55990
C	33.03880	-18.40510	-14.58600
H	33.46800	-19.12630	-15.28300
H	31.95750	-18.53410	-14.63800
C	33.52320	-18.72120	-13.16570
H	34.60850	-18.62120	-13.12520
H	33.11960	-17.98400	-12.47090
C	33.11870	-20.12790	-12.70490
H	33.49490	-20.86760	-13.41280
H	32.03210	-20.21420	-12.71740
C	33.64410	-20.45230	-11.30090
H	34.73320	-20.39510	-11.30210
H	33.29590	-19.69420	-10.59940
C	33.20410	-21.83570	-10.80580
H	33.52140	-22.59890	-11.51720
H	32.11560	-21.87560	-10.77260
C	33.77190	-22.16350	-9.41910
H	34.86150	-22.16040	-9.46480
H	33.48920	-21.37790	-8.71900
C	33.28400	-23.51330	-8.87940
H	33.53680	-24.30530	-9.58510
H	32.19710	-23.49610	-8.80640
C	33.88240	-23.84260	-7.50620
H	34.96820	-23.90220	-7.58840
H	33.66810	-23.02720	-6.81620
C	33.33750	-25.14990	-6.91870
H	33.52370	-25.97120	-7.61150
H	32.25600	-25.06930	-6.81450
C	33.95570	-25.48080	-5.55460
H	35.03370	-25.60420	-5.66420
H	33.80730	-24.63840	-4.88000
C	33.35360	-26.74140	-4.92170
H	33.47760	-27.58750	-5.59850
H	32.28070	-26.59930	-4.79540
C	33.98410	-27.07470	-3.56370
H	35.05260	-27.25080	-3.69280
H	33.89020	-26.21310	-2.90350
C	33.33740	-28.29530	-2.89610
H	33.40930	-29.15630	-3.56180
H	32.27430	-28.10450	-2.75160

C	33.97960	-28.63820	-1.54530
H	35.04100	-28.84530	-1.68680
H	33.91950	-27.77570	-0.88090
C	33.31580	-29.84180	-0.86840
H	33.37910	-30.73230	-1.49430
H	33.79770	-30.06810	0.08290
H	32.26250	-29.64750	-0.66800
O	30.90050	-13.62770	-20.58920
O	30.40420	-15.28770	-22.04750
O	32.04200	-17.69850	-21.30740
H	31.17840	-18.09050	-21.31200
C	30.62190	-13.92000	-21.91720
C	29.34740	-13.17390	-22.34350
H	29.48370	-12.10020	-22.20860
H	29.13560	-13.37360	-23.39420
H	28.49180	-13.49710	-21.75150
C	31.82870	-13.51590	-22.77310
H	31.64670	-13.76970	-23.81760
H	31.99560	-12.44100	-22.69500
H	32.72860	-14.02980	-22.43910

4-alpha monomer

C	1.77930000	6.24080000	-5.28770000
C	-0.41920000	7.76750000	-6.63820000
O	2.11250000	5.34980000	-6.07120000
O	-0.45820000	8.91860000	-6.20460000
N	2.62780000	7.18560000	-4.86900000
N	-0.09670000	7.48790000	-7.90350000
H	2.25290000	7.88010000	-4.21900000
H	-0.03350000	6.49560000	-8.15190000
C	0.35470000	6.28570000	-4.69480000
H	0.32320000	7.08180000	-3.95020000
C	0.05450000	4.96430000	-3.96340000
H	0.15350000	4.13330000	-4.65780000
H	0.80680000	4.79580000	-3.19560000
C	-2.11740000	6.73620000	-5.00610000
H	-2.06980000	7.59130000	-4.32800000
C	-1.33660000	4.91650000	-3.31060000
H	-1.55320000	3.87410000	-3.07490000
C	-2.47680000	5.46960000	-4.19730000
H	-3.34970000	5.68390000	-3.58130000
C	0.27280000	8.45660000	-8.91820000
H	-0.35800000	9.34390000	-8.83820000
H	0.07980000	8.01740000	-9.89730000
C	4.06160000	7.23590000	-5.12270000
H	4.44820000	6.25300000	-5.40010000
H	4.53380000	7.49180000	-4.17370000
C	-0.75890000	6.57040000	-5.73660000
H	-0.84460000	5.69600000	-6.37580000
C	1.75320000	8.82580000	-8.78380000
H	2.36160000	7.92470000	-8.87610000
H	1.93760000	9.22200000	-7.78400000
C	2.19420000	9.85620000	-9.82460000
H	2.05350000	9.44960000	-10.82620000
H	1.55790000	10.73910000	-9.75770000
C	3.65600000	10.26200000	-9.62710000
H	3.79250000	10.63220000	-8.61050000
H	4.29390000	9.38260000	-9.72280000
C	4.10190000	11.33560000	-10.62270000
H	3.98950000	10.96170000	-11.64030000
H	3.44880000	12.20540000	-10.54290000
C	5.55250000	11.76110000	-10.38410000
H	6.20680000	10.89490000	-10.48660000
H	5.66250000	12.10920000	-9.35690000
C	6.00130000	12.86480000	-11.34630000
H	5.89410000	12.51900000	-12.37450000

H	5.34440000	13.72940000	-11.24350000
C	7.45070000	13.28970000	-11.09370000
H	8.10640000	12.42590000	-11.20560000
H	7.55570000	13.62300000	-10.06110000
C	7.90320000	14.40660000	-12.04010000
H	7.79120000	14.07640000	-13.07320000
H	7.25040000	15.27240000	-11.92170000
C	9.35630000	14.82280000	-11.78950000
H	10.00680000	13.95600000	-11.90880000
H	9.46680000	15.14960000	-10.75530000
C	9.81270000	15.94220000	-12.73250000
H	9.69200000	15.61810000	-13.76680000
H	9.16690000	16.81200000	-12.60560000
C	11.27100000	16.34760000	-12.49050000
H	11.39040000	16.67120000	-11.45630000
H	11.91440000	15.47640000	-12.61610000
C	11.73120000	17.46580000	-13.43430000
H	11.60320000	17.14440000	-14.46850000
H	11.09190000	18.33970000	-13.30280000
C	13.19380000	17.86180000	-13.19910000
H	13.31980000	18.18180000	-12.16440000
H	13.83140000	16.98720000	-13.33030000
C	13.65810000	18.98020000	-14.14140000
H	13.53550000	18.66300000	-15.17770000
H	13.02300000	19.85740000	-14.01220000
C	15.11870000	19.37490000	-13.90240000
H	15.26960000	19.72340000	-12.88100000
H	15.42210000	20.17730000	-14.57500000
H	15.78720000	18.53060000	-14.06900000
C	4.43260000	8.28150000	-6.18990000
H	3.85800000	9.19360000	-6.02900000
H	4.15000000	7.91030000	-7.17570000
C	5.93480000	8.61410000	-6.16140000
H	6.19740000	8.94980000	-5.15710000
H	6.51230000	7.70590000	-6.33920000
C	6.35740000	9.69560000	-7.16970000
H	5.71390000	10.56860000	-7.05820000
H	6.20920000	9.32780000	-8.18560000
C	7.82380000	10.11950000	-6.97910000
H	7.96160000	10.48220000	-5.95950000
H	8.46910000	9.24600000	-7.08130000
C	8.28020000	11.20800000	-7.96380000
H	7.62380000	12.07370000	-7.87590000
H	8.17530000	10.84410000	-8.98630000
C	9.73480000	11.64360000	-7.72270000
H	9.83910000	12.00510000	-6.69900000
H	10.39030000	10.77590000	-7.80690000
C	10.20650000	12.73580000	-8.69580000
H	9.55530000	13.60490000	-8.60650000
H	10.10440000	12.38100000	-9.72160000
C	11.66270000	13.16000000	-8.44810000
H	11.76970000	13.51020000	-7.42120000
H	12.31360000	12.28990000	-8.54160000
C	12.13620000	14.25940000	-9.41170000
H	12.02650000	13.91660000	-10.44070000
H	11.49080000	15.13150000	-9.31030000
C	13.59540000	14.67240000	-9.16760000
H	14.24120000	13.80000000	-9.27350000
H	13.71020000	15.01200000	-8.13800000
C	14.06710000	15.77890000	-10.12310000
H	13.95030000	15.44680000	-11.15490000
H	13.42590000	16.65270000	-10.00960000
C	15.52860000	16.18290000	-9.88250000
H	16.17080000	15.30950000	-10.00250000
H	15.65010000	16.51040000	-8.84980000
C	15.99770000	17.29840000	-10.82770000
H	15.87810000	16.97700000	-11.86270000
H	15.35700000	18.17120000	-10.70360000

C	17.45940000	17.69840000	-10.58450000
H	18.10500000	16.82980000	-10.71970000
H	17.58640000	18.01400000	-9.54890000
C	17.92450000	18.82550000	-11.51300000
H	17.86620000	18.52810000	-12.55980000
H	18.95830000	19.10080000	-11.30320000
H	17.31260000	19.71800000	-11.38740000
O	-1.27060000	5.62110000	-2.08490000
Si	-2.28330000	5.34840000	-0.75540000
C	-2.99490000	3.60250000	-0.81420000
H	-3.66430000	3.42160000	0.02550000
H	-3.56480000	3.43040000	-1.72670000
H	-2.21000000	2.85110000	-0.76790000
C	-3.69100000	6.59640000	-0.79650000
H	-3.31420000	7.60210000	-0.96170000
H	-4.39570000	6.38780000	-1.59930000
H	-4.25310000	6.60210000	0.13560000
C	-1.22780000	5.57520000	0.82040000
C	-0.64210000	6.99500000	0.83230000
H	-1.42920000	7.74800000	0.83510000
H	-0.02000000	7.16600000	1.71090000
H	-0.02290000	7.16740000	-0.04920000
C	-2.10460000	5.36050000	2.06600000
H	-2.92460000	6.07790000	2.10620000
H	-2.54150000	4.36140000	2.07860000
H	-1.52590000	5.47540000	2.98300000
C	-0.08040000	4.55090000	0.80500000
H	-0.45620000	3.52750000	0.79530000
H	0.54720000	4.67710000	-0.07850000
H	0.56080000	4.65610000	1.68040000
O	-2.90880000	4.52640000	-5.15450000
H	-3.32180000	5.11940000	-5.78650000
O	-3.19800000	6.91440000	-5.92670000
H	-3.14600000	7.79950000	-6.26460000

4-alpha dimer

C	2.48860000	2.07920000	-8.46470000
C	0.05160000	3.72000000	-8.97610000
O	2.67680000	1.15970000	-9.26370000
O	0.17810000	4.80150000	-8.40830000
N	3.28770000	3.14790000	-8.36790000
N	0.07790000	3.63150000	-10.31130000
H	3.02280000	3.86400000	-7.69750000
H	-0.07710000	2.70630000	-10.71840000
C	1.29790000	2.04630000	-7.50190000
H	1.50810000	2.79310000	-6.73340000
C	1.19700000	0.68060000	-6.79570000
H	0.86200000	-0.07970000	-7.50430000
H	2.16980000	0.34530000	-6.43840000
C	-1.11810000	2.53020000	-7.01900000
H	-0.86820000	3.39860000	-6.41550000
C	0.20440000	0.76160000	-5.62970000
H	0.05700000	-0.23140000	-5.21060000
C	-1.16830000	1.29110000	-6.09180000
H	-1.78030000	1.51560000	-5.21620000
C	0.31630000	4.74680000	-11.22290000
H	-0.36150000	5.56640000	-10.97830000
H	0.06440000	4.42000000	-12.23200000
C	4.46050000	3.38160000	-9.19240000
H	4.19690000	3.24780000	-10.24250000
H	5.21730000	2.63220000	-8.95580000
C	-0.06470000	2.41580000	-8.15560000
H	-0.35980000	1.60750000	-8.82250000
C	1.78150000	5.22290000	-11.17010000
H	2.43650000	4.38090000	-11.39150000

H	2.02260000	5.53660000	-10.15390000
C	2.09580000	6.38270000	-12.12910000
H	1.90830000	6.06810000	-13.15590000
H	1.41790000	7.21370000	-11.93180000
C	3.54980000	6.86410000	-11.99130000
H	3.71880000	7.20640000	-10.96990000
H	4.22090000	6.01920000	-12.14380000
C	3.92490000	7.98780000	-12.97120000
H	3.77290000	7.64530000	-13.99510000
H	3.25610000	8.83700000	-12.83070000
C	5.38170000	8.44670000	-12.79660000
H	6.04440000	7.59030000	-12.91780000
H	5.52910000	8.80230000	-11.77690000
C	5.79640000	9.54910000	-13.78400000
H	5.67180000	9.18890000	-14.80570000
H	5.12990000	10.40550000	-13.67870000
C	7.24950000	10.00350000	-13.57530000
H	7.91290000	9.14490000	-13.67400000
H	7.37350000	10.36740000	-12.55570000
C	7.68580000	11.09810000	-14.56070000
H	7.58250000	10.72970000	-15.58200000
H	7.01680000	11.95490000	-14.47440000
C	9.13330000	11.55580000	-14.32600000
H	9.80080000	10.69860000	-14.40800000
H	9.23770000	11.92670000	-13.30680000
C	9.58010000	12.64580000	-15.31050000
H	9.48710000	12.27240000	-16.33110000
H	8.90940000	13.50240000	-15.23400000
C	11.02450000	13.10580000	-15.06500000
H	11.12040000	13.48220000	-14.04690000
H	11.69420000	12.24940000	-15.13810000
C	11.47330000	14.19130000	-16.05300000
H	11.37740000	13.81450000	-17.07210000
H	10.80520000	15.05010000	-15.97770000
C	12.91920000	14.64760000	-15.81320000
H	13.01950000	15.02740000	-14.79670000
H	13.58620000	13.78890000	-15.88490000
C	13.36500000	15.72830000	-16.80780000
H	13.25120000	15.35830000	-17.82750000
H	12.71210000	16.59750000	-16.72080000
C	14.81770000	16.16480000	-16.59180000
H	14.96710000	16.56090000	-15.58780000
H	15.09970000	16.94440000	-17.29980000
H	15.50420000	15.32980000	-16.72850000
C	5.01300000	4.79590000	-8.96390000
H	5.28210000	4.92030000	-7.91380000
H	4.23500000	5.53210000	-9.16970000
C	6.23530000	5.08070000	-9.84630000
H	7.01560000	4.35110000	-9.62540000
H	5.96770000	4.93380000	-10.89300000
C	6.79650000	6.49720000	-9.66330000
H	7.06440000	6.65330000	-8.61750000
H	6.02510000	7.23180000	-9.89710000
C	8.02600000	6.73580000	-10.54970000
H	8.79180000	5.99720000	-10.30900000
H	7.75980000	6.57050000	-11.59400000
C	8.61620000	8.14200000	-10.39460000
H	8.87710000	8.31450000	-9.34990000
H	7.86260000	8.88660000	-10.65250000
C	9.86090000	8.33560000	-11.27060000
H	10.60810000	7.58620000	-11.00650000
H	9.60360000	8.15670000	-12.31460000
C	10.47710000	9.73180000	-11.13060000
H	10.73180000	9.91260000	-10.08600000
H	9.73870000	10.48560000	-11.40470000
C	11.73300000	9.89570000	-11.99610000
H	12.46800000	9.14100000	-11.71410000
H	11.48340000	9.70680000	-13.04010000

C	12.36270000	11.28680000	-11.86570000
H	11.63190000	12.04470000	-12.14840000
H	12.61430000	11.47320000	-10.82150000
C	13.62360000	11.43640000	-12.72600000
H	13.37760000	11.23830000	-13.76910000
H	14.35460000	10.68200000	-12.43300000
C	14.25600000	12.82750000	-12.60570000
H	13.52580000	13.58430000	-12.89300000
H	14.50860000	13.02070000	-11.56310000
C	15.51610000	12.97210000	-13.46810000
H	15.26890000	12.76110000	-14.50820000
H	16.25000000	12.22340000	-13.16740000
C	16.14290000	14.36750000	-13.36460000
H	15.40730000	15.11800000	-13.65480000
H	16.39940000	14.57160000	-12.32490000
C	17.39790000	14.51150000	-14.23520000
H	17.15190000	14.29320000	-15.27460000
H	18.13950000	13.77190000	-13.93180000
C	18.01840000	15.90870000	-14.14730000
H	17.31340000	16.67540000	-14.46810000
H	18.90120000	15.98340000	-14.78250000
H	18.32470000	16.13800000	-13.12680000
O	0.73140000	1.61380000	-4.62400000
Si	1.43880000	1.12960000	-3.16210000
C	1.45750000	-0.74680000	-2.97330000
H	1.77630000	-1.03250000	-1.97210000
H	0.47490000	-1.18710000	-3.13240000
H	2.15690000	-1.20670000	-3.66690000
C	0.41530000	1.85900000	-1.76170000
H	0.37960000	2.94440000	-1.81590000
H	-0.60850000	1.49180000	-1.80010000
H	0.82540000	1.59510000	-0.78830000
C	3.23550000	1.78850000	-3.08610000
C	3.22610000	3.32380000	-3.10900000
H	2.68900000	3.73490000	-2.25400000
H	4.23660000	3.73190000	-3.08730000
H	2.74640000	3.69250000	-4.01550000
C	3.90060000	1.29610000	-1.78900000
H	3.35450000	1.63510000	-0.90790000
H	3.94300000	0.20720000	-1.75010000
H	4.92290000	1.66490000	-1.70190000
C	4.02320000	1.27450000	-4.30290000
H	4.00870000	0.18610000	-4.36440000
H	3.60650000	1.66510000	-5.23150000
H	5.06670000	1.58750000	-4.26250000
O	-1.84350000	0.30200000	-6.83420000
H	-2.42620000	0.82190000	-7.38450000
O	-2.44720000	2.69890000	-7.50430000
H	-2.92500000	3.12980000	-6.79990000
C	1.77930000	6.24080000	-5.28770000
C	-0.41920000	7.76750000	-6.63820000
O	2.11250000	5.34980000	-6.07120000
O	-0.45820000	8.91860000	-6.20460000
N	2.62780000	7.18560000	-4.86900000
N	-0.09670000	7.48790000	-7.90350000
H	2.25290000	7.88010000	-4.21900000
H	-0.03350000	6.49560000	-8.15190000
C	0.35470000	6.28570000	-4.69480000
H	0.32320000	7.08180000	-3.95020000
C	0.05450000	4.96430000	-3.96340000
H	0.15350000	4.13330000	-4.65780000
H	0.80680000	4.79580000	-3.19560000
C	-2.11740000	6.73620000	-5.00610000
H	-2.06980000	7.59130000	-4.32800000
C	-1.33660000	4.91650000	-3.31060000
H	-1.55320000	3.87410000	-3.07490000
C	-2.47680000	5.46960000	-4.19730000
H	-3.34970000	5.68390000	-3.58130000

C	0.27280000	8.45660000	-8.91820000
H	-0.35800000	9.34390000	-8.83820000
H	0.07980000	8.01740000	-9.89730000
C	4.06160000	7.23590000	-5.12270000
H	4.44820000	6.25300000	-5.40010000
H	4.53380000	7.49180000	-4.17370000
C	-0.75890000	6.57040000	-5.73660000
H	-0.84460000	5.69600000	-6.37580000
C	1.75320000	8.82580000	-8.78380000
H	2.36160000	7.92470000	-8.87610000
H	1.93760000	9.22200000	-7.78400000
C	2.19420000	9.85620000	-9.82460000
H	2.05350000	9.44960000	-10.82620000
H	1.55790000	10.73910000	-9.75770000
C	3.65600000	10.26200000	-9.62710000
H	3.79250000	10.63220000	-8.61050000
H	4.29390000	9.38260000	-9.72280000
C	4.10190000	11.33560000	-10.62270000
H	3.98950000	10.96170000	-11.64030000
H	3.44880000	12.20540000	-10.54290000
C	5.55250000	11.76110000	-10.38410000
H	6.20680000	10.89490000	-10.48660000
H	5.66250000	12.10920000	-9.35690000
C	6.00130000	12.86480000	-11.34630000
H	5.89410000	12.51900000	-12.37450000
H	5.34440000	13.72940000	-11.24350000
C	7.45070000	13.28970000	-11.09370000
H	8.10640000	12.42590000	-11.20560000
H	7.55570000	13.62300000	-10.06110000
C	7.90320000	14.40660000	-12.04010000
H	7.79120000	14.07640000	-13.07320000
H	7.25040000	15.27240000	-11.92170000
C	9.35630000	14.82280000	-11.78950000
H	10.00680000	13.95600000	-11.90880000
H	9.46680000	15.14960000	-10.75530000
C	9.81270000	15.94220000	-12.73250000
H	9.69200000	15.61810000	-13.76680000
H	9.16690000	16.81200000	-12.60560000
C	11.27100000	16.34760000	-12.49050000
H	11.39040000	16.67120000	-11.45630000
H	11.91440000	15.47640000	-12.61610000
C	11.73120000	17.46580000	-13.43430000
H	11.60320000	17.14440000	-14.46850000
H	11.09190000	18.33970000	-13.30280000
C	13.19380000	17.86180000	-13.19910000
H	13.31980000	18.18180000	-12.16440000
H	13.83140000	16.98720000	-13.33030000
C	13.65810000	18.98020000	-14.14140000
H	13.53550000	18.66300000	-15.17770000
H	13.02300000	19.85740000	-14.01220000
C	15.11870000	19.37490000	-13.90240000
H	15.26960000	19.72340000	-12.88100000
H	15.42210000	20.17730000	-14.57500000
H	15.78720000	18.53060000	-14.06900000
C	4.43260000	8.28150000	-6.18990000
H	3.85800000	9.19360000	-6.02900000
H	4.15000000	7.91030000	-7.17570000
C	5.93480000	8.61410000	-6.16140000
H	6.19740000	8.94980000	-5.15710000
H	6.51230000	7.70590000	-6.33920000
C	6.35740000	9.69560000	-7.16970000
H	5.71390000	10.56860000	-7.05820000
H	6.20920000	9.32780000	-8.18560000
C	7.82380000	10.11950000	-6.97910000
H	7.96160000	10.48220000	-5.95950000
H	8.46910000	9.24600000	-7.08130000
C	8.28020000	11.20800000	-7.96380000
H	7.62380000	12.07370000	-7.87590000

H	8.17530000	10.84410000	-8.98630000
C	9.73480000	11.64360000	-7.72270000
H	9.83910000	12.00510000	-6.69900000
H	10.39030000	10.77590000	-7.80690000
C	10.20650000	12.73580000	-8.69580000
H	9.55530000	13.60490000	-8.60650000
H	10.10440000	12.38100000	-9.72160000
C	11.66270000	13.16000000	-8.44810000
H	11.76970000	13.51020000	-7.42120000
H	12.31360000	12.28990000	-8.54160000
C	12.13620000	14.25940000	-9.41170000
H	12.02650000	13.91660000	-10.44070000
H	11.49080000	15.13150000	-9.31030000
C	13.59540000	14.67240000	-9.16760000
H	14.24120000	13.80000000	-9.27350000
H	13.71020000	15.01200000	-8.13800000
C	14.06710000	15.77890000	-10.12310000
H	13.95030000	15.44680000	-11.15490000
H	13.42590000	16.65270000	-10.00960000
C	15.52860000	16.18290000	-9.88250000
H	16.17080000	15.30950000	-10.00250000
H	15.65010000	16.51040000	-8.84980000
C	15.99770000	17.29840000	-10.82770000
H	15.87810000	16.97700000	-11.86270000
H	15.35700000	18.17120000	-10.70360000
C	17.45940000	17.69840000	-10.58450000
H	18.10500000	16.82980000	-10.71970000
H	17.58640000	18.01400000	-9.54890000
C	17.92450000	18.82550000	-11.51300000
H	17.86620000	18.52810000	-12.55980000
H	18.95830000	19.10080000	-11.30320000
H	17.31260000	19.71800000	-11.38740000
O	-1.27060000	5.62110000	-2.08490000
Si	-2.28330000	5.34840000	-0.75540000
C	-2.99490000	3.60250000	-0.81420000
H	-3.66430000	3.42160000	0.02550000
H	-3.56480000	3.43040000	-1.72670000
H	-2.21000000	2.85110000	-0.76790000
C	-3.69100000	6.59640000	-0.79650000
H	-3.31420000	7.60210000	-0.96170000
H	-4.39570000	6.38780000	-1.59930000
H	-4.25310000	6.60210000	0.13560000
C	-1.22780000	5.57520000	0.82040000
C	-0.64210000	6.99500000	0.83230000
H	-1.42920000	7.74800000	0.83510000
H	-0.02000000	7.16600000	1.71090000
H	-0.02290000	7.16740000	-0.04920000
C	-2.10460000	5.36050000	2.06600000
H	-2.92460000	6.07790000	2.10620000
H	-2.54150000	4.36140000	2.07860000
H	-1.52590000	5.47540000	2.98300000
C	-0.08040000	4.55090000	0.80500000
H	-0.45620000	3.52750000	0.79530000
H	0.54720000	4.67710000	-0.07850000
H	0.56080000	4.65610000	1.68040000
O	-2.90880000	4.52640000	-5.15450000
H	-3.32180000	5.11940000	-5.78650000
O	-3.19800000	6.91440000	-5.92670000
H	-3.14600000	7.79950000	-6.26460000

4-alpha tetramer

C	2.03620000	-1.71850000	-11.68240000
C	-0.30310000	0.13300000	-11.97910000
O	2.05230000	-2.31500000	-12.75780000
O	-0.52140000	1.13990000	-11.30960000

N	3.03780000	-0.93330000	-11.27270000
N	0.10470000	0.19970000	-13.25250000
H	2.90940000	-0.38290000	-10.42510000
H	0.29870000	-0.67860000	-13.71750000
C	0.81360000	-1.83780000	-10.76560000
H	1.03630000	-1.28400000	-9.85120000
C	0.59250000	-3.31780000	-10.39930000
H	0.29640000	-3.87880000	-11.28780000
H	1.52040000	-3.77310000	-10.05150000
C	-1.60580000	-1.30700000	-10.28100000
H	-1.33550000	-0.62890000	-9.46860000
C	-0.49390000	-3.46600000	-9.32910000
H	-0.72840000	-4.52420000	-9.20530000
C	-1.79690000	-2.72080000	-9.68270000
H	-2.42850000	-2.65220000	-8.79530000
C	0.48610000	1.42160000	-13.94610000
H	-0.19980000	2.23330000	-13.69620000
H	0.38890000	1.24770000	-15.01820000
C	4.29180000	-0.77040000	-11.99080000
H	4.11380000	-0.76920000	-13.06780000
H	4.93150000	-1.62800000	-11.77780000
C	-0.50020000	-1.27050000	-11.37570000
H	-0.80990000	-1.92640000	-12.19140000
C	1.93390000	1.80550000	-13.60050000
H	2.58440000	0.95220000	-13.79440000
H	2.01250000	2.01530000	-12.53200000
C	2.44190000	3.01140000	-14.39920000
H	2.34670000	2.80110000	-15.46540000
H	1.81010000	3.87660000	-14.19720000
C	3.90500000	3.34830000	-14.07670000
H	3.99820000	3.60700000	-13.02170000
H	4.52290000	2.46300000	-14.23160000
C	4.44560000	4.49450000	-14.94000000
H	4.36280000	4.22120000	-15.99300000
H	3.82250000	5.37840000	-14.80320000
C	5.90620000	4.84060000	-14.61880000
H	6.52460000	3.94880000	-14.72720000
H	5.98910000	5.14880000	-13.57630000
C	6.44980000	5.94850000	-15.53020000
H	6.38380000	5.62380000	-16.56950000
H	5.81570000	6.83130000	-15.44520000
C	7.90110000	6.33000000	-15.20820000
H	8.53460000	5.44500000	-15.27690000
H	7.96760000	6.67850000	-14.17720000
C	8.43430000	7.41340000	-16.15530000
H	8.38280000	7.05060000	-17.18280000
H	7.78530000	8.28840000	-16.10750000
C	9.87600000	7.83110000	-15.83570000
H	10.52540000	6.95590000	-15.87490000
H	9.92880000	8.20960000	-14.81450000
C	10.39750000	8.89780000	-16.80840000
H	10.35540000	8.50880000	-17.82670000
H	9.73660000	9.76500000	-16.78480000
C	11.83210000	9.34160000	-16.49250000
H	11.87530000	9.74140000	-15.47900000
H	12.49270000	8.47420000	-16.51090000
C	12.34630000	10.39580000	-17.48270000
H	12.31040000	9.98970000	-18.49450000
H	11.67870000	11.25820000	-17.47490000
C	13.77670000	10.85450000	-17.16880000
H	13.81390000	11.26470000	-16.15940000
H	14.44410000	9.99210000	-17.17610000
C	14.28710000	11.90360000	-18.16620000
H	14.26070000	11.49300000	-19.17640000
H	13.61790000	12.76480000	-18.16850000
C	15.71130000	12.37120000	-17.84920000
H	15.77010000	12.81250000	-16.85480000
H	16.04250000	13.12410000	-18.56470000

H	16.41690000	11.54110000	-17.88940000
C	4.98430000	0.53290000	-11.57630000
H	5.17030000	0.52510000	-10.50140000
H	4.31440000	1.37140000	-11.76730000
C	6.30390000	0.74910000	-12.32560000
H	6.98610000	-0.07210000	-12.10100000
H	6.11950000	0.70940000	-13.40010000
C	6.97210000	2.08430000	-11.97480000
H	7.16410000	2.12860000	-10.90200000
H	6.28810000	2.90210000	-12.20210000
C	8.28390000	2.28550000	-12.74270000
H	8.97720000	1.48120000	-12.49230000
H	8.09250000	2.20240000	-13.81360000
C	8.94370000	3.63860000	-12.45080000
H	9.14470000	3.72640000	-11.38230000
H	8.25190000	4.44230000	-12.70350000
C	10.24780000	3.81850000	-13.23810000
H	10.94620000	3.02500000	-12.96820000
H	10.04620000	3.69950000	-14.30360000
C	10.90710000	5.18200000	-12.99690000
H	11.11970000	5.30300000	-11.93400000
H	10.21020000	5.97570000	-13.26630000
C	12.20220000	5.34240000	-13.80360000
H	12.90410000	4.55600000	-13.52230000
H	11.98880000	5.19770000	-14.86360000
C	12.86280000	6.71170000	-13.60120000
H	12.16200000	7.49840000	-13.88100000
H	13.08500000	6.85660000	-12.54320000
C	14.15060000	6.85820000	-14.42250000
H	13.92770000	6.69780000	-15.47830000
H	14.85460000	6.07600000	-14.13510000
C	14.81260000	8.23050000	-14.24480000
H	14.11050000	9.01290000	-14.53350000
H	15.03830000	8.39130000	-13.18980000
C	16.09820000	8.36720000	-15.07150000
H	15.87220000	8.19840000	-16.12530000
H	16.80180000	7.58660000	-14.77890000
C	16.76190000	9.74040000	-14.90550000
H	16.06130000	10.52060000	-15.20370000
H	16.98410000	9.91110000	-13.85130000
C	18.05090000	9.86970000	-15.72840000
H	17.83170000	9.69930000	-16.78330000
H	18.75540000	9.09170000	-15.43120000
C	18.71560000	11.24040000	-15.56540000
H	18.05360000	12.04060000	-15.89550000
H	19.62990000	11.30290000	-16.15600000
H	18.97910000	11.43020000	-14.52450000
O	0.03070000	-2.96960000	-8.11550000
Si	0.53510000	-3.94130000	-6.83170000
C	1.85300000	-2.97180000	-5.90940000
H	2.17060000	-3.47920000	-5.00000000
H	2.73320000	-2.81130000	-6.53010000
H	1.46900000	-1.99470000	-5.63570000
C	1.28380000	-5.54450000	-7.48920000
H	0.55350000	-6.13810000	-8.03690000
H	2.11680000	-5.34740000	-8.16210000
H	1.65900000	-6.16540000	-6.67710000
C	-0.96310000	-4.30500000	-5.70310000
C	-1.98920000	-5.16130000	-6.46550000
H	-1.56010000	-6.11020000	-6.78810000
H	-2.85560000	-5.38960000	-5.84440000
H	-2.35630000	-4.64880000	-7.35480000
C	-0.48590000	-5.06350000	-4.45270000
H	-0.00470000	-6.00560000	-4.71780000
H	0.23310000	-4.47710000	-3.87980000
H	-1.31860000	-5.29650000	-3.78840000
C	-1.61020000	-2.97340000	-5.29010000
H	-0.91540000	-2.35100000	-4.72850000

H	-1.92520000	-2.39930000	-6.16300000
H	-2.48980000	-3.13020000	-4.66570000
O	-2.51830000	-3.44800000	-10.64830000
H	-3.07620000	-2.77010000	-11.02550000
O	-2.87730000	-0.92220000	-10.80780000
H	-2.86620000	0.02170000	-10.90920000
C	2.48860000	2.07920000	-8.46470000
C	0.05160000	3.72000000	-8.97610000
O	2.67680000	1.15970000	-9.26370000
O	0.17810000	4.80150000	-8.40830000
N	3.28770000	3.14790000	-8.36790000
N	0.07790000	3.63150000	-10.31130000
H	3.02280000	3.86400000	-7.69750000
H	-0.07710000	2.70630000	-10.71840000
C	1.29790000	2.04630000	-7.50190000
H	1.50810000	2.79310000	-6.73340000
C	1.19700000	0.68060000	-6.79570000
H	0.86200000	-0.07970000	-7.50430000
H	2.16980000	0.34530000	-6.43840000
C	-1.11810000	2.53020000	-7.01900000
H	-0.86820000	3.39860000	-6.41550000
C	0.20440000	0.76160000	-5.62970000
H	0.05700000	-0.23140000	-5.21060000
C	-1.16830000	1.29110000	-6.09180000
H	-1.78030000	1.51560000	-5.21620000
C	0.31630000	4.74680000	-11.22290000
H	-0.36150000	5.56640000	-10.97830000
H	0.06440000	4.42000000	-12.23200000
C	4.46050000	3.38160000	-9.19240000
H	4.19690000	3.24780000	-10.24250000
H	5.21730000	2.63220000	-8.95580000
C	-0.06470000	2.41580000	-8.15560000
H	-0.35980000	1.60750000	-8.82250000
C	1.78150000	5.22290000	-11.17010000
H	2.43650000	4.38090000	-11.39150000
H	2.02260000	5.53660000	-10.15390000
C	2.09580000	6.38270000	-12.12910000
H	1.90830000	6.06810000	-13.15590000
H	1.41790000	7.21370000	-11.93180000
C	3.54980000	6.86410000	-11.99130000
H	3.71880000	7.20640000	-10.96990000
H	4.22090000	6.01920000	-12.14380000
C	3.92490000	7.98780000	-12.97120000
H	3.77290000	7.64530000	-13.99510000
H	3.25610000	8.83700000	-12.83070000
C	5.38170000	8.44670000	-12.79660000
H	6.04440000	7.59030000	-12.91780000
H	5.52910000	8.80230000	-11.77690000
C	5.79640000	9.54910000	-13.78400000
H	5.67180000	9.18890000	-14.80570000
H	5.12990000	10.40550000	-13.67870000
C	7.24950000	10.00350000	-13.57530000
H	7.91290000	9.14490000	-13.67400000
H	7.37350000	10.36740000	-12.55570000
C	7.68580000	11.09810000	-14.56070000
H	7.58250000	10.72970000	-15.58200000
H	7.01680000	11.95490000	-14.47440000
C	9.13330000	11.55580000	-14.32600000
H	9.80080000	10.69860000	-14.40800000
H	9.23770000	11.92670000	-13.30680000
C	9.58010000	12.64580000	-15.31050000
H	9.48710000	12.27240000	-16.33110000
H	8.90940000	13.50240000	-15.23400000
C	11.02450000	13.10580000	-15.06500000
H	11.12040000	13.48220000	-14.04690000
H	11.69420000	12.24940000	-15.13810000
C	11.47330000	14.19130000	-16.05300000
H	11.37740000	13.81450000	-17.07210000

H	10.80520000	15.05010000	-15.97770000
C	12.91920000	14.64760000	-15.81320000
H	13.01950000	15.02740000	-14.79670000
H	13.58620000	13.78890000	-15.88490000
C	13.36500000	15.72830000	-16.80780000
H	13.25120000	15.35830000	-17.82750000
H	12.71210000	16.59750000	-16.72080000
C	14.81770000	16.16480000	-16.59180000
H	14.96710000	16.56090000	-15.58780000
H	15.09970000	16.94440000	-17.29980000
H	15.50420000	15.32980000	-16.72850000
C	5.01300000	4.79590000	-8.96390000
H	5.28210000	4.92030000	-7.91380000
H	4.23500000	5.53210000	-9.16970000
C	6.23530000	5.08070000	-9.84630000
H	7.01560000	4.35110000	-9.62540000
H	5.96770000	4.93380000	-10.89300000
C	6.79650000	6.49720000	-9.66330000
H	7.06440000	6.65330000	-8.61750000
H	6.02510000	7.23180000	-9.89710000
C	8.02600000	6.73580000	-10.54970000
H	8.79180000	5.99720000	-10.30900000
H	7.75980000	6.57050000	-11.59400000
C	8.61620000	8.14200000	-10.39460000
H	8.87710000	8.31450000	-9.34990000
H	7.86260000	8.88660000	-10.65250000
C	9.86090000	8.33560000	-11.27060000
H	10.60810000	7.58620000	-11.00650000
H	9.60360000	8.15670000	-12.31460000
C	10.47710000	9.73180000	-11.13060000
H	10.73180000	9.91260000	-10.08600000
H	9.73870000	10.48560000	-11.40470000
C	11.73300000	9.89570000	-11.99610000
H	12.46800000	9.14100000	-11.71410000
H	11.48340000	9.70680000	-13.04010000
C	12.36270000	11.28680000	-11.86570000
H	11.63190000	12.04470000	-12.14840000
H	12.61430000	11.47320000	-10.82150000
C	13.62360000	11.43640000	-12.72600000
H	13.37760000	11.23830000	-13.76910000
H	14.35460000	10.68200000	-12.43300000
C	14.25600000	12.82750000	-12.60570000
H	13.52580000	13.58430000	-12.89300000
H	14.50860000	13.02070000	-11.56310000
C	15.51610000	12.97210000	-13.46810000
H	15.26890000	12.76110000	-14.50820000
H	16.25000000	12.22340000	-13.16740000
C	16.14290000	14.36750000	-13.36460000
H	15.40730000	15.11800000	-13.65480000
H	16.39940000	14.57160000	-12.32490000
C	17.39790000	14.51150000	-14.23520000
H	17.15190000	14.29320000	-15.27460000
H	18.13950000	13.77190000	-13.93180000
C	18.01840000	15.90870000	-14.14730000
H	17.31340000	16.67540000	-14.46810000
H	18.90120000	15.98340000	-14.78250000
H	18.32470000	16.13800000	-13.12680000
O	0.73140000	1.61380000	-4.62400000
Si	1.43880000	1.12960000	-3.16210000
C	1.45750000	-0.74680000	-2.97330000
H	1.77630000	-1.03250000	-1.97210000
H	0.47490000	-1.18710000	-3.13240000
H	2.15690000	-1.20670000	-3.66690000
C	0.41530000	1.85900000	-1.76170000
H	0.37960000	2.94440000	-1.81590000
H	-0.60850000	1.49180000	-1.80010000
H	0.82540000	1.59510000	-0.78830000
C	3.23550000	1.78850000	-3.08610000

C	3.22610000	3.32380000	-3.10900000
H	2.68900000	3.73490000	-2.25400000
H	4.23660000	3.73190000	-3.08730000
H	2.74640000	3.69250000	-4.01550000
C	3.90060000	1.29610000	-1.78900000
H	3.35450000	1.63510000	-0.90790000
H	3.94300000	0.20720000	-1.75010000
H	4.92290000	1.66490000	-1.70190000
C	4.02320000	1.27450000	-4.30290000
H	4.00870000	0.18610000	-4.36440000
H	3.60650000	1.66510000	-5.23150000
H	5.06670000	1.58750000	-4.26250000
O	-1.84350000	0.30200000	-6.83420000
H	-2.42620000	0.82190000	-7.38450000
O	-2.44720000	2.69890000	-7.50430000
H	-2.92500000	3.12980000	-6.79990000
C	1.77930000	6.24080000	-5.28770000
C	-0.41920000	7.76750000	-6.63820000
O	2.11250000	5.34980000	-6.07120000
O	-0.45820000	8.91860000	-6.20460000
N	2.62780000	7.18560000	-4.86900000
N	-0.09670000	7.48790000	-7.90350000
H	2.25290000	7.88010000	-4.21900000
H	-0.03350000	6.49560000	-8.15190000
C	0.35470000	6.28570000	-4.69480000
H	0.32320000	7.08180000	-3.95020000
C	0.05450000	4.96430000	-3.96340000
H	0.15350000	4.13330000	-4.65780000
H	0.80680000	4.79580000	-3.19560000
C	-2.11740000	6.73620000	-5.00610000
H	-2.06980000	7.59130000	-4.32800000
C	-1.33660000	4.91650000	-3.31060000
H	-1.55320000	3.87410000	-3.07490000
C	-2.47680000	5.46960000	-4.19730000
H	-3.34970000	5.68390000	-3.58130000
C	0.27280000	8.45660000	-8.91820000
H	-0.35800000	9.34390000	-8.83820000
H	0.07980000	8.01740000	-9.89730000
C	4.06160000	7.23590000	-5.12270000
H	4.44820000	6.25300000	-5.40010000
H	4.53380000	7.49180000	-4.17370000
C	-0.75890000	6.57040000	-5.73660000
H	-0.84460000	5.69600000	-6.37580000
C	1.75320000	8.82580000	-8.78380000
H	2.36160000	7.92470000	-8.87610000
H	1.93760000	9.22200000	-7.78400000
C	2.19420000	9.85620000	-9.82460000
H	2.05350000	9.44960000	-10.82620000
H	1.55790000	10.73910000	-9.75770000
C	3.65600000	10.26200000	-9.62710000
H	3.79250000	10.63220000	-8.61050000
H	4.29390000	9.38260000	-9.72280000
C	4.10190000	11.33560000	-10.62270000
H	3.98950000	10.96170000	-11.64030000
H	3.44880000	12.20540000	-10.54290000
C	5.55250000	11.76110000	-10.38410000
H	6.20680000	10.89490000	-10.48660000
H	5.66250000	12.10920000	-9.35690000
C	6.00130000	12.86480000	-11.34630000
H	5.89410000	12.51900000	-12.37450000
H	5.34440000	13.72940000	-11.24350000
C	7.45070000	13.28970000	-11.09370000
H	8.10640000	12.42590000	-11.20560000
H	7.55570000	13.62300000	-10.06110000
C	7.90320000	14.40660000	-12.04010000
H	7.79120000	14.07640000	-13.07320000
H	7.25040000	15.27240000	-11.92170000
C	9.35630000	14.82280000	-11.78950000

H	10.00680000	13.95600000	-11.90880000
H	9.46680000	15.14960000	-10.75530000
C	9.81270000	15.94220000	-12.73250000
H	9.69200000	15.61810000	-13.76680000
H	9.16690000	16.81200000	-12.60560000
C	11.27100000	16.34760000	-12.49050000
H	11.39040000	16.67120000	-11.45630000
H	11.91440000	15.47640000	-12.61610000
C	11.73120000	17.46580000	-13.43430000
H	11.60320000	17.14440000	-14.46850000
H	11.09190000	18.33970000	-13.30280000
C	13.19380000	17.86180000	-13.19910000
H	13.31980000	18.18180000	-12.16440000
H	13.83140000	16.98720000	-13.33030000
C	13.65810000	18.98020000	-14.14140000
H	13.53550000	18.66300000	-15.17770000
H	13.02300000	19.85740000	-14.01220000
C	15.11870000	19.37490000	-13.90240000
H	15.26960000	19.72340000	-12.88100000
H	15.42210000	20.17730000	-14.57500000
H	15.78720000	18.53060000	-14.06900000
C	4.43260000	8.28150000	-6.18990000
H	3.85800000	9.19360000	-6.02900000
H	4.15000000	7.91030000	-7.17570000
C	5.93480000	8.61410000	-6.16140000
H	6.19740000	8.94980000	-5.15710000
H	6.51230000	7.70590000	-6.33920000
C	6.35740000	9.69560000	-7.16970000
H	5.71390000	10.56860000	-7.05820000
H	6.20920000	9.32780000	-8.18560000
C	7.82380000	10.11950000	-6.97910000
H	7.96160000	10.48220000	-5.95950000
H	8.46910000	9.24600000	-7.08130000
C	8.28020000	11.20800000	-7.96380000
H	7.62380000	12.07370000	-7.87590000
H	8.17530000	10.84410000	-8.98630000
C	9.73480000	11.64360000	-7.72270000
H	9.83910000	12.00510000	-6.69900000
H	10.39030000	10.77590000	-7.80690000
C	10.20650000	12.73580000	-8.69580000
H	9.55530000	13.60490000	-8.60650000
H	10.10440000	12.38100000	-9.72160000
C	11.66270000	13.16000000	-8.44810000
H	11.76970000	13.51020000	-7.42120000
H	12.31360000	12.28990000	-8.54160000
C	12.13620000	14.25940000	-9.41170000
H	12.02650000	13.91660000	-10.44070000
H	11.49080000	15.13150000	-9.31030000
C	13.59540000	14.67240000	-9.16760000
H	14.24120000	13.80000000	-9.27350000
H	13.71020000	15.01200000	-8.13800000
C	14.06710000	15.77890000	-10.12310000
H	13.95030000	15.44680000	-11.15490000
H	13.42590000	16.65270000	-10.00960000
C	15.52860000	16.18290000	-9.88250000
H	16.17080000	15.30950000	-10.00250000
H	15.65010000	16.51040000	-8.84980000
C	15.99770000	17.29840000	-10.82770000
H	15.87810000	16.97700000	-11.86270000
H	15.35700000	18.17120000	-10.70360000
C	17.45940000	17.69840000	-10.58450000
H	18.10500000	16.82980000	-10.71970000
H	17.58640000	18.01400000	-9.54890000
C	17.92450000	18.82550000	-11.51300000
H	17.86620000	18.52810000	-12.55980000
H	18.95830000	19.10080000	-11.30320000
H	17.31260000	19.71800000	-11.38740000
O	-1.27060000	5.62110000	-2.08490000

Si	-2.28330000	5.34840000	-0.75540000
C	-2.99490000	3.60250000	-0.81420000
H	-3.66430000	3.42160000	0.02550000
H	-3.56480000	3.43040000	-1.72670000
H	-2.21000000	2.85110000	-0.76790000
C	-3.69100000	6.59640000	-0.79650000
H	-3.31420000	7.60210000	-0.96170000
H	-4.39570000	6.38780000	-1.59930000
H	-4.25310000	6.60210000	0.13560000
C	-1.22780000	5.57520000	0.82040000
C	-0.64210000	6.99500000	0.83230000
H	-1.42920000	7.74800000	0.83510000
H	-0.02000000	7.16600000	1.71090000
H	-0.02290000	7.16740000	-0.04920000
C	-2.10460000	5.36050000	2.06600000
H	-2.92460000	6.07790000	2.10620000
H	-2.54150000	4.36140000	2.07860000
H	-1.52590000	5.47540000	2.98300000
C	-0.08040000	4.55090000	0.80500000
H	-0.45620000	3.52750000	0.79530000
H	0.54720000	4.67710000	-0.07850000
H	0.56080000	4.65610000	1.68040000
O	-2.90880000	4.52640000	-5.15450000
H	-3.32180000	5.11940000	-5.78650000
O	-3.19800000	6.91440000	-5.92670000
H	-3.14600000	7.79950000	-6.26460000
C	1.52580000	10.25290000	-2.68020000
C	-0.02000000	12.02870000	-4.51060000
O	1.67000000	9.11070000	-3.11020000
O	0.15570000	13.19640000	-4.16560000
N	2.54210000	11.11180000	-2.56940000
N	0.41470000	11.54860000	-5.68240000
H	2.33200000	12.04070000	-2.23400000
H	0.20040000	10.57240000	-5.89670000
C	0.13340000	10.77810000	-2.30540000
H	0.26310000	11.71760000	-1.76470000
C	-0.57280000	9.78740000	-1.36180000
H	-0.71880000	8.82940000	-1.86560000
H	0.05090000	9.58550000	-0.49050000
C	-2.13490000	11.59800000	-3.14990000
H	-2.02580000	12.60200000	-2.73330000
C	-1.93220000	10.32900000	-0.90750000
H	-2.43780000	9.57320000	-0.30730000
C	-2.83220000	10.71260000	-2.09620000
H	-3.72550000	11.22250000	-1.73470000
C	1.11230000	12.33060000	-6.69550000
H	0.59480000	13.28250000	-6.82940000
H	1.03970000	11.80300000	-7.64620000
C	3.87700000	10.86510000	-3.08870000
H	3.79730000	10.53510000	-4.12510000
H	4.35180000	10.05700000	-2.52960000
C	-0.73960000	11.05200000	-3.56260000
H	-0.88710000	10.10150000	-4.07240000
C	2.59100000	12.57280000	-6.33650000
H	3.12550000	11.62310000	-6.30090000
H	2.65510000	13.00370000	-5.33700000
C	3.27360000	13.52570000	-7.32910000
H	3.24130000	13.09520000	-8.32960000
H	2.69600000	14.45000000	-7.37800000
C	4.72660000	13.86830000	-6.96140000
H	4.76320000	14.24550000	-5.93870000
H	5.33740000	12.96570000	-6.98070000
C	5.31880000	14.92080000	-7.91160000
H	5.27770000	14.54870000	-8.93530000
H	4.69140000	15.81300000	-7.88870000
C	6.76380000	15.31820000	-7.57350000
H	7.40960000	14.44230000	-7.62870000
H	6.81300000	15.67490000	-6.54410000

C	7.29020000	16.40820000	-8.51980000
H	7.22300000	16.05610000	-9.54940000
H	6.64090000	17.28250000	-8.45500000
C	8.73620000	16.83160000	-8.22310000
H	9.39470000	15.96700000	-8.30080000
H	8.80950000	17.18470000	-7.19400000
C	9.21980000	17.93130000	-9.18010000
H	9.12850000	17.58140000	-10.20880000
H	8.56310000	18.79800000	-9.09340000
C	10.66920000	18.36580000	-8.92030000
H	11.33100000	17.50520000	-9.01300000
H	10.76530000	18.72030000	-7.89350000
C	11.12460000	19.46660000	-9.88890000
H	11.01430000	19.11420000	-10.91490000
H	10.46540000	20.33000000	-9.78970000
C	12.57670000	19.90660000	-9.65720000
H	12.69040000	20.26420000	-8.63330000
H	13.23900000	19.04720000	-9.75900000
C	13.01260000	21.00550000	-10.63630000
H	12.88690000	20.64930000	-11.65910000
H	12.35330000	21.86790000	-10.52900000
C	14.46720000	21.44740000	-10.42690000
H	14.59380000	21.80980000	-9.40600000
H	15.12750000	20.58670000	-10.53150000
C	14.89240000	22.54130000	-11.41660000
H	14.76320000	22.18330000	-12.43890000
H	14.23600000	23.40580000	-11.30950000
C	16.34550000	22.98570000	-11.21740000
H	16.50330000	23.37790000	-10.21260000
H	16.61210000	23.77110000	-11.92510000
H	17.03820000	22.15770000	-11.36750000
C	4.71030000	12.14840000	-3.01230000
H	4.89120000	12.40170000	-1.96640000
H	4.14100000	12.97750000	-3.43550000
C	6.04620000	12.04290000	-3.75750000
H	6.63090000	11.20980000	-3.36500000
H	5.85650000	11.82100000	-4.80850000
C	6.84930000	13.34390000	-3.64060000
H	7.11060000	13.51300000	-2.59490000
H	6.22090000	14.18510000	-3.93700000
C	8.12130000	13.35320000	-4.49530000
H	8.75900000	12.51050000	-4.22530000
H	7.85440000	13.21800000	-5.54400000
C	8.89490000	14.66630000	-4.32150000
H	9.20330000	14.76790000	-3.28000000
H	8.23120000	15.50740000	-4.52660000
C	10.12450000	14.76840000	-5.22980000
H	10.79590000	13.93020000	-5.03890000
H	9.81210000	14.68520000	-6.27100000
C	10.87370000	16.09040000	-5.01820000
H	11.21180000	16.15320000	-3.98290000
H	10.18700000	16.92470000	-5.16690000
C	12.07380000	16.25250000	-5.95730000
H	12.76650000	15.42230000	-5.81290000
H	11.73290000	16.19520000	-6.99130000
C	12.80710000	17.58100000	-5.72950000
H	12.10600000	18.40830000	-5.84690000
H	13.16540000	17.62590000	-4.70010000
C	13.98650000	17.77500000	-6.68970000
H	13.62660000	17.72750000	-7.71770000
H	14.69130000	16.95110000	-6.57090000
C	14.71220000	19.10770000	-6.46090000
H	14.00310000	19.93050000	-6.56050000
H	15.08670000	19.14640000	-5.43710000
C	15.87490000	19.31580000	-7.43930000
H	15.49740000	19.27640000	-8.46140000
H	16.58350000	18.49250000	-7.34010000
C	16.60370000	20.64730000	-7.21330000

H	15.89090000	21.46970000	-7.28740000
H	17.00180000	20.67540000	-6.19790000
C	17.74300000	20.86850000	-8.21730000
H	17.34310000	20.84840000	-9.23080000
H	18.45700000	20.04650000	-8.15250000
C	18.47650000	22.19480000	-7.99130000
H	17.79560000	23.04240000	-8.07350000
H	19.26720000	22.33220000	-8.72940000
H	18.93540000	22.22880000	-7.00280000
O	-1.70640000	11.46450000	-0.09660000
Si	-2.54980000	11.79290000	1.32800000
C	-2.25790000	10.37340000	2.53340000
H	-2.69390000	10.58440000	3.50880000
H	-2.69730000	9.44400000	2.17460000
H	-1.19360000	10.19550000	2.68210000
C	-4.38420000	11.95480000	0.92850000
H	-4.56290000	12.73760000	0.19260000
H	-4.79080000	11.02670000	0.52860000
H	-4.96140000	12.20640000	1.81710000
C	-1.86060000	13.42890000	2.02380000
C	-2.06160000	14.53810000	0.97710000
H	-3.11820000	14.69320000	0.75660000
H	-1.65580000	15.48980000	1.32090000
H	-1.56580000	14.28900000	0.03750000
C	-2.60260000	13.79120000	3.32150000
H	-3.67200000	13.91610000	3.14830000
H	-2.48180000	13.01700000	4.07990000
H	-2.22900000	14.72410000	3.74470000
C	-0.35940000	13.25410000	2.30940000
H	-0.18250000	12.47850000	3.05530000
H	0.18300000	12.96770000	1.40700000
H	0.08610000	14.17630000	2.68330000
O	-3.26720000	9.56710000	-2.78880000
H	-3.50440000	9.93430000	-3.63840000
O	-3.02620000	11.64610000	-4.26660000
H	-2.75350000	12.36110000	-4.82700000

4-alpha hexamer

C	1.75210	10.09470	-2.58580
C	-0.64590	11.69730	-3.39810
O	1.75390	9.19080	-3.41900
O	-0.52940	12.84270	-2.96470
N	2.75520	10.97200	-2.47310
N	-0.56960	11.40900	-4.70530
H	2.67910	11.69550	-1.77310
H	-0.69660	10.44150	-4.98090
C	0.54700	10.28800	-1.65000
H	0.73270	11.17450	-1.03960
C	0.43250	9.08120	-0.69920
H	0.28130	8.17130	-1.27620
H	1.36940	8.93590	-0.16290
C	-1.95350	10.70430	-1.43130
H	-1.80240	11.63180	-0.88260
C	-0.72880	9.23280	0.29700
H	-0.84160	8.30010	0.85280
C	-2.06200	9.55250	-0.40710
H	-2.80550	9.80710	0.34580
C	-0.20270	12.35890	-5.74720
H	-0.83180	13.24650	-5.66890
H	-0.41640	11.90490	-6.71520
C	3.92830	10.97830	-3.33390
H	3.64690	10.72750	-4.35850
H	4.62390	10.20900	-2.99550
C	-0.78380	10.50540	-2.43030
H	-0.99070	9.60790	-3.01360
C	1.28810	12.73490	-5.65720

H	1.89710	11.83570	-5.75680
H	1.50470	13.14030	-4.66820
C	1.71640	13.76460	-6.70910
H	1.59410	13.34270	-7.70720
H	1.05710	14.63120	-6.65570
C	3.16850	14.21910	-6.50620
H	3.28000	14.62790	-5.50140
H	3.83060	13.35490	-6.56170
C	3.61400	15.27100	-7.52980
H	3.52940	14.85990	-8.53620
H	2.93940	16.12630	-7.48990
C	5.05250	15.74580	-7.28500
H	5.72820	14.89210	-7.33250
H	5.13390	16.14680	-6.27450
C	5.50430	16.81100	-8.29240
H	5.42630	16.41150	-9.30380
H	4.82790	17.66460	-8.24820
C	6.94070	17.28600	-8.03710
H	7.61900	16.43540	-8.09870
H	7.02080	17.67010	-7.02020
C	7.38750	18.36940	-9.02730
H	7.29930	17.98920	-10.04540
H	6.71350	19.22360	-8.96070
C	8.82780	18.83600	-8.77820
H	9.50290	17.98510	-8.86410
H	8.92100	19.19990	-7.75520
C	9.26430	19.93730	-9.75310
H	9.15670	19.57830	-10.77710
H	8.59590	20.79340	-9.65690
C	10.71140	20.39150	-9.52100
H	10.82490	20.73820	-8.49430
H	11.37960	19.53820	-9.63180
C	11.13730	21.50510	-10.48700
H	11.00630	21.16270	-11.51400
H	10.47780	22.36480	-10.36340
C	12.59270	21.94410	-10.27850
H	12.72830	22.28140	-9.25120
H	13.25050	21.08550	-10.40700
C	13.01210	23.06130	-11.24370
H	12.86180	22.73320	-12.27280
H	12.36780	23.92920	-11.09950
C	14.47340	23.48220	-11.06020
H	14.66020	23.82460	-10.04300
H	14.73200	24.29640	-11.73730
H	15.15010	22.65330	-11.26480
C	4.59540	12.35500	-3.31190
H	4.89300	12.60100	-2.29160
H	3.87260	13.11360	-3.61490
C	5.81860	12.41920	-4.23320
H	6.54110	11.65770	-3.93640
H	5.52220	12.18100	-5.25530
C	6.48240	13.79850	-4.19770
H	6.77160	14.03170	-3.17230
H	5.75720	14.55760	-4.49260
C	7.71420	13.88720	-5.10490
H	8.43570	13.12040	-4.81950
H	7.42800	13.67410	-6.13500
C	8.37430	15.26740	-5.02450
H	8.65490	15.47010	-3.99030
H	7.64910	16.03280	-5.30200
C	9.61310	15.38920	-5.91790
H	10.33610	14.61860	-5.64740
H	9.33420	15.20140	-6.95450
C	10.26800	16.77030	-5.79980
H	10.55030	16.94550	-4.76130
H	9.53990	17.54050	-6.05580
C	11.50460	16.91960	-6.69250
H	12.23110	16.14650	-6.43970

H	11.22290	16.75180	-7.73160
C	12.15790	18.30000	-6.55190
H	11.42720	19.07430	-6.78760
H	12.44910	18.45540	-5.51290
C	13.38780	18.46540	-7.45220
H	13.09830	18.30880	-8.49090
H	14.11760	17.69070	-7.21430
C	14.04240	19.84470	-7.30390
H	13.30930	20.62120	-7.52410
H	14.34460	19.98940	-6.26650
C	15.26370	20.01770	-8.21570
H	14.96540	19.86180	-9.25220
H	15.99860	19.24520	-7.98660
C	15.91590	21.39910	-8.07230
H	15.17700	22.17320	-8.28130
H	16.22960	21.54220	-7.03830
C	17.12650	21.57990	-8.99820
H	16.82450	21.41810	-10.03310
H	17.87500	20.81900	-8.77440
C	17.76500	22.96720	-8.87150
H	17.05070	23.75440	-9.11270
H	18.61150	23.06810	-9.55090
H	18.12980	23.14280	-7.85930
O	-0.42770	10.26110	1.22270
Si	0.24130	10.00420	2.75780
C	1.77470	8.90140	2.59400
H	2.23690	8.71690	3.56310
H	1.52580	7.93160	2.16430
H	2.53020	9.35850	1.95610
C	-1.04450	9.13370	3.84450
H	-1.91180	9.76470	4.02890
H	-1.40280	8.21450	3.38230
H	-0.62880	8.86880	4.81600
C	0.71990	11.70210	3.48920
C	1.78500	12.35280	2.58960
H	1.44100	12.42950	1.55870
H	2.03360	13.35830	2.93000
H	2.70770	11.77230	2.58000
C	-0.53530	12.58700	3.54390
H	-0.99880	12.68320	2.56270
H	-1.28240	12.16240	4.21160
H	-0.30540	13.59070	3.90200
C	1.28330	11.51290	4.90800
H	0.55110	11.04790	5.56900
H	2.17110	10.87990	4.90410
H	1.56420	12.46840	5.35190
O	-2.53050	8.39390	-1.06690
H	-3.22470	8.67680	-1.64580
O	-3.20300	10.81190	-2.09660
H	-3.81080	11.23850	-1.50500
C	-0.58960	14.65240	-0.20130
C	-2.44450	15.96990	-2.52500
O	-0.45750	13.47790	0.13000
O	-2.81960	17.14150	-2.48650
N	0.41500	15.39980	-0.67420
N	-1.66920	15.47990	-3.50290
H	0.20000	16.35630	-0.91370
H	-1.40160	14.50360	-3.43950
C	-1.95730	15.35100	-0.12970
H	-1.75340	16.42420	-0.11970
C	-2.68880	15.08500	1.21120
H	-1.96980	14.95670	2.02180
H	-3.26530	15.97160	1.47900
C	-4.32480	15.04470	-1.05090
H	-4.56670	16.04130	-0.67650
C	-3.63890	13.87850	1.16790
H	-3.03580	12.98020	1.02240
C	-4.69690	14.01120	0.04220

H	-5.65730	14.31410	0.45950
C	-1.02530	16.26950	-4.54460
H	-1.59900	17.17290	-4.75880
H	-1.01140	15.68610	-5.46510
C	1.75980	14.94210	-0.98980
H	1.73700	14.43800	-1.95710
H	2.10980	14.21740	-0.25270
C	-2.81350	14.99840	-1.38860
H	-2.59070	13.98110	-1.71660
C	0.40830	16.62600	-4.12230
H	0.98260	15.70940	-3.98000
H	0.37960	17.12850	-3.15510
C	1.12900	17.53230	-5.12810
H	1.15090	17.05280	-6.10680
H	0.55810	18.45370	-5.24980
C	2.56080	17.87330	-4.68630
H	2.53770	18.29280	-3.68020
H	3.15380	16.96000	-4.62740
C	3.24410	18.87380	-5.62750
H	3.25300	18.47250	-6.64080
H	2.65180	19.78890	-5.66660
C	4.67850	19.21690	-5.19780
H	5.28900	18.31380	-5.20020
H	4.67230	19.58350	-4.17090
C	5.31640	20.27340	-6.11010
H	5.29150	19.92210	-7.14160
H	4.71250	21.18130	-6.08250
C	6.76280	20.61520	-5.72350
H	7.37960	19.71820	-5.77970
H	6.79310	20.94800	-4.68560
C	7.35570	21.70490	-6.62780
H	7.28830	21.38620	-7.66820
H	6.74930	22.60780	-6.54540
C	8.81600	22.04220	-6.29470
H	9.43220	21.14940	-6.40210
H	8.88990	22.34650	-5.25040
C	9.36760	23.15770	-7.19390
H	9.25940	22.86630	-8.23900
H	8.76300	24.05600	-7.06310
C	10.83920	23.48990	-6.91010
H	10.95260	23.76930	-5.86240
H	11.45130	22.60070	-7.06170
C	11.35760	24.62670	-7.80230
H	11.21910	24.35760	-8.84980
H	10.75510	25.52010	-7.63330
C	12.83590	24.95580	-7.55310
H	12.97710	25.21490	-6.50350
H	13.44390	24.06970	-7.73740
C	13.33350	26.10890	-8.43590
H	13.18660	25.85680	-9.48670
H	12.73260	26.99920	-8.24690
C	14.81030	26.43950	-8.19810
H	14.98500	26.73100	-7.16250
H	15.13140	27.26440	-8.83440
H	15.44770	25.58360	-8.41910
C	2.69170	16.16020	-1.03260
H	2.80470	16.55220	-0.02080
H	2.21770	16.95420	-1.61030
C	4.07770	15.89300	-1.63630
H	4.57830	15.08970	-1.09460
H	3.96740	15.55620	-2.66790
C	4.93140	17.16950	-1.59160
H	5.10370	17.44340	-0.54980
H	4.36690	17.99330	-2.03010
C	6.27860	17.06510	-2.31750
H	6.85880	16.23570	-1.91160
H	6.10930	16.84430	-3.37180
C	7.07340	18.37220	-2.17900

H	7.27790	18.55510	-1.12320
H	6.45790	19.20630	-2.51780
C	8.39330	18.38400	-2.95900
H	9.01600	17.54590	-2.64460
H	8.18780	18.24040	-4.01990
C	9.15790	19.69940	-2.75070
H	9.39640	19.81320	-1.69240
H	8.51310	20.53960	-3.01050
C	10.44780	19.78580	-3.57490
H	11.09510	18.94230	-3.33210
H	10.20430	19.69580	-4.63360
C	11.20460	21.09870	-3.32820
H	10.54340	21.94350	-3.52410
H	11.48190	21.16380	-2.27540
C	12.46130	21.22970	-4.19720
H	12.17710	21.17850	-5.24840
H	13.12130	20.38110	-4.01360
C	13.22510	22.53510	-3.93380
H	12.55830	23.38530	-4.08090
H	13.53870	22.56700	-2.88960
C	14.45160	22.68880	-4.84220
H	14.13090	22.66660	-5.88390
H	15.11450	21.83360	-4.70510
C	15.23080	23.98350	-4.57270
H	14.56560	24.84050	-4.68320
H	15.57400	23.99150	-3.53740
C	16.43280	24.14730	-5.51230
H	16.08710	24.14890	-6.54610
H	17.09760	23.28870	-5.41090
C	17.22560	25.43030	-5.24210
H	16.59780	26.31460	-5.35310
H	18.05900	25.52470	-5.93870
H	17.63440	25.43500	-4.23140
O	-4.26440	13.81290	2.43990
Si	-5.19930	12.52330	3.02300
C	-4.40260	10.89600	2.48160
H	-3.32250	10.92000	2.61670
H	-4.79060	10.04550	3.04030
H	-4.59670	10.70650	1.42730
C	-6.95830	12.64790	2.32360
H	-7.39150	13.63080	2.50490
H	-6.97880	12.47310	1.24900
H	-7.62040	11.91120	2.77660
C	-5.25530	12.66790	4.92980
C	-5.86470	14.02800	5.31070
H	-6.88500	14.12670	4.93910
H	-5.89640	14.16010	6.39250
H	-5.28340	14.85250	4.89580
C	-6.11430	11.53170	5.51190
H	-7.13900	11.57440	5.14220
H	-5.71110	10.55240	5.25280
H	-6.15870	11.58820	6.59990
C	-3.82580	12.57070	5.48490
H	-3.36860	11.61270	5.23570
H	-3.19090	13.35890	5.07830
H	-3.81300	12.66670	6.57090
O	-4.86050	12.74530	-0.56940
H	-5.34670	12.90640	-1.36830
O	-5.15210	14.81250	-2.18480
H	-5.02040	15.52160	-2.79800
C	2.92900	-5.00310	-15.09270
C	0.66490	-3.13330	-14.46670
O	2.77230	-5.38860	-16.25090
O	0.96400	-2.40620	-13.52220
N	3.97970	-4.27270	-14.69710
N	0.53690	-2.66710	-15.71720
H	4.02110	-3.98650	-13.72950
H	0.29820	-3.32740	-16.44330

C	1.88260	-5.33160	-14.01860
H	2.27000	-4.96730	-13.06500
C	1.71640	-6.85880	-13.90210
H	1.30580	-7.25710	-14.83150
H	2.68700	-7.33640	-13.77840
C	-0.44230	-4.99150	-13.08550
H	-0.04490	-4.53910	-12.17560
C	0.78390	-7.23400	-12.73900
H	0.61890	-8.31290	-12.74080
C	-0.57510	-6.51580	-12.85020
H	-1.14630	-6.69590	-11.94180
C	0.84110	-1.30170	-16.12750
H	0.27390	-0.59870	-15.51560
H	0.49950	-1.17230	-17.15470
C	5.05720	-3.85140	-15.57720
H	4.65260	-3.56380	-16.54950
H	5.72230	-4.69920	-15.74760
C	0.50330	-4.65400	-14.26980
H	0.07350	-5.06930	-15.18320
C	2.35080	-1.01280	-16.03250
H	2.90060	-1.76940	-16.59390
H	2.68090	-1.10140	-14.99690
C	2.73380	0.37630	-16.55100
H	2.48730	0.44500	-17.61130
H	2.14060	1.13810	-16.04510
C	4.22530	0.66900	-16.34370
H	4.45450	0.66720	-15.27700
H	4.81550	-0.13290	-16.78850
C	4.65080	2.00500	-16.96030
H	4.42260	1.99490	-18.02700
H	4.06300	2.81480	-16.52760
C	6.14460	2.28900	-16.76330
H	6.72580	1.45000	-17.14670
H	6.36850	2.36140	-15.69850
C	6.58480	3.57140	-17.47710
H	6.35340	3.48700	-18.53980
H	6.00460	4.41520	-17.10330
C	8.08120	3.85760	-17.30740
H	8.65600	2.99740	-17.65110
H	8.31280	3.98420	-16.24960
C	8.51920	5.10100	-18.08930
H	8.28890	4.96070	-19.14620
H	7.93590	5.96110	-17.76030
C	10.01430	5.40250	-17.93310
H	10.59420	4.53580	-18.25050
H	10.24630	5.56340	-16.88010
C	10.44340	6.62580	-18.75170
H	10.22080	6.45150	-19.80510
H	9.84840	7.48860	-18.45150
C	11.93400	6.94980	-18.59460
H	12.15880	7.12680	-17.54270
H	12.52680	6.08690	-18.89800
C	12.35220	8.17020	-19.42420
H	12.14240	7.98160	-20.47780
H	11.74160	9.02690	-19.13870
C	13.83650	8.51880	-19.25520
H	14.05010	8.69260	-18.20030
H	14.44570	7.66730	-19.55840
C	14.24340	9.75240	-20.07130
H	14.05110	9.57270	-21.12960
H	13.61950	10.59950	-19.78660
C	15.71770	10.12240	-19.88190
H	15.94480	10.31000	-18.83280
H	15.96900	11.02260	-20.44280
H	16.37020	9.32160	-20.22880
C	5.83470	-2.67380	-14.97590
H	6.25250	-2.95850	-14.00920
H	5.15590	-1.84080	-14.78900

C	6.96010	-2.22250	-15.91410
H	7.63450	-3.06110	-16.09300
H	6.53490	-1.96150	-16.88420
C	7.76510	-1.03150	-15.38100
H	8.20550	-1.28670	-14.41640
H	7.09890	-0.18630	-15.20580
C	8.87010	-0.62660	-16.36520
H	9.52720	-1.47990	-16.53900
H	8.42230	-0.38460	-17.33000
C	9.70730	0.56390	-15.88500
H	10.16940	0.32320	-14.92710
H	9.05670	1.42120	-15.71090
C	10.79430	0.93640	-16.90180
H	11.44180	0.07480	-17.07010
H	10.32930	1.16240	-17.86220
C	11.64610	2.13090	-16.45780
H	12.12060	1.90480	-15.50230
H	11.00140	2.99350	-16.28820
C	12.72200	2.48600	-17.49260
H	13.37050	1.62350	-17.65140
H	12.24790	2.69470	-18.45250
C	13.57180	3.69120	-17.07280
H	12.92310	4.55270	-16.91200
H	14.05120	3.48180	-16.11590
C	14.64250	4.04070	-18.11530
H	14.16500	4.23260	-19.07700
H	15.29880	3.18220	-18.26330
C	15.48010	5.25990	-17.71000
H	14.82160	6.11570	-17.55940
H	15.95970	5.06650	-16.74990
C	16.54970	5.61310	-18.75270
H	16.07290	5.79200	-19.71720
H	17.21670	4.76130	-18.89170
C	17.37080	6.84570	-18.35230
H	16.70050	7.69370	-18.20980
H	17.84880	6.66340	-17.38920
C	18.44060	7.20930	-19.39110
H	17.96830	7.38280	-20.35860
H	19.11980	6.36700	-19.52710
C	19.24850	8.45010	-18.99370
H	18.60480	9.32300	-18.88320
H	19.99770	8.68440	-19.75020
H	19.76870	8.29580	-18.04800
O	1.39640	-6.88220	-11.51200
Si	2.34490	-7.91780	-10.56530
C	3.55440	-8.87600	-11.66750
H	4.16470	-9.56290	-11.08230
H	3.03060	-9.46710	-12.41800
H	4.23540	-8.20750	-12.19280
C	1.20320	-9.14440	-9.68070
H	0.54990	-8.64640	-8.96720
H	0.56790	-9.67800	-10.38680
H	1.77370	-9.88950	-9.12740
C	3.30090	-6.83790	-9.31440
C	4.25520	-5.90680	-10.08260
H	3.71740	-5.30440	-10.81490
H	4.77070	-5.22190	-9.40900
H	5.01620	-6.47080	-10.62220
C	2.29030	-6.00040	-8.51650
H	1.67810	-5.38700	-9.17700
H	1.61480	-6.63750	-7.94940
H	2.78700	-5.33380	-7.81120
C	4.10370	-7.73870	-8.36070
H	3.45030	-8.40980	-7.80220
H	4.82140	-8.35460	-8.90350
H	4.66210	-7.14680	-7.63490
O	-1.30760	-7.08920	-13.91500
H	-2.02390	-6.49860	-14.10180

O	-1.74790	-4.47020	-13.28190
H	-2.15610	-4.38660	-12.42920
C	1.97380	-2.19620	-10.36890
C	-0.09270	0.16680	-11.13670
O	1.98010	-3.31580	-10.87500
O	-0.42030	1.15960	-10.48810
N	2.99270	-1.33460	-10.46340
N	0.52720	0.25120	-12.32250
H	2.89110	-0.45670	-9.96700
H	0.74910	-0.61910	-12.79460
C	0.74490	-1.66890	-9.61050
H	1.06830	-0.77670	-9.07520
C	0.27930	-2.64300	-8.49880
H	1.11790	-3.23270	-8.12630
H	-0.05490	-2.06180	-7.64110
C	-1.76420	-1.34060	-9.90100
H	-1.74030	-0.71070	-9.01000
C	-0.86570	-3.57510	-8.91870
H	-0.48170	-4.26060	-9.67640
C	-2.08800	-2.79100	-9.46190
H	-2.86610	-2.72830	-8.70120
C	0.94250	1.48820	-12.97440
H	0.29300	2.31600	-12.68460
H	0.81150	1.35950	-14.04830
C	4.17420	-1.45440	-11.30010
H	3.86570	-1.43870	-12.34680
H	4.67900	-2.40440	-11.11820
C	-0.38530	-1.25100	-10.60120
H	-0.41470	-1.94040	-11.44530
C	2.41050	1.82440	-12.66080
H	3.04270	0.99110	-12.97030
H	2.53820	1.93150	-11.58250
C	2.88110	3.10910	-13.36120
H	2.69830	3.01860	-14.43230
H	2.27470	3.94800	-13.01760
C	4.36880	3.42490	-13.13050
H	4.55860	3.53620	-12.06250
H	4.97390	2.58230	-13.46740
C	4.81340	4.69870	-13.86870
H	4.60390	4.58710	-14.93280
H	4.21190	5.54170	-13.52790
C	6.30290	5.03040	-13.68290
H	6.90680	4.18280	-14.00810
H	6.51510	5.17590	-12.62360
C	6.72230	6.28450	-14.46730
H	6.50150	6.13840	-15.52470
H	6.11660	7.13150	-14.14510
C	8.20980	6.63440	-14.30810
H	8.81800	5.78150	-14.60960
H	8.42900	6.81710	-13.25620
C	8.61510	7.86380	-15.13600
H	8.39580	7.68040	-16.18770
H	8.00240	8.71580	-14.84170
C	10.09960	8.22780	-14.98590
H	10.71400	7.37140	-15.26370
H	10.31570	8.43930	-13.93890
C	10.49960	9.43730	-15.84350
H	10.29030	9.22490	-16.89180
H	9.87790	10.29140	-15.57580
C	11.97970	9.81490	-15.68840
H	12.18650	10.04120	-14.64250
H	12.60320	8.96000	-15.95050
C	12.37560	11.01700	-16.55700
H	12.18110	10.78940	-17.60480
H	11.74130	11.86730	-16.30720
C	13.84900	11.41230	-16.38730
H	14.04170	11.64440	-15.34010
H	14.48570	10.56380	-16.63880

C	14.23880	12.61700	-17.25450
H	14.06650	12.38680	-18.30560
H	13.59170	13.46110	-17.01880
C	15.70040	13.03380	-17.06440
H	15.89950	13.30590	-16.02860
H	15.94260	13.89640	-17.68530
H	16.38030	12.22650	-17.33630
C	5.09960	-0.26750	-10.98750
H	5.50050	-0.38390	-9.97940
H	4.50950	0.64880	-10.96870
C	6.25010	-0.06110	-11.98000
H	6.87370	-0.95470	-12.02550
H	5.83950	0.08640	-12.97980
C	7.10040	1.15780	-11.58400
H	7.57430	0.96810	-10.62000
H	6.44960	2.02000	-11.43580
C	8.17160	1.52400	-12.61830
H	8.83100	0.67060	-12.77930
H	7.69280	1.73270	-13.57570
C	9.00100	2.74230	-12.18320
H	9.49700	2.52440	-11.23650
H	8.33740	3.58590	-11.99270
C	10.04880	3.14870	-13.22740
H	10.71230	2.30500	-13.41940
H	9.55060	3.37170	-14.17130
C	10.88280	4.36220	-12.79200
H	11.38290	4.14170	-11.84810
H	10.22260	5.20740	-12.59920
C	11.92670	4.75880	-13.84470
H	12.58850	3.91260	-14.03160
H	11.42480	4.97260	-14.78870
C	12.76500	5.97490	-13.42840
H	12.10700	6.82510	-13.25250
H	13.26240	5.76880	-12.47990
C	13.81180	6.34930	-14.48640
H	13.31290	6.54580	-15.43570
H	14.47350	5.49920	-14.65510
C	14.64920	7.57260	-14.09090
H	13.99040	8.42650	-13.93920
H	15.13920	7.38660	-13.13440
C	15.70430	7.92430	-15.14810
H	15.21260	8.10410	-16.10450
H	16.36590	7.07010	-15.29520
C	16.54020	9.15360	-14.76910
H	15.88090	10.01120	-14.64280
H	17.01950	8.98660	-13.80370
C	17.60700	9.48240	-15.82150
H	17.12820	9.64960	-16.78630
H	18.27100	8.62710	-15.94750
C	18.44130	10.71330	-15.45790
H	17.81350	11.59940	-15.37770
H	19.19600	10.91000	-16.21960
H	18.95460	10.57720	-14.50580
O	-1.21520	-4.31160	-7.75660
Si	-2.26030	-5.64500	-7.68970
C	-1.92180	-6.75700	-9.18030
H	-0.85360	-6.86050	-9.36360
H	-2.33850	-7.75480	-9.05080
H	-2.36790	-6.33310	-10.07820
C	-4.06200	-5.05090	-7.72380
H	-4.24540	-4.28930	-6.96680
H	-4.32830	-4.62450	-8.68970
H	-4.75540	-5.86980	-7.53740
C	-1.92240	-6.55600	-6.04090
C	-2.19730	-5.59260	-4.87330
H	-3.23700	-5.26430	-4.86240
H	-1.99360	-6.06510	-3.91200
H	-1.57290	-4.70060	-4.94040

C	-2.84090	-7.78530	-5.92870
H	-3.89350	-7.50210	-5.94940
H	-2.67230	-8.48660	-6.74630
H	-2.66740	-8.32390	-4.99670
C	-0.45320	-7.00330	-6.00110
H	-0.22510	-7.68740	-6.81850
H	0.22040	-6.14940	-6.08240
H	-0.21870	-7.51780	-5.06880
O	-2.60970	-3.49340	-10.57530
H	-3.18800	-2.88210	-11.01370
O	-2.83260	-0.87140	-10.71540
H	-2.69970	0.05360	-10.86770
C	3.01600	1.75310	-7.79720
C	0.65370	3.59000	-7.89010
O	2.91830	1.05360	-8.80350
O	0.89290	4.57260	-7.19010
N	4.03320	2.59910	-7.59510
N	0.56660	3.66260	-9.22600
H	4.02960	3.14930	-6.74830
H	0.34240	2.81140	-9.72890
C	1.92110	1.71650	-6.71970
H	2.22230	2.38940	-5.91430
C	1.81930	0.29750	-6.12830
H	1.53630	-0.40910	-6.90590
H	2.79470	-0.03230	-5.77380
C	-0.50500	2.15580	-6.11100
H	-0.23380	2.89540	-5.35960
C	0.78650	0.21930	-4.99270
H	0.66530	-0.82340	-4.69310
C	-0.58630	0.77600	-5.41930
H	-1.22410	0.85120	-4.54120
C	0.85300	4.85350	-10.01600
H	0.24930	5.68520	-9.65050
H	0.54030	4.66380	-11.04320
C	5.11640	2.81820	-8.54130
H	4.72770	2.82180	-9.56130
H	5.82260	1.98970	-8.47150
C	0.53810	2.18830	-7.25760
H	0.21350	1.48750	-8.02640
C	2.35190	5.20730	-9.97650
H	2.93420	4.36050	-10.34080
H	2.66500	5.37040	-8.94490
C	2.70140	6.45790	-10.79110
H	2.48250	6.27940	-11.84430
H	2.06580	7.28660	-10.47820
C	4.17400	6.85720	-10.62150
H	4.37470	7.06080	-9.56870
H	4.81060	6.01730	-10.90100
C	4.55780	8.08430	-11.45690
H	4.37320	7.87560	-12.51120
H	3.91320	8.92270	-11.19200
C	6.02620	8.48600	-11.26250
H	6.66700	7.63510	-11.49440
H	6.20120	8.73030	-10.21400
C	6.43030	9.67670	-12.14090
H	6.25550	9.42910	-13.18820
H	5.78770	10.52800	-11.91680
C	7.89910	10.07750	-11.95040
H	8.53810	9.21920	-12.15890
H	8.07060	10.34680	-10.90780
C	8.30910	11.24560	-12.85640
H	8.13930	10.97350	-13.89840
H	7.66550	12.10200	-12.65670
C	9.77680	11.65180	-12.66900
H	10.41810	10.79270	-12.86560
H	9.94560	11.93370	-11.62950
C	10.18510	12.81100	-13.58720
H	10.02040	12.52680	-14.62660

H	9.53680	13.66660	-13.39960
C	11.64980	13.22710	-13.39820
H	11.81380	13.51580	-12.35990
H	12.29700	12.37090	-13.58740
C	12.05400	14.38420	-14.32120
H	11.89500	14.09370	-15.35960
H	11.39900	15.23620	-14.14090
C	13.51480	14.81190	-14.12760
H	13.67030	15.10730	-13.09000
H	14.16940	13.95920	-14.30650
C	13.91710	15.96720	-15.05420
H	13.78180	15.67150	-16.09470
H	13.25170	16.81420	-14.89030
C	15.36640	16.41530	-14.84300
H	15.53570	16.72740	-13.81300
H	15.61180	17.25770	-15.48990
H	16.06440	15.60950	-15.06780
C	5.81820	4.15010	-8.25390
H	6.21030	4.14650	-7.23590
H	5.09240	4.96280	-8.30250
C	6.95880	4.42390	-9.24140
H	7.68630	3.61260	-9.18940
H	6.56710	4.41890	-10.25900
C	7.66110	5.75990	-8.96950
H	8.04410	5.76880	-7.94820
H	6.93640	6.57230	-9.03300
C	8.81240	6.01920	-9.94900
H	9.53340	5.20300	-9.88640
H	8.42860	6.01130	-10.96930
C	9.52440	7.35060	-9.67880
H	9.90260	7.35870	-8.65590
H	8.80560	8.16760	-9.74790
C	10.68280	7.60080	-10.65290
H	11.39930	6.78130	-10.58340
H	10.30380	7.59130	-11.67460
C	11.40070	8.93020	-10.38600
H	11.77930	8.93870	-9.36340
H	10.68450	9.74910	-10.45710
C	12.56010	9.17680	-11.36030
H	13.27640	8.35750	-11.28700
H	12.18120	9.16400	-12.38200
C	13.27800	10.50760	-11.10010
H	12.56100	11.32550	-11.17470
H	13.65740	10.52060	-10.07790
C	14.43680	10.75240	-12.07600
H	14.05790	10.73220	-13.09740
H	15.15620	9.93640	-11.99730
C	15.14970	12.08780	-11.82540
H	14.42920	12.90180	-11.90670
H	15.52800	12.10950	-10.80290
C	16.30870	12.33200	-12.80170
H	15.93210	12.30060	-13.82360
H	17.03350	11.52180	-12.71440
C	17.01160	13.67470	-12.56100
H	16.28460	14.48220	-12.64920
H	17.38820	13.70590	-11.53820
C	18.17050	13.92310	-13.53670
H	17.80090	13.89080	-14.56160
H	18.90250	13.11970	-13.44710
C	18.86680	15.26760	-13.30040
H	18.17270	16.09930	-13.42240
H	19.68290	15.41140	-14.00880
H	19.28630	15.32400	-12.29570
O	1.26110	0.94440	-3.87350
Si	2.10380	0.26310	-2.57140
C	3.51140	-0.82570	-3.22580
H	4.07410	-1.27930	-2.41060
H	3.13410	-1.63600	-3.84870

H	4.21670	-0.25160	-3.82540
C	0.90570	-0.80400	-1.56300
H	0.13370	-0.20340	-1.08620
H	0.40500	-1.54280	-2.18780
H	1.42500	-1.34420	-0.77220
C	2.80470	1.69140	-1.51670
C	3.81000	2.49310	-2.36170
H	3.35500	2.85350	-3.28420
H	4.18330	3.36140	-1.81820
H	4.67130	1.88580	-2.64050
C	1.64170	2.59880	-1.08890
H	1.09090	2.96690	-1.95380
H	0.93420	2.05910	-0.46240
H	1.99100	3.46350	-0.52440
C	3.50670	1.12010	-0.27300
H	2.81660	0.54440	0.34470
H	4.33060	0.46080	-0.54750
H	3.91670	1.91590	0.34950
O	-1.20840	-0.14090	-6.29610
H	-1.93420	0.31970	-6.69390
O	-1.80580	2.48450	-6.57490
H	-2.31960	2.75980	-5.82520
C	1.30250	5.60330	-4.08180
C	-0.76600	7.49950	-5.70180
O	1.43860	4.38860	-4.20050
O	-1.22450	8.59670	-5.38540
N	2.27200	6.48320	-4.35550
N	-0.00790	7.32140	-6.79350
H	2.06340	7.45870	-4.17600
H	0.32400	6.38100	-6.98110
C	-0.03760	6.21720	-3.64580
H	0.17040	7.24390	-3.35090
C	-0.59680	5.55970	-2.35940
H	0.21360	5.17900	-1.73680
H	-1.07530	6.32740	-1.75380
C	-2.50090	6.23720	-4.29380
H	-2.62810	7.09200	-3.62670
C	-1.62640	4.45160	-2.61420
H	-1.11490	3.61530	-3.09420
C	-2.80680	4.94900	-3.48730
H	-3.67350	5.16390	-2.86220
C	0.42190	8.37070	-7.71180
H	-0.27740	9.20850	-7.70060
H	0.39460	7.96320	-8.72210
C	3.54050	6.20940	-5.00970
H	3.34320	5.92200	-6.04390
H	4.05060	5.37410	-4.52740
C	-1.04800	6.25300	-4.83430
H	-0.92520	5.36470	-5.45600
C	1.84370	8.85620	-7.38240
H	2.52840	8.00750	-7.41160
H	1.86740	9.24370	-6.36280
C	2.33630	9.94220	-8.35300
H	2.26100	9.57030	-9.37520
H	1.67200	10.80500	-8.29140
C	3.78360	10.39040	-8.08590
H	3.86650	10.76180	-7.06380
H	4.44850	9.52870	-8.15860
C	4.25010	11.47920	-9.06740
H	4.14800	11.11130	-10.08850
H	3.58800	12.34170	-8.98800
C	5.70210	11.93060	-8.83700
H	6.36780	11.07200	-8.93060
H	5.80980	12.29800	-7.81610
C	6.14120	13.02710	-9.82250
H	6.02780	12.66300	-10.84350
H	5.47280	13.88310	-9.73070
C	7.59100	13.49090	-9.60830

H	8.26310	12.63850	-9.71090
H	7.70620	13.85640	-8.58790
C	8.01310	14.59430	-10.59240
H	7.89530	14.23460	-11.61430
H	7.34030	15.44580	-10.49180
C	9.46100	15.06380	-10.38220
H	10.13650	14.21470	-10.48930
H	9.57810	15.42610	-9.36100
C	9.87660	16.17210	-11.36200
H	9.75750	15.81690	-12.38520
H	9.20270	17.02210	-11.25560
C	11.32400	16.64060	-11.14970
H	11.44090	17.00060	-10.12780
H	11.99960	15.79180	-11.25770
C	11.73970	17.75050	-12.12630
H	11.62020	17.39800	-13.15040
H	11.06670	18.60090	-12.01760
C	13.18740	18.21520	-11.91120
H	13.30310	18.57610	-10.88950
H	13.86170	17.36490	-12.01540
C	13.60630	19.32220	-12.88890
H	13.49990	18.96940	-13.91460
H	12.93330	20.17370	-12.78860
C	15.04810	19.79060	-12.66610
H	15.18320	20.18460	-11.65950
H	15.31280	20.58090	-13.36860
H	15.75570	18.97290	-12.80280
C	4.39770	7.48310	-4.95440
H	4.71220	7.65490	-3.92390
H	3.78040	8.34060	-5.22320
C	5.62470	7.47690	-5.87650
H	6.27240	6.63240	-5.63780
H	5.30010	7.34090	-6.90920
C	6.40580	8.79460	-5.74730
H	6.79940	8.87840	-4.73340
H	5.71970	9.63270	-5.87320
C	7.55290	8.94640	-6.75510
H	8.25020	8.11430	-6.64960
H	7.15650	8.89400	-7.76990
C	8.29760	10.27600	-6.55810
H	8.70930	10.31130	-5.54870
H	7.58830	11.10120	-6.62460
C	9.42520	10.50060	-7.57400
H	10.13760	9.67710	-7.51440
H	9.01460	10.48350	-8.58400
C	10.15730	11.83000	-7.33750
H	10.56380	11.84450	-6.32550
H	9.44310	12.65130	-7.39080
C	11.28960	12.07640	-8.34340
H	12.00620	11.25650	-8.28800
H	10.88460	12.06650	-9.35550
C	12.01640	13.40600	-8.09540
H	11.30230	14.22610	-8.15930
H	12.41090	13.42080	-7.07860
C	13.15920	13.65020	-9.08960
H	12.76480	13.63420	-10.10580
H	13.87590	12.83120	-9.02220
C	13.88360	14.98120	-8.84490
H	13.17140	15.80110	-8.92720
H	14.26590	15.00580	-7.82370
C	15.03780	15.21230	-9.82860
H	14.65370	15.18710	-10.84850
H	15.75100	14.39180	-9.74480
C	15.76500	16.54280	-9.59210
H	15.05740	17.36470	-9.69360
H	16.13660	16.57880	-8.56720
C	16.93040	16.75410	-10.56670
H	16.55860	16.72110	-11.59070

H	17.64150	15.93390	-10.46670
C	17.66260	18.08020	-10.34270
H	16.99230	18.92670	-10.48450
H	18.48870	18.19090	-11.04540
H	18.07200	18.14080	-9.33400
O	-2.08150	4.04830	-1.33160
Si	-3.07210	2.71690	-0.98330
C	-2.51550	1.24790	-2.03470
H	-1.43000	1.17060	-2.06290
H	-2.90840	0.30410	-1.65920
H	-2.86410	1.35780	-3.06020
C	-4.88030	3.14140	-1.37060
H	-5.18210	4.07350	-0.89430
H	-5.04920	3.24980	-2.44090
H	-5.55620	2.36240	-1.02070
C	-2.89010	2.34740	0.88520
C	-3.34550	3.58040	1.68470
H	-4.39360	3.81440	1.49590
H	-3.23410	3.42180	2.75770
H	-2.76040	4.46240	1.42020
C	-3.75670	1.13320	1.26230
H	-4.81120	1.31290	1.05170
H	-3.45770	0.24160	0.71080
H	-3.67190	0.90250	2.32470
C	-1.41590	2.04880	1.19930
H	-1.05770	1.18490	0.63890
H	-0.78060	2.89790	0.94480
H	-1.26950	1.83620	2.25860
O	-3.15850	3.91930	-4.39210
H	-3.72630	4.32680	-5.03390
O	-3.47610	6.36370	-5.32220
H	-3.37540	7.21790	-5.71790

4-alpha octamer

C	0.62432378	17.86320082	4.42385394
C	-1.58307622	19.87190082	4.30835394
O	0.65072378	17.01180082	3.53455394
O	-1.22507622	20.83300082	4.99005394
N	1.55682378	18.81430082	4.55115394
N	-1.69167622	19.95240082	2.97665394
H	1.42342378	19.48170082	5.29805394
H	-2.00407622	19.10900082	2.48945394
C	-0.50017622	17.87430082	5.46905394
H	-0.15037622	18.48340082	6.30545394
C	-0.75137622	16.45230082	6.00605394
H	-1.18107622	15.82580082	5.22265394
H	0.18552378	15.97570082	6.29265394
C	-2.84887622	18.57160082	6.12645394
H	-2.50797622	19.31020082	6.84915394
C	-1.71367622	16.49210082	7.19825394
H	-1.95847622	15.47760082	7.50675394
C	-3.02377622	17.22250082	6.85885394
H	-3.58017622	17.38540082	7.77805394
C	-1.37097622	21.11890082	2.16425394
H	-1.96357622	21.96760082	2.50695394
H	-1.68017622	20.91160082	1.13935394
C	2.69782378	18.98980082	3.66655394
H	2.35442378	19.00290082	2.63125394
H	3.36422378	18.13200082	3.76995394
C	-1.83097622	18.49490082	4.95605394
H	-2.24037622	17.82610082	4.20265394
C	0.13392378	21.44360082	2.20715394
H	0.69832378	20.56300082	1.90215394
H	0.43192378	21.65490082	3.23415394
C	0.53992378	22.63700082	1.33395394

H	0.35882378	22.40140082	0.28505394
H	-0.08627622	23.49740082	1.56945394
C	2.01562378	23.00540082	1.54425394
H	2.17552378	23.27300082	2.58995394
H	2.63442378	22.12850082	1.35365394
C	2.48802378	24.15770082	0.65005394
H	2.35522378	23.88360082	-0.39674606
H	1.86272378	25.03470082	0.81825394
C	3.95892378	24.51510082	0.90525394
H	4.57412378	23.62500082	0.77245394
H	4.08422378	24.82510082	1.94375394
C	4.46922378	25.62200082	-0.02524606
H	4.35422378	25.30540082	-1.06214606
H	3.84982378	26.51130082	0.09195394
C	5.93872378	25.97790082	0.23865394
H	6.55152378	25.08190082	0.13895394
H	6.05312378	26.31840082	1.26865394
C	6.45922378	27.05560082	-0.72054606
H	6.35462378	26.70690082	-1.74824606
H	5.83682378	27.94660082	-0.63724606
C	7.92472378	27.42590082	-0.45624606
H	8.54432378	26.53240082	-0.53334606
H	8.03082378	27.78970082	0.56635394
C	8.44022378	28.48750082	-1.43624606
H	8.34402378	28.11610082	-2.45674606
H	7.80862378	29.37380082	-1.37424606
C	9.90002378	28.87990082	-1.17364606
H	9.99752378	29.26080082	-0.15664606
H	10.53112378	27.99330082	-1.23444606
C	10.40502378	29.93370082	-2.16784606
H	10.31922378	29.54570082	-3.18314606
H	9.76072378	30.81190082	-2.12154606
C	11.85722378	30.35100082	-1.90294606
H	11.94292378	30.74140082	-0.88854606
H	12.50212378	29.47350082	-1.95214606
C	12.35222378	31.40550082	-2.90174606
H	12.28852378	31.01090082	-3.91634606
H	11.69512378	32.27460082	-2.87054606
C	13.79112378	31.85080082	-2.62384606
H	13.88852378	32.26720082	-1.62154606
H	14.10572378	32.61720082	-3.33244606
H	14.48532378	31.01520082	-2.70954606
C	3.44352378	20.29370082	3.99205394
H	3.77862378	20.27490082	5.03015394
H	2.75882378	21.13810082	3.90405394
C	4.65192378	20.52010082	3.07195394
H	5.34192378	19.68080082	3.17025394
H	4.32032378	20.52240082	2.03335394
C	5.39752378	21.83030082	3.36775394
H	5.72402378	21.83850082	4.40875394
H	4.71452378	22.67280082	3.25315394
C	6.61302378	22.02260082	2.44825394
H	7.29382378	21.17860082	2.56735394
H	6.28602378	22.00680082	1.40855394
C	7.37332378	23.32830082	2.71915394
H	7.69862378	23.35060082	3.75995394
H	6.69872378	24.17500082	2.58825394
C	8.59262378	23.49230082	1.79915394
H	9.26702378	22.64590082	1.93585394
H	8.26752378	23.46010082	0.75945394
C	9.35982378	24.79780082	2.04935394
H	9.68652378	24.83300082	3.08915394
H	8.68772378	25.64460082	1.90695394
C	10.57942378	24.94940082	1.12825394
H	11.25672378	24.10790082	1.27955394
H	10.25562378	24.90050082	0.08895394
C	11.33862378	26.26270082	1.36105394
H	10.66062378	27.10270082	1.20665394

H	11.66362378	26.31420082	2.40075394
C	12.55792378	26.41310082	0.44015394
H	12.23662378	26.34460082	-0.59864606
H	13.24492378	25.58230082	0.60615394
C	13.29892378	27.73970082	0.65485394
H	12.60992378	28.56800082	0.48705394
H	13.62142378	27.81100082	1.69425394
C	14.51702378	27.89430082	-0.26644606
H	14.19802378	27.80470082	-1.30444606
H	15.21602378	27.07640082	-0.08684606
C	15.23702378	29.23530082	-0.07054606
H	14.53552378	30.05060082	-0.25004606
H	15.55782378	29.32520082	0.96785394
C	16.45262378	29.39530082	-0.99384606
H	16.13902378	29.29970082	-2.03354606
H	17.16092378	28.58670082	-0.80954606
C	17.16412378	30.74000082	-0.80824606
H	16.49612378	31.57490082	-1.02104606
H	18.01942378	30.82240082	-1.47934606
H	17.53212378	30.85560082	0.21155394
O	-1.09287622	17.15810082	8.28025394
Si	-0.29477622	16.40670082	9.56375394
C	1.21102378	15.47970082	8.90765394
H	1.80102378	15.05900082	9.72035394
H	0.92862378	14.66020082	8.25125394
H	1.86152378	16.14310082	8.33975394
C	-1.48897622	15.22310082	10.41955394
H	-2.36057622	15.75060082	10.80315394
H	-1.84777622	14.44940082	9.74415394
H	-1.01507622	14.72400082	11.26315394
C	0.26932378	17.77200082	10.76865394
C	1.34402378	18.63480082	10.08545394
H	0.98202378	19.03490082	9.13775394
H	1.63382378	19.48040082	10.70925394
H	2.24422378	18.05860082	9.87135394
C	-0.94917622	18.63940082	11.11425394
H	-1.37487622	19.08390082	10.21455394
H	-1.73417622	18.04720082	11.58095394
H	-0.69207622	19.45060082	11.79525394
C	0.84312378	17.13600082	12.04615394
H	0.09572378	16.53110082	12.56065394
H	1.69222378	16.48930082	11.82285394
H	1.18512378	17.89730082	12.74775394
O	-3.83027622	16.43080082	6.01925394
H	-4.36647622	17.08770082	5.57865394
O	-4.15227622	18.93860082	5.67435394
H	-4.61837622	19.30810082	6.42885394
C	-0.53748223	22.70024580	8.54162375
C	-2.44158223	24.59074580	6.81342375
O	-0.45388223	21.49624580	8.32092375
O	-2.87338223	25.71664580	7.06312375
N	0.49291777	23.53664580	8.40242375
N	-1.64068223	24.35414580	5.76822375
H	0.33581777	24.50964580	8.66962375
H	-1.34448223	23.38534580	5.61772375
C	-1.87548223	23.34154580	8.93242375
H	-1.66658223	24.36524580	9.23542375
C	-2.49278223	22.68084580	10.19052375
H	-1.71378223	22.24934580	10.82002375
H	-2.95318223	23.45444580	10.80452375
C	-4.30258223	23.45524580	8.17342375
H	-4.42838223	24.34104580	8.79992375
C	-3.55818223	21.62194580	9.88302375
H	-3.07958223	20.77724580	9.38482375
C	-4.69158223	22.20554580	9.00452375
H	-5.54398223	22.49154580	9.62072375
C	-1.16368223	25.35434580	4.82152375
H	-1.84538223	26.20584580	4.77922375

H	-1.17478223	24.90804580	3.82832375
C	1.75751777	23.22794580	7.75872375
H	1.55771777	22.91464580	6.73222375
H	2.24721777	22.39504580	8.26622375
C	-2.82868223	23.39034580	7.70052375
H	-2.71928223	22.48074580	7.10902375
C	0.25801777	25.82424580	5.16732375
H	0.92581777	24.96214580	5.19642375
H	0.26201777	26.25884580	6.16832375
C	0.79021777	26.85174580	4.15632375
H	0.73741777	26.42824580	3.15312375
H	0.13501777	27.72354580	4.15352375
C	2.23411777	27.29794580	4.43702375
H	2.29581777	27.72504580	5.43852375
H	2.89191777	26.42774580	4.42882375
C	2.73121777	28.32554580	3.40732375
H	2.65751777	27.89924580	2.40682375
H	2.07071777	29.19294580	3.41552375
C	4.17631777	28.78554580	3.65262375
H	4.84131777	27.92134580	3.63142375
H	4.25651777	29.21534580	4.65122375
C	4.64441777	29.81714580	2.61352375
H	4.56231777	29.38924580	1.61462375
H	3.97491777	30.67724580	2.63032375
C	6.08671777	30.29194580	2.84332375
H	6.75861777	29.43344580	2.82442375
H	6.16921777	30.72944580	3.83822375
C	6.54321777	31.32014580	1.79682375
H	6.46221777	30.88594580	0.80052375
H	5.86881777	32.17654580	1.81102375
C	7.98301777	31.80174580	2.02542375
H	8.65771777	30.94524580	2.01552375
H	8.06141777	32.24714580	3.01692375
C	8.44101777	32.82274580	0.97392375
H	8.36661777	32.38104580	-0.01967625
H	7.76461777	33.67784580	0.97902375
C	9.87831777	33.30744580	1.20982375
H	9.94861777	33.76044580	2.19862375
H	10.55461777	32.45204580	1.21172375
C	10.34211777	34.32104580	0.15472375
H	10.27721777	33.87114580	-0.83597625
H	9.66341777	35.17414580	0.14772375
C	11.77571777	34.81114580	0.40032375
H	11.83541777	35.27184580	1.38632375
H	12.45461777	33.95784580	0.41612375
C	12.24411777	35.81784580	-0.65897625
H	12.20021777	35.36104580	-1.64787625
H	11.56091777	36.66694580	-0.68467625
C	13.66701777	36.32344580	-0.40047625
H	13.73751777	36.82234580	0.56542375
H	13.96991777	37.03984580	-1.16437625
H	14.38611777	35.50414580	-0.40877625
C	2.63691777	24.48674580	7.78152375
H	2.94341777	24.68694580	8.80942375
H	2.03641777	25.34384580	7.47702375
C	3.87241777	24.42744580	6.87322375
H	4.50431777	23.58254580	7.15022375
H	3.55611777	24.25794580	5.84282375
C	4.67131777	25.73734580	6.96322375
H	5.05451777	25.85344580	7.97822375
H	3.99911777	26.57944580	6.79732375
C	5.83181777	25.83404580	5.96472375
H	6.51801777	24.99934580	6.11332375
H	5.44791777	25.74484580	4.94742375
C	6.58811777	27.16254580	6.11762375
H	6.98441777	27.23704580	7.13132375
H	5.88931777	27.99144580	6.00422375
C	7.73381777	27.33064580	5.11172375

H	8.43701777	26.50434580	5.22142375
H	7.34061777	27.27194580	4.09612375
C	8.47431777	28.66264580	5.30082375
H	8.85961777	28.72234580	6.31962375
H	7.77011777	29.48694580	5.19252375
C	9.62961777	28.84924580	4.30882375
H	10.33711777	28.02724580	4.42162375
H	9.24741777	28.79224580	3.28902375
C	10.36241777	30.18354580	4.50702375
H	9.65861777	31.00514580	4.38042375
H	10.72951777	30.24914580	5.53222375
C	11.53381777	30.36444580	3.53312375
H	11.16851777	30.29504580	2.50792375
H	12.24161777	29.54574580	3.66632375
C	12.26041777	31.70244580	3.72592375
H	11.55831777	32.52084580	3.57212375
H	12.60831777	31.78434580	4.75652375
C	13.44891777	31.86784580	2.77052375
H	13.10111777	31.78154580	1.74062375
H	14.15391777	31.05114580	2.92932375
C	14.17401777	33.20794580	2.95172375
H	13.47541777	34.02424580	2.77102375
H	14.50511777	33.30814580	3.98622375
C	15.37761777	33.35184580	2.01182375
H	15.04711777	33.25124580	0.97792375
H	16.08081777	32.53854580	2.19332375
C	16.10441777	34.68964580	2.17812375
H	15.44381777	35.52564580	1.95202375
H	16.95981777	34.75344580	1.50512375
H	16.47161777	34.81814580	3.19672375
O	-4.05328223	21.20064580	11.14482375
Si	-5.20628223	19.99654580	11.44372375
C	-4.78438223	18.45874580	10.44862375
H	-3.71838223	18.24344580	10.48942375
H	-5.31698223	17.58204580	10.81252375
H	-5.06588223	18.59614580	9.40612375
C	-6.93748223	20.61524580	11.01272375
H	-7.13618223	21.58494580	11.46642375
H	-7.06908223	20.71624580	9.93652375
H	-7.70348223	19.92554580	11.36232375
C	-5.13418223	19.62244580	13.31752375
C	-5.46288223	20.90964580	14.09292375
H	-6.46888223	21.26524580	13.86852375
H	-5.40188223	20.75434580	15.17022375
H	-4.76998223	21.71224580	13.83482375
C	-6.15298223	18.52524580	13.67272375
H	-7.17088223	18.82894580	13.42752375
H	-5.94818223	17.59954580	13.13452375
H	-6.13048223	18.29424580	14.73802375
C	-3.71758223	19.15124580	13.68152375
H	-3.44678223	18.24694580	13.13562375
H	-2.97818223	19.91654580	13.44112375
H	-3.62908223	18.93244580	14.74572375
O	-5.10708223	21.24854580	8.05182375
H	-5.47348223	21.83244580	7.38042375
O	-5.23188223	23.53744580	7.08692375
H	-5.13098223	24.39374580	6.68892375
C	1.06970000	10.36510000	-2.42360000
C	-1.13770000	12.37380000	-2.53910000
O	1.09610000	9.51370000	-3.31290000
O	-0.77970000	13.33490000	-1.85740000
N	2.00220000	11.31620000	-2.29630000
N	-1.24630000	12.45430000	-3.87080000
H	1.86880000	11.98360000	-1.54940000
H	-1.55870000	11.61090000	-4.35800000
C	-0.05480000	10.37620000	-1.37840000
H	0.29500000	10.98530000	-0.54200000
C	-0.30600000	8.95420000	-0.84140000

H	-0.73570000	8.32770000	-1.62480000
H	0.63090000	8.47760000	-0.55480000
C	-2.40350000	11.07350000	-0.72100000
H	-2.06260000	11.81210000	0.00170000
C	-1.26830000	8.99400000	0.35080000
H	-1.51310000	7.97950000	0.65930000
C	-2.57840000	9.72440000	0.01140000
H	-3.13480000	9.88730000	0.93060000
C	-0.92560000	13.62080000	-4.68320000
H	-1.51820000	14.46950000	-4.34050000
H	-1.23480000	13.41350000	-5.70810000
C	3.14320000	11.49170000	-3.18090000
H	2.79980000	11.50480000	-4.21620000
H	3.80960000	10.63390000	-3.07750000
C	-1.38560000	10.99680000	-1.89140000
H	-1.79500000	10.32800000	-2.64480000
C	0.57930000	13.94550000	-4.64030000
H	1.14370000	13.06490000	-4.94530000
H	0.87730000	14.15680000	-3.61330000
C	0.98530000	15.13890000	-5.51350000
H	0.80420000	14.90330000	-6.56240000
H	0.35910000	15.99930000	-5.27800000
C	2.46100000	15.50730000	-5.30320000
H	2.62090000	15.77490000	-4.25750000
H	3.07980000	14.63040000	-5.49380000
C	2.93340000	16.65960000	-6.19740000
H	2.80060000	16.38550000	-7.24420000
H	2.30810000	17.53660000	-6.02920000
C	4.40430000	17.01700000	-5.94220000
H	5.01950000	16.12690000	-6.07500000
H	4.52960000	17.32700000	-4.90370000
C	4.91460000	18.12390000	-6.87270000
H	4.79960000	17.80730000	-7.90960000
H	4.29520000	19.01320000	-6.75550000
C	6.38410000	18.47980000	-6.60880000
H	6.99690000	17.58380000	-6.70850000
H	6.49850000	18.82030000	-5.57880000
C	6.90460000	19.55750000	-7.56800000
H	6.80000000	19.20880000	-8.59570000
H	6.28220000	20.44850000	-7.48470000
C	8.37010000	19.92780000	-7.30370000
H	8.98970000	19.03430000	-7.38080000
H	8.47620000	20.29160000	-6.28110000
C	8.88560000	20.98940000	-8.28370000
H	8.78940000	20.61800000	-9.30420000
H	8.25400000	21.87570000	-8.22170000
C	10.34540000	21.38180000	-8.02110000
H	10.44290000	21.76270000	-7.00410000
H	10.97650000	20.49520000	-8.08190000
C	10.85040000	22.43560000	-9.01530000
H	10.76460000	22.04760000	-10.03060000
H	10.20610000	23.31380000	-8.96900000
C	12.30260000	22.85290000	-8.75040000
H	12.38830000	23.24330000	-7.73600000
H	12.94750000	21.97540000	-8.79960000
C	12.79760000	23.90740000	-9.74920000
H	12.73390000	23.51280000	-10.76380000
H	12.14050000	24.77650000	-9.71800000
C	14.23650000	24.35270000	-9.47130000
H	14.33390000	24.76910000	-8.46900000
H	14.55110000	25.11910000	-10.17990000
H	14.93070000	23.51710000	-9.55700000
C	3.88890000	12.79560000	-2.85540000
H	4.22400000	12.77680000	-1.81730000
H	3.20420000	13.64000000	-2.94340000
C	5.09730000	13.02200000	-3.77550000
H	5.78730000	12.18270000	-3.67720000
H	4.76570000	13.02430000	-4.81410000

C	5.84290000	14.33220000	-3.47970000
H	6.16940000	14.34040000	-2.43870000
H	5.15990000	15.17470000	-3.59430000
C	7.05840000	14.52450000	-4.39920000
H	7.73920000	13.68050000	-4.28010000
H	6.73140000	14.50870000	-5.43890000
C	7.81870000	15.83020000	-4.12830000
H	8.14400000	15.85250000	-3.08750000
H	7.14410000	16.67690000	-4.25920000
C	9.03800000	15.99420000	-5.04830000
H	9.71240000	15.14780000	-4.91160000
H	8.71290000	15.96200000	-6.08800000
C	9.80520000	17.29970000	-4.79810000
H	10.13190000	17.33490000	-3.75830000
H	9.13310000	18.14650000	-4.94050000
C	11.02480000	17.45130000	-5.71920000
H	11.70210000	16.60980000	-5.56790000
H	10.70100000	17.40240000	-6.75850000
C	11.78400000	18.76460000	-5.48640000
H	11.10600000	19.60460000	-5.64080000
H	12.10900000	18.81610000	-4.44670000
C	13.00330000	18.91500000	-6.40730000
H	12.68200000	18.84650000	-7.44610000
H	13.69030000	18.08420000	-6.24130000
C	13.74430000	20.24160000	-6.19260000
H	13.05530000	21.06990000	-6.36040000
H	14.06680000	20.31290000	-5.15320000
C	14.96240000	20.39620000	-7.11390000
H	14.64340000	20.30660000	-8.15190000
H	15.66140000	19.57830000	-6.93430000
C	15.68240000	21.73720000	-6.91800000
H	14.98090000	22.55250000	-7.09750000
H	16.00320000	21.82710000	-5.87960000
C	16.89800000	21.89720000	-7.84130000
H	16.58440000	21.80160000	-8.88100000
H	17.60630000	21.08860000	-7.65700000
C	17.60950000	23.24190000	-7.65570000
H	16.94150000	24.07680000	-7.86850000
H	18.46480000	23.32430000	-8.32680000
H	17.97750000	23.35750000	-6.63590000
O	-0.64750000	9.66000000	1.43280000
Si	0.15060000	8.90860000	2.71630000
C	1.65640000	7.98160000	2.06020000
H	2.24640000	7.56090000	2.87290000
H	1.37400000	7.16210000	1.40380000
H	2.30690000	8.64500000	1.49230000
C	-1.04360000	7.72500000	3.57210000
H	-1.91520000	8.25250000	3.95570000
H	-1.40240000	6.95130000	2.89670000
H	-0.56970000	7.22590000	4.41570000
C	0.71470000	10.27390000	3.92120000
C	1.78940000	11.13670000	3.23800000
H	1.42740000	11.53680000	2.29030000
H	2.07920000	11.98230000	3.86180000
H	2.68960000	10.56050000	3.02390000
C	-0.50380000	11.14130000	4.26680000
H	-0.92950000	11.58580000	3.36710000
H	-1.28880000	10.54910000	4.73350000
H	-0.24670000	11.95250000	4.94780000
C	1.28850000	9.63790000	5.19870000
H	0.54110000	9.03300000	5.71320000
H	2.13760000	8.99120000	4.97540000
H	1.63050000	10.39920000	5.90030000
O	-3.38490000	8.93270000	-0.82820000
H	-3.92110000	9.58960000	-1.26880000
O	-3.70690000	11.44050000	-1.17310000
H	-4.17300000	11.81000000	-0.41860000
C	-0.36900000	14.33320000	1.36810000

C	-2.27310000	16.22370000	-0.36010000
O	-0.28540000	13.12920000	1.14740000
O	-2.70490000	17.34960000	-0.11040000
N	0.66140000	15.16960000	1.22890000
N	-1.47220000	15.98710000	-1.40530000
H	0.50430000	16.14260000	1.49610000
H	-1.17600000	15.01830000	-1.55580000
C	-1.70700000	14.97450000	1.75890000
H	-1.49810000	15.99820000	2.06190000
C	-2.32430000	14.31380000	3.01700000
H	-1.54530000	13.88230000	3.64650000
H	-2.78470000	15.08740000	3.63100000
C	-4.13410000	15.08820000	0.99990000
H	-4.25990000	15.97400000	1.62640000
C	-3.38970000	13.25490000	2.70950000
H	-2.91110000	12.41020000	2.21130000
C	-4.52310000	13.83850000	1.83100000
H	-5.37550000	14.12450000	2.44720000
C	-0.99520000	16.98730000	-2.35200000
H	-1.67690000	17.83880000	-2.39430000
H	-1.00630000	16.54100000	-3.34520000
C	1.92600000	14.86090000	0.58520000
H	1.72620000	14.54760000	-0.44130000
H	2.41570000	14.02800000	1.09270000
C	-2.66020000	15.02330000	0.52700000
H	-2.55080000	14.11370000	-0.06450000
C	0.42650000	17.45720000	-2.00620000
H	1.09430000	16.59510000	-1.97710000
H	0.43050000	17.89180000	-1.00520000
C	0.95870000	18.48470000	-3.01720000
H	0.90590000	18.06120000	-4.02040000
H	0.30350000	19.35650000	-3.02000000
C	2.40260000	18.93090000	-2.73650000
H	2.46430000	19.35800000	-1.73500000
H	3.06040000	18.06070000	-2.74470000
C	2.89970000	19.95850000	-3.76620000
H	2.82600000	19.53220000	-4.76670000
H	2.23920000	20.82590000	-3.75800000
C	4.34480000	20.41850000	-3.52090000
H	5.00980000	19.55430000	-3.54210000
H	4.42500000	20.84830000	-2.52230000
C	4.81290000	21.45010000	-4.56000000
H	4.73080000	21.02220000	-5.55890000
H	4.14340000	22.31020000	-4.54320000
C	6.25520000	21.92490000	-4.33020000
H	6.92710000	21.06640000	-4.34910000
H	6.33770000	22.36240000	-3.33530000
C	6.71170000	22.95310000	-5.37670000
H	6.63070000	22.51890000	-6.37300000
H	6.03730000	23.80950000	-5.36250000
C	8.15150000	23.43470000	-5.14810000
H	8.82620000	22.57820000	-5.15800000
H	8.22990000	23.88010000	-4.15660000
C	8.60950000	24.45570000	-6.19960000
H	8.53510000	24.01400000	-7.19320000
H	7.93310000	25.31080000	-6.19450000
C	10.04680000	24.94040000	-5.96370000
H	10.11710000	25.39340000	-4.97490000
H	10.72310000	24.08500000	-5.96180000
C	10.51060000	25.95400000	-7.01880000
H	10.44570000	25.50410000	-8.00950000
H	9.83190000	26.80710000	-7.02580000
C	11.94420000	26.44410000	-6.77320000
H	12.00390000	26.90480000	-5.78720000
H	12.62310000	25.59080000	-6.75740000
C	12.41260000	27.45080000	-7.83250000
H	12.36870000	26.99400000	-8.82140000
H	11.72940000	28.29990000	-7.85820000

C	13.83550000	27.95640000	-7.57400000
H	13.90600000	28.45530000	-6.60810000
H	14.13840000	28.67280000	-8.33790000
H	14.55460000	27.13710000	-7.58230000
C	2.80540000	16.11970000	0.60800000
H	3.11190000	16.31990000	1.63590000
H	2.20490000	16.97680000	0.30350000
C	4.04090000	16.06040000	-0.30030000
H	4.67280000	15.21550000	-0.02330000
H	3.72460000	15.89090000	-1.33070000
C	4.83980000	17.37030000	-0.21030000
H	5.22300000	17.48640000	0.80470000
H	4.16760000	18.21240000	-0.37620000
C	6.00030000	17.46700000	-1.20880000
H	6.68650000	16.63230000	-1.06020000
H	5.61640000	17.37780000	-2.22610000
C	6.75660000	18.79550000	-1.05590000
H	7.15290000	18.87000000	-0.04220000
H	6.05780000	19.62440000	-1.16930000
C	7.90230000	18.96360000	-2.06180000
H	8.60550000	18.13730000	-1.95210000
H	7.50910000	18.90490000	-3.07740000
C	8.64280000	20.29560000	-1.87270000
H	9.02810000	20.35530000	-0.85390000
H	7.93860000	21.11990000	-1.98100000
C	9.79810000	20.48220000	-2.86470000
H	10.50560000	19.66020000	-2.75190000
H	9.41590000	20.42520000	-3.88450000
C	10.53090000	21.81650000	-2.66650000
H	9.82710000	22.63810000	-2.79310000
H	10.89800000	21.88210000	-1.64130000
C	11.70230000	21.99740000	-3.64040000
H	11.33700000	21.92800000	-4.66560000
H	12.41010000	21.17870000	-3.50720000
C	12.42890000	23.33540000	-3.44760000
H	11.72680000	24.15380000	-3.60140000
H	12.77680000	23.41730000	-2.41700000
C	13.61740000	23.50080000	-4.40300000
H	13.26960000	23.41450000	-5.43290000
H	14.32240000	22.68410000	-4.24420000
C	14.34250000	24.84090000	-4.22180000
H	13.64390000	25.65720000	-4.40250000
H	14.67360000	24.94110000	-3.18730000
C	15.54610000	24.98480000	-5.16170000
H	15.21560000	24.88420000	-6.19560000
H	16.24930000	24.17150000	-4.98020000
C	16.27290000	26.32260000	-4.99540000
H	15.61230000	27.15860000	-5.22150000
H	17.12830000	26.38640000	-5.66840000
H	16.64010000	26.45110000	-3.97680000
O	-3.88480000	12.83360000	3.97130000
Si	-5.03780000	11.62950000	4.27020000
C	-4.61590000	10.09170000	3.27510000
H	-3.54990000	9.87640000	3.31590000
H	-5.14850000	9.21500000	3.63900000
H	-4.89740000	10.22910000	2.23260000
C	-6.76900000	12.24820000	3.83920000
H	-6.96770000	13.21790000	4.29290000
H	-6.90060000	12.34920000	2.76300000
H	-7.53500000	11.55850000	4.18880000
C	-4.96570000	11.25540000	6.14400000
C	-5.29440000	12.54260000	6.91940000
H	-6.30040000	12.89820000	6.69500000
H	-5.23340000	12.38730000	7.99670000
H	-4.60150000	13.34520000	6.66130000
C	-5.98450000	10.15820000	6.49920000
H	-7.00240000	10.46190000	6.25400000
H	-5.77970000	9.23250000	5.96100000

H	-5.96200000	9.92720000	7.56450000
C	-3.54910000	10.78420000	6.50800000
H	-3.27830000	9.87990000	5.96210000
H	-2.80970000	11.54950000	6.26760000
H	-3.46060000	10.56540000	7.57220000
O	-4.93860000	12.88150000	0.87830000
H	-5.30500000	13.46540000	0.20690000
O	-5.06340000	15.17040000	-0.08660000
H	-4.96250000	16.02670000	-0.48460000
C	3.43950000	-5.92580000	-14.14460000
C	1.23210000	-3.91710000	-14.26010000
O	3.46590000	-6.77720000	-15.03390000
O	1.59010000	-2.95600000	-13.57840000
N	4.37200000	-4.97470000	-14.01730000
N	1.12350000	-3.83660000	-15.59180000
H	4.23860000	-4.30730000	-13.27040000
H	0.81110000	-4.68000000	-16.07900000
C	2.31500000	-5.91470000	-13.09940000
H	2.66480000	-5.30560000	-12.26300000
C	2.06380000	-7.33670000	-12.56240000
H	1.63410000	-7.96320000	-13.34580000
H	3.00070000	-7.81330000	-12.27580000
C	-0.03370000	-5.21740000	-12.44200000
H	0.30720000	-4.47880000	-11.71930000
C	1.10150000	-7.29690000	-11.37020000
H	0.85670000	-8.31140000	-11.06170000
C	-0.20860000	-6.56650000	-11.70960000
H	-0.76500000	-6.40360000	-10.79040000
C	1.44420000	-2.67010000	-16.40420000
H	0.85160000	-1.82140000	-16.06150000
H	1.13500000	-2.87740000	-17.42910000
C	5.51300000	-4.79920000	-14.90190000
H	5.16960000	-4.78610000	-15.93720000
H	6.17940000	-5.65700000	-14.79850000
C	0.98420000	-5.29410000	-13.61240000
H	0.57480000	-5.96290000	-14.36580000
C	2.94910000	-2.34540000	-16.36130000
H	3.51350000	-3.22600000	-16.66630000
H	3.24710000	-2.13410000	-15.33430000
C	3.35510000	-1.15200000	-17.23450000
H	3.17400000	-1.38760000	-18.28340000
H	2.72890000	-0.29160000	-16.99900000
C	4.83080000	-0.78360000	-17.02420000
H	4.99070000	-0.51600000	-15.97850000
H	5.44960000	-1.66050000	-17.21480000
C	5.30320000	0.36870000	-17.91840000
H	5.17040000	0.09460000	-18.96520000
H	4.67790000	1.24570000	-17.75020000
C	6.77410000	0.72610000	-17.66320000
H	7.38930000	-0.16400000	-17.79600000
H	6.89940000	1.03610000	-16.62470000
C	7.28440000	1.83300000	-18.59370000
H	7.16940000	1.51640000	-19.63060000
H	6.66500000	2.72230000	-18.47650000
C	8.75390000	2.18890000	-18.32980000
H	9.36670000	1.29290000	-18.42950000
H	8.86830000	2.52940000	-17.29980000
C	9.27440000	3.26660000	-19.28900000
H	9.16980000	2.91790000	-20.31670000
H	8.65200000	4.15760000	-19.20570000
C	10.73990000	3.63690000	-19.02470000
H	11.35950000	2.74340000	-19.10180000
H	10.84600000	4.00070000	-18.00210000
C	11.25540000	4.69850000	-20.00470000
H	11.15920000	4.32710000	-21.02520000
H	10.62380000	5.58480000	-19.94270000
C	12.71520000	5.09090000	-19.74210000
H	12.81270000	5.47180000	-18.72510000

H	13.34630000	4.20430000	-19.80290000
C	13.22020000	6.14470000	-20.73630000
H	13.13440000	5.75670000	-21.75160000
H	12.57590000	7.02290000	-20.69000000
C	14.67240000	6.56200000	-20.47140000
H	14.75810000	6.95240000	-19.45700000
H	15.31730000	5.68450000	-20.52060000
C	15.16740000	7.61650000	-21.47020000
H	15.10370000	7.22190000	-22.48480000
H	14.51030000	8.48560000	-21.43900000
C	16.60630000	8.06180000	-21.19230000
H	16.70370000	8.47820000	-20.19000000
H	16.92090000	8.82820000	-21.90090000
H	17.30050000	7.22620000	-21.27800000
C	6.25870000	-3.49530000	-14.57640000
H	6.59380000	-3.51410000	-13.53830000
H	5.57400000	-2.65090000	-14.66440000
C	7.46710000	-3.26890000	-15.49650000
H	8.15710000	-4.10820000	-15.39820000
H	7.13550000	-3.26660000	-16.53510000
C	8.21270000	-1.95870000	-15.20070000
H	8.53920000	-1.95050000	-14.15970000
H	7.52970000	-1.11620000	-15.31530000
C	9.42820000	-1.76640000	-16.12020000
H	10.10900000	-2.61040000	-16.00110000
H	9.10120000	-1.78220000	-17.15990000
C	10.18850000	-0.46070000	-15.84930000
H	10.51380000	-0.43840000	-14.80850000
H	9.51390000	0.38600000	-15.98020000
C	11.40780000	-0.29670000	-16.76930000
H	12.08220000	-1.14310000	-16.63260000
H	11.08270000	-0.32890000	-17.80900000
C	12.17500000	1.00880000	-16.51910000
H	12.50170000	1.04400000	-15.47930000
H	11.50290000	1.85560000	-16.66150000
C	13.39460000	1.16040000	-17.44020000
H	14.07190000	0.31890000	-17.28890000
H	13.07080000	1.11150000	-18.47950000
C	14.15380000	2.47370000	-17.20740000
H	13.47580000	3.31370000	-17.36180000
H	14.47880000	2.52520000	-16.16770000
C	15.37310000	2.62410000	-18.12830000
H	15.05180000	2.55560000	-19.16710000
H	16.06010000	1.79330000	-17.96230000
C	16.11410000	3.95070000	-17.91360000
H	15.42510000	4.77900000	-18.08140000
H	16.43660000	4.02200000	-16.87420000
C	17.33220000	4.10530000	-18.83490000
H	17.01320000	4.01570000	-19.87290000
H	18.03120000	3.28740000	-18.65530000
C	18.05220000	5.44630000	-18.63900000
H	17.35070000	6.26160000	-18.81850000
H	18.37300000	5.53620000	-17.60060000
C	19.26780000	5.60630000	-19.56230000
H	18.95420000	5.51070000	-20.60200000
H	19.97610000	4.79770000	-19.37800000
C	19.97930000	6.95100000	-19.37670000
H	19.31130000	7.78590000	-19.58950000
H	20.83460000	7.03340000	-20.04780000
H	20.34730000	7.06660000	-18.35690000
O	1.72230000	-6.63090000	-10.28820000
Si	2.52040000	-7.38230000	-9.00470000
C	4.02620000	-8.30930000	-9.66080000
H	4.61620000	-8.73000000	-8.84810000
H	3.74380000	-9.12880000	-10.31720000
H	4.67670000	-7.64590000	-10.22870000
C	1.32620000	-8.56590000	-8.14890000
H	0.45460000	-8.03840000	-7.76530000

H	0.96740000	-9.33960000	-8.82430000
H	1.80010000	-9.06500000	-7.30530000
C	3.08450000	-6.01700000	-7.79980000
C	4.15920000	-5.15420000	-8.48300000
H	3.79720000	-4.75410000	-9.43070000
H	4.44900000	-4.30860000	-7.85920000
H	5.05940000	-5.73040000	-8.69710000
C	1.86600000	-5.14960000	-7.45420000
H	1.44030000	-4.70510000	-8.35390000
H	1.08100000	-5.74180000	-6.98750000
H	2.12310000	-4.33840000	-6.77320000
C	3.65830000	-6.65300000	-6.52230000
H	2.91090000	-7.25790000	-6.00780000
H	4.50740000	-7.29970000	-6.74560000
H	4.00030000	-5.89170000	-5.82070000
O	-1.01510000	-7.35820000	-12.54920000
H	-1.55130000	-6.70130000	-12.98980000
O	-1.33710000	-4.85040000	-12.89410000
H	-1.80320000	-4.48090000	-12.13960000
C	2.00080000	-1.95770000	-10.35290000
C	0.09670000	-0.06720000	-12.08110000
O	2.08440000	-3.16170000	-10.57360000
O	-0.33510000	1.05870000	-11.83140000
N	3.03120000	-1.12130000	-10.49210000
N	0.89760000	-0.30380000	-13.12630000
H	2.87410000	-0.14830000	-10.22490000
H	1.19380000	-1.27260000	-13.27680000
C	0.66280000	-1.31640000	-9.96210000
H	0.87170000	-0.29270000	-9.65910000
C	0.04550000	-1.97710000	-8.70400000
H	0.82450000	-2.40860000	-8.07450000
H	-0.41490000	-1.20350000	-8.09000000
C	-1.76430000	-1.20270000	-10.72110000
H	-1.89010000	-0.31690000	-10.09460000
C	-1.01990000	-3.03600000	-9.01150000
H	-0.54130000	-3.88070000	-9.50970000
C	-2.15330000	-2.45240000	-9.89000000
H	-3.00570000	-2.16640000	-9.27380000
C	1.37460000	0.69640000	-14.07300000
H	0.69290000	1.54790000	-14.11530000
H	1.36350000	0.25010000	-15.06620000
C	4.29580000	-1.43000000	-11.13580000
H	4.09600000	-1.74330000	-12.16230000
H	4.78550000	-2.26290000	-10.62830000
C	-0.29040000	-1.26760000	-11.19400000
H	-0.18100000	-2.17720000	-11.78550000
C	2.79630000	1.16630000	-13.72720000
H	3.46410000	0.30420000	-13.69810000
H	2.80030000	1.60090000	-12.72620000
C	3.32850000	2.19380000	-14.73820000
H	3.27570000	1.77030000	-15.74140000
H	2.67330000	3.06560000	-14.74100000
C	4.77240000	2.64000000	-14.45750000
H	4.83410000	3.06710000	-13.45600000
H	5.43020000	1.76980000	-14.46570000
C	5.26950000	3.66760000	-15.48720000
H	5.19580000	3.24130000	-16.48770000
H	4.60900000	4.53500000	-15.47900000
C	6.71460000	4.12760000	-15.24190000
H	7.37960000	3.26340000	-15.26310000
H	6.79480000	4.55740000	-14.24330000
C	7.18270000	5.15920000	-16.28100000
H	7.10060000	4.73130000	-17.27990000
H	6.51320000	6.01930000	-16.26420000
C	8.62500000	5.63400000	-16.05120000
H	9.29690000	4.77550000	-16.07010000
H	8.70750000	6.07150000	-15.05630000
C	9.08150000	6.66220000	-17.09770000

H	9.00050000	6.22800000	-18.09400000
H	8.40710000	7.51860000	-17.08350000
C	10.52130000	7.14380000	-16.86910000
H	11.19600000	6.28730000	-16.87900000
H	10.59970000	7.58920000	-15.87760000
C	10.97930000	8.16480000	-17.92060000
H	10.90490000	7.72310000	-18.91420000
H	10.30290000	9.01990000	-17.91550000
C	12.41660000	8.64950000	-17.68470000
H	12.48690000	9.10250000	-16.69590000
H	13.09290000	7.79410000	-17.68280000
C	12.88040000	9.66310000	-18.73980000
H	12.81550000	9.21320000	-19.73050000
H	12.20170000	10.51620000	-18.74680000
C	14.31400000	10.15320000	-18.49420000
H	14.37370000	10.61390000	-17.50820000
H	14.99290000	9.29990000	-18.47840000
C	14.78240000	11.15990000	-19.55350000
H	14.73850000	10.70310000	-20.54240000
H	14.09920000	12.00900000	-19.57920000
C	16.20530000	11.66550000	-19.29500000
H	16.27580000	12.16440000	-18.32910000
H	16.50820000	12.38190000	-20.05890000
H	16.92440000	10.84620000	-19.30330000
C	5.17520000	-0.17120000	-11.11300000
H	5.48170000	0.02900000	-10.08510000
H	4.57470000	0.68590000	-11.41750000
C	6.41070000	-0.23050000	-12.02130000
H	7.04260000	-1.07540000	-11.74430000
H	6.09440000	-0.40000000	-13.05170000
C	7.20960000	1.07940000	-11.93130000
H	7.59280000	1.19550000	-10.91630000
H	6.53740000	1.92150000	-12.09720000
C	8.37010000	1.17610000	-12.92980000
H	9.05630000	0.34140000	-12.78120000
H	7.98620000	1.08690000	-13.94710000
C	9.12640000	2.50460000	-12.77690000
H	9.52270000	2.57910000	-11.76320000
H	8.42760000	3.33350000	-12.89030000
C	10.27210000	2.67270000	-13.78280000
H	10.97530000	1.84640000	-13.67310000
H	9.87890000	2.61400000	-14.79840000
C	11.01260000	4.00470000	-13.59370000
H	11.39790000	4.06440000	-12.57490000
H	10.30840000	4.82900000	-13.70200000
C	12.16790000	4.19130000	-14.58570000
H	12.87540000	3.36930000	-14.47290000
H	11.78570000	4.13430000	-15.60550000
C	12.90070000	5.52560000	-14.38750000
H	12.19690000	6.34720000	-14.51410000
H	13.26780000	5.59120000	-13.36230000
C	14.07210000	5.70650000	-15.36140000
H	13.70680000	5.63710000	-16.38660000
H	14.77990000	4.88780000	-15.22820000
C	14.79870000	7.04450000	-15.16860000
H	14.09660000	7.86290000	-15.32240000
H	15.14660000	7.12640000	-14.13800000
C	15.98720000	7.20990000	-16.12400000
H	15.63940000	7.12360000	-17.15390000
H	16.69220000	6.39320000	-15.96520000
C	16.71230000	8.55000000	-15.94280000
H	16.01370000	9.36630000	-16.12350000
H	17.04340000	8.65020000	-14.90830000
C	17.91590000	8.69390000	-16.88270000
H	17.58540000	8.59330000	-17.91660000
H	18.61910000	7.88060000	-16.70120000
C	18.64270000	10.03170000	-16.71640000
H	17.98210000	10.86770000	-16.94250000

H	19.49810000	10.09550000	-17.38940000
H	19.00990000	10.16020000	-15.69780000
O	-1.51500000	-3.45730000	-7.74970000
Si	-2.66800000	-4.66140000	-7.45080000
C	-2.24610000	-6.19920000	-8.44590000
H	-1.18010000	-6.41450000	-8.40510000
H	-2.77870000	-7.07590000	-8.08200000
H	-2.52760000	-6.06180000	-9.48840000
C	-4.39920000	-4.04270000	-7.88180000
H	-4.59790000	-3.07300000	-7.42810000
H	-4.53080000	-3.94170000	-8.95800000
H	-5.16520000	-4.73240000	-7.53220000
C	-2.59590000	-5.03550000	-5.57700000
C	-2.92460000	-3.74830000	-4.80160000
H	-3.93060000	-3.39270000	-5.02600000
H	-2.86360000	-3.90360000	-3.72430000
H	-2.23170000	-2.94570000	-5.05970000
C	-3.61470000	-6.13270000	-5.22180000
H	-4.63260000	-5.82900000	-5.46700000
H	-3.40990000	-7.05840000	-5.76000000
H	-3.59220000	-6.36370000	-4.15650000
C	-1.17930000	-5.50670000	-5.21300000
H	-0.90850000	-6.41100000	-5.75890000
H	-0.43990000	-4.74140000	-5.45340000
H	-1.09080000	-5.72550000	-4.14880000
O	-2.56880000	-3.40940000	-10.84270000
H	-2.93520000	-2.82550000	-11.51410000
O	-2.69360000	-1.12050000	-11.80760000
H	-2.59270000	-0.26420000	-12.20560000
C	2.71840000	2.09300000	-8.47000000
C	0.51100000	4.10170000	-8.58550000
O	2.74480000	1.24160000	-9.35930000
O	0.86900000	5.06280000	-7.90380000
N	3.65090000	3.04410000	-8.34270000
N	0.40240000	4.18220000	-9.91720000
H	3.51750000	3.71150000	-7.59580000
H	0.09000000	3.33880000	-10.40440000
C	1.59390000	2.10410000	-7.42480000
H	1.94370000	2.71320000	-6.58840000
C	1.34270000	0.68210000	-6.88780000
H	0.91300000	0.05560000	-7.67120000
H	2.27960000	0.20550000	-6.60120000
C	-0.75480000	2.80140000	-6.76740000
H	-0.41390000	3.54000000	-6.04470000
C	0.38040000	0.72190000	-5.69560000
H	0.13560000	-0.29260000	-5.38710000
C	-0.92970000	1.45230000	-6.03500000
H	-1.48610000	1.61520000	-5.11580000
C	0.72310000	5.34870000	-10.72960000
H	0.13050000	6.19740000	-10.38690000
H	0.41390000	5.14140000	-11.75450000
C	4.79190000	3.21960000	-9.22730000
H	4.44850000	3.23270000	-10.26260000
H	5.45830000	2.36180000	-9.12390000
C	0.26310000	2.72470000	-7.93780000
H	-0.14630000	2.05590000	-8.69120000
C	2.22800000	5.67340000	-10.68670000
H	2.79240000	4.79280000	-10.99170000
H	2.52600000	5.88470000	-9.65970000
C	2.63400000	6.86680000	-11.55990000
H	2.45290000	6.63120000	-12.60880000
H	2.00780000	7.72720000	-11.32440000
C	4.10970000	7.23520000	-11.34960000
H	4.26960000	7.50280000	-10.30390000
H	4.72850000	6.35830000	-11.54020000
C	4.58210000	8.38750000	-12.24380000
H	4.44930000	8.11340000	-13.29060000
H	3.95680000	9.26450000	-12.07560000

C	6.05300000	8.74490000	-11.98860000
H	6.66820000	7.85480000	-12.12140000
H	6.17830000	9.05490000	-10.95010000
C	6.56330000	9.85180000	-12.91910000
H	6.44830000	9.53520000	-13.95600000
H	5.94390000	10.74110000	-12.80190000
C	8.03280000	10.20770000	-12.65520000
H	8.64560000	9.31170000	-12.75490000
H	8.14720000	10.54820000	-11.62520000
C	8.55330000	11.28540000	-13.61440000
H	8.44870000	10.93670000	-14.64210000
H	7.93090000	12.17640000	-13.53110000
C	10.01880000	11.65570000	-13.35010000
H	10.63840000	10.76220000	-13.42720000
H	10.12490000	12.01950000	-12.32750000
C	10.53430000	12.71730000	-14.33010000
H	10.43810000	12.34590000	-15.35060000
H	9.90270000	13.60360000	-14.26810000
C	11.99410000	13.10970000	-14.06750000
H	12.09160000	13.49060000	-13.05050000
H	12.62520000	12.22310000	-14.12830000
C	12.49910000	14.16350000	-15.06170000
H	12.41330000	13.77550000	-16.07700000
H	11.85480000	15.04170000	-15.01540000
C	13.95130000	14.58080000	-14.79680000
H	14.03700000	14.97120000	-13.78240000
H	14.59620000	13.70330000	-14.84600000
C	14.44630000	15.63530000	-15.79560000
H	14.38260000	15.24070000	-16.81020000
H	13.78920000	16.50440000	-15.76440000
C	15.88520000	16.08060000	-15.51770000
H	15.98260000	16.49700000	-14.51540000
H	16.19980000	16.84700000	-16.22630000
H	16.57940000	15.24500000	-15.60340000
C	5.53760000	4.52350000	-8.90180000
H	5.87270000	4.50470000	-7.86370000
H	4.85290000	5.36790000	-8.98980000
C	6.74600000	4.74990000	-9.82190000
H	7.43600000	3.91060000	-9.72360000
H	6.41440000	4.75220000	-10.86050000
C	7.49160000	6.06010000	-9.52610000
H	7.81810000	6.06830000	-8.48510000
H	6.80860000	6.90260000	-9.64070000
C	8.70710000	6.25240000	-10.44560000
H	9.38790000	5.40840000	-10.32650000
H	8.38010000	6.23660000	-11.48530000
C	9.46740000	7.55810000	-10.17470000
H	9.79270000	7.58040000	-9.13390000
H	8.79280000	8.40480000	-10.30560000
C	10.68670000	7.72210000	-11.09470000
H	11.36110000	6.87570000	-10.95800000
H	10.36160000	7.68990000	-12.13440000
C	11.45390000	9.02760000	-10.84450000
H	11.78060000	9.06280000	-9.80470000
H	10.78180000	9.87440000	-10.98690000
C	12.67350000	9.17920000	-11.76560000
H	13.35080000	8.33770000	-11.61430000
H	12.34970000	9.13030000	-12.80490000
C	13.43270000	10.49250000	-11.53280000
H	12.75470000	11.33250000	-11.68720000
H	13.75770000	10.54400000	-10.49310000
C	14.65200000	10.64290000	-12.45370000
H	14.33070000	10.57440000	-13.49250000
H	15.33900000	9.81210000	-12.28770000
C	15.39300000	11.96950000	-12.23900000
H	14.70400000	12.79780000	-12.40680000
H	15.71550000	12.04080000	-11.19960000
C	16.61110000	12.12410000	-13.16030000

H	16.29210000	12.03450000	-14.19830000
H	17.31010000	11.30620000	-12.98070000
C	17.33110000	13.46510000	-12.96440000
H	16.62960000	14.28040000	-13.14390000
H	17.65190000	13.55500000	-11.92600000
C	18.54670000	13.62510000	-13.88770000
H	18.23310000	13.52950000	-14.92740000
H	19.25500000	12.81650000	-13.70340000
C	19.25820000	14.96980000	-13.70210000
H	18.59020000	15.80470000	-13.91490000
H	20.11350000	15.05220000	-14.37320000
H	19.62620000	15.08540000	-12.68230000
O	1.00120000	1.38790000	-4.61360000
Si	1.79930000	0.63650000	-3.33010000
C	3.30510000	-0.29050000	-3.98620000
H	3.89510000	-0.71120000	-3.17350000
H	3.02270000	-1.11000000	-4.64260000
H	3.95560000	0.37290000	-4.55410000
C	0.60510000	-0.54710000	-2.47430000
H	-0.26650000	-0.01960000	-2.09070000
H	0.24630000	-1.32080000	-3.14970000
H	1.07900000	-1.04620000	-1.63070000
C	2.36340000	2.00180000	-2.12520000
C	3.43810000	2.86460000	-2.80840000
H	3.07610000	3.26470000	-3.75610000
H	3.72790000	3.71020000	-2.18460000
H	4.33830000	2.28840000	-3.02250000
C	1.14490000	2.86920000	-1.77960000
H	0.71920000	3.31370000	-2.67930000
H	0.35990000	2.27700000	-1.31290000
H	1.40200000	3.68040000	-1.09860000
C	2.93720000	1.36580000	-0.84770000
H	2.18980000	0.76090000	-0.33320000
H	3.78630000	0.71910000	-1.07100000
H	3.27920000	2.12710000	-0.14610000
O	-1.73620000	0.66060000	-6.87460000
H	-2.27240000	1.31750000	-7.31520000
O	-2.05820000	3.16840000	-7.21950000
H	-2.52430000	3.53790000	-6.46500000
C	1.27970000	6.06110000	-4.67830000
C	-0.62440000	7.95160000	-6.40650000
O	1.36330000	4.85710000	-4.89900000
O	-1.05620000	9.07750000	-6.15680000
N	2.31010000	6.89750000	-4.81750000
N	0.17650000	7.71500000	-7.45170000
H	2.15300000	7.87050000	-4.55030000
H	0.47270000	6.74620000	-7.60220000
C	-0.05830000	6.70240000	-4.28750000
H	0.15060000	7.72610000	-3.98450000
C	-0.67560000	6.04170000	-3.02940000
H	0.10340000	5.61020000	-2.39990000
H	-1.13600000	6.81530000	-2.41540000
C	-2.48540000	6.81610000	-5.04650000
H	-2.61120000	7.70190000	-4.42000000
C	-1.74100000	4.98280000	-3.33690000
H	-1.26240000	4.13810000	-3.83510000
C	-2.87440000	5.56640000	-4.21540000
H	-3.72680000	5.85240000	-3.59920000
C	0.65350000	8.71520000	-8.39840000
H	-0.02820000	9.56670000	-8.44070000
H	0.64240000	8.26890000	-9.39160000
C	3.57470000	6.58880000	-5.46120000
H	3.37490000	6.27550000	-6.48770000
H	4.06440000	5.75590000	-4.95370000
C	-1.01150000	6.75120000	-5.51940000
H	-0.90210000	5.84160000	-6.11090000
C	2.07520000	9.18510000	-8.05260000
H	2.74300000	8.32300000	-8.02350000

H	2.07920000	9.61970000	-7.05160000
C	2.60740000	10.21260000	-9.06360000
H	2.55460000	9.78910000	-10.06680000
H	1.95220000	11.08440000	-9.06640000
C	4.05130000	10.65880000	-8.78290000
H	4.11300000	11.08590000	-7.78140000
H	4.70910000	9.78860000	-8.79110000
C	4.54840000	11.68640000	-9.81260000
H	4.47470000	11.26010000	-10.81310000
H	3.88790000	12.55380000	-9.80440000
C	5.99350000	12.14640000	-9.56730000
H	6.65850000	11.28220000	-9.58850000
H	6.07370000	12.57620000	-8.56870000
C	6.46160000	13.17800000	-10.60640000
H	6.37950000	12.75010000	-11.60530000
H	5.79210000	14.03810000	-10.58960000
C	7.90390000	13.65280000	-10.37660000
H	8.57580000	12.79430000	-10.39550000
H	7.98640000	14.09030000	-9.38170000
C	8.36040000	14.68100000	-11.42310000
H	8.27940000	14.24680000	-12.41940000
H	7.68600000	15.53740000	-11.40890000
C	9.80020000	15.16260000	-11.19450000
H	10.47490000	14.30610000	-11.20440000
H	9.87860000	15.60800000	-10.20300000
C	10.25820000	16.18360000	-12.24600000
H	10.18380000	15.74190000	-13.23960000
H	9.58180000	17.03870000	-12.24090000
C	11.69550000	16.66830000	-12.01010000
H	11.76580000	17.12130000	-11.02130000
H	12.37180000	15.81290000	-12.00820000
C	12.15930000	17.68190000	-13.06520000
H	12.09440000	17.23200000	-14.05590000
H	11.48060000	18.53500000	-13.07220000
C	13.59290000	18.17200000	-12.81960000
H	13.65260000	18.63270000	-11.83360000
H	14.27180000	17.31870000	-12.80380000
C	14.06130000	19.17870000	-13.87890000
H	14.01740000	18.72190000	-14.86780000
H	13.37810000	20.02780000	-13.90460000
C	15.48420000	19.68430000	-13.62040000
H	15.55470000	20.18320000	-12.65450000
H	15.78710000	20.40070000	-14.38430000
H	16.20330000	18.86500000	-13.62870000
C	4.45410000	7.84760000	-5.43840000
H	4.76060000	8.04780000	-4.41050000
H	3.85360000	8.70470000	-5.74290000
C	5.68960000	7.78830000	-6.34670000
H	6.32150000	6.94340000	-6.06970000
H	5.37330000	7.61880000	-7.37710000
C	6.48850000	9.09820000	-6.25670000
H	6.87170000	9.21430000	-5.24170000
H	5.81630000	9.94030000	-6.42260000
C	7.64900000	9.19490000	-7.25520000
H	8.33520000	8.36020000	-7.10660000
H	7.26510000	9.10570000	-8.27250000
C	8.40530000	10.52340000	-7.10230000
H	8.80160000	10.59790000	-6.08860000
H	7.70650000	11.35230000	-7.21570000
C	9.55100000	10.69150000	-8.10820000
H	10.25420000	9.86520000	-7.99850000
H	9.15780000	10.63280000	-9.12380000
C	10.29150000	12.02350000	-7.91910000
H	10.67680000	12.08320000	-6.90030000
H	9.58730000	12.84780000	-8.02740000
C	11.44680000	12.21010000	-8.91110000
H	12.15430000	11.38810000	-8.79830000
H	11.06460000	12.15310000	-9.93090000

C	12.17960000	13.54440000	-8.71290000
H	11.47580000	14.36600000	-8.83950000
H	12.54670000	13.61000000	-7.68770000
C	13.35100000	13.72530000	-9.68680000
H	12.98570000	13.65590000	-10.71200000
H	14.05880000	12.90660000	-9.55360000
C	14.07760000	15.06330000	-9.49400000
H	13.37550000	15.88170000	-9.64780000
H	14.42550000	15.14520000	-8.46340000
C	15.26610000	15.22870000	-10.44940000
H	14.91830000	15.14240000	-11.47930000
H	15.97110000	14.41200000	-10.29060000
C	15.99120000	16.56880000	-10.26820000
H	15.29260000	17.38510000	-10.44890000
H	16.32230000	16.66900000	-9.23370000
C	17.19480000	16.71270000	-11.20810000
H	16.86430000	16.61210000	-12.24200000
H	17.89800000	15.89940000	-11.02660000
C	17.92160000	18.05050000	-11.04180000
H	17.26100000	18.88650000	-11.26790000
H	18.77700000	18.11430000	-11.71480000
H	18.28880000	18.17900000	-10.02320000
O	-2.23610000	4.56150000	-2.07510000
Si	-3.38910000	3.35740000	-1.77620000
C	-2.96720000	1.81960000	-2.77130000
H	-1.90120000	1.60430000	-2.73050000
H	-3.49980000	0.94290000	-2.40740000
H	-3.24870000	1.95700000	-3.81380000
C	-5.12030000	3.97610000	-2.20720000
H	-5.31900000	4.94580000	-1.75350000
H	-5.25190000	4.07710000	-3.28340000
H	-5.88630000	3.28640000	-1.85760000
C	-3.31700000	2.98330000	0.09760000
C	-3.64570000	4.27050000	0.87300000
H	-4.65170000	4.62610000	0.64860000
H	-3.58470000	4.11520000	1.95030000
H	-2.95280000	5.07310000	0.61490000
C	-4.33580000	1.88610000	0.45280000
H	-5.35370000	2.18980000	0.20760000
H	-4.13100000	0.96040000	-0.08540000
H	-4.31330000	1.65510000	1.51810000
C	-1.90040000	2.51210000	0.46160000
H	-1.62960000	1.60780000	-0.08430000
H	-1.16100000	3.27740000	0.22120000
H	-1.81190000	2.29330000	1.52580000
O	-3.28990000	4.60940000	-5.16810000
H	-3.65630000	5.19330000	-5.83950000
O	-3.41470000	6.89830000	-6.13300000
H	-3.31380000	7.75460000	-6.53100000

4-beta monomer

C	-11.27900000	0.33430000	-9.31330000
C	-9.09740000	0.76340000	-7.29770000
O	-11.42450000	-0.77520000	-9.82870000
O	-9.33780000	1.77910000	-6.64220000
N	-12.16680000	0.86220000	-8.46370000
N	-9.14810000	-0.45590000	-6.74900000
H	-11.94200000	1.76630000	-8.07240000
H	-8.94590000	-1.24460000	-7.36860000
C	-10.05650000	1.20600000	-9.63450000
H	-10.33280000	2.23460000	-9.39330000
C	-9.72390000	1.15330000	-11.13740000
H	-9.33000000	0.16880000	-11.39780000
H	-10.62380000	1.27580000	-11.73670000
C	-7.66620000	1.86750000	-9.11280000

H	-7.91670000	2.81930000	-8.64760000
C	-8.68270000	2.22410000	-11.49150000
H	-8.39070000	2.12300000	-12.53440000
C	-7.41420000	2.09730000	-10.62450000
H	-6.79550000	2.98410000	-10.75850000
C	-9.49230000	-0.74350000	-5.36200000
H	-8.85760000	-0.14950000	-4.70230000
H	-9.26030000	-1.79010000	-5.16420000
C	-13.37200000	0.19150000	-8.00800000
H	-13.10710000	-0.79110000	-7.61550000
H	-14.03470000	0.02740000	-8.85930000
C	-8.79150000	0.84770000	-8.80930000
H	-8.44150000	-0.12300000	-9.14920000
C	-10.98110000	-0.46360000	-5.08480000
H	-11.58770000	-1.04430000	-5.77920000
H	-11.19670000	0.58420000	-5.29460000
C	-11.42000000	-0.76830000	-3.64650000
H	-11.29470000	-1.83220000	-3.44450000
H	-10.77400000	-0.24260000	-2.94320000
C	-12.88010000	-0.35480000	-3.40800000
H	-12.98320000	0.71830000	-3.57650000
H	-13.51450000	-0.84380000	-4.14750000
C	-13.39290000	-0.69770000	-2.00310000
H	-13.30430000	-1.77150000	-1.83670000
H	-12.76370000	-0.21860000	-1.25280000
C	-14.85320000	-0.26520000	-1.80530000
H	-15.46930000	-0.71750000	-2.58260000
H	-14.93440000	0.81470000	-1.93730000
C	-15.41310000	-0.65660000	-0.43130000
H	-15.33610000	-1.73650000	-0.30290000
H	-14.80120000	-0.21210000	0.35370000
C	-16.87560000	-0.22460000	-0.25250000
H	-17.47900000	-0.65700000	-1.05080000
H	-16.95220000	0.85820000	-0.35930000
C	-17.45540000	-0.64890000	1.10340000
H	-17.38510000	-1.73220000	1.20480000
H	-16.84930000	-0.22870000	1.90610000
C	-18.91700000	-0.21460000	1.28150000
H	-19.51880000	-0.63140000	0.47400000
H	-18.98880000	0.87010000	1.19300000
C	-19.50030000	-0.65920000	2.62940000
H	-19.43590000	-1.74440000	2.71230000
H	-18.89190000	-0.25570000	3.43880000
C	-20.95880000	-0.21980000	2.81700000
H	-21.02470000	0.86610000	2.74200000
H	-21.56590000	-0.62330000	2.00650000
C	-21.53830000	-0.67710000	4.16220000
H	-21.48260000	-1.76380000	4.23120000
H	-20.92200000	-0.28840000	4.97300000
C	-22.99130000	-0.22710000	4.36410000
H	-23.04690000	0.86000000	4.29900000
H	-23.60790000	-0.61710000	3.55420000
C	-23.56540000	-0.68930000	5.71020000
H	-23.53190000	-1.77760000	5.77110000
H	-22.93900000	-0.31890000	6.52170000
C	-25.00600000	-0.21680000	5.92950000
H	-25.07460000	0.87020000	5.89450000
H	-25.37840000	-0.54040000	6.90170000
H	-25.67290000	-0.62040000	5.16810000
C	-14.08110000	1.01700000	-6.92520000
H	-14.35750000	1.99280000	-7.32770000
H	-13.39680000	1.20530000	-6.09730000
C	-15.33350000	0.30590000	-6.39850000
H	-16.01900000	0.12340000	-7.22730000
H	-15.05470000	-0.67380000	-6.00990000
C	-16.06060000	1.09480000	-5.30210000
H	-16.34050000	2.07830000	-5.68220000
H	-15.38490000	1.26780000	-4.46370000

C	-17.31330000	0.35620000	-4.81290000
H	-17.97990000	0.17740000	-5.65760000
H	-17.02960000	-0.62540000	-4.43290000
C	-18.07410000	1.12030000	-3.72350000
H	-18.35410000	2.10530000	-4.09880000
H	-17.41750000	1.28850000	-2.86940000
C	-19.33260000	0.36880000	-3.26950000
H	-19.98200000	0.19660000	-4.12880000
H	-19.05100000	-0.61520000	-2.89480000
C	-20.11510000	1.12040000	-2.18660000
H	-20.39560000	2.10560000	-2.56080000
H	-19.47010000	1.28780000	-1.32360000
C	-21.37660000	0.36500000	-1.74810000
H	-22.02070000	0.19970000	-2.61270000
H	-21.09720000	-0.62130000	-1.37840000
C	-22.16220000	1.11150000	-0.66330000
H	-21.51780000	1.27510000	0.20100000
H	-22.44130000	2.09860000	-1.03320000
C	-23.42470000	0.35730000	-0.22600000
H	-23.14730000	-0.63230000	0.13550000
H	-24.07310000	0.20090000	-1.08900000
C	-24.20120000	1.09970000	0.86870000
H	-23.55050000	1.25570000	1.72970000
H	-24.47920000	2.09030000	0.50710000
C	-25.46340000	0.34790000	1.31150000
H	-25.18730000	-0.64630000	1.66140000
H	-26.12050000	0.20200000	0.45320000
C	-26.22540000	1.08480000	2.42060000
H	-25.56530000	1.23110000	3.27610000
H	-26.50240000	2.07940000	2.06940000
C	-27.48630000	0.33500000	2.87050000
H	-27.21580000	-0.66210000	3.21800000
H	-28.15230000	0.19410000	2.01830000
C	-28.24120000	1.06680000	3.98570000
H	-27.61610000	1.19500000	4.86980000
H	-29.12770000	0.50770000	4.28620000
H	-28.56790000	2.05470000	3.66000000
O	-9.23770000	3.51570000	-11.31580000
Si	-10.08760000	4.39600000	-12.48530000
C	-11.64300000	3.46000000	-12.99870000
H	-12.27950000	4.07360000	-13.63420000
H	-11.41070000	2.55510000	-13.55430000
H	-12.23240000	3.17130000	-12.12950000
C	-8.97190000	4.67410000	-13.98100000
H	-8.11990000	5.30180000	-13.72700000
H	-8.58160000	3.73920000	-14.37850000
H	-9.50960000	5.17230000	-14.78620000
C	-10.60590000	6.06350000	-11.71330000
C	-11.67510000	5.80530000	-10.63770000
H	-11.32730000	5.07840000	-9.90440000
H	-11.93260000	6.71800000	-10.10030000
H	-12.59310000	5.40940000	-11.07230000
C	-9.37110000	6.71580000	-11.07430000
H	-8.90280000	6.03950000	-10.35810000
H	-8.62250000	6.96600000	-11.82460000
H	-9.62730000	7.63380000	-10.54550000
C	-11.18030000	6.98580000	-12.80240000
H	-10.44230000	7.19500000	-13.57750000
H	-12.04760000	6.53810000	-13.28850000
H	-11.49520000	7.94250000	-12.38500000
O	-6.63850000	0.98870000	-11.02220000
H	-6.39650000	0.64150000	-10.15850000
O	-6.42750000	1.39170000	-8.59120000
H	-5.94300000	2.16560000	-8.27940000

4-beta dimer

C	-9.64340000	5.27170000	-7.04680000
C	-8.11140000	4.38940000	-4.42800000
O	-9.69950000	4.53380000	-8.02630000
O	-7.92990000	5.21050000	-3.52730000
N	-10.71170000	5.60200000	-6.31740000
N	-8.81420000	3.26510000	-4.24940000
H	-10.56070000	6.26140000	-5.55240000
H	-8.95070000	2.66880000	-5.06730000
C	-8.29650000	5.78950000	-6.53120000
H	-8.50460000	6.55740000	-5.78800000
C	-7.46660000	6.50820000	-7.62850000
H	-8.12200000	6.95430000	-8.37640000
H	-6.94320000	7.34990000	-7.17250000
C	-6.02690000	4.92230000	-5.82340000
H	-5.87210000	5.89350000	-5.34810000
C	-6.42650000	5.60490000	-8.31140000
H	-6.94740000	4.85230000	-8.90350000
C	-5.48070000	4.95110000	-7.27350000
H	-4.51450000	5.45580000	-7.28270000
C	-9.36340000	2.81120000	-2.98230000
H	-8.70100000	3.09620000	-2.16230000
H	-9.38280000	1.72200000	-2.99850000
C	-12.00150000	4.93560000	-6.36810000
H	-11.84430000	3.85870000	-6.27820000
H	-12.48090000	5.11750000	-7.33120000
C	-7.53820000	4.62260000	-5.83550000
H	-7.67550000	3.70460000	-6.40720000
C	-10.77700000	3.36330000	-2.74920000
H	-11.42330000	3.07030000	-3.57790000
H	-10.74420000	4.45400000	-2.74630000
C	-11.37130000	2.86370000	-1.42620000
H	-11.36120000	1.77350000	-1.41820000
H	-10.73080000	3.18210000	-0.60260000
C	-12.80280000	3.35840000	-1.18060000
H	-12.81660000	4.44880000	-1.18230000
H	-13.44570000	3.03650000	-2.00120000
C	-13.36650000	2.83790000	0.14890000
H	-13.33670000	1.74820000	0.15240000
H	-12.72210000	3.16180000	0.96670000
C	-14.80290000	3.30560000	0.41640000
H	-15.45040000	2.97360000	-0.39600000
H	-14.83550000	4.39530000	0.41540000
C	-15.34550000	2.77930000	1.75280000
H	-15.30820000	1.69020000	1.75660000
H	-14.69530000	3.10840000	2.56360000
C	-16.78190000	3.23980000	2.03280000
H	-17.43310000	2.90770000	1.22370000
H	-16.81810000	4.32920000	2.03400000
C	-17.31860000	2.71200000	3.37070000
H	-17.28040000	1.62280000	3.37430000
H	-16.66720000	3.04230000	4.17990000
C	-18.75500000	3.17270000	3.65090000
H	-19.40550000	2.84390000	2.83990000
H	-18.78990000	4.26200000	3.65470000
C	-19.29640000	2.64330000	4.98570000
H	-19.26180000	1.55400000	4.98670000
H	-18.64620000	2.97030000	5.79740000
C	-20.73210000	3.10920000	5.26130000
H	-20.76230000	4.19870000	5.26950000
H	-21.38090000	2.78700000	4.44620000
C	-21.28150000	2.57670000	6.59120000
H	-21.25370000	1.48720000	6.58650000
H	-20.63170000	2.89550000	7.40650000
C	-22.71490000	3.05100000	6.86490000
H	-22.73700000	4.14070000	6.88020000
H	-23.36370000	2.73990000	6.04540000
C	-23.27080000	2.51280000	8.19000000

H	-23.26460000	1.42260000	8.17770000
H	-22.61780000	2.81290000	9.00970000
C	-24.69350000	3.00550000	8.47240000
H	-24.72890000	4.09320000	8.52620000
H	-25.05870000	2.61600000	9.42280000
H	-25.38540000	2.68470000	7.69330000
C	-12.86680000	5.45000000	-5.20820000
H	-13.12820000	6.49240000	-5.39660000
H	-12.27070000	5.45120000	-4.29530000
C	-14.14130000	4.63440000	-4.94700000
H	-14.75810000	4.60320000	-5.84610000
H	-13.87020000	3.60280000	-4.71710000
C	-14.94250000	5.23320000	-3.77990000
H	-15.27030000	6.23790000	-4.05110000
H	-14.28520000	5.35310000	-2.91860000
C	-16.15830000	4.39620000	-3.35870000
H	-16.82040000	4.25140000	-4.21340000
H	-15.82960000	3.40370000	-3.04730000
C	-16.93180000	5.06760000	-2.21330000
H	-17.27080000	6.05270000	-2.53730000
H	-16.25700000	5.24020000	-1.37520000
C	-18.13760000	4.25110000	-1.72840000
H	-18.81170000	4.06750000	-2.56570000
H	-17.80130000	3.27420000	-1.37900000
C	-18.90290000	4.96340000	-0.60330000
H	-19.23290000	5.94260000	-0.95290000
H	-18.22760000	5.14940000	0.23110000
C	-20.11600000	4.16610000	-0.10510000
H	-20.79110000	3.97740000	-0.94040000
H	-19.78820000	3.19070000	0.25620000
C	-20.87890000	4.89420000	1.01050000
H	-20.20740000	5.07460000	1.84890000
H	-21.19560000	5.87540000	0.65430000
C	-22.10310000	4.10980000	1.50130000
H	-21.78730000	3.13050000	1.86230000
H	-22.77530000	3.92930000	0.66190000
C	-22.86560000	4.84170000	2.61420000
H	-22.19890000	5.00930000	3.45910000
H	-23.16880000	5.82820000	2.26090000
C	-24.10130000	4.06650000	3.08930000
H	-23.79740000	3.08150000	3.44510000
H	-24.76830000	3.89850000	2.24310000
C	-24.86630000	4.79570000	4.20180000
H	-24.20540000	4.94900000	5.05410000
H	-25.15790000	5.78800000	3.85520000
C	-26.11220000	4.02510000	4.65710000
H	-25.82170000	3.03440000	5.00700000
H	-26.77690000	3.87060000	3.80660000
C	-26.88050000	4.74520000	5.76930000
H	-26.25990000	4.87370000	6.65510000
H	-27.76280000	4.17630000	6.06360000
H	-27.21290000	5.73240000	5.44700000
O	-5.65920000	6.42860000	-9.17100000
Si	-4.75610000	5.92300000	-10.51340000
C	-5.92380000	5.75220000	-11.97930000
H	-6.33150000	6.71720000	-12.27250000
H	-5.41500000	5.33140000	-12.84530000
H	-6.76270000	5.10230000	-11.73850000
C	-3.87460000	4.28700000	-10.22450000
H	-3.20600000	4.33830000	-9.36670000
H	-4.57500000	3.47630000	-10.04520000
H	-3.27020000	3.99940000	-11.08320000
C	-3.46160000	7.28500000	-10.85720000
C	-2.61140000	7.47910000	-9.59070000
H	-2.10840000	6.55730000	-9.29710000
H	-1.85000000	8.24600000	-9.72770000
H	-3.23260000	7.78420000	-8.74870000
C	-2.56780000	6.85830000	-12.03420000

H	-2.04810000	5.92440000	-11.81810000
H	-3.15340000	6.70220000	-12.94090000
H	-1.81190000	7.61220000	-12.25390000
C	-4.18310000	8.60090000	-11.19410000
H	-4.77720000	8.51140000	-12.10360000
H	-4.85950000	8.89410000	-10.38950000
H	-3.47560000	9.41650000	-11.34370000
O	-5.28190000	3.58610000	-7.56560000
H	-5.12030000	3.23970000	-6.68170000
O	-5.25800000	3.94130000	-5.12180000
H	-4.46860000	4.39010000	-4.81640000
C	1.29160000	7.17470000	-4.53570000
C	-0.55590000	8.97220000	-6.43550000
O	1.30570000	5.96130000	-4.71770000
O	-0.88130000	10.14090000	-6.22380000
N	2.37520000	7.94090000	-4.66410000
N	0.19940000	8.62820000	-7.48560000
H	2.28410000	8.92120000	-4.39730000
H	0.42770000	7.63560000	-7.59130000
C	-0.01660000	7.90590000	-4.21760000
H	0.22740000	8.94780000	-4.02560000
C	-0.64670000	7.40540000	-2.89530000
H	0.12560000	7.05590000	-2.20800000
H	-1.12890000	8.24180000	-2.38960000
C	-2.43970000	7.95700000	-4.99750000
H	-2.57840000	8.91400000	-4.49150000
C	-1.69420000	6.31160000	-3.09390000
H	-1.20480000	5.42410000	-3.50000000
C	-2.82040000	6.80070000	-4.03050000
H	-3.68640000	7.11570000	-3.45420000
C	0.73930000	9.54620000	-8.48130000
H	0.11310000	10.43620000	-8.56560000
H	0.69550000	9.05360000	-9.45130000
C	3.61690000	7.52420000	-5.28960000
H	3.39240000	7.12390000	-6.28050000
H	4.07790000	6.72200000	-4.71080000
C	-0.96470000	7.85260000	-5.45480000
H	-0.85630000	6.89760000	-5.96980000
C	2.19150000	9.93690000	-8.15680000
H	2.79870000	9.03350000	-8.07990000
H	2.22470000	10.41990000	-7.17920000
C	2.79750000	10.87400000	-9.21480000
H	2.71980000	10.40570000	-10.19610000
H	2.20290000	11.78650000	-9.26680000
C	4.26840000	11.23540000	-8.94620000
H	4.35570000	11.70090000	-7.96400000
H	4.86610000	10.32340000	-8.91140000
C	4.84090000	12.18180000	-10.01530000
H	4.74390000	11.71940000	-10.99740000
H	4.24120000	13.09150000	-10.05050000
C	6.31330000	12.55430000	-9.77820000
H	6.91950000	11.64790000	-9.76390000
H	6.41780000	13.01340000	-8.79480000
C	6.85490000	13.51500000	-10.85020000
H	6.74620000	13.05970000	-11.83430000
H	6.24630000	14.41920000	-10.86700000
C	8.32620000	13.89890000	-10.63100000
H	8.93890000	12.99710000	-10.62370000
H	8.43700000	14.35770000	-9.64840000
C	8.85220000	14.86400000	-11.70560000
H	8.73870000	14.40970000	-12.68970000
H	8.24000000	15.76590000	-11.71500000
C	10.32320000	15.25060000	-11.49230000
H	10.93720000	14.34960000	-11.48420000
H	10.43610000	15.71190000	-10.51130000
C	10.84730000	16.21340000	-12.56840000
H	10.73530000	15.75680000	-13.55170000
H	10.23430000	17.11480000	-12.57930000

C	12.31750000	16.59960000	-12.35100000
H	12.42740000	17.06380000	-11.37120000
H	12.93150000	15.69860000	-12.33800000
C	12.84480000	17.55900000	-13.42730000
H	12.73810000	17.09880000	-14.40950000
H	12.23050000	18.45950000	-13.44370000
C	14.31270000	17.94750000	-13.20230000
H	14.41620000	18.41620000	-12.22370000
H	14.92830000	17.04770000	-13.18070000
C	14.84220000	18.90310000	-14.28030000
H	14.75360000	18.43810000	-15.26220000
H	14.22340000	19.79990000	-14.31090000
C	16.30130000	19.30620000	-14.04390000
H	16.42080000	19.80810000	-13.08430000
H	16.64590000	19.99100000	-14.81900000
H	16.95880000	18.43680000	-14.05210000
C	4.54950000	8.73680000	-5.39730000
H	4.87530000	9.02500000	-4.39650000
H	3.98880000	9.58660000	-5.78750000
C	5.77030000	8.51050000	-6.29790000
H	6.34350000	7.65160000	-5.94610000
H	5.43610000	8.27110000	-7.30880000
C	6.66120000	9.75960000	-6.32570000
H	7.05940000	9.93570000	-5.32520000
H	6.05200000	10.63150000	-6.56520000
C	7.81750000	9.67710000	-7.32940000
H	8.43040000	8.79930000	-7.12020000
H	7.42110000	9.54760000	-8.33760000
C	8.68370000	10.94230000	-7.26820000
H	9.09680000	11.04620000	-6.26370000
H	8.05510000	11.81840000	-7.42830000
C	9.82690000	10.95170000	-8.28920000
H	10.45780000	10.07440000	-8.14100000
H	9.41920000	10.87570000	-9.29800000
C	10.67620000	12.22370000	-8.16730000
H	11.07980000	12.29390000	-7.15620000
H	10.03910000	13.09760000	-8.30140000
C	11.82790000	12.27520000	-9.17760000
H	12.46770000	11.40250000	-9.04250000
H	11.42880000	12.21450000	-10.19080000
C	12.66610000	13.55160000	-9.02990000
H	12.02570000	14.42190000	-9.16920000
H	13.05390000	13.61650000	-8.01240000
C	13.83160000	13.61200000	-10.02430000
H	13.44580000	13.54790000	-11.04220000
H	14.47390000	12.74260000	-9.88040000
C	14.66410000	14.89140000	-9.87190000
H	14.02430000	15.75890000	-10.02890000
H	15.03660000	14.96490000	-8.84920000
C	15.84340000	14.94420000	-10.85080000
H	15.47050000	14.86960000	-11.87270000
H	16.48410000	14.07660000	-10.69020000
C	16.67500000	16.22480000	-10.70360000
H	16.03770000	17.09080000	-10.87920000
H	17.03600000	16.31060000	-9.67780000
C	17.86460000	16.26290000	-11.67090000
H	17.50430000	16.17870000	-12.69640000
H	18.50550000	15.39820000	-11.49580000
C	18.69730000	17.54080000	-11.53390000
H	18.09730000	18.42410000	-11.74820000
H	19.53700000	17.53450000	-12.22930000
H	19.10010000	17.64580000	-10.52610000
O	-2.20390000	6.02170000	-1.80270000
Si	-3.31250000	4.80810000	-1.39530000
C	-2.87270000	3.23000000	-2.31780000
H	-1.79390000	3.10530000	-2.38520000
H	-3.28190000	2.34810000	-1.82850000
H	-3.27370000	3.24800000	-3.32810000

C	-5.06790000	5.35770000	-1.80190000
H	-5.30790000	6.29930000	-1.31090000
H	-5.22270000	5.49790000	-2.86810000
H	-5.80290000	4.62400000	-1.47580000
C	-3.17230000	4.54510000	0.49190000
C	-3.47940000	5.87510000	1.20180000
H	-4.49410000	6.21540000	0.99230000
H	-3.38060000	5.78350000	2.28350000
H	-2.79960000	6.66280000	0.87310000
C	-4.17620000	3.47040000	0.94480000
H	-5.20200000	3.76120000	0.71610000
H	-3.98920000	2.51520000	0.45380000
H	-4.11730000	3.30160000	2.02030000
C	-1.74110000	4.09830000	0.82980000
H	-1.48870000	3.16170000	0.33140000
H	-1.01330000	4.84660000	0.51300000
H	-1.61130000	3.94640000	1.90150000
O	-3.19640000	5.76340000	-4.90330000
H	-3.34720000	6.30890000	-5.69050000
O	-3.35860000	7.88220000	-6.09390000
H	-4.11880000	8.43080000	-5.88100000

4-beta tetramer

C	-11.27900000	0.33430000	-9.31330000
C	-9.09740000	0.76340000	-7.29770000
O	-11.42450000	-0.77520000	-9.82870000
O	-9.33780000	1.77910000	-6.64220000
N	-12.16680000	0.86220000	-8.46370000
N	-9.14810000	-0.45590000	-6.74900000
H	-11.94200000	1.76630000	-8.07240000
H	-8.94590000	-1.24460000	-7.36860000
C	-10.05650000	1.20600000	-9.63450000
H	-10.33280000	2.23460000	-9.39330000
C	-9.72390000	1.15330000	-11.13740000
H	-9.33000000	0.16880000	-11.39780000
H	-10.62380000	1.27580000	-11.73670000
C	-7.66620000	1.86750000	-9.11280000
H	-7.91670000	2.81930000	-8.64760000
C	-8.68270000	2.22410000	-11.49150000
H	-8.39070000	2.12300000	-12.53440000
C	-7.41420000	2.09730000	-10.62450000
H	-6.79550000	2.98410000	-10.75850000
C	-9.49230000	-0.74350000	-5.36200000
H	-8.85760000	-0.14950000	-4.70230000
H	-9.26030000	-1.79010000	-5.16420000
C	-13.37200000	0.19150000	-8.00800000
H	-13.10710000	-0.79110000	-7.61550000
H	-14.03470000	0.02740000	-8.85930000
C	-8.79150000	0.84770000	-8.80930000
H	-8.44150000	-0.12300000	-9.14920000
C	-10.98110000	-0.46360000	-5.08480000
H	-11.58770000	-1.04430000	-5.77920000
H	-11.19670000	0.58420000	-5.29460000
C	-11.42000000	-0.76830000	-3.64650000
H	-11.29470000	-1.83220000	-3.44450000
H	-10.77400000	-0.24260000	-2.94320000
C	-12.88010000	-0.35480000	-3.40800000
H	-12.98320000	0.71830000	-3.57650000
H	-13.51450000	-0.84380000	-4.14750000
C	-13.39290000	-0.69770000	-2.00310000
H	-13.30430000	-1.77150000	-1.83670000
H	-12.76370000	-0.21860000	-1.25280000
C	-14.85320000	-0.26520000	-1.80530000
H	-15.46930000	-0.71750000	-2.58260000
H	-14.93440000	0.81470000	-1.93730000

C	-15.41310000	-0.65660000	-0.43130000
H	-15.33610000	-1.73650000	-0.30290000
H	-14.80120000	-0.21210000	0.35370000
C	-16.87560000	-0.22460000	-0.25250000
H	-17.47900000	-0.65700000	-1.05080000
H	-16.95220000	0.85820000	-0.35930000
C	-17.45540000	-0.64890000	1.10340000
H	-17.38510000	-1.73220000	1.20480000
H	-16.84930000	-0.22870000	1.90610000
C	-18.91700000	-0.21460000	1.28150000
H	-19.51880000	-0.63140000	0.47400000
H	-18.98880000	0.87010000	1.19300000
C	-19.50030000	-0.65920000	2.62940000
H	-19.43590000	-1.74440000	2.71230000
H	-18.89190000	-0.25570000	3.43880000
C	-20.95880000	-0.21980000	2.81700000
H	-21.02470000	0.86610000	2.74200000
H	-21.56590000	-0.62330000	2.00650000
C	-21.53830000	-0.67710000	4.16220000
H	-21.48260000	-1.76380000	4.23120000
H	-20.92200000	-0.28840000	4.97300000
C	-22.99130000	-0.22710000	4.36410000
H	-23.04690000	0.86000000	4.29900000
H	-23.60790000	-0.61710000	3.55420000
C	-23.56540000	-0.68930000	5.71020000
H	-23.53190000	-1.77760000	5.77110000
H	-22.93900000	-0.31890000	6.52170000
C	-25.00600000	-0.21680000	5.92950000
H	-25.07460000	0.87020000	5.89450000
H	-25.37840000	-0.54040000	6.90170000
H	-25.67290000	-0.62040000	5.16810000
C	-14.08110000	1.01700000	-6.92520000
H	-14.35750000	1.99280000	-7.32770000
H	-13.39680000	1.20530000	-6.09730000
C	-15.33350000	0.30590000	-6.39850000
H	-16.01900000	0.12340000	-7.22730000
H	-15.05470000	-0.67380000	-6.00990000
C	-16.06060000	1.09480000	-5.30210000
H	-16.34050000	2.07830000	-5.68220000
H	-15.38490000	1.26780000	-4.46370000
C	-17.31330000	0.35620000	-4.81290000
H	-17.97990000	0.17740000	-5.65760000
H	-17.02960000	-0.62540000	-4.43290000
C	-18.07410000	1.12030000	-3.72350000
H	-18.35410000	2.10530000	-4.09880000
H	-17.41750000	1.28850000	-2.86940000
C	-19.33260000	0.36880000	-3.26950000
H	-19.98200000	0.19660000	-4.12880000
H	-19.05100000	-0.61520000	-2.89480000
C	-20.11510000	1.12040000	-2.18660000
H	-20.39560000	2.10560000	-2.56080000
H	-19.47010000	1.28780000	-1.32360000
C	-21.37660000	0.36500000	-1.74810000
H	-22.02070000	0.19970000	-2.61270000
H	-21.09720000	-0.62130000	-1.37840000
C	-22.16220000	1.11150000	-0.66330000
H	-21.51780000	1.27510000	0.20100000
H	-22.44130000	2.09860000	-1.03320000
C	-23.42470000	0.35730000	-0.22600000
H	-23.14730000	-0.63230000	0.13550000
H	-24.07310000	0.20090000	-1.08900000
C	-24.20120000	1.09970000	0.86870000
H	-23.55050000	1.25570000	1.72970000
H	-24.47920000	2.09030000	0.50710000
C	-25.46340000	0.34790000	1.31150000
H	-25.18730000	-0.64630000	1.66140000
H	-26.12050000	0.20200000	0.45320000
C	-26.22540000	1.08480000	2.42060000

H	-25.56530000	1.23110000	3.27610000
H	-26.50240000	2.07940000	2.06940000
C	-27.48630000	0.33500000	2.87050000
H	-27.21580000	-0.66210000	3.21800000
H	-28.15230000	0.19410000	2.01830000
C	-28.24120000	1.06680000	3.98570000
H	-27.61610000	1.19500000	4.86980000
H	-29.12770000	0.50770000	4.28620000
H	-28.56790000	2.05470000	3.66000000
O	-9.23770000	3.51570000	-11.31580000
Si	-10.08760000	4.39600000	-12.48530000
C	-11.64300000	3.46000000	-12.99870000
H	-12.27950000	4.07360000	-13.63420000
H	-11.41070000	2.55510000	-13.55430000
H	-12.23240000	3.17130000	-12.12950000
C	-8.97190000	4.67410000	-13.98100000
H	-8.11990000	5.30180000	-13.72700000
H	-8.58160000	3.73920000	-14.37850000
H	-9.50960000	5.17230000	-14.78620000
C	-10.60590000	6.06350000	-11.71330000
C	-11.67510000	5.80530000	-10.63770000
H	-11.32730000	5.07840000	-9.90440000
H	-11.93260000	6.71800000	-10.10030000
H	-12.59310000	5.40940000	-11.07230000
C	-9.37110000	6.71580000	-11.07430000
H	-8.90280000	6.03950000	-10.35810000
H	-8.62250000	6.96600000	-11.82460000
H	-9.62730000	7.63380000	-10.54550000
C	-11.18030000	6.98580000	-12.80240000
H	-10.44230000	7.19500000	-13.57750000
H	-12.04760000	6.53810000	-13.28850000
H	-11.49520000	7.94250000	-12.38500000
O	-6.63850000	0.98870000	-11.02220000
H	-6.39650000	0.64150000	-10.15850000
O	-6.42750000	1.39170000	-8.59120000
H	-5.94300000	2.16560000	-8.27940000
C	-9.64340000	5.27170000	-7.04680000
C	-8.11140000	4.38940000	-4.42800000
O	-9.69950000	4.53380000	-8.02630000
O	-7.92990000	5.21050000	-3.52730000
N	-10.71170000	5.60200000	-6.31740000
N	-8.81420000	3.26510000	-4.24940000
H	-10.56070000	6.26140000	-5.55240000
H	-8.95070000	2.66880000	-5.06730000
C	-8.29650000	5.78950000	-6.53120000
H	-8.50460000	6.55740000	-5.78800000
C	-7.46660000	6.50820000	-7.62850000
H	-8.12200000	6.95430000	-8.37640000
H	-6.94320000	7.34990000	-7.17250000
C	-6.02690000	4.92230000	-5.82340000
H	-5.87210000	5.89350000	-5.34810000
C	-6.42650000	5.60490000	-8.31140000
H	-6.94740000	4.85230000	-8.90350000
C	-5.48070000	4.95110000	-7.27350000
H	-4.51450000	5.45580000	-7.28270000
C	-9.36340000	2.81120000	-2.98230000
H	-8.70100000	3.09620000	-2.16230000
H	-9.38280000	1.72200000	-2.99850000
C	-12.00150000	4.93560000	-6.36810000
H	-11.84430000	3.85870000	-6.27820000
H	-12.48090000	5.11750000	-7.33120000
C	-7.53820000	4.62260000	-5.83550000
H	-7.67550000	3.70460000	-6.40720000
C	-10.77700000	3.36330000	-2.74920000
H	-11.42330000	3.07030000	-3.57790000
H	-10.74420000	4.45400000	-2.74630000
C	-11.37130000	2.86370000	-1.42620000
H	-11.36120000	1.77350000	-1.41820000

H	-10.73080000	3.18210000	-0.60260000
C	-12.80280000	3.35840000	-1.18060000
H	-12.81660000	4.44880000	-1.18230000
H	-13.44570000	3.03650000	-2.00120000
C	-13.36650000	2.83790000	0.14890000
H	-13.33670000	1.74820000	0.15240000
H	-12.72210000	3.16180000	0.96670000
C	-14.80290000	3.30560000	0.41640000
H	-15.45040000	2.97360000	-0.39600000
H	-14.83550000	4.39530000	0.41540000
C	-15.34550000	2.77930000	1.75280000
H	-15.30820000	1.69020000	1.75660000
H	-14.69530000	3.10840000	2.56360000
C	-16.78190000	3.23980000	2.03280000
H	-17.43310000	2.90770000	1.22370000
H	-16.81810000	4.32920000	2.03400000
C	-17.31860000	2.71200000	3.37070000
H	-17.28040000	1.62280000	3.37430000
H	-16.66720000	3.04230000	4.17990000
C	-18.75500000	3.17270000	3.65090000
H	-19.40550000	2.84390000	2.83990000
H	-18.78990000	4.26200000	3.65470000
C	-19.29640000	2.64330000	4.98570000
H	-19.26180000	1.55400000	4.98670000
H	-18.64620000	2.97030000	5.79740000
C	-20.73210000	3.10920000	5.26130000
H	-20.76230000	4.19870000	5.26950000
H	-21.38090000	2.78700000	4.44620000
C	-21.28150000	2.57670000	6.59120000
H	-21.25370000	1.48720000	6.58650000
H	-20.63170000	2.89550000	7.40650000
C	-22.71490000	3.05100000	6.86490000
H	-22.73700000	4.14070000	6.88020000
H	-23.36370000	2.73990000	6.04540000
C	-23.27080000	2.51280000	8.19000000
H	-23.26460000	1.42260000	8.17770000
H	-22.61780000	2.81290000	9.00970000
C	-24.69350000	3.00550000	8.47240000
H	-24.72890000	4.09320000	8.52620000
H	-25.05870000	2.61600000	9.42280000
H	-25.38540000	2.68470000	7.69330000
C	-12.86680000	5.45000000	-5.20820000
H	-13.12820000	6.49240000	-5.39660000
H	-12.27070000	5.45120000	-4.29530000
C	-14.14130000	4.63440000	-4.94700000
H	-14.75810000	4.60320000	-5.84610000
H	-13.87020000	3.60280000	-4.71710000
C	-14.94250000	5.23320000	-3.77990000
H	-15.27030000	6.23790000	-4.05110000
H	-14.28520000	5.35310000	-2.91860000
C	-16.15830000	4.39620000	-3.35870000
H	-16.82040000	4.25140000	-4.21340000
H	-15.82960000	3.40370000	-3.04730000
C	-16.93180000	5.06760000	-2.21330000
H	-17.27080000	6.05270000	-2.53730000
H	-16.25700000	5.24020000	-1.37520000
C	-18.13760000	4.25110000	-1.72840000
H	-18.81170000	4.06750000	-2.56570000
H	-17.80130000	3.27420000	-1.37900000
C	-18.90290000	4.96340000	-0.60330000
H	-19.23290000	5.94260000	-0.95290000
H	-18.22760000	5.14940000	0.23110000
C	-20.11600000	4.16610000	-0.10510000
H	-20.79110000	3.97740000	-0.94040000
H	-19.78820000	3.19070000	0.25620000
C	-20.87890000	4.89420000	1.01050000
H	-20.20740000	5.07460000	1.84890000
H	-21.19560000	5.87540000	0.65430000

C	-22.10310000	4.10980000	1.50130000
H	-21.78730000	3.13050000	1.86230000
H	-22.77530000	3.92930000	0.66190000
C	-22.86560000	4.84170000	2.61420000
H	-22.19890000	5.00930000	3.45910000
H	-23.16880000	5.82820000	2.26090000
C	-24.10130000	4.06650000	3.08930000
H	-23.79740000	3.08150000	3.44510000
H	-24.76830000	3.89850000	2.24310000
C	-24.86630000	4.79570000	4.20180000
H	-24.20540000	4.94900000	5.05410000
H	-25.15790000	5.78800000	3.85520000
C	-26.11220000	4.02510000	4.65710000
H	-25.82170000	3.03440000	5.00700000
H	-26.77690000	3.87060000	3.80660000
C	-26.88050000	4.74520000	5.76930000
H	-26.25990000	4.87370000	6.65510000
H	-27.76280000	4.17630000	6.06360000
H	-27.21290000	5.73240000	5.44700000
O	-5.65920000	6.42860000	-9.17100000
Si	-4.75610000	5.92300000	-10.51340000
C	-5.92380000	5.75220000	-11.97930000
H	-6.33150000	6.71720000	-12.27250000
H	-5.41500000	5.33140000	-12.84530000
H	-6.76270000	5.10230000	-11.73850000
C	-3.87460000	4.28700000	-10.22450000
H	-3.20600000	4.33830000	-9.36670000
H	-4.57500000	3.47630000	-10.04520000
H	-3.27020000	3.99940000	-11.08320000
C	-3.46160000	7.28500000	-10.85720000
C	-2.61140000	7.47910000	-9.59070000
H	-2.10840000	6.55730000	-9.29710000
H	-1.85000000	8.24600000	-9.72770000
H	-3.23260000	7.78420000	-8.74870000
C	-2.56780000	6.85830000	-12.03420000
H	-2.04810000	5.92440000	-11.81810000
H	-3.15340000	6.70220000	-12.94090000
H	-1.81190000	7.61220000	-12.25390000
C	-4.18310000	8.60090000	-11.19410000
H	-4.77720000	8.51140000	-12.10360000
H	-4.85950000	8.89410000	-10.38950000
H	-3.47560000	9.41650000	-11.34370000
O	-5.28190000	3.58610000	-7.56560000
H	-5.12030000	3.23970000	-6.68170000
O	-5.25800000	3.94130000	-5.12180000
H	-4.46860000	4.39010000	-4.81640000
C	2.49770000	2.83820000	-8.21540000
C	0.41900000	4.95400000	-8.40160000
O	2.35720000	2.01990000	-9.12130000
O	0.80920000	5.93750000	-7.77130000
N	3.55320000	3.64970000	-8.10700000
N	0.32120000	4.96270000	-9.73660000
H	3.54470000	4.30470000	-7.33880000
H	-0.03720000	4.11350000	-10.18210000
C	1.42390000	2.98530000	-7.13920000
H	1.82340000	3.63800000	-6.35950000
C	1.13260000	1.61510000	-6.50170000
H	0.67040000	0.95360000	-7.23720000
H	2.05780000	1.12790000	-6.19610000
C	-0.85540000	3.81880000	-6.48770000
H	-0.45350000	4.56960000	-5.80950000
C	0.19040000	1.76780000	-5.30390000
H	-0.06630000	0.78150000	-4.92160000
C	-1.09470000	2.52140000	-5.68700000
H	-1.65320000	2.75020000	-4.78300000
C	0.71830000	6.05980000	-10.60890000
H	0.17930000	6.96150000	-10.32110000
H	0.40430000	5.81620000	-11.62340000

C	4.66070000	3.71020000	-9.04320000
H	4.27870000	3.70730000	-10.06530000
H	5.27360000	2.81510000	-8.92770000
C	0.11660000	3.62680000	-7.67720000
H	-0.33860000	2.93860000	-8.39050000
C	2.23870000	6.29620000	-10.57270000
H	2.75080000	5.37210000	-10.83880000
H	2.54840000	6.53370000	-9.55490000
C	2.70680000	7.42720000	-11.49580000
H	2.49790000	7.16510000	-12.53270000
H	2.13610000	8.33190000	-11.28730000
C	4.20410000	7.71280000	-11.31920000
H	4.39550000	8.01330000	-10.28790000
H	4.76600000	6.79290000	-11.48110000
C	4.72740000	8.79570000	-12.26950000
H	4.56200000	8.48580000	-13.30150000
H	4.15600000	9.71350000	-12.13120000
C	6.21990000	9.07720000	-12.05180000
H	6.78090000	8.14860000	-12.15810000
H	6.38080000	9.42040000	-11.02880000
C	6.77450000	10.11480000	-13.03490000
H	6.62540000	9.76360000	-14.05620000
H	6.20730000	11.04120000	-12.94570000
C	8.26510000	10.39750000	-12.80600000
H	8.82590000	9.46600000	-12.88320000
H	8.41520000	10.76630000	-11.79060000
C	8.82780000	11.41080000	-13.81010000
H	8.68650000	11.03410000	-14.82350000
H	8.25810000	12.33800000	-13.74780000
C	10.31560000	11.70500000	-13.57930000
H	10.88230000	10.77570000	-13.63890000
H	10.45880000	12.09120000	-12.56950000
C	10.87480000	12.70670000	-14.59720000
H	10.74000000	12.31330000	-15.60510000
H	10.29760000	13.63030000	-14.55080000
C	12.35920000	13.01750000	-14.36640000
H	12.49550000	13.41560000	-13.36040000
H	12.93520000	12.09340000	-14.41440000
C	12.91120000	14.01510000	-15.39280000
H	12.78430000	13.61040000	-16.39710000
H	12.32380000	14.93270000	-15.35720000
C	14.39090000	14.34510000	-15.15880000
H	14.51720000	14.75010000	-14.15430000
H	14.97820000	13.42770000	-15.19810000
C	14.93640000	15.34480000	-16.18710000
H	14.83140000	14.93460000	-17.19200000
H	14.33690000	16.25490000	-16.16480000
C	16.40530000	15.70130000	-15.93910000
H	16.54520000	16.12780000	-14.94620000
H	16.75690000	16.43330000	-16.66640000
H	17.04320000	14.82150000	-16.01950000
C	5.49160000	4.97560000	-8.79220000
H	5.85900000	4.97550000	-7.76480000
H	4.85570000	5.85610000	-8.89320000
C	6.67820000	5.09280000	-9.75440000
H	7.31950000	4.21700000	-9.64490000
H	6.31450000	5.07960000	-10.78230000
C	7.50340000	6.36530000	-9.52110000
H	7.85500000	6.39050000	-8.48860000
H	6.86900000	7.24290000	-9.65150000
C	8.70330000	6.44970000	-10.47200000
H	9.33590000	5.57130000	-10.33520000
H	8.35130000	6.41480000	-11.50320000
C	9.54070000	7.71720000	-10.26200000
H	9.88690000	7.75930000	-9.22840000
H	8.91480000	8.59730000	-10.41370000
C	10.74680000	7.77050000	-11.20880000
H	11.37160000	6.89030000	-11.05050000

H	10.39870000	7.71670000	-12.24040000
C	11.59310000	9.03570000	-11.02220000
H	11.94190000	9.09070000	-9.99030000
H	10.97060000	9.91600000	-11.18550000
C	12.79860000	9.07430000	-11.97100000
H	13.42320000	8.19620000	-11.80110000
H	12.44940000	9.00780000	-13.00130000
C	13.64410000	10.34290000	-11.80060000
H	13.01850000	11.21980000	-11.97030000
H	13.99560000	10.40840000	-10.77040000
C	14.84760000	10.37990000	-12.75200000
H	14.49710000	10.30260000	-13.78110000
H	15.47810000	9.50740000	-12.57510000
C	15.68410000	11.65630000	-12.59360000
H	15.05150000	12.52690000	-12.76940000
H	16.03630000	11.73280000	-11.56430000
C	16.88670000	11.69740000	-13.54620000
H	16.53670000	11.60900000	-14.57460000
H	17.52540000	10.83220000	-13.36290000
C	17.71100000	12.98330000	-13.39730000
H	17.06980000	13.84630000	-13.57910000
H	18.06180000	13.07000000	-12.36830000
C	18.91380000	13.03070000	-14.34980000
H	18.57060000	12.94160000	-15.38080000
H	19.56100000	12.17220000	-14.16590000
C	19.73120000	14.31910000	-14.20390000
H	19.12470000	15.19990000	-14.41630000
H	20.57440000	14.32430000	-14.89500000
H	20.13040000	14.42220000	-13.19460000
O	0.84200000	2.48350000	-4.27350000
Si	1.60710000	1.77810000	-2.94540000
C	2.92330000	0.57810000	-3.56410000
H	3.48260000	0.14300000	-2.73720000
H	2.48140000	-0.24190000	-4.12830000
H	3.63820000	1.07890000	-4.21520000
C	0.32740000	0.85020000	-1.91650000
H	-0.39770000	1.52950000	-1.47380000
H	-0.22410000	0.12820000	-2.51710000
H	0.79830000	0.30660000	-1.09900000
C	2.41040000	3.17830000	-1.93030000
C	3.48150000	3.86860000	-2.79210000
H	3.05430000	4.24030000	-3.72450000
H	3.92500000	4.71850000	-2.27350000
H	4.28770000	3.18330000	-3.05440000
C	1.32150000	4.19340000	-1.54890000
H	0.83360000	4.59480000	-2.43730000
H	0.54790000	3.73250000	-0.93720000
H	1.73030000	5.03480000	-0.98950000
C	3.05250000	2.59420000	-0.66070000
H	2.31150000	2.09890000	-0.03210000
H	3.82010000	1.85900000	-0.90420000
H	3.52270000	3.37340000	-0.06020000
O	-1.92160000	1.72330000	-6.49820000
H	-2.36800000	2.36370000	-7.04840000
O	-2.13780000	4.23950000	-6.92560000
H	-2.56150000	4.62520000	-6.16090000
C	1.29160000	7.17470000	-4.53570000
C	-0.55590000	8.97220000	-6.43550000
O	1.30570000	5.96130000	-4.71770000
O	-0.88130000	10.14090000	-6.22380000
N	2.37520000	7.94090000	-4.66410000
N	0.19940000	8.62820000	-7.48560000
H	2.28410000	8.92120000	-4.39730000
H	0.42770000	7.63560000	-7.59130000
C	-0.01660000	7.90590000	-4.21760000
H	0.22740000	8.94780000	-4.02560000
C	-0.64670000	7.40540000	-2.89530000
H	0.12560000	7.05590000	-2.20800000

H	-1.12890000	8.24180000	-2.38960000
C	-2.43970000	7.95700000	-4.99750000
H	-2.57840000	8.91400000	-4.49150000
C	-1.69420000	6.31160000	-3.09390000
H	-1.20480000	5.42410000	-3.50000000
C	-2.82040000	6.80070000	-4.03050000
H	-3.68640000	7.11570000	-3.45420000
C	0.73930000	9.54620000	-8.48130000
H	0.11310000	10.43620000	-8.56560000
H	0.69550000	9.05360000	-9.45130000
C	3.61690000	7.52420000	-5.28960000
H	3.39240000	7.12390000	-6.28050000
H	4.07790000	6.72200000	-4.71080000
C	-0.96470000	7.85260000	-5.45480000
H	-0.85630000	6.89760000	-5.96980000
C	2.19150000	9.93690000	-8.15680000
H	2.79870000	9.03350000	-8.07990000
H	2.22470000	10.41990000	-7.17920000
C	2.79750000	10.87400000	-9.21480000
H	2.71980000	10.40570000	-10.19610000
H	2.20290000	11.78650000	-9.26680000
C	4.26840000	11.23540000	-8.94620000
H	4.35570000	11.70090000	-7.96400000
H	4.86610000	10.32340000	-8.91140000
C	4.84090000	12.18180000	-10.01530000
H	4.74390000	11.71940000	-10.99740000
H	4.24120000	13.09150000	-10.05050000
C	6.31330000	12.55430000	-9.77820000
H	6.91950000	11.64790000	-9.76390000
H	6.41780000	13.01340000	-8.79480000
C	6.85490000	13.51500000	-10.85020000
H	6.74620000	13.05970000	-11.83430000
H	6.24630000	14.41920000	-10.86700000
C	8.32620000	13.89890000	-10.63100000
H	8.93890000	12.99710000	-10.62370000
H	8.43700000	14.35770000	-9.64840000
C	8.85220000	14.86400000	-11.70560000
H	8.73870000	14.40970000	-12.68970000
H	8.24000000	15.76590000	-11.71500000
C	10.32320000	15.25060000	-11.49230000
H	10.93720000	14.34960000	-11.48420000
H	10.43610000	15.71190000	-10.51130000
C	10.84730000	16.21340000	-12.56840000
H	10.73530000	15.75680000	-13.55170000
H	10.23430000	17.11480000	-12.57930000
C	12.31750000	16.59960000	-12.35100000
H	12.42740000	17.06380000	-11.37120000
H	12.93150000	15.69860000	-12.33800000
C	12.84480000	17.55900000	-13.42730000
H	12.73810000	17.09880000	-14.40950000
H	12.23050000	18.45950000	-13.44370000
C	14.31270000	17.94750000	-13.20230000
H	14.41620000	18.41620000	-12.22370000
H	14.92830000	17.04770000	-13.18070000
C	14.84220000	18.90310000	-14.28030000
H	14.75360000	18.43810000	-15.26220000
H	14.22340000	19.79990000	-14.31090000
C	16.30130000	19.30620000	-14.04390000
H	16.42080000	19.80810000	-13.08430000
H	16.64590000	19.99100000	-14.81900000
H	16.95880000	18.43680000	-14.05210000
C	4.54950000	8.73680000	-5.39730000
H	4.87530000	9.02500000	-4.39650000
H	3.98880000	9.58660000	-5.78750000
C	5.77030000	8.51050000	-6.29790000
H	6.34350000	7.65160000	-5.94610000
H	5.43610000	8.27110000	-7.30880000
C	6.66120000	9.75960000	-6.32570000

H	7.05940000	9.93570000	-5.32520000
H	6.05200000	10.63150000	-6.56520000
C	7.81750000	9.67710000	-7.32940000
H	8.43040000	8.79930000	-7.12020000
H	7.42110000	9.54760000	-8.33760000
C	8.68370000	10.94230000	-7.26820000
H	9.09680000	11.04620000	-6.26370000
H	8.05510000	11.81840000	-7.42830000
C	9.82690000	10.95170000	-8.28920000
H	10.45780000	10.07440000	-8.14100000
H	9.41920000	10.87570000	-9.29800000
C	10.67620000	12.22370000	-8.16730000
H	11.07980000	12.29390000	-7.15620000
H	10.03910000	13.09760000	-8.30140000
C	11.82790000	12.27520000	-9.17760000
H	12.46770000	11.40250000	-9.04250000
H	11.42880000	12.21450000	-10.19080000
C	12.66610000	13.55160000	-9.02990000
H	12.02570000	14.42190000	-9.16920000
H	13.05390000	13.61650000	-8.01240000
C	13.83160000	13.61200000	-10.02430000
H	13.44580000	13.54790000	-11.04220000
H	14.47390000	12.74260000	-9.88040000
C	14.66410000	14.89140000	-9.87190000
H	14.02430000	15.75890000	-10.02890000
H	15.03660000	14.96490000	-8.84920000
C	15.84340000	14.94420000	-10.85080000
H	15.47050000	14.86960000	-11.87270000
H	16.48410000	14.07660000	-10.69020000
C	16.67500000	16.22480000	-10.70360000
H	16.03770000	17.09080000	-10.87920000
H	17.03600000	16.31060000	-9.67780000
C	17.86460000	16.26290000	-11.67090000
H	17.50430000	16.17870000	-12.69640000
H	18.50550000	15.39820000	-11.49580000
C	18.69730000	17.54080000	-11.53390000
H	18.09730000	18.42410000	-11.74820000
H	19.53700000	17.53450000	-12.22930000
H	19.10010000	17.64580000	-10.52610000
O	-2.20390000	6.02170000	-1.80270000
Si	-3.31250000	4.80810000	-1.39530000
C	-2.87270000	3.23000000	-2.31780000
H	-1.79390000	3.10530000	-2.38520000
H	-3.28190000	2.34810000	-1.82850000
H	-3.27370000	3.24800000	-3.32810000
C	-5.06790000	5.35770000	-1.80190000
H	-5.30790000	6.29930000	-1.31090000
H	-5.22270000	5.49790000	-2.86810000
H	-5.80290000	4.62400000	-1.47580000
C	-3.17230000	4.54510000	0.49190000
C	-3.47940000	5.87510000	1.20180000
H	-4.49410000	6.21540000	0.99230000
H	-3.38060000	5.78350000	2.28350000
H	-2.79960000	6.66280000	0.87310000
C	-4.17620000	3.47040000	0.94480000
H	-5.20200000	3.76120000	0.71610000
H	-3.98920000	2.51520000	0.45380000
H	-4.11730000	3.30160000	2.02030000
C	-1.74110000	4.09830000	0.82980000
H	-1.48870000	3.16170000	0.33140000
H	-1.01330000	4.84660000	0.51300000
H	-1.61130000	3.94640000	1.90150000
O	-3.19640000	5.76340000	-4.90330000
H	-3.34720000	6.30890000	-5.69050000
O	-3.35860000	7.88220000	-6.09390000
H	-4.11880000	8.43080000	-5.88100000

4-beta hexamer

C	-10.75280000	-4.22120000	-11.09300000
C	-8.59130000	-3.57540000	-9.14680000
O	-10.74840000	-5.44700000	-11.19790000
O	-8.67300000	-2.46090000	-8.63620000
N	-11.79440000	-3.54270000	-10.60210000
N	-8.85500000	-4.68800000	-8.44980000
H	-11.69380000	-2.53760000	-10.46240000
H	-8.81010000	-5.56580000	-8.94830000
C	-9.51690000	-3.41690000	-11.51380000
H	-9.74880000	-2.35530000	-11.40920000
C	-9.21930000	-3.70450000	-12.99820000
H	-8.96970000	-4.76020000	-13.12160000
H	-10.10510000	-3.53090000	-13.61050000
C	-7.08090000	-2.84490000	-11.10230000
H	-7.29580000	-1.80380000	-10.85190000
C	-8.05460000	-2.86150000	-13.52400000
H	-7.77670000	-3.24470000	-14.50670000
C	-6.80960000	-2.94280000	-12.62300000
H	-6.10460000	-2.16560000	-12.91620000
C	-9.35360000	-4.70810000	-7.08020000
H	-8.75230000	-4.04230000	-6.45790000
H	-9.21910000	-5.71470000	-6.68340000
C	-13.04850000	-4.17040000	-10.21690000
H	-12.85050000	-5.11780000	-9.71160000
H	-13.61390000	-4.40680000	-11.11950000
C	-8.26650000	-3.73960000	-10.64520000
H	-7.99130000	-4.78150000	-10.81690000
C	-10.84060000	-4.30940000	-7.02410000
H	-11.40970000	-4.94820000	-7.70060000
H	-10.96130000	-3.28940000	-7.39350000
C	-11.43710000	-4.40830000	-5.61410000
H	-11.33360000	-5.43240000	-5.25290000
H	-10.86450000	-3.78070000	-4.93080000
C	-12.91760000	-3.99850000	-5.57250000
H	-13.01970000	-2.95840000	-5.88370000
H	-13.47790000	-4.59220000	-6.29600000
C	-13.53750000	-4.18370000	-4.18150000
H	-13.44730000	-5.23030000	-3.88680000
H	-12.97010000	-3.61020000	-3.44850000
C	-15.01410000	-3.76650000	-4.12470000
H	-15.57600000	-4.31530000	-4.88150000
H	-15.10880000	-2.70910000	-4.37410000
C	-15.63270000	-4.02970000	-2.74550000
H	-15.54140000	-5.09070000	-2.50910000
H	-15.06330000	-3.49720000	-1.98350000
C	-17.10860000	-3.61790000	-2.65950000
H	-17.67440000	-4.13030000	-3.43830000
H	-17.20460000	-2.54970000	-2.85660000
C	-17.71670000	-3.94760000	-1.28980000
H	-17.62180000	-5.01830000	-1.10330000
H	-17.14270000	-3.44940000	-0.50830000
C	-19.19220000	-3.54290000	-1.17540000
H	-19.76370000	-4.02850000	-1.96690000
H	-19.29050000	-2.46880000	-1.33420000
C	-19.78930000	-3.91880000	0.18730000
H	-19.69710000	-4.99550000	0.33670000
H	-19.20710000	-3.44880000	0.98020000
C	-21.26190000	-3.51110000	0.32570000
H	-21.35540000	-2.43320000	0.19340000
H	-21.84260000	-3.97430000	-0.47260000
C	-21.85140000	-3.91380000	1.68420000
H	-21.76920000	-4.99440000	1.80820000
H	-21.25870000	-3.46740000	2.48300000
C	-23.31840000	-3.49290000	1.84160000
H	-23.40050000	-2.41210000	1.72600000

H	-23.91070000	-3.93680000	1.04080000
C	-23.90310000	-3.90660000	3.19880000
H	-23.83830000	-4.98960000	3.31120000
H	-23.30330000	-3.47850000	4.00220000
C	-25.36100000	-3.46790000	3.36980000
H	-25.45700000	-2.38480000	3.30230000
H	-25.74800000	-3.77280000	4.34230000
H	-25.99810000	-3.91130000	2.60440000
C	-13.86330000	-3.25620000	-9.29640000
H	-14.07850000	-2.31690000	-9.80730000
H	-13.26530000	-3.00620000	-8.42030000
C	-15.17250000	-3.92080000	-8.85310000
H	-15.77480000	-4.15550000	-9.73210000
H	-14.94610000	-4.87460000	-8.37410000
C	-15.98990000	-3.05280000	-7.88900000
H	-16.23940000	-2.10610000	-8.36980000
H	-15.38220000	-2.81000000	-7.01740000
C	-17.27470000	-3.75850000	-7.43620000
H	-17.88440000	-3.99740000	-8.30880000
H	-17.01820000	-4.71160000	-6.97130000
C	-18.10070000	-2.92140000	-6.45160000
H	-18.37690000	-1.97490000	-6.91810000
H	-17.48890000	-2.67350000	-5.58440000
C	-19.36520000	-3.65900000	-5.99140000
H	-19.97760000	-3.90640000	-6.85970000
H	-19.08330000	-4.60860000	-5.53410000
C	-20.19980000	-2.84410000	-4.99510000
H	-20.49690000	-1.90020000	-5.45380000
H	-19.58580000	-2.58920000	-4.13150000
C	-21.44790000	-3.60490000	-4.52700000
H	-22.06340000	-3.85950000	-5.39100000
H	-21.14670000	-4.55100000	-4.07470000
C	-22.28610000	-2.80460000	-3.52180000
H	-21.66940000	-2.54560000	-2.66130000
H	-22.59640000	-1.86220000	-3.97480000
C	-23.52340000	-3.57870000	-3.04690000
H	-23.21060000	-4.52210000	-2.59720000
H	-24.14180000	-3.83780000	-3.90740000
C	-24.36200000	-2.78540000	-2.03590000
H	-23.74390000	-2.52730000	-1.17620000
H	-24.67650000	-1.84230000	-2.48470000
C	-25.59630000	-3.56350000	-1.55980000
H	-25.28050000	-4.50630000	-1.11070000
H	-26.21490000	-3.82360000	-2.41990000
C	-26.43580000	-2.77200000	-0.54790000
H	-25.81980000	-2.51990000	0.31530000
H	-26.74500000	-1.82550000	-0.99340000
C	-27.67520000	-3.54710000	-0.08040000
H	-27.36890000	-4.49240000	0.36990000
H	-28.29420000	-3.80270000	-0.94160000
C	-28.51610000	-2.75640000	0.92750000
H	-27.94230000	-2.52620000	1.82470000
H	-29.39390000	-3.32600000	1.23390000
H	-28.86370000	-1.81530000	0.50050000
O	-8.48180000	-1.51980000	-13.67250000
Si	-8.62920000	-0.74380000	-15.16770000
C	-9.85390000	0.67000000	-14.96900000
H	-10.11920000	1.10500000	-15.93130000
H	-10.77230000	0.32850000	-14.49360000
H	-9.43880000	1.46460000	-14.35850000
C	-9.30800000	-1.95540000	-16.44590000
H	-8.62090000	-2.77940000	-16.63160000
H	-10.25330000	-2.38600000	-16.11890000
H	-9.48410000	-1.46290000	-17.40110000
C	-6.92370000	-0.06840000	-15.71040000
C	-5.98340000	-1.23600000	-16.05810000
H	-6.38110000	-1.84240000	-16.87220000
H	-5.00290000	-0.87620000	-16.37100000

H	-5.82600000	-1.89740000	-15.20740000
C	-7.10790000	0.82320000	-16.95060000
H	-7.55200000	0.26780000	-17.77760000
H	-7.75870000	1.67250000	-16.73940000
H	-6.15450000	1.22220000	-17.29800000
C	-6.32110000	0.75890000	-14.56210000
H	-6.93860000	1.62450000	-14.32500000
H	-6.23180000	0.16700000	-13.65060000
H	-5.32550000	1.12780000	-14.80980000
O	-6.16960000	-4.18190000	-12.82730000
H	-5.73630000	-4.33630000	-11.99090000
O	-5.85430000	-3.23110000	-10.48280000
H	-5.19840000	-2.57870000	-10.75220000
C	-11.27900000	0.33430000	-9.31330000
C	-9.09740000	0.76340000	-7.29770000
O	-11.42450000	-0.77520000	-9.82870000
O	-9.33780000	1.77910000	-6.64220000
N	-12.16680000	0.86220000	-8.46370000
N	-9.14810000	-0.45590000	-6.74900000
H	-11.94200000	1.76630000	-8.07240000
H	-8.94590000	-1.24460000	-7.36860000
C	-10.05650000	1.20600000	-9.63450000
H	-10.33280000	2.23460000	-9.39330000
C	-9.72390000	1.15330000	-11.13740000
H	-9.33000000	0.16880000	-11.39780000
H	-10.62380000	1.27580000	-11.73670000
C	-7.66620000	1.86750000	-9.11280000
H	-7.91670000	2.81930000	-8.64760000
C	-8.68270000	2.22410000	-11.49150000
H	-8.39070000	2.12300000	-12.53440000
C	-7.41420000	2.09730000	-10.62450000
H	-6.79550000	2.98410000	-10.75850000
C	-9.49230000	-0.74350000	-5.36200000
H	-8.85760000	-0.14950000	-4.70230000
H	-9.26030000	-1.79010000	-5.16420000
C	-13.37200000	0.19150000	-8.00800000
H	-13.10710000	-0.79110000	-7.61550000
H	-14.03470000	0.02740000	-8.85930000
C	-8.79150000	0.84770000	-8.80930000
H	-8.44150000	-0.12300000	-9.14920000
C	-10.98110000	-0.46360000	-5.08480000
H	-11.58770000	-1.04430000	-5.77920000
H	-11.19670000	0.58420000	-5.29460000
C	-11.42000000	-0.76830000	-3.64650000
H	-11.29470000	-1.83220000	-3.44450000
H	-10.77400000	-0.24260000	-2.94320000
C	-12.88010000	-0.35480000	-3.40800000
H	-12.98320000	0.71830000	-3.57650000
H	-13.51450000	-0.84380000	-4.14750000
C	-13.39290000	-0.69770000	-2.00310000
H	-13.30430000	-1.77150000	-1.83670000
H	-12.76370000	-0.21860000	-1.25280000
C	-14.85320000	-0.26520000	-1.80530000
H	-15.46930000	-0.71750000	-2.58260000
H	-14.93440000	0.81470000	-1.93730000
C	-15.41310000	-0.65660000	-0.43130000
H	-15.33610000	-1.73650000	-0.30290000
H	-14.80120000	-0.21210000	0.35370000
C	-16.87560000	-0.22460000	-0.25250000
H	-17.47900000	-0.65700000	-1.05080000
H	-16.95220000	0.85820000	-0.35930000
C	-17.45540000	-0.64890000	1.10340000
H	-17.38510000	-1.73220000	1.20480000
H	-16.84930000	-0.22870000	1.90610000
C	-18.91700000	-0.21460000	1.28150000
H	-19.51880000	-0.63140000	0.47400000
H	-18.98880000	0.87010000	1.19300000
C	-19.50030000	-0.65920000	2.62940000

H	-19.43590000	-1.74440000	2.71230000
H	-18.89190000	-0.25570000	3.43880000
C	-20.95880000	-0.21980000	2.81700000
H	-21.02470000	0.86610000	2.74200000
H	-21.56590000	-0.62330000	2.00650000
C	-21.53830000	-0.67710000	4.16220000
H	-21.48260000	-1.76380000	4.23120000
H	-20.92200000	-0.28840000	4.97300000
C	-22.99130000	-0.22710000	4.36410000
H	-23.04690000	0.86000000	4.29900000
H	-23.60790000	-0.61710000	3.55420000
C	-23.56540000	-0.68930000	5.71020000
H	-23.53190000	-1.77760000	5.77110000
H	-22.93900000	-0.31890000	6.52170000
C	-25.00600000	-0.21680000	5.92950000
H	-25.07460000	0.87020000	5.89450000
H	-25.37840000	-0.54040000	6.90170000
H	-25.67290000	-0.62040000	5.16810000
C	-14.08110000	1.01700000	-6.92520000
H	-14.35750000	1.99280000	-7.32770000
H	-13.39680000	1.20530000	-6.09730000
C	-15.33350000	0.30590000	-6.39850000
H	-16.01900000	0.12340000	-7.22730000
H	-15.05470000	-0.67380000	-6.00990000
C	-16.06060000	1.09480000	-5.30210000
H	-16.34050000	2.07830000	-5.68220000
H	-15.38490000	1.26780000	-4.46370000
C	-17.31330000	0.35620000	-4.81290000
H	-17.97990000	0.17740000	-5.65760000
H	-17.02960000	-0.62540000	-4.43290000
C	-18.07410000	1.12030000	-3.72350000
H	-18.35410000	2.10530000	-4.09880000
H	-17.41750000	1.28850000	-2.86940000
C	-19.33260000	0.36880000	-3.26950000
H	-19.98200000	0.19660000	-4.12880000
H	-19.05100000	-0.61520000	-2.89480000
C	-20.11510000	1.12040000	-2.18660000
H	-20.39560000	2.10560000	-2.56080000
H	-19.47010000	1.28780000	-1.32360000
C	-21.37660000	0.36500000	-1.74810000
H	-22.02070000	0.19970000	-2.61270000
H	-21.09720000	-0.62130000	-1.37840000
C	-22.16220000	1.11150000	-0.66330000
H	-21.51780000	1.27510000	0.20100000
H	-22.44130000	2.09860000	-1.03320000
C	-23.42470000	0.35730000	-0.22600000
H	-23.14730000	-0.63230000	0.13550000
H	-24.07310000	0.20090000	-1.08900000
C	-24.20120000	1.09970000	0.86870000
H	-23.55050000	1.25570000	1.72970000
H	-24.47920000	2.09030000	0.50710000
C	-25.46340000	0.34790000	1.31150000
H	-25.18730000	-0.64630000	1.66140000
H	-26.12050000	0.20200000	0.45320000
C	-26.22540000	1.08480000	2.42060000
H	-25.56530000	1.23110000	3.27610000
H	-26.50240000	2.07940000	2.06940000
C	-27.48630000	0.33500000	2.87050000
H	-27.21580000	-0.66210000	3.21800000
H	-28.15230000	0.19410000	2.01830000
C	-28.24120000	1.06680000	3.98570000
H	-27.61610000	1.19500000	4.86980000
H	-29.12770000	0.50770000	4.28620000
H	-28.56790000	2.05470000	3.66000000
O	-9.23770000	3.51570000	-11.31580000
Si	-10.08760000	4.39600000	-12.48530000
C	-11.64300000	3.46000000	-12.99870000
H	-12.27950000	4.07360000	-13.63420000

H	-11.41070000	2.55510000	-13.55430000
H	-12.23240000	3.17130000	-12.12950000
C	-8.97190000	4.67410000	-13.98100000
H	-8.11990000	5.30180000	-13.72700000
H	-8.58160000	3.73920000	-14.37850000
H	-9.50960000	5.17230000	-14.78620000
C	-10.60590000	6.06350000	-11.71330000
C	-11.67510000	5.80530000	-10.63770000
H	-11.32730000	5.07840000	-9.90440000
H	-11.93260000	6.71800000	-10.10030000
H	-12.59310000	5.40940000	-11.07230000
C	-9.37110000	6.71580000	-11.07430000
H	-8.90280000	6.03950000	-10.35810000
H	-8.62250000	6.96600000	-11.82460000
H	-9.62730000	7.63380000	-10.54550000
C	-11.18030000	6.98580000	-12.80240000
H	-10.44230000	7.19500000	-13.57750000
H	-12.04760000	6.53810000	-13.28850000
H	-11.49520000	7.94250000	-12.38500000
O	-6.63850000	0.98870000	-11.02220000
H	-6.39650000	0.64150000	-10.15850000
O	-6.42750000	1.39170000	-8.59120000
H	-5.94300000	2.16560000	-8.27940000
C	-9.64340000	5.27170000	-7.04680000
C	-8.11140000	4.38940000	-4.42800000
O	-9.69950000	4.53380000	-8.02630000
O	-7.92990000	5.21050000	-3.52730000
N	-10.71170000	5.60200000	-6.31740000
N	-8.81420000	3.26510000	-4.24940000
H	-10.56070000	6.26140000	-5.55240000
H	-8.95070000	2.66880000	-5.06730000
C	-8.29650000	5.78950000	-6.53120000
H	-8.50460000	6.55740000	-5.78800000
C	-7.46660000	6.50820000	-7.62850000
H	-8.12200000	6.95430000	-8.37640000
H	-6.94320000	7.34990000	-7.17250000
C	-6.02690000	4.92230000	-5.82340000
H	-5.87210000	5.89350000	-5.34810000
C	-6.42650000	5.60490000	-8.31140000
H	-6.94740000	4.85230000	-8.90350000
C	-5.48070000	4.95110000	-7.27350000
H	-4.51450000	5.45580000	-7.28270000
C	-9.36340000	2.81120000	-2.98230000
H	-8.70100000	3.09620000	-2.16230000
H	-9.38280000	1.72200000	-2.99850000
C	-12.00150000	4.93560000	-6.36810000
H	-11.84430000	3.85870000	-6.27820000
H	-12.48090000	5.11750000	-7.33120000
C	-7.53820000	4.62260000	-5.83550000
H	-7.67550000	3.70460000	-6.40720000
C	-10.77700000	3.36330000	-2.74920000
H	-11.42330000	3.07030000	-3.57790000
H	-10.74420000	4.45400000	-2.74630000
C	-11.37130000	2.86370000	-1.42620000
H	-11.36120000	1.77350000	-1.41820000
H	-10.73080000	3.18210000	-0.60260000
C	-12.80280000	3.35840000	-1.18060000
H	-12.81660000	4.44880000	-1.18230000
H	-13.44570000	3.03650000	-2.00120000
C	-13.36650000	2.83790000	0.14890000
H	-13.33670000	1.74820000	0.15240000
H	-12.72210000	3.16180000	0.96670000
C	-14.80290000	3.30560000	0.41640000
H	-15.45040000	2.97360000	-0.39600000
H	-14.83550000	4.39530000	0.41540000
C	-15.34550000	2.77930000	1.75280000
H	-15.30820000	1.69020000	1.75660000
H	-14.69530000	3.10840000	2.56360000

C	-16.78190000	3.23980000	2.03280000
H	-17.43310000	2.90770000	1.22370000
H	-16.81810000	4.32920000	2.03400000
C	-17.31860000	2.71200000	3.37070000
H	-17.28040000	1.62280000	3.37430000
H	-16.66720000	3.04230000	4.17990000
C	-18.75500000	3.17270000	3.65090000
H	-19.40550000	2.84390000	2.83990000
H	-18.78990000	4.26200000	3.65470000
C	-19.29640000	2.64330000	4.98570000
H	-19.26180000	1.55400000	4.98670000
H	-18.64620000	2.97030000	5.79740000
C	-20.73210000	3.10920000	5.26130000
H	-20.76230000	4.19870000	5.26950000
H	-21.38090000	2.78700000	4.44620000
C	-21.28150000	2.57670000	6.59120000
H	-21.25370000	1.48720000	6.58650000
H	-20.63170000	2.89550000	7.40650000
C	-22.71490000	3.05100000	6.86490000
H	-22.73700000	4.14070000	6.88020000
H	-23.36370000	2.73990000	6.04540000
C	-23.27080000	2.51280000	8.19000000
H	-23.26460000	1.42260000	8.17770000
H	-22.61780000	2.81290000	9.00970000
C	-24.69350000	3.00550000	8.47240000
H	-24.72890000	4.09320000	8.52620000
H	-25.05870000	2.61600000	9.42280000
H	-25.38540000	2.68470000	7.69330000
C	-12.86680000	5.45000000	-5.20820000
H	-13.12820000	6.49240000	-5.39660000
H	-12.27070000	5.45120000	-4.29530000
C	-14.14130000	4.63440000	-4.94700000
H	-14.75810000	4.60320000	-5.84610000
H	-13.87020000	3.60280000	-4.71710000
C	-14.94250000	5.23320000	-3.77990000
H	-15.27030000	6.23790000	-4.05110000
H	-14.28520000	5.35310000	-2.91860000
C	-16.15830000	4.39620000	-3.35870000
H	-16.82040000	4.25140000	-4.21340000
H	-15.82960000	3.40370000	-3.04730000
C	-16.93180000	5.06760000	-2.21330000
H	-17.27080000	6.05270000	-2.53730000
H	-16.25700000	5.24020000	-1.37520000
C	-18.13760000	4.25110000	-1.72840000
H	-18.81170000	4.06750000	-2.56570000
H	-17.80130000	3.27420000	-1.37900000
C	-18.90290000	4.96340000	-0.60330000
H	-19.23290000	5.94260000	-0.95290000
H	-18.22760000	5.14940000	0.23110000
C	-20.11600000	4.16610000	-0.10510000
H	-20.79110000	3.97740000	-0.94040000
H	-19.78820000	3.19070000	0.25620000
C	-20.87890000	4.89420000	1.01050000
H	-20.20740000	5.07460000	1.84890000
H	-21.19560000	5.87540000	0.65430000
C	-22.10310000	4.10980000	1.50130000
H	-21.78730000	3.13050000	1.86230000
H	-22.77530000	3.92930000	0.66190000
C	-22.86560000	4.84170000	2.61420000
H	-22.19890000	5.00930000	3.45910000
H	-23.16880000	5.82820000	2.26090000
C	-24.10130000	4.06650000	3.08930000
H	-23.79740000	3.08150000	3.44510000
H	-24.76830000	3.89850000	2.24310000
C	-24.86630000	4.79570000	4.20180000
H	-24.20540000	4.94900000	5.05410000
H	-25.15790000	5.78800000	3.85520000
C	-26.11220000	4.02510000	4.65710000

H	-25.82170000	3.03440000	5.00700000
H	-26.77690000	3.87060000	3.80660000
C	-26.88050000	4.74520000	5.76930000
H	-26.25990000	4.87370000	6.65510000
H	-27.76280000	4.17630000	6.06360000
H	-27.21290000	5.73240000	5.44700000
O	-5.65920000	6.42860000	-9.17100000
Si	-4.75610000	5.92300000	-10.51340000
C	-5.92380000	5.75220000	-11.97930000
H	-6.33150000	6.71720000	-12.27250000
H	-5.41500000	5.33140000	-12.84530000
H	-6.76270000	5.10230000	-11.73850000
C	-3.87460000	4.28700000	-10.22450000
H	-3.20600000	4.33830000	-9.36670000
H	-4.57500000	3.47630000	-10.04520000
H	-3.27020000	3.99940000	-11.08320000
C	-3.46160000	7.28500000	-10.85720000
C	-2.61140000	7.47910000	-9.59070000
H	-2.10840000	6.55730000	-9.29710000
H	-1.85000000	8.24600000	-9.72770000
H	-3.23260000	7.78420000	-8.74870000
C	-2.56780000	6.85830000	-12.03420000
H	-2.04810000	5.92440000	-11.81810000
H	-3.15340000	6.70220000	-12.94090000
H	-1.81190000	7.61220000	-12.25390000
C	-4.18310000	8.60090000	-11.19410000
H	-4.77720000	8.51140000	-12.10360000
H	-4.85950000	8.89410000	-10.38950000
H	-3.47560000	9.41650000	-11.34370000
O	-5.28190000	3.58610000	-7.56560000
H	-5.12030000	3.23970000	-6.68170000
O	-5.25800000	3.94130000	-5.12180000
H	-4.46860000	4.39010000	-4.81640000
C	0.63040000	-0.63960000	-11.41010000
C	-0.96280000	1.86980000	-11.84170000
O	0.56440000	-1.39090000	-12.38210000
O	-0.72820000	2.70990000	-10.97330000
N	1.77500000	-0.03690000	-11.06250000
N	-0.62180000	2.04850000	-13.12320000
H	1.78270000	0.61980000	-10.28320000
H	-0.83870000	1.29780000	-13.76300000
C	-0.65090000	-0.32840000	-10.61050000
H	-0.39070000	0.22350000	-9.70480000
C	-1.32180000	-1.66200000	-10.20450000
H	-1.50100000	-2.26420000	-11.09750000
H	-0.64730000	-2.24950000	-9.58070000
C	-2.99880000	0.74640000	-10.80300000
H	-2.89970000	1.47000000	-9.99020000
C	-2.66650000	-1.48560000	-9.48550000
H	-3.14500000	-2.46410000	-9.43740000
C	-3.62740000	-0.54660000	-10.23660000
H	-4.45320000	-0.27370000	-9.58140000
C	0.05510000	3.21860000	-13.66080000
H	-0.41010000	4.12690000	-13.27310000
H	-0.10010000	3.22570000	-14.74000000
C	3.01130000	-0.18300000	-11.82440000
H	2.79090000	-0.11250000	-12.89140000
H	3.42310000	-1.17840000	-11.65170000
C	-1.62020000	0.52390000	-11.48100000
H	-1.79890000	-0.03060000	-12.40450000
C	1.56080000	3.20130000	-13.34380000
H	1.98700000	2.24060000	-13.63610000
H	1.70900000	3.29020000	-12.26610000
C	2.30860000	4.32910000	-14.06510000
H	2.16430000	4.21730000	-15.14070000
H	1.86150000	5.28620000	-13.79640000
C	3.81370000	4.36050000	-13.75950000
H	3.97100000	4.52920000	-12.69400000

H	4.25540000	3.38960000	-13.98810000
C	4.52710000	5.44980000	-14.57150000
H	4.38400000	5.25100000	-15.63460000
H	4.05320000	6.41210000	-14.37730000
C	6.03020000	5.55800000	-14.27770000
H	6.50860000	4.59350000	-14.45140000
H	6.18470000	5.79890000	-13.22560000
C	6.69480000	6.62570000	-15.15790000
H	6.54860000	6.36400000	-16.20690000
H	6.18880000	7.58000000	-15.01010000
C	8.19520000	6.80340000	-14.88780000
H	8.70640000	5.84940000	-15.02060000
H	8.34970000	7.10000000	-13.85020000
C	8.81520000	7.85120000	-15.82300000
H	8.66470000	7.54100000	-16.85800000
H	8.28370000	8.79620000	-15.70800000
C	10.31260000	8.07990000	-15.57730000
H	10.84800000	7.13590000	-15.68330000
H	10.46830000	8.41450000	-14.55160000
C	10.89920000	9.11220000	-16.55030000
H	10.74920000	8.76750000	-17.57430000
H	10.34740000	10.04820000	-16.45910000
C	12.39250000	9.37890000	-16.31880000
H	12.54480000	9.73790000	-15.30090000
H	12.94700000	8.44410000	-16.40620000
C	12.95740000	10.40250000	-17.31340000
H	12.81460000	10.03520000	-18.33050000
H	12.38860000	11.32980000	-17.23980000
C	14.44520000	10.69890000	-17.08360000
H	14.58880000	11.07020000	-16.06890000
H	15.01640000	9.77310000	-17.15960000
C	14.99690000	11.72310000	-18.08470000
H	14.86850000	11.34860000	-19.10110000
H	14.41770000	12.64470000	-18.02240000
C	16.47640000	12.04180000	-17.84740000
H	16.63640000	12.44980000	-16.85000000
H	16.83590000	12.77860000	-18.56620000
H	17.09340000	11.14910000	-17.95020000
C	4.03250000	0.89680000	-11.44050000
H	4.31380000	0.77270000	-10.39400000
H	3.56280000	1.87710000	-11.52270000
C	5.29420000	0.87740000	-12.31340000
H	5.78110000	-0.09550000	-12.23410000
H	5.00800000	0.99590000	-13.35940000
C	6.28300000	1.98630000	-11.92340000
H	6.61010000	1.83700000	-10.89360000
H	5.77300000	2.94930000	-11.94840000
C	7.50540000	2.04490000	-12.84800000
H	8.02750000	1.08720000	-12.82650000
H	7.17190000	2.19070000	-13.87650000
C	8.47790000	3.16910000	-12.46240000
H	8.84780000	3.00240000	-11.44980000
H	7.94480000	4.11960000	-12.44250000
C	9.66070000	3.27620000	-13.43310000
H	10.20240000	2.32950000	-13.45420000
H	9.28240000	3.43630000	-14.44380000
C	10.62710000	4.41090000	-13.06580000
H	11.03550000	4.23730000	-12.06930000
H	10.07980000	5.35200000	-13.01420000
C	11.77200000	4.54580000	-14.07820000
H	12.32540000	3.60700000	-14.12870000
H	11.35500000	4.71050000	-15.07280000
C	12.73620000	5.68960000	-13.73590000
H	12.18030000	6.62450000	-13.66850000
H	13.17270000	5.51910000	-12.75080000
C	13.85290000	5.83610000	-14.77810000
H	13.40950000	5.99900000	-15.76160000
H	14.41280000	4.90220000	-14.84410000

C	14.81580000	6.98700000	-14.45780000
H	14.25490000	7.91860000	-14.38730000
H	15.26740000	6.82470000	-13.47810000
C	15.91770000	7.13530000	-15.51570000
H	15.46160000	7.29530000	-16.49380000
H	16.48010000	6.20320000	-15.58670000
C	16.88020000	8.28990000	-15.20760000
H	16.31760000	9.22090000	-15.14290000
H	17.33400000	8.13560000	-14.22760000
C	17.98090000	8.43310000	-16.26750000
H	17.52820000	8.59300000	-17.24710000
H	18.54620000	7.50280000	-16.33740000
C	18.94280000	9.58580000	-15.96220000
H	18.41550000	10.53850000	-15.93480000
H	19.71830000	9.65930000	-16.72490000
H	19.43500000	9.44770000	-14.99910000
O	-2.45830000	-1.04600000	-8.15690000
Si	-2.86110000	-1.94380000	-6.77430000
C	-1.65880000	-1.50390000	-5.39490000
H	-1.70920000	-2.21790000	-4.57500000
H	-0.63370000	-1.47660000	-5.75970000
H	-1.89810000	-0.52330000	-4.98890000
C	-2.68600000	-3.78910000	-7.13940000
H	-3.42330000	-4.13110000	-7.86470000
H	-1.69960000	-4.02260000	-7.53740000
H	-2.82640000	-4.38290000	-6.23770000
C	-4.64460000	-1.54730000	-6.19880000
C	-5.66360000	-2.06910000	-7.22480000
H	-5.57530000	-3.14470000	-7.37830000
H	-6.68400000	-1.87240000	-6.89920000
H	-5.54470000	-1.59360000	-8.19520000
C	-4.90400000	-2.23150000	-4.84530000
H	-4.79140000	-3.31370000	-4.91970000
H	-4.20960000	-1.88060000	-4.08120000
H	-5.91360000	-2.02910000	-4.48710000
C	-4.79260000	-0.02440000	-6.04410000
H	-4.14260000	0.36640000	-5.26080000
H	-4.51870000	0.48800000	-6.96650000
H	-5.81610000	0.26090000	-5.79890000
O	-4.17740000	-1.23220000	-11.34140000
H	-4.21220000	-0.54620000	-12.01010000
O	-3.91900000	1.23210000	-11.78970000
H	-4.79390000	1.31090000	-11.40570000
C	2.49770000	2.83820000	-8.21540000
C	0.41900000	4.95400000	-8.40160000
O	2.35720000	2.01990000	-9.12130000
O	0.80920000	5.93750000	-7.77130000
N	3.55320000	3.64970000	-8.10700000
N	0.32120000	4.96270000	-9.73660000
H	3.54470000	4.30470000	-7.33880000
H	-0.03720000	4.11350000	-10.18210000
C	1.42390000	2.98530000	-7.13920000
H	1.82340000	3.63800000	-6.35950000
C	1.13260000	1.61510000	-6.50170000
H	0.67040000	0.95360000	-7.23720000
H	2.05780000	1.12790000	-6.19610000
C	-0.85540000	3.81880000	-6.48770000
H	-0.45350000	4.56960000	-5.80950000
C	0.19040000	1.76780000	-5.30390000
H	-0.06630000	0.78150000	-4.92160000
C	-1.09470000	2.52140000	-5.68700000
H	-1.65320000	2.75020000	-4.78300000
C	0.71830000	6.05980000	-10.60890000
H	0.17930000	6.96150000	-10.32110000
H	0.40430000	5.81620000	-11.62340000
C	4.66070000	3.71020000	-9.04320000
H	4.27870000	3.70730000	-10.06530000
H	5.27360000	2.81510000	-8.92770000

C	0.11660000	3.62680000	-7.67720000
H	-0.33860000	2.93860000	-8.39050000
C	2.23870000	6.29620000	-10.57270000
H	2.75080000	5.37210000	-10.83880000
H	2.54840000	6.53370000	-9.55490000
C	2.70680000	7.42720000	-11.49580000
H	2.49790000	7.16510000	-12.53270000
H	2.13610000	8.33190000	-11.28730000
C	4.20410000	7.71280000	-11.31920000
H	4.39550000	8.01330000	-10.28790000
H	4.76600000	6.79290000	-11.48110000
C	4.72740000	8.79570000	-12.26950000
H	4.56200000	8.48580000	-13.30150000
H	4.15600000	9.71350000	-12.13120000
C	6.21990000	9.07720000	-12.05180000
H	6.78090000	8.14860000	-12.15810000
H	6.38080000	9.42040000	-11.02880000
C	6.77450000	10.11480000	-13.03490000
H	6.62540000	9.76360000	-14.05620000
H	6.20730000	11.04120000	-12.94570000
C	8.26510000	10.39750000	-12.80600000
H	8.82590000	9.46600000	-12.88320000
H	8.41520000	10.76630000	-11.79060000
C	8.82780000	11.41080000	-13.81010000
H	8.68650000	11.03410000	-14.82350000
H	8.25810000	12.33800000	-13.74780000
C	10.31560000	11.70500000	-13.57930000
H	10.88230000	10.77570000	-13.63890000
H	10.45880000	12.09120000	-12.56950000
C	10.87480000	12.70670000	-14.59720000
H	10.74000000	12.31330000	-15.60510000
H	10.29760000	13.63030000	-14.55080000
C	12.35920000	13.01750000	-14.36640000
H	12.49550000	13.41560000	-13.36040000
H	12.93520000	12.09340000	-14.41440000
C	12.91120000	14.01510000	-15.39280000
H	12.78430000	13.61040000	-16.39710000
H	12.32380000	14.93270000	-15.35720000
C	14.39090000	14.34510000	-15.15880000
H	14.51720000	14.75010000	-14.15430000
H	14.97820000	13.42770000	-15.19810000
C	14.93640000	15.34480000	-16.18710000
H	14.83140000	14.93460000	-17.19200000
H	14.33690000	16.25490000	-16.16480000
C	16.40530000	15.70130000	-15.93910000
H	16.54520000	16.12780000	-14.94620000
H	16.75690000	16.43330000	-16.66640000
H	17.04320000	14.82150000	-16.01950000
C	5.49160000	4.97560000	-8.79220000
H	5.85900000	4.97550000	-7.76480000
H	4.85570000	5.85610000	-8.89320000
C	6.67820000	5.09280000	-9.75440000
H	7.31950000	4.21700000	-9.64490000
H	6.31450000	5.07960000	-10.78230000
C	7.50340000	6.36530000	-9.52110000
H	7.85500000	6.39050000	-8.48860000
H	6.86900000	7.24290000	-9.65150000
C	8.70330000	6.44970000	-10.47200000
H	9.33590000	5.57130000	-10.33520000
H	8.35130000	6.41480000	-11.50320000
C	9.54070000	7.71720000	-10.26200000
H	9.88690000	7.75930000	-9.22840000
H	8.91480000	8.59730000	-10.41370000
C	10.74680000	7.77050000	-11.20880000
H	11.37160000	6.89030000	-11.05050000
H	10.39870000	7.71670000	-12.24040000
C	11.59310000	9.03570000	-11.02220000
H	11.94190000	9.09070000	-9.99030000

H	10.97060000	9.91600000	-11.18550000
C	12.79860000	9.07430000	-11.97100000
H	13.42320000	8.19620000	-11.80110000
H	12.44940000	9.00780000	-13.00130000
C	13.64410000	10.34290000	-11.80060000
H	13.01850000	11.21980000	-11.97030000
H	13.99560000	10.40840000	-10.77040000
C	14.84760000	10.37990000	-12.75200000
H	14.49710000	10.30260000	-13.78110000
H	15.47810000	9.50740000	-12.57510000
C	15.68410000	11.65630000	-12.59360000
H	15.05150000	12.52690000	-12.76940000
H	16.03630000	11.73280000	-11.56430000
C	16.88670000	11.69740000	-13.54620000
H	16.53670000	11.60900000	-14.57460000
H	17.52540000	10.83220000	-13.36290000
C	17.71100000	12.98330000	-13.39730000
H	17.06980000	13.84630000	-13.57910000
H	18.06180000	13.07000000	-12.36830000
C	18.91380000	13.03070000	-14.34980000
H	18.57060000	12.94160000	-15.38080000
H	19.56100000	12.17220000	-14.16590000
C	19.73120000	14.31910000	-14.20390000
H	19.12470000	15.19990000	-14.41630000
H	20.57440000	14.32430000	-14.89500000
H	20.13040000	14.42220000	-13.19460000
O	0.84200000	2.48350000	-4.27350000
Si	1.60710000	1.77810000	-2.94540000
C	2.92330000	0.57810000	-3.56410000
H	3.48260000	0.14300000	-2.73720000
H	2.48140000	-0.24190000	-4.12830000
H	3.63820000	1.07890000	-4.21520000
C	0.32740000	0.85020000	-1.91650000
H	-0.39770000	1.52950000	-1.47380000
H	-0.22410000	0.12820000	-2.51710000
H	0.79830000	0.30660000	-1.09900000
C	2.41040000	3.17830000	-1.93030000
C	3.48150000	3.86860000	-2.79210000
H	3.05430000	4.24030000	-3.72450000
H	3.92500000	4.71850000	-2.27350000
H	4.28770000	3.18330000	-3.05440000
C	1.32150000	4.19340000	-1.54890000
H	0.83360000	4.59480000	-2.43730000
H	0.54790000	3.73250000	-0.93720000
H	1.73030000	5.03480000	-0.98950000
C	3.05250000	2.59420000	-0.66070000
H	2.31150000	2.09890000	-0.03210000
H	3.82010000	1.85900000	-0.90420000
H	3.52270000	3.37340000	-0.06020000
O	-1.92160000	1.72330000	-6.49820000
H	-2.36800000	2.36370000	-7.04840000
O	-2.13780000	4.23950000	-6.92560000
H	-2.56150000	4.62520000	-6.16090000
C	1.29160000	7.17470000	-4.53570000
C	-0.55590000	8.97220000	-6.43550000
O	1.30570000	5.96130000	-4.71770000
O	-0.88130000	10.14090000	-6.22380000
N	2.37520000	7.94090000	-4.66410000
N	0.19940000	8.62820000	-7.48560000
H	2.28410000	8.92120000	-4.39730000
H	0.42770000	7.63560000	-7.59130000
C	-0.01660000	7.90590000	-4.21760000
H	0.22740000	8.94780000	-4.02560000
C	-0.64670000	7.40540000	-2.89530000
H	0.12560000	7.05590000	-2.20800000
H	-1.12890000	8.24180000	-2.38960000
C	-2.43970000	7.95700000	-4.99750000
H	-2.57840000	8.91400000	-4.49150000

C	-1.69420000	6.31160000	-3.09390000
H	-1.20480000	5.42410000	-3.50000000
C	-2.82040000	6.80070000	-4.03050000
H	-3.68640000	7.11570000	-3.45420000
C	0.73930000	9.54620000	-8.48130000
H	0.11310000	10.43620000	-8.56560000
H	0.69550000	9.05360000	-9.45130000
C	3.61690000	7.52420000	-5.28960000
H	3.39240000	7.12390000	-6.28050000
H	4.07790000	6.72200000	-4.71080000
C	-0.96470000	7.85260000	-5.45480000
H	-0.85630000	6.89760000	-5.96980000
C	2.19150000	9.93690000	-8.15680000
H	2.79870000	9.03350000	-8.07990000
H	2.22470000	10.41990000	-7.17920000
C	2.79750000	10.87400000	-9.21480000
H	2.71980000	10.40570000	-10.19610000
H	2.20290000	11.78650000	-9.26680000
C	4.26840000	11.23540000	-8.94620000
H	4.35570000	11.70090000	-7.96400000
H	4.86610000	10.32340000	-8.91140000
C	4.84090000	12.18180000	-10.01530000
H	4.74390000	11.71940000	-10.99740000
H	4.24120000	13.09150000	-10.05050000
C	6.31330000	12.55430000	-9.77820000
H	6.91950000	11.64790000	-9.76390000
H	6.41780000	13.01340000	-8.79480000
C	6.85490000	13.51500000	-10.85020000
H	6.74620000	13.05970000	-11.83430000
H	6.24630000	14.41920000	-10.86700000
C	8.32620000	13.89890000	-10.63100000
H	8.93890000	12.99710000	-10.62370000
H	8.43700000	14.35770000	-9.64840000
C	8.85220000	14.86400000	-11.70560000
H	8.73870000	14.40970000	-12.68970000
H	8.24000000	15.76590000	-11.71500000
C	10.32320000	15.25060000	-11.49230000
H	10.93720000	14.34960000	-11.48420000
H	10.43610000	15.71190000	-10.51130000
C	10.84730000	16.21340000	-12.56840000
H	10.73530000	15.75680000	-13.55170000
H	10.23430000	17.11480000	-12.57930000
C	12.31750000	16.59960000	-12.35100000
H	12.42740000	17.06380000	-11.37120000
H	12.93150000	15.69860000	-12.33800000
C	12.84480000	17.55900000	-13.42730000
H	12.73810000	17.09880000	-14.40950000
H	12.23050000	18.45950000	-13.44370000
C	14.31270000	17.94750000	-13.20230000
H	14.41620000	18.41620000	-12.22370000
H	14.92830000	17.04770000	-13.18070000
C	14.84220000	18.90310000	-14.28030000
H	14.75360000	18.43810000	-15.26220000
H	14.22340000	19.79990000	-14.31090000
C	16.30130000	19.30620000	-14.04390000
H	16.42080000	19.80810000	-13.08430000
H	16.64590000	19.99100000	-14.81900000
H	16.95880000	18.43680000	-14.05210000
C	4.54950000	8.73680000	-5.39730000
H	4.87530000	9.02500000	-4.39650000
H	3.98880000	9.58660000	-5.78750000
C	5.77030000	8.51050000	-6.29790000
H	6.34350000	7.65160000	-5.94610000
H	5.43610000	8.27110000	-7.30880000
C	6.66120000	9.75960000	-6.32570000
H	7.05940000	9.93570000	-5.32520000
H	6.05200000	10.63150000	-6.56520000
C	7.81750000	9.67710000	-7.32940000

H	8.43040000	8.79930000	-7.12020000
H	7.42110000	9.54760000	-8.33760000
C	8.68370000	10.94230000	-7.26820000
H	9.09680000	11.04620000	-6.26370000
H	8.05510000	11.81840000	-7.42830000
C	9.82690000	10.95170000	-8.28920000
H	10.45780000	10.07440000	-8.14100000
H	9.41920000	10.87570000	-9.29800000
C	10.67620000	12.22370000	-8.16730000
H	11.07980000	12.29390000	-7.15620000
H	10.03910000	13.09760000	-8.30140000
C	11.82790000	12.27520000	-9.17760000
H	12.46770000	11.40250000	-9.04250000
H	11.42880000	12.21450000	-10.19080000
C	12.66610000	13.55160000	-9.02990000
H	12.02570000	14.42190000	-9.16920000
H	13.05390000	13.61650000	-8.01240000
C	13.83160000	13.61200000	-10.02430000
H	13.44580000	13.54790000	-11.04220000
H	14.47390000	12.74260000	-9.88040000
C	14.66410000	14.89140000	-9.87190000
H	14.02430000	15.75890000	-10.02890000
H	15.03660000	14.96490000	-8.84920000
C	15.84340000	14.94420000	-10.85080000
H	15.47050000	14.86960000	-11.87270000
H	16.48410000	14.07660000	-10.69020000
C	16.67500000	16.22480000	-10.70360000
H	16.03770000	17.09080000	-10.87920000
H	17.03600000	16.31060000	-9.67780000
C	17.86460000	16.26290000	-11.67090000
H	17.50430000	16.17870000	-12.69640000
H	18.50550000	15.39820000	-11.49580000
C	18.69730000	17.54080000	-11.53390000
H	18.09730000	18.42410000	-11.74820000
H	19.53700000	17.53450000	-12.22930000
H	19.10010000	17.64580000	-10.52610000
O	-2.20390000	6.02170000	-1.80270000
Si	-3.31250000	4.80810000	-1.39530000
C	-2.87270000	3.23000000	-2.31780000
H	-1.79390000	3.10530000	-2.38520000
H	-3.28190000	2.34810000	-1.82850000
H	-3.27370000	3.24800000	-3.32810000
C	-5.06790000	5.35770000	-1.80190000
H	-5.30790000	6.29930000	-1.31090000
H	-5.22270000	5.49790000	-2.86810000
H	-5.80290000	4.62400000	-1.47580000
C	-3.17230000	4.54510000	0.49190000
C	-3.47940000	5.87510000	1.20180000
H	-4.49410000	6.21540000	0.99230000
H	-3.38060000	5.78350000	2.28350000
H	-2.79960000	6.66280000	0.87310000
C	-4.17620000	3.47040000	0.94480000
H	-5.20200000	3.76120000	0.71610000
H	-3.98920000	2.51520000	0.45380000
H	-4.11730000	3.30160000	2.02030000
C	-1.74110000	4.09830000	0.82980000
H	-1.48870000	3.16170000	0.33140000
H	-1.01330000	4.84660000	0.51300000
H	-1.61130000	3.94640000	1.90150000
O	-3.19640000	5.76340000	-4.90330000
H	-3.34720000	6.30890000	-5.69050000
O	-3.35860000	7.88220000	-6.09390000
H	-4.11880000	8.43080000	-5.88100000

4-beta octamer

C	-10.75280	-4.22120	-11.09300
C	-8.59130	-3.57540	-9.14680
O	-10.74840	-5.44700	-11.19790
O	-8.67300	-2.46090	-8.63620
N	-11.79440	-3.54270	-10.60210
N	-8.85500	-4.68800	-8.44980
H	-11.69380	-2.53760	-10.46240
H	-8.81010	-5.56580	-8.94830
C	-9.51690	-3.41690	-11.51380
H	-9.74880	-2.35530	-11.40920
C	-9.21930	-3.70450	-12.99820
H	-8.96970	-4.76020	-13.12160
H	-10.10510	-3.53090	-13.61050
C	-7.08090	-2.84490	-11.10230
H	-7.29580	-1.80380	-10.85190
C	-8.05460	-2.86150	-13.52400
H	-7.77670	-3.24470	-14.50670
C	-6.80960	-2.94280	-12.62300
H	-6.10460	-2.16560	-12.91620
C	-9.35360	-4.70810	-7.08020
H	-8.75230	-4.04230	-6.45790
H	-9.21910	-5.71470	-6.68340
C	-13.04850	-4.17040	-10.21690
H	-12.85050	-5.11780	-9.71160
H	-13.61390	-4.40680	-11.11950
C	-8.26650	-3.73960	-10.64520
H	-7.99130	-4.78150	-10.81690
C	-10.84060	-4.30940	-7.02410
H	-11.40970	-4.94820	-7.70060
H	-10.96130	-3.28940	-7.39350
C	-11.43710	-4.40830	-5.61410
H	-11.33360	-5.43240	-5.25290
H	-10.86450	-3.78070	-4.93080
C	-12.91760	-3.99850	-5.57250
H	-13.01970	-2.95840	-5.88370
H	-13.47790	-4.59220	-6.29600
C	-13.53750	-4.18370	-4.18150
H	-13.44730	-5.23030	-3.88680
H	-12.97010	-3.61020	-3.44850
C	-15.01410	-3.76650	-4.12470
H	-15.57600	-4.31530	-4.88150
H	-15.10880	-2.70910	-4.37410
C	-15.63270	-4.02970	-2.74550
H	-15.54140	-5.09070	-2.50910
H	-15.06330	-3.49720	-1.98350
C	-17.10860	-3.61790	-2.65950
H	-17.67440	-4.13030	-3.43830
H	-17.20460	-2.54970	-2.85660
C	-17.71670	-3.94760	-1.28980
H	-17.62180	-5.01830	-1.10330
H	-17.14270	-3.44940	-0.50830
C	-19.19220	-3.54290	-1.17540
H	-19.76370	-4.02850	-1.96690
H	-19.29050	-2.46880	-1.33420
C	-19.78930	-3.91880	0.18730
H	-19.69710	-4.99550	0.33670
H	-19.20710	-3.44880	0.98020
C	-21.26190	-3.51110	0.32570
H	-21.35540	-2.43320	0.19340
H	-21.84260	-3.97430	-0.47260
C	-21.85140	-3.91380	1.68420
H	-21.76920	-4.99440	1.80820
H	-21.25870	-3.46740	2.48300
C	-23.31840	-3.49290	1.84160
H	-23.40050	-2.41210	1.72600
H	-23.91070	-3.93680	1.04080
C	-23.90310	-3.90660	3.19880
H	-23.83830	-4.98960	3.31120

H	-23.30330	-3.47850	4.00220
C	-25.36100	-3.46790	3.36980
H	-25.45700	-2.38480	3.30230
H	-25.74800	-3.77280	4.34230
H	-25.99810	-3.91130	2.60440
C	-13.86330	-3.25620	-9.29640
H	-14.07850	-2.31690	-9.80730
H	-13.26530	-3.00620	-8.42030
C	-15.17250	-3.92080	-8.85310
H	-15.77480	-4.15550	-9.73210
H	-14.94610	-4.87460	-8.37410
C	-15.98990	-3.05280	-7.88900
H	-16.23940	-2.10610	-8.36980
H	-15.38220	-2.81000	-7.01740
C	-17.27470	-3.75850	-7.43620
H	-17.88440	-3.99740	-8.30880
H	-17.01820	-4.71160	-6.97130
C	-18.10070	-2.92140	-6.45160
H	-18.37690	-1.97490	-6.91810
H	-17.48890	-2.67350	-5.58440
C	-19.36520	-3.65900	-5.99140
H	-19.97760	-3.90640	-6.85970
H	-19.08330	-4.60860	-5.53410
C	-20.19980	-2.84410	-4.99510
H	-20.49690	-1.90020	-5.45380
H	-19.58580	-2.58920	-4.13150
C	-21.44790	-3.60490	-4.52700
H	-22.06340	-3.85950	-5.39100
H	-21.14670	-4.55100	-4.07470
C	-22.28610	-2.80460	-3.52180
H	-21.66940	-2.54560	-2.66130
H	-22.59640	-1.86220	-3.97480
C	-23.52340	-3.57870	-3.04690
H	-23.21060	-4.52210	-2.59720
H	-24.14180	-3.83780	-3.90740
C	-24.36200	-2.78540	-2.03590
H	-23.74390	-2.52730	-1.17620
H	-24.67650	-1.84230	-2.48470
C	-25.59630	-3.56350	-1.55980
H	-25.28050	-4.50630	-1.11070
H	-26.21490	-3.82360	-2.41990
C	-26.43580	-2.77200	-0.54790
H	-25.81980	-2.51990	0.31530
H	-26.74500	-1.82550	-0.99340
C	-27.67520	-3.54710	-0.08040
H	-27.36890	-4.49240	0.36990
H	-28.29420	-3.80270	-0.94160
C	-28.51610	-2.75640	0.92750
H	-27.94230	-2.52620	1.82470
H	-29.39390	-3.32600	1.23390
H	-28.86370	-1.81530	0.50050
O	-8.48180	-1.51980	-13.67250
Si	-8.62920	-0.74380	-15.16770
C	-9.85390	0.67000	-14.96900
H	-10.11920	1.10500	-15.93130
H	-10.77230	0.32850	-14.49360
H	-9.43880	1.46460	-14.35850
C	-9.30800	-1.95540	-16.44590
H	-8.62090	-2.77940	-16.63160
H	-10.25330	-2.38600	-16.11890
H	-9.48410	-1.46290	-17.40110
C	-6.92370	-0.06840	-15.71040
C	-5.98340	-1.23600	-16.05810
H	-6.38110	-1.84240	-16.87220
H	-5.00290	-0.87620	-16.37100
H	-5.82600	-1.89740	-15.20740
C	-7.10790	0.82320	-16.95060
H	-7.55200	0.26780	-17.77760

H	-7.75870	1.67250	-16.73940
H	-6.15450	1.22220	-17.29800
C	-6.32110	0.75890	-14.56210
H	-6.93860	1.62450	-14.32500
H	-6.23180	0.16700	-13.65060
H	-5.32550	1.12780	-14.80980
O	-6.16960	-4.18190	-12.82730
H	-5.73630	-4.33630	-11.99090
O	-5.85430	-3.23110	-10.48280
H	-5.19840	-2.57870	-10.75220
C	-11.27900	0.33430	-9.31330
C	-9.09740	0.76340	-7.29770
O	-11.42450	-0.77520	-9.82870
O	-9.33780	1.77910	-6.64220
N	-12.16680	0.86220	-8.46370
N	-9.14810	-0.45590	-6.74900
H	-11.94200	1.76630	-8.07240
H	-8.94590	-1.24460	-7.36860
C	-10.05650	1.20600	-9.63450
H	-10.33280	2.23460	-9.39330
C	-9.72390	1.15330	-11.13740
H	-9.33000	0.16880	-11.39780
H	-10.62380	1.27580	-11.73670
C	-7.66620	1.86750	-9.11280
H	-7.91670	2.81930	-8.64760
C	-8.68270	2.22410	-11.49150
H	-8.39070	2.12300	-12.53440
C	-7.41420	2.09730	-10.62450
H	-6.79550	2.98410	-10.75850
C	-9.49230	-0.74350	-5.36200
H	-8.85760	-0.14950	-4.70230
H	-9.26030	-1.79010	-5.16420
C	-13.37200	0.19150	-8.00800
H	-13.10710	-0.79110	-7.61550
H	-14.03470	0.02740	-8.85930
C	-8.79150	0.84770	-8.80930
H	-8.44150	-0.12300	-9.14920
C	-10.98110	-0.46360	-5.08480
H	-11.58770	-1.04430	-5.77920
H	-11.19670	0.58420	-5.29460
C	-11.42000	-0.76830	-3.64650
H	-11.29470	-1.83220	-3.44450
H	-10.77400	-0.24260	-2.94320
C	-12.88010	-0.35480	-3.40800
H	-12.98320	0.71830	-3.57650
H	-13.51450	-0.84380	-4.14750
C	-13.39290	-0.69770	-2.00310
H	-13.30430	-1.77150	-1.83670
H	-12.76370	-0.21860	-1.25280
C	-14.85320	-0.26520	-1.80530
H	-15.46930	-0.71750	-2.58260
H	-14.93440	0.81470	-1.93730
C	-15.41310	-0.65660	-0.43130
H	-15.33610	-1.73650	-0.30290
H	-14.80120	-0.21210	0.35370
C	-16.87560	-0.22460	-0.25250
H	-17.47900	-0.65700	-1.05080
H	-16.95220	0.85820	-0.35930
C	-17.45540	-0.64890	1.10340
H	-17.38510	-1.73220	1.20480
H	-16.84930	-0.22870	1.90610
C	-18.91700	-0.21460	1.28150
H	-19.51880	-0.63140	0.47400
H	-18.98880	0.87010	1.19300
C	-19.50030	-0.65920	2.62940
H	-19.43590	-1.74440	2.71230
H	-18.89190	-0.25570	3.43880
C	-20.95880	-0.21980	2.81700

H	-21.02470	0.86610	2.74200
H	-21.56590	-0.62330	2.00650
C	-21.53830	-0.67710	4.16220
H	-21.48260	-1.76380	4.23120
H	-20.92200	-0.28840	4.97300
C	-22.99130	-0.22710	4.36410
H	-23.04690	0.86000	4.29900
H	-23.60790	-0.61710	3.55420
C	-23.56540	-0.68930	5.71020
H	-23.53190	-1.77760	5.77110
H	-22.93900	-0.31890	6.52170
C	-25.00600	-0.21680	5.92950
H	-25.07460	0.87020	5.89450
H	-25.37840	-0.54040	6.90170
H	-25.67290	-0.62040	5.16810
C	-14.08110	1.01700	-6.92520
H	-14.35750	1.99280	-7.32770
H	-13.39680	1.20530	-6.09730
C	-15.33350	0.30590	-6.39850
H	-16.01900	0.12340	-7.22730
H	-15.05470	-0.67380	-6.00990
C	-16.06060	1.09480	-5.30210
H	-16.34050	2.07830	-5.68220
H	-15.38490	1.26780	-4.46370
C	-17.31330	0.35620	-4.81290
H	-17.97990	0.17740	-5.65760
H	-17.02960	-0.62540	-4.43290
C	-18.07410	1.12030	-3.72350
H	-18.35410	2.10530	-4.09880
H	-17.41750	1.28850	-2.86940
C	-19.33260	0.36880	-3.26950
H	-19.98200	0.19660	-4.12880
H	-19.05100	-0.61520	-2.89480
C	-20.11510	1.12040	-2.18660
H	-20.39560	2.10560	-2.56080
H	-19.47010	1.28780	-1.32360
C	-21.37660	0.36500	-1.74810
H	-22.02070	0.19970	-2.61270
H	-21.09720	-0.62130	-1.37840
C	-22.16220	1.11150	-0.66330
H	-21.51780	1.27510	0.20100
H	-22.44130	2.09860	-1.03320
C	-23.42470	0.35730	-0.22600
H	-23.14730	-0.63230	0.13550
H	-24.07310	0.20090	-1.08900
C	-24.20120	1.09970	0.86870
H	-23.55050	1.25570	1.72970
H	-24.47920	2.09030	0.50710
C	-25.46340	0.34790	1.31150
H	-25.18730	-0.64630	1.66140
H	-26.12050	0.20200	0.45320
C	-26.22540	1.08480	2.42060
H	-25.56530	1.23110	3.27610
H	-26.50240	2.07940	2.06940
C	-27.48630	0.33500	2.87050
H	-27.21580	-0.66210	3.21800
H	-28.15230	0.19410	2.01830
C	-28.24120	1.06680	3.98570
H	-27.61610	1.19500	4.86980
H	-29.12770	0.50770	4.28620
H	-28.56790	2.05470	3.66000
O	-9.23770	3.51570	-11.31580
Si	-10.08760	4.39600	-12.48530
C	-11.64300	3.46000	-12.99870
H	-12.27950	4.07360	-13.63420
H	-11.41070	2.55510	-13.55430
H	-12.23240	3.17130	-12.12950
C	-8.97190	4.67410	-13.98100

H	-8.11990	5.30180	-13.72700
H	-8.58160	3.73920	-14.37850
H	-9.50960	5.17230	-14.78620
C	-10.60590	6.06350	-11.71330
C	-11.67510	5.80530	-10.63770
H	-11.32730	5.07840	-9.90440
H	-11.93260	6.71800	-10.10030
H	-12.59310	5.40940	-11.07230
C	-9.37110	6.71580	-11.07430
H	-8.90280	6.03950	-10.35810
H	-8.62250	6.96600	-11.82460
H	-9.62730	7.63380	-10.54550
C	-11.18030	6.98580	-12.80240
H	-10.44230	7.19500	-13.57750
H	-12.04760	6.53810	-13.28850
H	-11.49520	7.94250	-12.38500
O	-6.63850	0.98870	-11.02220
H	-6.39650	0.64150	-10.15850
O	-6.42750	1.39170	-8.59120
H	-5.94300	2.16560	-8.27940
C	-9.64340	5.27170	-7.04680
C	-8.11140	4.38940	-4.42800
O	-9.69950	4.53380	-8.02630
O	-7.92990	5.21050	-3.52730
N	-10.71170	5.60200	-6.31740
N	-8.81420	3.26510	-4.24940
H	-10.56070	6.26140	-5.55240
H	-8.95070	2.66880	-5.06730
C	-8.29650	5.78950	-6.53120
H	-8.50460	6.55740	-5.78800
C	-7.46660	6.50820	-7.62850
H	-8.12200	6.95430	-8.37640
H	-6.94320	7.34990	-7.17250
C	-6.02690	4.92230	-5.82340
H	-5.87210	5.89350	-5.34810
C	-6.42650	5.60490	-8.31140
H	-6.94740	4.85230	-8.90350
C	-5.48070	4.95110	-7.27350
H	-4.51450	5.45580	-7.28270
C	-9.36340	2.81120	-2.98230
H	-8.70100	3.09620	-2.16230
H	-9.38280	1.72200	-2.99850
C	-12.00150	4.93560	-6.36810
H	-11.84430	3.85870	-6.27820
H	-12.48090	5.11750	-7.33120
C	-7.53820	4.62260	-5.83550
H	-7.67550	3.70460	-6.40720
C	-10.77700	3.36330	-2.74920
H	-11.42330	3.07030	-3.57790
H	-10.74420	4.45400	-2.74630
C	-11.37130	2.86370	-1.42620
H	-11.36120	1.77350	-1.41820
H	-10.73080	3.18210	-0.60260
C	-12.80280	3.35840	-1.18060
H	-12.81660	4.44880	-1.18230
H	-13.44570	3.03650	-2.00120
C	-13.36650	2.83790	0.14890
H	-13.33670	1.74820	0.15240
H	-12.72210	3.16180	0.96670
C	-14.80290	3.30560	0.41640
H	-15.45040	2.97360	-0.39600
H	-14.83550	4.39530	0.41540
C	-15.34550	2.77930	1.75280
H	-15.30820	1.69020	1.75660
H	-14.69530	3.10840	2.56360
C	-16.78190	3.23980	2.03280
H	-17.43310	2.90770	1.22370
H	-16.81810	4.32920	2.03400

C	-17.31860	2.71200	3.37070
H	-17.28040	1.62280	3.37430
H	-16.66720	3.04230	4.17990
C	-18.75500	3.17270	3.65090
H	-19.40550	2.84390	2.83990
H	-18.78990	4.26200	3.65470
C	-19.29640	2.64330	4.98570
H	-19.26180	1.55400	4.98670
H	-18.64620	2.97030	5.79740
C	-20.73210	3.10920	5.26130
H	-20.76230	4.19870	5.26950
H	-21.38090	2.78700	4.44620
C	-21.28150	2.57670	6.59120
H	-21.25370	1.48720	6.58650
H	-20.63170	2.89550	7.40650
C	-22.71490	3.05100	6.86490
H	-22.73700	4.14070	6.88020
H	-23.36370	2.73990	6.04540
C	-23.27080	2.51280	8.19000
H	-23.26460	1.42260	8.17770
H	-22.61780	2.81290	9.00970
C	-24.69350	3.00550	8.47240
H	-24.72890	4.09320	8.52620
H	-25.05870	2.61600	9.42280
H	-25.38540	2.68470	7.69330
C	-12.86680	5.45000	-5.20820
H	-13.12820	6.49240	-5.39660
H	-12.27070	5.45120	-4.29530
C	-14.14130	4.63440	-4.94700
H	-14.75810	4.60320	-5.84610
H	-13.87020	3.60280	-4.71710
C	-14.94250	5.23320	-3.77990
H	-15.27030	6.23790	-4.05110
H	-14.28520	5.35310	-2.91860
C	-16.15830	4.39620	-3.35870
H	-16.82040	4.25140	-4.21340
H	-15.82960	3.40370	-3.04730
C	-16.93180	5.06760	-2.21330
H	-17.27080	6.05270	-2.53730
H	-16.25700	5.24020	-1.37520
C	-18.13760	4.25110	-1.72840
H	-18.81170	4.06750	-2.56570
H	-17.80130	3.27420	-1.37900
C	-18.90290	4.96340	-0.60330
H	-19.23290	5.94260	-0.95290
H	-18.22760	5.14940	0.23110
C	-20.11600	4.16610	-0.10510
H	-20.79110	3.97740	-0.94040
H	-19.78820	3.19070	0.25620
C	-20.87890	4.89420	1.01050
H	-20.20740	5.07460	1.84890
H	-21.19560	5.87540	0.65430
C	-22.10310	4.10980	1.50130
H	-21.78730	3.13050	1.86230
H	-22.77530	3.92930	0.66190
C	-22.86560	4.84170	2.61420
H	-22.19890	5.00930	3.45910
H	-23.16880	5.82820	2.26090
C	-24.10130	4.06650	3.08930
H	-23.79740	3.08150	3.44510
H	-24.76830	3.89850	2.24310
C	-24.86630	4.79570	4.20180
H	-24.20540	4.94900	5.05410
H	-25.15790	5.78800	3.85520
C	-26.11220	4.02510	4.65710
H	-25.82170	3.03440	5.00700
H	-26.77690	3.87060	3.80660
C	-26.88050	4.74520	5.76930

H	-26.25990	4.87370	6.65510
H	-27.76280	4.17630	6.06360
H	-27.21290	5.73240	5.44700
O	-5.65920	6.42860	-9.17100
Si	-4.75610	5.92300	-10.51340
C	-5.92380	5.75220	-11.97930
H	-6.33150	6.71720	-12.27250
H	-5.41500	5.33140	-12.84530
H	-6.76270	5.10230	-11.73850
C	-3.87460	4.28700	-10.22450
H	-3.20600	4.33830	-9.36670
H	-4.57500	3.47630	-10.04520
H	-3.27020	3.99940	-11.08320
C	-3.46160	7.28500	-10.85720
C	-2.61140	7.47910	-9.59070
H	-2.10840	6.55730	-9.29710
H	-1.85000	8.24600	-9.72770
H	-3.23260	7.78420	-8.74870
C	-2.56780	6.85830	-12.03420
H	-2.04810	5.92440	-11.81810
H	-3.15340	6.70220	-12.94090
H	-1.81190	7.61220	-12.25390
C	-4.18310	8.60090	-11.19410
H	-4.77720	8.51140	-12.10360
H	-4.85950	8.89410	-10.38950
H	-3.47560	9.41650	-11.34370
O	-5.28190	3.58610	-7.56560
H	-5.12030	3.23970	-6.68170
O	-5.25800	3.94130	-5.12180
H	-4.46860	4.39010	-4.81640
C	-10.11790	8.40570	-3.45310
C	-8.01620	8.07080	-1.34120
O	-10.26940	7.53470	-4.30740
O	-8.24660	8.89080	-0.45180
N	-11.04060	8.65930	-2.52040
N	-8.16520	6.75050	-1.16930
H	-10.81920	9.36050	-1.82790
H	-7.99380	6.14990	-1.97840
C	-8.84380	9.26890	-3.41960
H	-9.07890	10.15780	-2.83120
C	-8.47640	9.74120	-4.84120
H	-8.20900	8.88630	-5.46300
H	-9.33640	10.20880	-5.32260
C	-6.39950	9.51590	-2.70160
H	-6.58820	10.31920	-1.98590
C	-7.30440	10.72850	-4.80630
H	-7.03020	10.99520	-5.82830
C	-6.07710	10.12970	-4.08370
H	-5.29020	10.87660	-3.98910
C	-8.61080	6.10830	0.05980
H	-8.05170	6.51620	0.90400
H	-8.36600	5.04740	0.00160
C	-12.28860	7.92700	-2.37040
H	-12.07150	6.86500	-2.24490
H	-12.88340	8.02700	-3.27990
C	-7.63020	8.55890	-2.74870
H	-7.37010	7.69230	-3.35640
C	-10.12330	6.29580	0.26970
H	-10.66640	5.86600	-0.57210
H	-10.34880	7.36230	0.27010
C	-10.63920	5.68720	1.58070
H	-10.53380	4.60210	1.55110
H	-10.02020	6.03740	2.40800
C	-12.10220	6.06950	1.85100
H	-12.18850	7.15660	1.86350
H	-12.72950	5.72190	1.03030
C	-12.63640	5.51270	3.17740
H	-12.60140	4.42290	3.15820

H	-11.98380	5.82820	3.99270
C	-14.06980	5.98210	3.46510
H	-14.72440	5.67390	2.64970
H	-14.09560	7.07240	3.48310
C	-14.61740	5.44160	4.79290
H	-14.60350	4.35120	4.77560
H	-13.95860	5.74500	5.60770
C	-16.04310	5.93350	5.08020
H	-16.70100	5.63670	4.26320
H	-16.05300	7.02400	5.10220
C	-16.59840	5.39250	6.40480
H	-16.58830	4.30210	6.38470
H	-15.94130	5.69100	7.22290
C	-18.02390	5.88610	6.68910
H	-18.67930	5.59290	5.86880
H	-18.03230	6.97640	6.71540
C	-18.58360	5.33910	8.00950
H	-18.57350	4.24880	7.98400
H	-17.92900	5.63350	8.83090
C	-20.01000	5.83050	8.29250
H	-20.01880	6.92070	8.32580
H	-20.66280	5.54240	7.46820
C	-20.57330	5.27420	9.60750
H	-20.56340	4.18420	9.57390
H	-19.92080	5.56230	10.43280
C	-22.00030	5.76380	9.89040
H	-22.00780	6.85360	9.93570
H	-22.64990	5.48600	9.06010
C	-22.56940	5.19360	11.19710
H	-22.56690	4.10370	11.15480
H	-21.92220	5.47030	12.03040
C	-23.99360	5.68270	11.48080
H	-24.02820	6.76900	11.56730
H	-24.37080	5.26380	12.41400
H	-24.67810	5.38760	10.68580
C	-13.06240	8.46200	-1.15960
H	-13.29130	9.51740	-1.31510
H	-12.42730	8.41410	-0.27450
C	-14.36370	7.69890	-0.88040
H	-15.02110	7.76030	-1.74880
H	-14.14450	6.64120	-0.73110
C	-15.08180	8.25600	0.35620
H	-15.29480	9.31490	0.20230
H	-14.41330	8.20180	1.21630
C	-16.38690	7.52200	0.68650
H	-17.06610	7.57960	-0.16520
H	-16.18140	6.46400	0.84930
C	-17.06610	8.11210	1.92980
H	-17.25840	9.17350	1.76650
H	-16.38340	8.05190	2.77820
C	-18.38140	7.40960	2.28610
H	-19.06700	7.46810	1.43960
H	-18.19230	6.35030	2.45910
C	-19.04410	8.02230	3.52740
H	-19.22380	9.08440	3.35520
H	-18.35830	7.96000	4.37310
C	-20.36650	7.33630	3.89150
H	-21.05250	7.39760	3.04560
H	-20.18730	6.27580	4.06780
C	-21.02530	7.95650	5.13100
H	-20.34080	7.89120	5.97740
H	-21.19830	9.01930	4.95670
C	-22.35240	7.27700	5.49270
H	-22.17920	6.21510	5.66630
H	-23.03670	7.34350	4.64570
C	-23.01190	7.89550	6.73270
H	-22.33020	7.82320	7.58080
H	-23.17910	8.95990	6.56220

C	-24.34380	7.22050	7.08720
H	-24.17740	6.15620	7.25320
H	-25.02610	7.29680	6.23930
C	-25.00230	7.83370	8.33060
H	-24.32240	7.75100	9.17920
H	-25.16210	8.90050	8.16760
C	-26.33970	7.16500	8.67840
H	-26.18670	6.09790	8.84100
H	-27.02390	7.25170	7.83320
C	-26.99490	7.77380	9.92300
H	-26.35330	7.67120	10.79850
H	-27.94040	7.27880	10.14590
H	-27.20160	8.83510	9.78160
O	-7.77920	11.89760	-4.16070
Si	-6.95410	13.36500	-4.01650
C	-5.81900	13.59210	-5.50640
H	-5.25460	14.52070	-5.43650
H	-5.10640	12.77500	-5.60270
H	-6.39080	13.62920	-6.43270
C	-5.98770	13.33960	-2.39610
H	-6.64970	13.18120	-1.54570
H	-5.24350	12.54850	-2.37690
H	-5.46570	14.28110	-2.23220
C	-8.26980	14.74520	-3.96150
C	-9.08870	14.70610	-5.26310
H	-9.56350	13.73340	-5.40160
H	-9.87690	15.45940	-5.26140
H	-8.46290	14.89060	-6.13650
C	-9.20080	14.50200	-2.76120
H	-9.67670	13.52220	-2.82320
H	-8.65680	14.54090	-1.81710
H	-9.99250	15.25010	-2.71250
C	-7.57730	16.11140	-3.81960
H	-6.98740	16.16700	-2.90420
H	-6.90500	16.30670	-4.65570
H	-8.30470	16.92320	-3.78900
O	-5.52880	9.05250	-4.80960
H	-5.32000	8.45570	-4.08740
O	-5.21600	8.81000	-2.32290
H	-4.53260	9.43640	-2.08340
C	0.63040	-0.63960	-11.41010
C	-0.96280	1.86980	-11.84170
O	0.56440	-1.39090	-12.38210
O	-0.72820	2.70990	-10.97330
N	1.77500	-0.03690	-11.06250
N	-0.62180	2.04850	-13.12320
H	1.78270	0.61980	-10.28320
H	-0.83870	1.29780	-13.76300
C	-0.65090	-0.32840	-10.61050
H	-0.39070	0.22350	-9.70480
C	-1.32180	-1.66200	-10.20450
H	-1.50100	-2.26420	-11.09750
H	-0.64730	-2.24950	-9.58070
C	-2.99880	0.74640	-10.80300
H	-2.89970	1.47000	-9.99020
C	-2.66650	-1.48560	-9.48550
H	-3.14500	-2.46410	-9.43740
C	-3.62740	-0.54660	-10.23660
H	-4.45320	-0.27370	-9.58140
C	0.05510	3.21860	-13.66080
H	-0.41010	4.12690	-13.27310
H	-0.10010	3.22570	-14.74000
C	3.01130	-0.18300	-11.82440
H	2.79090	-0.11250	-12.89140
H	3.42310	-1.17840	-11.65170
C	-1.62020	0.52390	-11.48100
H	-1.79890	-0.03060	-12.40450
C	1.56080	3.20130	-13.34380

H	1.98700	2.24060	-13.63610
H	1.70900	3.29020	-12.26610
C	2.30860	4.32910	-14.06510
H	2.16430	4.21730	-15.14070
H	1.86150	5.28620	-13.79640
C	3.81370	4.36050	-13.75950
H	3.97100	4.52920	-12.69400
H	4.25540	3.38960	-13.98810
C	4.52710	5.44980	-14.57150
H	4.38400	5.25100	-15.63460
H	4.05320	6.41210	-14.37730
C	6.03020	5.55800	-14.27770
H	6.50860	4.59350	-14.45140
H	6.18470	5.79890	-13.22560
C	6.69480	6.62570	-15.15790
H	6.54860	6.36400	-16.20690
H	6.18880	7.58000	-15.01010
C	8.19520	6.80340	-14.88780
H	8.70640	5.84940	-15.02060
H	8.34970	7.10000	-13.85020
C	8.81520	7.85120	-15.82300
H	8.66470	7.54100	-16.85800
H	8.28370	8.79620	-15.70800
C	10.31260	8.07990	-15.57730
H	10.84800	7.13590	-15.68330
H	10.46830	8.41450	-14.55160
C	10.89920	9.11220	-16.55030
H	10.74920	8.76750	-17.57430
H	10.34740	10.04820	-16.45910
C	12.39250	9.37890	-16.31880
H	12.54480	9.73790	-15.30090
H	12.94700	8.44410	-16.40620
C	12.95740	10.40250	-17.31340
H	12.81460	10.03520	-18.33050
H	12.38860	11.32980	-17.23980
C	14.44520	10.69890	-17.08360
H	14.58880	11.07020	-16.06890
H	15.01640	9.77310	-17.15960
C	14.99690	11.72310	-18.08470
H	14.86850	11.34860	-19.10110
H	14.41770	12.64470	-18.02240
C	16.47640	12.04180	-17.84740
H	16.63640	12.44980	-16.85000
H	16.83590	12.77860	-18.56620
H	17.09340	11.14910	-17.95020
C	4.03250	0.89680	-11.44050
H	4.31380	0.77270	-10.39400
H	3.56280	1.87710	-11.52270
C	5.29420	0.87740	-12.31340
H	5.78110	-0.09550	-12.23410
H	5.00800	0.99590	-13.35940
C	6.28300	1.98630	-11.92340
H	6.61010	1.83700	-10.89360
H	5.77300	2.94930	-11.94840
C	7.50540	2.04490	-12.84800
H	8.02750	1.08720	-12.82650
H	7.17190	2.19070	-13.87650
C	8.47790	3.16910	-12.46240
H	8.84780	3.00240	-11.44980
H	7.94480	4.11960	-12.44250
C	9.66070	3.27620	-13.43310
H	10.20240	2.32950	-13.45420
H	9.28240	3.43630	-14.44380
C	10.62710	4.41090	-13.06580
H	11.03550	4.23730	-12.06930
H	10.07980	5.35200	-13.01420
C	11.77200	4.54580	-14.07820
H	12.32540	3.60700	-14.12870

H	11.35500	4.71050	-15.07280
C	12.73620	5.68960	-13.73590
H	12.18030	6.62450	-13.66850
H	13.17270	5.51910	-12.75080
C	13.85290	5.83610	-14.77810
H	13.40950	5.99900	-15.76160
H	14.41280	4.90220	-14.84410
C	14.81580	6.98700	-14.45780
H	14.25490	7.91860	-14.38730
H	15.26740	6.82470	-13.47810
C	15.91770	7.13530	-15.51570
H	15.46160	7.29530	-16.49380
H	16.48010	6.20320	-15.58670
C	16.88020	8.28990	-15.20760
H	16.31760	9.22090	-15.14290
H	17.33400	8.13560	-14.22760
C	17.98090	8.43310	-16.26750
H	17.52820	8.59300	-17.24710
H	18.54620	7.50280	-16.33740
C	18.94280	9.58580	-15.96220
H	18.41550	10.53850	-15.93480
H	19.71830	9.65930	-16.72490
H	19.43500	9.44770	-14.99910
O	-2.45830	-1.04600	-8.15690
Si	-2.86110	-1.94380	-6.77430
C	-1.65880	-1.50390	-5.39490
H	-1.70920	-2.21790	-4.57500
H	-0.63370	-1.47660	-5.75970
H	-1.89810	-0.52330	-4.98890
C	-2.68600	-3.78910	-7.13940
H	-3.42330	-4.13110	-7.86470
H	-1.69960	-4.02260	-7.53740
H	-2.82640	-4.38290	-6.23770
C	-4.64460	-1.54730	-6.19880
C	-5.66360	-2.06910	-7.22480
H	-5.57530	-3.14470	-7.37830
H	-6.68400	-1.87240	-6.89920
H	-5.54470	-1.59360	-8.19520
C	-4.90400	-2.23150	-4.84530
H	-4.79140	-3.31370	-4.91970
H	-4.20960	-1.88060	-4.08120
H	-5.91360	-2.02910	-4.48710
C	-4.79260	-0.02440	-6.04410
H	-4.14260	0.36640	-5.26080
H	-4.51870	0.48800	-6.96650
H	-5.81610	0.26090	-5.79890
O	-4.17740	-1.23220	-11.34140
H	-4.21220	-0.54620	-12.01010
O	-3.91900	1.23210	-11.78970
H	-4.79390	1.31090	-11.40570
C	2.49770	2.83820	-8.21540
C	0.41900	4.95400	-8.40160
O	2.35720	2.01990	-9.12130
O	0.80920	5.93750	-7.77130
N	3.55320	3.64970	-8.10700
N	0.32120	4.96270	-9.73660
H	3.54470	4.30470	-7.33880
H	-0.03720	4.11350	-10.18210
C	1.42390	2.98530	-7.13920
H	1.82340	3.63800	-6.35950
C	1.13260	1.61510	-6.50170
H	0.67040	0.95360	-7.23720
H	2.05780	1.12790	-6.19610
C	-0.85540	3.81880	-6.48770
H	-0.45350	4.56960	-5.80950
C	0.19040	1.76780	-5.30390
H	-0.06630	0.78150	-4.92160
C	-1.09470	2.52140	-5.68700

H	-1.65320	2.75020	-4.78300
C	0.71830	6.05980	-10.60890
H	0.17930	6.96150	-10.32110
H	0.40430	5.81620	-11.62340
C	4.66070	3.71020	-9.04320
H	4.27870	3.70730	-10.06530
H	5.27360	2.81510	-8.92770
C	0.11660	3.62680	-7.67720
H	-0.33860	2.93860	-8.39050
C	2.23870	6.29620	-10.57270
H	2.75080	5.37210	-10.83880
H	2.54840	6.53370	-9.55490
C	2.70680	7.42720	-11.49580
H	2.49790	7.16510	-12.53270
H	2.13610	8.33190	-11.28730
C	4.20410	7.71280	-11.31920
H	4.39550	8.01330	-10.28790
H	4.76600	6.79290	-11.48110
C	4.72740	8.79570	-12.26950
H	4.56200	8.48580	-13.30150
H	4.15600	9.71350	-12.13120
C	6.21990	9.07720	-12.05180
H	6.78090	8.14860	-12.15810
H	6.38080	9.42040	-11.02880
C	6.77450	10.11480	-13.03490
H	6.62540	9.76360	-14.05620
H	6.20730	11.04120	-12.94570
C	8.26510	10.39750	-12.80600
H	8.82590	9.46600	-12.88320
H	8.41520	10.76630	-11.79060
C	8.82780	11.41080	-13.81010
H	8.68650	11.03410	-14.82350
H	8.25810	12.33800	-13.74780
C	10.31560	11.70500	-13.57930
H	10.88230	10.77570	-13.63890
H	10.45880	12.09120	-12.56950
C	10.87480	12.70670	-14.59720
H	10.74000	12.31330	-15.60510
H	10.29760	13.63030	-14.55080
C	12.35920	13.01750	-14.36640
H	12.49550	13.41560	-13.36040
H	12.93520	12.09340	-14.41440
C	12.91120	14.01510	-15.39280
H	12.78430	13.61040	-16.39710
H	12.32380	14.93270	-15.35720
C	14.39090	14.34510	-15.15880
H	14.51720	14.75010	-14.15430
H	14.97820	13.42770	-15.19810
C	14.93640	15.34480	-16.18710
H	14.83140	14.93460	-17.19200
H	14.33690	16.25490	-16.16480
C	16.40530	15.70130	-15.93910
H	16.54520	16.12780	-14.94620
H	16.75690	16.43330	-16.66640
H	17.04320	14.82150	-16.01950
C	5.49160	4.97560	-8.79220
H	5.85900	4.97550	-7.76480
H	4.85570	5.85610	-8.89320
C	6.67820	5.09280	-9.75440
H	7.31950	4.21700	-9.64490
H	6.31450	5.07960	-10.78230
C	7.50340	6.36530	-9.52110
H	7.85500	6.39050	-8.48860
H	6.86900	7.24290	-9.65150
C	8.70330	6.44970	-10.47200
H	9.33590	5.57130	-10.33520
H	8.35130	6.41480	-11.50320
C	9.54070	7.71720	-10.26200

H	9.88690	7.75930	-9.22840
H	8.91480	8.59730	-10.41370
C	10.74680	7.77050	-11.20880
H	11.37160	6.89030	-11.05050
H	10.39870	7.71670	-12.24040
C	11.59310	9.03570	-11.02220
H	11.94190	9.09070	-9.99030
H	10.97060	9.91600	-11.18550
C	12.79860	9.07430	-11.97100
H	13.42320	8.19620	-11.80110
H	12.44940	9.00780	-13.00130
C	13.64410	10.34290	-11.80060
H	13.01850	11.21980	-11.97030
H	13.99560	10.40840	-10.77040
C	14.84760	10.37990	-12.75200
H	14.49710	10.30260	-13.78110
H	15.47810	9.50740	-12.57510
C	15.68410	11.65630	-12.59360
H	15.05150	12.52690	-12.76940
H	16.03630	11.73280	-11.56430
C	16.88670	11.69740	-13.54620
H	16.53670	11.60900	-14.57460
H	17.52540	10.83220	-13.36290
C	17.71100	12.98330	-13.39730
H	17.06980	13.84630	-13.57910
H	18.06180	13.07000	-12.36830
C	18.91380	13.03070	-14.34980
H	18.57060	12.94160	-15.38080
H	19.56100	12.17220	-14.16590
C	19.73120	14.31910	-14.20390
H	19.12470	15.19990	-14.41630
H	20.57440	14.32430	-14.89500
H	20.13040	14.42220	-13.19460
O	0.84200	2.48350	-4.27350
Si	1.60710	1.77810	-2.94540
C	2.92330	0.57810	-3.56410
H	3.48260	0.14300	-2.73720
H	2.48140	-0.24190	-4.12830
H	3.63820	1.07890	-4.21520
C	0.32740	0.85020	-1.91650
H	-0.39770	1.52950	-1.47380
H	-0.22410	0.12820	-2.51710
H	0.79830	0.30660	-1.09900
C	2.41040	3.17830	-1.93030
C	3.48150	3.86860	-2.79210
H	3.05430	4.24030	-3.72450
H	3.92500	4.71850	-2.27350
H	4.28770	3.18330	-3.05440
C	1.32150	4.19340	-1.54890
H	0.83360	4.59480	-2.43730
H	0.54790	3.73250	-0.93720
H	1.73030	5.03480	-0.98950
C	3.05250	2.59420	-0.66070
H	2.31150	2.09890	-0.03210
H	3.82010	1.85900	-0.90420
H	3.52270	3.37340	-0.06020
O	-1.92160	1.72330	-6.49820
H	-2.36800	2.36370	-7.04840
O	-2.13780	4.23950	-6.92560
H	-2.56150	4.62520	-6.16090
C	1.29160	7.17470	-4.53570
C	-0.55590	8.97220	-6.43550
O	1.30570	5.96130	-4.71770
O	-0.88130	10.14090	-6.22380
N	2.37520	7.94090	-4.66410
N	0.19940	8.62820	-7.48560
H	2.28410	8.92120	-4.39730
H	0.42770	7.63560	-7.59130

C	-0.01660	7.90590	-4.21760
H	0.22740	8.94780	-4.02560
C	-0.64670	7.40540	-2.89530
H	0.12560	7.05590	-2.20800
H	-1.12890	8.24180	-2.38960
C	-2.43970	7.95700	-4.99750
H	-2.57840	8.91400	-4.49150
C	-1.69420	6.31160	-3.09390
H	-1.20480	5.42410	-3.50000
C	-2.82040	6.80070	-4.03050
H	-3.68640	7.11570	-3.45420
C	0.73930	9.54620	-8.48130
H	0.11310	10.43620	-8.56560
H	0.69550	9.05360	-9.45130
C	3.61690	7.52420	-5.28960
H	3.39240	7.12390	-6.28050
H	4.07790	6.72200	-4.71080
C	-0.96470	7.85260	-5.45480
H	-0.85630	6.89760	-5.96980
C	2.19150	9.93690	-8.15680
H	2.79870	9.03350	-8.07990
H	2.22470	10.41990	-7.17920
C	2.79750	10.87400	-9.21480
H	2.71980	10.40570	-10.19610
H	2.20290	11.78650	-9.26680
C	4.26840	11.23540	-8.94620
H	4.35570	11.70090	-7.96400
H	4.86610	10.32340	-8.91140
C	4.84090	12.18180	-10.01530
H	4.74390	11.71940	-10.99740
H	4.24120	13.09150	-10.05050
C	6.31330	12.55430	-9.77820
H	6.91950	11.64790	-9.76390
H	6.41780	13.01340	-8.79480
C	6.85490	13.51500	-10.85020
H	6.74620	13.05970	-11.83430
H	6.24630	14.41920	-10.86700
C	8.32620	13.89890	-10.63100
H	8.93890	12.99710	-10.62370
H	8.43700	14.35770	-9.64840
C	8.85220	14.86400	-11.70560
H	8.73870	14.40970	-12.68970
H	8.24000	15.76590	-11.71500
C	10.32320	15.25060	-11.49230
H	10.93720	14.34960	-11.48420
H	10.43610	15.71190	-10.51130
C	10.84730	16.21340	-12.56840
H	10.73530	15.75680	-13.55170
H	10.23430	17.11480	-12.57930
C	12.31750	16.59960	-12.35100
H	12.42740	17.06380	-11.37120
H	12.93150	15.69860	-12.33800
C	12.84480	17.55900	-13.42730
H	12.73810	17.09880	-14.40950
H	12.23050	18.45950	-13.44370
C	14.31270	17.94750	-13.20230
H	14.41620	18.41620	-12.22370
H	14.92830	17.04770	-13.18070
C	14.84220	18.90310	-14.28030
H	14.75360	18.43810	-15.26220
H	14.22340	19.79990	-14.31090
C	16.30130	19.30620	-14.04390
H	16.42080	19.80810	-13.08430
H	16.64590	19.99100	-14.81900
H	16.95880	18.43680	-14.05210
C	4.54950	8.73680	-5.39730
H	4.87530	9.02500	-4.39650
H	3.98880	9.58660	-5.78750

C	5.77030	8.51050	-6.29790
H	6.34350	7.65160	-5.94610
H	5.43610	8.27110	-7.30880
C	6.66120	9.75960	-6.32570
H	7.05940	9.93570	-5.32520
H	6.05200	10.63150	-6.56520
C	7.81750	9.67710	-7.32940
H	8.43040	8.79930	-7.12020
H	7.42110	9.54760	-8.33760
C	8.68370	10.94230	-7.26820
H	9.09680	11.04620	-6.26370
H	8.05510	11.81840	-7.42830
C	9.82690	10.95170	-8.28920
H	10.45780	10.07440	-8.14100
H	9.41920	10.87570	-9.29800
C	10.67620	12.22370	-8.16730
H	11.07980	12.29390	-7.15620
H	10.03910	13.09760	-8.30140
C	11.82790	12.27520	-9.17760
H	12.46770	11.40250	-9.04250
H	11.42880	12.21450	-10.19080
C	12.66610	13.55160	-9.02990
H	12.02570	14.42190	-9.16920
H	13.05390	13.61650	-8.01240
C	13.83160	13.61200	-10.02430
H	13.44580	13.54790	-11.04220
H	14.47390	12.74260	-9.88040
C	14.66410	14.89140	-9.87190
H	14.02430	15.75890	-10.02890
H	15.03660	14.96490	-8.84920
C	15.84340	14.94420	-10.85080
H	15.47050	14.86960	-11.87270
H	16.48410	14.07660	-10.69020
C	16.67500	16.22480	-10.70360
H	16.03770	17.09080	-10.87920
H	17.03600	16.31060	-9.67780
C	17.86460	16.26290	-11.67090
H	17.50430	16.17870	-12.69640
H	18.50550	15.39820	-11.49580
C	18.69730	17.54080	-11.53390
H	18.09730	18.42410	-11.74820
H	19.53700	17.53450	-12.22930
H	19.10010	17.64580	-10.52610
O	-2.20390	6.02170	-1.80270
Si	-3.31250	4.80810	-1.39530
C	-2.87270	3.23000	-2.31780
H	-1.79390	3.10530	-2.38520
H	-3.28190	2.34810	-1.82850
H	-3.27370	3.24800	-3.32810
C	-5.06790	5.35770	-1.80190
H	-5.30790	6.29930	-1.31090
H	-5.22270	5.49790	-2.86810
H	-5.80290	4.62400	-1.47580
C	-3.17230	4.54510	0.49190
C	-3.47940	5.87510	1.20180
H	-4.49410	6.21540	0.99230
H	-3.38060	5.78350	2.28350
H	-2.79960	6.66280	0.87310
C	-4.17620	3.47040	0.94480
H	-5.20200	3.76120	0.71610
H	-3.98920	2.51520	0.45380
H	-4.11730	3.30160	2.02030
C	-1.74110	4.09830	0.82980
H	-1.48870	3.16170	0.33140
H	-1.01330	4.84660	0.51300
H	-1.61130	3.94640	1.90150
O	-3.19640	5.76340	-4.90330
H	-3.34720	6.30890	-5.69050

O	-3.35860	7.88220	-6.09390
H	-4.11880	8.43080	-5.88100
C	1.87210	11.69520	-3.23890
C	-0.20930	13.26360	-4.70450
O	2.03380	10.54040	-3.62760
O	-0.02480	14.43970	-4.39540
N	2.80950	12.63220	-3.41570
N	-0.05340	12.80820	-5.95280
H	2.60480	13.57450	-3.11530
H	-0.27290	11.82450	-6.12600
C	0.54620	12.11540	-2.57420
H	0.69570	13.08430	-2.09390
C	0.15080	11.09970	-1.48110
H	0.04580	10.10880	-1.91870
H	0.94590	11.01530	-0.73920
C	-1.94150	12.64160	-2.95610
H	-1.88320	13.67220	-2.59750
C	-1.16440	11.47580	-0.78350
H	-1.44640	10.67650	-0.09530
C	-2.32310	11.73260	-1.76830
H	-3.15700	12.18760	-1.23490
C	0.41230	13.60810	-7.07780
H	-0.16370	14.53410	-7.12690
H	0.20960	13.06140	-7.99930
C	4.04950	12.39000	-4.14370
H	3.81250	11.97270	-5.12420
H	4.64040	11.63980	-3.61510
C	-0.59660	12.24430	-3.62070
H	-0.72550	11.26460	-4.07400
C	1.91530	13.91510	-6.96080
H	2.48040	12.98330	-6.94490
H	2.10930	14.40630	-6.00690
C	2.43440	14.81870	-8.08560
H	2.32210	14.31500	-9.04620
H	1.82190	15.72020	-8.13510
C	3.90040	15.21710	-7.86710
H	3.99810	15.70790	-6.89800
H	4.51950	14.32110	-7.82100
C	4.43280	16.15460	-8.95750
H	4.35430	15.66700	-9.92990
H	3.80620	17.04620	-9.00830
C	5.88780	16.56890	-8.70090
H	6.51480	15.67880	-8.64530
H	5.95960	17.05660	-7.72800
C	6.43280	17.51150	-9.78100
H	6.35750	17.02940	-10.75630
H	5.80990	18.40570	-9.83070
C	7.88930	17.91700	-9.51850
H	8.51080	17.02280	-9.46430
H	7.96170	18.40190	-8.54420
C	8.44020	18.85760	-10.59770
H	8.36140	18.37640	-11.57330
H	7.82250	19.75540	-10.64670
C	9.89950	19.25520	-10.33860
H	10.51630	18.35760	-10.28690
H	9.97710	19.73920	-9.36420
C	10.45140	20.19320	-11.42020
H	10.36760	19.71110	-12.39480
H	9.83680	21.09310	-11.46840
C	11.91310	20.58610	-11.16740
H	11.99580	21.07520	-10.19600
H	12.52570	19.68590	-11.11220
C	12.46670	21.51460	-12.25670
H	12.37840	21.02600	-13.22770
H	11.85610	22.41700	-12.31020
C	13.93080	21.90300	-12.01010
H	14.01530	22.40690	-11.04640
H	14.53780	21.00020	-11.93920

C	14.49180	22.81040	-13.11390
H	14.41300	22.30760	-14.07870
H	13.88630	23.71470	-13.18800
C	15.95300	23.20060	-12.86830
H	16.06550	23.73770	-11.92610
H	16.32280	23.84700	-13.66460
H	16.59550	22.32110	-12.83180
C	4.85460	13.68410	-4.31140
H	5.13110	14.06880	-3.32860
H	4.22780	14.44620	-4.77570
C	6.12110	13.48880	-5.15970
H	6.75190	12.72160	-4.70830
H	5.84650	13.11930	-6.14850
C	6.91800	14.79260	-5.30190
H	7.19640	15.15160	-4.30990
H	6.27720	15.56170	-5.73460
C	8.18310	14.64810	-6.16020
H	8.82700	13.87380	-5.74070
H	7.91230	14.31630	-7.16280
C	8.95620	15.97180	-6.24430
H	9.21810	16.29810	-5.23680
H	8.30460	16.74410	-6.65450
C	10.23230	15.88450	-7.09220
H	10.88730	15.10950	-6.69160
H	9.97670	15.58020	-8.10700
C	10.98120	17.22440	-7.12820
H	11.22940	17.52560	-6.10950
H	10.32040	17.99710	-7.52270
C	12.26440	17.17760	-7.96790
H	12.92780	16.40380	-7.57900
H	12.01880	16.88970	-8.98980
C	12.99800	18.52630	-7.97710
H	12.33170	19.29830	-8.36350
H	13.23890	18.81390	-6.95280
C	14.28450	18.49900	-8.81300
H	14.04400	18.21700	-9.83780
H	14.95220	17.72680	-8.42860
C	15.01110	19.85130	-8.81250
H	14.34340	20.62210	-9.19890
H	15.24700	20.13550	-7.78610
C	16.30100	19.82950	-9.64390
H	16.06600	19.54510	-10.66950
H	16.97020	19.06010	-9.25650
C	17.02350	21.18390	-9.64310
H	16.35550	21.95180	-10.03500
H	17.25320	21.47160	-8.61610
C	18.31790	21.16210	-10.46800
H	18.09430	20.87710	-11.49640
H	18.98970	20.39720	-10.07630
C	19.03810	22.51490	-10.46700
H	18.40860	23.29890	-10.88850
H	19.95170	22.46930	-11.06040
H	19.31440	22.81460	-9.45560
O	-0.93580	12.65870	-0.04450
Si	-1.55480	12.95790	1.49830
C	-0.78130	11.72400	2.69290
H	-1.09920	11.91050	3.71770
H	-1.05660	10.69960	2.44630
H	0.30600	11.78370	2.67210
C	-3.42990	12.76190	1.46140
H	-3.89130	13.44860	0.75310
H	-3.72410	11.75060	1.18310
H	-3.86580	12.96670	2.43840
C	-1.07220	14.74490	1.95580
C	-1.67460	15.70450	0.91520
H	-2.76370	15.65170	0.90540
H	-1.39940	16.73930	1.12100
H	-1.32680	15.46540	-0.09100

C	-1.61290	15.08200	3.35570
H	-2.69960	14.99900	3.39430
H	-1.20610	14.41060	4.11280
H	-1.35230	16.09980	3.64730
C	0.46120	14.86620	1.94250
H	0.92080	14.20430	2.67720
H	0.86950	14.60250	0.96570
H	0.78330	15.88250	2.17090
O	-2.82630	10.55040	-2.35140
H	-3.06840	10.89140	-3.21590
O	-3.01380	12.52010	-3.89540
H	-2.90200	13.19430	-4.55540

5-alpha monomer

C	33.05780000	-9.57780000	-17.43180000
C	30.30480000	-10.81810000	-17.11620000
O	32.93180000	-8.50750000	-16.84360000
O	30.44670000	-12.03610000	-17.22510000
N	33.91030000	-10.52050000	-17.01740000
N	29.80370000	-10.24910000	-16.01100000
H	33.95750000	-11.40300000	-17.52320000
H	29.70930000	-9.23220000	-16.01680000
C	32.17790000	-9.85720000	-18.65820000
H	32.32640000	-8.98350000	-19.29520000
C	30.66180000	-9.87940000	-18.28890000
H	30.39650000	-8.88270000	-17.95060000
C	32.62500000	-11.08380000	-19.49090000
H	33.67790000	-10.99460000	-19.76380000
H	32.52790000	-11.99310000	-18.90560000
C	29.77640000	-10.16040000	-19.52780000
H	28.75550000	-10.38420000	-19.21080000
C	31.77510000	-11.18640000	-20.75880000
H	31.93850000	-10.28120000	-21.34800000
C	30.28300000	-11.32170000	-20.41060000
H	30.09390000	-12.27710000	-19.91740000
C	29.21040000	-10.95810000	-14.88290000
H	28.38170000	-10.35160000	-14.51680000
H	28.77060000	-11.89890000	-15.21940000
C	34.73080000	-10.44600000	-15.81960000
H	35.59360000	-11.09490000	-15.97450000
H	35.11750000	-9.43550000	-15.67340000
C	30.20660000	-11.21420000	-13.73800000
H	31.03100000	-11.82950000	-14.10010000
H	30.64210000	-10.26930000	-13.40980000
C	29.52040000	-11.91110000	-12.54930000
H	28.68210000	-11.30120000	-12.21350000
H	29.08860000	-12.85280000	-12.88890000
C	30.44350000	-12.18440000	-11.35110000
H	30.88210000	-11.24880000	-11.00360000
H	31.27230000	-12.81760000	-11.66610000
C	29.69000000	-12.86100000	-10.19340000
H	28.85480000	-12.23050000	-9.88950000
H	29.24900000	-13.79400000	-10.54480000
C	30.56940000	-13.15070000	-8.96790000
H	30.99870000	-12.22020000	-8.59760000
H	31.40730000	-13.78180000	-9.26170000
C	29.78370000	-13.83890000	-7.84090000
H	28.92790000	-13.22190000	-7.56720000
H	29.37320000	-14.77990000	-8.20850000
C	30.63190000	-14.10960000	-6.58990000
H	31.02780000	-13.16920000	-6.20760000
H	31.49400000	-14.71940000	-6.85850000
C	29.83370000	-14.81660000	-5.48510000
H	28.95030000	-14.22590000	-5.24160000
H	29.46760000	-15.77330000	-5.85950000

C	30.65590000	-15.04840000	-4.20920000
H	31.00830000	-14.09150000	-3.82510000
H	31.54610000	-15.62950000	-4.44900000
C	29.85360000	-15.77320000	-3.12000000
H	28.94330000	-15.21200000	-2.90630000
H	29.53290000	-16.74620000	-3.49450000
C	30.65030000	-15.96200000	-1.82130000
H	30.95960000	-14.98910000	-1.43990000
H	31.56620000	-16.51430000	-2.03110000
C	29.84550000	-16.70150000	-0.74420000
H	28.91250000	-16.16810000	-0.55860000
H	29.56650000	-17.68830000	-1.11590000
C	30.61690000	-16.85050000	0.57460000
H	30.88480000	-15.86390000	0.95230000
H	31.55590000	-17.37320000	0.39170000
C	29.81150000	-17.60600000	1.64000000
H	28.85890000	-17.10180000	1.80630000
H	29.57030000	-18.60480000	1.27390000
C	30.55950000	-17.72400000	2.97270000
H	30.76860000	-16.74350000	3.39940000
H	29.97070000	-18.28260000	3.70070000
H	31.51010000	-18.24190000	2.84850000
C	33.93290000	-10.91140000	-14.59340000
H	33.42460000	-11.84620000	-14.83060000
H	33.14830000	-10.18580000	-14.37330000
C	34.81660000	-11.11300000	-13.35680000
H	35.62320000	-11.80650000	-13.59900000
H	35.29010000	-10.16810000	-13.08670000
C	34.02360000	-11.65790000	-12.16350000
H	33.52770000	-12.58470000	-12.45370000
H	33.23300000	-10.95350000	-11.90310000
C	34.90660000	-11.91600000	-10.93580000
H	35.70450000	-12.61270000	-11.19700000
H	35.39300000	-10.98690000	-10.63580000
C	34.10410000	-12.48010000	-9.75770000
H	33.64160000	-13.42160000	-10.05500000
H	33.28780000	-11.79790000	-9.51880000
C	34.96240000	-12.70210000	-8.50510000
H	35.78600000	-13.37810000	-8.73810000
H	35.41510000	-11.75670000	-8.20340000
C	34.14490000	-13.27430000	-7.34030000
H	33.72280000	-14.23490000	-7.63540000
H	33.29930000	-12.61720000	-7.13580000
C	34.97170000	-13.44870000	-6.05910000
H	35.82810000	-14.09350000	-6.25900000
H	35.37790000	-12.48310000	-5.75520000
C	34.14250000	-14.04120000	-4.91210000
H	33.76430000	-15.01900000	-5.20990000
H	33.26750000	-13.41480000	-4.73680000
C	34.93930000	-14.17660000	-3.60740000
H	35.82610000	-14.78680000	-3.78100000
H	35.29830000	-13.19420000	-3.29810000
C	34.10540000	-14.79820000	-2.47890000
H	33.77170000	-15.79020000	-2.78230000
H	33.20330000	-14.20560000	-2.32490000
C	34.87810000	-14.90150000	-1.15680000
H	35.79120000	-15.47710000	-1.31100000
H	35.19290000	-13.90570000	-0.84220000
C	34.04730000	-15.55340000	-0.04320000
H	33.75260000	-16.55600000	-0.35210000
H	33.12270000	-14.99290000	0.09730000
C	34.80480000	-15.63220000	1.28920000
H	35.73630000	-16.18150000	1.15160000
H	35.08680000	-14.62880000	1.61060000
C	33.98240000	-16.30510000	2.39320000
H	33.72090000	-17.32740000	2.12300000
H	34.54200000	-16.34350000	3.32800000
H	33.05540000	-15.76440000	2.58270000

O	29.69840000	-9.02620000	-20.37300000
H	29.41870000	-9.41350000	-21.20510000
O	29.60040000	-11.28060000	-21.64800000
H	30.15260000	-11.77660000	-22.24550000
O	32.15850000	-12.28430000	-21.56590000
H	31.80050000	-13.10180000	-21.20800000

5-alpha dimer

C	31.65770000	-5.00940000	-15.73940000
C	28.91090000	-6.31960000	-15.87020000
O	31.53720000	-4.13550000	-14.88430000
O	29.22690000	-7.44950000	-16.24390000
N	32.48150000	-6.04940000	-15.57480000
N	28.21170000	-6.10340000	-14.75030000
H	32.52390000	-6.77290000	-16.29080000
H	27.99080000	-5.13450000	-14.51380000
C	30.80440000	-4.92990000	-17.01440000
H	30.95060000	-3.90890000	-17.37120000
C	29.28180000	-5.07360000	-16.70060000
H	28.99260000	-4.22650000	-16.08610000
C	31.28780000	-5.86140000	-18.15400000
H	32.33860000	-5.67100000	-18.37820000
H	31.22080000	-6.89880000	-17.84610000
C	28.42490000	-4.98500000	-17.98370000
H	27.39610000	-5.27830000	-17.76380000
C	30.44720000	-5.64140000	-19.41430000
H	30.61430000	-4.61750000	-19.75640000
C	28.95080000	-5.86430000	-19.13560000
H	28.75860000	-6.91600000	-18.91460000
C	27.63230000	-7.12730000	-13.89540000
H	26.65530000	-6.76360000	-13.57600000
H	27.44570000	-8.04430000	-14.45700000
C	33.25590000	-6.31850000	-14.37540000
H	34.10560000	-6.94230000	-14.65510000
H	33.66370000	-5.39170000	-13.96710000
C	28.51190000	-7.41440000	-12.66950000
H	29.44860000	-7.87470000	-12.98720000
H	28.77680000	-6.47680000	-12.17810000
C	27.78910000	-8.32870000	-11.67120000
H	26.84290000	-7.86470000	-11.39230000
H	27.52820000	-9.26750000	-12.15980000
C	28.60040000	-8.61720000	-10.40030000
H	28.93010000	-7.67770000	-9.95550000
H	29.50260000	-9.17240000	-10.65780000
C	27.77960000	-9.40740000	-9.37130000
H	26.87770000	-8.84550000	-9.12780000
H	27.44040000	-10.34270000	-9.81750000
C	28.55230000	-9.70540000	-8.07870000
H	28.90870000	-8.77120000	-7.64490000
H	29.43820000	-10.29670000	-8.30970000
C	27.69280000	-10.45300000	-7.04930000
H	26.80130000	-9.86480000	-6.83060000
H	27.34040000	-11.38930000	-7.48330000
C	28.44210000	-10.74440000	-5.74090000
H	28.79320000	-9.80800000	-5.30740000
H	29.33120000	-11.33790000	-5.95300000
C	27.56870000	-11.48630000	-4.71950000
H	26.67080000	-10.90080000	-4.52000000
H	27.22940000	-12.42900000	-5.15050000
C	28.30040000	-11.76180000	-3.39760000
H	28.63500000	-10.81890000	-2.96460000
H	29.19900000	-12.34690000	-3.59240000
C	27.42000000	-12.50700000	-2.38500000
H	26.51170000	-11.93150000	-2.20400000
H	27.10050000	-13.45750000	-2.81410000

C	28.13470000	-12.76250000	-1.05020000
H	28.45050000	-11.81200000	-0.61960000
H	29.04370000	-13.33770000	-1.22640000
C	27.24810000	-13.51030000	-0.04530000
H	26.32950000	-12.94530000	0.11620000
H	26.94850000	-14.46930000	-0.46990000
C	27.94540000	-13.74320000	1.30240000
H	28.24680000	-12.78460000	1.72550000
H	28.86210000	-14.31210000	1.14630000
C	27.05000000	-14.48520000	2.30370000
H	26.12520000	-13.92660000	2.45060000
H	26.76180000	-15.45310000	1.89140000
C	27.73090000	-14.69580000	3.66030000
H	28.01570000	-13.74540000	4.11110000
H	27.06320000	-15.20480000	4.35570000
H	28.63130000	-15.30160000	3.56170000
C	32.39010000	-7.04180000	-13.33570000
H	31.96870000	-7.94610000	-13.77790000
H	31.54540000	-6.40580000	-13.06580000
C	33.17580000	-7.40480000	-12.07330000
H	33.99840000	-8.07220000	-12.33360000
H	33.62630000	-6.50290000	-11.65660000
C	32.28650000	-8.06750000	-11.01720000
H	31.84790000	-8.97700000	-11.42860000
H	31.45450000	-7.40340000	-10.77990000
C	33.05760000	-8.39770000	-9.73510000
H	33.87970000	-9.07640000	-9.96560000
H	33.50950000	-7.48680000	-9.34020000
C	32.15800000	-9.02490000	-8.66610000
H	31.72650000	-9.94790000	-9.05240000
H	31.32140000	-8.35650000	-8.46050000
C	32.91340000	-9.31270000	-7.36350000
H	33.74800000	-9.98520000	-7.56500000
H	33.34780000	-8.38730000	-6.98330000
C	32.00670000	-9.92780000	-6.29250000
H	31.59440000	-10.86510000	-6.66460000
H	31.15720000	-9.26820000	-6.11410000
C	32.74380000	-10.17840000	-4.97130000
H	33.59670000	-10.83470000	-5.14710000
H	33.15070000	-9.23840000	-4.59690000
C	31.83050000	-10.79920000	-3.90840000
H	31.44320000	-11.74840000	-4.27730000
H	30.96510000	-10.15510000	-3.75190000
C	32.54790000	-11.02240000	-2.57140000
H	33.42040000	-11.65740000	-2.72680000
H	32.92390000	-10.07020000	-2.19560000
C	31.63080000	-11.66080000	-1.52180000
H	31.27100000	-12.61950000	-1.89430000
H	30.74860000	-11.03610000	-1.38110000
C	32.33060000	-11.86550000	-0.17240000
H	33.22060000	-12.47910000	-0.31310000
H	32.67790000	-10.90450000	0.20820000
C	31.41290000	-12.52540000	0.86380000
H	31.07640000	-13.48980000	0.48410000
H	30.51690000	-11.91860000	0.99540000
C	32.10060000	-12.72110000	2.22080000
H	33.00440000	-13.31640000	2.09200000
H	32.42430000	-11.75650000	2.61250000
C	31.18700000	-13.40230000	3.24410000
H	30.87710000	-14.38790000	2.89930000
H	31.69530000	-13.52830000	4.20010000
H	30.28660000	-12.81500000	3.42100000
O	28.37160000	-3.65750000	-18.47530000
H	28.09940000	-3.78530000	-19.38560000
O	28.28430000	-5.49460000	-20.32580000
H	28.84430000	-5.81960000	-21.02470000
O	30.83660000	-6.51200000	-20.46040000
H	30.48000000	-7.39010000	-20.30000000

C	33.05780000	-9.57780000	-17.43180000
C	30.30480000	-10.81810000	-17.11620000
O	32.93180000	-8.50750000	-16.84360000
O	30.44670000	-12.03610000	-17.22510000
N	33.91030000	-10.52050000	-17.01740000
N	29.80370000	-10.24910000	-16.01100000
H	33.95750000	-11.40300000	-17.52320000
H	29.70930000	-9.23220000	-16.01680000
C	32.17790000	-9.85720000	-18.65820000
H	32.32640000	-8.98350000	-19.29520000
C	30.66180000	-9.87940000	-18.28890000
H	30.39650000	-8.88270000	-17.95060000
C	32.62500000	-11.08380000	-19.49090000
H	33.67790000	-10.99460000	-19.76380000
H	32.52790000	-11.99310000	-18.90560000
C	29.77640000	-10.16040000	-19.52780000
H	28.75550000	-10.38420000	-19.21080000
C	31.77510000	-11.18640000	-20.75880000
H	31.93850000	-10.28120000	-21.34800000
C	30.28300000	-11.32170000	-20.41060000
H	30.09390000	-12.27710000	-19.91740000
C	29.21040000	-10.95810000	-14.88290000
H	28.38170000	-10.35160000	-14.51680000
H	28.77060000	-11.89890000	-15.21940000
C	34.73080000	-10.44600000	-15.81960000
H	35.59360000	-11.09490000	-15.97450000
H	35.11750000	-9.43550000	-15.67340000
C	30.20660000	-11.21420000	-13.73800000
H	31.03100000	-11.82950000	-14.10010000
H	30.64210000	-10.26930000	-13.40980000
C	29.52040000	-11.91110000	-12.54930000
H	28.68210000	-11.30120000	-12.21350000
H	29.08860000	-12.85280000	-12.88890000
C	30.44350000	-12.18440000	-11.35110000
H	30.88210000	-11.24880000	-11.00360000
H	31.27230000	-12.81760000	-11.66610000
C	29.69000000	-12.86100000	-10.19340000
H	28.85480000	-12.23050000	-9.88950000
H	29.24900000	-13.79400000	-10.54480000
C	30.56940000	-13.15070000	-8.96790000
H	30.99870000	-12.22020000	-8.59760000
H	31.40730000	-13.78180000	-9.26170000
C	29.78370000	-13.83890000	-7.84090000
H	28.92790000	-13.22190000	-7.56720000
H	29.37320000	-14.77990000	-8.20850000
C	30.63190000	-14.10960000	-6.58990000
H	31.02780000	-13.16920000	-6.20760000
H	31.49400000	-14.71940000	-6.85850000
C	29.83370000	-14.81660000	-5.48510000
H	28.95030000	-14.22590000	-5.24160000
H	29.46760000	-15.77330000	-5.85950000
C	30.65590000	-15.04840000	-4.20920000
H	31.00830000	-14.09150000	-3.82510000
H	31.54610000	-15.62950000	-4.44900000
C	29.85360000	-15.77320000	-3.12000000
H	28.94330000	-15.21200000	-2.90630000
H	29.53290000	-16.74620000	-3.49450000
C	30.65030000	-15.96200000	-1.82130000
H	30.95960000	-14.98910000	-1.43990000
H	31.56620000	-16.51430000	-2.03110000
C	29.84550000	-16.70150000	-0.74420000
H	28.91250000	-16.16810000	-0.55860000
H	29.56650000	-17.68830000	-1.11590000
C	30.61690000	-16.85050000	0.57460000
H	30.88480000	-15.86390000	0.95230000
H	31.55590000	-17.37320000	0.39170000
C	29.81150000	-17.60600000	1.64000000
H	28.85890000	-17.10180000	1.80630000

H	29.57030000	-18.60480000	1.27390000
C	30.55950000	-17.72400000	2.97270000
H	30.76860000	-16.74350000	3.39940000
H	29.97070000	-18.28260000	3.70070000
H	31.51010000	-18.24190000	2.84850000
C	33.93290000	-10.91140000	-14.59340000
H	33.42460000	-11.84620000	-14.83060000
H	33.14830000	-10.18580000	-14.37330000
C	34.81660000	-11.11300000	-13.35680000
H	35.62320000	-11.80650000	-13.59900000
H	35.29010000	-10.16810000	-13.08670000
C	34.02360000	-11.65790000	-12.16350000
H	33.52770000	-12.58470000	-12.45370000
H	33.23300000	-10.95350000	-11.90310000
C	34.90660000	-11.91600000	-10.93580000
H	35.70450000	-12.61270000	-11.19700000
H	35.39300000	-10.98690000	-10.63580000
C	34.10410000	-12.48010000	-9.75770000
H	33.64160000	-13.42160000	-10.05500000
H	33.28780000	-11.79790000	-9.51880000
C	34.96240000	-12.70210000	-8.50510000
H	35.78600000	-13.37810000	-8.73810000
H	35.41510000	-11.75670000	-8.20340000
C	34.14490000	-13.27430000	-7.34030000
H	33.72280000	-14.23490000	-7.63540000
H	33.29930000	-12.61720000	-7.13580000
C	34.97170000	-13.44870000	-6.05910000
H	35.82810000	-14.09350000	-6.25900000
H	35.37790000	-12.48310000	-5.75520000
C	34.14250000	-14.04120000	-4.91210000
H	33.76430000	-15.01900000	-5.20990000
H	33.26750000	-13.41480000	-4.73680000
C	34.93930000	-14.17660000	-3.60740000
H	35.82610000	-14.78680000	-3.78100000
H	35.29830000	-13.19420000	-3.29810000
C	34.10540000	-14.79820000	-2.47890000
H	33.77170000	-15.79020000	-2.78230000
H	33.20330000	-14.20560000	-2.32490000
C	34.87810000	-14.90150000	-1.15680000
H	35.79120000	-15.47710000	-1.31100000
H	35.19290000	-13.90570000	-0.84220000
C	34.04730000	-15.55340000	-0.04320000
H	33.75260000	-16.55600000	-0.35210000
H	33.12270000	-14.99290000	0.09730000
C	34.80480000	-15.63220000	1.28920000
H	35.73630000	-16.18150000	1.15160000
H	35.08680000	-14.62880000	1.61060000
C	33.98240000	-16.30510000	2.39320000
H	33.72090000	-17.32740000	2.12300000
H	34.54200000	-16.34350000	3.32800000
H	33.05540000	-15.76440000	2.58270000
O	29.69840000	-9.02620000	-20.37300000
H	29.41870000	-9.41350000	-21.20510000
O	29.60040000	-11.28060000	-21.64800000
H	30.15260000	-11.77660000	-22.24550000
O	32.15850000	-12.28430000	-21.56590000
H	31.80050000	-13.10180000	-21.20800000

5-alpha tetramer

C	31.65770000	-5.00940000	-15.73940000
C	28.91090000	-6.31960000	-15.87020000
O	31.53720000	-4.13550000	-14.88430000
O	29.22690000	-7.44950000	-16.24390000
N	32.48150000	-6.04940000	-15.57480000
N	28.21170000	-6.10340000	-14.75030000
H	32.52390000	-6.77290000	-16.29080000

H	27.99080000	-5.13450000	-14.51380000
C	30.80440000	-4.92990000	-17.01440000
H	30.95060000	-3.90890000	-17.37120000
C	29.28180000	-5.07360000	-16.70060000
H	28.99260000	-4.22650000	-16.08610000
C	31.28780000	-5.86140000	-18.15400000
H	32.33860000	-5.67100000	-18.37820000
H	31.22080000	-6.89880000	-17.84610000
C	28.42490000	-4.98500000	-17.98370000
H	27.39610000	-5.27830000	-17.76380000
C	30.44720000	-5.64140000	-19.41430000
H	30.61430000	-4.61750000	-19.75640000
C	28.95080000	-5.86430000	-19.13560000
H	28.75860000	-6.91600000	-18.91460000
C	27.63230000	-7.12730000	-13.89540000
H	26.65530000	-6.76360000	-13.57600000
H	27.44570000	-8.04430000	-14.45700000
C	33.25590000	-6.31850000	-14.37540000
H	34.10560000	-6.94230000	-14.65510000
H	33.66370000	-5.39170000	-13.96710000
C	28.51190000	-7.41440000	-12.66950000
H	29.44860000	-7.87470000	-12.98720000
H	28.77680000	-6.47680000	-12.17810000
C	27.78910000	-8.32870000	-11.67120000
H	26.84290000	-7.86470000	-11.39230000
H	27.52820000	-9.26750000	-12.15980000
C	28.60040000	-8.61720000	-10.40030000
H	28.93010000	-7.67770000	-9.95550000
H	29.50260000	-9.17240000	-10.65780000
C	27.77960000	-9.40740000	-9.37130000
H	26.87770000	-8.84550000	-9.12780000
H	27.44040000	-10.34270000	-9.81750000
C	28.55230000	-9.70540000	-8.07870000
H	28.90870000	-8.77120000	-7.64490000
H	29.43820000	-10.29670000	-8.30970000
C	27.69280000	-10.45300000	-7.04930000
H	26.80130000	-9.86480000	-6.83060000
H	27.34040000	-11.38930000	-7.48330000
C	28.44210000	-10.74440000	-5.74090000
H	28.79320000	-9.80800000	-5.30740000
H	29.33120000	-11.33790000	-5.95300000
C	27.56870000	-11.48630000	-4.71950000
H	26.67080000	-10.90080000	-4.52000000
H	27.22940000	-12.42900000	-5.15050000
C	28.30040000	-11.76180000	-3.39760000
H	28.63500000	-10.81890000	-2.96460000
H	29.19900000	-12.34690000	-3.59240000
C	27.42000000	-12.50700000	-2.38500000
H	26.51170000	-11.93150000	-2.20400000
H	27.10050000	-13.45750000	-2.81410000
C	28.13470000	-12.76250000	-1.05020000
H	28.45050000	-11.81200000	-0.61960000
H	29.04370000	-13.33770000	-1.22640000
C	27.24810000	-13.51030000	-0.04530000
H	26.32950000	-12.94530000	0.11620000
H	26.94850000	-14.46930000	-0.46990000
C	27.94540000	-13.74320000	1.30240000
H	28.24680000	-12.78460000	1.72550000
H	28.86210000	-14.31210000	1.14630000
C	27.05000000	-14.48520000	2.30370000
H	26.12520000	-13.92660000	2.45060000
H	26.76180000	-15.45310000	1.89140000
C	27.73090000	-14.69580000	3.66030000
H	28.01570000	-13.74540000	4.11110000
H	27.06320000	-15.20480000	4.35570000
H	28.63130000	-15.30160000	3.56170000
C	32.39010000	-7.04180000	-13.33570000
H	31.96870000	-7.94610000	-13.77790000

H	31.54540000	-6.40580000	-13.06580000
C	33.17580000	-7.40480000	-12.07330000
H	33.99840000	-8.07220000	-12.33360000
H	33.62630000	-6.50290000	-11.65660000
C	32.28650000	-8.06750000	-11.01720000
H	31.84790000	-8.97700000	-11.42860000
H	31.45450000	-7.40340000	-10.77990000
C	33.05760000	-8.39770000	-9.73510000
H	33.87970000	-9.07640000	-9.96560000
H	33.50950000	-7.48680000	-9.34020000
C	32.15800000	-9.02490000	-8.66610000
H	31.72650000	-9.94790000	-9.05240000
H	31.32140000	-8.35650000	-8.46050000
C	32.91340000	-9.31270000	-7.36350000
H	33.74800000	-9.98520000	-7.56500000
H	33.34780000	-8.38730000	-6.98330000
C	32.00670000	-9.92780000	-6.29250000
H	31.59440000	-10.86510000	-6.66460000
H	31.15720000	-9.26820000	-6.11410000
C	32.74380000	-10.17840000	-4.97130000
H	33.59670000	-10.83470000	-5.14710000
H	33.15070000	-9.23840000	-4.59690000
C	31.83050000	-10.79920000	-3.90840000
H	31.44320000	-11.74840000	-4.27730000
H	30.96510000	-10.15510000	-3.75190000
C	32.54790000	-11.02240000	-2.57140000
H	33.42040000	-11.65740000	-2.72680000
H	32.92390000	-10.07020000	-2.19560000
C	31.63080000	-11.66080000	-1.52180000
H	31.27100000	-12.61950000	-1.89430000
H	30.74860000	-11.03610000	-1.38110000
C	32.33060000	-11.86550000	-0.17240000
H	33.22060000	-12.47910000	-0.31310000
H	32.67790000	-10.90450000	0.20820000
C	31.41290000	-12.52540000	0.86380000
H	31.07640000	-13.48980000	0.48410000
H	30.51690000	-11.91860000	0.99540000
C	32.10060000	-12.72110000	2.22080000
H	33.00440000	-13.31640000	2.09200000
H	32.42430000	-11.75650000	2.61250000
C	31.18700000	-13.40230000	3.24410000
H	30.87710000	-14.38790000	2.89930000
H	31.69530000	-13.52830000	4.20010000
H	30.28660000	-12.81500000	3.42100000
O	28.37160000	-3.65750000	-18.47530000
H	28.09940000	-3.78530000	-19.38560000
O	28.28430000	-5.49460000	-20.32580000
H	28.84430000	-5.81960000	-21.02470000
O	30.83660000	-6.51200000	-20.46040000
H	30.48000000	-7.39010000	-20.30000000
C	30.15740000	-1.29540000	-12.65160000
C	27.47410000	-2.54680000	-13.24880000
O	29.94300000	-0.76490000	-11.56370000
O	27.44960000	-3.44760000	-14.08870000
N	31.03710000	-2.29700000	-12.79410000
N	27.18490000	-2.75640000	-11.95740000
H	31.14260000	-2.74460000	-13.70360000
H	27.31260000	-1.96970000	-11.33440000
C	29.33460000	-0.83820000	-13.86750000
H	29.46680000	0.24470000	-13.89180000
C	27.81120000	-1.09230000	-13.65080000
H	27.49690000	-0.46770000	-12.81230000
C	29.85140000	-1.37420000	-15.22290000
H	30.90880000	-1.13720000	-15.35040000
H	29.76420000	-2.45270000	-15.24070000
C	26.97480000	-0.63210000	-14.87390000
H	25.95200000	-1.00330000	-14.78390000
C	29.04410000	-0.77500000	-16.37740000

H	29.19750000	0.30640000	-16.37100000
C	27.54660000	-1.09310000	-16.23190000
H	27.37320000	-2.16220000	-16.36520000
C	26.62830000	-3.97490000	-11.38000000
H	25.56570000	-3.79610000	-11.21050000
H	26.69460000	-4.81650000	-12.07070000
C	31.83900000	-2.85750000	-11.71470000
H	32.66660000	-3.41540000	-12.15420000
H	32.27910000	-2.04650000	-11.13120000
C	27.32230000	-4.33010000	-10.05510000
H	28.30960000	-4.74040000	-10.26620000
H	27.48190000	-3.42540000	-9.46630000
C	26.51890000	-5.33220000	-9.21120000
H	25.53960000	-4.90320000	-8.99420000
H	26.33460000	-6.24070000	-9.78390000
C	27.21590000	-5.69110000	-7.88930000
H	27.51900000	-4.77590000	-7.37850000
H	28.13200000	-6.24390000	-8.09850000
C	26.32010000	-6.51250000	-6.94970000
H	25.40780000	-5.95120000	-6.74260000
H	26.00770000	-7.43160000	-7.44560000
C	27.01330000	-6.85570000	-5.62300000
H	27.36140000	-5.93720000	-5.14870000
H	27.90180000	-7.45560000	-5.82060000
C	26.09210000	-7.60880000	-4.65200000
H	25.20020000	-7.01000000	-4.46250000
H	25.74770000	-8.53340000	-5.11610000
C	26.77980000	-7.93060000	-3.31770000
H	27.12840000	-7.00470000	-2.85850000
H	27.66750000	-8.53570000	-3.50220000
C	25.85430000	-8.66750000	-2.33910000
H	24.96080000	-8.06690000	-2.16330000
H	25.51370000	-9.59990000	-2.79060000
C	26.53680000	-8.96770000	-0.99740000
H	26.87270000	-8.03330000	-0.54610000
H	27.43200000	-9.56540000	-1.17010000
C	25.61310000	-9.70460000	-0.01750000
H	24.71090000	-9.11320000	0.14500000
H	25.28790000	-10.64610000	-0.46170000
C	26.28960000	-9.98210000	1.33210000
H	26.60910000	-9.03900000	1.77720000
H	27.19420000	-10.56880000	1.17110000
C	25.36880000	-10.72290000	2.31170000
H	24.45930000	-10.14080000	2.46590000
H	25.05670000	-11.67030000	1.87070000
C	26.04120000	-10.98570000	3.66630000
H	26.34440000	-10.03670000	4.11040000
H	26.95560000	-11.55890000	3.51220000
C	25.12490000	-11.73750000	4.64190000
H	24.20820000	-11.16690000	4.79680000
H	24.82350000	-12.68980000	4.20370000
C	25.79220000	-11.99590000	5.99740000
H	26.07370000	-11.06150000	6.48390000
H	25.11690000	-12.52790000	6.66790000
H	26.69210000	-12.60100000	5.88720000
C	31.00000000	-3.77890000	-10.81440000
H	30.61250000	-4.61550000	-11.39750000
H	30.13360000	-3.22250000	-10.45570000
C	31.77990000	-4.30080000	-9.60050000
H	32.59160000	-4.95110000	-9.92850000
H	32.24600000	-3.45610000	-9.09100000
C	30.87440000	-5.04430000	-8.60880000
H	30.46820000	-5.93970000	-9.07940000
H	30.02110000	-4.41040000	-8.36380000
C	31.59720000	-5.41890000	-7.30880000
H	32.42740000	-6.09110000	-7.52740000
H	32.03490000	-4.52120000	-6.86960000
C	30.65340000	-6.06940000	-6.28880000

H	30.25090000	-6.99390000	-6.70240000
H	29.80010000	-5.41220000	-6.11620000
C	31.34100000	-6.35890000	-4.94880000
H	32.19420000	-7.01810000	-5.11050000
H	31.74250000	-5.43040000	-4.54050000
C	30.38520000	-6.99180000	-3.92900000
H	30.00180000	-7.93170000	-4.32600000
H	29.52100000	-6.34130000	-3.78880000
C	31.05520000	-7.24200000	-2.57210000
H	31.92830000	-7.88040000	-2.70980000
H	31.42510000	-6.29740000	-2.17100000
C	30.10140000	-7.88860000	-1.55890000
H	29.74210000	-8.83870000	-1.95490000
H	29.22260000	-7.25560000	-1.43130000
C	30.76380000	-8.11890000	-0.19440000
H	31.65330000	-8.73630000	-0.32270000
H	31.10730000	-7.16480000	0.20760000
C	29.81770000	-8.78710000	0.81220000
H	29.47890000	-9.74280000	0.41150000
H	28.92610000	-8.17170000	0.93720000
C	30.47810000	-9.00940000	2.17940000
H	31.38000000	-9.60880000	2.05280000
H	30.80150000	-8.05040000	2.58650000
C	29.54100000	-9.69980000	3.17970000
H	29.21570000	-10.65670000	2.77060000
H	28.64070000	-9.09780000	3.30830000
C	30.20280000	-9.92510000	4.54620000
H	31.10770000	-10.52130000	4.42300000
H	30.52040000	-8.96810000	4.96200000
C	29.27050000	-10.62360000	5.54200000
H	28.95920000	-11.60110000	5.17380000
H	29.76700000	-10.77400000	6.50090000
H	28.37250000	-10.03250000	5.72310000
O	26.88870000	0.77810000	-14.93700000
H	26.64320000	0.94310000	-15.84530000
O	26.90550000	-0.38150000	-17.27260000
H	27.50210000	-0.45070000	-18.01150000
O	29.48270000	-1.26230000	-17.63330000
H	29.11910000	-2.13800000	-17.78860000
C	34.04650000	-14.44600000	-17.59710000
C	31.22990000	-15.55670000	-17.30250000
O	34.34240000	-13.25460000	-17.61730000
O	31.39670000	-16.75110000	-17.06260000
N	34.30930000	-15.23210000	-16.54630000
N	30.64580000	-14.72260000	-16.43380000
H	33.98650000	-16.18920000	-16.58500000
H	30.56290000	-13.73790000	-16.69480000
C	33.29480000	-15.05640000	-18.78790000
H	33.55120000	-14.41450000	-19.63300000
C	31.74560000	-14.95560000	-18.62290000
H	31.48060000	-13.90210000	-18.59890000
C	33.79240000	-16.47590000	-19.15690000
H	34.87320000	-16.46230000	-19.30460000
H	33.59820000	-17.18500000	-18.35140000
C	31.00640000	-15.57530000	-19.83130000
H	29.94470000	-15.68700000	-19.60020000
C	33.09260000	-16.94660000	-20.43640000
H	33.36830000	-16.27760000	-21.25500000
C	31.56330000	-16.94980000	-20.25790000
H	31.27420000	-17.71360000	-19.53250000
C	30.09740000	-15.12150000	-15.14610000
H	29.36110000	-14.37870000	-14.83940000
H	29.55760000	-16.06410000	-15.25870000
C	34.80290000	-14.73210000	-15.27210000
H	35.69740000	-14.12500000	-15.42490000
H	34.04210000	-14.07540000	-14.84900000
C	31.19340000	-15.25560000	-14.07600000
H	31.97620000	-15.92080000	-14.44020000

H	31.66270000	-14.28710000	-13.89900000
C	30.63770000	-15.82050000	-12.76430000
H	29.85110000	-15.16590000	-12.39000000
H	30.16200000	-16.78080000	-12.96940000
C	31.70760000	-16.01470000	-11.68100000
H	32.15380000	-15.05420000	-11.42180000
H	32.51420000	-16.63690000	-12.07010000
C	31.11650000	-16.67310000	-10.42840000
H	30.28610000	-16.06940000	-10.06160000
H	30.69170000	-17.64060000	-10.70000000
C	32.13630000	-16.87060000	-9.30000000
H	32.54660000	-15.90580000	-9.00140000
H	32.97500000	-17.46580000	-9.66260000
C	31.50060000	-17.56280000	-8.08740000
H	30.63300000	-16.99000000	-7.75830000
H	31.12330000	-18.54110000	-8.38870000
C	32.46810000	-17.73290000	-6.91020000
H	32.83570000	-16.75610000	-6.59580000
H	33.34000000	-18.30340000	-7.23160000
C	31.79940000	-18.43950000	-5.72380000
H	30.90200000	-17.89160000	-5.43420000
H	31.46570000	-19.43050000	-6.03470000
C	32.72480000	-18.57110000	-4.50830000
H	33.04860000	-17.58030000	-4.19010000
H	33.62650000	-19.11460000	-4.79230000
C	32.03990000	-19.28940000	-3.33820000
H	31.11620000	-18.76880000	-3.08240000
H	31.74960000	-20.29380000	-3.64950000
C	32.93370000	-19.37710000	-2.09530000
H	33.21560000	-18.37200000	-1.78170000
H	33.86080000	-19.89240000	-2.34800000
C	32.24460000	-20.10340000	-0.93270000
H	31.29900000	-19.61000000	-0.70450000
H	31.99450000	-21.12060000	-1.23730000
C	33.11440000	-20.14560000	0.33000000
H	33.36030000	-19.12750000	0.63150000
H	34.06150000	-20.63670000	0.10390000
C	32.42510000	-20.87290000	1.49210000
H	31.46310000	-20.40370000	1.70080000
H	32.20650000	-21.90160000	1.20240000
C	33.27420000	-20.87630000	2.76780000
H	33.48840000	-19.86180000	3.10490000
H	32.75790000	-21.39240000	3.57760000
H	34.22530000	-21.38320000	2.60500000
C	35.09520000	-15.88210000	-14.29940000
H	35.95410000	-16.45320000	-14.65490000
H	34.25180000	-16.57350000	-14.27700000
C	35.35760000	-15.37400000	-12.87520000
H	36.18120000	-14.65860000	-12.88250000
H	34.48040000	-14.82700000	-12.52780000
C	35.66930000	-16.50710000	-11.88820000
H	36.60930000	-16.98550000	-12.16690000
H	34.89970000	-17.27710000	-11.95780000
C	35.74830000	-16.00750000	-10.43990000
H	36.49310000	-15.21410000	-10.36440000
H	34.79320000	-15.55940000	-10.16430000
C	36.09020000	-17.12620000	-9.44680000
H	37.08250000	-17.52040000	-9.67020000
H	35.39350000	-17.95580000	-9.57460000
C	36.03960000	-16.64400000	-7.99160000
H	36.71370000	-15.79600000	-7.86420000
H	35.03720000	-16.27600000	-7.77130000
C	36.41000000	-17.74530000	-6.98920000
H	37.43660000	-18.06860000	-7.16730000
H	35.77880000	-18.61940000	-7.15440000
C	36.26020000	-17.27830000	-5.53570000
H	36.87210000	-16.38990000	-5.37440000
H	35.22730000	-16.97630000	-5.36030000

C	36.65500000	-18.35970000	-4.52100000
H	37.70340000	-18.62550000	-4.66300000
H	36.07900000	-19.26670000	-4.70870000
C	36.43030000	-17.90410000	-3.07320000
H	36.98900000	-16.98520000	-2.89140000
H	35.37770000	-17.65720000	-2.93260000
C	36.84790000	-18.96470000	-2.04560000
H	37.91070000	-19.18230000	-2.16020000
H	36.31930000	-19.89740000	-2.24620000
C	36.56670000	-18.51860000	-0.60460000
H	37.07800000	-17.57490000	-0.41100000
H	35.50060000	-18.32060000	-0.48990000
C	37.00870000	-19.55810000	0.43420000
H	38.08210000	-19.72910000	0.34140000
H	36.52590000	-20.51330000	0.22360000
C	36.67990000	-19.12590000	1.86990000
H	37.14690000	-18.16320000	2.08140000
H	35.60480000	-18.97310000	1.96760000
C	37.14170000	-20.15020000	2.91200000
H	38.22350000	-20.28390000	2.88130000
H	36.87570000	-19.82730000	3.91890000
H	36.68020000	-21.12330000	2.74290000
O	31.08800000	-14.73850000	-20.97000000
H	30.88550000	-15.34830000	-21.68250000
O	31.03180000	-17.28040000	-21.52490000
H	31.53350000	-18.04530000	-21.78930000
O	33.49000000	-18.26040000	-20.78370000
H	34.41530000	-18.25690000	-20.98280000
C	33.05780000	-9.57780000	-17.43180000
C	30.30480000	-10.81810000	-17.11620000
O	32.93180000	-8.50750000	-16.84360000
O	30.44670000	-12.03610000	-17.22510000
N	33.91030000	-10.52050000	-17.01740000
N	29.80370000	-10.24910000	-16.01100000
H	33.95750000	-11.40300000	-17.52320000
H	29.70930000	-9.23220000	-16.01680000
C	32.17790000	-9.85720000	-18.65820000
H	32.32640000	-8.98350000	-19.29520000
C	30.66180000	-9.87940000	-18.28890000
H	30.39650000	-8.88270000	-17.95060000
C	32.62500000	-11.08380000	-19.49090000
H	33.67790000	-10.99460000	-19.76380000
H	32.52790000	-11.99310000	-18.90560000
C	29.77640000	-10.16040000	-19.52780000
H	28.75550000	-10.38420000	-19.21080000
C	31.77510000	-11.18640000	-20.75880000
H	31.93850000	-10.28120000	-21.34800000
C	30.28300000	-11.32170000	-20.41060000
H	30.09390000	-12.27710000	-19.91740000
C	29.21040000	-10.95810000	-14.88290000
H	28.38170000	-10.35160000	-14.51680000
H	28.77060000	-11.89890000	-15.21940000
C	34.73080000	-10.44600000	-15.81960000
H	35.59360000	-11.09490000	-15.97450000
H	35.11750000	-9.43550000	-15.67340000
C	30.20660000	-11.21420000	-13.73800000
H	31.03100000	-11.82950000	-14.10010000
H	30.64210000	-10.26930000	-13.40980000
C	29.52040000	-11.91110000	-12.54930000
H	28.68210000	-11.30120000	-12.21350000
H	29.08860000	-12.85280000	-12.88890000
C	30.44350000	-12.18440000	-11.35110000
H	30.88210000	-11.24880000	-11.00360000
H	31.27230000	-12.81760000	-11.66610000
C	29.69000000	-12.86100000	-10.19340000
H	28.85480000	-12.23050000	-9.88950000
H	29.24900000	-13.79400000	-10.54480000
C	30.56940000	-13.15070000	-8.96790000

H	30.99870000	-12.22020000	-8.59760000
H	31.40730000	-13.78180000	-9.26170000
C	29.78370000	-13.83890000	-7.84090000
H	28.92790000	-13.22190000	-7.56720000
H	29.37320000	-14.77990000	-8.20850000
C	30.63190000	-14.10960000	-6.58990000
H	31.02780000	-13.16920000	-6.20760000
H	31.49400000	-14.71940000	-6.85850000
C	29.83370000	-14.81660000	-5.48510000
H	28.95030000	-14.22590000	-5.24160000
H	29.46760000	-15.77330000	-5.85950000
C	30.65590000	-15.04840000	-4.20920000
H	31.00830000	-14.09150000	-3.82510000
H	31.54610000	-15.62950000	-4.44900000
C	29.85360000	-15.77320000	-3.12000000
H	28.94330000	-15.21200000	-2.90630000
H	29.53290000	-16.74620000	-3.49450000
C	30.65030000	-15.96200000	-1.82130000
H	30.95960000	-14.98910000	-1.43990000
H	31.56620000	-16.51430000	-2.03110000
C	29.84550000	-16.70150000	-0.74420000
H	28.91250000	-16.16810000	-0.55860000
H	29.56650000	-17.68830000	-1.11590000
C	30.61690000	-16.85050000	0.57460000
H	30.88480000	-15.86390000	0.95230000
H	31.55590000	-17.37320000	0.39170000
C	29.81150000	-17.60600000	1.64000000
H	28.85890000	-17.10180000	1.80630000
H	29.57030000	-18.60480000	1.27390000
C	30.55950000	-17.72400000	2.97270000
H	30.76860000	-16.74350000	3.39940000
H	29.97070000	-18.28260000	3.70070000
H	31.51010000	-18.24190000	2.84850000
C	33.93290000	-10.91140000	-14.59340000
H	33.42460000	-11.84620000	-14.83060000
H	33.14830000	-10.18580000	-14.37330000
C	34.81660000	-11.11300000	-13.35680000
H	35.62320000	-11.80650000	-13.59900000
H	35.29010000	-10.16810000	-13.08670000
C	34.02360000	-11.65790000	-12.16350000
H	33.52770000	-12.58470000	-12.45370000
H	33.23300000	-10.95350000	-11.90310000
C	34.90660000	-11.91600000	-10.93580000
H	35.70450000	-12.61270000	-11.19700000
H	35.39300000	-10.98690000	-10.63580000
C	34.10410000	-12.48010000	-9.75770000
H	33.64160000	-13.42160000	-10.05500000
H	33.28780000	-11.79790000	-9.51880000
C	34.96240000	-12.70210000	-8.50510000
H	35.78600000	-13.37810000	-8.73810000
H	35.41510000	-11.75670000	-8.20340000
C	34.14490000	-13.27430000	-7.34030000
H	33.72280000	-14.23490000	-7.63540000
H	33.29930000	-12.61720000	-7.13580000
C	34.97170000	-13.44870000	-6.05910000
H	35.82810000	-14.09350000	-6.25900000
H	35.37790000	-12.48310000	-5.75520000
C	34.14250000	-14.04120000	-4.91210000
H	33.76430000	-15.01900000	-5.20990000
H	33.26750000	-13.41480000	-4.73680000
C	34.93930000	-14.17660000	-3.60740000
H	35.82610000	-14.78680000	-3.78100000
H	35.29830000	-13.19420000	-3.29810000
C	34.10540000	-14.79820000	-2.47890000
H	33.77170000	-15.79020000	-2.78230000
H	33.20330000	-14.20560000	-2.32490000
C	34.87810000	-14.90150000	-1.15680000
H	35.79120000	-15.47710000	-1.31100000

H	35.19290000	-13.90570000	-0.84220000
C	34.04730000	-15.55340000	-0.04320000
H	33.75260000	-16.55600000	-0.35210000
H	33.12270000	-14.99290000	0.09730000
C	34.80480000	-15.63220000	1.28920000
H	35.73630000	-16.18150000	1.15160000
H	35.08680000	-14.62880000	1.61060000
C	33.98240000	-16.30510000	2.39320000
H	33.72090000	-17.32740000	2.12300000
H	34.54200000	-16.34350000	3.32800000
H	33.05540000	-15.76440000	2.58270000
O	29.69840000	-9.02620000	-20.37300000
H	29.41870000	-9.41350000	-21.20510000
O	29.60040000	-11.28060000	-21.64800000
H	30.15260000	-11.77660000	-22.24550000
O	32.15850000	-12.28430000	-21.56590000
H	31.80050000	-13.10180000	-21.20800000

5-alpha hexamer

C	29.89920	3.75620	-10.82850
C	27.07190	2.69140	-11.33880
O	29.83120	4.11740	-9.65420
O	27.09510	1.90310	-12.28260
N	30.73090	2.78630	-11.23770
N	26.69210	2.33140	-10.10390
H	30.72030	2.52020	-12.21480
H	26.73680	3.03880	-9.38400
C	28.96740	4.40720	-11.86650
H	29.15110	5.47670	-11.75250
C	27.46100	4.17600	-11.53070
H	27.26910	4.66150	-10.57130
C	29.31750	4.06530	-13.33510
H	30.36580	4.29220	-13.53610
H	29.19210	2.99940	-13.51220
C	26.54700	4.87470	-12.57270
H	25.51120	4.56690	-12.41820
C	28.43200	4.85370	-14.30990
H	28.64180	5.91920	-14.20190
C	26.94060	4.57530	-14.03980
H	26.72150	3.53160	-14.26700
C	26.15180	1.03440	-9.71040
H	25.19240	1.22410	-9.22820
H	25.93840	0.40350	-10.57450
C	31.60960	2.01720	-10.36920
H	32.38580	1.55570	-10.98040
H	32.11490	2.68910	-9.67320
C	27.08820	0.30260	-8.73590
H	27.98450	-0.02790	-9.26160
H	27.42000	0.99840	-7.96380
C	26.40160	-0.89880	-8.06570
H	25.46790	-0.55720	-7.61700
H	26.12510	-1.63810	-8.81740
C	27.26020	-1.55770	-6.97430
H	27.64840	-0.78220	-6.31290
H	28.12710	-2.04250	-7.42340
C	26.46870	-2.57290	-6.13410
H	25.56190	-2.09290	-5.76400
H	26.14100	-3.40200	-6.76160
C	27.26720	-3.10780	-4.93560
H	27.66140	-2.26390	-4.36850
H	28.12980	-3.67430	-5.28660
C	26.41800	-3.98060	-3.99940
H	25.51910	-3.43120	-3.71680
H	26.07940	-4.87110	-4.52970
C	27.17250	-4.38810	-2.72550
H	27.54160	-3.49140	-2.22670

H	28.05050	-4.97740	-2.99020
C	26.29510	-5.18000	-1.74570
H	25.39680	-4.60390	-1.52030
H	25.95770	-6.10210	-2.22000
C	27.02380	-5.50840	-0.43490
H	27.36900	-4.58310	0.02750
H	27.91580	-6.09660	-0.65090
C	26.13540	-6.26900	0.55930
H	25.23690	-5.68480	0.76200
H	25.79960	-7.20210	0.10580
C	26.85370	-6.56940	1.88250
H	27.18140	-5.63420	2.33780
H	27.75590	-7.14760	1.68250
C	25.96570	-7.33450	2.87360
H	25.06350	-6.75540	3.07450
H	25.63600	-8.26820	2.41700
C	26.68220	-7.63670	4.19740
H	26.99210	-6.70110	4.66430
H	27.59540	-8.19680	3.99580
C	25.80270	-8.42920	5.17490
H	24.89250	-7.86670	5.38610
H	25.48610	-9.36330	4.70980
C	26.51890	-8.74250	6.49300
H	26.81930	-7.82840	7.00560
H	25.86760	-9.30130	7.16530
H	27.41310	-9.34250	6.32370
C	30.82430	0.93670	-9.61090
H	30.40280	0.21730	-10.31460
H	29.97940	1.40300	-9.10290
C	31.68230	0.21260	-8.56830
H	32.46820	-0.36230	-9.05910
H	32.18440	0.95830	-7.95040
C	30.84480	-0.69960	-7.66460
H	30.44640	-1.53380	-8.24230
H	29.98370	-0.14040	-7.29640
C	31.64140	-1.21880	-6.46280
H	32.47390	-1.83360	-6.80470
H	32.08130	-0.37190	-5.93450
C	30.76760	-2.01440	-5.48730
H	30.38650	-2.90900	-5.97990
H	29.89720	-1.41630	-5.21580
C	31.52350	-2.39910	-4.21080
H	32.38770	-3.01230	-4.46610
H	31.91520	-1.49720	-3.73870
C	30.63360	-3.14540	-3.21120
H	30.26490	-4.06450	-3.66710
H	29.75630	-2.53830	-2.98610
C	31.36840	-3.47050	-1.90560
H	32.24970	-4.07390	-2.12360
H	31.73210	-2.54610	-1.45520
C	30.47410	-4.20740	-0.90190
H	30.11780	-5.13630	-1.34780
H	29.58930	-3.60470	-0.69500
C	31.19970	-4.51110	0.41460
H	32.09250	-5.10220	0.20940
H	31.54470	-3.57810	0.86210
C	30.30850	-5.25770	1.41480
H	29.96460	-6.19020	0.96670
H	29.41580	-4.66510	1.61760
C	31.03170	-5.55670	2.73440
H	31.93420	-6.13330	2.53070
H	31.36060	-4.62090	3.18800
C	30.14790	-6.32280	3.72740
H	29.81580	-7.25540	3.27080
H	29.24770	-5.74280	3.93340
C	30.87180	-6.62650	5.04630
H	31.77840	-7.19800	4.84460
H	31.19350	-5.69350	5.51040

C	29.99410	-7.40390	6.03240
H	29.68290	-8.36060	5.61330
H	30.53360	-7.60680	6.95760
H	29.09600	-6.84110	6.28770
O	26.58760	6.27470	-12.35940
H	26.14540	6.67710	-13.09480
O	26.15460	5.37410	-14.90300
H	26.53030	5.30010	-15.76900
O	28.75200	4.47390	-15.63410
H	28.43070	3.59330	-15.78660
C	30.93860	0.30800	-14.54520
C	28.14380	-0.96210	-14.49650
O	30.98700	1.08240	-13.59260
O	28.29370	-2.02260	-15.10030
N	31.72720	-0.77200	-14.63400
N	27.68980	-0.90840	-13.23610
H	31.60250	-1.39150	-15.42530
H	27.59700	0.00700	-12.81090
C	29.91590	0.56420	-15.66650
H	30.03970	1.62350	-15.89790
C	28.44300	0.38990	-15.18140
H	28.27050	1.14870	-14.42060
C	30.21700	-0.19530	-16.98090
H	31.24070	0.00130	-17.30350
H	30.13930	-1.26970	-16.82730
C	27.43610	0.67300	-16.32700
H	26.43360	0.37020	-16.01890
C	29.24110	0.22510	-18.08680
H	29.37890	1.28570	-18.30410
C	27.78810	-0.04720	-17.65050
H	27.64240	-1.12070	-17.52490
C	27.19720	-2.04550	-12.46880
H	26.38070	-1.68520	-11.84260
H	26.76240	-2.79580	-13.13160
C	32.67230	-1.20920	-13.61640
H	33.46500	-1.77350	-14.10850
H	33.14730	-0.34960	-13.14020
C	28.28520	-2.67890	-11.58680
H	29.09520	-3.06250	-12.20850
H	28.71960	-1.91410	-10.94120
C	27.70790	-3.81460	-10.72790
H	26.82570	-3.44250	-10.20680
H	27.35860	-4.62000	-11.37450
C	28.68950	-4.37840	-9.69000
H	29.10370	-3.55730	-9.10430
H	29.52910	-4.86060	-10.19110
C	28.00010	-5.37500	-8.74620
H	27.10050	-4.91010	-8.34260
H	27.66330	-6.24390	-9.31180
C	28.88410	-5.82890	-7.57610
H	29.27800	-4.95130	-7.06300
H	29.74420	-6.38390	-7.95160
C	28.10350	-6.68970	-6.57260
H	27.20700	-6.15040	-6.26550
H	27.75780	-7.59970	-7.06300
C	28.91590	-7.05340	-5.32230
H	29.28930	-6.14080	-4.85710
H	29.79060	-7.63830	-5.60790
C	28.08010	-7.83650	-4.30030
H	27.18950	-7.26010	-4.04880
H	27.72540	-8.76200	-4.75360
C	28.85140	-8.15820	-3.01320
H	29.21560	-7.23200	-2.56800
H	29.73190	-8.75390	-3.25410
C	27.98760	-8.90870	-1.99040
H	27.10030	-8.31740	-1.76390
H	27.62850	-9.83840	-2.43150
C	28.73820	-9.21620	-0.68780

H	29.09950	-8.28610	-0.24850
H	29.62040	-9.81680	-0.90950
C	27.86030	-9.95400	0.33180
H	26.97490	-9.35610	0.54750
H	27.49890	-10.88420	-0.10610
C	28.60030	-10.25780	1.64120
H	28.95960	-9.32650	2.07950
H	29.48400	-10.85970	1.42850
C	27.71510	-10.99140	2.65740
H	26.82890	-10.39400	2.86940
H	27.35440	-11.92400	2.22420
C	28.44660	-11.29100	3.96980
H	28.79270	-10.37400	4.44600
H	27.78860	-11.80210	4.67290
H	29.31450	-11.92870	3.80230
C	31.97160	-2.09080	-12.57330
H	31.52550	-2.95450	-13.06930
H	31.14830	-1.53260	-12.12510
C	32.91770	-2.56760	-11.46410
H	33.74190	-3.13430	-11.89890
H	33.36100	-1.70030	-10.97350
C	32.18930	-3.42900	-10.42550
H	31.80070	-4.32590	-10.90880
H	31.32420	-2.87900	-10.05480
C	33.07750	-3.82730	-9.23880
H	33.93220	-4.40390	-9.59420
H	33.48250	-2.92810	-8.77340
C	32.30120	-4.63900	-8.19300
H	31.92640	-5.55360	-8.65230
H	31.42460	-4.07010	-7.88330
C	33.13540	-4.99200	-6.95290
H	34.00080	-5.58560	-7.24930
H	33.52690	-4.07750	-6.50620
C	32.31420	-5.75910	-5.90630
H	31.93760	-6.68120	-6.34850
H	31.43760	-5.16970	-5.63780
C	33.10830	-6.08870	-4.63370
H	33.97500	-6.69830	-4.89150
H	33.49830	-5.16800	-4.19880
C	32.25360	-6.82240	-3.58960
H	31.87160	-7.74710	-4.02140
H	31.38070	-6.21660	-3.34790
C	33.02130	-7.14010	-2.29820
H	33.88960	-7.75650	-2.53350
H	33.40800	-6.21600	-1.86740
C	32.14730	-7.85950	-1.26020
H	31.76330	-8.78420	-1.69000
H	31.27630	-7.24590	-1.03150
C	32.90120	-8.17470	0.03950
H	33.77330	-8.78910	-0.18700
H	33.28130	-7.24920	0.47340
C	32.02090	-8.89700	1.06960
H	31.64180	-9.82230	0.63690
H	31.14700	-8.28600	1.29380
C	32.76970	-9.21100	2.37220
H	33.64920	-9.81720	2.15270
H	33.13760	-8.28530	2.81570
C	31.89360	-9.94500	3.39310
H	31.54960	-10.90230	3.00320
H	32.44600	-10.13980	4.31240
H	31.01380	-9.35780	3.65300
O	27.37690	2.06780	-16.56570
H	26.87730	2.18160	-17.36440
O	26.90540	0.39330	-18.66410
H	27.23890	0.06370	-19.48690
O	29.53400	-0.50290	-19.26250
H	29.23830	-1.39850	-19.15100
C	33.55600	-14.06550	-18.39890

C	30.95080	-15.58520	-18.12780
O	33.24800	-13.01040	-17.85010
O	31.19720	-16.79030	-18.12580
N	34.51690	-14.86680	-17.91820
N	30.36960	-14.95660	-17.09320
H	34.69630	-15.73890	-18.40000
H	30.23700	-13.95540	-17.17310
C	32.78350	-14.51480	-19.65610
H	32.86100	-13.67030	-20.34180
C	31.26040	-14.72530	-19.37360
H	30.84420	-13.74130	-19.15940
C	33.43360	-15.71720	-20.38030
H	34.47770	-15.50020	-20.61190
H	33.42760	-16.58650	-19.72850
C	30.50170	-15.26170	-20.62090
H	29.50620	-15.60080	-20.32800
C	32.68540	-16.06330	-21.67120
H	32.72990	-15.21340	-22.35450
C	31.22340	-16.42080	-21.34730
H	31.19640	-17.31320	-20.72130
C	29.75510	-15.61010	-15.94190
H	28.91730	-14.98740	-15.62630
H	29.32480	-16.56680	-16.24390
C	35.23420	-14.66230	-16.66820
H	36.17760	-15.20550	-16.73140
H	35.48420	-13.60820	-16.53470
C	30.71850	-15.81210	-14.75790
H	31.55970	-16.43440	-15.06660
H	31.13330	-14.85080	-14.45120
C	29.99410	-16.46950	-13.56890
H	29.13960	-15.85170	-13.28950
H	29.58280	-17.42670	-13.89170
C	30.86960	-16.69690	-12.32510
H	31.26070	-15.74200	-11.97270
H	31.73130	-17.30960	-12.59060
C	30.08140	-17.38100	-11.19380
H	29.21730	-16.76750	-10.93730
H	29.68180	-18.32800	-11.55770
C	30.90830	-17.64200	-9.92450
H	31.28400	-16.69650	-9.53380
H	31.78240	-18.24280	-10.17500
C	30.09180	-18.36020	-8.83690
H	29.21370	-17.76230	-8.59190
H	29.71480	-19.30360	-9.23270
C	30.89210	-18.63590	-7.55440
H	31.25250	-17.69380	-7.14210
H	31.77690	-19.22440	-7.79550
C	30.06370	-19.37680	-6.49290
H	29.17270	-18.79360	-6.25880
H	29.70920	-20.32210	-6.90470
C	30.84740	-19.64940	-5.20010
H	31.19360	-18.70500	-4.78080
H	31.74120	-20.22870	-5.43010
C	30.01240	-20.39920	-4.15130
H	29.11160	-19.82600	-3.93010
H	29.67560	-21.34940	-4.56670
C	30.78330	-20.65840	-2.84850
H	31.11980	-19.70910	-2.43240
H	31.68290	-21.23400	-3.06490
C	29.94130	-21.40430	-1.80320
H	29.03400	-20.83560	-1.59780
H	29.61650	-22.36100	-2.21330
C	30.70020	-21.64380	-0.49000
H	31.03090	-20.68860	-0.08290
H	31.60370	-22.21890	-0.69100
C	29.85050	-22.37880	0.55650
H	28.93910	-21.81350	0.75310
H	29.53340	-23.34290	0.15800

C	30.59720	-22.60040	1.87630
H	30.87600	-21.65330	2.33760
H	29.97600	-23.14530	2.58720
H	31.50850	-23.17730	1.72310
C	34.40750	-15.18170	-15.48330
H	34.11510	-16.21520	-15.67740
H	33.48260	-14.60850	-15.40450
C	35.16440	-15.11210	-14.15170
H	36.09920	-15.66630	-14.24220
H	35.43370	-14.07820	-13.93120
C	34.33830	-15.69000	-12.99660
H	33.99790	-16.68960	-13.26960
H	33.44200	-15.08530	-12.85410
C	35.12200	-15.77050	-11.68030
H	36.03770	-16.34010	-11.84130
H	35.42850	-14.77100	-11.37030
C	34.30190	-16.43190	-10.56560
H	33.95460	-17.40660	-10.90980
H	33.40950	-15.83620	-10.37170
C	35.09270	-16.61300	-9.26320
H	35.99670	-17.18810	-9.46470
H	35.42260	-15.64130	-8.89600
C	34.26770	-17.32270	-8.18130
H	33.92070	-18.28190	-8.56580
H	33.37430	-16.73630	-7.96600
C	35.05330	-17.55420	-6.88360
H	35.95260	-18.13070	-7.09950
H	35.39240	-16.59770	-6.48680
C	34.22080	-18.28650	-5.82250
H	33.87790	-19.23910	-6.22640
H	33.32490	-17.70580	-5.60230
C	34.99910	-18.53750	-4.52400
H	35.89700	-19.11540	-4.74230
H	35.34080	-17.58670	-4.11570
C	34.16040	-19.27590	-3.47220
H	33.82420	-20.22890	-3.88100
H	33.26090	-18.69880	-3.25720
C	34.93090	-19.52470	-2.16860
H	35.82930	-20.10390	-2.38130
H	35.27170	-18.57320	-1.76130
C	34.08680	-20.25870	-1.11800
H	33.75600	-21.21470	-1.52420
H	33.18420	-19.68330	-0.91110
C	34.85050	-20.49810	0.19150
H	35.75740	-21.06660	-0.01220
H	35.17640	-19.54510	0.60820
C	34.01120	-21.24360	1.23380
H	33.68940	-22.21420	0.85790
H	34.58420	-21.41440	2.14520
H	33.11980	-20.67640	1.50190
O	30.30280	-14.21280	-21.54810
H	29.97010	-14.61760	-22.33970
O	30.53580	-16.71690	-22.54780
H	31.08130	-17.31070	-23.04460
O	33.32470	-17.15840	-22.29600
H	33.08950	-17.95730	-21.83980
C	35.36060	-18.92910	-18.50620
C	32.67590	-20.26860	-17.92900
O	34.98230	-17.80050	-18.20020
O	32.92860	-21.42580	-17.59500
N	36.39010	-19.52450	-17.88880
N	32.03340	-19.40560	-17.12880
H	36.65580	-20.44740	-18.19950
H	31.87820	-18.46430	-17.47260
C	34.60460	-19.71700	-19.59650
H	34.75660	-19.13760	-20.50820
C	33.06500	-19.75060	-19.33060
H	32.70190	-18.72410	-19.38000

C	35.18920	-21.12900	-19.86440
H	36.24500	-21.05220	-20.12930
H	35.14200	-21.73540	-18.95880
C	32.31710	-20.54000	-20.43600
H	31.27270	-20.67450	-20.14780
C	34.44740	-21.84640	-21.00330
H	34.62820	-21.31580	-21.93990
C	32.93410	-21.92850	-20.72580
H	32.75590	-22.59090	-19.87810
C	31.39400	-19.75200	-15.86860
H	30.63910	-18.99480	-15.65480
H	30.85670	-20.69450	-15.98960
C	37.05280	-19.01280	-16.69720
H	38.01730	-19.51380	-16.61070
H	37.25860	-17.94520	-16.79440
C	32.38390	-19.84660	-14.69640
H	33.20320	-20.51960	-14.95230
H	32.82950	-18.86990	-14.50210
C	31.67520	-20.36790	-13.44010
H	30.86000	-19.69380	-13.17730
H	31.21050	-21.32600	-13.67720
C	32.59570	-20.55420	-12.22740
H	32.99280	-19.58940	-11.91030
H	33.45090	-21.17040	-12.50640
C	31.84350	-21.22450	-11.07020
H	30.97540	-20.62060	-10.80610
H	31.45240	-22.18430	-11.41060
C	32.70310	-21.45280	-9.82140
H	33.05930	-20.49540	-9.44070
H	33.58730	-22.03350	-10.08560
C	31.91700	-22.19030	-8.72970
H	31.01920	-21.62210	-8.48550
H	31.57380	-23.14900	-9.12070
C	32.73030	-22.42960	-7.45220
H	33.05970	-21.47330	-7.04560
H	33.63220	-22.99310	-7.69300
C	31.91990	-23.19040	-6.39480
H	31.00700	-22.63710	-6.17280
H	31.60390	-24.15080	-6.80400
C	32.70160	-23.42450	-5.09630
H	33.01550	-22.46540	-4.68430
H	33.61310	-23.98160	-5.31470
C	31.87440	-24.18710	-4.05290
H	30.95260	-23.64020	-3.85290
H	31.57560	-25.15280	-4.46250
C	32.63370	-24.40400	-2.73780
H	32.93640	-23.43870	-2.33200
H	33.55180	-24.95840	-2.93480
C	31.79510	-25.15780	-1.69670
H	30.86870	-24.61220	-1.51520
H	31.50560	-26.12980	-2.09800
C	32.54000	-25.35440	-0.36960
H	32.83960	-24.38290	0.02350
H	33.46020	-25.91150	-0.54900
C	31.69290	-26.09050	0.67770
H	30.76680	-25.54180	0.85200
H	31.40280	-27.06850	0.29220
C	32.42920	-26.27100	2.00960
H	32.70540	-25.30910	2.44210
H	31.80120	-26.79160	2.73280
H	33.34060	-26.85500	1.88020
C	36.19490	-19.29410	-15.45410
H	35.88000	-20.33880	-15.47450
H	35.28010	-18.70020	-15.49070
C	36.92860	-19.02310	-14.13600
H	37.87790	-19.56020	-14.14250
H	37.17000	-17.96340	-14.04610
C	36.09740	-19.48120	-12.93160

H	35.73360	-20.49150	-13.12240
H	35.21270	-18.85170	-12.83070
C	36.88920	-19.49060	-11.61870
H	37.81670	-20.04650	-11.76180
H	37.17530	-18.47490	-11.34440
C	36.08770	-20.14070	-10.48510
H	35.74620	-21.12370	-10.81140
H	35.19020	-19.55400	-10.28950
C	36.89660	-20.30360	-9.19300
H	37.81260	-20.85610	-9.40550
H	37.20400	-19.32480	-8.82390
C	36.09810	-21.04480	-8.11370
H	35.77890	-22.01170	-8.50440
H	35.18780	-20.48810	-7.89150
C	36.89640	-21.26290	-6.82260
H	37.80990	-21.81460	-7.04800
H	37.20900	-20.29910	-6.41990
C	36.08590	-22.02750	-5.76790
H	35.78020	-22.99150	-6.17600
H	35.16820	-21.48010	-5.55230
C	36.86480	-22.25110	-4.46530
H	37.77970	-22.80540	-4.67830
H	37.17530	-21.28800	-4.05890
C	36.03910	-23.00970	-3.41740
H	35.74360	-23.97900	-3.82040
H	35.11620	-22.46340	-3.22170
C	36.79810	-23.21440	-2.09950
H	37.71590	-23.77200	-2.29030
H	37.10350	-22.24520	-1.70410
C	35.95840	-23.95480	-1.04910
H	35.67650	-24.93500	-1.43540
H	35.02840	-23.41140	-0.88160
C	36.69660	-24.12360	0.28610
H	37.62310	-24.67590	0.12480
H	36.98430	-23.14550	0.67310
C	35.85180	-24.85050	1.33770
H	35.57500	-25.84910	0.99900
H	36.40190	-24.95740	2.27270
H	34.93450	-24.30290	1.55390
O	32.30730	-19.78230	-21.63160
H	31.98190	-20.35880	-22.31160
O	32.28760	-22.49260	-21.85050
H	32.78520	-23.25590	-22.10880
O	34.96590	-23.15730	-21.12040
H	34.81600	-23.61550	-20.30680
C	31.52380	-4.24970	-16.82330
C	28.70600	-5.44270	-16.64900
O	31.42510	-3.32450	-16.02120
O	28.74670	-6.62320	-16.99430
N	32.39250	-5.25380	-16.65170
N	28.29260	-5.06310	-15.43040
H	32.38830	-6.00630	-17.32760
H	28.31930	-4.07330	-15.21390
C	30.58990	-4.28900	-18.04630
H	30.76360	-3.33340	-18.54360
C	29.08530	-4.31320	-17.63550
H	28.88560	-3.37880	-17.11410
C	30.94290	-5.38660	-19.07910
H	31.99060	-5.30680	-19.37340
H	30.81560	-6.37470	-18.63860
C	28.16150	-4.32190	-18.88200
H	27.13180	-4.51450	-18.57490
C	30.05630	-5.26580	-20.32560
H	30.25500	-4.31160	-20.81650
C	28.56650	-5.37030	-19.94390
H	28.35980	-6.36640	-19.55360
C	27.65530	-5.94050	-14.45510
H	26.96660	-5.33250	-13.86780

H	27.04080	-6.68130	-14.97010
C	33.20560	-5.46030	-15.46340
H	34.04780	-6.09580	-15.73870
H	33.62220	-4.51550	-15.10970
C	28.65530	-6.63970	-13.51780
H	29.38520	-7.20480	-14.09930
H	29.21400	-5.88860	-12.95740
C	27.92570	-7.58390	-12.54700
H	27.12400	-7.03170	-12.05510
H	27.44180	-8.37880	-13.11600
C	28.82440	-8.20660	-11.46780
H	29.31030	-7.40910	-10.90490
H	29.61900	-8.79140	-11.93230
C	28.01920	-9.09520	-10.50480
H	27.16150	-8.53170	-10.13560
H	27.61130	-9.94760	-11.04900
C	28.83250	-9.59440	-9.30200
H	29.26990	-8.73840	-8.78810
H	29.66460	-10.20950	-9.64520
C	27.97170	-10.39160	-8.30950
H	27.10360	-9.79470	-8.02760
H	27.58190	-11.28490	-8.79810
C	28.73530	-10.79080	-7.03900
H	29.14310	-9.89570	-6.56960
H	29.58790	-11.41540	-7.30490
C	27.84830	-11.53140	-6.02740
H	26.98080	-10.91510	-5.78850
H	27.46010	-12.44340	-6.48130
C	28.59250	-11.87980	-4.73070
H	28.98470	-10.96690	-4.28300
H	29.45590	-12.50270	-4.96310
C	27.69900	-12.60150	-3.71200
H	26.83040	-11.98100	-3.48840
H	27.31370	-13.52120	-4.15330
C	28.43810	-12.93060	-2.40730
H	28.82140	-12.01090	-1.96600
H	29.30780	-13.54860	-2.62870
C	27.54500	-13.65150	-1.38800
H	26.67140	-13.03540	-1.17190
H	27.16830	-14.57640	-1.82590
C	28.28090	-13.96880	-0.07870
H	28.65270	-13.04450	0.36260
H	29.15840	-14.57790	-0.29410
C	27.39030	-14.69800	0.93670
H	26.50690	-14.09500	1.14940
H	27.02840	-15.63020	0.50200
C	28.11970	-15.00340	2.24940
H	28.44530	-14.08880	2.74390
H	27.46960	-15.54080	2.93990
H	29.00170	-15.61890	2.07670
C	32.37210	-6.13630	-14.36630
H	31.91130	-7.03970	-14.76730
H	31.55160	-5.48020	-14.07200
C	33.19590	-6.50380	-13.13050
H	34.04450	-7.12180	-13.42700
H	33.60700	-5.59930	-12.68060
C	32.34830	-7.26020	-12.10390
H	31.94200	-8.16150	-12.56530
H	31.49400	-6.64550	-11.81840
C	33.14050	-7.64480	-10.85210
H	33.99420	-8.26210	-11.13380
H	33.54600	-6.74570	-10.38710
C	32.27020	-8.39840	-9.84250
H	31.88140	-9.30660	-10.30460
H	31.40560	-7.78640	-9.58450
C	33.03370	-8.75820	-8.56540
H	33.89190	-9.38170	-8.81670
H	33.43360	-7.85030	-8.11330

C	32.14470	-9.48500	-7.55190
H	31.75690	-10.40060	-7.99940
H	31.27970	-8.86360	-7.31880
C	32.89130	-9.82050	-6.25740
H	33.75510	-10.44400	-6.48740
H	33.28340	-8.90420	-5.81620
C	31.99530	-10.53480	-5.24030
H	31.61170	-11.45630	-5.67900
H	31.12780	-9.91200	-5.02120
C	32.73480	-10.85510	-3.93700
H	33.60520	-11.47300	-4.15670
H	33.11780	-9.93310	-3.49980
C	31.83980	-11.57100	-2.91930
H	31.46280	-12.49590	-3.35640
H	30.96800	-10.95270	-2.70490
C	32.57690	-11.88490	-1.61220
H	33.45360	-12.49500	-1.82840
H	32.95090	-10.95990	-1.17370
C	31.68500	-12.60970	-0.59720
H	31.31500	-13.53580	-1.03770
H	30.80830	-11.99830	-0.38280
C	32.42050	-12.92390	0.71130
H	33.30190	-13.52820	0.49920
H	32.78730	-12.00030	1.15790
C	31.53280	-13.65850	1.72000
H	31.17640	-14.60420	1.31290
H	32.08110	-13.87590	2.63670
H	30.66170	-13.06020	1.98620
O	28.16610	-3.04060	-19.48360
H	27.72570	-3.13690	-20.31880
O	27.77430	-5.18660	-21.10160
H	28.14110	-5.73510	-21.78060
O	30.38950	-6.30200	-21.22730
H	30.03990	-7.11850	-20.89050
C	32.29780	-9.17380	-17.81540
C	29.63460	-10.64440	-17.56600
O	32.11580	-8.14660	-17.16810
O	29.82650	-11.84560	-17.75560
N	33.21330	-10.08850	-17.46830
N	29.14080	-10.16190	-16.41520
H	33.27410	-10.93340	-18.02220
H	29.04260	-9.15800	-16.33490
C	31.41910	-9.44340	-19.05050
H	31.49560	-8.52670	-19.63660
C	29.91220	-9.60990	-18.68070
H	29.58000	-8.65160	-18.28250
C	31.94500	-10.57480	-19.96330
H	32.98690	-10.39210	-20.23140
H	31.91630	-11.52650	-19.43690
C	29.04260	-9.88990	-19.93680
H	28.04160	-10.19880	-19.62960
C	31.09860	-10.67970	-21.23710
H	31.17400	-9.74710	-21.79900
C	29.62980	-10.97140	-20.87230
H	29.56230	-11.94040	-20.37830
C	28.59400	-10.96990	-15.33100
H	27.80630	-10.38550	-14.85440
H	28.10870	-11.85970	-15.73670
C	34.03770	-10.04570	-16.26850
H	34.94910	-10.60910	-16.47010
H	34.34270	-9.02220	-16.04300
C	29.64550	-11.37020	-14.28170
H	30.44670	-11.94140	-14.75280
H	30.10280	-10.47280	-13.86260
C	29.00740	-12.20310	-13.15740
H	28.15130	-11.65720	-12.75920
H	28.60910	-13.12720	-13.57850
C	29.95690	-12.54330	-11.99810

H	30.37010	-11.62230	-11.58580
H	30.79900	-13.12900	-12.36780
C	29.23230	-13.32080	-10.88770
H	28.35720	-12.75270	-10.57100
H	28.85430	-14.25970	-11.29350
C	30.10660	-13.61340	-9.65890
H	30.49610	-12.67700	-9.25880
H	30.96950	-14.21000	-9.95500
C	29.32290	-14.34950	-8.56150
H	28.44710	-13.75940	-8.29070
H	28.94320	-15.29070	-8.95950
C	30.15150	-14.62960	-7.29890
H	30.52850	-13.69000	-6.89460
H	31.02490	-15.22750	-7.55920
C	29.33500	-15.35810	-6.22130
H	28.45470	-14.76460	-5.97380
H	28.96250	-16.29950	-6.62510
C	30.13520	-15.63630	-4.94070
H	30.50020	-14.69570	-4.52790
H	31.01680	-16.22980	-5.18250
C	29.30090	-16.37010	-3.88110
H	28.41280	-15.78200	-3.64900
H	28.94180	-17.31350	-4.29250
C	30.08230	-16.64330	-2.58850
H	30.43500	-15.70030	-2.17080
H	30.97170	-17.23050	-2.81740
C	29.23890	-17.38260	-1.54060
H	28.34330	-16.80150	-1.32020
H	28.89290	-18.32920	-1.95570
C	30.00860	-17.64690	-0.23940
H	30.35010	-16.70030	0.17940
H	30.90530	-18.22650	-0.45930
C	29.16300	-18.39110	0.80300
H	28.26220	-17.81810	1.02390
H	28.82490	-19.34080	0.38890
C	29.92780	-18.65170	2.10500
H	30.23230	-17.71840	2.57870
H	29.30880	-19.19950	2.81580
H	30.82590	-19.24170	1.92370
C	33.29030	-10.66620	-15.07940
H	32.93780	-11.66090	-15.35760
H	32.40090	-10.07350	-14.86070
C	34.15570	-10.77420	-13.81710
H	35.05650	-11.34540	-14.04490
H	34.48400	-9.78060	-13.50920
C	33.40110	-11.45080	-12.66600
H	33.03350	-12.42080	-13.00320
H	32.52270	-10.85530	-12.41460
C	34.26180	-11.65000	-11.41090
H	35.14430	-12.23810	-11.66530
H	34.62290	-10.68440	-11.05540
C	33.48300	-12.35340	-10.29170
H	33.10630	-13.30660	-10.66430
H	32.60890	-11.75500	-10.03360
C	34.32200	-12.60020	-9.03000
H	35.19570	-13.20030	-9.28590
H	34.70050	-11.65120	-8.64960
C	33.51540	-13.30800	-7.93250
H	33.13720	-14.25410	-8.32030
H	32.64090	-12.70630	-7.68450
C	34.32910	-13.56990	-6.65690
H	35.20530	-14.17090	-6.90060
H	34.70570	-12.62600	-6.26230
C	33.50200	-14.28200	-5.57700
H	33.12970	-15.22640	-5.97400
H	32.62340	-13.68190	-5.34070
C	34.29600	-14.54810	-4.29000
H	35.17850	-15.14420	-4.52300

H	34.66320	-13.60480	-3.88540
C	33.45780	-15.26910	-3.22490
H	33.09590	-16.21430	-3.62910
H	32.57270	-14.67520	-2.99730
C	34.23910	-15.53390	-1.93020
H	35.12720	-16.12480	-2.15540
H	34.59730	-14.58920	-1.52070
C	33.39540	-16.26090	-0.87390
H	33.04210	-17.20770	-1.28180
H	32.50480	-15.67310	-0.65290
C	34.17000	-16.52220	0.42530
H	35.05780	-17.11640	0.20870
H	34.52830	-15.57900	0.83840
C	33.32380	-17.24360	1.47920
H	32.96150	-18.20040	1.10500
H	33.90610	-17.43850	2.37990
H	32.45750	-16.64800	1.76590
O	28.87970	-8.69820	-20.68120
H	28.46040	-8.94890	-21.49500
O	28.85470	-11.03300	-22.05450
H	29.31490	-11.59550	-22.66170
O	31.61130	-11.71790	-22.04680
H	31.32190	-12.55050	-21.69290

5-alpha octamer

C	29.32000	7.53480	-7.04560
C	26.43450	6.53130	-7.36360
O	29.43510	7.99650	-5.91300
O	26.51310	5.70370	-8.27110
N	29.99200	6.44760	-7.45160
N	25.96620	6.22550	-6.14380
H	29.82500	6.09690	-8.38730
H	25.94360	6.96920	-5.45900
C	28.33790	8.20390	-8.01980
H	28.54820	9.26940	-7.91220
C	26.85280	8.00360	-7.58900
H	26.74470	8.49060	-6.62130
C	28.59120	7.87490	-9.50820
H	29.63320	8.06910	-9.76740
H	28.41130	6.81970	-9.69830
C	25.88700	8.72200	-8.56820
H	24.85850	8.42560	-8.35430
C	27.66940	8.70990	-10.40550
H	27.89920	9.76840	-10.27260
C	26.19300	8.43220	-10.05530
H	25.95810	7.38870	-10.26400
C	25.37850	4.94760	-5.75150
H	24.49200	5.17230	-5.15770
H	25.02700	4.39180	-6.62280
C	30.86970	5.65090	-6.60460
H	31.57010	5.11080	-7.24230
H	31.46640	6.30420	-5.96530
C	26.34700	4.08340	-4.92600
H	27.20590	3.80250	-5.53580
H	26.73510	4.67250	-4.09380
C	25.66080	2.81650	-4.38370
H	24.73480	3.10910	-3.88700
H	25.37040	2.17030	-5.21340
C	26.52190	2.02300	-3.38680
H	26.90260	2.70630	-2.62720
H	27.39190	1.60610	-3.89390
C	25.73370	0.89680	-2.69620
H	24.80650	1.31120	-2.29900
H	25.44140	0.14460	-3.42970
C	26.50800	0.23080	-1.54680
H	26.90290	1.00550	-0.88950

H	27.36950	-0.30800	-1.94140
C	25.63160	-0.72250	-0.71870
H	24.73600	-0.18980	-0.39680
H	25.28880	-1.54590	-1.34570
C	26.35110	-1.27870	0.52040
H	26.75300	-0.45010	1.10380
H	27.20560	-1.88010	0.21040
C	25.42390	-2.11690	1.41420
H	24.55380	-1.51920	1.68810
H	25.04460	-2.96840	0.84870
C	26.11350	-2.61490	2.69360
H	26.51120	-1.76300	3.24490
H	26.96930	-3.23540	2.42880
C	25.16460	-3.40770	3.60520
H	24.30150	-2.78980	3.85470
H	24.77620	-4.26940	3.06200
C	25.83900	-3.88140	4.90120
H	26.22820	-3.02050	5.44410
H	26.70000	-4.50280	4.65620
C	24.88240	-4.66490	5.81230
H	24.02130	-4.04230	6.05620
H	24.49200	-5.52670	5.27100
C	25.55180	-5.13800	7.11070
H	25.93630	-4.27630	7.65580
H	26.41590	-5.75680	6.86970
C	24.59400	-5.92580	8.01590
H	23.73190	-5.30620	8.26390
H	24.20400	-6.78880	7.47590
C	25.26030	-6.40340	9.31100
H	25.64010	-5.56450	9.89300
H	24.55000	-6.94480	9.93600
H	26.09680	-7.07070	9.10450
C	30.05560	4.65970	-5.75950
H	29.54660	3.95350	-6.41750
H	29.27450	5.20140	-5.22390
C	30.91100	3.89740	-4.73960
H	31.68150	3.32070	-5.25290
H	31.42980	4.61720	-4.10510
C	30.06110	2.96820	-3.86350
H	29.63790	2.17510	-4.48100
H	29.21800	3.53210	-3.46250
C	30.84660	2.35510	-2.69640
H	31.66030	1.73930	-3.08090
H	31.30950	3.15380	-2.11570
C	29.94740	1.51780	-1.77720
H	29.52300	0.68970	-2.34520
H	29.10670	2.12880	-1.44660
C	30.68470	0.97390	-0.54590
H	31.51300	0.34070	-0.86440
H	31.12710	1.80260	0.00730
C	29.75370	0.18250	0.38320
H	29.33280	-0.66200	-0.16260
H	28.91150	0.81160	0.67210
C	30.45930	-0.32490	1.64820
H	31.30050	-0.95890	1.36760
H	30.88200	0.51920	2.19280
C	29.51150	-1.10210	2.57190
H	29.10550	-1.95850	2.03370
H	28.66080	-0.47250	2.83160
C	30.19510	-1.58280	3.85900
H	31.05150	-2.20720	3.60380
H	30.59390	-0.72630	4.40210
C	29.24050	-2.36470	4.77170
H	28.84560	-3.22320	4.22850
H	28.38340	-1.73980	5.02220
C	29.91500	-2.84410	6.06410
H	30.78190	-3.45570	5.81430
H	30.29770	-1.98590	6.61610

C	28.96150	-3.64410	6.96230
H	28.56570	-4.49270	6.40470
H	28.10450	-3.02360	7.22420
C	29.63820	-4.14800	8.24460
H	30.49230	-4.77560	7.98930
H	30.03730	-3.30220	8.80400
C	28.68290	-4.94000	9.14310
H	28.31460	-5.83240	8.63750
H	29.18300	-5.25870	10.05770
H	27.82080	-4.33830	9.42910
O	25.95360	10.12100	-8.36560
H	25.44670	10.51420	-9.06490
O	25.35820	9.23710	-10.86570
H	25.66550	9.14940	-11.75700
O	27.91600	8.37900	-11.75680
H	27.49170	7.55110	-11.94800
C	28.83180	10.53210	-2.90930
C	25.86970	9.82740	-3.60770
O	28.77360	10.62070	-1.68320
O	25.44230	9.45230	-4.69830
N	29.64790	9.66630	-3.52880
N	25.85400	9.03670	-2.52510
H	29.61390	9.60720	-4.53810
H	26.24690	9.40940	-1.67160
C	27.91850	11.42650	-3.76500
H	28.20600	12.43320	-3.45740
C	26.40990	11.26460	-3.40850
H	26.28990	11.48190	-2.34500
C	28.17050	11.35830	-5.28880
H	29.23230	11.48250	-5.50690
H	27.88490	10.38040	-5.67670
C	25.56670	12.32090	-4.17490
H	24.50410	12.11490	-4.03500
C	27.37770	12.46390	-6.00500
H	27.76210	13.43630	-5.69200
C	25.86830	12.37110	-5.69500
H	25.45770	11.48100	-6.16820
C	25.24930	7.71100	-2.45280
H	24.25040	7.82920	-2.03140
H	25.12270	7.26680	-3.44120
C	30.53280	8.72690	-2.85230
H	31.28580	8.38110	-3.56110
H	31.06700	9.24400	-2.05320
C	26.08840	6.77560	-1.57270
H	27.01680	6.52500	-2.08560
H	26.36890	7.30010	-0.65800
C	25.34480	5.48500	-1.20020
H	24.38120	5.75280	-0.76490
H	25.12640	4.90070	-2.09440
C	26.12860	4.63250	-0.19170
H	26.48760	5.28080	0.60840
H	27.01490	4.21000	-0.66550
C	25.28080	3.51450	0.43030
H	24.34900	3.94470	0.79980
H	25.00230	2.78450	-0.32990
C	25.99920	2.81830	1.59470
H	26.39390	3.57750	2.27080
H	26.85890	2.25990	1.22380
C	25.07260	1.89040	2.39060
H	24.18050	2.44450	2.68540
H	24.73050	1.07240	1.75640
C	25.74830	1.33180	3.64960
H	26.15020	2.15760	4.23770
H	26.59930	0.71270	3.36630
C	24.78230	0.52260	4.52370
H	23.91840	1.14000	4.77300
H	24.39990	-0.32630	3.95680
C	25.43840	0.02880	5.81930

H	25.83430	0.88120	6.37250
H	26.29170	-0.60410	5.57650
C	24.45960	-0.74350	6.71290
H	23.60670	-0.10680	6.95130
H	24.06210	-1.59610	6.16210
C	25.10990	-1.23010	8.01430
H	25.50120	-0.37520	8.56680
H	25.96600	-1.86150	7.77660
C	24.13010	-2.00660	8.90400
H	23.27880	-1.37010	9.14860
H	23.72990	-2.85380	8.34670
C	24.78110	-2.50830	10.19990
H	25.16580	-1.65860	10.76490
H	25.64220	-3.13000	9.95450
C	23.80660	-3.30630	11.07750
H	22.94810	-2.68330	11.33070
H	23.41570	-4.15420	10.51430
C	24.45710	-3.81680	12.36780
H	24.82200	-2.99090	12.97880
H	23.74170	-4.38140	12.96600
H	25.30040	-4.47340	12.15270
C	29.74410	7.53210	-2.29570
H	29.31810	6.95010	-3.11440
H	28.90220	7.91050	-1.71570
C	30.58540	6.62810	-1.38720
H	31.35390	6.11410	-1.96570
H	31.10830	7.25350	-0.66240
C	29.71490	5.61430	-0.63310
H	29.32150	4.87050	-1.32660
H	28.85090	6.13480	-0.21850
C	30.45880	4.92790	0.51840
H	31.26050	4.30110	0.12710
H	30.93570	5.68810	1.13850
C	29.51310	4.09760	1.39470
H	29.09590	3.27770	0.81040
H	28.66970	4.71840	1.69870
C	30.19770	3.55230	2.65290
H	31.02360	2.89980	2.36910
H	30.63680	4.37960	3.21190
C	29.21860	2.79600	3.55930
H	28.79770	1.95180	3.01340
H	28.38130	3.44780	3.81110
C	29.87550	2.30100	4.85310
H	30.71970	1.65580	4.60840
H	30.28620	3.15090	5.39960
C	28.88980	1.54480	5.75300
H	28.47870	0.69700	5.20480
H	28.04680	2.19300	5.99490
C	29.53990	1.04950	7.05100
H	30.39500	0.41780	6.80860
H	29.93430	1.90030	7.60810
C	28.55950	0.26880	7.93640
H	28.15300	-0.56980	7.37090
H	27.71290	0.90680	8.19270
C	29.21540	-0.25300	9.22180
H	30.07230	-0.87540	8.96250
H	29.60810	0.58650	9.79670
C	28.24240	-1.05920	10.09320
H	27.83110	-1.88220	9.50890
H	27.39800	-0.42820	10.37290
C	28.90770	-1.61630	11.35940
H	29.75280	-2.24770	11.08270
H	29.31660	-0.79520	11.94930
C	27.93860	-2.42650	12.22660
H	27.54370	-3.28370	11.68140
H	28.43770	-2.80360	13.11940
H	27.09520	-1.81620	12.55010
O	25.80610	13.60070	-3.61560

H	25.40500	14.23220	-4.19750
O	25.21120	13.48680	-6.26540
H	25.53490	13.57760	-7.15040
O	27.57760	12.33500	-7.39840
H	27.16160	11.53060	-7.68550
C	29.62060	4.06990	-10.81240
C	26.80460	2.96840	-11.10360
O	29.62580	4.60510	-9.70620
O	26.88040	2.03380	-11.90100
N	30.38910	3.00590	-11.09180
N	26.38250	2.81810	-9.83820
H	30.33240	2.59430	-12.01610
H	26.38620	3.64140	-9.24840
C	28.67280	4.61230	-11.90100
H	28.84840	5.68870	-11.89950
C	27.16710	4.40590	-11.53880
H	26.96750	5.03510	-10.67240
C	29.02190	4.12870	-13.32600
H	30.06840	4.33890	-13.55310
H	28.89300	3.05220	-13.38990
C	26.22070	4.90140	-12.66790
H	25.20590	4.54550	-12.48000
C	28.12100	4.79570	-14.37090
H	28.27560	5.87580	-14.34720
C	26.64710	4.45250	-14.08330
H	26.49810	3.37660	-14.17250
C	25.81450	1.60210	-9.26490
H	24.90440	1.89180	-8.73880
H	25.50520	0.90420	-10.04510
C	31.27130	2.34740	-10.13620
H	32.00340	1.75860	-10.68990
H	31.83150	3.09770	-9.57530
C	26.77690	0.90920	-8.28610
H	27.65780	0.55630	-8.82290
H	27.12770	1.63120	-7.54680
C	26.10040	-0.27190	-7.56910
H	25.18480	0.08530	-7.09580
H	25.79410	-1.01770	-8.30380
C	26.98100	-0.93850	-6.50000
H	27.34830	-0.17630	-5.81160
H	27.85750	-1.38560	-6.96900
C	26.21730	-2.01130	-5.70640
H	25.30520	-1.56900	-5.30390
H	25.89970	-2.80920	-6.37860
C	27.03280	-2.60760	-4.54840
H	27.41460	-1.79710	-3.92720
H	27.90220	-3.13540	-4.94090
C	26.20100	-3.55980	-3.67540
H	25.29860	-3.04270	-3.34720
H	25.86720	-4.40860	-4.27310
C	26.96300	-4.06560	-2.44100
H	27.34240	-3.21150	-1.87980
H	27.83390	-4.64120	-2.75540
C	26.08300	-4.92270	-1.51910
H	25.19350	-4.35460	-1.24460
H	25.73090	-5.80000	-2.06270
C	26.80890	-5.36790	-0.24100
H	27.17680	-4.48940	0.28900
H	27.68630	-5.95840	-0.50440
C	25.90340	-6.18230	0.69410
H	25.01720	-5.59560	0.93830
H	25.54790	-7.07130	0.17220
C	26.60730	-6.60020	1.99300
H	26.96190	-5.71200	2.51500
H	27.49280	-7.18910	1.75530
C	25.69050	-7.40680	2.92340
H	24.80220	-6.81840	3.15510
H	25.33970	-8.29850	2.40320

C	26.38280	-7.81920	4.22990
H	26.72810	-6.92930	4.75480
H	27.27500	-8.40130	4.00220
C	25.46120	-8.63080	5.15120
H	24.57790	-8.04000	5.39420
H	25.10110	-9.51600	4.62630
C	26.15220	-9.06330	6.44860
H	26.52330	-8.20310	7.00400
H	25.46100	-9.60340	7.09570
H	26.99870	-9.71890	6.24710
C	30.47800	1.43890	-9.18310
H	30.00960	0.63470	-9.75260
H	29.66640	2.01030	-8.73060
C	31.34170	0.84880	-8.06070
H	32.14300	0.24340	-8.48630
H	31.82070	1.66270	-7.51460
C	30.51190	0.00400	-7.08530
H	30.10720	-0.86030	-7.61330
H	29.65640	0.58870	-6.74430
C	31.31100	-0.46540	-5.86210
H	32.16110	-1.06580	-6.18730
H	31.72240	0.40170	-5.34360
C	30.44530	-1.27680	-4.88900
H	30.04970	-2.15160	-5.40580
H	29.58440	-0.67790	-4.58920
C	31.20690	-1.72650	-3.63480
H	32.06830	-2.32710	-3.92730
H	31.60200	-0.85260	-3.11620
C	30.31860	-2.53290	-2.67710
H	29.92600	-3.40560	-3.19970
H	29.45580	-1.93000	-2.39190
C	31.05880	-2.98840	-1.41200
H	31.92490	-3.58750	-1.69250
H	31.44740	-2.11750	-0.88440
C	30.15810	-3.79870	-0.46910
H	29.78100	-4.67600	-0.99520
H	29.28620	-3.20180	-0.20140
C	30.88030	-4.24260	0.81040
H	31.75680	-4.83410	0.54720
H	31.25270	-3.36650	1.34050
C	29.97330	-5.05640	1.74380
H	29.61440	-5.94190	1.21870
H	29.09050	-4.46800	1.99380
C	30.68210	-5.48120	3.03710
H	31.57160	-6.06040	2.79120
H	31.03390	-4.59630	3.56640
C	29.77430	-6.30160	3.96360
H	29.43140	-7.19380	3.43940
H	28.88180	-5.72240	4.20080
C	30.47490	-6.71140	5.26620
H	31.36810	-7.29310	5.03890
H	30.82070	-5.82240	5.79160
C	29.56380	-7.52140	6.19400
H	29.24450	-8.44980	5.72150
H	30.07970	-7.77870	7.11920
H	28.66980	-6.95760	6.45840
O	26.15190	6.31320	-12.65460
H	25.68400	6.56390	-13.44150
O	25.81980	5.08540	-15.04100
H	26.19660	4.91400	-15.89280
O	28.48120	4.33210	-15.65640
H	28.12050	3.46240	-15.78000
C	30.48160	0.40300	-14.35080
C	27.68850	-0.83990	-14.26620
O	30.51920	1.18370	-13.40280
O	27.82570	-1.92480	-14.82880
N	31.28020	-0.67140	-14.41750
N	27.24870	-0.72890	-13.00430

H	31.17890	-1.30200	-15.20390
H	27.18180	0.20300	-12.61050
C	29.46520	0.64230	-15.48420
H	29.59470	1.69510	-15.73880
C	27.98520	0.48040	-15.01030
H	27.79610	1.27110	-14.28520
C	29.77710	-0.14820	-16.77690
H	30.80360	0.03850	-17.09640
H	29.69370	-1.21630	-16.59310
C	26.98280	0.69780	-16.17710
H	25.98400	0.38440	-15.86740
C	28.81000	0.23360	-17.90260
H	28.93240	1.29130	-18.14180
C	27.35990	-0.05840	-17.47160
H	27.23610	-1.12900	-17.30780
C	26.74210	-1.82180	-12.18470
H	25.93210	-1.41970	-11.57520
H	26.29690	-2.59680	-12.81120
C	32.22020	-1.07080	-13.37960
H	32.98200	-1.70680	-13.83160
H	32.73690	-0.19490	-12.98300
C	27.82280	-2.42520	-11.27390
H	28.62730	-2.84600	-11.87850
H	28.26680	-1.63710	-10.66360
C	27.23330	-3.51410	-10.36450
H	26.37470	-3.10050	-9.83360
H	26.84890	-4.32960	-10.97840
C	28.22540	-4.07360	-9.33500
H	28.63590	-3.25160	-8.74700
H	29.06480	-4.54660	-9.84520
C	27.55330	-5.08610	-8.39610
H	26.65970	-4.63070	-7.96760
H	27.21160	-5.94700	-8.97200
C	28.46190	-5.55900	-7.25300
H	28.83850	-4.69070	-6.71130
H	29.33130	-6.07740	-7.65820
C	27.71720	-6.47970	-6.27650
H	26.80650	-5.98110	-5.94280
H	27.39820	-7.38280	-6.79790
C	28.55270	-6.86120	-5.04740
H	28.89890	-5.95420	-4.55100
H	29.44440	-7.40440	-5.36030
C	27.75240	-7.71060	-4.05130
H	26.84150	-7.17750	-3.77740
H	27.43320	-8.63380	-4.53560
C	28.54060	-8.04510	-2.77840
H	28.86970	-7.12090	-2.30270
H	29.44310	-8.59700	-3.04020
C	27.70880	-8.86430	-1.78300
H	26.79930	-8.31580	-1.53690
H	27.38740	-9.79220	-2.25620
C	28.47220	-9.18700	-0.49220
H	28.79340	-8.25880	-0.01900
H	29.37970	-9.74010	-0.73350
C	27.62640	-10.00040	0.49600
H	26.71500	-9.45010	0.72980
H	27.30790	-10.92930	0.02320
C	28.37760	-10.31970	1.79490
H	28.69520	-9.39010	2.26740
H	29.28770	-10.87290	1.56330
C	27.52530	-11.13010	2.78030
H	26.61090	-10.58320	3.00840
H	27.21000	-12.06270	2.31370
C	28.26820	-11.44090	4.08350
H	28.57510	-10.52610	4.58910
H	27.63300	-12.00310	4.76820
H	29.16350	-12.03360	3.89770
C	31.49850	-1.83100	-12.25800

H	31.05510	-2.74040	-12.66690
H	30.67160	-1.22730	-11.88050
C	32.42410	-2.18580	-11.08880
H	33.26170	-2.78420	-11.44900
H	32.84840	-1.27080	-10.67320
C	31.67780	-2.94990	-9.99010
H	31.29390	-3.88280	-10.40440
H	30.80990	-2.36960	-9.67390
C	32.55170	-3.25580	-8.76790
H	33.43120	-3.82120	-9.07790
H	32.91660	-2.32310	-8.33620
C	31.77870	-4.04740	-7.70680
H	31.41430	-4.97430	-8.15040
H	30.89710	-3.48010	-7.40650
C	32.61800	-4.37630	-6.46600
H	33.50610	-4.93520	-6.76320
H	32.97210	-3.45240	-6.00790
C	31.81930	-5.18880	-5.43890
H	31.46150	-6.10570	-5.90780
H	30.93240	-4.62610	-5.14630
C	32.63280	-5.54380	-4.18760
H	33.52160	-6.10540	-4.47720
H	32.98770	-4.62950	-3.71130
C	31.81260	-6.36280	-3.18200
H	31.46080	-7.27560	-3.66280
H	30.92140	-5.80100	-2.90150
C	32.60440	-6.72630	-1.91880
H	33.49560	-7.29010	-2.19690
H	32.95640	-5.81500	-1.43460
C	31.76880	-7.54480	-0.92500
H	31.42350	-8.45810	-1.40890
H	30.87330	-6.98360	-0.65800
C	32.54400	-7.90530	0.34970
H	33.43980	-8.46770	0.08400
H	32.88850	-6.99250	0.83640
C	31.69940	-8.72340	1.33630
H	31.36360	-9.64050	0.85340
H	30.79770	-8.16630	1.58990
C	32.46200	-9.07270	2.62150
H	33.36650	-9.62860	2.37280
H	32.79170	-8.15700	3.11270
C	31.61650	-9.89370	3.60090
H	31.31980	-10.84670	3.16550
H	32.17160	-10.10420	4.51500
H	30.70730	-9.36190	3.87830
O	26.89120	2.07700	-16.47760
H	26.39920	2.14110	-17.28680
O	26.47590	0.32640	-18.50700
H	26.81810	-0.03230	-19.31390
O	29.12900	-0.51470	-19.05850
H	28.81560	-1.40380	-18.94370
C	33.28440	-13.71930	-18.21920
C	30.78480	-15.40870	-17.94380
O	32.91500	-12.68610	-17.66670
O	31.12150	-16.59180	-17.94610
N	34.29490	-14.46000	-17.74330
N	30.15690	-14.82970	-16.90740
H	34.52760	-15.31880	-18.22640
H	29.94510	-13.84220	-16.98600
C	32.53580	-14.21220	-19.47450
H	32.55450	-13.36100	-20.15600
C	31.03110	-14.52310	-19.18560
H	30.55250	-13.56990	-18.96310
C	33.25930	-15.36540	-20.20870
H	34.28610	-15.08000	-20.44350
H	33.31250	-16.23660	-19.56180
C	30.30290	-15.10070	-20.43260
H	29.33270	-15.50480	-20.13740

C	32.52850	-15.75270	-21.49790
H	32.51350	-14.89800	-22.17640
C	31.09490	-16.20690	-21.16850
H	31.12980	-17.10220	-20.54700
C	29.59580	-15.53100	-15.75690
H	28.71360	-14.97470	-15.43850
H	29.23920	-16.51700	-16.06080
C	35.00360	-14.20790	-16.49700
H	35.97310	-14.70340	-16.55620
H	35.20020	-13.14110	-16.37570
C	30.57380	-15.66180	-14.57470
H	31.45870	-16.21860	-14.88600
H	30.91600	-14.67260	-14.26720
C	29.90340	-16.37340	-13.38510
H	29.00830	-15.81930	-13.09950
H	29.56010	-17.35640	-13.71000
C	30.79880	-16.54270	-12.14620
H	31.12280	-15.56390	-11.79160
H	31.70070	-17.09120	-12.41880
C	30.06800	-17.28610	-11.01380
H	29.16540	-16.73540	-10.74730
H	29.73240	-18.25660	-11.38080
C	30.92070	-17.49670	-9.75220
H	31.23250	-16.52980	-9.35800
H	31.83290	-18.03290	-10.01340
C	30.16560	-18.27710	-8.66310
H	29.25100	-17.74270	-8.40500
H	29.85030	-19.24130	-9.06300
C	30.99580	-18.50680	-7.39040
H	31.29420	-17.54520	-6.97380
H	31.91700	-19.03010	-7.64490
C	30.23190	-19.31150	-6.32690
H	29.30610	-18.79240	-6.07740
H	29.93800	-20.27530	-6.74360
C	31.04790	-19.54100	-5.04560
H	31.33340	-18.57890	-4.62130
H	31.97670	-20.05490	-5.29110
C	30.27930	-20.35550	-3.99420
H	29.34450	-19.84700	-3.75640
H	30.00280	-21.32280	-4.41490
C	31.08300	-20.57330	-2.70360
H	31.35910	-19.60700	-2.28230
H	32.01720	-21.08340	-2.93650
C	30.30790	-21.38450	-1.65530
H	29.36640	-20.88100	-1.43320
H	30.04430	-22.35770	-2.07060
C	31.09840	-21.58280	-0.35390
H	31.36870	-20.61080	0.05790
H	32.03630	-22.09320	-0.57110
C	30.31410	-22.38260	0.69620
H	29.36780	-21.88340	0.90700
H	30.06060	-23.36370	0.29370
C	31.08930	-22.56030	2.00620
H	31.30660	-21.59900	2.47110
H	30.51540	-23.15160	2.71980
H	32.03670	-23.07160	1.83900
C	34.20600	-14.75470	-15.30440
H	33.97950	-15.80800	-15.47960
H	33.24660	-14.23950	-15.23820
C	34.95150	-14.61090	-13.97250
H	35.92100	-15.10430	-14.05030
H	35.15170	-13.55780	-13.77050
C	34.16020	-15.22050	-12.80950
H	33.88800	-16.24480	-13.06650
H	33.22500	-14.67450	-12.67950
C	34.94320	-15.22700	-11.49060
H	35.90090	-15.72510	-11.64470
H	35.17020	-14.20400	-11.18830

C	34.17460	-15.94220	-10.37270
H	33.89380	-16.93830	-10.71640
H	33.24430	-15.40930	-10.17350
C	34.98380	-16.07110	-9.07570
H	35.92800	-16.57380	-9.28690
H	35.23990	-15.07970	-8.70190
C	34.22270	-16.85240	-7.99670
H	33.93940	-17.82810	-8.39250
H	33.29320	-16.33300	-7.76230
C	35.03920	-17.04860	-6.71260
H	35.97590	-17.55280	-6.94980
H	35.31160	-16.07670	-6.30180
C	34.27720	-17.86080	-5.65700
H	33.99610	-18.82680	-6.07690
H	33.34630	-17.34960	-5.41130
C	35.09270	-18.08360	-4.37640
H	36.02500	-18.59220	-4.62070
H	35.37410	-17.11960	-3.95360
C	34.32560	-18.90070	-3.32820
H	34.05050	-19.86680	-3.75160
H	33.39150	-18.39270	-3.08780
C	35.13360	-19.12010	-2.04210
H	36.06550	-19.63270	-2.27950
H	35.41640	-18.15500	-1.62280
C	34.35930	-19.92800	-0.99200
H	34.08950	-20.89820	-1.40930
H	33.42230	-19.42030	-0.76250
C	35.15810	-20.13400	0.30220
H	36.09770	-20.63730	0.07680
H	35.42650	-19.16750	0.72840
C	34.38720	-20.94840	1.34580
H	34.12550	-21.93340	0.96070
H	34.98440	-21.09250	2.24620
H	33.46400	-20.44620	1.63500
O	30.03300	-14.06170	-21.35310
H	29.72050	-14.48220	-22.14460
O	30.42160	-16.54160	-22.36700
H	31.00270	-17.09480	-22.87020
O	33.23470	-16.80000	-22.13270
H	33.05810	-17.61480	-21.67830
C	35.42250	-18.44440	-18.37300
C	32.83630	-19.96280	-17.78160
O	34.97730	-17.33860	-18.07430
O	33.17000	-21.10000	-17.44970
N	36.48570	-18.97260	-17.75170
N	32.13900	-19.14710	-16.97800
H	36.80660	-19.88030	-18.05490
H	31.91730	-18.21840	-17.31990
C	34.71520	-19.28240	-19.45830
H	34.82320	-18.69450	-20.37100
C	33.18280	-19.41830	-19.18430
H	32.75170	-18.41830	-19.23000
C	35.39160	-20.65190	-19.72970
H	36.43880	-20.50460	-19.99900
H	35.38900	-21.26080	-18.82460
C	32.48350	-20.25420	-20.28710
H	31.45190	-20.45830	-19.99400
C	34.69370	-21.41590	-20.86610
H	34.83490	-20.87380	-21.80290
C	33.19030	-21.59810	-20.58230
H	33.06000	-22.27210	-19.73500
C	31.53110	-19.53720	-15.71500
H	30.72500	-18.83570	-15.49800
H	31.06200	-20.51580	-15.83330
C	37.11880	-18.40920	-16.56730
H	38.11010	-18.85350	-16.47490
H	37.26400	-17.33310	-16.67880
C	32.53060	-19.55990	-14.54760

H	33.39550	-20.17130	-14.80820
H	32.90480	-18.55350	-14.35450
C	31.86850	-20.13240	-13.28820
H	31.00830	-19.51950	-13.01910
H	31.47300	-21.12130	-13.52430
C	32.80820	-20.25340	-12.08210
H	33.13510	-19.26290	-11.76400
H	33.70470	-20.80370	-12.36930
C	32.11680	-20.98230	-10.92250
H	31.20940	-20.44480	-10.64740
H	31.79380	-21.96630	-11.26510
C	33.00290	-21.15390	-9.68320
H	33.29090	-20.17520	-9.29880
H	33.92510	-21.66560	-9.96000
C	32.28490	-21.95590	-8.59020
H	31.35000	-21.45810	-8.33240
H	32.01000	-22.93440	-8.98590
C	33.12750	-22.14380	-7.32320
H	33.38800	-21.16900	-6.91060
H	34.06690	-22.63520	-7.57830
C	32.38820	-22.97380	-6.26570
H	31.43780	-22.49430	-6.02990
H	32.14260	-23.95190	-6.68140
C	33.19850	-23.15920	-4.97720
H	33.44200	-22.18260	-4.55830
H	34.14800	-23.64230	-5.20980
C	32.44280	-23.99320	-3.93430
H	31.48400	-23.52070	-3.71960
H	32.21460	-24.97480	-4.35150
C	33.23020	-24.16470	-2.62920
H	33.46250	-23.18310	-2.21600
H	34.18600	-24.64500	-2.84100
C	32.46260	-24.99090	-1.58830
H	31.49880	-24.52010	-1.39230
H	32.24480	-25.97830	-1.99710
C	33.23400	-25.14220	-0.27050
H	33.46170	-24.15440	0.12980
H	34.19260	-25.62470	-0.46440
C	32.45710	-25.95160	0.77700
H	31.49230	-25.47890	0.96440
H	32.24110	-26.94600	0.38490
C	33.21770	-26.08500	2.10080
H	33.42080	-25.10800	2.54000
H	32.63990	-26.66030	2.82430
H	34.17150	-26.59350	1.95780
C	36.27860	-18.72150	-15.31940
H	36.03060	-19.78420	-15.32140
H	35.32820	-18.18740	-15.36620
C	36.99270	-18.38050	-14.00680
H	37.97510	-18.85430	-14.00590
H	37.16440	-17.30600	-13.93480
C	36.19400	-18.87240	-12.79370
H	35.89420	-19.90590	-12.97070
H	35.27150	-18.29870	-12.69780
C	36.98870	-18.81580	-11.48370
H	37.95350	-19.30370	-11.62820
H	37.20140	-17.78050	-11.21570
C	36.24220	-19.51740	-10.34340
H	35.96260	-20.52000	-10.66880
H	35.31020	-18.99120	-10.13660
C	37.07470	-19.63150	-9.06120
H	38.02720	-20.11150	-9.28910
H	37.31200	-18.63620	-8.68450
C	36.34710	-20.44330	-7.98270
H	36.09180	-21.42460	-8.38430
H	35.40280	-19.95650	-7.73890
C	37.17910	-20.62440	-6.70740
H	38.12750	-21.10230	-6.95620

H	37.42570	-19.64730	-6.29110
C	36.44430	-21.46690	-5.65670
H	36.20550	-22.44360	-6.07910
H	35.49150	-20.99420	-5.41800
C	37.25770	-21.65530	-4.36990
H	38.20780	-22.13610	-4.60600
H	37.50220	-20.67910	-3.95000
C	36.50660	-22.49180	-3.32510
H	36.28140	-23.47470	-3.74040
H	35.54690	-22.02170	-3.10960
C	37.29610	-22.65740	-2.01970
H	38.25100	-23.14080	-2.22980
H	37.53230	-21.67340	-1.61350
C	36.52850	-23.47410	-0.97070
H	36.31870	-24.46820	-1.36720
H	35.56110	-23.00680	-0.78610
C	37.29280	-23.60190	0.35430
H	38.25610	-24.08120	0.17630
H	37.51090	-22.60930	0.74990
C	36.51700	-24.40170	1.40610
H	36.31040	-25.41390	1.05790
H	37.08500	-24.47880	2.33330
H	35.56460	-23.92580	1.63980
O	32.41710	-19.49670	-21.48110
H	32.12880	-20.09250	-22.16120
O	32.57760	-22.20220	-21.70520
H	33.12370	-22.93000	-21.96730
O	35.29710	-22.68960	-20.98690
H	35.18160	-23.15730	-20.17310
C	31.12190	-4.13730	-16.67240
C	28.30390	-5.31930	-16.37130
O	31.10320	-3.16230	-15.92570
O	28.33810	-6.50670	-16.69250
N	31.94770	-5.17200	-16.46970
N	27.92100	-4.91350	-15.15130
H	31.87360	-5.96830	-17.08880
H	27.94380	-3.91840	-14.95860
C	30.14000	-4.19750	-17.85720
H	30.29540	-3.25170	-18.37870
C	28.65040	-4.21130	-17.39290
H	28.46940	-3.26650	-16.88280
C	30.45580	-5.31160	-18.88240
H	31.49330	-5.24030	-19.21290
H	30.33980	-6.28780	-18.41700
C	27.68250	-4.25060	-18.60610
H	26.66470	-4.43680	-18.25820
C	29.52440	-5.21930	-20.09820
H	29.70140	-4.27490	-20.61580
C	28.05090	-5.32210	-19.65810
H	27.86460	-6.31020	-19.23800
C	27.31720	-5.77020	-14.13770
H	26.63560	-5.15490	-13.54960
H	26.70030	-6.53240	-14.61740
C	32.81090	-5.32820	-15.30800
H	33.62080	-6.00770	-15.57430
H	33.27210	-4.37600	-15.03980
C	28.34970	-6.42960	-13.20810
H	29.08030	-6.98840	-13.79490
H	28.90170	-5.65670	-12.67140
C	27.66190	-7.37420	-12.20880
H	26.86390	-6.83130	-11.70040
H	27.17840	-8.18320	-12.75820
C	28.60160	-7.97110	-11.15080
H	29.06350	-7.16080	-10.58590
H	29.41040	-8.51950	-11.63490
C	27.84860	-8.90410	-10.18900
H	26.97600	-8.37980	-9.79730
H	27.46680	-9.76360	-10.74140

C	28.70070	-9.39050	-9.00810
H	29.09770	-8.52620	-8.47530
H	29.55970	-9.95350	-9.37480
C	27.88970	-10.25710	-8.03290
H	26.99120	-9.71500	-7.73540
H	27.54840	-11.15840	-8.54300
C	28.67470	-10.64570	-6.77260
H	29.03340	-9.74160	-6.28060
H	29.55930	-11.21860	-7.05170
C	27.82300	-11.45490	-5.78370
H	26.92370	-10.88970	-5.53610
H	27.48430	-12.37460	-6.26130
C	28.57400	-11.79670	-4.49000
H	28.91510	-10.87570	-4.01750
H	29.46940	-12.37030	-4.72900
C	27.70630	-12.58560	-3.49900
H	26.80680	-12.01300	-3.26960
H	27.36910	-13.51110	-3.96610
C	28.44610	-12.91230	-2.19500
H	28.77820	-11.98610	-1.72670
H	29.34740	-13.48130	-2.42170
C	27.57730	-13.70200	-1.20590
H	26.67250	-13.13480	-0.98440
H	27.24980	-14.63220	-1.67110
C	28.31340	-14.01790	0.10310
H	28.63370	-13.08770	0.57130
H	29.22270	-14.57650	-0.11740
C	27.44940	-14.81720	1.08850
H	26.53130	-14.26850	1.30100
H	27.14440	-15.75760	0.62860
C	28.17590	-15.11240	2.40500
H	28.43850	-14.19280	2.92640
H	27.54870	-15.70450	3.07150
H	29.09600	-15.66940	2.23250
C	32.01070	-5.89810	-14.12910
H	31.55660	-6.84310	-14.42960
H	31.18580	-5.22690	-13.88510
C	32.86000	-6.12530	-12.87560
H	33.72350	-6.74290	-13.12550
H	33.24810	-5.17180	-12.51520
C	32.04370	-6.80900	-11.77470
H	31.65810	-7.75500	-12.15740
H	31.17520	-6.19560	-11.53210
C	32.85110	-7.07440	-10.50080
H	33.74390	-7.65020	-10.74760
H	33.19480	-6.12940	-10.07870
C	32.02040	-7.83670	-9.46360
H	31.66910	-8.77060	-9.90440
H	31.13080	-7.25720	-9.21480
C	32.80010	-8.14530	-8.18220
H	33.70290	-8.70420	-8.43030
H	33.12800	-7.21390	-7.71970
C	31.95600	-8.94800	-7.18640
H	31.62600	-9.87440	-7.65790
H	31.05390	-8.38650	-6.94290
C	32.71450	-9.27640	-5.89670
H	33.62200	-9.82950	-6.13940
H	33.03570	-8.35160	-5.41730
C	31.86030	-10.09220	-4.91950
H	31.53930	-11.01580	-5.40160
H	30.95300	-9.53670	-4.68260
C	32.60700	-10.42640	-3.62380
H	33.51670	-10.97750	-3.86140
H	32.92590	-9.50340	-3.13990
C	31.74880	-11.24510	-2.65200
H	31.43170	-12.16900	-3.13590
H	30.83920	-10.69200	-2.41870
C	32.49120	-11.57820	-1.35300

H	33.40280	-12.12720	-1.58740
H	32.80870	-10.65480	-0.86930
C	31.63350	-12.39820	-0.38120
H	31.32000	-13.32360	-0.86440
H	30.72160	-11.84800	-0.15000
C	32.37490	-12.72740	0.92010
H	33.29000	-13.27210	0.69170
H	32.68750	-11.80470	1.40720
C	31.52180	-13.55110	1.88950
H	31.22280	-14.49870	1.44300
H	32.07360	-13.77290	2.80300
H	30.61540	-13.01490	2.16890
O	27.66200	-2.98650	-19.24170
H	27.19170	-3.10680	-20.05720
O	27.21360	-5.16820	-20.78830
H	27.54970	-5.73890	-21.46490
O	29.82680	-6.27350	-20.98980
H	29.46490	-7.07840	-20.63870
C	31.64440	-8.98450	-17.52210
C	29.07390	-10.61360	-17.35480
O	31.32000	-8.04630	-16.79910
O	29.29850	-11.80330	-17.57790
N	32.68600	-9.77730	-17.23700
N	28.58870	-10.17340	-16.18380
H	32.86310	-10.56770	-17.84340
H	28.46950	-9.17460	-16.07510
C	30.80730	-9.28300	-18.78070
H	30.84480	-8.35530	-19.35120
C	29.30540	-9.54230	-18.44480
H	28.90790	-8.61430	-18.03380
C	31.41110	-10.36560	-19.70400
H	32.44630	-10.12280	-19.94940
H	31.42250	-11.32640	-19.19490
C	28.47490	-9.84330	-19.72240
H	27.48850	-10.21560	-19.44000
C	30.59650	-10.48950	-20.99700
H	30.63170	-9.54260	-21.53860
C	29.13900	-10.86850	-20.66990
H	29.11480	-11.85130	-20.19980
C	28.11090	-11.02020	-15.09730
H	27.28070	-10.49940	-14.61900
H	27.69770	-11.94850	-15.49620
C	33.50880	-9.68180	-16.03890
H	34.45710	-10.17990	-16.24230
H	33.74090	-8.63850	-15.81820
C	29.19750	-11.32680	-14.05310
H	30.03760	-11.83740	-14.52630
H	29.58630	-10.39270	-13.64480
C	28.63460	-12.19560	-12.91690
H	27.74890	-11.70790	-12.50720
H	28.29550	-13.14730	-13.32850
C	29.62300	-12.46300	-11.77200
H	29.97160	-11.51380	-11.36370
H	30.50130	-12.98430	-12.15390
C	28.97280	-13.29260	-10.65420
H	28.06510	-12.78850	-10.32050
H	28.65540	-14.25450	-11.05820
C	29.88590	-13.52700	-9.44210
H	30.21350	-12.56690	-9.04280
H	30.78430	-14.05940	-9.75520
C	29.17300	-14.32170	-8.33840
H	28.26320	-13.79480	-8.04980
H	28.85260	-15.28440	-8.73720
C	30.03970	-14.55150	-7.09210
H	30.35840	-13.59070	-6.68790
H	30.94710	-15.08770	-7.37010
C	29.29050	-15.33850	-6.00770
H	28.37850	-14.80400	-5.74180

H	28.97230	-16.29900	-6.41320
C	30.12820	-15.57410	-4.74310
H	30.43950	-14.61530	-4.32860
H	31.04150	-16.10900	-5.00360
C	29.35810	-16.36630	-3.67770
H	28.43990	-15.83490	-3.42720
H	29.05050	-17.32640	-4.09210
C	30.17480	-16.60220	-2.39990
H	30.47500	-15.64290	-1.97810
H	31.09520	-17.13130	-2.64710
C	29.39320	-17.40130	-1.34820
H	28.46650	-16.87840	-1.11190
H	29.10090	-18.36400	-1.76740
C	30.19450	-17.62820	-0.05920
H	30.48240	-16.66580	0.36370
H	31.12230	-18.14960	-0.29530
C	29.40940	-18.43150	0.98670
H	28.47700	-17.91780	1.22200
H	29.12680	-19.39760	0.56900
C	30.20430	-18.65250	2.27790
H	30.45380	-17.70510	2.75580
H	29.63000	-19.24410	2.99100
H	31.13610	-19.18240	2.08170
C	32.81220	-10.34840	-14.84350
H	32.55120	-11.37500	-15.10630
H	31.87260	-9.83460	-14.63600
C	33.67590	-10.35330	-13.57620
H	34.61880	-10.86090	-13.78360
H	33.92600	-9.32920	-13.29620
C	32.96600	-11.04870	-12.40880
H	32.67360	-12.05280	-12.71890
H	32.04420	-10.51350	-12.17720
C	33.83300	-11.14610	-11.14700
H	34.76280	-11.66370	-11.38600
H	34.11000	-10.14640	-10.81050
C	33.10990	-11.88990	-10.01750
H	32.79760	-12.86950	-10.38090
H	32.19860	-11.35060	-9.75670
C	33.97470	-12.07210	-8.76320
H	34.89170	-12.59950	-9.02840
H	34.27730	-11.09720	-8.38030
C	33.23610	-12.85020	-7.66630
H	32.91840	-13.81410	-8.06470
H	32.32650	-12.31380	-7.39510
C	34.08900	-13.07840	-6.41050
H	35.00200	-13.60900	-6.68210
H	34.40200	-12.11790	-6.00100
C	33.33540	-13.87350	-5.33570
H	33.01990	-14.83040	-5.75160
H	32.42330	-13.34050	-5.06710
C	34.17340	-14.11990	-4.07310
H	35.08860	-14.64890	-4.33970
H	34.48500	-13.16500	-3.64970
C	33.40790	-14.92420	-3.01340
H	33.10080	-15.88010	-3.43700
H	32.49040	-14.39660	-2.75350
C	34.23160	-15.17170	-1.74170
H	35.14990	-15.70000	-1.99830
H	34.53910	-14.21690	-1.31510
C	33.45600	-15.97530	-0.68870
H	33.15810	-16.93440	-1.11180
H	32.53240	-15.45230	-0.44230
C	34.26730	-16.21220	0.59280
H	35.18570	-16.74700	0.35090
H	34.57530	-15.25700	1.01820
C	33.48430	-17.00190	1.64660
H	33.17190	-17.97120	1.25980
H	34.09340	-17.17900	2.53300

H	32.58980	-16.46440	1.96000
O	28.25650	-8.64500	-20.44240
H	27.86060	-8.89890	-21.26680
O	28.39250	-10.94350	-21.86960
H	28.89710	-11.46140	-22.48100
O	31.18070	-11.48100	-21.81680
H	30.93640	-12.33540	-21.48110

5-beta monomer

C	30.83570000	-8.61190000	-17.07750000
C	28.11460000	-9.84570000	-17.29470000
O	30.52880000	-7.68270000	-16.33390000
O	28.30840000	-11.05350000	-17.45250000
N	31.74500000	-9.52590000	-16.72180000
N	27.48820000	-9.33490000	-16.22680000
H	31.92240000	-10.31130000	-17.34460000
H	27.39170000	-8.32130000	-16.18040000
C	30.15170000	-8.73430000	-18.45600000
H	30.37380000	-7.78700000	-18.94390000
C	28.59520000	-8.83410000	-18.34830000
H	28.23930000	-7.86690000	-18.00230000
C	30.76800000	-9.82730000	-19.35870000
H	31.83920000	-9.65660000	-19.47890000
H	30.65360000	-10.79750000	-18.88750000
C	27.89730000	-9.09040000	-19.70960000
H	26.86670000	-9.41030000	-19.54040000
C	30.09710000	-9.83540000	-20.73420000
H	30.23730000	-8.85220000	-21.18690000
C	28.59260000	-10.13840000	-20.60010000
H	28.43400000	-11.13620000	-20.19120000
C	26.93980000	-10.10350000	-15.12120000
H	26.15700000	-9.50700000	-14.65120000
H	26.46120000	-11.00950000	-15.49790000
C	32.53520000	-9.50980000	-15.50020000
H	33.50410000	-9.94430000	-15.74860000
H	32.72970000	-8.48720000	-15.17020000
C	28.02430000	-10.45240000	-14.09230000
H	28.80830000	-11.03840000	-14.57470000
H	28.49740000	-9.53600000	-13.73520000
C	27.45870000	-11.23850000	-12.90330000
H	26.68790000	-10.64490000	-12.41000000
H	26.96890000	-12.14260000	-13.26740000
C	28.54020000	-11.62040000	-11.88630000
H	29.02950000	-10.71690000	-11.52050000
H	29.30990000	-12.21110000	-12.38420000
C	27.97510000	-12.41420000	-10.70140000
H	27.21440000	-11.81980000	-10.19450000
H	27.47230000	-13.30980000	-11.06830000
C	29.06380000	-12.81700000	-9.69990000
H	29.56610000	-11.92240000	-9.33130000
H	29.82310000	-13.40770000	-10.21200000
C	28.51060000	-13.61960000	-8.51510000
H	27.76340000	-13.02480000	-7.98960000
H	27.99300000	-14.50620000	-8.88240000
C	29.61360000	-14.04270000	-7.53730000
H	30.12880000	-13.15650000	-7.16740000
H	30.36060000	-14.63210000	-8.06850000
C	29.07660000	-14.85500000	-6.35190000
H	28.33790000	-14.26340000	-5.81090000
H	28.55250000	-15.73780000	-6.71940000
C	30.19270000	-15.28720000	-5.39260000
H	30.71780000	-14.40460000	-5.02820000
H	30.92900000	-15.87880000	-5.93630000
C	29.66800000	-16.09840000	-4.20110000
H	28.93470000	-15.50540000	-3.65400000

H	29.14010000	-16.98130000	-4.56310000
C	30.79180000	-16.52960000	-3.24990000
H	31.32330000	-15.64700000	-2.89500000
H	31.52100000	-17.12740000	-3.79660000
C	30.27260000	-17.33110000	-2.04900000
H	29.54150000	-16.73320000	-1.50430000
H	29.74290000	-18.21660000	-2.40200000
C	31.39860000	-17.75520000	-1.09700000
H	31.93390000	-16.87060000	-0.75260000
H	32.12430000	-18.36010000	-1.64070000
C	30.88010000	-18.54400000	0.11310000
H	30.15500000	-17.94170000	0.66180000
H	30.34620000	-19.43150000	-0.22820000
C	32.00480000	-18.96750000	1.06320000
H	32.52610000	-18.10140000	1.47000000
H	31.61060000	-19.54140000	1.90210000
H	32.73840000	-19.59070000	0.55250000
C	31.86770000	-10.32350000	-14.38050000
H	31.48920000	-11.26130000	-14.78760000
H	31.00200000	-9.78000000	-13.99980000
C	32.84970000	-10.62110000	-13.23850000
H	33.72520000	-11.12300000	-13.65320000
H	33.20790000	-9.68310000	-12.81160000
C	32.25630000	-11.49780000	-12.12700000
H	31.80310000	-12.38850000	-12.56340000
H	31.45650000	-10.95570000	-11.62240000
C	33.32410000	-11.91710000	-11.10670000
H	34.11800000	-12.46100000	-11.62060000
H	33.78850000	-11.02590000	-10.68260000
C	32.77020000	-12.78960000	-9.97290000
H	32.28310000	-13.66970000	-10.39360000
H	32.00210000	-12.23790000	-9.43110000
C	33.87020000	-13.22720000	-8.99600000
H	34.63350000	-13.78770000	-9.53720000
H	34.36750000	-12.34440000	-8.59250000
C	33.33670000	-14.08170000	-7.83910000
H	32.83480000	-14.96270000	-8.23940000
H	32.58260000	-13.51940000	-7.28840000
C	34.45100000	-14.51460000	-6.87680000
H	35.20590000	-15.07900000	-7.42520000
H	34.95540000	-13.63050000	-6.48530000
C	33.92930000	-15.36120000	-5.70880000
H	33.41900000	-16.24190000	-6.09900000
H	33.18330000	-14.79410000	-5.15200000
C	35.05200000	-15.79520000	-4.75720000
H	35.80040000	-16.36180000	-5.31230000
H	35.56220000	-14.91230000	-4.37090000
C	34.53740000	-16.64050000	-3.58520000
H	34.02030000	-17.51860000	-3.97200000
H	33.79790000	-16.07100000	-3.02210000
C	35.66550000	-17.08090000	-2.64290000
H	36.40820000	-17.64800000	-3.20510000
H	36.18110000	-16.20110000	-2.25670000
C	35.15440000	-17.92940000	-1.47170000
H	34.63080000	-18.80260000	-1.86120000
H	34.41960000	-17.36010000	-0.90230000
C	36.28490000	-18.38170000	-0.53760000
H	37.02640000	-18.94450000	-1.10560000
H	36.80340000	-17.51030000	-0.13610000
C	35.77660000	-19.24510000	0.62090000
H	35.24790000	-20.12350000	0.25230000
H	36.60340000	-19.59060000	1.24200000
H	35.09260000	-18.68700000	1.26010000
O	27.82180000	-7.91590000	-20.49480000
H	27.60830000	-8.26650000	-21.36240000
O	27.98680000	-10.05690000	-21.87870000
H	28.59230000	-10.42270000	-22.52780000
O	30.71700000	-10.78890000	-21.57490000

H	30.24660000	-11.62800000	-21.50560000

6-beta -dimer

C	27.79360000	-6.59860000	-27.14340000
C	30.78140000	-6.48870000	-27.32410000
O	27.56790000	-5.39790000	-27.27810000
O	31.11670000	-7.56960000	-27.81330000
N	27.31530000	-7.50450000	-28.00360000
N	30.99190000	-5.31100000	-27.92640000
H	27.57810000	-8.48100000	-27.88710000
H	30.66450000	-4.47340000	-27.44630000
C	28.65670000	-7.07610000	-25.95580000
H	28.13940000	-6.68410000	-25.08130000
C	30.08420000	-6.43780000	-25.95540000
H	29.96000000	-5.38030000	-25.73580000
C	28.67670000	-8.61310000	-25.78040000
H	27.65800000	-8.99330000	-25.68620000
H	29.10710000	-9.07950000	-26.66010000
C	31.01260000	-7.00270000	-24.84880000
H	32.04840000	-6.72780000	-25.06060000
C	29.48110000	-8.99960000	-24.53670000
H	29.02080000	-8.50890000	-23.67790000
C	30.94140000	-8.53260000	-24.66910000
H	31.43240000	-9.03550000	-25.50290000
C	31.64350000	-5.12300000	-29.21250000
H	32.04630000	-4.10990000	-29.24140000
H	32.49350000	-5.80200000	-29.30330000
C	26.41900000	-7.23040000	-29.11690000
H	25.76820000	-8.10000000	-29.21550000
H	25.76890000	-6.38050000	-28.89920000
C	30.65890000	-5.33050000	-30.37180000
H	30.25610000	-6.34370000	-30.32890000
H	29.81020000	-4.65420000	-30.25590000
C	31.31810000	-5.10100000	-31.73740000
H	31.70060000	-4.08070000	-31.78890000
H	32.18040000	-5.76130000	-31.83860000
C	30.35100000	-5.34520000	-32.90150000
H	29.48940000	-4.68380000	-32.80220000
H	29.96850000	-6.36470000	-32.84280000
C	31.01090000	-5.12680000	-34.26920000
H	31.37960000	-4.10290000	-34.33700000
H	31.88310000	-5.77500000	-34.36190000
C	30.04670000	-5.40340000	-35.42880000
H	29.17550000	-4.75450000	-35.33820000
H	29.67790000	-6.42620000	-35.35340000
C	30.69690000	-5.19940000	-36.80360000
H	31.05020000	-4.17190000	-36.89080000
H	31.57870000	-5.83480000	-36.89050000
C	29.72960000	-5.51110000	-37.95210000
H	28.84910000	-4.87470000	-37.86610000
H	29.37610000	-6.53760000	-37.85770000
C	30.36620000	-5.31970000	-39.33480000
H	30.70930000	-4.29010000	-39.43770000
H	31.25300000	-5.94840000	-39.42080000
C	29.39200000	-5.65350000	-40.47160000
H	28.50430000	-5.02770000	-40.38210000
H	29.05220000	-6.68370000	-40.36670000
C	30.01410000	-5.46220000	-41.86090000
H	30.34950000	-4.43050000	-41.96870000
H	30.90420000	-6.08540000	-41.95270000
C	29.03300000	-5.80340000	-42.98990000
H	28.14020000	-5.18620000	-42.89160000
H	28.70410000	-6.83720000	-42.88500000
C	29.64320000	-5.60110000	-44.38320000
H	29.97300000	-4.56710000	-44.48680000

H	30.53570000	-6.21930000	-44.48470000
C	28.65700000	-5.93910000	-45.50890000
H	27.76050000	-5.32910000	-45.40020000
H	28.33590000	-6.97600000	-45.40990000
C	29.25880000	-5.72160000	-46.90390000
H	29.57880000	-4.68440000	-47.00930000
H	30.15560000	-6.33190000	-47.01640000
C	28.27430000	-6.06150000	-48.02740000
H	27.38740000	-5.43030000	-47.98070000
H	28.73380000	-5.91610000	-49.00520000
H	27.94980000	-7.09990000	-47.96620000
C	27.18920000	-7.00210000	-30.42730000
H	27.97680000	-7.74980000	-30.52180000
H	27.68620000	-6.03170000	-30.39520000
C	26.25960000	-7.08010000	-31.64730000
H	25.73800000	-8.03810000	-31.62730000
H	25.48990000	-6.31080000	-31.56890000
C	26.98840000	-6.94170000	-32.99160000
H	27.82490000	-7.64030000	-33.02970000
H	27.41300000	-5.94130000	-33.07600000
C	26.04720000	-7.20830000	-34.17520000
H	25.62660000	-8.21050000	-34.08070000
H	25.20430000	-6.51750000	-34.12930000
C	26.73500000	-7.08000000	-35.54080000
H	27.59370000	-7.75070000	-35.58180000
H	27.12400000	-6.06890000	-35.65950000
C	25.77750000	-7.39830000	-36.69770000
H	25.39440000	-8.41290000	-36.58190000
H	24.91200000	-6.73700000	-36.64290000
C	26.43580000	-7.25840000	-38.07650000
H	27.30530000	-7.91360000	-38.13240000
H	26.80650000	-6.24110000	-38.20200000
C	25.46530000	-7.59000000	-39.21830000
H	25.09450000	-8.60820000	-39.09560000
H	24.59340000	-6.93820000	-39.15360000
C	26.10690000	-7.44430000	-40.60430000
H	26.98360000	-8.08890000	-40.66780000
H	26.46500000	-6.42300000	-40.73490000
C	25.13060000	-7.78910000	-41.73680000
H	24.77100000	-8.81050000	-41.60790000
H	24.25280000	-7.14570000	-41.66810000
C	25.76280000	-7.64180000	-43.12680000
H	26.64620000	-8.27730000	-43.19200000
H	26.10980000	-6.61750000	-43.26290000
C	24.78570000	-8.00180000	-44.25380000
H	24.43600000	-9.02580000	-44.11790000
H	23.90260000	-7.36580000	-44.18650000
C	25.41470000	-7.85740000	-45.64550000
H	26.30440000	-8.48450000	-45.70570000
H	25.75260000	-6.83100000	-45.78830000
C	24.44200000	-8.23580000	-46.77060000
H	24.09620000	-9.25990000	-46.62670000
H	23.55510000	-7.60300000	-46.72060000
C	25.07530000	-8.10860000	-48.15950000
H	25.96950000	-8.72570000	-48.24050000
H	24.37960000	-8.42740000	-48.93610000
H	25.35980000	-7.07820000	-48.37200000
O	30.71020000	-6.45420000	-23.57930000
H	31.17090000	-7.05270000	-22.98700000
O	31.62820000	-8.83710000	-23.46790000
H	31.28770000	-9.66700000	-23.12860000
O	29.42720000	-10.39660000	-24.31110000
H	30.13470000	-10.83430000	-24.79710000
C	30.83570000	-8.61190000	-17.07750000
C	28.11460000	-9.84570000	-17.29470000
O	30.52880000	-7.68270000	-16.33390000
O	28.30840000	-11.05350000	-17.45250000
N	31.74500000	-9.52590000	-16.72180000

N	27.48820000	-9.33490000	-16.22680000
H	31.92240000	-10.31130000	-17.34460000
H	27.39170000	-8.32130000	-16.18040000
C	30.15170000	-8.73430000	-18.45600000
H	30.37380000	-7.78700000	-18.94390000
C	28.59520000	-8.83410000	-18.34830000
H	28.23930000	-7.86690000	-18.00230000
C	30.76800000	-9.82730000	-19.35870000
H	31.83920000	-9.65660000	-19.47890000
H	30.65360000	-10.79750000	-18.88750000
C	27.89730000	-9.09040000	-19.70960000
H	26.86670000	-9.41030000	-19.54040000
C	30.09710000	-9.83540000	-20.73420000
H	30.23730000	-8.85220000	-21.18690000
C	28.59260000	-10.13840000	-20.60010000
H	28.43400000	-11.13620000	-20.19120000
C	26.93980000	-10.10350000	-15.12120000
H	26.15700000	-9.50700000	-14.65120000
H	26.46120000	-11.00950000	-15.49790000
C	32.53520000	-9.50980000	-15.50020000
H	33.50410000	-9.94430000	-15.74860000
H	32.72970000	-8.48720000	-15.17020000
C	28.02430000	-10.45240000	-14.09230000
H	28.80830000	-11.03840000	-14.57470000
H	28.49740000	-9.53600000	-13.73520000
C	27.45870000	-11.23850000	-12.90330000
H	26.68790000	-10.64490000	-12.41000000
H	26.96890000	-12.14260000	-13.26740000
C	28.54020000	-11.62040000	-11.88630000
H	29.02950000	-10.71690000	-11.52050000
H	29.30990000	-12.21110000	-12.38420000
C	27.97510000	-12.41420000	-10.70140000
H	27.21440000	-11.81980000	-10.19450000
H	27.47230000	-13.30980000	-11.06830000
C	29.06380000	-12.81700000	-9.69990000
H	29.56610000	-11.92240000	-9.33130000
H	29.82310000	-13.40770000	-10.21200000
C	28.51060000	-13.61960000	-8.51510000
H	27.76340000	-13.02480000	-7.98960000
H	27.99300000	-14.50620000	-8.88240000
C	29.61360000	-14.04270000	-7.53730000
H	30.12880000	-13.15650000	-7.16740000
H	30.36060000	-14.63210000	-8.06850000
C	29.07660000	-14.85500000	-6.35190000
H	28.33790000	-14.26340000	-5.81090000
H	28.55250000	-15.73780000	-6.71940000
C	30.19270000	-15.28720000	-5.39260000
H	30.71780000	-14.40460000	-5.02820000
H	30.92900000	-15.87880000	-5.93630000
C	29.66800000	-16.09840000	-4.20110000
H	28.93470000	-15.50540000	-3.65400000
H	29.14010000	-16.98130000	-4.56310000
C	30.79180000	-16.52960000	-3.24990000
H	31.32330000	-15.64700000	-2.89500000
H	31.52100000	-17.12740000	-3.79660000
C	30.27260000	-17.33110000	-2.04900000
H	29.54150000	-16.73320000	-1.50430000
H	29.74290000	-18.21660000	-2.40200000
C	31.39860000	-17.75520000	-1.09700000
H	31.93390000	-16.87060000	-0.75260000
H	32.12430000	-18.36010000	-1.64070000
C	30.88010000	-18.54400000	0.11310000
H	30.15500000	-17.94170000	0.66180000
H	30.34620000	-19.43150000	-0.22820000
C	32.00480000	-18.96750000	1.06320000
H	32.52610000	-18.10140000	1.47000000
H	31.61060000	-19.54140000	1.90210000
H	32.73840000	-19.59070000	0.55250000

C	31.86770000	-10.32350000	-14.38050000
H	31.48920000	-11.26130000	-14.78760000
H	31.00200000	-9.78000000	-13.99980000
C	32.84970000	-10.62110000	-13.23850000
H	33.72520000	-11.12300000	-13.65320000
H	33.20790000	-9.68310000	-12.81160000
C	32.25630000	-11.49780000	-12.12700000
H	31.80310000	-12.38850000	-12.56340000
H	31.45650000	-10.95570000	-11.62240000
C	33.32410000	-11.91710000	-11.10670000
H	34.11800000	-12.46100000	-11.62060000
H	33.78850000	-11.02590000	-10.68260000
C	32.77020000	-12.78960000	-9.97290000
H	32.28310000	-13.66970000	-10.39360000
H	32.00210000	-12.23790000	-9.43110000
C	33.87020000	-13.22720000	-8.99600000
H	34.63350000	-13.78770000	-9.53720000
H	34.36750000	-12.34440000	-8.59250000
C	33.33670000	-14.08170000	-7.83910000
H	32.83480000	-14.96270000	-8.23940000
H	32.58260000	-13.51940000	-7.28840000
C	34.45100000	-14.51460000	-6.87680000
H	35.20590000	-15.07900000	-7.42520000
H	34.95540000	-13.63050000	-6.48530000
C	33.92930000	-15.36120000	-5.70880000
H	33.41900000	-16.24190000	-6.09900000
H	33.18330000	-14.79410000	-5.15200000
C	35.05200000	-15.79520000	-4.75720000
H	35.80040000	-16.36180000	-5.31230000
H	35.56220000	-14.91230000	-4.37090000
C	34.53740000	-16.64050000	-3.58520000
H	34.02030000	-17.51860000	-3.97200000
H	33.79790000	-16.07100000	-3.02210000
C	35.66550000	-17.08090000	-2.64290000
H	36.40820000	-17.64800000	-3.20510000
H	36.18110000	-16.20110000	-2.25670000
C	35.15440000	-17.92940000	-1.47170000
H	34.63080000	-18.80260000	-1.86120000
H	34.41960000	-17.36010000	-0.90230000
C	36.28490000	-18.38170000	-0.53760000
H	37.02640000	-18.94450000	-1.10560000
H	36.80340000	-17.51030000	-0.13610000
C	35.77660000	-19.24510000	0.62090000
H	35.24790000	-20.12350000	0.25230000
H	36.60340000	-19.59060000	1.24200000
H	35.09260000	-18.68700000	1.26010000
O	27.82180000	-7.91590000	-20.49480000
H	27.60830000	-8.26650000	-21.36240000
O	27.98680000	-10.05690000	-21.87870000
H	28.59230000	-10.42270000	-22.52780000
O	30.71700000	-10.78890000	-21.57490000
H	30.24660000	-11.62800000	-21.50560000

5-beta tetramer

C	26.80080000	-1.78030000	-25.96710000
C	29.85250000	-1.72860000	-25.97880000
O	26.29660000	-0.66250000	-26.02400000
O	30.14590000	-2.86970000	-26.34550000
N	26.65080000	-2.69230000	-26.93790000
N	30.21380000	-0.63550000	-26.66110000
H	27.11480000	-3.59320000	-26.83470000
H	29.96170000	0.26540000	-26.26220000
C	27.61830000	-2.15280000	-24.72190000
H	27.06080000	-1.70930000	-23.89500000
C	29.02710000	-1.49080000	-24.70010000

H	28.86850000	-0.42000000	-24.64570000
C	27.65430000	-3.66760000	-24.44410000
H	26.64140000	-4.07270000	-24.41280000
H	28.17770000	-4.18010000	-25.24170000
C	29.82830000	-1.88430000	-23.43760000
H	30.86450000	-1.55500000	-23.54070000
C	28.36620000	-3.93750000	-23.12290000
H	27.80790000	-3.42870000	-22.33780000
C	29.81100000	-3.39680000	-23.15010000
H	30.40750000	-3.91730000	-23.89890000
C	30.94080000	-0.64110000	-27.92240000
H	31.36770000	0.35060000	-28.07200000
H	31.77940000	-1.33760000	-27.86480000
C	25.79000000	-2.56630000	-28.10670000
H	25.05430000	-3.36910000	-28.04730000
H	25.22720000	-1.63080000	-28.09770000
C	30.01570000	-1.00610000	-29.09420000
H	29.60610000	-2.00550000	-28.93830000
H	29.16430000	-0.32410000	-29.10570000
C	30.72770000	-0.96260000	-30.45230000
H	31.14530000	0.03250000	-30.60850000
H	31.57000000	-1.65560000	-30.44760000
C	29.78260000	-1.31020000	-31.61020000
H	28.91830000	-0.64560000	-31.57810000
H	29.39850000	-2.32200000	-31.47320000
C	30.45590000	-1.19800000	-32.98520000
H	30.85300000	-0.19050000	-33.11290000
H	31.30980000	-1.87470000	-33.03270000
C	29.48600000	-1.51120000	-34.13250000
H	28.61860000	-0.85480000	-34.05920000
H	29.11140000	-2.52890000	-34.02060000
C	30.12570000	-1.34990000	-35.51900000
H	30.49980000	-0.33190000	-35.63080000
H	30.99240000	-2.00620000	-35.60140000
C	29.13820000	-1.65730000	-36.65300000
H	28.26600000	-1.01110000	-36.55680000
H	28.77480000	-2.67940000	-36.54710000
C	29.75140000	-1.47540000	-38.04860000
H	30.10480000	-0.45020000	-38.16050000
H	30.62890000	-2.11470000	-38.14920000
C	28.75230000	-1.79660000	-39.16850000
H	27.87260000	-1.16260000	-39.06100000
H	28.40420000	-2.82350000	-39.05830000
C	29.34580000	-1.60790000	-40.57140000
H	29.68240000	-0.57760000	-40.68860000
H	30.23170000	-2.23400000	-40.68060000
C	28.34030000	-1.94670000	-41.68030000
H	27.45310000	-1.32410000	-41.56730000
H	28.00720000	-2.97800000	-41.56400000
C	28.92140000	-1.75480000	-43.08770000
H	29.24690000	-0.72140000	-43.20870000
H	29.81290000	-2.37220000	-43.20120000
C	27.91340000	-2.10560000	-44.19030000
H	27.02160000	-1.48970000	-44.07610000
H	27.58910000	-3.13920000	-44.06870000
C	28.48970000	-1.91450000	-45.59970000
H	28.80700000	-0.87960000	-45.73120000
H	29.38500000	-2.52580000	-45.71600000
C	27.48580000	-2.27730000	-46.69870000
H	26.59990000	-1.64480000	-46.64990000
H	27.92810000	-2.15320000	-47.68730000
H	27.16070000	-3.31330000	-46.60970000
C	26.59040000	-2.68910000	-29.41330000
H	27.26960000	-3.54140000	-29.35590000
H	27.21070000	-1.80180000	-29.53940000
C	25.66970000	-2.85860000	-30.63070000
H	25.09080000	-3.77530000	-30.50900000
H	24.94770000	-2.04140000	-30.65320000

C	26.42060000	-2.90820000	-31.97000000
H	27.19650000	-3.67410000	-31.93210000
H	26.92790000	-1.95740000	-32.13310000
C	25.47350000	-3.19360000	-33.14480000
H	25.01550000	-4.17390000	-33.00680000
H	24.65700000	-2.47070000	-33.13120000
C	26.16640000	-3.14270000	-34.51430000
H	26.99730000	-3.84830000	-34.53260000
H	26.59640000	-2.15270000	-34.66580000
C	25.19640000	-3.45720000	-35.66250000
H	24.79300000	-4.46140000	-35.52870000
H	24.34520000	-2.77740000	-35.61130000
C	25.84550000	-3.34850000	-37.04970000
H	26.70280000	-4.01920000	-37.10530000
H	26.23250000	-2.33960000	-37.19240000
C	24.85910000	-3.67880000	-38.17870000
H	24.48490000	-4.69410000	-38.04540000
H	23.99180000	-3.02190000	-38.10510000
C	25.47970000	-3.54320000	-39.57610000
H	26.35150000	-4.19290000	-39.65160000
H	25.84070000	-2.52450000	-39.71770000
C	24.48320000	-3.88950000	-40.69100000
H	24.12700000	-4.91060000	-40.55260000
H	23.60680000	-3.24630000	-40.60670000
C	25.08590000	-3.74610000	-42.09520000
H	25.96640000	-4.38300000	-42.17910000
H	25.43090000	-2.72250000	-42.24140000
C	24.08370000	-4.10870000	-43.19950000
H	23.73720000	-5.13180000	-43.05160000
H	23.20270000	-3.47210000	-43.11360000
C	24.67750000	-3.97130000	-44.60780000
H	25.56090000	-4.60440000	-44.69090000
H	25.01770000	-2.94720000	-44.76200000
C	23.67290000	-4.34710000	-45.70570000
H	23.32260000	-5.36730000	-45.54710000
H	22.79310000	-3.70650000	-45.63520000
C	24.26770000	-4.23410000	-47.11300000
H	25.14020000	-4.87700000	-47.22370000
H	23.53970000	-4.53180000	-47.86800000
H	24.57460000	-3.21170000	-47.33310000
O	29.31370000	-1.26740000	-22.26910000
H	29.73170000	-1.77600000	-21.57190000
O	30.39020000	-3.58010000	-21.87230000
H	30.14300000	-4.45230000	-21.56080000
O	28.35460000	-5.32620000	-22.86200000
H	29.20060000	-5.69140000	-23.14590000
C	27.79360000	-6.59860000	-27.14340000
C	30.78140000	-6.48870000	-27.32410000
O	27.56790000	-5.39790000	-27.27810000
O	31.11670000	-7.56960000	-27.81330000
N	27.31530000	-7.50450000	-28.00360000
N	30.99190000	-5.31100000	-27.92640000
H	27.57810000	-8.48100000	-27.88710000
H	30.66450000	-4.47340000	-27.44630000
C	28.65670000	-7.07610000	-25.95580000
H	28.13940000	-6.68410000	-25.08130000
C	30.08420000	-6.43780000	-25.95540000
H	29.96000000	-5.38030000	-25.73580000
C	28.67670000	-8.61310000	-25.78040000
H	27.65800000	-8.99330000	-25.68620000
H	29.10710000	-9.07950000	-26.66010000
C	31.01260000	-7.00270000	-24.84880000
H	32.04840000	-6.72780000	-25.06060000
C	29.48110000	-8.99960000	-24.53670000
H	29.02080000	-8.50890000	-23.67790000
C	30.94140000	-8.53260000	-24.66910000
H	31.43240000	-9.03550000	-25.50290000
C	31.64350000	-5.12300000	-29.21250000

H	32.04630000	-4.10990000	-29.24140000
H	32.49350000	-5.80200000	-29.30330000
C	26.41900000	-7.23040000	-29.11690000
H	25.76820000	-8.10000000	-29.21550000
H	25.76890000	-6.38050000	-28.89920000
C	30.65890000	-5.33050000	-30.37180000
H	30.25610000	-6.34370000	-30.32890000
H	29.81020000	-4.65420000	-30.25590000
C	31.31810000	-5.10100000	-31.73740000
H	31.70060000	-4.08070000	-31.78890000
H	32.18040000	-5.76130000	-31.83860000
C	30.35100000	-5.34520000	-32.90150000
H	29.48940000	-4.68380000	-32.80220000
H	29.96850000	-6.36470000	-32.84280000
C	31.01090000	-5.12680000	-34.26920000
H	31.37960000	-4.10290000	-34.33700000
H	31.88310000	-5.77500000	-34.36190000
C	30.04670000	-5.40340000	-35.42880000
H	29.17550000	-4.75450000	-35.33820000
H	29.67790000	-6.42620000	-35.35340000
C	30.69690000	-5.19940000	-36.80360000
H	31.05020000	-4.17190000	-36.89080000
H	31.57870000	-5.83480000	-36.89050000
C	29.72960000	-5.51110000	-37.95210000
H	28.84910000	-4.87470000	-37.86610000
H	29.37610000	-6.53760000	-37.85770000
C	30.36620000	-5.31970000	-39.33480000
H	30.70930000	-4.29010000	-39.43770000
H	31.25300000	-5.94840000	-39.42080000
C	29.39200000	-5.65350000	-40.47160000
H	28.50430000	-5.02770000	-40.38210000
H	29.05220000	-6.68370000	-40.36670000
C	30.01410000	-5.46220000	-41.86090000
H	30.34950000	-4.43050000	-41.96870000
H	30.90420000	-6.08540000	-41.95270000
C	29.03300000	-5.80340000	-42.98990000
H	28.14020000	-5.18620000	-42.89160000
H	28.70410000	-6.83720000	-42.88500000
C	29.64320000	-5.60110000	-44.38320000
H	29.97300000	-4.56710000	-44.48680000
H	30.53570000	-6.21930000	-44.48470000
C	28.65700000	-5.93910000	-45.50890000
H	27.76050000	-5.32910000	-45.40020000
H	28.33590000	-6.97600000	-45.40990000
C	29.25880000	-5.72160000	-46.90390000
H	29.57880000	-4.68440000	-47.00930000
H	30.15560000	-6.33190000	-47.01640000
C	28.27430000	-6.06150000	-48.02740000
H	27.38740000	-5.43030000	-47.98070000
H	28.73380000	-5.91610000	-49.00520000
H	27.94980000	-7.09990000	-47.96620000
C	27.18920000	-7.00210000	-30.42730000
H	27.97680000	-7.74980000	-30.52180000
H	27.68620000	-6.03170000	-30.39520000
C	26.25960000	-7.08010000	-31.64730000
H	25.73800000	-8.03810000	-31.62730000
H	25.48990000	-6.31080000	-31.56890000
C	26.98840000	-6.94170000	-32.99160000
H	27.82490000	-7.64030000	-33.02970000
H	27.41300000	-5.94130000	-33.07600000
C	26.04720000	-7.20830000	-34.17520000
H	25.62660000	-8.21050000	-34.08070000
H	25.20430000	-6.51750000	-34.12930000
C	26.73500000	-7.08000000	-35.54080000
H	27.59370000	-7.75070000	-35.58180000
H	27.12400000	-6.06890000	-35.65950000
C	25.77750000	-7.39830000	-36.69770000
H	25.39440000	-8.41290000	-36.58190000

H	24.91200000	-6.73700000	-36.64290000
C	26.43580000	-7.25840000	-38.07650000
H	27.30530000	-7.91360000	-38.13240000
H	26.80650000	-6.24110000	-38.20200000
C	25.46530000	-7.59000000	-39.21830000
H	25.09450000	-8.60820000	-39.09560000
H	24.59340000	-6.93820000	-39.15360000
C	26.10690000	-7.44430000	-40.60430000
H	26.98360000	-8.08890000	-40.66780000
H	26.46500000	-6.42300000	-40.73490000
C	25.13060000	-7.78910000	-41.73680000
H	24.77100000	-8.81050000	-41.60790000
H	24.25280000	-7.14570000	-41.66810000
C	25.76280000	-7.64180000	-43.12680000
H	26.64620000	-8.27730000	-43.19200000
H	26.10980000	-6.61750000	-43.26290000
C	24.78570000	-8.00180000	-44.25380000
H	24.43600000	-9.02580000	-44.11790000
H	23.90260000	-7.36580000	-44.18650000
C	25.41470000	-7.85740000	-45.64550000
H	26.30440000	-8.48450000	-45.70570000
H	25.75260000	-6.83100000	-45.78830000
C	24.44200000	-8.23580000	-46.77060000
H	24.09620000	-9.25990000	-46.62670000
H	23.55510000	-7.60300000	-46.72060000
C	25.07530000	-8.10860000	-48.15950000
H	25.96950000	-8.72570000	-48.24050000
H	24.37960000	-8.42740000	-48.93610000
H	25.35980000	-7.07820000	-48.37200000
O	30.71020000	-6.45420000	-23.57930000
H	31.17090000	-7.05270000	-22.98700000
O	31.62820000	-8.83710000	-23.46790000
H	31.28770000	-9.66700000	-23.12860000
O	29.42720000	-10.39660000	-24.31110000
H	30.13470000	-10.83430000	-24.79710000
C	29.62270000	-3.93060000	-15.62680000
C	26.84920000	-5.14490000	-16.01060000
O	29.62780000	-2.89160000	-14.97300000
O	27.09800000	-6.32130000	-16.28720000
N	30.21320000	-5.05640000	-15.20180000
N	26.12520000	-4.79350000	-14.94110000
H	30.15190000	-5.88790000	-15.78690000
H	25.93500000	-3.80200000	-14.81350000
C	28.92720000	-3.93200000	-16.99580000
H	29.16510000	-2.95420000	-17.41830000
C	27.37510000	-3.99260000	-16.88810000
H	27.05970000	-3.07970000	-16.39680000
C	29.51000000	-4.96930000	-17.97440000
H	30.59150000	-4.84870000	-18.05730000
H	29.32880000	-5.97100000	-17.60440000
C	26.70350000	-3.99910000	-18.28060000
H	25.63940000	-4.22130000	-18.17530000
C	28.86000000	-4.82090000	-19.34610000
H	29.08030000	-3.81940000	-19.71570000
C	27.33120000	-5.01310000	-19.25400000
H	27.08490000	-6.02400000	-18.93100000
C	25.57700000	-5.72200000	-13.96250000
H	24.79160000	-5.20700000	-13.40960000
H	25.10120000	-6.56100000	-14.47380000
C	31.03810000	-5.19520000	-14.00870000
H	32.03820000	-5.47200000	-14.34390000
H	31.14200000	-4.24900000	-13.47410000
C	26.66480000	-6.22460000	-12.99950000
H	27.43980000	-6.74540000	-13.56440000
H	27.15090000	-5.36910000	-12.52850000
C	26.11650000	-7.16510000	-11.91800000
H	25.33530000	-6.65170000	-11.35670000
H	25.64430000	-8.02870000	-12.38800000

C	27.21280000	-7.64010000	-10.95460000
H	27.71160000	-6.77050000	-10.52490000
H	27.97380000	-8.18740000	-11.51280000
C	26.67210000	-8.52430000	-9.82170000
H	25.89860000	-7.98210000	-9.27700000
H	26.19120000	-9.40800000	-10.24260000
C	27.77480000	-8.95460000	-8.84450000
H	28.27410000	-8.06780000	-8.45360000
H	28.53380000	-9.52170000	-9.38390000
C	27.24480000	-9.79320000	-7.67230000
H	26.48470000	-9.22640000	-7.13410000
H	26.74880000	-10.68580000	-8.05460000
C	28.35970000	-10.20140000	-6.69930000
H	28.86330000	-9.30710000	-6.33290000
H	29.11280000	-10.77840000	-7.23570000
C	27.84610000	-11.01790000	-5.50470000
H	27.10060000	-10.43770000	-4.96050000
H	27.33600000	-11.91200000	-5.86410000
C	28.97640000	-11.42260000	-4.54820000
H	29.49010000	-10.52820000	-4.19670000
H	29.71810000	-12.00680000	-5.09260000
C	28.47880000	-12.22980000	-3.34110000
H	27.74650000	-11.64200000	-2.78710000
H	27.95650000	-13.12180000	-3.68810000
C	29.62180000	-12.63950000	-2.40230000
H	30.14630000	-11.74790000	-2.05980000
H	30.35140000	-13.22960000	-2.95660000
C	29.13480000	-13.44200000	-1.18810000
H	28.41110000	-12.84980000	-0.62750000
H	28.60460000	-14.33210000	-1.52810000
C	30.28490000	-13.85600000	-0.26010000
H	30.81600000	-12.96660000	0.07830000
H	31.00740000	-14.44890000	-0.82110000
C	29.80260000	-14.65760000	0.95660000
H	29.08570000	-14.06620000	1.52710000
H	29.26760000	-15.54680000	0.62200000
C	30.95340000	-15.07840000	1.87630000
H	31.47890000	-14.21130000	2.27500000
H	30.58430000	-15.66020000	2.72110000
H	31.67940000	-15.69240000	1.34430000
C	30.48600000	-6.27680000	-13.06590000
H	30.22910000	-7.17210000	-13.63470000
H	29.56240000	-5.91890000	-12.61150000
C	31.49510000	-6.64920000	-11.96990000
H	32.39440000	-7.04940000	-12.44050000
H	31.80220000	-5.74650000	-11.44050000
C	30.95460000	-7.67160000	-10.95860000
H	30.57750000	-8.54980000	-11.48450000
H	30.10740000	-7.23800000	-10.42750000
C	32.02900000	-8.09990000	-9.94830000
H	32.84610000	-8.59040000	-10.47880000
H	32.45730000	-7.21190000	-9.48200000
C	31.49730000	-9.03570000	-8.85280000
H	31.04960000	-9.91900000	-9.30880000
H	30.70180000	-8.53320000	-8.30270000
C	32.60100000	-9.46270000	-7.87420000
H	33.37970000	-9.99660000	-8.41970000
H	33.07550000	-8.57400000	-7.45670000
C	32.08460000	-10.34420000	-6.72810000
H	31.60160000	-11.23090000	-7.13830000
H	31.31880000	-9.80480000	-6.17060000
C	33.20780000	-10.76610000	-5.77040000
H	33.96530000	-11.32080000	-6.32470000
H	33.70450000	-9.87670000	-5.38140000
C	32.70670000	-11.62020000	-4.59750000
H	32.20400000	-12.50730000	-4.98240000
H	31.95950000	-11.06240000	-4.03300000
C	33.84460000	-12.04160000	-3.65770000

H	34.58880000	-12.60450000	-4.22150000
H	34.35330000	-11.15280000	-3.28310000
C	33.35730000	-12.88620000	-2.47260000
H	32.84320000	-13.77200000	-2.84530000
H	32.62190000	-12.32120000	-1.89960000
C	34.50640000	-13.31090000	-1.54830000
H	35.24280000	-13.87380000	-2.12230000
H	35.02080000	-12.42400000	-1.17760000
C	34.03030000	-14.15870000	-0.36120000
H	33.51270000	-15.04290000	-0.73340000
H	33.29880000	-13.59610000	0.21920000
C	35.18660000	-14.58820000	0.55210000
H	35.92560000	-15.14050000	-0.02890000
H	35.69800000	-13.70610000	0.93890000
C	34.72040000	-15.45610000	1.72520000
H	34.21780000	-16.35620000	1.37270000
H	35.56610000	-15.76820000	2.33830000
H	34.02640000	-14.91460000	2.36800000
O	26.80060000	-2.73850000	-18.92310000
H	26.57320000	-2.95350000	-19.82930000
O	26.75910000	-4.77400000	-20.52510000
H	27.31180000	-5.20170000	-21.18280000
O	29.42960000	-5.76330000	-20.23040000
H	28.83380000	-6.51970000	-20.28700000
C	30.83570000	-8.61190000	-17.07750000
C	28.11460000	-9.84570000	-17.29470000
O	30.52880000	-7.68270000	-16.33390000
O	28.30840000	-11.05350000	-17.45250000
N	31.74500000	-9.52590000	-16.72180000
N	27.48820000	-9.33490000	-16.22680000
H	31.92240000	-10.31130000	-17.34460000
H	27.39170000	-8.32130000	-16.18040000
C	30.15170000	-8.73430000	-18.45600000
H	30.37380000	-7.78700000	-18.94390000
C	28.59520000	-8.83410000	-18.34830000
H	28.23930000	-7.86690000	-18.00230000
C	30.76800000	-9.82730000	-19.35870000
H	31.83920000	-9.65660000	-19.47890000
H	30.65360000	-10.79750000	-18.88750000
C	27.89730000	-9.09040000	-19.70960000
H	26.86670000	-9.41030000	-19.54040000
C	30.09710000	-9.83540000	-20.73420000
H	30.23730000	-8.85220000	-21.18690000
C	28.59260000	-10.13840000	-20.60010000
H	28.43400000	-11.13620000	-20.19120000
C	26.93980000	-10.10350000	-15.12120000
H	26.15700000	-9.50700000	-14.65120000
H	26.46120000	-11.00950000	-15.49790000
C	32.53520000	-9.50980000	-15.50020000
H	33.50410000	-9.94430000	-15.74860000
H	32.72970000	-8.48720000	-15.17020000
C	28.02430000	-10.45240000	-14.09230000
H	28.80830000	-11.03840000	-14.57470000
H	28.49740000	-9.53600000	-13.73520000
C	27.45870000	-11.23850000	-12.90330000
H	26.68790000	-10.64490000	-12.41000000
H	26.96890000	-12.14260000	-13.26740000
C	28.54020000	-11.62040000	-11.88630000
H	29.02950000	-10.71690000	-11.52050000
H	29.30990000	-12.21110000	-12.38420000
C	27.97510000	-12.41420000	-10.70140000
H	27.21440000	-11.81980000	-10.19450000
H	27.47230000	-13.30980000	-11.06830000
C	29.06380000	-12.81700000	-9.69990000
H	29.56610000	-11.92240000	-9.33130000
H	29.82310000	-13.40770000	-10.21200000
C	28.51060000	-13.61960000	-8.51510000
H	27.76340000	-13.02480000	-7.98960000

H	27.99300000	-14.50620000	-8.88240000
C	29.61360000	-14.04270000	-7.53730000
H	30.12880000	-13.15650000	-7.16740000
H	30.36060000	-14.63210000	-8.06850000
C	29.07660000	-14.85500000	-6.35190000
H	28.33790000	-14.26340000	-5.81090000
H	28.55250000	-15.73780000	-6.71940000
C	30.19270000	-15.28720000	-5.39260000
H	30.71780000	-14.40460000	-5.02820000
H	30.92900000	-15.87880000	-5.93630000
C	29.66800000	-16.09840000	-4.20110000
H	28.93470000	-15.50540000	-3.65400000
H	29.14010000	-16.98130000	-4.56310000
C	30.79180000	-16.52960000	-3.24990000
H	31.32330000	-15.64700000	-2.89500000
H	31.52100000	-17.12740000	-3.79660000
C	30.27260000	-17.33110000	-2.04900000
H	29.54150000	-16.73320000	-1.50430000
H	29.74290000	-18.21660000	-2.40200000
C	31.39860000	-17.75520000	-1.09700000
H	31.93390000	-16.87060000	-0.75260000
H	32.12430000	-18.36010000	-1.64070000
C	30.88010000	-18.54400000	0.11310000
H	30.15500000	-17.94170000	0.66180000
H	30.34620000	-19.43150000	-0.22820000
C	32.00480000	-18.96750000	1.06320000
H	32.52610000	-18.10140000	1.47000000
H	31.61060000	-19.54140000	1.90210000
H	32.73840000	-19.59070000	0.55250000
C	31.86770000	-10.32350000	-14.38050000
H	31.48920000	-11.26130000	-14.78760000
H	31.00200000	-9.78000000	-13.99980000
C	32.84970000	-10.62110000	-13.23850000
H	33.72520000	-11.12300000	-13.65320000
H	33.20790000	-9.68310000	-12.81160000
C	32.25630000	-11.49780000	-12.12700000
H	31.80310000	-12.38850000	-12.56340000
H	31.45650000	-10.95570000	-11.62240000
C	33.32410000	-11.91710000	-11.10670000
H	34.11800000	-12.46100000	-11.62060000
H	33.78850000	-11.02590000	-10.68260000
C	32.77020000	-12.78960000	-9.97290000
H	32.28310000	-13.66970000	-10.39360000
H	32.00210000	-12.23790000	-9.43110000
C	33.87020000	-13.22720000	-8.99600000
H	34.63350000	-13.78770000	-9.53720000
H	34.36750000	-12.34440000	-8.59250000
C	33.33670000	-14.08170000	-7.83910000
H	32.83480000	-14.96270000	-8.23940000
H	32.58260000	-13.51940000	-7.28840000
C	34.45100000	-14.51460000	-6.87680000
H	35.20590000	-15.07900000	-7.42520000
H	34.95540000	-13.63050000	-6.48530000
C	33.92930000	-15.36120000	-5.70880000
H	33.41900000	-16.24190000	-6.09900000
H	33.18330000	-14.79410000	-5.15200000
C	35.05200000	-15.79520000	-4.75720000
H	35.80040000	-16.36180000	-5.31230000
H	35.56220000	-14.91230000	-4.37090000
C	34.53740000	-16.64050000	-3.58520000
H	34.02030000	-17.51860000	-3.97200000
H	33.79790000	-16.07100000	-3.02210000
C	35.66550000	-17.08090000	-2.64290000
H	36.40820000	-17.64800000	-3.20510000
H	36.18110000	-16.20110000	-2.25670000
C	35.15440000	-17.92940000	-1.47170000
H	34.63080000	-18.80260000	-1.86120000
H	34.41960000	-17.36010000	-0.90230000

C	36.28490000	-18.38170000	-0.53760000
H	37.02640000	-18.94450000	-1.10560000
H	36.80340000	-17.51030000	-0.13610000
C	35.77660000	-19.24510000	0.62090000
H	35.24790000	-20.12350000	0.25230000
H	36.60340000	-19.59060000	1.24200000
H	35.09260000	-18.68700000	1.26010000
O	27.82180000	-7.91590000	-20.49480000
H	27.60830000	-8.26650000	-21.36240000
O	27.98680000	-10.05690000	-21.87870000
H	28.59230000	-10.42270000	-22.52780000
O	30.71700000	-10.78890000	-21.57490000
H	30.24660000	-11.62800000	-21.50560000

5-beta hexamer

C	26.80080000	-1.78030000	-25.96710000
C	29.85250000	-1.72860000	-25.97880000
O	26.29660000	-0.66250000	-26.02400000
O	30.14590000	-2.86970000	-26.34550000
N	26.65080000	-2.69230000	-26.93790000
N	30.21380000	-0.63550000	-26.66110000
H	27.11480000	-3.59320000	-26.83470000
H	29.96170000	0.26540000	-26.26220000
C	27.61830000	-2.15280000	-24.72190000
H	27.06080000	-1.70930000	-23.89500000
C	29.02710000	-1.49080000	-24.70010000
H	28.86850000	-0.42000000	-24.64570000
C	27.65430000	-3.66760000	-24.44410000
H	26.64140000	-4.07270000	-24.41280000
H	28.17770000	-4.18010000	-25.24170000
C	29.82830000	-1.88430000	-23.43760000
H	30.86450000	-1.55500000	-23.54070000
C	28.36620000	-3.93750000	-23.12290000
H	27.80790000	-3.42870000	-22.33780000
C	29.81100000	-3.39680000	-23.15010000
H	30.40750000	-3.91730000	-23.89890000
C	30.94080000	-0.64110000	-27.92240000
H	31.36770000	0.35060000	-28.07200000
H	31.77940000	-1.33760000	-27.86480000
C	25.79000000	-2.56630000	-28.10670000
H	25.05430000	-3.36910000	-28.04730000
H	25.22720000	-1.63080000	-28.09770000
C	30.01570000	-1.00610000	-29.09420000
H	29.60610000	-2.00550000	-28.93830000
H	29.16430000	-0.32410000	-29.10570000
C	30.72770000	-0.96260000	-30.45230000
H	31.14530000	0.03250000	-30.60850000
H	31.57000000	-1.65560000	-30.44760000
C	29.78260000	-1.31020000	-31.61020000
H	28.91830000	-0.64560000	-31.57810000
H	29.39850000	-2.32200000	-31.47320000
C	30.45590000	-1.19800000	-32.98520000
H	30.85300000	-0.19050000	-33.11290000
H	31.30980000	-1.87470000	-33.03270000
C	29.48600000	-1.51120000	-34.13250000
H	28.61860000	-0.85480000	-34.05920000
H	29.11140000	-2.52890000	-34.02060000
C	30.12570000	-1.34990000	-35.51900000
H	30.49980000	-0.33190000	-35.63080000
H	30.99240000	-2.00620000	-35.60140000
C	29.13820000	-1.65730000	-36.65300000
H	28.26600000	-1.01110000	-36.55680000
H	28.77480000	-2.67940000	-36.54710000
C	29.75140000	-1.47540000	-38.04860000
H	30.10480000	-0.45020000	-38.16050000

H	30.62890000	-2.11470000	-38.14920000
C	28.75230000	-1.79660000	-39.16850000
H	27.87260000	-1.16260000	-39.06100000
H	28.40420000	-2.82350000	-39.05830000
C	29.34580000	-1.60790000	-40.57140000
H	29.68240000	-0.57760000	-40.68860000
H	30.23170000	-2.23400000	-40.68060000
C	28.34030000	-1.94670000	-41.68030000
H	27.45310000	-1.32410000	-41.56730000
H	28.00720000	-2.97800000	-41.56400000
C	28.92140000	-1.75480000	-43.08770000
H	29.24690000	-0.72140000	-43.20870000
H	29.81290000	-2.37220000	-43.20120000
C	27.91340000	-2.10560000	-44.19030000
H	27.02160000	-1.48970000	-44.07610000
H	27.58910000	-3.13920000	-44.06870000
C	28.48970000	-1.91450000	-45.59970000
H	28.80700000	-0.87960000	-45.73120000
H	29.38500000	-2.52580000	-45.71600000
C	27.48580000	-2.27730000	-46.69870000
H	26.59990000	-1.64480000	-46.64990000
H	27.92810000	-2.15320000	-47.68730000
H	27.16070000	-3.31330000	-46.60970000
C	26.59040000	-2.68910000	-29.41330000
H	27.26960000	-3.54140000	-29.35590000
H	27.21070000	-1.80180000	-29.53940000
C	25.66970000	-2.85860000	-30.63070000
H	25.09080000	-3.77530000	-30.50900000
H	24.94770000	-2.04140000	-30.65320000
C	26.42060000	-2.90820000	-31.97000000
H	27.19650000	-3.67410000	-31.93210000
H	26.92790000	-1.95740000	-32.13310000
C	25.47350000	-3.19360000	-33.14480000
H	25.01550000	-4.17390000	-33.00680000
H	24.65700000	-2.47070000	-33.13120000
C	26.16640000	-3.14270000	-34.51430000
H	26.99730000	-3.84830000	-34.53260000
H	26.59640000	-2.15270000	-34.66580000
C	25.19640000	-3.45720000	-35.66250000
H	24.79300000	-4.46140000	-35.52870000
H	24.34520000	-2.77740000	-35.61130000
C	25.84550000	-3.34850000	-37.04970000
H	26.70280000	-4.01920000	-37.10530000
H	26.23250000	-2.33960000	-37.19240000
C	24.85910000	-3.67880000	-38.17870000
H	24.48490000	-4.69410000	-38.04540000
H	23.99180000	-3.02190000	-38.10510000
C	25.47970000	-3.54320000	-39.57610000
H	26.35150000	-4.19290000	-39.65160000
H	25.84070000	-2.52450000	-39.71770000
C	24.48320000	-3.88950000	-40.69100000
H	24.12700000	-4.91060000	-40.55260000
H	23.60680000	-3.24630000	-40.60670000
C	25.08590000	-3.74610000	-42.09520000
H	25.96640000	-4.38300000	-42.17910000
H	25.43090000	-2.72250000	-42.24140000
C	24.08370000	-4.10870000	-43.19950000
H	23.73720000	-5.13180000	-43.05160000
H	23.20270000	-3.47210000	-43.11360000
C	24.67750000	-3.97130000	-44.60780000
H	25.56090000	-4.60440000	-44.69090000
H	25.01770000	-2.94720000	-44.76200000
C	23.67290000	-4.34710000	-45.70570000
H	23.32260000	-5.36730000	-45.54710000
H	22.79310000	-3.70650000	-45.63520000
C	24.26770000	-4.23410000	-47.11300000
H	25.14020000	-4.87700000	-47.22370000
H	23.53970000	-4.53180000	-47.86800000

H	24.57460000	-3.21170000	-47.33310000
O	29.31370000	-1.26740000	-22.26910000
H	29.73170000	-1.77600000	-21.57190000
O	30.39020000	-3.58010000	-21.87230000
H	30.14300000	-4.45230000	-21.56080000
O	28.35460000	-5.32620000	-22.86200000
H	29.20060000	-5.69140000	-23.14590000
C	26.23990000	3.18830000	-25.30930000
C	29.21680000	3.05810000	-24.80330000
O	26.20670000	4.32630000	-25.77260000
O	29.61520000	1.89310000	-24.82510000
N	25.80700000	2.12590000	-26.00010000
N	29.57590000	3.95080000	-25.73240000
H	25.95840000	1.19650000	-25.61620000
H	29.15510000	4.86810000	-25.66870000
C	26.79680000	2.97480000	-23.88660000
H	26.14900000	3.58490000	-23.25510000
C	28.23360000	3.55660000	-23.72220000
H	28.15610000	4.63680000	-23.86040000
C	26.66710000	1.52160000	-23.36460000
H	25.63180000	1.18500000	-23.43750000
H	27.25520000	0.85020000	-23.98260000
C	28.78210000	3.34320000	-22.28760000
H	29.84960000	3.57080000	-22.26510000
C	27.12240000	1.42660000	-21.90280000
H	26.45120000	2.03780000	-21.29580000
C	28.57350000	1.91870000	-21.73000000
H	29.26950000	1.24730000	-22.23120000
C	30.37640000	3.67000000	-26.91460000
H	30.89480000	4.58780000	-27.19420000
H	31.14720000	2.92800000	-26.69730000
C	25.05820000	2.16040000	-27.24850000
H	24.23640000	1.45210000	-27.13800000
H	24.59920000	3.13840000	-27.40670000
C	29.47380000	3.20030000	-28.06400000
H	29.03150000	2.23450000	-27.81290000
H	28.64120000	3.89830000	-28.16890000
C	30.21100000	3.10100000	-29.40520000
H	30.67200000	4.06350000	-29.63160000
H	31.02350000	2.37760000	-29.33590000
C	29.26230000	2.71510000	-30.54650000
H	28.41360000	3.40080000	-30.55030000
H	28.85170000	1.72170000	-30.36330000
C	29.93590000	2.75290000	-31.92370000
H	30.35390000	3.74600000	-32.09430000
H	30.77430000	2.05620000	-31.94810000
C	28.94880000	2.41800000	-33.04820000
H	28.08970000	3.08700000	-32.98250000
H	28.56340000	1.40820000	-32.90500000
C	29.57100000	2.53910000	-34.44450000
H	29.95540000	3.55040000	-34.58400000
H	30.42750000	1.86900000	-34.52540000
C	28.55770000	2.22130000	-35.55110000
H	27.69010000	2.87390000	-35.44450000
H	28.19240000	1.20180000	-35.42470000
C	29.14250000	2.38640000	-36.95920000
H	29.49870000	3.40950000	-37.08700000
H	30.01380000	1.74020000	-37.07140000
C	28.11510000	2.06190000	-38.05140000
H	27.23880000	2.69920000	-37.92550000
H	27.76880000	1.03560000	-37.92780000
C	28.67670000	2.24570000	-39.46710000
H	29.01080000	3.27630000	-39.59460000
H	29.55950000	1.61790000	-39.59290000
C	27.64460000	1.90430000	-40.55020000
H	26.75990000	2.52870000	-40.41870000
H	27.31470000	0.87310000	-40.42240000
C	28.19420000	2.09240000	-41.97030000

H	28.51310000	3.12720000	-42.10260000
H	29.08510000	1.47690000	-42.09930000
C	27.16270000	1.73300000	-43.04840000
H	26.27260000	2.35040000	-42.92060000
H	26.84240000	0.69990000	-42.91200000
C	27.71000000	1.91370000	-44.47060000
H	28.02180000	2.94870000	-44.61600000
H	28.60410000	1.30300000	-44.59940000
C	26.68340000	1.53930000	-45.54490000
H	25.79520000	2.16830000	-45.47930000
H	27.10270000	1.65870000	-46.54410000
H	26.36590000	0.50200000	-45.44150000
C	25.91740000	1.76700000	-28.46220000
H	26.50630000	0.87650000	-28.23770000
H	26.62670000	2.56680000	-28.67470000
C	25.03970000	1.49840000	-29.69380000
H	24.38910000	0.64800000	-29.48400000
H	24.38110000	2.35340000	-29.85420000
C	25.82610000	1.22740000	-30.98460000
H	26.52730000	0.40520000	-30.83500000
H	26.42140000	2.10660000	-31.23120000
C	24.88210000	0.90200000	-32.15200000
H	24.36890000	-0.03910000	-31.95110000
H	24.10510000	1.66610000	-32.20520000
C	25.58540000	0.82360000	-33.51350000
H	26.37290000	0.07020000	-33.48500000
H	26.07290000	1.77670000	-33.72020000
C	24.59580000	0.50290000	-34.64280000
H	24.16230000	-0.48360000	-34.47550000
H	23.76650000	1.21070000	-34.60550000
C	25.22970000	0.55590000	-36.03860000
H	26.06560000	-0.14140000	-36.09030000
H	25.64360000	1.55010000	-36.20940000
C	24.21380000	0.23070000	-37.14240000
H	23.82480000	-0.77660000	-36.99100000
H	23.36010000	0.90460000	-37.05780000
C	24.80580000	0.34460000	-38.55310000
H	25.66680000	-0.31750000	-38.64190000
H	25.17680000	1.35800000	-38.70950000
C	23.77920000	0.00290000	-39.64170000
H	23.41690000	-1.01440000	-39.49070000
H	22.91150000	0.65600000	-39.53970000
C	24.35060000	0.13650000	-41.05930000
H	25.22560000	-0.50550000	-41.16010000
H	24.69730000	1.15850000	-41.21600000
C	23.32230000	-0.22550000	-42.13960000
H	22.97500000	-1.24690000	-41.98080000
H	22.44630000	0.41640000	-42.03710000
C	23.88690000	-0.09400000	-43.56030000
H	24.76970000	-0.72580000	-43.65900000
H	24.22130000	0.93090000	-43.72490000
C	22.86150000	-0.47670000	-44.63620000
H	22.51990000	-1.49860000	-44.46950000
H	21.98010000	0.15970000	-44.54690000
C	23.42680000	-0.36060000	-46.05560000
H	24.29670000	-1.00340000	-46.18650000
H	22.68310000	-0.65610000	-46.79600000
H	23.72950000	0.66280000	-46.27840000
O	28.16250000	4.22000000	-21.36750000
H	28.31760000	3.79180000	-20.52660000
O	28.85450000	1.94320000	-20.33810000
H	28.60180000	1.09350000	-19.96950000
O	27.03710000	0.08770000	-21.44820000
H	27.81100000	-0.37990000	-21.77570000
C	27.79360000	-6.59860000	-27.14340000
C	30.78140000	-6.48870000	-27.32410000
O	27.56790000	-5.39790000	-27.27810000
O	31.11670000	-7.56960000	-27.81330000

N	27.31530000	-7.50450000	-28.00360000
N	30.99190000	-5.31100000	-27.92640000
H	27.57810000	-8.48100000	-27.88710000
H	30.66450000	-4.47340000	-27.44630000
C	28.65670000	-7.07610000	-25.95580000
H	28.13940000	-6.68410000	-25.08130000
C	30.08420000	-6.43780000	-25.95540000
H	29.96000000	-5.38030000	-25.73580000
C	28.67670000	-8.61310000	-25.78040000
H	27.65800000	-8.99330000	-25.68620000
H	29.10710000	-9.07950000	-26.66010000
C	31.01260000	-7.00270000	-24.84880000
H	32.04840000	-6.72780000	-25.06060000
C	29.48110000	-8.99960000	-24.53670000
H	29.02080000	-8.50890000	-23.67790000
C	30.94140000	-8.53260000	-24.66910000
H	31.43240000	-9.03550000	-25.50290000
C	31.64350000	-5.12300000	-29.21250000
H	32.04630000	-4.10990000	-29.24140000
H	32.49350000	-5.80200000	-29.30330000
C	26.41900000	-7.23040000	-29.11690000
H	25.76820000	-8.10000000	-29.21550000
H	25.76890000	-6.38050000	-28.89920000
C	30.65890000	-5.33050000	-30.37180000
H	30.25610000	-6.34370000	-30.32890000
H	29.81020000	-4.65420000	-30.25590000
C	31.31810000	-5.10100000	-31.73740000
H	31.70060000	-4.08070000	-31.78890000
H	32.18040000	-5.76130000	-31.83860000
C	30.35100000	-5.34520000	-32.90150000
H	29.48940000	-4.68380000	-32.80220000
H	29.96850000	-6.36470000	-32.84280000
C	31.01090000	-5.12680000	-34.26920000
H	31.37960000	-4.10290000	-34.33700000
H	31.88310000	-5.77500000	-34.36190000
C	30.04670000	-5.40340000	-35.42880000
H	29.17550000	-4.75450000	-35.33820000
H	29.67790000	-6.42620000	-35.35340000
C	30.69690000	-5.19940000	-36.80360000
H	31.05020000	-4.17190000	-36.89080000
H	31.57870000	-5.83480000	-36.89050000
C	29.72960000	-5.51110000	-37.95210000
H	28.84910000	-4.87470000	-37.86610000
H	29.37610000	-6.53760000	-37.85770000
C	30.36620000	-5.31970000	-39.33480000
H	30.70930000	-4.29010000	-39.43770000
H	31.25300000	-5.94840000	-39.42080000
C	29.39200000	-5.65350000	-40.47160000
H	28.50430000	-5.02770000	-40.38210000
H	29.05220000	-6.68370000	-40.36670000
C	30.01410000	-5.46220000	-41.86090000
H	30.34950000	-4.43050000	-41.96870000
H	30.90420000	-6.08540000	-41.95270000
C	29.03300000	-5.80340000	-42.98990000
H	28.14020000	-5.18620000	-42.89160000
H	28.70410000	-6.83720000	-42.88500000
C	29.64320000	-5.60110000	-44.38320000
H	29.97300000	-4.56710000	-44.48680000
H	30.53570000	-6.21930000	-44.48470000
C	28.65700000	-5.93910000	-45.50890000
H	27.76050000	-5.32910000	-45.40020000
H	28.33590000	-6.97600000	-45.40990000
C	29.25880000	-5.72160000	-46.90390000
H	29.57880000	-4.68440000	-47.00930000
H	30.15560000	-6.33190000	-47.01640000
C	28.27430000	-6.06150000	-48.02740000
H	27.38740000	-5.43030000	-47.98070000
H	28.73380000	-5.91610000	-49.00520000

H	27.94980000	-7.09990000	-47.96620000
C	27.18920000	-7.00210000	-30.42730000
H	27.97680000	-7.74980000	-30.52180000
H	27.68620000	-6.03170000	-30.39520000
C	26.25960000	-7.08010000	-31.64730000
H	25.73800000	-8.03810000	-31.62730000
H	25.48990000	-6.31080000	-31.56890000
C	26.98840000	-6.94170000	-32.99160000
H	27.82490000	-7.64030000	-33.02970000
H	27.41300000	-5.94130000	-33.07600000
C	26.04720000	-7.20830000	-34.17520000
H	25.62660000	-8.21050000	-34.08070000
H	25.20430000	-6.51750000	-34.12930000
C	26.73500000	-7.08000000	-35.54080000
H	27.59370000	-7.75070000	-35.58180000
H	27.12400000	-6.06890000	-35.65950000
C	25.77750000	-7.39830000	-36.69770000
H	25.39440000	-8.41290000	-36.58190000
H	24.91200000	-6.73700000	-36.64290000
C	26.43580000	-7.25840000	-38.07650000
H	27.30530000	-7.91360000	-38.13240000
H	26.80650000	-6.24110000	-38.20200000
C	25.46530000	-7.59000000	-39.21830000
H	25.09450000	-8.60820000	-39.09560000
H	24.59340000	-6.93820000	-39.15360000
C	26.10690000	-7.44430000	-40.60430000
H	26.98360000	-8.08890000	-40.66780000
H	26.46500000	-6.42300000	-40.73490000
C	25.13060000	-7.78910000	-41.73680000
H	24.77100000	-8.81050000	-41.60790000
H	24.25280000	-7.14570000	-41.66810000
C	25.76280000	-7.64180000	-43.12680000
H	26.64620000	-8.27730000	-43.19200000
H	26.10980000	-6.61750000	-43.26290000
C	24.78570000	-8.00180000	-44.25380000
H	24.43600000	-9.02580000	-44.11790000
H	23.90260000	-7.36580000	-44.18650000
C	25.41470000	-7.85740000	-45.64550000
H	26.30440000	-8.48450000	-45.70570000
H	25.75260000	-6.83100000	-45.78830000
C	24.44200000	-8.23580000	-46.77060000
H	24.09620000	-9.25990000	-46.62670000
H	23.55510000	-7.60300000	-46.72060000
C	25.07530000	-8.10860000	-48.15950000
H	25.96950000	-8.72570000	-48.24050000
H	24.37960000	-8.42740000	-48.93610000
H	25.35980000	-7.07820000	-48.37200000
O	30.71020000	-6.45420000	-23.57930000
H	31.17090000	-7.05270000	-22.98700000
O	31.62820000	-8.83710000	-23.46790000
H	31.28770000	-9.66700000	-23.12860000
O	29.42720000	-10.39660000	-24.31110000
H	30.13470000	-10.83430000	-24.79710000
C	29.62270000	-3.93060000	-15.62680000
C	26.84920000	-5.14490000	-16.01060000
O	29.62780000	-2.89160000	-14.97300000
O	27.09800000	-6.32130000	-16.28720000
N	30.21320000	-5.05640000	-15.20180000
N	26.12520000	-4.79350000	-14.94110000
H	30.15190000	-5.88790000	-15.78690000
H	25.93500000	-3.80200000	-14.81350000
C	28.92720000	-3.93200000	-16.99580000
H	29.16510000	-2.95420000	-17.41830000
C	27.37510000	-3.99260000	-16.88810000
H	27.05970000	-3.07970000	-16.39680000
C	29.51000000	-4.96930000	-17.97440000
H	30.59150000	-4.84870000	-18.05730000
H	29.32880000	-5.97100000	-17.60440000

C	26.70350000	-3.99910000	-18.28060000
H	25.63940000	-4.22130000	-18.17530000
C	28.86000000	-4.82090000	-19.34610000
H	29.08030000	-3.81940000	-19.71570000
C	27.33120000	-5.01310000	-19.25400000
H	27.08490000	-6.02400000	-18.93100000
C	25.57700000	-5.72200000	-13.96250000
H	24.79160000	-5.20700000	-13.40960000
H	25.10120000	-6.56100000	-14.47380000
C	31.03810000	-5.19520000	-14.00870000
H	32.03820000	-5.47200000	-14.34390000
H	31.14200000	-4.24900000	-13.47410000
C	26.66480000	-6.22460000	-12.99950000
H	27.43980000	-6.74540000	-13.56440000
H	27.15090000	-5.36910000	-12.52850000
C	26.11650000	-7.16510000	-11.91800000
H	25.33530000	-6.65170000	-11.35670000
H	25.64430000	-8.02870000	-12.38800000
C	27.21280000	-7.64010000	-10.95460000
H	27.71160000	-6.77050000	-10.52490000
H	27.97380000	-8.18740000	-11.51280000
C	26.67210000	-8.52430000	-9.82170000
H	25.89860000	-7.98210000	-9.27700000
H	26.19120000	-9.40800000	-10.24260000
C	27.77480000	-8.95460000	-8.84450000
H	28.27410000	-8.06780000	-8.45360000
H	28.53380000	-9.52170000	-9.38390000
C	27.24480000	-9.79320000	-7.67230000
H	26.48470000	-9.22640000	-7.13410000
H	26.74880000	-10.68580000	-8.05460000
C	28.35970000	-10.20140000	-6.69930000
H	28.86330000	-9.30710000	-6.33290000
H	29.11280000	-10.77840000	-7.23570000
C	27.84610000	-11.01790000	-5.50470000
H	27.10060000	-10.43770000	-4.96050000
H	27.33600000	-11.91200000	-5.86410000
C	28.97640000	-11.42260000	-4.54820000
H	29.49010000	-10.52820000	-4.19670000
H	29.71810000	-12.00680000	-5.09260000
C	28.47880000	-12.22980000	-3.34110000
H	27.74650000	-11.64200000	-2.78710000
H	27.95650000	-13.12180000	-3.68810000
C	29.62180000	-12.63950000	-2.40230000
H	30.14630000	-11.74790000	-2.05980000
H	30.35140000	-13.22960000	-2.95660000
C	29.13480000	-13.44200000	-1.18810000
H	28.41110000	-12.84980000	-0.62750000
H	28.60460000	-14.33210000	-1.52810000
C	30.28490000	-13.85600000	-0.26010000
H	30.81600000	-12.96660000	0.07830000
H	31.00740000	-14.44890000	-0.82110000
C	29.80260000	-14.65760000	0.95660000
H	29.08570000	-14.06620000	1.52710000
H	29.26760000	-15.54680000	0.62200000
C	30.95340000	-15.07840000	1.87630000
H	31.47890000	-14.21130000	2.27500000
H	30.58430000	-15.66020000	2.72110000
H	31.67940000	-15.69240000	1.34430000
C	30.48600000	-6.27680000	-13.06590000
H	30.22910000	-7.17210000	-13.63470000
H	29.56240000	-5.91890000	-12.61150000
C	31.49510000	-6.64920000	-11.96990000
H	32.39440000	-7.04940000	-12.44050000
H	31.80220000	-5.74650000	-11.44050000
C	30.95460000	-7.67160000	-10.95860000
H	30.57750000	-8.54980000	-11.48450000
H	30.10740000	-7.23800000	-10.42750000
C	32.02900000	-8.09990000	-9.94830000

H	32.84610000	-8.59040000	-10.47880000
H	32.45730000	-7.21190000	-9.48200000
C	31.49730000	-9.03570000	-8.85280000
H	31.04960000	-9.91900000	-9.30880000
H	30.70180000	-8.53320000	-8.30270000
C	32.60100000	-9.46270000	-7.87420000
H	33.37970000	-9.99660000	-8.41970000
H	33.07550000	-8.57400000	-7.45670000
C	32.08460000	-10.34420000	-6.72810000
H	31.60160000	-11.23090000	-7.13830000
H	31.31880000	-9.80480000	-6.17060000
C	33.20780000	-10.76610000	-5.77040000
H	33.96530000	-11.32080000	-6.32470000
H	33.70450000	-9.87670000	-5.38140000
C	32.70670000	-11.62020000	-4.59750000
H	32.20400000	-12.50730000	-4.98240000
H	31.95950000	-11.06240000	-4.03300000
C	33.84460000	-12.04160000	-3.65770000
H	34.58880000	-12.60450000	-4.22150000
H	34.35330000	-11.15280000	-3.28310000
C	33.35730000	-12.88620000	-2.47260000
H	32.84320000	-13.77200000	-2.84530000
H	32.62190000	-12.32120000	-1.89960000
C	34.50640000	-13.31090000	-1.54830000
H	35.24280000	-13.87380000	-2.12230000
H	35.02080000	-12.42400000	-1.17760000
C	34.03030000	-14.15870000	-0.36120000
H	33.51270000	-15.04290000	-0.73340000
H	33.29880000	-13.59610000	0.21920000
C	35.18660000	-14.58820000	0.55210000
H	35.92560000	-15.14050000	-0.02890000
H	35.69800000	-13.70610000	0.93890000
C	34.72040000	-15.45610000	1.72520000
H	34.21780000	-16.35620000	1.37270000
H	35.56610000	-15.76820000	2.33830000
H	34.02640000	-14.91460000	2.36800000
O	26.80060000	-2.73850000	-18.92310000
H	26.57320000	-2.95350000	-19.82930000
O	26.75910000	-4.77400000	-20.52510000
H	27.31180000	-5.20170000	-21.18280000
O	29.42960000	-5.76330000	-20.23040000
H	28.83380000	-6.51970000	-20.28700000
C	27.93240000	0.39800000	-13.67760000
C	25.26340000	-0.68720000	-14.57480000
O	27.52420000	1.04300000	-12.71400000
O	25.42990000	-1.75580000	-15.16380000
N	28.83970000	-0.57830000	-13.54680000
N	24.62240000	-0.61220000	-13.40330000
H	29.06680000	-1.14950000	-14.35680000
H	24.58100000	0.29720000	-12.96330000
C	27.38020000	0.72020000	-15.08180000
H	27.63250000	1.77120000	-15.23140000
C	25.82350000	0.63890000	-15.13280000
H	25.44500000	1.42540000	-14.47690000
C	28.07140000	-0.05790000	-16.23090000
H	29.15080000	0.09730000	-16.19470000
H	27.90840000	-1.12410000	-16.10940000
C	25.27160000	0.95280000	-16.54690000
H	24.21460000	0.68410000	-16.59690000
C	27.54350000	0.40340000	-17.59600000
H	27.80540000	1.45550000	-17.72670000
C	26.01440000	0.23140000	-17.69050000
H	25.74430000	-0.82380000	-17.66740000
C	24.12390000	-1.74320000	-12.63580000
H	23.29160000	-1.39300000	-12.02440000
H	23.72520000	-2.51610000	-13.29590000
C	29.60860000	-0.88120000	-12.34750000
H	30.63940000	-1.03450000	-12.66880000

H	29.62500000	-0.02970000	-11.66430000
C	25.23650000	-2.30830000	-11.74220000
H	26.02280000	-2.74080000	-12.36340000
H	25.70050000	-1.48850000	-11.19070000
C	24.72910000	-3.35530000	-10.74340000
H	23.92810000	-2.91950000	-10.14450000
H	24.29310000	-4.20190000	-11.27400000
C	25.84790000	-3.84000000	-9.81410000
H	26.32520000	-2.97510000	-9.35090000
H	26.62110000	-4.33930000	-10.39890000
C	25.34370000	-4.77850000	-8.71140000
H	24.56300000	-4.27630000	-8.13840000
H	24.88260000	-5.66140000	-9.15490000
C	26.47420000	-5.20180000	-7.76640000
H	26.96090000	-4.31060000	-7.36770000
H	27.23580000	-5.74200000	-8.32940000
C	25.98320000	-6.06810000	-6.60060000
H	25.22030000	-5.52500000	-6.04110000
H	25.50210000	-6.96710000	-6.98700000
C	27.12800000	-6.45710000	-5.65730000
H	27.62450000	-5.55380000	-5.30050000
H	27.87800000	-7.02100000	-6.21240000
C	26.65280000	-7.28040000	-4.45410000
H	25.90910000	-6.71100000	-3.89490000
H	26.15120000	-8.18340000	-4.80370000
C	27.81140000	-7.66110000	-3.52370000
H	28.31990000	-6.75590000	-3.18910000
H	28.54810000	-8.23860000	-4.08250000
C	27.35000000	-8.46520000	-2.30170000
H	26.62230000	-7.88160000	-1.73610000
H	26.83210000	-9.36590000	-2.63290000
C	28.51900000	-8.85200000	-1.38610000
H	29.04010000	-7.95040000	-1.06150000
H	29.24290000	-9.43800000	-1.95300000
C	28.06660000	-9.64990000	-0.15620000
H	27.35150000	-9.05910000	0.41770000
H	27.53590000	-10.54600000	-0.47970000
C	29.24220000	-10.04840000	0.74620000
H	29.77090000	-9.15170000	1.07220000
H	29.95790000	-10.63450000	0.16930000
C	28.79560000	-10.85350000	1.97390000
H	28.08760000	-10.26690000	2.56080000
H	28.26030000	-11.74690000	1.65130000
C	29.97320000	-11.26370000	2.86450000
H	30.50070000	-10.39050000	3.24930000
H	29.63170000	-11.84940000	3.71820000
H	30.69080000	-11.87070000	2.31320000
C	29.10170000	-2.14130000	-11.62450000
H	28.93170000	-2.94640000	-12.34100000
H	28.13940000	-1.92650000	-11.15980000
C	30.10830000	-2.60980000	-10.56260000
H	31.03930000	-2.88810000	-11.05860000
H	30.35090000	-1.77070000	-9.90860000
C	29.62220000	-3.78590000	-9.70230000
H	29.32830000	-4.62280000	-10.33740000
H	28.73190000	-3.48210000	-9.15170000
C	30.70840000	-4.23760000	-8.71380000
H	31.55590000	-4.64270000	-9.26790000
H	31.08570000	-3.36590000	-8.17700000
C	30.22360000	-5.27170000	-7.68850000
H	29.83310000	-6.15130000	-8.20060000
H	29.39600000	-4.84990000	-7.11790000
C	31.34760000	-5.68340000	-6.72630000
H	32.14420000	-6.17220000	-7.28800000
H	31.78960000	-4.78830000	-6.28650000
C	30.87360000	-6.60770000	-5.59720000
H	30.42220000	-7.50470000	-6.02060000
H	30.09210000	-6.10700000	-5.02480000

C	32.02170000	-6.99970000	-4.65640000
H	32.78770000	-7.53100000	-5.22200000
H	32.49700000	-6.09670000	-4.27080000
C	31.56130000	-7.86750000	-3.47780000
H	31.07530000	-8.76700000	-3.85500000
H	30.80930000	-7.32720000	-2.90190000
C	32.72510000	-8.25910000	-2.55670000
H	33.47130000	-8.80910000	-3.13070000
H	33.22020000	-7.35700000	-2.19470000
C	32.27600000	-9.10630000	-1.35880000
H	31.77000000	-10.00260000	-1.71710000
H	31.54220000	-8.54940000	-0.77500000
C	33.44980000	-9.50720000	-0.45470000
H	34.18390000	-10.06190000	-1.03970000
H	33.95640000	-8.60950000	-0.09750000
C	33.00880000	-10.35510000	0.74590000
H	32.49110000	-11.24570000	0.38970000
H	32.28580000	-9.79530000	1.34040000
C	34.18900000	-10.77090000	1.63460000
H	34.91710000	-11.32440000	1.04110000
H	34.70440000	-9.88210000	2.00100000
C	33.75430000	-11.63020000	2.82640000
H	33.25380000	-12.53930000	2.49450000
H	34.61470000	-11.92700000	3.42660000
H	33.06730000	-11.08720000	3.47590000
O	25.35090000	2.33490000	-16.83420000
H	25.30290000	2.36000000	-17.78870000
O	25.59540000	0.80060000	-18.92070000
H	26.14320000	0.42600000	-19.61170000
O	28.15250000	-0.33410000	-18.64210000
H	27.70550000	-1.18380000	-18.69920000
C	30.83570000	-8.61190000	-17.07750000
C	28.11460000	-9.84570000	-17.29470000
O	30.52880000	-7.68270000	-16.33390000
O	28.30840000	-11.05350000	-17.45250000
N	31.74500000	-9.52590000	-16.72180000
N	27.48820000	-9.33490000	-16.22680000
H	31.92240000	-10.31130000	-17.34460000
H	27.39170000	-8.32130000	-16.18040000
C	30.15170000	-8.73430000	-18.45600000
H	30.37380000	-7.78700000	-18.94390000
C	28.59520000	-8.83410000	-18.34830000
H	28.23930000	-7.86690000	-18.00230000
C	30.76800000	-9.82730000	-19.35870000
H	31.83920000	-9.65660000	-19.47890000
H	30.65360000	-10.79750000	-18.88750000
C	27.89730000	-9.09040000	-19.70960000
H	26.86670000	-9.41030000	-19.54040000
C	30.09710000	-9.83540000	-20.73420000
H	30.23730000	-8.85220000	-21.18690000
C	28.59260000	-10.13840000	-20.60010000
H	28.43400000	-11.13620000	-20.19120000
C	26.93980000	-10.10350000	-15.12120000
H	26.15700000	-9.50700000	-14.65120000
H	26.46120000	-11.00950000	-15.49790000
C	32.53520000	-9.50980000	-15.50020000
H	33.50410000	-9.94430000	-15.74860000
H	32.72970000	-8.48720000	-15.17020000
C	28.02430000	-10.45240000	-14.09230000
H	28.80830000	-11.03840000	-14.57470000
H	28.49740000	-9.53600000	-13.73520000
C	27.45870000	-11.23850000	-12.90330000
H	26.68790000	-10.64490000	-12.41000000
H	26.96890000	-12.14260000	-13.26740000
C	28.54020000	-11.62040000	-11.88630000
H	29.02950000	-10.71690000	-11.52050000
H	29.30990000	-12.21110000	-12.38420000
C	27.97510000	-12.41420000	-10.70140000

H	27.21440000	-11.81980000	-10.19450000
H	27.47230000	-13.30980000	-11.06830000
C	29.06380000	-12.81700000	-9.69990000
H	29.56610000	-11.92240000	-9.33130000
H	29.82310000	-13.40770000	-10.21200000
C	28.51060000	-13.61960000	-8.51510000
H	27.76340000	-13.02480000	-7.98960000
H	27.99300000	-14.50620000	-8.88240000
C	29.61360000	-14.04270000	-7.53730000
H	30.12880000	-13.15650000	-7.16740000
H	30.36060000	-14.63210000	-8.06850000
C	29.07660000	-14.85500000	-6.35190000
H	28.33790000	-14.26340000	-5.81090000
H	28.55250000	-15.73780000	-6.71940000
C	30.19270000	-15.28720000	-5.39260000
H	30.71780000	-14.40460000	-5.02820000
H	30.92900000	-15.87880000	-5.93630000
C	29.66800000	-16.09840000	-4.20110000
H	28.93470000	-15.50540000	-3.65400000
H	29.14010000	-16.98130000	-4.56310000
C	30.79180000	-16.52960000	-3.24990000
H	31.32330000	-15.64700000	-2.89500000
H	31.52100000	-17.12740000	-3.79660000
C	30.27260000	-17.33110000	-2.04900000
H	29.54150000	-16.73320000	-1.50430000
H	29.74290000	-18.21660000	-2.40200000
C	31.39860000	-17.75520000	-1.09700000
H	31.93390000	-16.87060000	-0.75260000
H	32.12430000	-18.36010000	-1.64070000
C	30.88010000	-18.54400000	0.11310000
H	30.15500000	-17.94170000	0.66180000
H	30.34620000	-19.43150000	-0.22820000
C	32.00480000	-18.96750000	1.06320000
H	32.52610000	-18.10140000	1.47000000
H	31.61060000	-19.54140000	1.90210000
H	32.73840000	-19.59070000	0.55250000
C	31.86770000	-10.32350000	-14.38050000
H	31.48920000	-11.26130000	-14.78760000
H	31.00200000	-9.78000000	-13.99980000
C	32.84970000	-10.62110000	-13.23850000
H	33.72520000	-11.12300000	-13.65320000
H	33.20790000	-9.68310000	-12.81160000
C	32.25630000	-11.49780000	-12.12700000
H	31.80310000	-12.38850000	-12.56340000
H	31.45650000	-10.95570000	-11.62240000
C	33.32410000	-11.91710000	-11.10670000
H	34.11800000	-12.46100000	-11.62060000
H	33.78850000	-11.02590000	-10.68260000
C	32.77020000	-12.78960000	-9.97290000
H	32.28310000	-13.66970000	-10.39360000
H	32.00210000	-12.23790000	-9.43110000
C	33.87020000	-13.22720000	-8.99600000
H	34.63350000	-13.78770000	-9.53720000
H	34.36750000	-12.34440000	-8.59250000
C	33.33670000	-14.08170000	-7.83910000
H	32.83480000	-14.96270000	-8.23940000
H	32.58260000	-13.51940000	-7.28840000
C	34.45100000	-14.51460000	-6.87680000
H	35.20590000	-15.07900000	-7.42520000
H	34.95540000	-13.63050000	-6.48530000
C	33.92930000	-15.36120000	-5.70880000
H	33.41900000	-16.24190000	-6.09900000
H	33.18330000	-14.79410000	-5.15200000
C	35.05200000	-15.79520000	-4.75720000
H	35.80040000	-16.36180000	-5.31230000
H	35.56220000	-14.91230000	-4.37090000
C	34.53740000	-16.64050000	-3.58520000
H	34.02030000	-17.51860000	-3.97200000

H	33.79790000	-16.07100000	-3.02210000
C	35.66550000	-17.08090000	-2.64290000
H	36.40820000	-17.64800000	-3.20510000
H	36.18110000	-16.20110000	-2.25670000
C	35.15440000	-17.92940000	-1.47170000
H	34.63080000	-18.80260000	-1.86120000
H	34.41960000	-17.36010000	-0.90230000
C	36.28490000	-18.38170000	-0.53760000
H	37.02640000	-18.94450000	-1.10560000
H	36.80340000	-17.51030000	-0.13610000
C	35.77660000	-19.24510000	0.62090000
H	35.24790000	-20.12350000	0.25230000
H	36.60340000	-19.59060000	1.24200000
H	35.09260000	-18.68700000	1.26010000
O	27.82180000	-7.91590000	-20.49480000
H	27.60830000	-8.26650000	-21.36240000
O	27.98680000	-10.05690000	-21.87870000
H	28.59230000	-10.42270000	-22.52780000
O	30.71700000	-10.78890000	-21.57490000
H	30.24660000	-11.62800000	-21.50560000

5-beta octamer

C	26.80080000	-1.78030000	-25.96710000
C	29.85250000	-1.72860000	-25.97880000
O	26.29660000	-0.66250000	-26.02400000
O	30.14590000	-2.86970000	-26.34550000
N	26.65080000	-2.69230000	-26.93790000
N	30.21380000	-0.63550000	-26.66110000
H	27.11480000	-3.59320000	-26.83470000
H	29.96170000	0.26540000	-26.26220000
C	27.61830000	-2.15280000	-24.72190000
H	27.06080000	-1.70930000	-23.89500000
C	29.02710000	-1.49080000	-24.70010000
H	28.86850000	-0.42000000	-24.64570000
C	27.65430000	-3.66760000	-24.44410000
H	26.64140000	-4.07270000	-24.41280000
H	28.17770000	-4.18010000	-25.24170000
C	29.82830000	-1.88430000	-23.43760000
H	30.86450000	-1.55500000	-23.54070000
C	28.36620000	-3.93750000	-23.12290000
H	27.80790000	-3.42870000	-22.33780000
C	29.81100000	-3.39680000	-23.15010000
H	30.40750000	-3.91730000	-23.89890000
C	30.94080000	-0.64110000	-27.92240000
H	31.36770000	0.35060000	-28.07200000
H	31.77940000	-1.33760000	-27.86480000
C	25.79000000	-2.56630000	-28.10670000
H	25.05430000	-3.36910000	-28.04730000
H	25.22720000	-1.63080000	-28.09770000
C	30.01570000	-1.00610000	-29.09420000
H	29.60610000	-2.00550000	-28.93830000
H	29.16430000	-0.32410000	-29.10570000
C	30.72770000	-0.96260000	-30.45230000
H	31.14530000	0.03250000	-30.60850000
H	31.57000000	-1.65560000	-30.44760000
C	29.78260000	-1.31020000	-31.61020000
H	28.91830000	-0.64560000	-31.57810000
H	29.39850000	-2.32200000	-31.47320000
C	30.45590000	-1.19800000	-32.98520000
H	30.85300000	-0.19050000	-33.11290000
H	31.30980000	-1.87470000	-33.03270000
C	29.48600000	-1.51120000	-34.13250000
H	28.61860000	-0.85480000	-34.05920000
H	29.11140000	-2.52890000	-34.02060000
C	30.12570000	-1.34990000	-35.51900000

H	30.49980000	-0.33190000	-35.63080000
H	30.99240000	-2.00620000	-35.60140000
C	29.13820000	-1.65730000	-36.65300000
H	28.26600000	-1.01110000	-36.55680000
H	28.77480000	-2.67940000	-36.54710000
C	29.75140000	-1.47540000	-38.04860000
H	30.10480000	-0.45020000	-38.16050000
H	30.62890000	-2.11470000	-38.14920000
C	28.75230000	-1.79660000	-39.16850000
H	27.87260000	-1.16260000	-39.06100000
H	28.40420000	-2.82350000	-39.05830000
C	29.34580000	-1.60790000	-40.57140000
H	29.68240000	-0.57760000	-40.68860000
H	30.23170000	-2.23400000	-40.68060000
C	28.34030000	-1.94670000	-41.68030000
H	27.45310000	-1.32410000	-41.56730000
H	28.00720000	-2.97800000	-41.56400000
C	28.92140000	-1.75480000	-43.08770000
H	29.24690000	-0.72140000	-43.20870000
H	29.81290000	-2.37220000	-43.20120000
C	27.91340000	-2.10560000	-44.19030000
H	27.02160000	-1.48970000	-44.07610000
H	27.58910000	-3.13920000	-44.06870000
C	28.48970000	-1.91450000	-45.59970000
H	28.80700000	-0.87960000	-45.73120000
H	29.38500000	-2.52580000	-45.71600000
C	27.48580000	-2.27730000	-46.69870000
H	26.59990000	-1.64480000	-46.64990000
H	27.92810000	-2.15320000	-47.68730000
H	27.16070000	-3.31330000	-46.60970000
C	26.59040000	-2.68910000	-29.41330000
H	27.26960000	-3.54140000	-29.35590000
H	27.21070000	-1.80180000	-29.53940000
C	25.66970000	-2.85860000	-30.63070000
H	25.09080000	-3.77530000	-30.50900000
H	24.94770000	-2.04140000	-30.65320000
C	26.42060000	-2.90820000	-31.97000000
H	27.19650000	-3.67410000	-31.93210000
H	26.92790000	-1.95740000	-32.13310000
C	25.47350000	-3.19360000	-33.14480000
H	25.01550000	-4.17390000	-33.00680000
H	24.65700000	-2.47070000	-33.13120000
C	26.16640000	-3.14270000	-34.51430000
H	26.99730000	-3.84830000	-34.53260000
H	26.59640000	-2.15270000	-34.66580000
C	25.19640000	-3.45720000	-35.66250000
H	24.79300000	-4.46140000	-35.52870000
H	24.34520000	-2.77740000	-35.61130000
C	25.84550000	-3.34850000	-37.04970000
H	26.70280000	-4.01920000	-37.10530000
H	26.23250000	-2.33960000	-37.19240000
C	24.85910000	-3.67880000	-38.17870000
H	24.48490000	-4.69410000	-38.04540000
H	23.99180000	-3.02190000	-38.10510000
C	25.47970000	-3.54320000	-39.57610000
H	26.35150000	-4.19290000	-39.65160000
H	25.84070000	-2.52450000	-39.71770000
C	24.48320000	-3.88950000	-40.69100000
H	24.12700000	-4.91060000	-40.55260000
H	23.60680000	-3.24630000	-40.60670000
C	25.08590000	-3.74610000	-42.09520000
H	25.96640000	-4.38300000	-42.17910000
H	25.43090000	-2.72250000	-42.24140000
C	24.08370000	-4.10870000	-43.19950000
H	23.73720000	-5.13180000	-43.05160000
H	23.20270000	-3.47210000	-43.11360000
C	24.67750000	-3.97130000	-44.60780000
H	25.56090000	-4.60440000	-44.69090000

H	25.01770000	-2.94720000	-44.76200000
C	23.67290000	-4.34710000	-45.70570000
H	23.32260000	-5.36730000	-45.54710000
H	22.79310000	-3.70650000	-45.63520000
C	24.26770000	-4.23410000	-47.11300000
H	25.14020000	-4.87700000	-47.22370000
H	23.53970000	-4.53180000	-47.86800000
H	24.57460000	-3.21170000	-47.33310000
O	29.31370000	-1.26740000	-22.26910000
H	29.73170000	-1.77600000	-21.57190000
O	30.39020000	-3.58010000	-21.87230000
H	30.14300000	-4.45230000	-21.56080000
O	28.35460000	-5.32620000	-22.86200000
H	29.20060000	-5.69140000	-23.14590000
C	26.23990000	3.18830000	-25.30930000
C	29.21680000	3.05810000	-24.80330000
O	26.20670000	4.32630000	-25.77260000
O	29.61520000	1.89310000	-24.82510000
N	25.80700000	2.12590000	-26.00010000
N	29.57590000	3.95080000	-25.73240000
H	25.95840000	1.19650000	-25.61620000
H	29.15510000	4.86810000	-25.66870000
C	26.79680000	2.97480000	-23.88660000
H	26.14900000	3.58490000	-23.25510000
C	28.23360000	3.55660000	-23.72220000
H	28.15610000	4.63680000	-23.86040000
C	26.66710000	1.52160000	-23.36460000
H	25.63180000	1.18500000	-23.43750000
H	27.25520000	0.85020000	-23.98260000
C	28.78210000	3.34320000	-22.28760000
H	29.84960000	3.57080000	-22.26510000
C	27.12240000	1.42660000	-21.90280000
H	26.45120000	2.03780000	-21.29580000
C	28.57350000	1.91870000	-21.73000000
H	29.26950000	1.24730000	-22.23120000
C	30.37640000	3.67000000	-26.91460000
H	30.89480000	4.58780000	-27.19420000
H	31.14720000	2.92800000	-26.69730000
C	25.05820000	2.16040000	-27.24850000
H	24.23640000	1.45210000	-27.13800000
H	24.59920000	3.13840000	-27.40670000
C	29.47380000	3.20030000	-28.06400000
H	29.03150000	2.23450000	-27.81290000
H	28.64120000	3.89830000	-28.16890000
C	30.21100000	3.10100000	-29.40520000
H	30.67200000	4.06350000	-29.63160000
H	31.02350000	2.37760000	-29.33590000
C	29.26230000	2.71510000	-30.54650000
H	28.41360000	3.40080000	-30.55030000
H	28.85170000	1.72170000	-30.36330000
C	29.93590000	2.75290000	-31.92370000
H	30.35390000	3.74600000	-32.09430000
H	30.77430000	2.05620000	-31.94810000
C	28.94880000	2.41800000	-33.04820000
H	28.08970000	3.08700000	-32.98250000
H	28.56340000	1.40820000	-32.90500000
C	29.57100000	2.53910000	-34.44450000
H	29.95540000	3.55040000	-34.58400000
H	30.42750000	1.86900000	-34.52540000
C	28.55770000	2.22130000	-35.55110000
H	27.69010000	2.87390000	-35.44450000
H	28.19240000	1.20180000	-35.42470000
C	29.14250000	2.38640000	-36.95920000
H	29.49870000	3.40950000	-37.08700000
H	30.01380000	1.74020000	-37.07140000
C	28.11510000	2.06190000	-38.05140000
H	27.23880000	2.69920000	-37.92550000
H	27.76880000	1.03560000	-37.92780000

C	28.67670000	2.24570000	-39.46710000
H	29.01080000	3.27630000	-39.59460000
H	29.55950000	1.61790000	-39.59290000
C	27.64460000	1.90430000	-40.55020000
H	26.75990000	2.52870000	-40.41870000
H	27.31470000	0.87310000	-40.42240000
C	28.19420000	2.09240000	-41.97030000
H	28.51310000	3.12720000	-42.10260000
H	29.08510000	1.47690000	-42.09930000
C	27.16270000	1.73300000	-43.04840000
H	26.27260000	2.35040000	-42.92060000
H	26.84240000	0.69990000	-42.91200000
C	27.71000000	1.91370000	-44.47060000
H	28.02180000	2.94870000	-44.61600000
H	28.60410000	1.30300000	-44.59940000
C	26.68340000	1.53930000	-45.54490000
H	25.79520000	2.16830000	-45.47930000
H	27.10270000	1.65870000	-46.54410000
H	26.36590000	0.50200000	-45.44150000
C	25.91740000	1.76700000	-28.46220000
H	26.50630000	0.87650000	-28.23770000
H	26.62670000	2.56680000	-28.67470000
C	25.03970000	1.49840000	-29.69380000
H	24.38910000	0.64800000	-29.48400000
H	24.38110000	2.35340000	-29.85420000
C	25.82610000	1.22740000	-30.98460000
H	26.52730000	0.40520000	-30.83500000
H	26.42140000	2.10660000	-31.23120000
C	24.88210000	0.90200000	-32.15200000
H	24.36890000	-0.03910000	-31.95110000
H	24.10510000	1.66610000	-32.20520000
C	25.58540000	0.82360000	-33.51350000
H	26.37290000	0.07020000	-33.48500000
H	26.07290000	1.77670000	-33.72020000
C	24.59580000	0.50290000	-34.64280000
H	24.16230000	-0.48360000	-34.47550000
H	23.76650000	1.21070000	-34.60550000
C	25.22970000	0.55590000	-36.03860000
H	26.06560000	-0.14140000	-36.09030000
H	25.64360000	1.55010000	-36.20940000
C	24.21380000	0.23070000	-37.14240000
H	23.82480000	-0.77660000	-36.99100000
H	23.36010000	0.90460000	-37.05780000
C	24.80580000	0.34460000	-38.55310000
H	25.66680000	-0.31750000	-38.64190000
H	25.17680000	1.35800000	-38.70950000
C	23.77920000	0.00290000	-39.64170000
H	23.41690000	-1.01440000	-39.49070000
H	22.91150000	0.65600000	-39.53970000
C	24.35060000	0.13650000	-41.05930000
H	25.22560000	-0.50550000	-41.16010000
H	24.69730000	1.15850000	-41.21600000
C	23.32230000	-0.22550000	-42.13960000
H	22.97500000	-1.24690000	-41.98080000
H	22.44630000	0.41640000	-42.03710000
C	23.88690000	-0.09400000	-43.56030000
H	24.76970000	-0.72580000	-43.65900000
H	24.22130000	0.93090000	-43.72490000
C	22.86150000	-0.47670000	-44.63620000
H	22.51990000	-1.49860000	-44.46950000
H	21.98010000	0.15970000	-44.54690000
C	23.42680000	-0.36060000	-46.05560000
H	24.29670000	-1.00340000	-46.18650000
H	22.68310000	-0.65610000	-46.79600000
H	23.72950000	0.66280000	-46.27840000
O	28.16250000	4.22000000	-21.36750000
H	28.31760000	3.79180000	-20.52660000
O	28.85450000	1.94320000	-20.33810000

H	28.60180000	1.09350000	-19.96950000
O	27.03710000	0.08770000	-21.44820000
H	27.81100000	-0.37990000	-21.77570000
C	28.60320000	-11.16340000	-28.97200000
C	31.62570000	-11.01390000	-29.11270000
O	27.90890000	-10.22990000	-28.58000000
O	31.93750000	-11.96130000	-29.83200000
N	28.63620000	-11.54080000	-30.25580000
N	31.83160000	-9.73790000	-29.46680000
H	29.28290000	-12.27500000	-30.50780000
H	31.54280000	-8.99690000	-28.82520000
C	29.48930000	-11.92560000	-27.97200000
H	28.97550000	-11.82270000	-27.01440000
C	30.89560000	-11.26860000	-27.78170000
H	30.75270000	-10.28720000	-27.33810000
C	29.54930000	-13.44560000	-28.27380000
H	28.53810000	-13.84950000	-28.34150000
H	30.02650000	-13.63800000	-29.23550000
C	31.77780000	-12.08290000	-26.80030000
H	32.81150000	-11.73420000	-26.85290000
C	30.32750000	-14.16470000	-27.16920000
H	29.79990000	-14.03240000	-26.22150000
C	31.75580000	-13.60370000	-27.05870000
H	32.32210000	-13.83960000	-27.96250000
C	32.38260000	-9.30720000	-30.74310000
H	32.76170000	-8.29070000	-30.63150000
H	33.23610000	-9.93530000	-31.00590000
C	27.91010000	-10.87970000	-31.33090000
H	26.85260000	-10.79970000	-31.07190000
H	28.28940000	-9.86350000	-31.44050000
C	31.31320000	-9.35510000	-31.84600000
H	30.88900000	-10.35860000	-31.88440000
H	30.49300000	-8.68000000	-31.59770000
C	31.87270000	-9.00800000	-33.22970000
H	32.21960000	-7.97430000	-33.24030000
H	32.74780000	-9.62970000	-33.42440000
C	30.84290000	-9.23020000	-34.34570000
H	29.98680000	-8.57100000	-34.19760000
H	30.46180000	-10.24990000	-34.28430000
C	31.44100000	-9.00820000	-35.73950000
H	31.78890000	-7.97900000	-35.82790000
H	32.32210000	-9.64050000	-35.85750000
C	30.44630000	-9.32040000	-36.86470000
H	29.57020000	-8.67880000	-36.76950000
H	30.09090000	-10.34590000	-36.76010000
C	31.07290000	-9.14210000	-38.25300000
H	31.42230000	-8.11540000	-38.36340000
H	31.95640000	-9.77670000	-38.33500000
C	30.10070000	-9.48050000	-39.39020000
H	29.21470000	-8.85050000	-39.31290000
H	29.75860000	-10.51000000	-39.28160000
C	30.74210000	-9.29970000	-40.77180000
H	31.08720000	-8.27080000	-40.87790000
H	31.62950000	-9.92980000	-40.84500000
C	29.78200000	-9.63880000	-41.91900000
H	28.88930000	-9.01820000	-41.84230000
H	29.44920000	-10.67250000	-41.82090000
C	30.42830000	-9.43850000	-43.29590000
H	30.76660000	-8.40610000	-43.38900000
H	31.32020000	-10.06180000	-43.37260000
C	29.47390000	-9.77010000	-44.45050000
H	28.57600000	-9.15780000	-44.36740000
H	29.14900000	-10.80760000	-44.36650000
C	30.12030000	-9.54600000	-45.82390000
H	30.45110000	-8.51000000	-45.90240000
H	31.01670000	-10.16190000	-45.90810000
C	29.16900000	-9.86860000	-46.98390000
H	28.26700000	-9.26340000	-46.89330000

H	28.85030000	-10.90910000	-46.91250000
C	29.81420000	-9.62380000	-48.35480000
H	30.13480000	-8.58390000	-48.42970000
H	30.71570000	-10.23080000	-48.44850000
C	28.86800000	-9.94490000	-49.51660000
H	27.97520000	-9.32080000	-49.48400000
H	29.35680000	-9.77180000	-50.47560000
H	28.55090000	-10.98770000	-49.49020000
C	28.08540000	-11.65170000	-32.64560000
H	27.66330000	-12.65190000	-32.53730000
H	29.14870000	-11.78790000	-32.84770000
C	27.43400000	-10.95240000	-33.84840000
H	26.36510000	-10.82860000	-33.66880000
H	27.84670000	-9.94870000	-33.95560000
C	27.65440000	-11.73670000	-35.15020000
H	27.22720000	-12.73520000	-35.04640000
H	28.72440000	-11.87840000	-35.30840000
C	27.04900000	-11.05230000	-36.38420000
H	25.97580000	-10.92000000	-36.24100000
H	27.47190000	-10.05350000	-36.49400000
C	27.30390000	-11.85960000	-37.66540000
H	26.88130000	-12.85900000	-37.55230000
H	28.37800000	-11.99570000	-37.79810000
C	26.71700000	-11.20130000	-38.92180000
H	25.64070000	-11.07470000	-38.79860000
H	27.13460000	-10.20130000	-39.03900000
C	26.99720000	-12.02320000	-40.18820000
H	26.58280000	-13.02520000	-40.06850000
H	28.07410000	-12.14950000	-40.30660000
C	26.41710000	-11.38250000	-41.45670000
H	25.33830000	-11.26710000	-41.34510000
H	26.82400000	-10.37840000	-41.57630000
C	26.71790000	-12.20710000	-42.71630000
H	26.31570000	-13.21390000	-42.59530000
H	27.79720000	-12.31920000	-42.82760000
C	26.13610000	-11.57640000	-43.98890000
H	25.05550000	-11.47400000	-43.88160000
H	26.53060000	-10.56720000	-44.10770000
C	26.45060000	-12.39670000	-45.24770000
H	26.06210000	-13.40900000	-45.12800000
H	27.53150000	-12.49310000	-45.35850000
C	25.85990000	-11.77100000	-46.51850000
H	24.77850000	-11.68050000	-46.40870000
H	26.24360000	-10.75750000	-46.63570000
C	26.17940000	-12.58420000	-47.78040000
H	25.80230000	-13.60100000	-47.66220000
H	27.26110000	-12.66830000	-47.89440000
C	25.57800000	-11.96060000	-49.04760000
H	24.49590000	-11.87650000	-48.93810000
H	25.95330000	-10.94470000	-49.17050000
C	25.89840000	-12.76970000	-50.30920000
H	25.50080000	-13.78270000	-50.24050000
H	25.46240000	-12.30170000	-51.19230000
H	26.97440000	-12.84200000	-50.47010000
O	31.35540000	-11.91600000	-25.45890000
H	31.71930000	-12.69250000	-25.02680000
O	32.33110000	-14.27080000	-25.95210000
H	32.12230000	-15.18590000	-26.11400000
O	30.42780000	-15.55310000	-27.42990000
H	29.56010000	-15.93110000	-27.40190000
C	27.79360000	-6.59860000	-27.14340000
C	30.78140000	-6.48870000	-27.32410000
O	27.56790000	-5.39790000	-27.27810000
O	31.11670000	-7.56960000	-27.81330000
N	27.31530000	-7.50450000	-28.00360000
N	30.99190000	-5.31100000	-27.92640000
H	27.57810000	-8.48100000	-27.88710000
H	30.66450000	-4.47340000	-27.44630000

C	28.65670000	-7.07610000	-25.95580000
H	28.13940000	-6.68410000	-25.08130000
C	30.08420000	-6.43780000	-25.95540000
H	29.96000000	-5.38030000	-25.73580000
C	28.67670000	-8.61310000	-25.78040000
H	27.65800000	-8.99330000	-25.68620000
H	29.10710000	-9.07950000	-26.66010000
C	31.01260000	-7.00270000	-24.84880000
H	32.04840000	-6.72780000	-25.06060000
C	29.48110000	-8.99960000	-24.53670000
H	29.02080000	-8.50890000	-23.67790000
C	30.94140000	-8.53260000	-24.66910000
H	31.43240000	-9.03550000	-25.50290000
C	31.64350000	-5.12300000	-29.21250000
H	32.04630000	-4.10990000	-29.24140000
H	32.49350000	-5.80200000	-29.30330000
C	26.41900000	-7.23040000	-29.11690000
H	25.76820000	-8.10000000	-29.21550000
H	25.76890000	-6.38050000	-28.89920000
C	30.65890000	-5.33050000	-30.37180000
H	30.25610000	-6.34370000	-30.32890000
H	29.81020000	-4.65420000	-30.25590000
C	31.31810000	-5.10100000	-31.73740000
H	31.70060000	-4.08070000	-31.78890000
H	32.18040000	-5.76130000	-31.83860000
C	30.35100000	-5.34520000	-32.90150000
H	29.48940000	-4.68380000	-32.80220000
H	29.96850000	-6.36470000	-32.84280000
C	31.01090000	-5.12680000	-34.26920000
H	31.37960000	-4.10290000	-34.33700000
H	31.88310000	-5.77500000	-34.36190000
C	30.04670000	-5.40340000	-35.42880000
H	29.17550000	-4.75450000	-35.33820000
H	29.67790000	-6.42620000	-35.35340000
C	30.69690000	-5.19940000	-36.80360000
H	31.05020000	-4.17190000	-36.89080000
H	31.57870000	-5.83480000	-36.89050000
C	29.72960000	-5.51110000	-37.95210000
H	28.84910000	-4.87470000	-37.86610000
H	29.37610000	-6.53760000	-37.85770000
C	30.36620000	-5.31970000	-39.33480000
H	30.70930000	-4.29010000	-39.43770000
H	31.25300000	-5.94840000	-39.42080000
C	29.39200000	-5.65350000	-40.47160000
H	28.50430000	-5.02770000	-40.38210000
H	29.05220000	-6.68370000	-40.36670000
C	30.01410000	-5.46220000	-41.86090000
H	30.34950000	-4.43050000	-41.96870000
H	30.90420000	-6.08540000	-41.95270000
C	29.03300000	-5.80340000	-42.98990000
H	28.14020000	-5.18620000	-42.89160000
H	28.70410000	-6.83720000	-42.88500000
C	29.64320000	-5.60110000	-44.38320000
H	29.97300000	-4.56710000	-44.48680000
H	30.53570000	-6.21930000	-44.48470000
C	28.65700000	-5.93910000	-45.50890000
H	27.76050000	-5.32910000	-45.40020000
H	28.33590000	-6.97600000	-45.40990000
C	29.25880000	-5.72160000	-46.90390000
H	29.57880000	-4.68440000	-47.00930000
H	30.15560000	-6.33190000	-47.01640000
C	28.27430000	-6.06150000	-48.02740000
H	27.38740000	-5.43030000	-47.98070000
H	28.73380000	-5.91610000	-49.00520000
H	27.94980000	-7.09990000	-47.96620000
C	27.18920000	-7.00210000	-30.42730000
H	27.97680000	-7.74980000	-30.52180000
H	27.68620000	-6.03170000	-30.39520000

C	26.25960000	-7.08010000	-31.64730000
H	25.73800000	-8.03810000	-31.62730000
H	25.48990000	-6.31080000	-31.56890000
C	26.98840000	-6.94170000	-32.99160000
H	27.82490000	-7.64030000	-33.02970000
H	27.41300000	-5.94130000	-33.07600000
C	26.04720000	-7.20830000	-34.17520000
H	25.62660000	-8.21050000	-34.08070000
H	25.20430000	-6.51750000	-34.12930000
C	26.73500000	-7.08000000	-35.54080000
H	27.59370000	-7.75070000	-35.58180000
H	27.12400000	-6.06890000	-35.65950000
C	25.77750000	-7.39830000	-36.69770000
H	25.39440000	-8.41290000	-36.58190000
H	24.91200000	-6.73700000	-36.64290000
C	26.43580000	-7.25840000	-38.07650000
H	27.30530000	-7.91360000	-38.13240000
H	26.80650000	-6.24110000	-38.20200000
C	25.46530000	-7.59000000	-39.21830000
H	25.09450000	-8.60820000	-39.09560000
H	24.59340000	-6.93820000	-39.15360000
C	26.10690000	-7.44430000	-40.60430000
H	26.98360000	-8.08890000	-40.66780000
H	26.46500000	-6.42300000	-40.73490000
C	25.13060000	-7.78910000	-41.73680000
H	24.77100000	-8.81050000	-41.60790000
H	24.25280000	-7.14570000	-41.66810000
C	25.76280000	-7.64180000	-43.12680000
H	26.64620000	-8.27730000	-43.19200000
H	26.10980000	-6.61750000	-43.26290000
C	24.78570000	-8.00180000	-44.25380000
H	24.43600000	-9.02580000	-44.11790000
H	23.90260000	-7.36580000	-44.18650000
C	25.41470000	-7.85740000	-45.64550000
H	26.30440000	-8.48450000	-45.70570000
H	25.75260000	-6.83100000	-45.78830000
C	24.44200000	-8.23580000	-46.77060000
H	24.09620000	-9.25990000	-46.62670000
H	23.55510000	-7.60300000	-46.72060000
C	25.07530000	-8.10860000	-48.15950000
H	25.96950000	-8.72570000	-48.24050000
H	24.37960000	-8.42740000	-48.93610000
H	25.35980000	-7.07820000	-48.37200000
O	30.71020000	-6.45420000	-23.57930000
H	31.17090000	-7.05270000	-22.98700000
O	31.62820000	-8.83710000	-23.46790000
H	31.28770000	-9.66700000	-23.12860000
O	29.42720000	-10.39660000	-24.31110000
H	30.13470000	-10.83430000	-24.79710000
C	29.62270000	-3.93060000	-15.62680000
C	26.84920000	-5.14490000	-16.01060000
O	29.62780000	-2.89160000	-14.97300000
O	27.09800000	-6.32130000	-16.28720000
N	30.21320000	-5.05640000	-15.20180000
N	26.12520000	-4.79350000	-14.94110000
H	30.15190000	-5.88790000	-15.78690000
H	25.93500000	-3.80200000	-14.81350000
C	28.92720000	-3.93200000	-16.99580000
H	29.16510000	-2.95420000	-17.41830000
C	27.37510000	-3.99260000	-16.88810000
H	27.05970000	-3.07970000	-16.39680000
C	29.51000000	-4.96930000	-17.97440000
H	30.59150000	-4.84870000	-18.05730000
H	29.32880000	-5.97100000	-17.60440000
C	26.70350000	-3.99910000	-18.28060000
H	25.63940000	-4.22130000	-18.17530000
C	28.86000000	-4.82090000	-19.34610000
H	29.08030000	-3.81940000	-19.71570000

C	27.33120000	-5.01310000	-19.25400000
H	27.08490000	-6.02400000	-18.93100000
C	25.57700000	-5.72200000	-13.96250000
H	24.79160000	-5.20700000	-13.40960000
H	25.10120000	-6.56100000	-14.47380000
C	31.03810000	-5.19520000	-14.00870000
H	32.03820000	-5.47200000	-14.34390000
H	31.14200000	-4.24900000	-13.47410000
C	26.66480000	-6.22460000	-12.99950000
H	27.43980000	-6.74540000	-13.56440000
H	27.15090000	-5.36910000	-12.52850000
C	26.11650000	-7.16510000	-11.91800000
H	25.33530000	-6.65170000	-11.35670000
H	25.64430000	-8.02870000	-12.38800000
C	27.21280000	-7.64010000	-10.95460000
H	27.71160000	-6.77050000	-10.52490000
H	27.97380000	-8.18740000	-11.51280000
C	26.67210000	-8.52430000	-9.82170000
H	25.89860000	-7.98210000	-9.27700000
H	26.19120000	-9.40800000	-10.24260000
C	27.77480000	-8.95460000	-8.84450000
H	28.27410000	-8.06780000	-8.45360000
H	28.53380000	-9.52170000	-9.38390000
C	27.24480000	-9.79320000	-7.67230000
H	26.48470000	-9.22640000	-7.13410000
H	26.74880000	-10.68580000	-8.05460000
C	28.35970000	-10.20140000	-6.69930000
H	28.86330000	-9.30710000	-6.33290000
H	29.11280000	-10.77840000	-7.23570000
C	27.84610000	-11.01790000	-5.50470000
H	27.10060000	-10.43770000	-4.96050000
H	27.33600000	-11.91200000	-5.86410000
C	28.97640000	-11.42260000	-4.54820000
H	29.49010000	-10.52820000	-4.19670000
H	29.71810000	-12.00680000	-5.09260000
C	28.47880000	-12.22980000	-3.34110000
H	27.74650000	-11.64200000	-2.78710000
H	27.95650000	-13.12180000	-3.68810000
C	29.62180000	-12.63950000	-2.40230000
H	30.14630000	-11.74790000	-2.05980000
H	30.35140000	-13.22960000	-2.95660000
C	29.13480000	-13.44200000	-1.18810000
H	28.41110000	-12.84980000	-0.62750000
H	28.60460000	-14.33210000	-1.52810000
C	30.28490000	-13.85600000	-0.26010000
H	30.81600000	-12.96660000	0.07830000
H	31.00740000	-14.44890000	-0.82110000
C	29.80260000	-14.65760000	0.95660000
H	29.08570000	-14.06620000	1.52710000
H	29.26760000	-15.54680000	0.62200000
C	30.95340000	-15.07840000	1.87630000
H	31.47890000	-14.21130000	2.27500000
H	30.58430000	-15.66020000	2.72110000
H	31.67940000	-15.69240000	1.34430000
C	30.48600000	-6.27680000	-13.06590000
H	30.22910000	-7.17210000	-13.63470000
H	29.56240000	-5.91890000	-12.61150000
C	31.49510000	-6.64920000	-11.96990000
H	32.39440000	-7.04940000	-12.44050000
H	31.80220000	-5.74650000	-11.44050000
C	30.95460000	-7.67160000	-10.95860000
H	30.57750000	-8.54980000	-11.48450000
H	30.10740000	-7.23800000	-10.42750000
C	32.02900000	-8.09990000	-9.94830000
H	32.84610000	-8.59040000	-10.47880000
H	32.45730000	-7.21190000	-9.48200000
C	31.49730000	-9.03570000	-8.85280000
H	31.04960000	-9.91900000	-9.30880000

H	30.70180000	-8.53320000	-8.30270000
C	32.60100000	-9.46270000	-7.87420000
H	33.37970000	-9.99660000	-8.41970000
H	33.07550000	-8.57400000	-7.45670000
C	32.08460000	-10.34420000	-6.72810000
H	31.60160000	-11.23090000	-7.13830000
H	31.31880000	-9.80480000	-6.17060000
C	33.20780000	-10.76610000	-5.77040000
H	33.96530000	-11.32080000	-6.32470000
H	33.70450000	-9.87670000	-5.38140000
C	32.70670000	-11.62020000	-4.59750000
H	32.20400000	-12.50730000	-4.98240000
H	31.95950000	-11.06240000	-4.03300000
C	33.84460000	-12.04160000	-3.65770000
H	34.58880000	-12.60450000	-4.22150000
H	34.35330000	-11.15280000	-3.28310000
C	33.35730000	-12.88620000	-2.47260000
H	32.84320000	-13.77200000	-2.84530000
H	32.62190000	-12.32120000	-1.89960000
C	34.50640000	-13.31090000	-1.54830000
H	35.24280000	-13.87380000	-2.12230000
H	35.02080000	-12.42400000	-1.17760000
C	34.03030000	-14.15870000	-0.36120000
H	33.51270000	-15.04290000	-0.73340000
H	33.29880000	-13.59610000	0.21920000
C	35.18660000	-14.58820000	0.55210000
H	35.92560000	-15.14050000	-0.02890000
H	35.69800000	-13.70610000	0.93890000
C	34.72040000	-15.45610000	1.72520000
H	34.21780000	-16.35620000	1.37270000
H	35.56610000	-15.76820000	2.33830000
H	34.02640000	-14.91460000	2.36800000
O	26.80060000	-2.73850000	-18.92310000
H	26.57320000	-2.95350000	-19.82930000
O	26.75910000	-4.77400000	-20.52510000
H	27.31180000	-5.20170000	-21.18280000
O	29.42960000	-5.76330000	-20.23040000
H	28.83380000	-6.51970000	-20.28700000
C	27.93240000	0.39800000	-13.67760000
C	25.26340000	-0.68720000	-14.57480000
O	27.52420000	1.04300000	-12.71400000
O	25.42990000	-1.75580000	-15.16380000
N	28.83970000	-0.57830000	-13.54680000
N	24.62240000	-0.61220000	-13.40330000
H	29.06680000	-1.14950000	-14.35680000
H	24.58100000	0.29720000	-12.96330000
C	27.38020000	0.72020000	-15.08180000
H	27.63250000	1.77120000	-15.23140000
C	25.82350000	0.63890000	-15.13280000
H	25.44500000	1.42540000	-14.47690000
C	28.07140000	-0.05790000	-16.23090000
H	29.15080000	0.09730000	-16.19470000
H	27.90840000	-1.12410000	-16.10940000
C	25.27160000	0.95280000	-16.54690000
H	24.21460000	0.68410000	-16.59690000
C	27.54350000	0.40340000	-17.59600000
H	27.80540000	1.45550000	-17.72670000
C	26.01440000	0.23140000	-17.69050000
H	25.74430000	-0.82380000	-17.66740000
C	24.12390000	-1.74320000	-12.63580000
H	23.29160000	-1.39300000	-12.02440000
H	23.72520000	-2.51610000	-13.29590000
C	29.60860000	-0.88120000	-12.34750000
H	30.63940000	-1.03450000	-12.66880000
H	29.62500000	-0.02970000	-11.66430000
C	25.23650000	-2.30830000	-11.74220000
H	26.02280000	-2.74080000	-12.36340000
H	25.70050000	-1.48850000	-11.19070000

C	24.72910000	-3.35530000	-10.74340000
H	23.92810000	-2.91950000	-10.14450000
H	24.29310000	-4.20190000	-11.27400000
C	25.84790000	-3.84000000	-9.81410000
H	26.32520000	-2.97510000	-9.35090000
H	26.62110000	-4.33930000	-10.39890000
C	25.34370000	-4.77850000	-8.71140000
H	24.56300000	-4.27630000	-8.13840000
H	24.88260000	-5.66140000	-9.15490000
C	26.47420000	-5.20180000	-7.76640000
H	26.96090000	-4.31060000	-7.36770000
H	27.23580000	-5.74200000	-8.32940000
C	25.98320000	-6.06810000	-6.60060000
H	25.22030000	-5.52500000	-6.04110000
H	25.50210000	-6.96710000	-6.98700000
C	27.12800000	-6.45710000	-5.65730000
H	27.62450000	-5.55380000	-5.30050000
H	27.87800000	-7.02100000	-6.21240000
C	26.65280000	-7.28040000	-4.45410000
H	25.90910000	-6.71100000	-3.89490000
H	26.15120000	-8.18340000	-4.80370000
C	27.81140000	-7.66110000	-3.52370000
H	28.31990000	-6.75590000	-3.18910000
H	28.54810000	-8.23860000	-4.08250000
C	27.35000000	-8.46520000	-2.30170000
H	26.62230000	-7.88160000	-1.73610000
H	26.83210000	-9.36590000	-2.63290000
C	28.51900000	-8.85200000	-1.38610000
H	29.04010000	-7.95040000	-1.06150000
H	29.24290000	-9.43800000	-1.95300000
C	28.06660000	-9.64990000	-0.15620000
H	27.35150000	-9.05910000	0.41770000
H	27.53590000	-10.54600000	-0.47970000
C	29.24220000	-10.04840000	0.74620000
H	29.77090000	-9.15170000	1.07220000
H	29.95790000	-10.63450000	0.16930000
C	28.79560000	-10.85350000	1.97390000
H	28.08760000	-10.26690000	2.56080000
H	28.26030000	-11.74690000	1.65130000
C	29.97320000	-11.26370000	2.86450000
H	30.50070000	-10.39050000	3.24930000
H	29.63170000	-11.84940000	3.71820000
H	30.69080000	-11.87070000	2.31320000
C	29.10170000	-2.14130000	-11.62450000
H	28.93170000	-2.94640000	-12.34100000
H	28.13940000	-1.92650000	-11.15980000
C	30.10830000	-2.60980000	-10.56260000
H	31.03930000	-2.88810000	-11.05860000
H	30.35090000	-1.77070000	-9.90860000
C	29.62220000	-3.78590000	-9.70230000
H	29.32830000	-4.62280000	-10.33740000
H	28.73190000	-3.48210000	-9.15170000
C	30.70840000	-4.23760000	-8.71380000
H	31.55590000	-4.64270000	-9.26790000
H	31.08570000	-3.36590000	-8.17700000
C	30.22360000	-5.27170000	-7.68850000
H	29.83310000	-6.15130000	-8.20060000
H	29.39600000	-4.84990000	-7.11790000
C	31.34760000	-5.68340000	-6.72630000
H	32.14420000	-6.17220000	-7.28800000
H	31.78960000	-4.78830000	-6.28650000
C	30.87360000	-6.60770000	-5.59720000
H	30.42220000	-7.50470000	-6.02060000
H	30.09210000	-6.10700000	-5.02480000
C	32.02170000	-6.99970000	-4.65640000
H	32.78770000	-7.53100000	-5.22200000
H	32.49700000	-6.09670000	-4.27080000
C	31.56130000	-7.86750000	-3.47780000

H	31.07530000	-8.76700000	-3.85500000
H	30.80930000	-7.32720000	-2.90190000
C	32.72510000	-8.25910000	-2.55670000
H	33.47130000	-8.80910000	-3.13070000
H	33.22020000	-7.35700000	-2.19470000
C	32.27600000	-9.10630000	-1.35880000
H	31.77000000	-10.00260000	-1.71710000
H	31.54220000	-8.54940000	-0.77500000
C	33.44980000	-9.50720000	-0.45470000
H	34.18390000	-10.06190000	-1.03970000
H	33.95640000	-8.60950000	-0.09750000
C	33.00880000	-10.35510000	0.74590000
H	32.49110000	-11.24570000	0.38970000
H	32.28580000	-9.79530000	1.34040000
C	34.18900000	-10.77090000	1.63460000
H	34.91710000	-11.32440000	1.04110000
H	34.70440000	-9.88210000	2.00100000
C	33.75430000	-11.63020000	2.82640000
H	33.25380000	-12.53930000	2.49450000
H	34.61470000	-11.92700000	3.42660000
H	33.06730000	-11.08720000	3.47590000
O	25.35090000	2.33490000	-16.83420000
H	25.30290000	2.36000000	-17.78870000
O	25.59540000	0.80060000	-18.92070000
H	26.14320000	0.42600000	-19.61170000
O	28.15250000	-0.33410000	-18.64210000
H	27.70550000	-1.18380000	-18.69920000
C	32.20240000	-13.30430000	-17.90310000
C	29.44130000	-14.53530000	-18.10900000
O	32.43040000	-12.12120000	-17.66760000
O	29.66520000	-15.74080000	-18.01440000
N	32.44090000	-14.26920000	-17.00600000
N	28.73720000	-13.85040000	-17.19700000
H	32.16560000	-15.21090000	-17.24870000
H	28.60400000	-12.84720000	-17.33600000
C	31.60810000	-13.70810000	-19.26380000
H	31.89940000	-12.90190000	-19.93980000
C	30.04410000	-13.73370000	-19.27430000
H	29.69540000	-12.71540000	-19.12770000
C	32.25460000	-14.99770000	-19.82600000
H	33.34090000	-14.89610000	-19.83020000
H	32.02320000	-15.86010000	-19.19920000
C	29.47500000	-14.21220000	-20.63650000
H	28.42050000	-14.47500000	-20.52660000
C	31.74520000	-15.24540000	-21.24710000
H	32.02640000	-14.39800000	-21.87730000
C	30.21730000	-15.41800000	-21.25110000
H	29.94400000	-16.33300000	-20.72030000
C	28.18770000	-14.41160000	-15.97230000
H	27.39080000	-13.75620000	-15.61900000
H	27.72980000	-15.37980000	-16.18460000
C	32.92900000	-14.02990000	-15.65450000
H	33.82990000	-13.41410000	-15.68800000
H	32.17690000	-13.46350000	-15.10480000
C	29.27500000	-14.55010000	-14.89550000
H	30.09220000	-15.15080000	-15.29450000
H	29.69420000	-13.57050000	-14.66120000
C	28.75680000	-15.21440000	-13.61580000
H	28.00240000	-14.58020000	-13.14920000
H	28.25640000	-16.14770000	-13.87850000
C	29.88290000	-15.51500000	-12.61770000
H	30.35590000	-14.58520000	-12.29960000
H	30.65620000	-16.10070000	-13.11540000
C	29.37990000	-16.29290000	-11.39640000
H	28.63600000	-15.69840000	-10.86590000
H	28.86760000	-17.19590000	-11.73170000
C	30.51160000	-16.68630000	-10.43880000
H	31.01710000	-15.79020000	-10.07850000

H	31.25940000	-17.26620000	-10.98060000
C	29.99840000	-17.50810000	-9.25010000
H	29.25540000	-16.92800000	-8.70250000
H	29.48220000	-18.39500000	-9.62020000
C	31.12040000	-17.93810000	-8.29700000
H	31.64020000	-17.05620000	-7.92290000
H	31.85910000	-18.52280000	-8.84600000
C	30.59130000	-18.76330000	-7.11720000
H	29.84910000	-18.17970000	-6.57190000
H	30.07090000	-19.64430000	-7.49550000
C	31.70320000	-19.20350000	-6.15670000
H	32.23090000	-18.32590000	-5.78360000
H	32.43800000	-19.79820000	-6.70010000
C	31.16110000	-20.01650000	-4.97400000
H	30.42070000	-19.42300000	-4.43680000
H	30.63610000	-20.89630000	-5.34850000
C	32.26540000	-20.45650000	-4.00410000
H	32.79820000	-19.57920000	-3.63730000
H	32.99800000	-21.06160000	-4.53890000
C	31.71350000	-21.25360000	-2.81480000
H	30.97570000	-20.64940000	-2.28600000
H	31.18360000	-22.13350000	-3.18220000
C	32.81200000	-21.69120000	-1.83690000
H	33.34850000	-20.81360000	-1.47620000
H	33.54350000	-22.30420000	-2.36470000
C	32.25310000	-22.47620000	-0.64230000
H	31.52110000	-21.86590000	-0.11160000
H	31.71740000	-23.35600000	-1.00120000
C	33.34700000	-22.91730000	0.33590000
H	33.87180000	-22.05950000	0.75600000
H	32.92200000	-23.48280000	1.16550000
H	34.08270000	-23.55380000	-0.15630000
C	33.21370000	-15.35730000	-14.93840000
H	34.00250000	-15.89350000	-15.46810000
H	32.32820000	-15.99270000	-14.98220000
C	33.62240000	-15.16910000	-13.46910000
H	34.52050000	-14.55230000	-13.41280000
H	32.84080000	-14.62310000	-12.93980000
C	33.87100000	-16.51180000	-12.76620000
H	34.66580000	-17.04940000	-13.28550000
H	32.97820000	-17.13320000	-12.84940000
C	34.24410000	-16.36150000	-11.28410000
H	35.14410000	-15.75210000	-11.19230000
H	33.45300000	-15.82480000	-10.75980000
C	34.47140000	-17.72300000	-10.61100000
H	35.26370000	-18.25770000	-11.13700000
H	33.57200000	-18.33150000	-10.71420000
C	34.83800000	-17.60850000	-9.12460000
H	35.74270000	-17.00890000	-9.01690000
H	34.04900000	-17.07530000	-8.59430000
C	35.05120000	-18.98350000	-8.47470000
H	35.83970000	-19.51690000	-9.00730000
H	34.14630000	-19.58190000	-8.58800000
C	35.41670000	-18.89090000	-6.98680000
H	36.32670000	-18.30080000	-6.87170000
H	34.63230000	-18.35540000	-6.45200000
C	35.61650000	-20.27290000	-6.34820000
H	36.39970000	-20.81020000	-6.88480000
H	34.70580000	-20.86170000	-6.46390000
C	35.98440000	-20.18950000	-4.86040000
H	36.89910000	-19.60660000	-4.74490000
H	35.20510000	-19.64900000	-4.32310000
C	36.17490000	-21.57340000	-4.22390000
H	36.95170000	-22.11720000	-4.76340000
H	35.25870000	-22.15480000	-4.33500000
C	36.54980000	-21.48940000	-2.73800000
H	37.46730000	-20.90990000	-2.62820000
H	35.77470000	-20.94470000	-2.19900000

C	36.73840000	-22.87150000	-2.09770000
H	37.51000000	-23.42020000	-2.63950000
H	35.81890000	-23.44930000	-2.20200000
C	37.12130000	-22.78280000	-0.61380000
H	38.04020000	-22.20500000	-0.50500000
H	36.35080000	-22.23850000	-0.06800000
C	37.31120000	-24.16190000	0.02720000
H	38.10580000	-24.72140000	-0.46740000
H	37.57800000	-24.06770000	1.08010000
H	36.39770000	-24.75440000	-0.03120000
O	29.53160000	-13.19320000	-21.61410000
H	29.45430000	-13.69240000	-22.43030000
O	29.87130000	-15.53200000	-22.62060000
H	30.50310000	-16.16320000	-22.95300000
O	32.31290000	-16.41800000	-21.80220000
H	33.24470000	-16.28430000	-21.90570000
C	30.83570000	-8.61190000	-17.07750000
C	28.11460000	-9.84570000	-17.29470000
O	30.52880000	-7.68270000	-16.33390000
O	28.30840000	-11.05350000	-17.45250000
N	31.74500000	-9.52590000	-16.72180000
N	27.48820000	-9.33490000	-16.22680000
H	31.92240000	-10.31130000	-17.34460000
H	27.39170000	-8.32130000	-16.18040000
C	30.15170000	-8.73430000	-18.45600000
H	30.37380000	-7.78700000	-18.94390000
C	28.59520000	-8.83410000	-18.34830000
H	28.23930000	-7.86690000	-18.00230000
C	30.76800000	-9.82730000	-19.35870000
H	31.83920000	-9.65660000	-19.47890000
H	30.65360000	-10.79750000	-18.88750000
C	27.89730000	-9.09040000	-19.70960000
H	26.86670000	-9.41030000	-19.54040000
C	30.09710000	-9.83540000	-20.73420000
H	30.23730000	-8.85220000	-21.18690000
C	28.59260000	-10.13840000	-20.60010000
H	28.43400000	-11.13620000	-20.19120000
C	26.93980000	-10.10350000	-15.12120000
H	26.15700000	-9.50700000	-14.65120000
H	26.46120000	-11.00950000	-15.49790000
C	32.53520000	-9.50980000	-15.50020000
H	33.50410000	-9.94430000	-15.74860000
H	32.72970000	-8.48720000	-15.17020000
C	28.02430000	-10.45240000	-14.09230000
H	28.80830000	-11.03840000	-14.57470000
H	28.49740000	-9.53600000	-13.73520000
C	27.45870000	-11.23850000	-12.90330000
H	26.68790000	-10.64490000	-12.41000000
H	26.96890000	-12.14260000	-13.26740000
C	28.54020000	-11.62040000	-11.88630000
H	29.02950000	-10.71690000	-11.52050000
H	29.30990000	-12.21110000	-12.38420000
C	27.97510000	-12.41420000	-10.70140000
H	27.21440000	-11.81980000	-10.19450000
H	27.47230000	-13.30980000	-11.06830000
C	29.06380000	-12.81700000	-9.69990000
H	29.56610000	-11.92240000	-9.33130000
H	29.82310000	-13.40770000	-10.21200000
C	28.51060000	-13.61960000	-8.51510000
H	27.76340000	-13.02480000	-7.98960000
H	27.99300000	-14.50620000	-8.88240000
C	29.61360000	-14.04270000	-7.53730000
H	30.12880000	-13.15650000	-7.16740000
H	30.36060000	-14.63210000	-8.06850000
C	29.07660000	-14.85500000	-6.35190000
H	28.33790000	-14.26340000	-5.81090000
H	28.55250000	-15.73780000	-6.71940000
C	30.19270000	-15.28720000	-5.39260000

H	30.71780000	-14.40460000	-5.02820000
H	30.92900000	-15.87880000	-5.93630000
C	29.66800000	-16.09840000	-4.20110000
H	28.93470000	-15.50540000	-3.65400000
H	29.14010000	-16.98130000	-4.56310000
C	30.79180000	-16.52960000	-3.24990000
H	31.32330000	-15.64700000	-2.89500000
H	31.52100000	-17.12740000	-3.79660000
C	30.27260000	-17.33110000	-2.04900000
H	29.54150000	-16.73320000	-1.50430000
H	29.74290000	-18.21660000	-2.40200000
C	31.39860000	-17.75520000	-1.09700000
H	31.93390000	-16.87060000	-0.75260000
H	32.12430000	-18.36010000	-1.64070000
C	30.88010000	-18.54400000	0.11310000
H	30.15500000	-17.94170000	0.66180000
H	30.34620000	-19.43150000	-0.22820000
C	32.00480000	-18.96750000	1.06320000
H	32.52610000	-18.10140000	1.47000000
H	31.61060000	-19.54140000	1.90210000
H	32.73840000	-19.59070000	0.55250000
C	31.86770000	-10.32350000	-14.38050000
H	31.48920000	-11.26130000	-14.78760000
H	31.00200000	-9.78000000	-13.99980000
C	32.84970000	-10.62110000	-13.23850000
H	33.72520000	-11.12300000	-13.65320000
H	33.20790000	-9.68310000	-12.81160000
C	32.25630000	-11.49780000	-12.12700000
H	31.80310000	-12.38850000	-12.56340000
H	31.45650000	-10.95570000	-11.62240000
C	33.32410000	-11.91710000	-11.10670000
H	34.11800000	-12.46100000	-11.62060000
H	33.78850000	-11.02590000	-10.68260000
C	32.77020000	-12.78960000	-9.97290000
H	32.28310000	-13.66970000	-10.39360000
H	32.00210000	-12.23790000	-9.43110000
C	33.87020000	-13.22720000	-8.99600000
H	34.63350000	-13.78770000	-9.53720000
H	34.36750000	-12.34440000	-8.59250000
C	33.33670000	-14.08170000	-7.83910000
H	32.83480000	-14.96270000	-8.23940000
H	32.58260000	-13.51940000	-7.28840000
C	34.45100000	-14.51460000	-6.87680000
H	35.20590000	-15.07900000	-7.42520000
H	34.95540000	-13.63050000	-6.48530000
C	33.92930000	-15.36120000	-5.70880000
H	33.41900000	-16.24190000	-6.09900000
H	33.18330000	-14.79410000	-5.15200000
C	35.05200000	-15.79520000	-4.75720000
H	35.80040000	-16.36180000	-5.31230000
H	35.56220000	-14.91230000	-4.37090000
C	34.53740000	-16.64050000	-3.58520000
H	34.02030000	-17.51860000	-3.97200000
H	33.79790000	-16.07100000	-3.02210000
C	35.66550000	-17.08090000	-2.64290000
H	36.40820000	-17.64800000	-3.20510000
H	36.18110000	-16.20110000	-2.25670000
C	35.15440000	-17.92940000	-1.47170000
H	34.63080000	-18.80260000	-1.86120000
H	34.41960000	-17.36010000	-0.90230000
C	36.28490000	-18.38170000	-0.53760000
H	37.02640000	-18.94450000	-1.10560000
H	36.80340000	-17.51030000	-0.13610000
C	35.77660000	-19.24510000	0.62090000
H	35.24790000	-20.12350000	0.25230000
H	36.60340000	-19.59060000	1.24200000
H	35.09260000	-18.68700000	1.26010000
O	27.82180000	-7.91590000	-20.49480000

H	27.60830000	-8.26650000	-21.36240000
O	27.98680000	-10.05690000	-21.87870000
H	28.59230000	-10.42270000	-22.52780000
O	30.71700000	-10.78890000	-21.57490000
H	30.24660000	-11.62800000	-21.50560000
